# Supplementary figures and images for: Neurons enhance blood–brain barrier function via upregulating claudin-5 and VE-cadherin expression due to glial cell line-derived neurotrophic factor secretion (part 1 of 2)
Source: eLife. 2024 Oct 30;13:RP96161. doi: 10.7554/eLife.96161 (PMC11524583; doi:10.7554/eLife.96161)

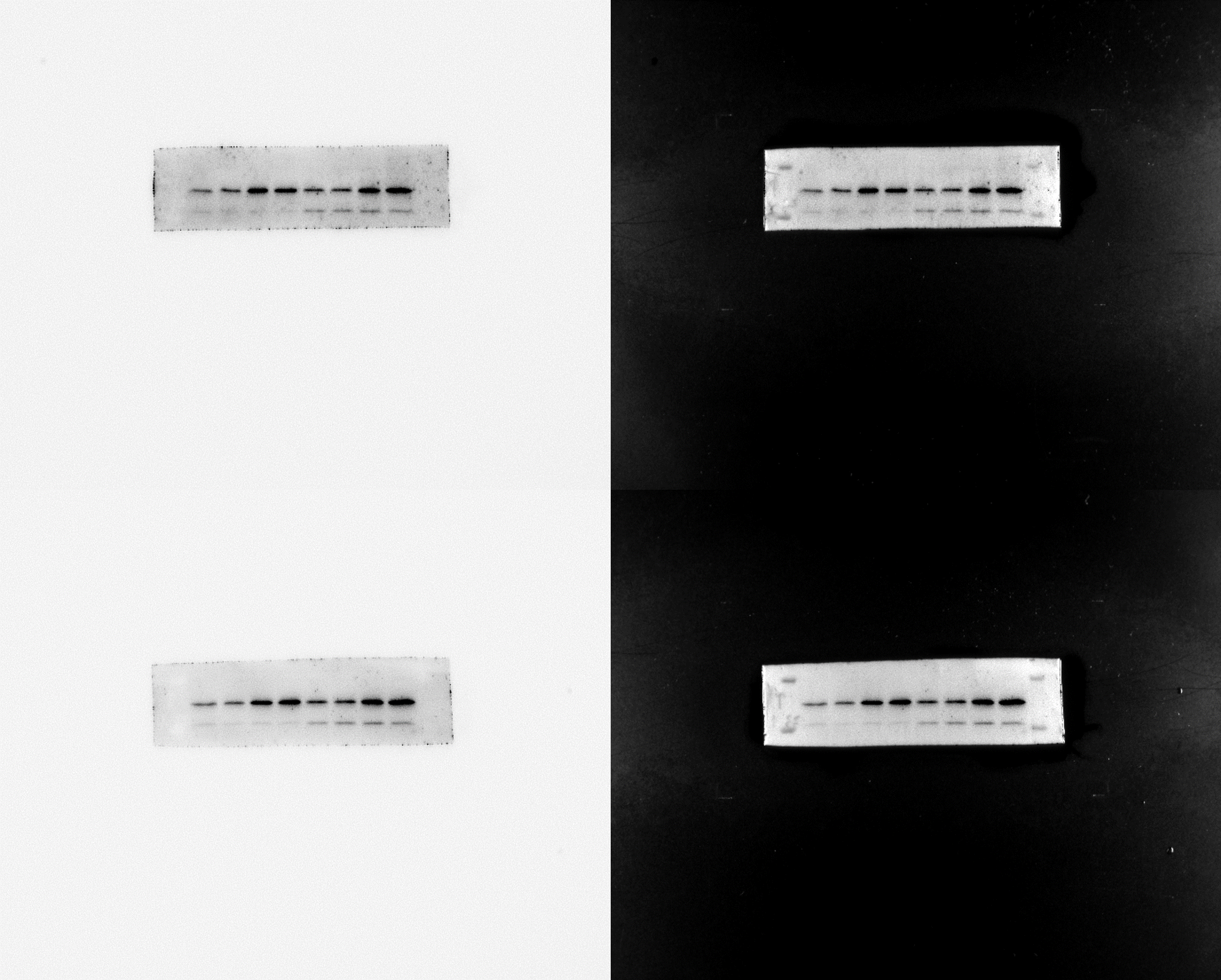

Supplement: Figure 1—source data 1. [file elife-96161-fig1-data1.zip › Figure 1-Source data1/Figure 1K-Source data1-Claudin-5.png]

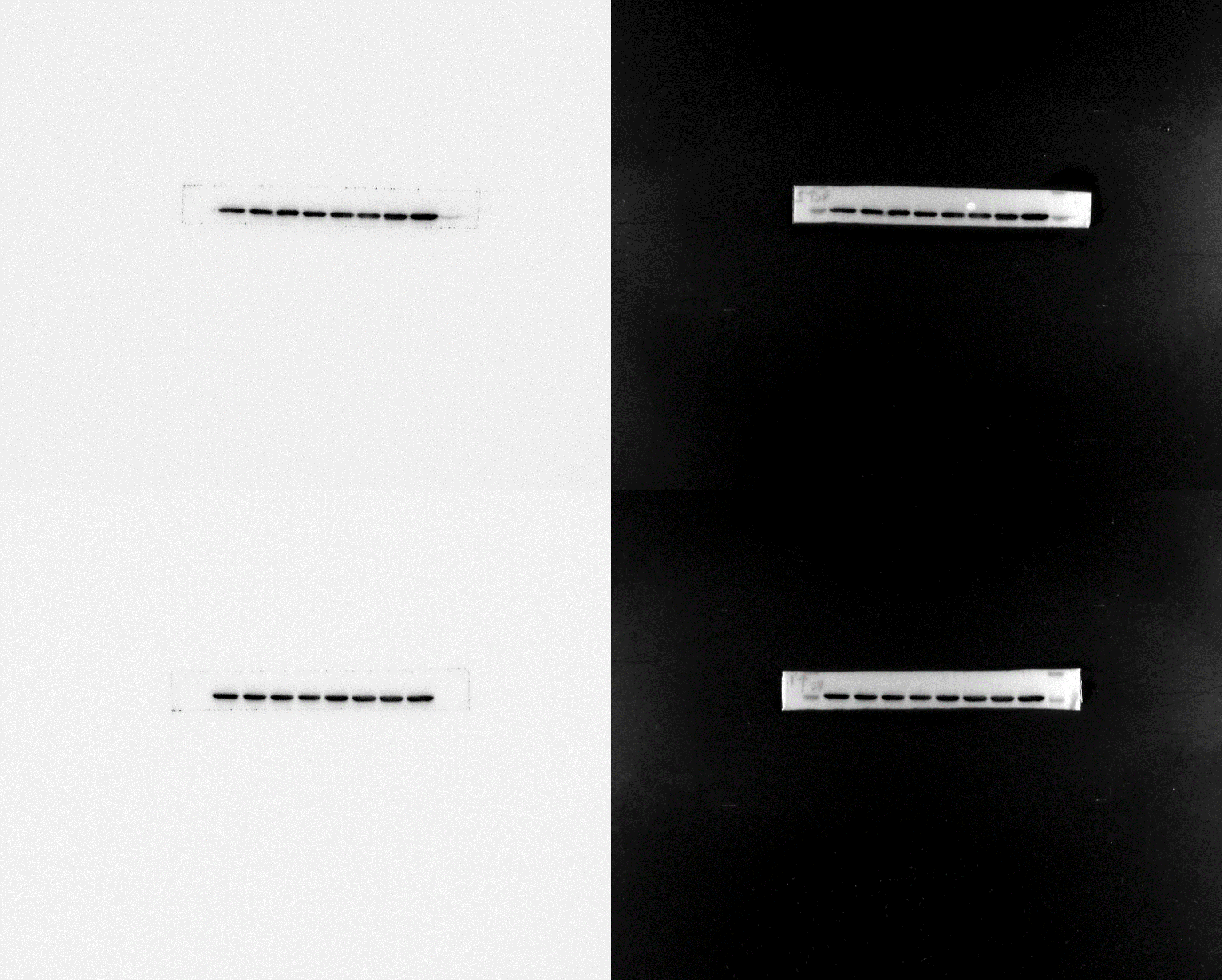

Supplement: Figure 1—source data 1. [file elife-96161-fig1-data1.zip › Figure 1-Source data1/Figure 1K-Source data1-a┬-actin.png]

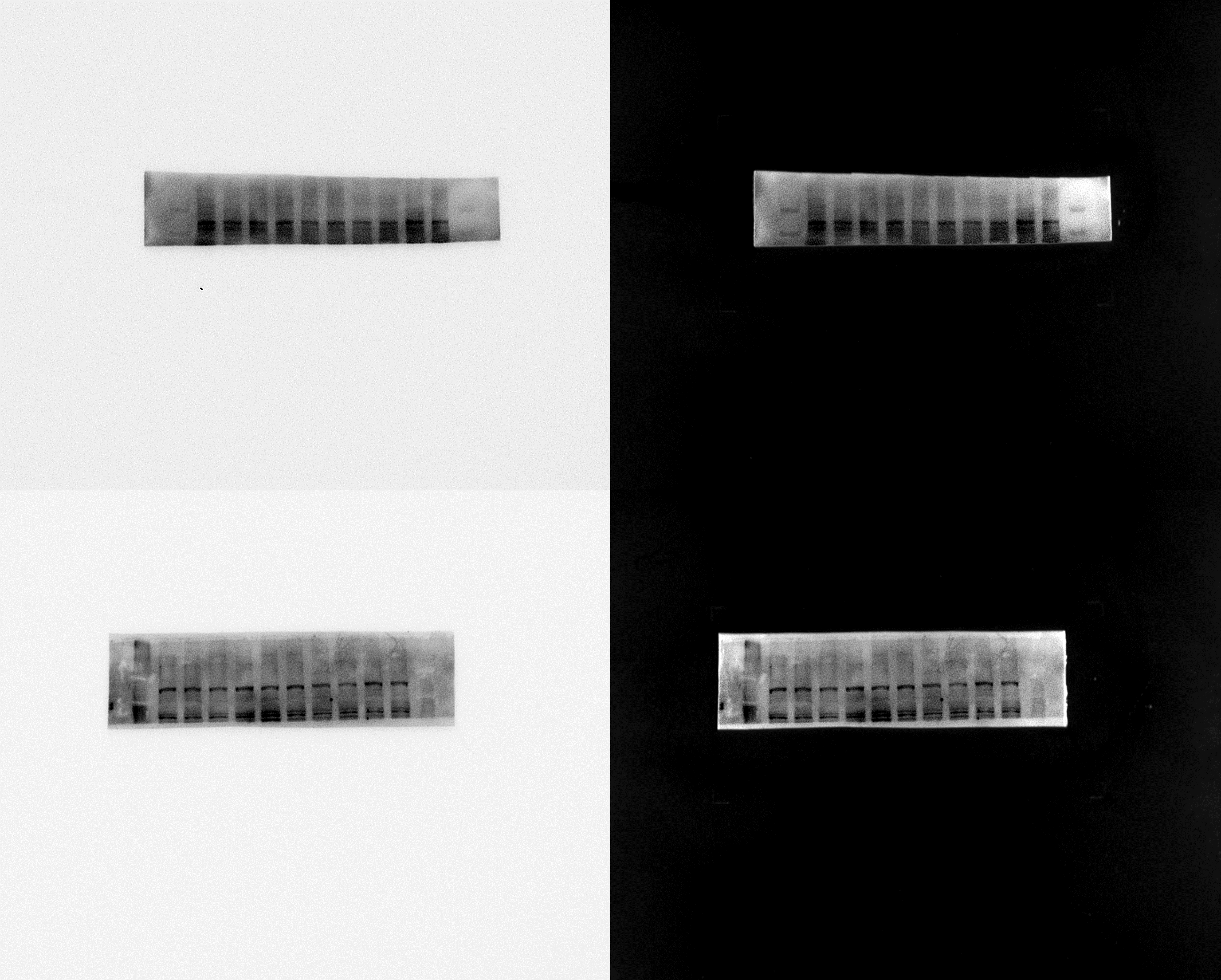

Supplement: Figure 1—source data 1. [file elife-96161-fig1-data1.zip › Figure 1-Source data1/Figure 1K-Source data2-ZO-1.png]

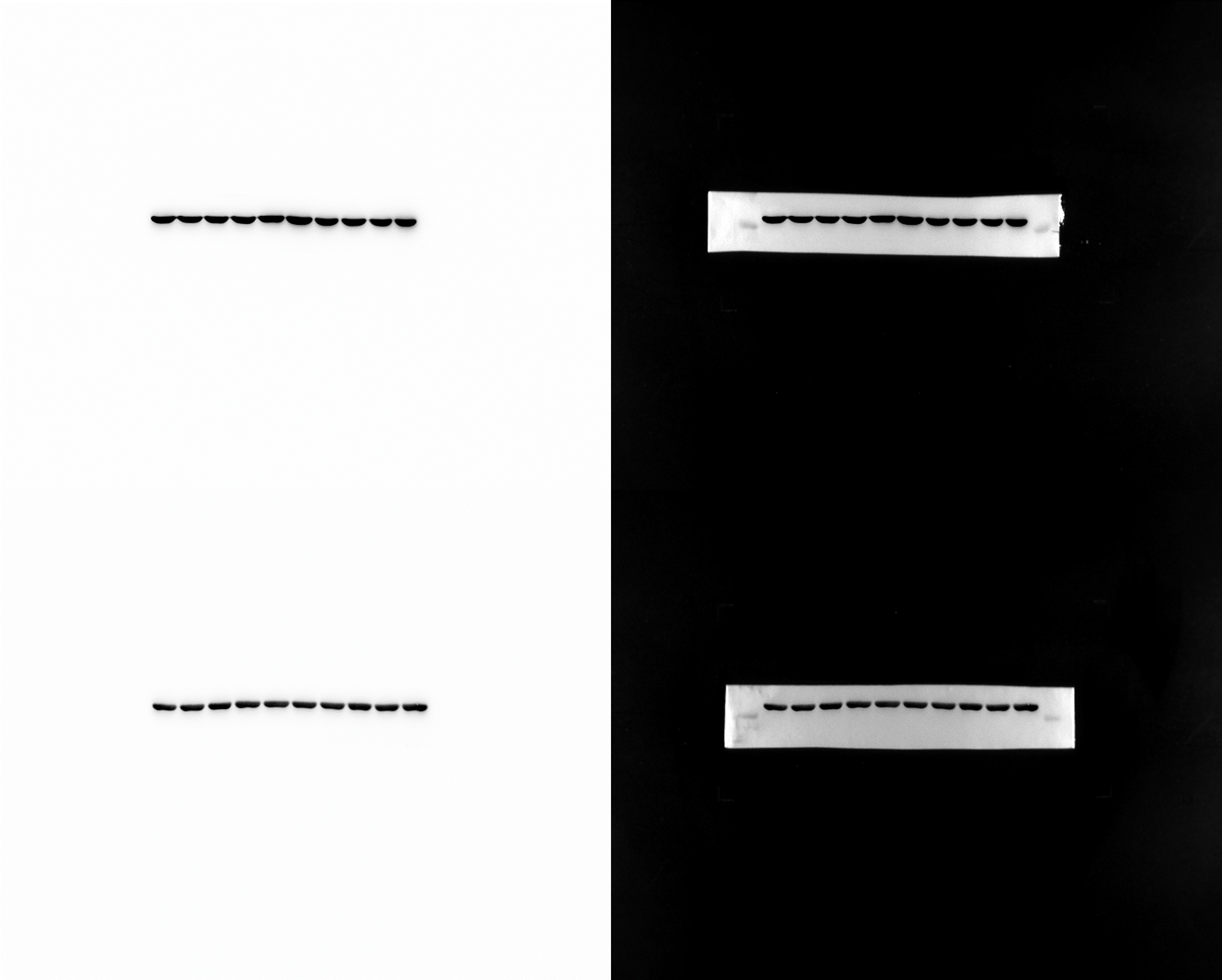

Supplement: Figure 1—source data 1. [file elife-96161-fig1-data1.zip › Figure 1-Source data1/Figure 1K-Source data2-a┬-actin.png]

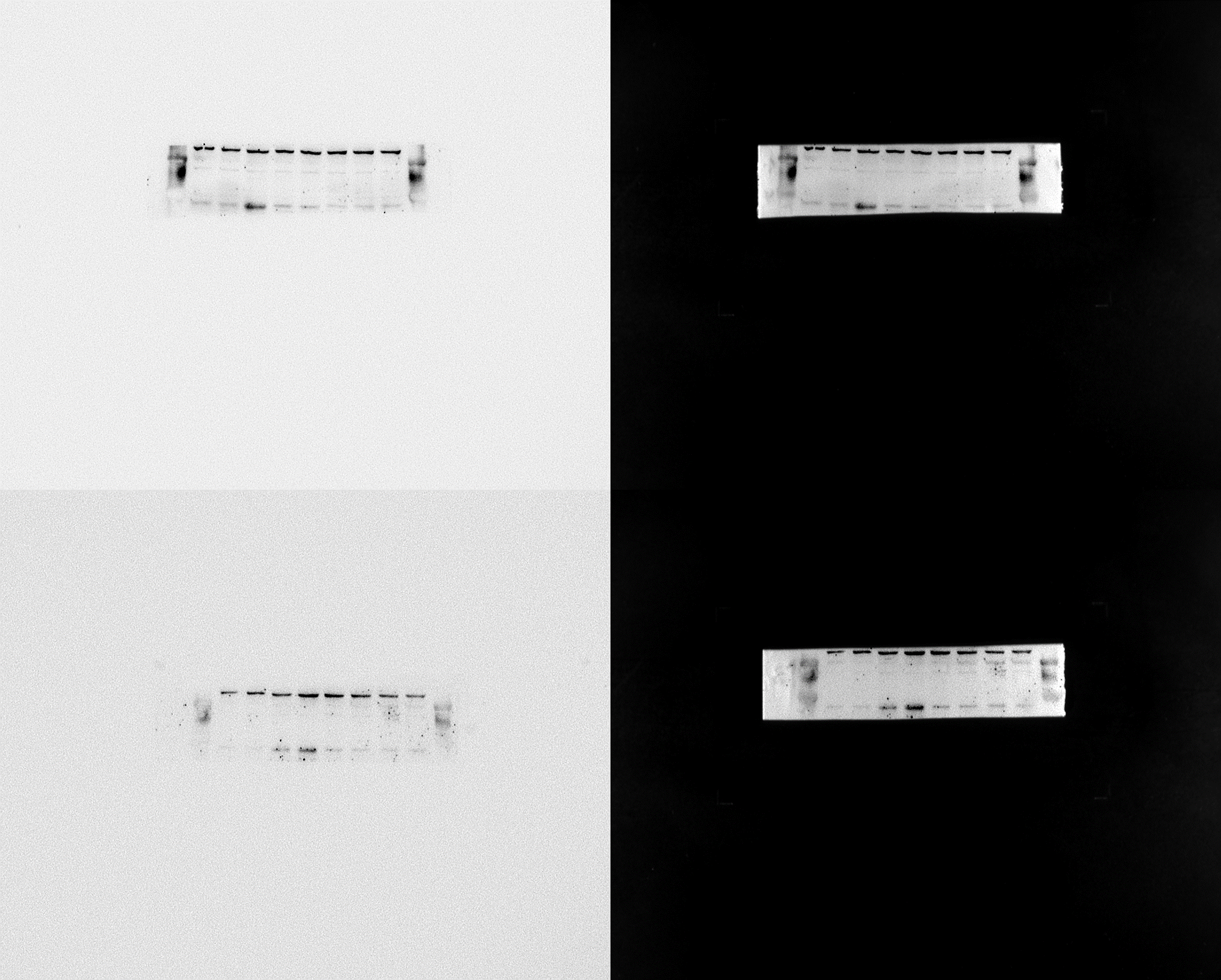

Supplement: Figure 1—source data 1. [file elife-96161-fig1-data1.zip › Figure 1-Source data1/Figure 1K-Source data3-Occludin.png]

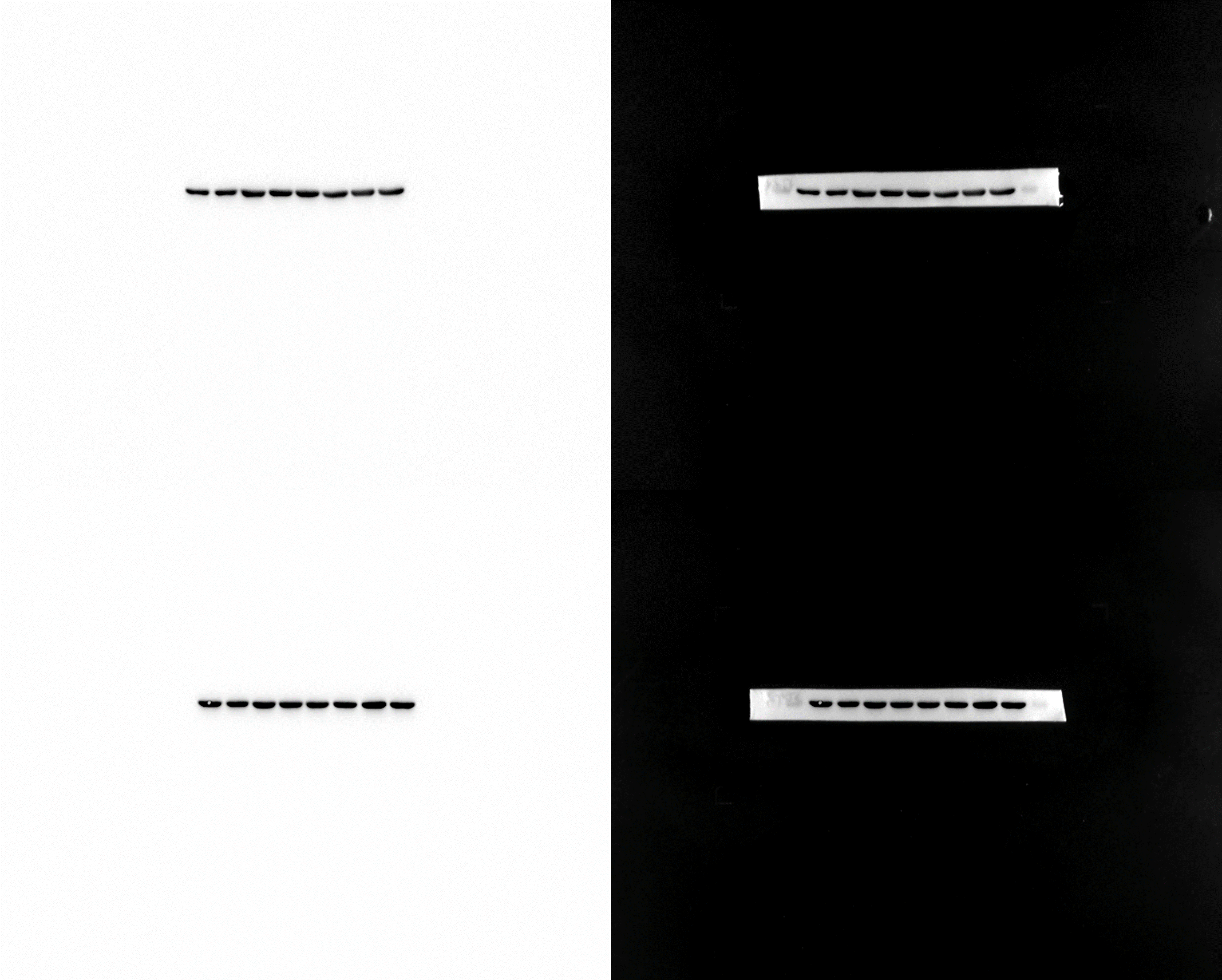

Supplement: Figure 1—source data 1. [file elife-96161-fig1-data1.zip › Figure 1-Source data1/Figure 1K-Source data3-a┬-actin.png]

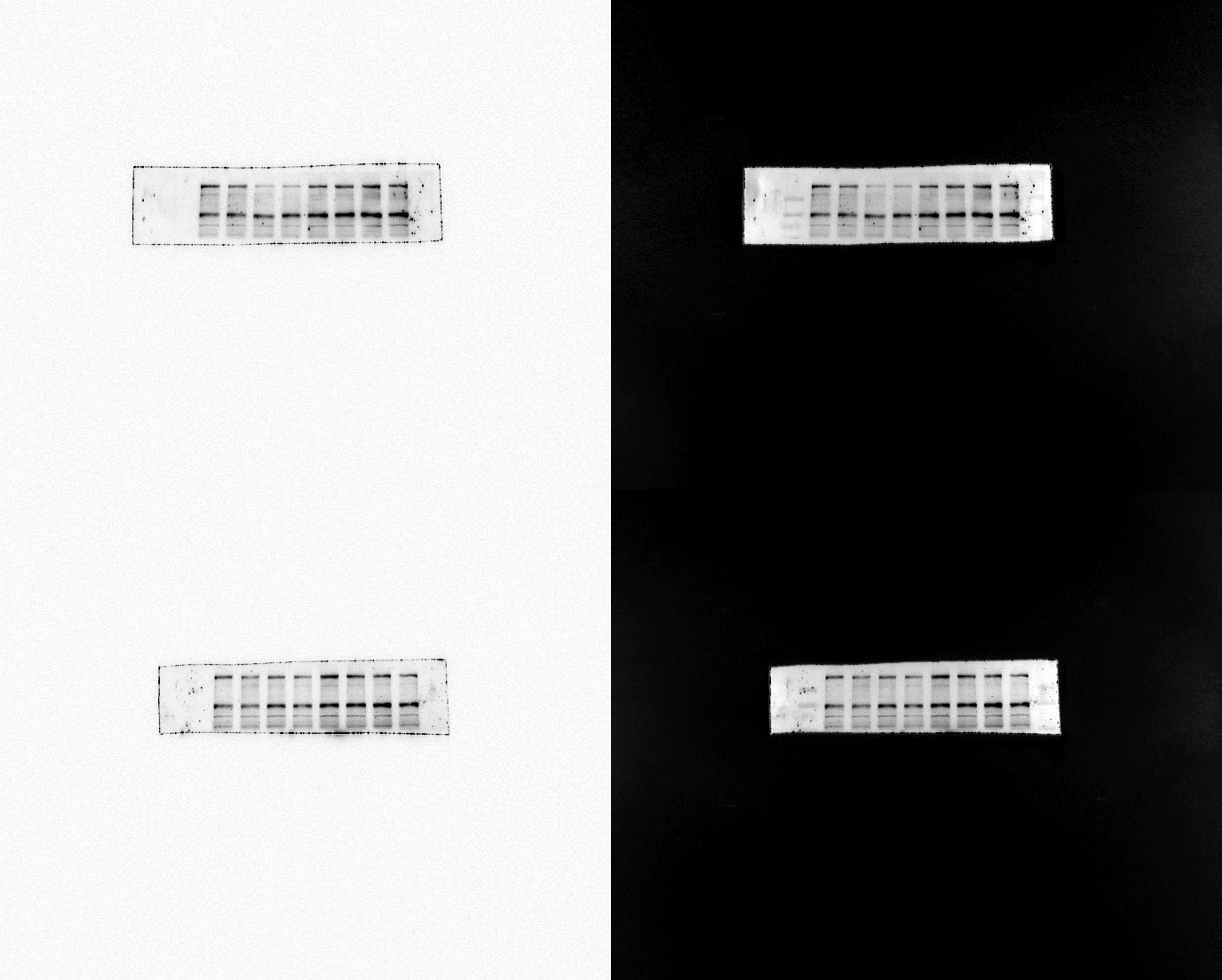

Supplement: Figure 1—source data 1. [file elife-96161-fig1-data1.zip › Figure 1-Source data1/Figure 1M-Source data1-VE-Cadherin.png]

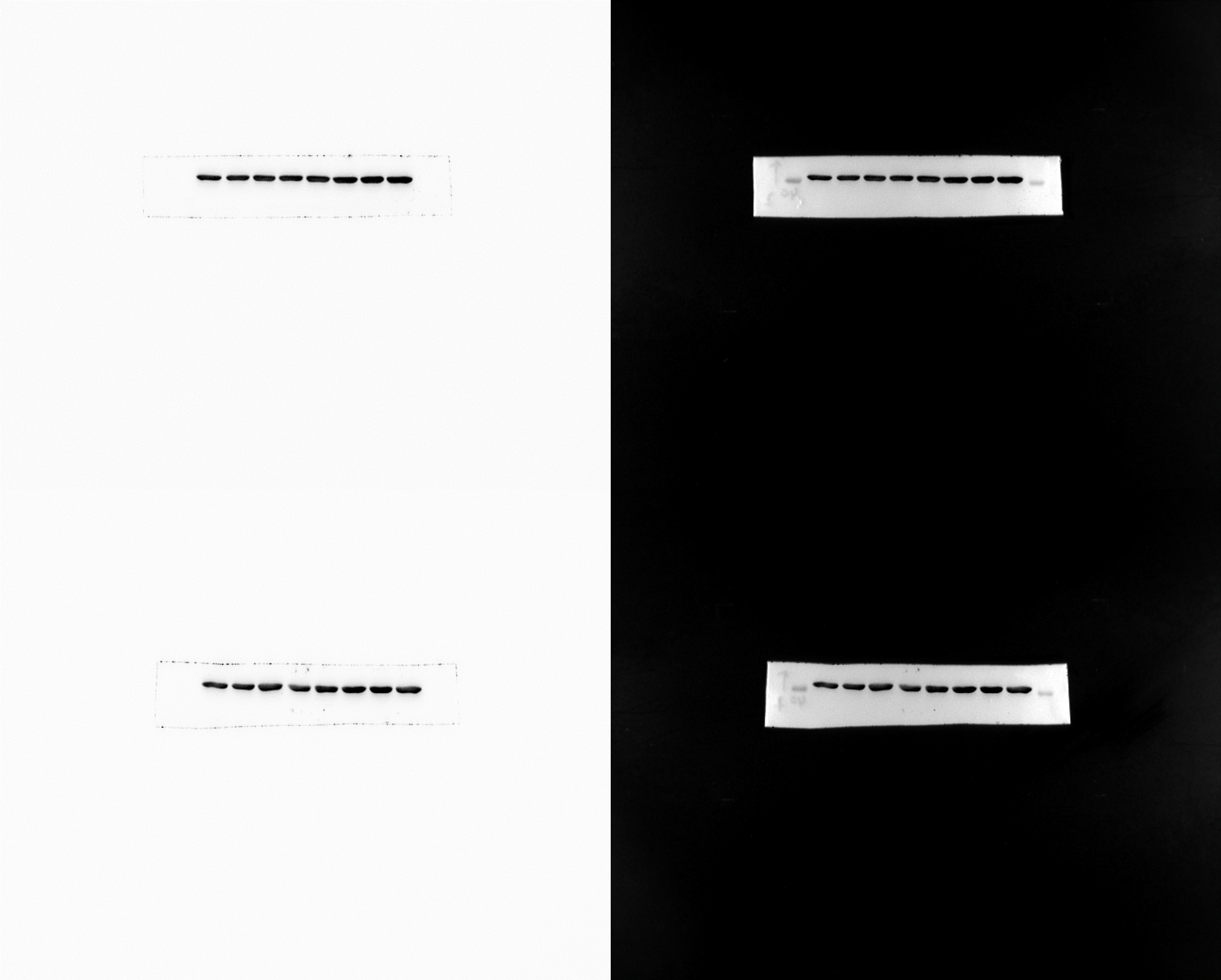

Supplement: Figure 1—source data 1. [file elife-96161-fig1-data1.zip › Figure 1-Source data1/Figure 1M-Source data1-a┬-actin.png]

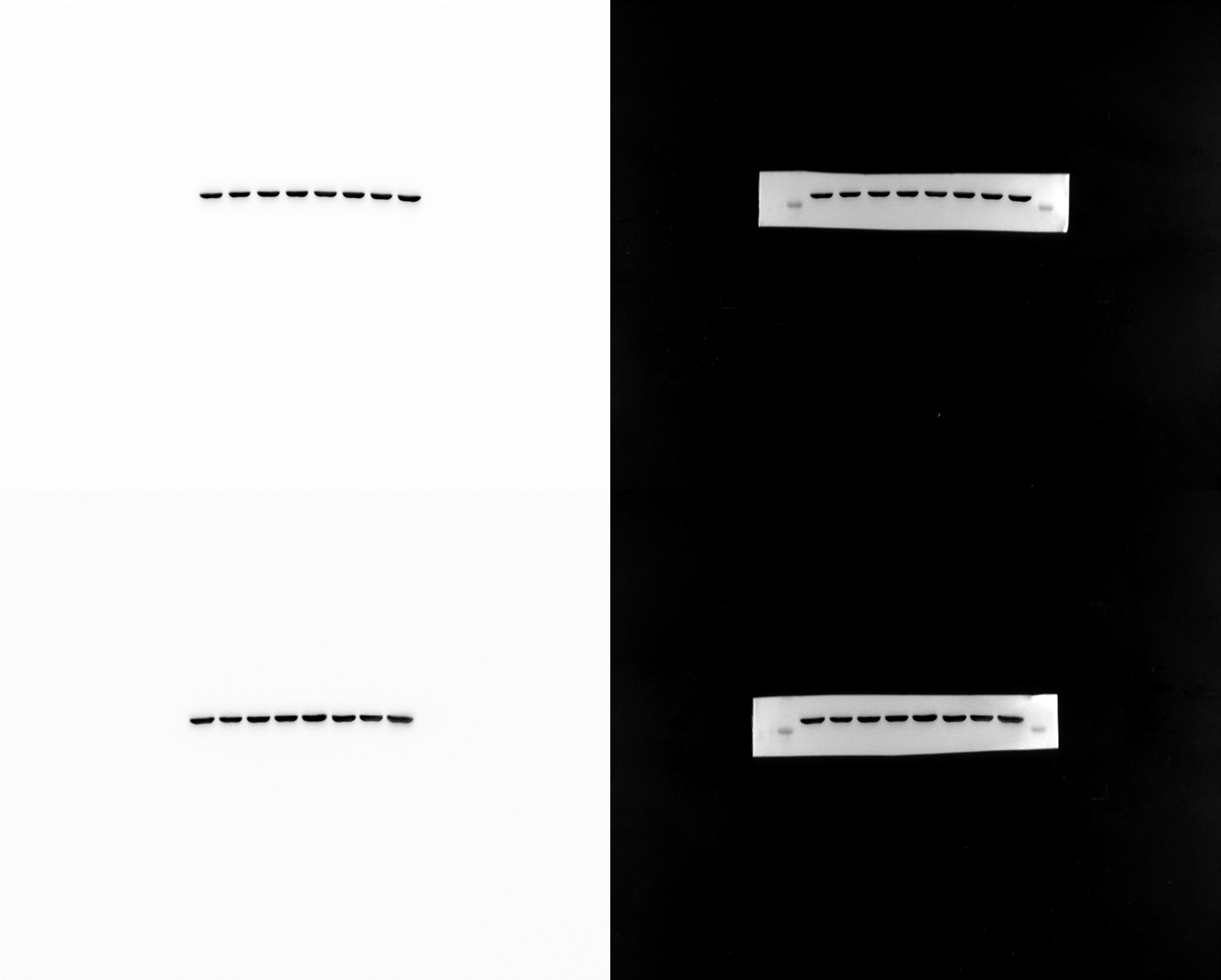

Supplement: Figure 1—source data 1. [file elife-96161-fig1-data1.zip › Figure 1-Source data1/Figure 1M-Source data2-a┬-actin.png]

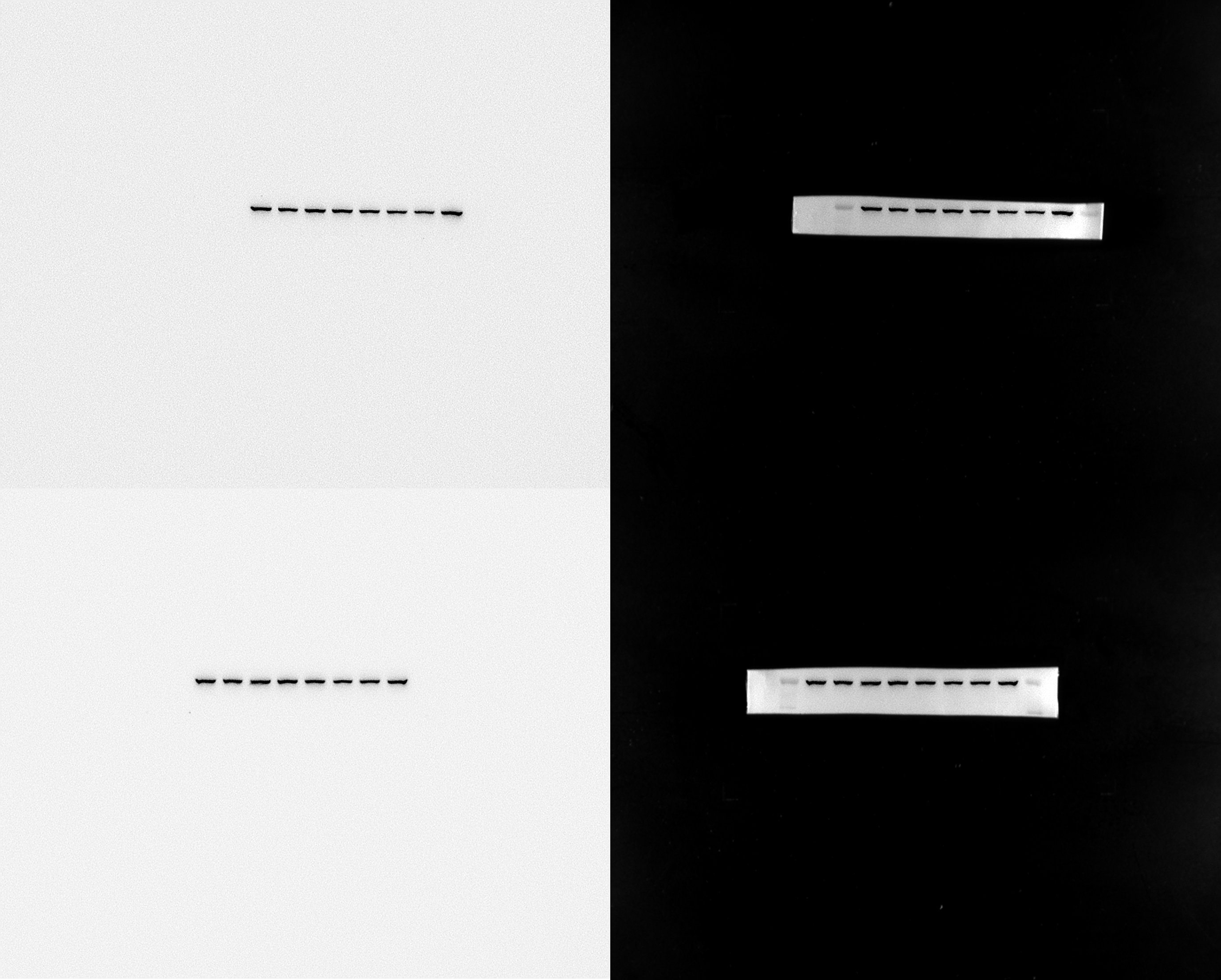

Supplement: Figure 1—source data 1. [file elife-96161-fig1-data1.zip › Figure 1-Source data1/Figure 1M-Source data2-a┬-Catenin.png]

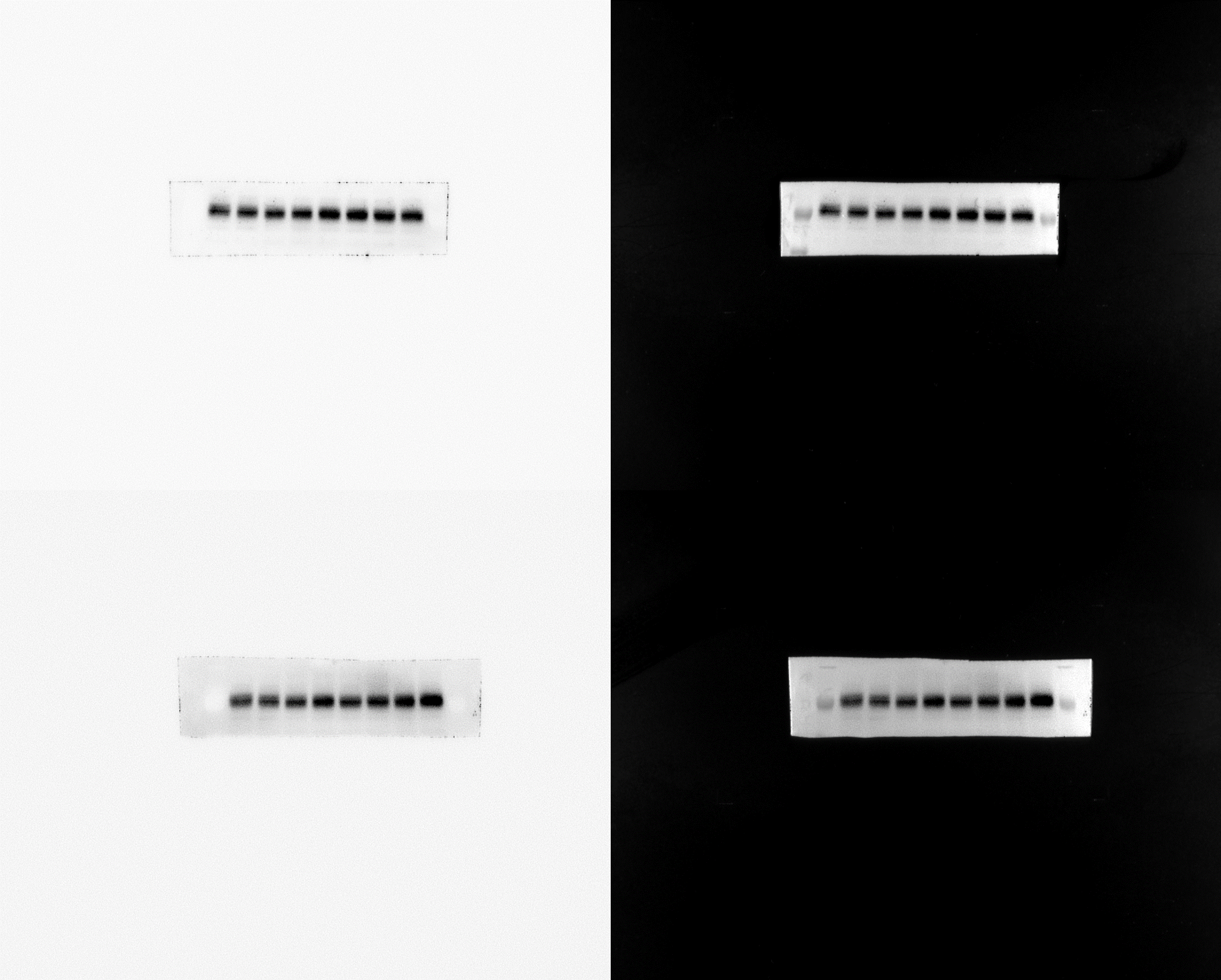

Supplement: Figure 1—source data 1. [file elife-96161-fig1-data1.zip › Figure 1-Source data1/Figure 1M-Source data3-BCRP.png]

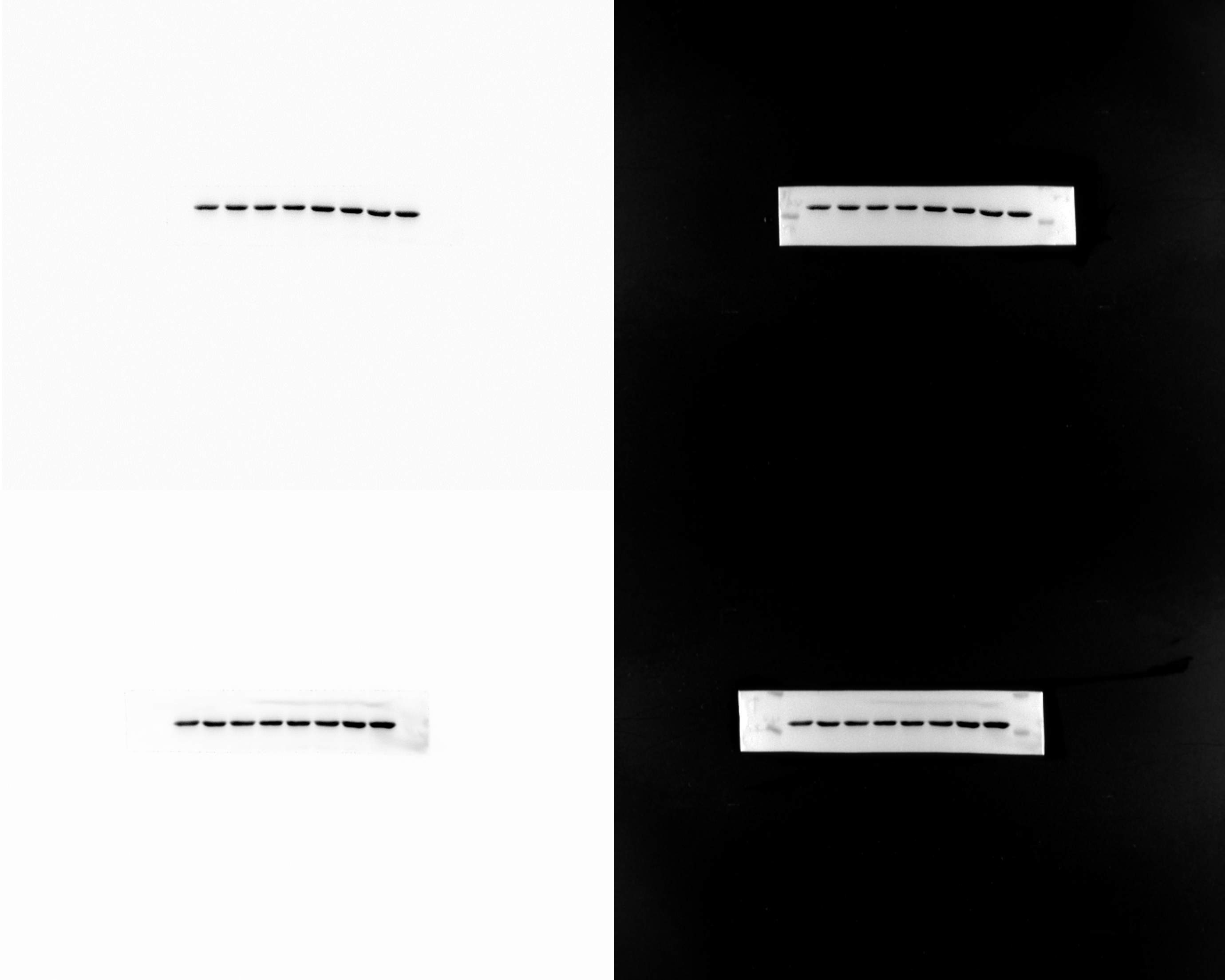

Supplement: Figure 1—source data 1. [file elife-96161-fig1-data1.zip › Figure 1-Source data1/Figure 1M-Source data3-a┬-actin.png]

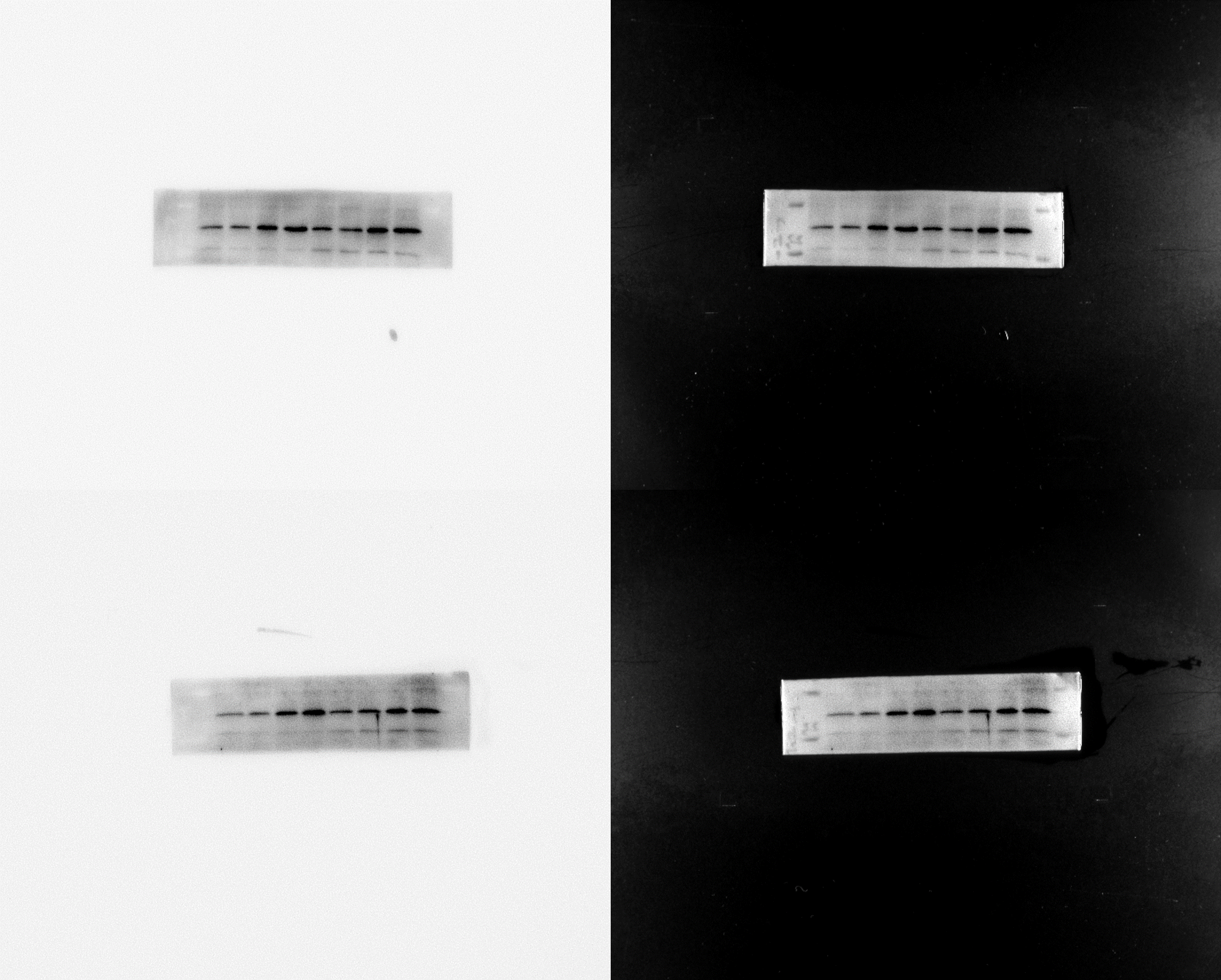

Supplement: Figure 2—source data 1. [file elife-96161-fig2-data1.zip › Figure 2-Source data1/Figure2A-Source data1-Claudin-5.png]

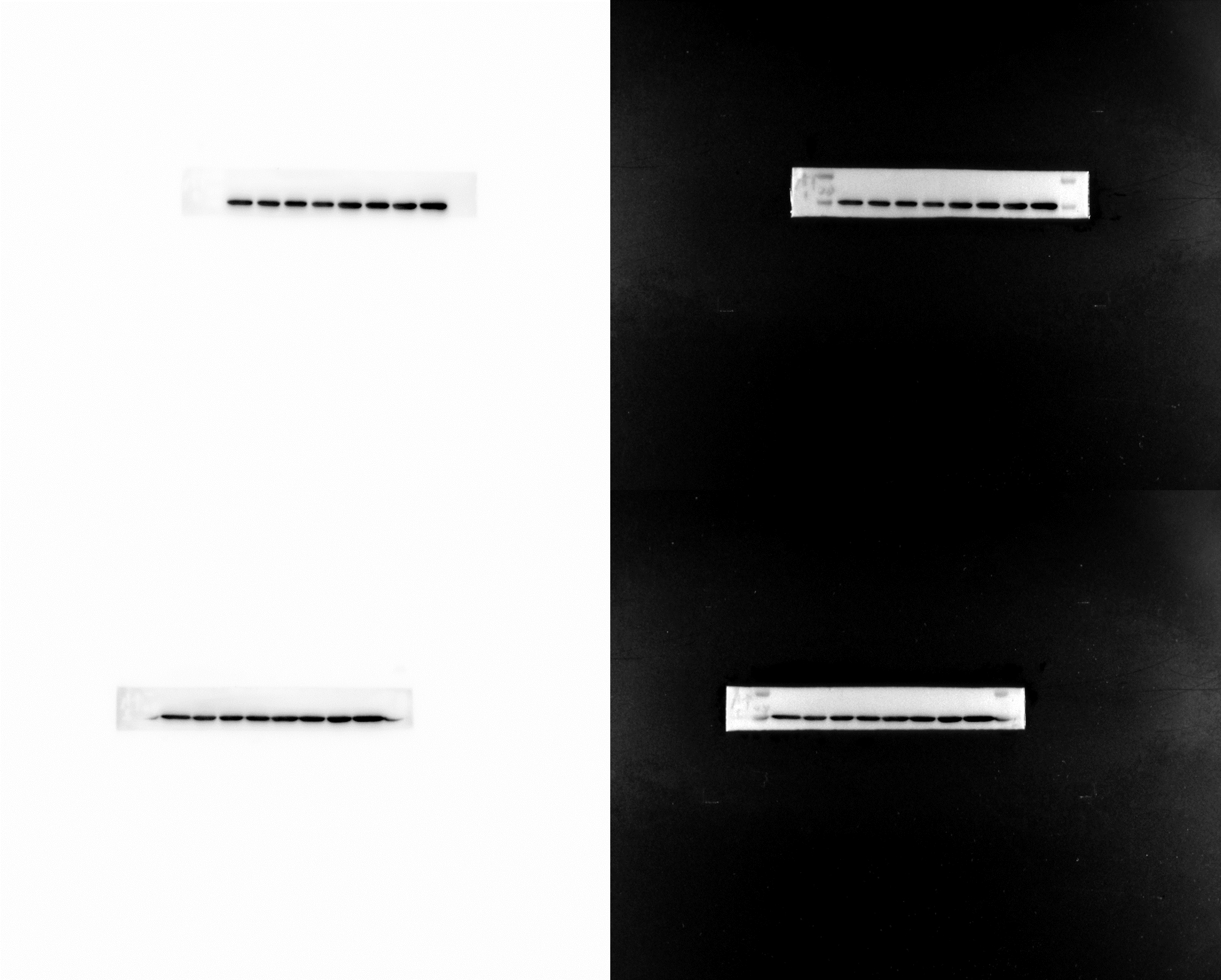

Supplement: Figure 2—source data 1. [file elife-96161-fig2-data1.zip › Figure 2-Source data1/Figure2A-Source data1-a┬-actin.png]

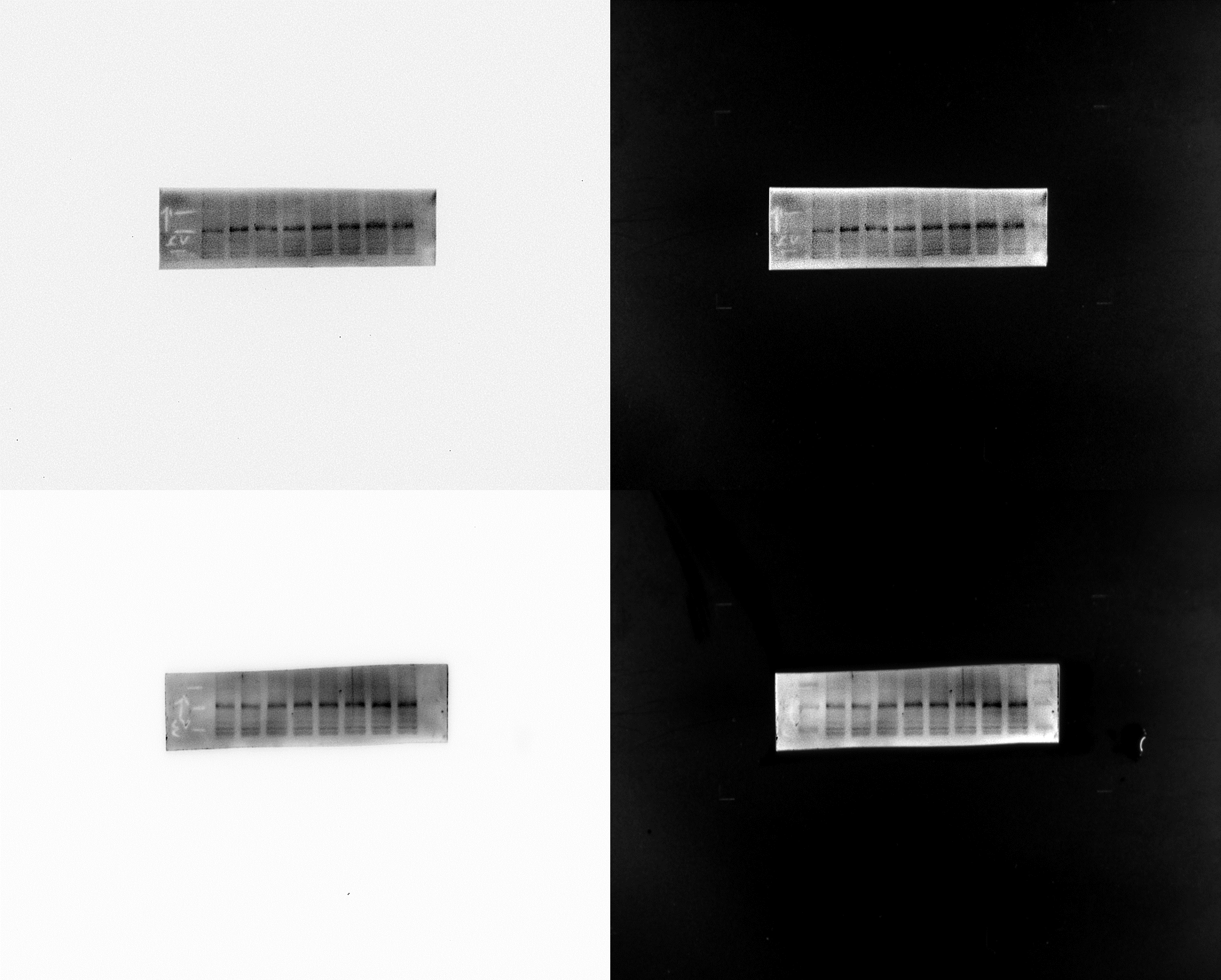

Supplement: Figure 2—source data 1. [file elife-96161-fig2-data1.zip › Figure 2-Source data1/Figure2A-Source data2-VE-Cadherin.png]

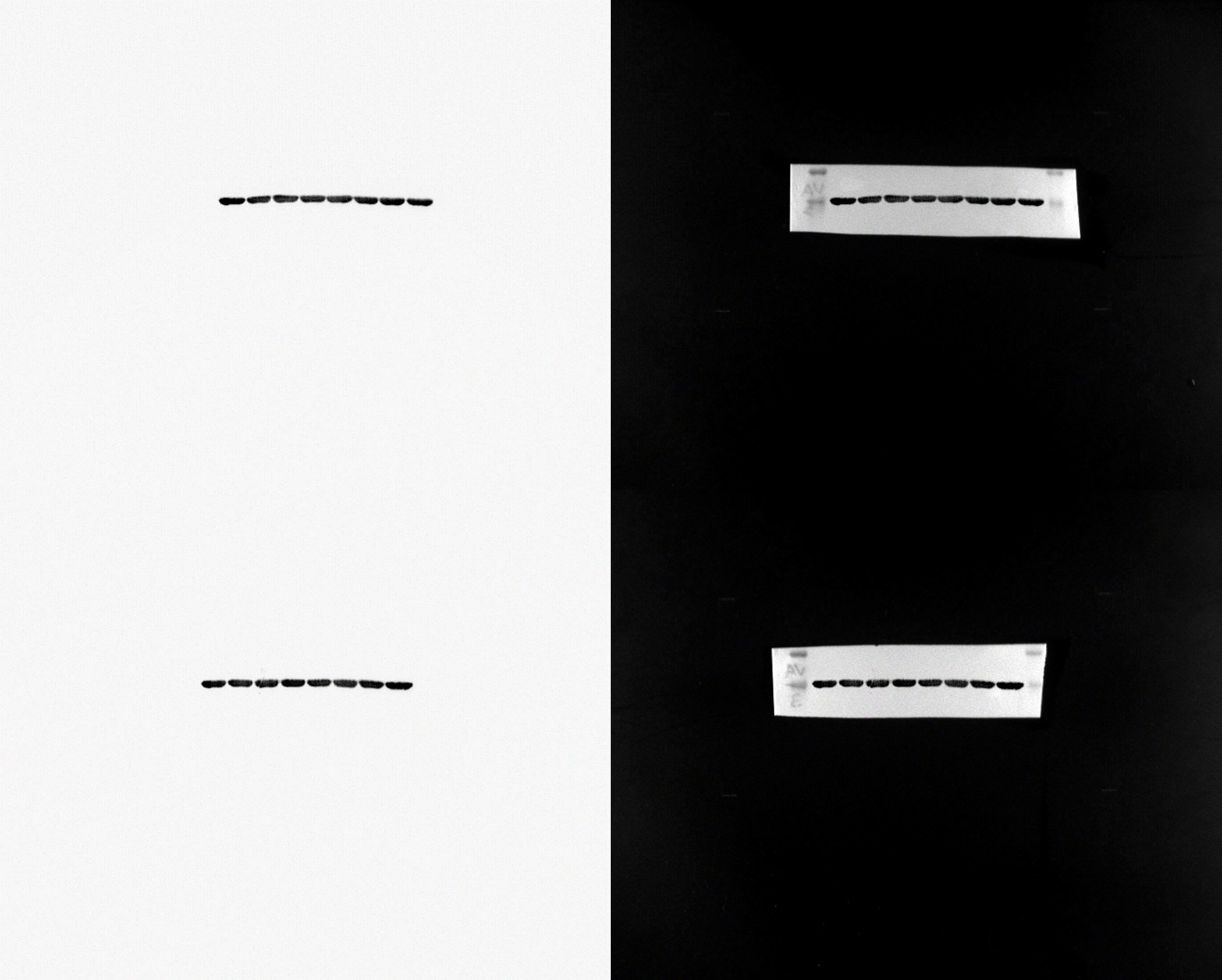

Supplement: Figure 2—source data 1. [file elife-96161-fig2-data1.zip › Figure 2-Source data1/Figure2A-Source data2-a┬-actin.png]

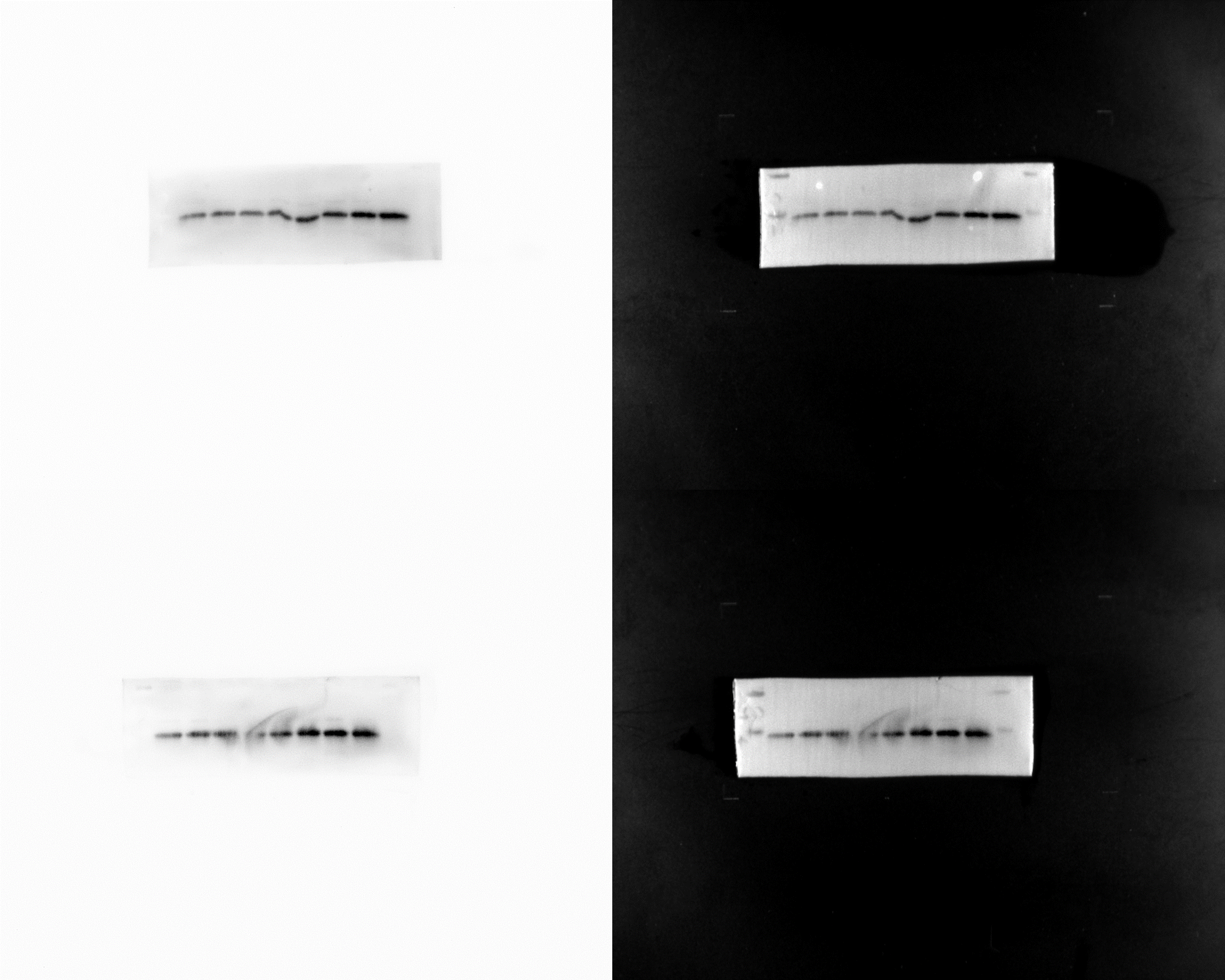

Supplement: Figure 2—source data 1. [file elife-96161-fig2-data1.zip › Figure 2-Source data1/Figure2D-Source data1-Claudin-5.png]

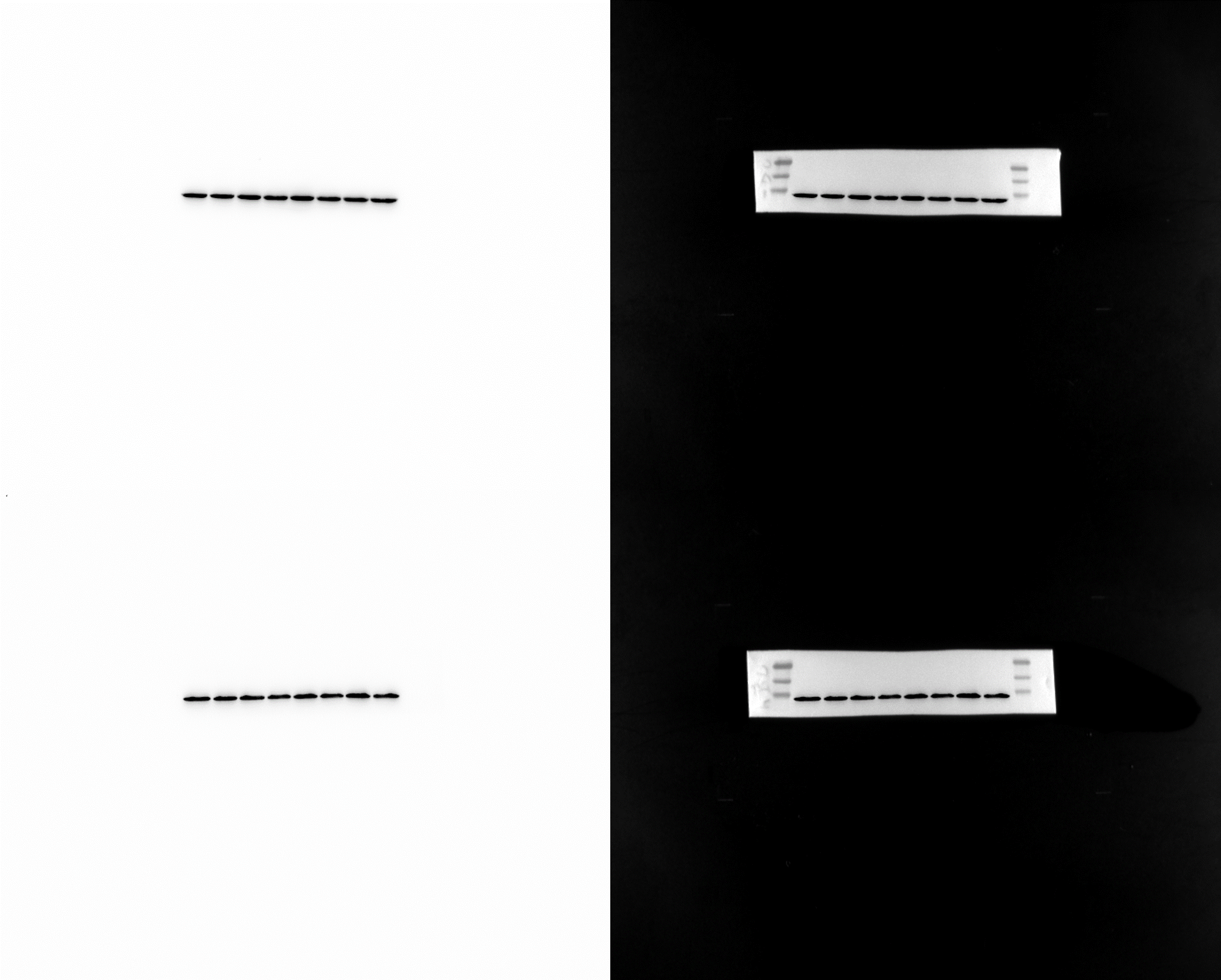

Supplement: Figure 2—source data 1. [file elife-96161-fig2-data1.zip › Figure 2-Source data1/Figure2D-Source data1-a┬-actin.png]

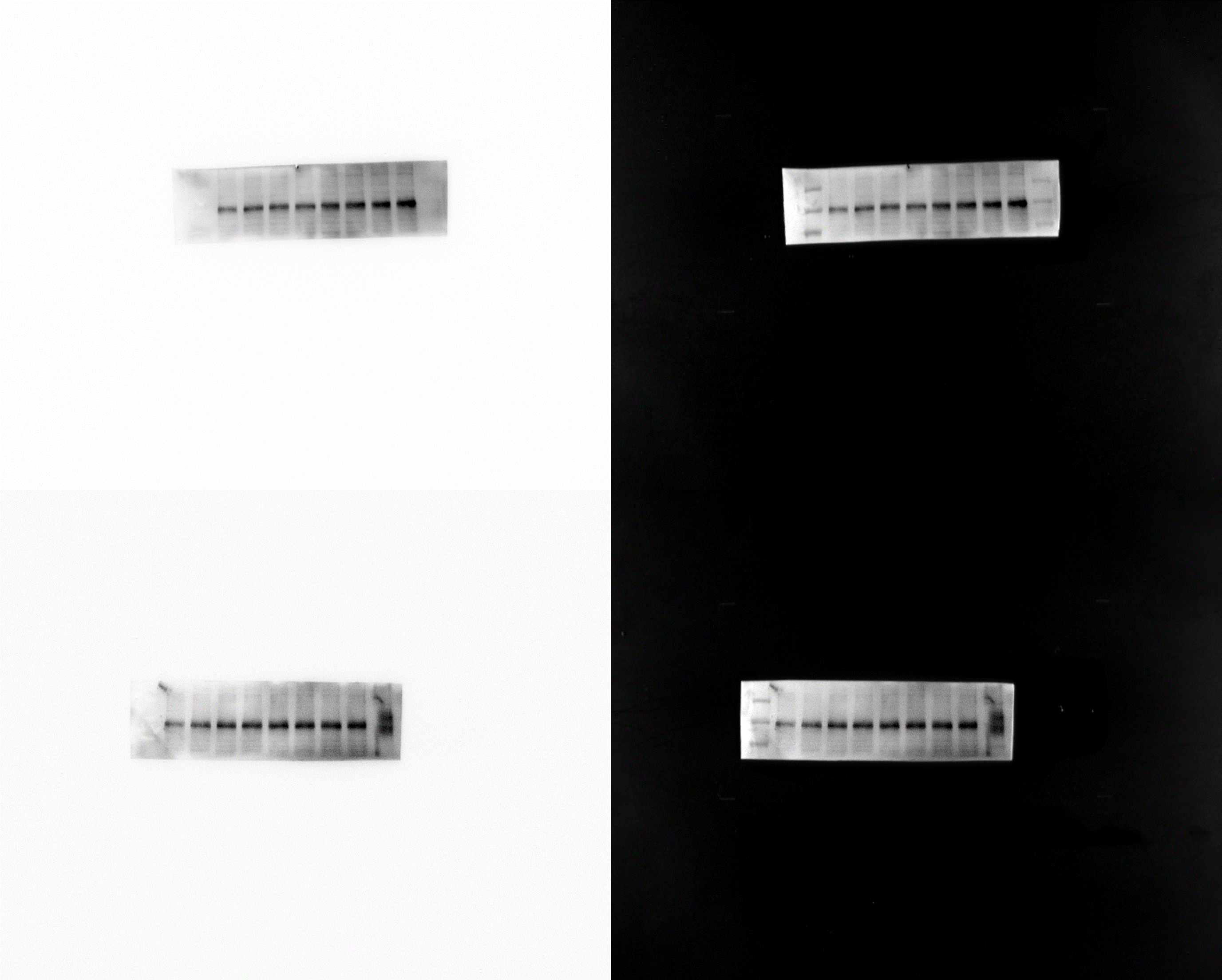

Supplement: Figure 2—source data 1. [file elife-96161-fig2-data1.zip › Figure 2-Source data1/Figure2D-Source data2-VE-Cadherin.png]

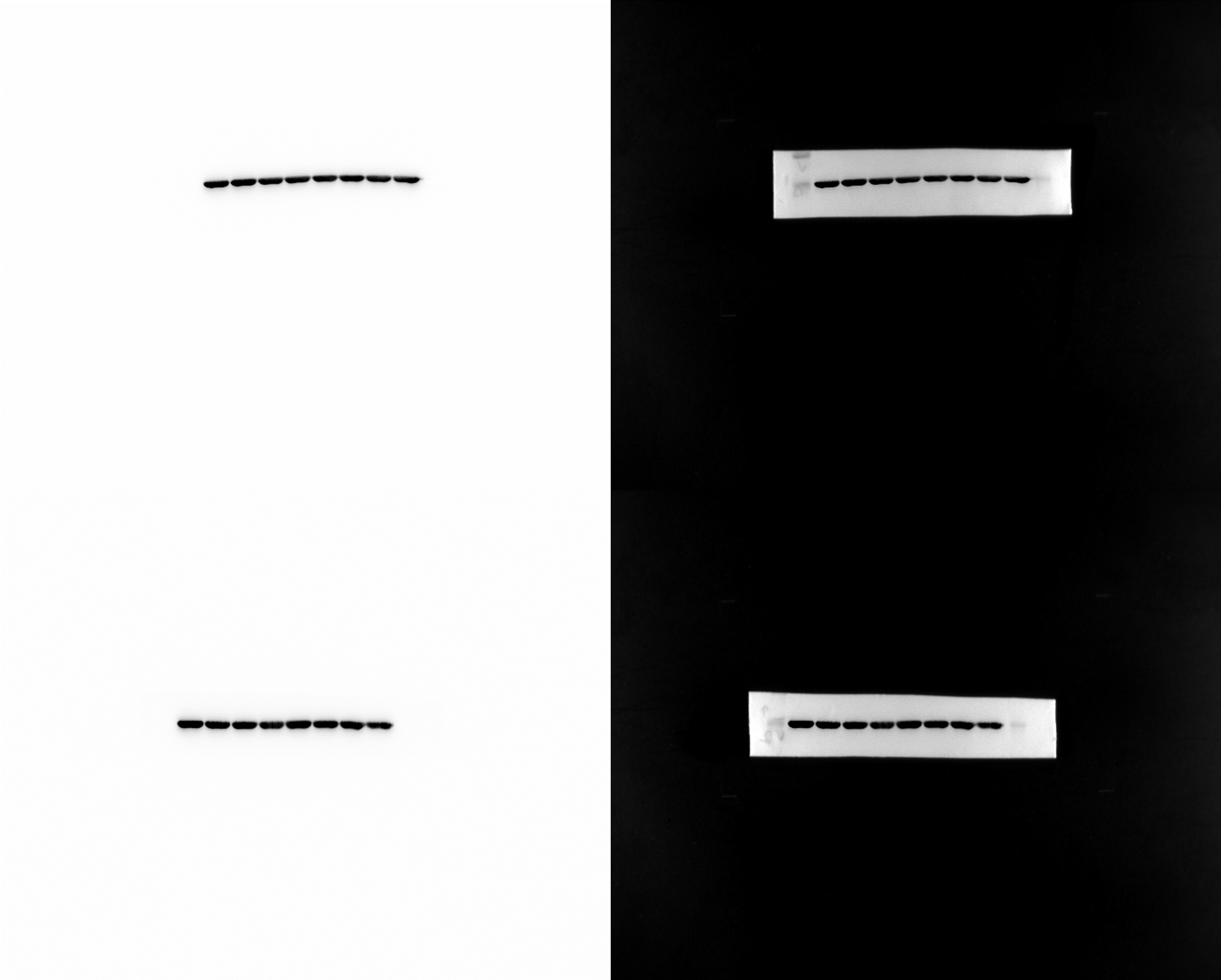

Supplement: Figure 2—source data 1. [file elife-96161-fig2-data1.zip › Figure 2-Source data1/Figure2D-Source data2-a┬-actin.png]

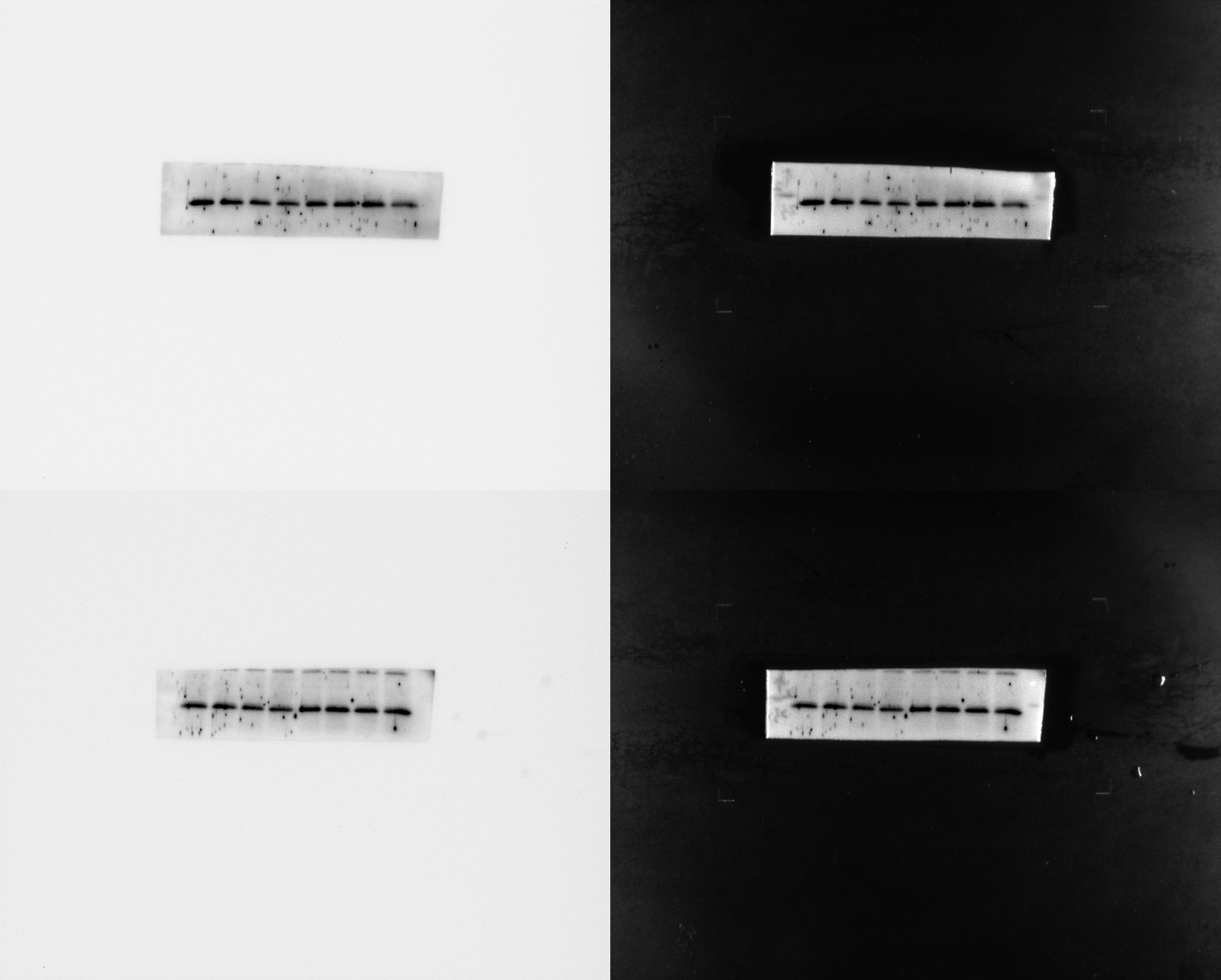

Supplement: Figure 2—source data 1. [file elife-96161-fig2-data1.zip › Figure 2-Source data1/Figure2E-Source data1-Claudin-5.png]

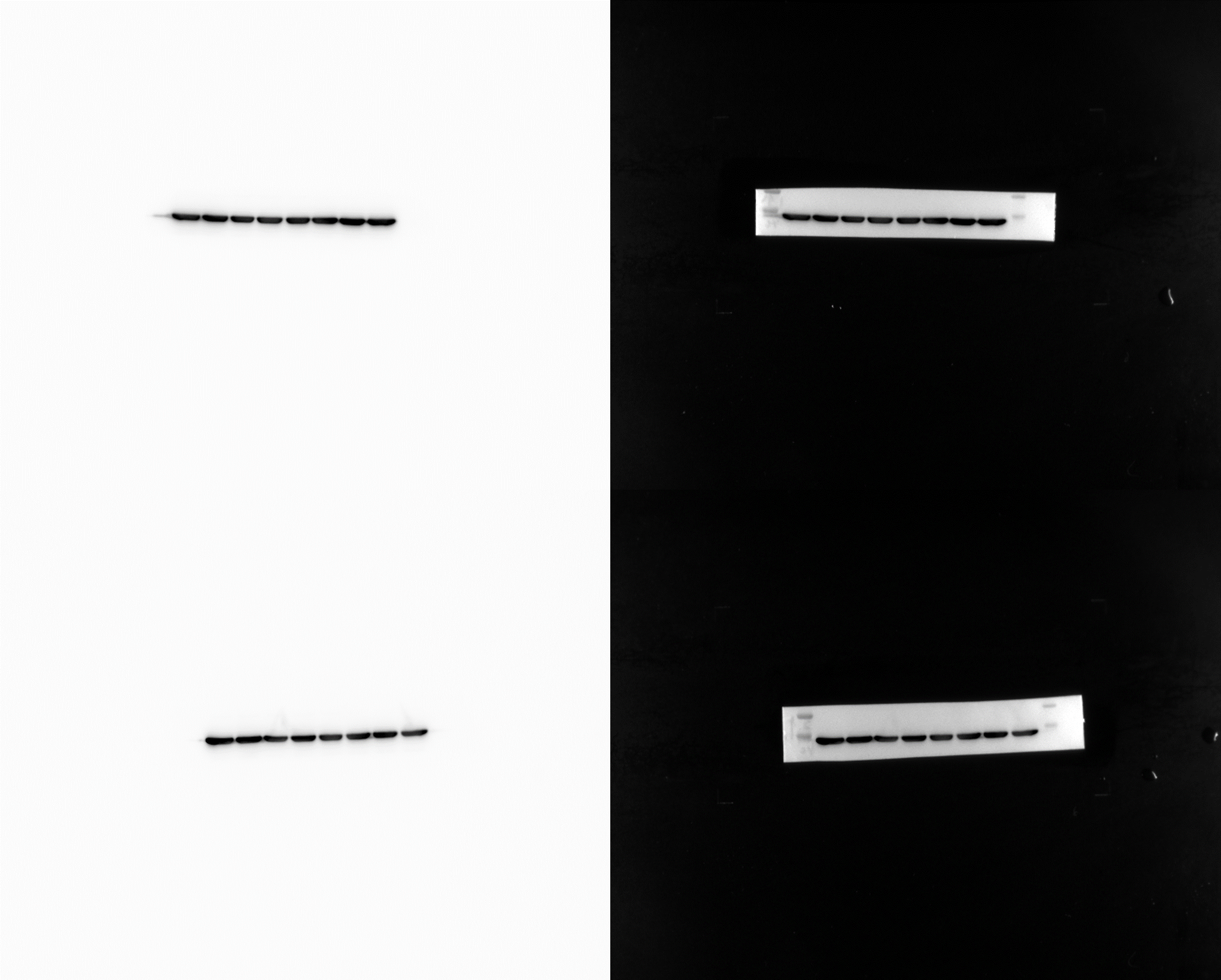

Supplement: Figure 2—source data 1. [file elife-96161-fig2-data1.zip › Figure 2-Source data1/Figure2E-Source data1-a┬-actin.png]

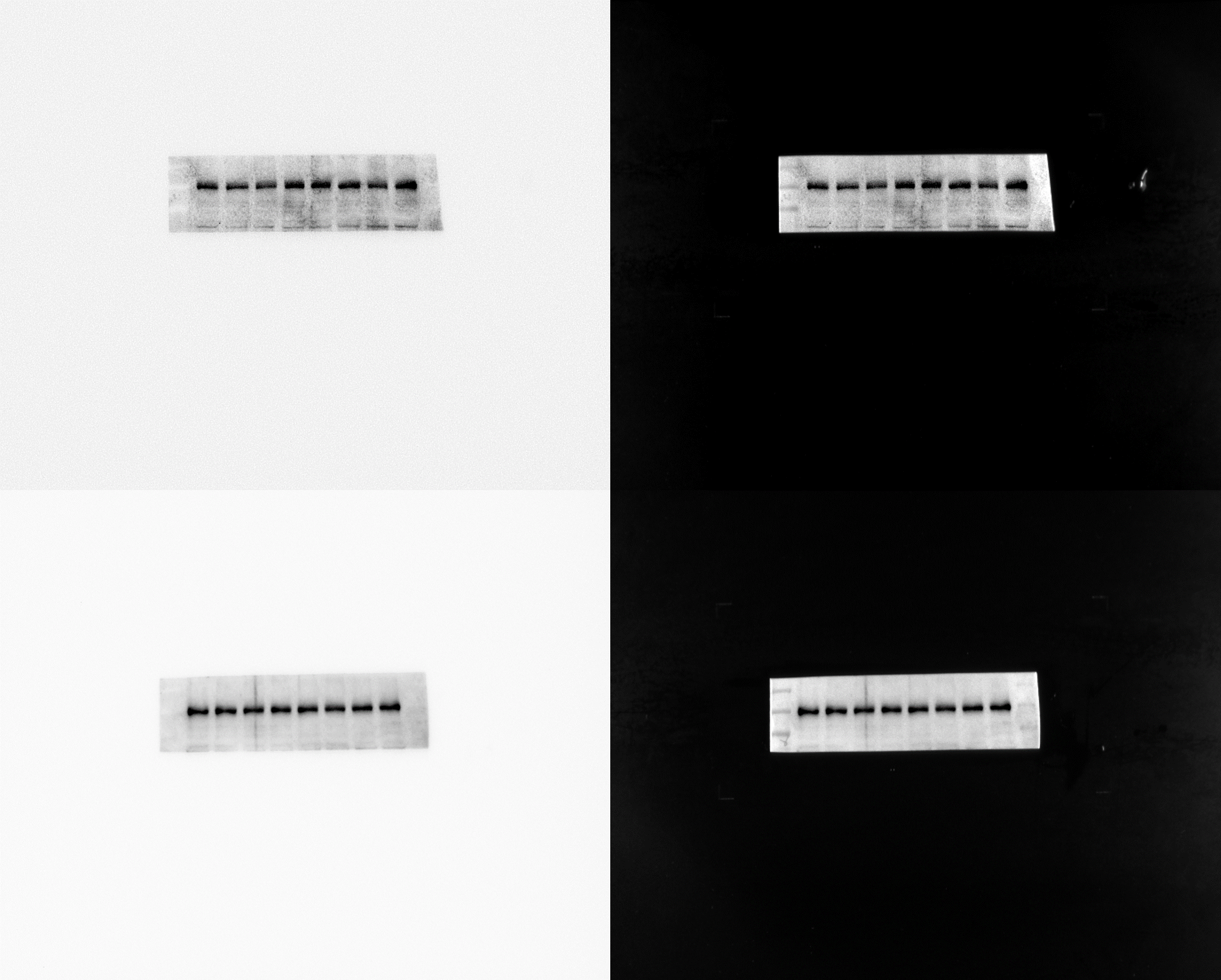

Supplement: Figure 2—source data 1. [file elife-96161-fig2-data1.zip › Figure 2-Source data1/Figure2E-Source data2-VE-Cadherin.png]

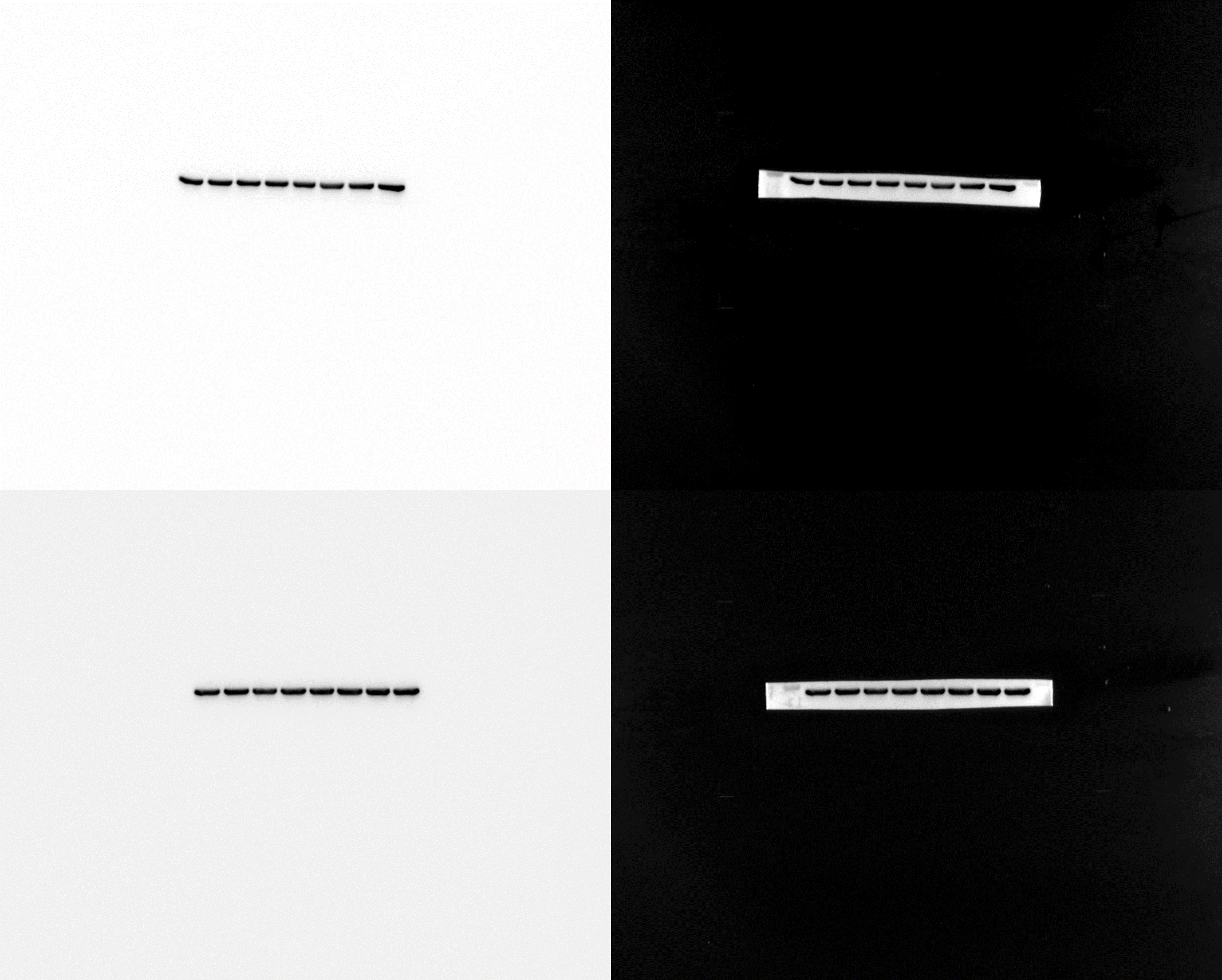

Supplement: Figure 2—source data 1. [file elife-96161-fig2-data1.zip › Figure 2-Source data1/Figure2E-Source data2-a┬-actin.png]

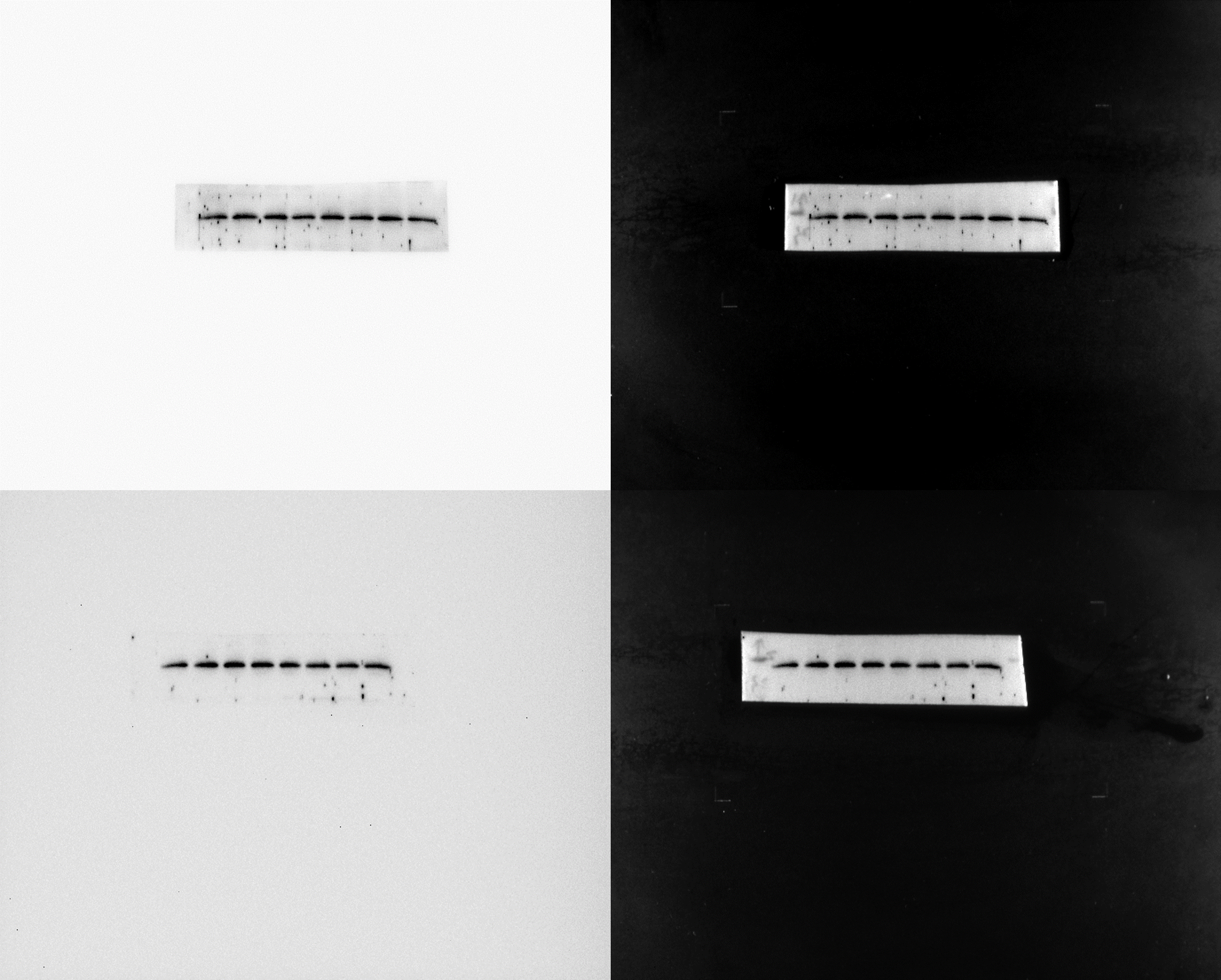

Supplement: Figure 2—source data 1. [file elife-96161-fig2-data1.zip › Figure 2-Source data1/Figure2F-Source data1-Claudin-5.png]

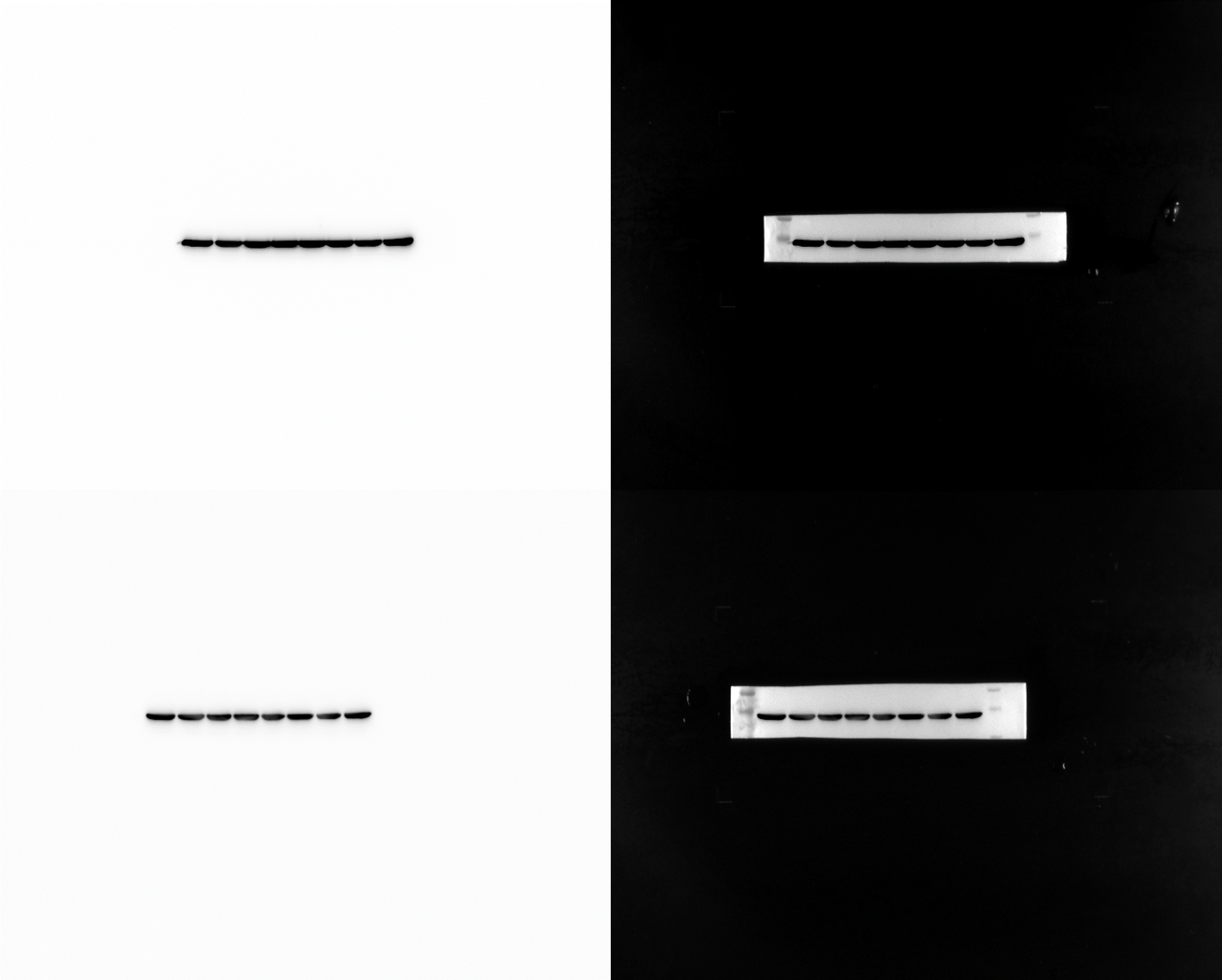

Supplement: Figure 2—source data 1. [file elife-96161-fig2-data1.zip › Figure 2-Source data1/Figure2F-Source data1-a┬-actin.png]

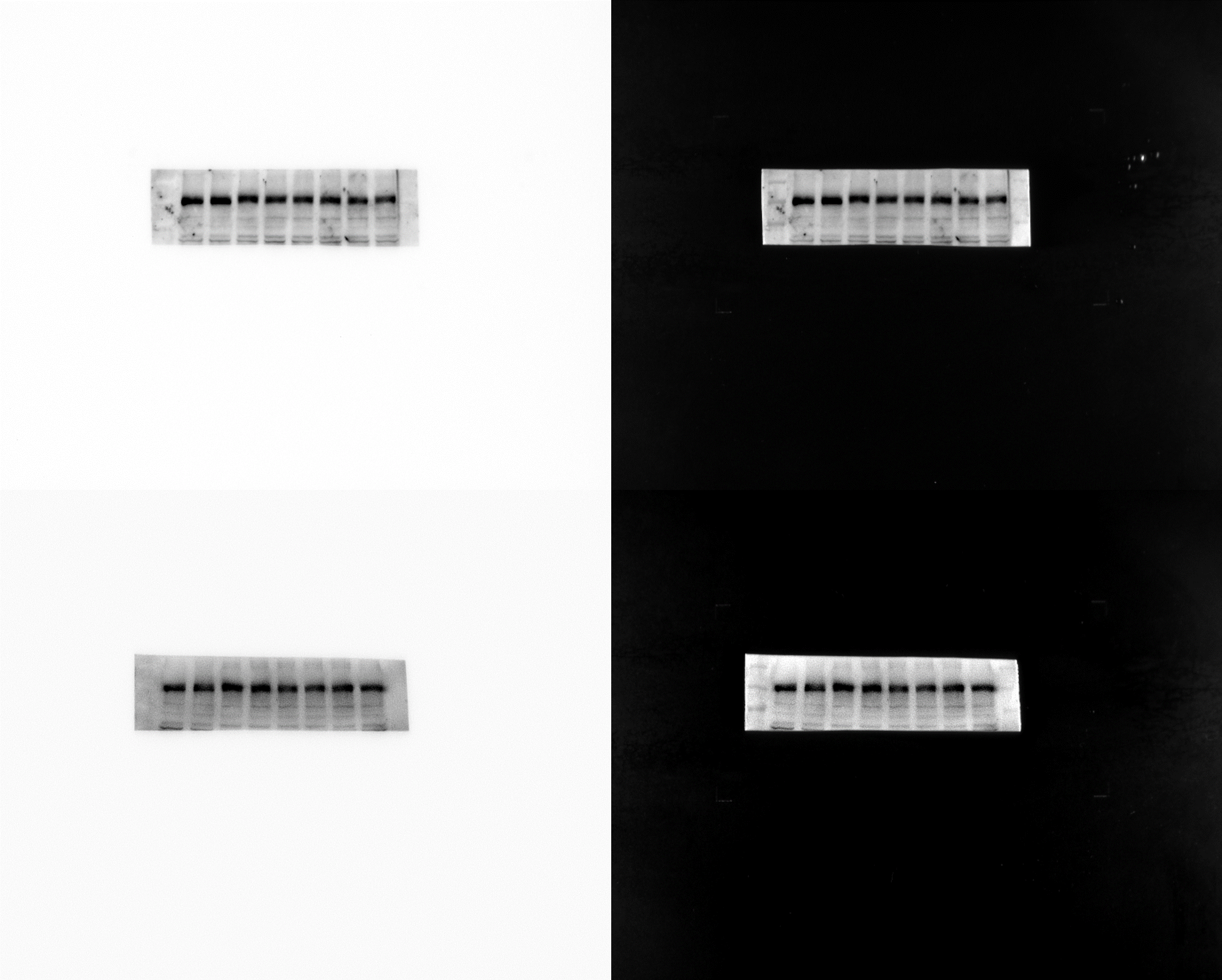

Supplement: Figure 2—source data 1. [file elife-96161-fig2-data1.zip › Figure 2-Source data1/Figure2F-Source data2-VE-Cadherin.png]

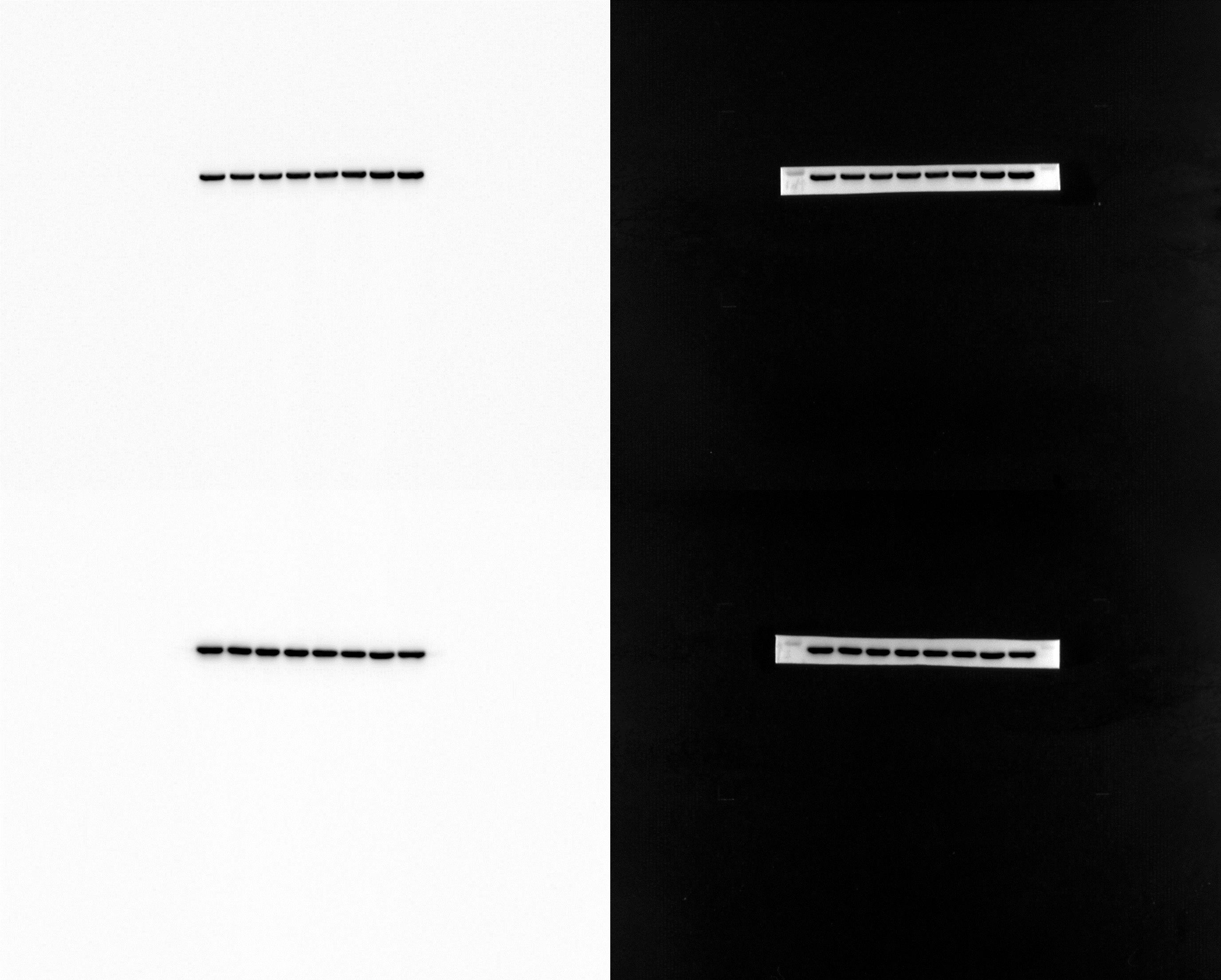

Supplement: Figure 2—source data 1. [file elife-96161-fig2-data1.zip › Figure 2-Source data1/Figure2F-Source data2-a┬-actin.png]

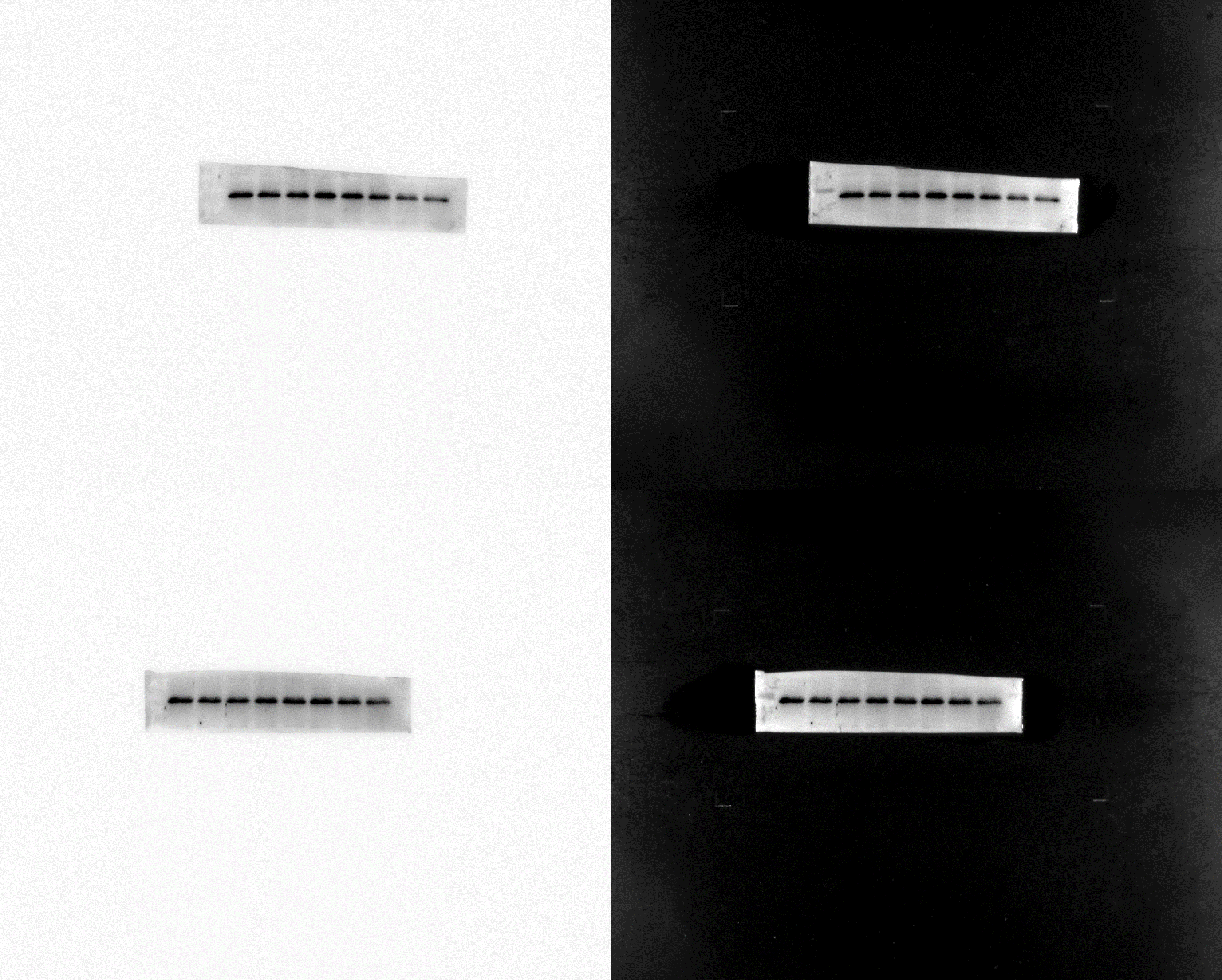

Supplement: Figure 2—source data 1. [file elife-96161-fig2-data1.zip › Figure 2-Source data1/Figure2G-Source data1-Claudin-5.png]

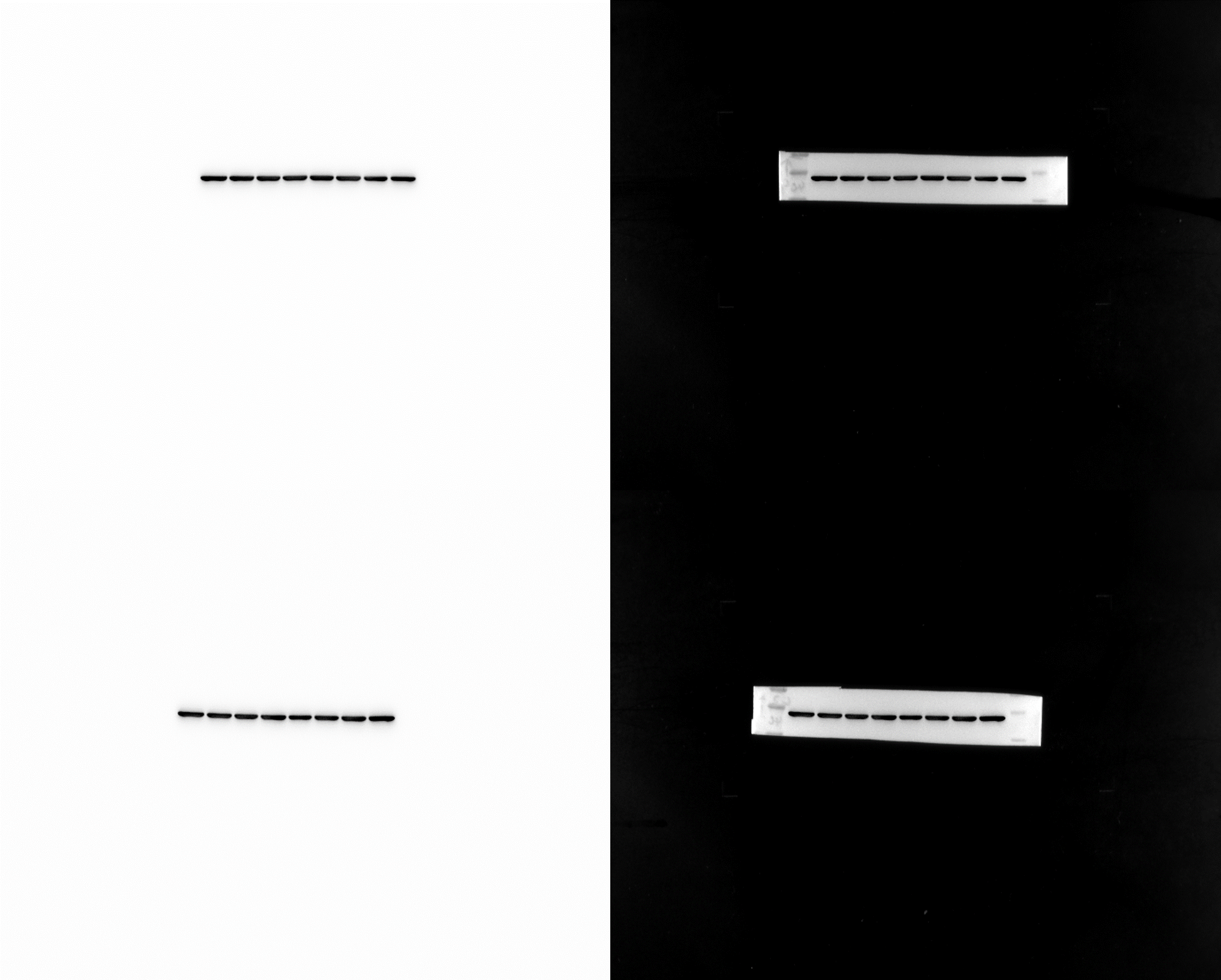

Supplement: Figure 2—source data 1. [file elife-96161-fig2-data1.zip › Figure 2-Source data1/Figure2G-Source data1-a┬-actin.png]

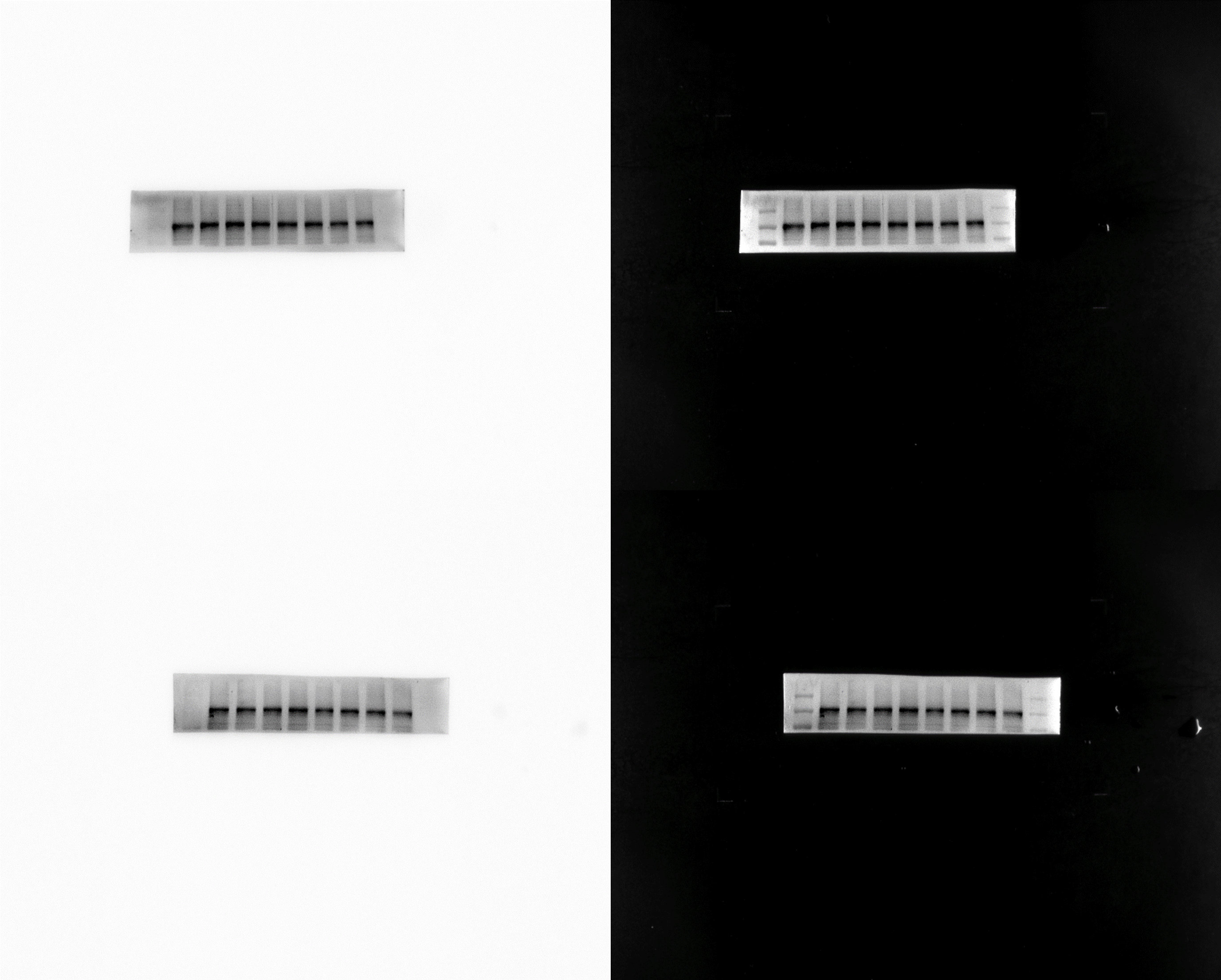

Supplement: Figure 2—source data 1. [file elife-96161-fig2-data1.zip › Figure 2-Source data1/Figure2G-Source data2-VE-Cadherin.png]

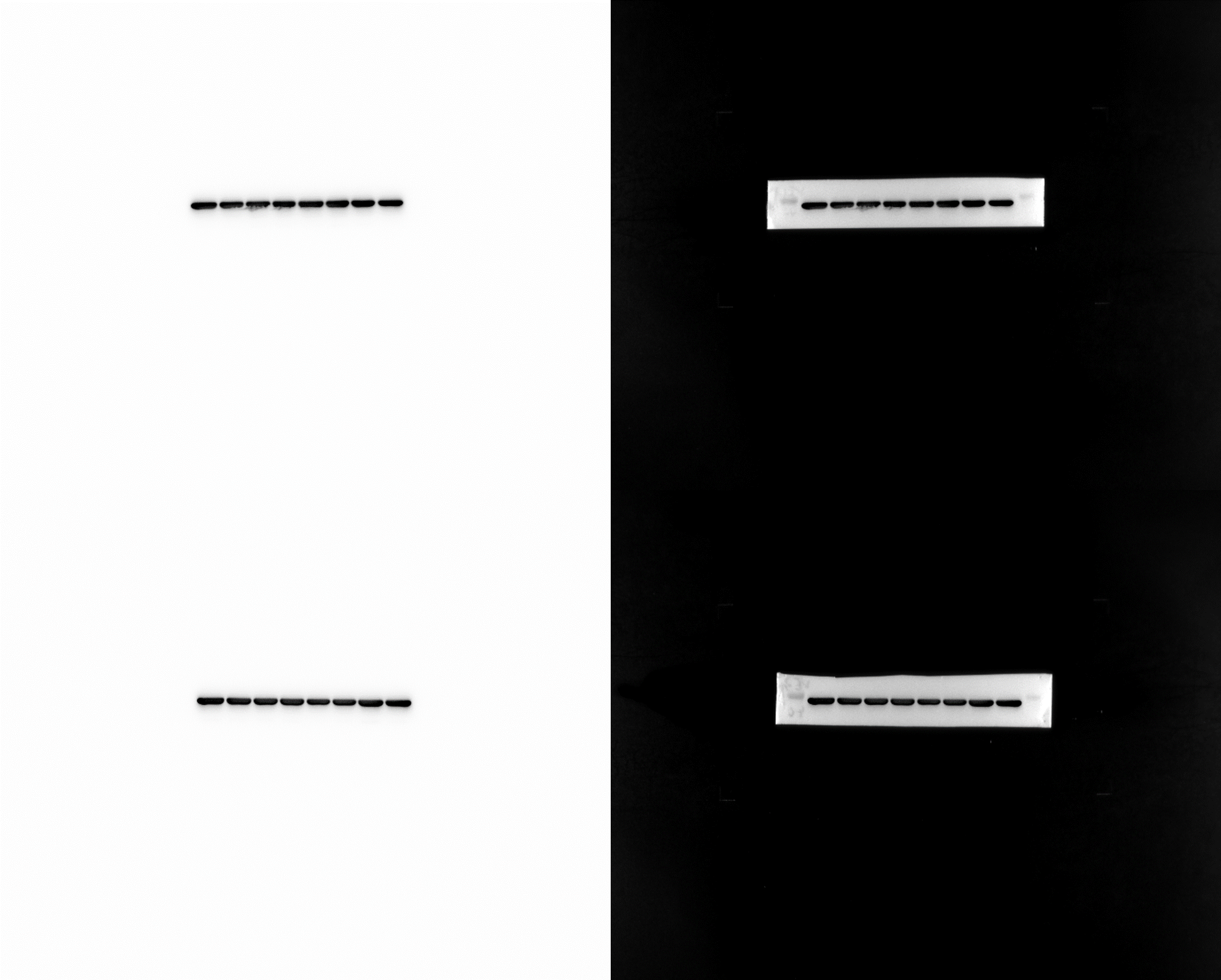

Supplement: Figure 2—source data 1. [file elife-96161-fig2-data1.zip › Figure 2-Source data1/Figure2G-Source data2-a┬-actin.png]

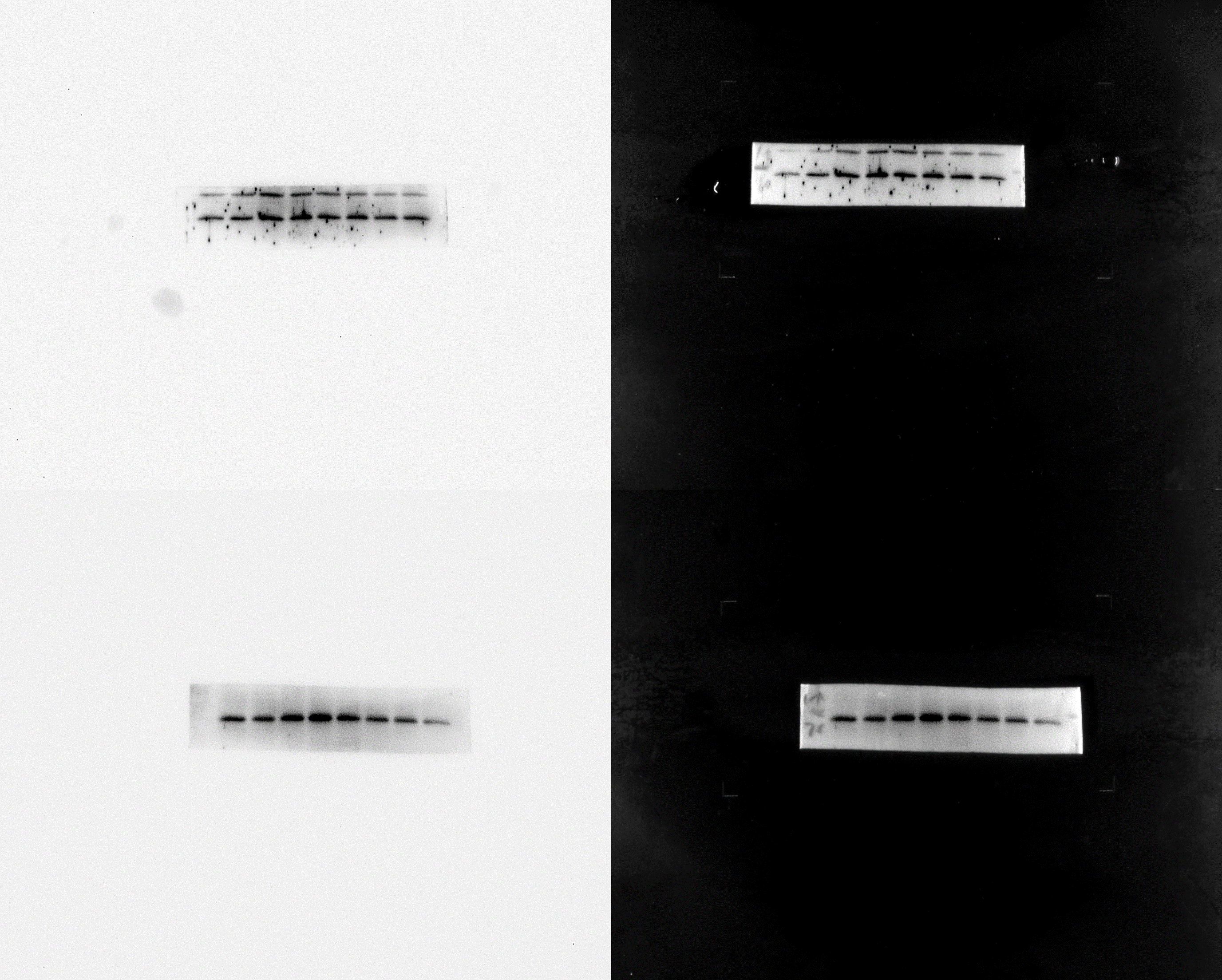

Supplement: Figure 2—source data 1. [file elife-96161-fig2-data1.zip › Figure 2-Source data1/Figure2H-Source data1-Claudin-5.png]

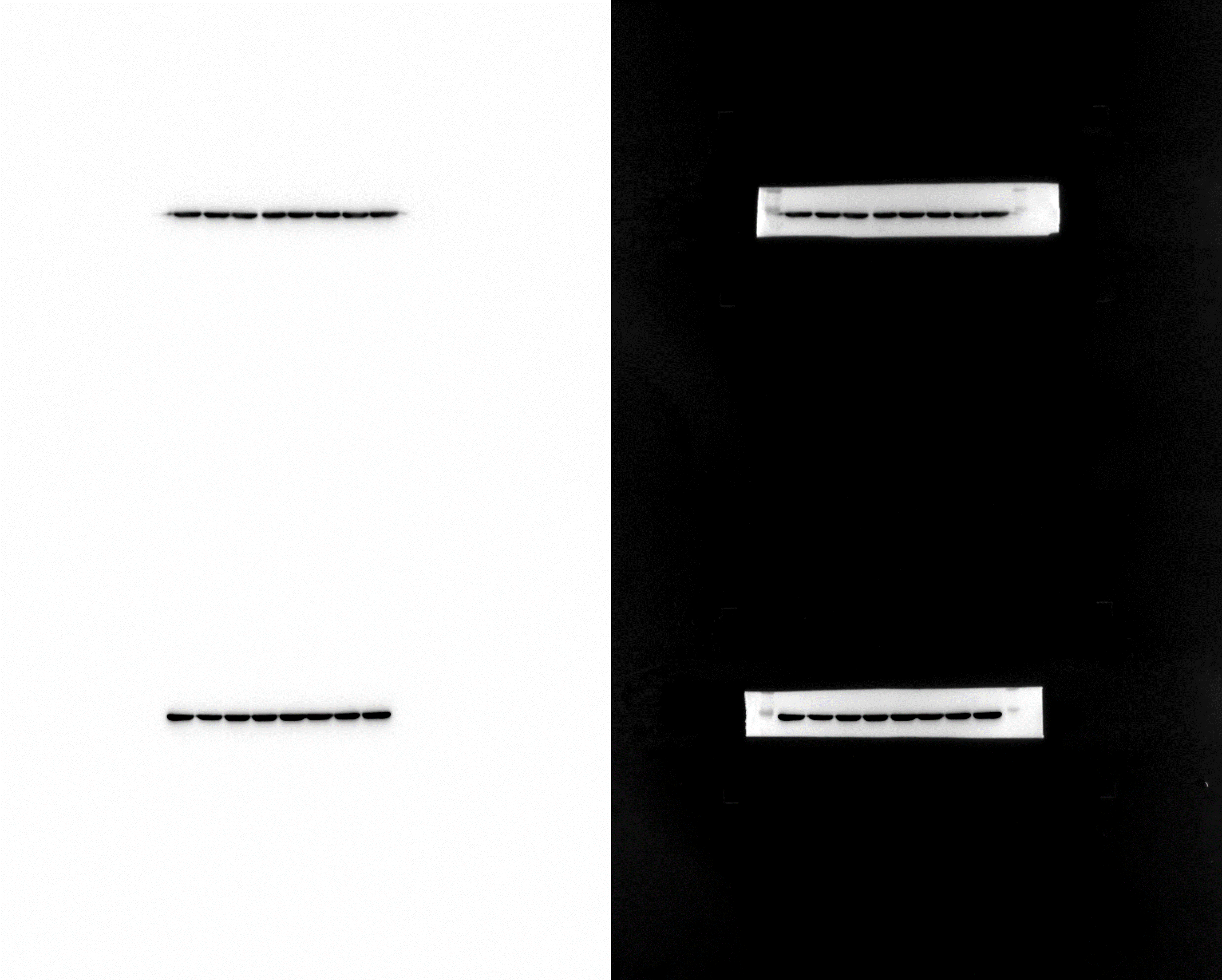

Supplement: Figure 2—source data 1. [file elife-96161-fig2-data1.zip › Figure 2-Source data1/Figure2H-Source data1-a┬-actin.png]

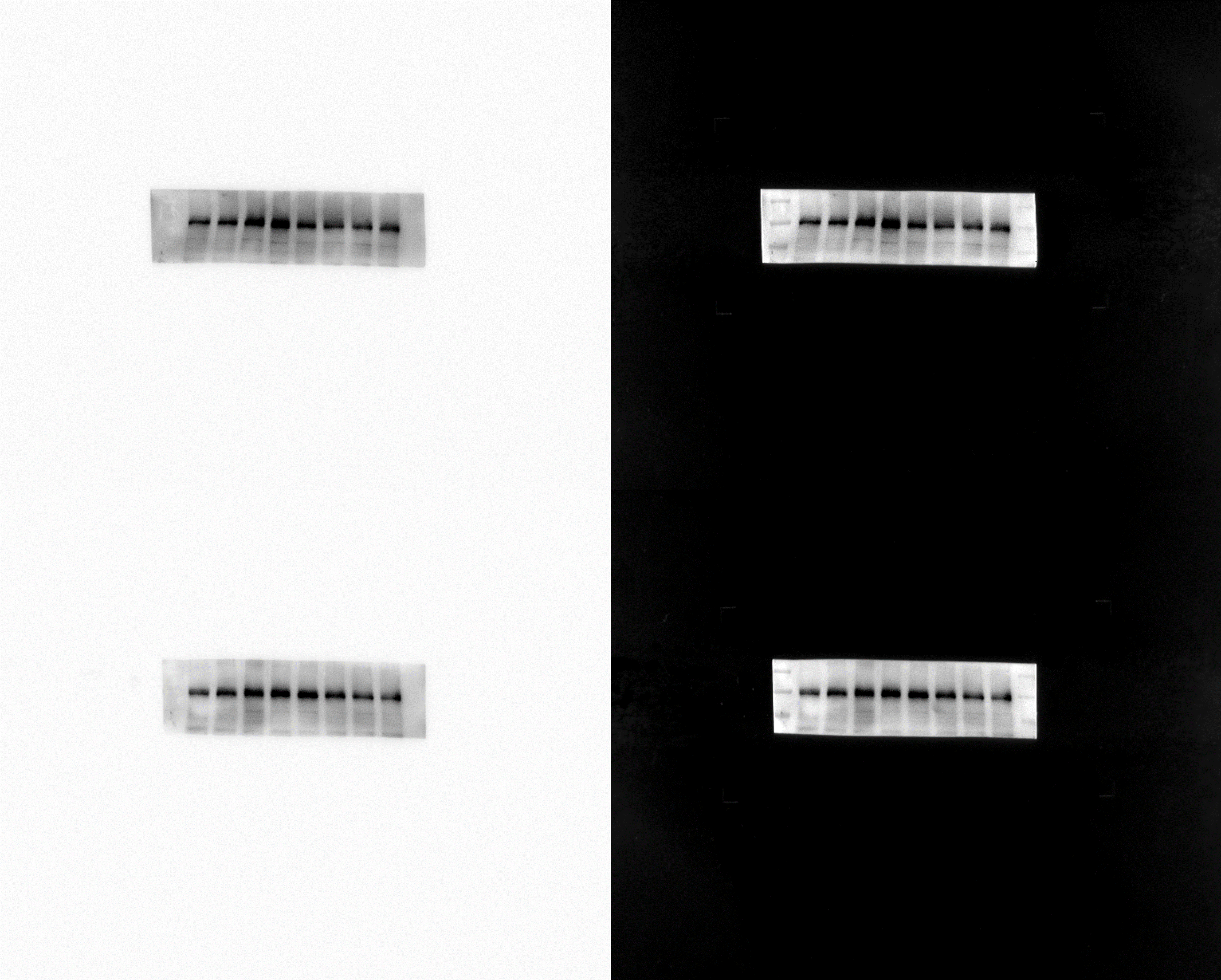

Supplement: Figure 2—source data 1. [file elife-96161-fig2-data1.zip › Figure 2-Source data1/Figure2H-Source data2-VE-Cadherin.png]

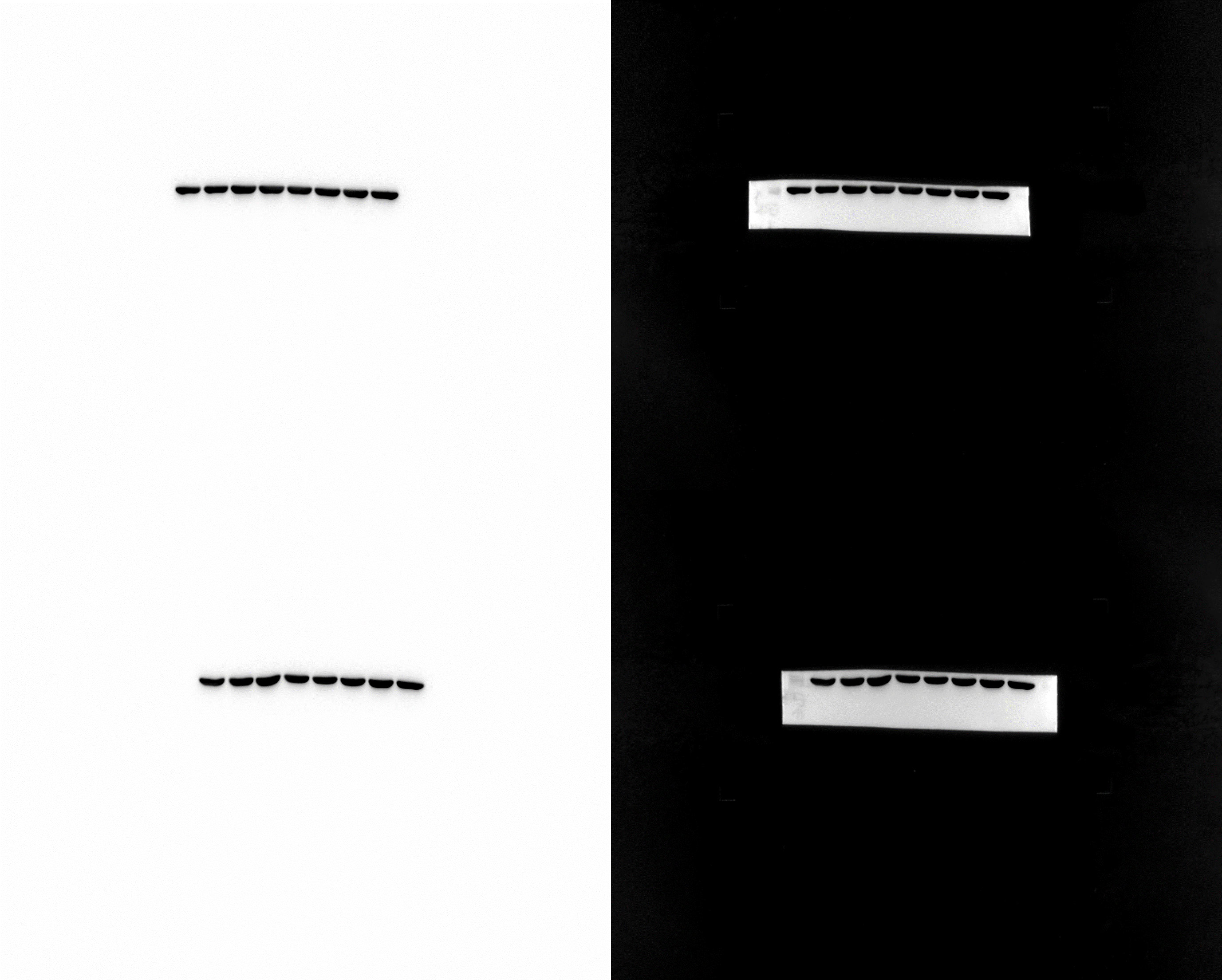

Supplement: Figure 2—source data 1. [file elife-96161-fig2-data1.zip › Figure 2-Source data1/Figure2H-Source data2-a┬-actin.png]

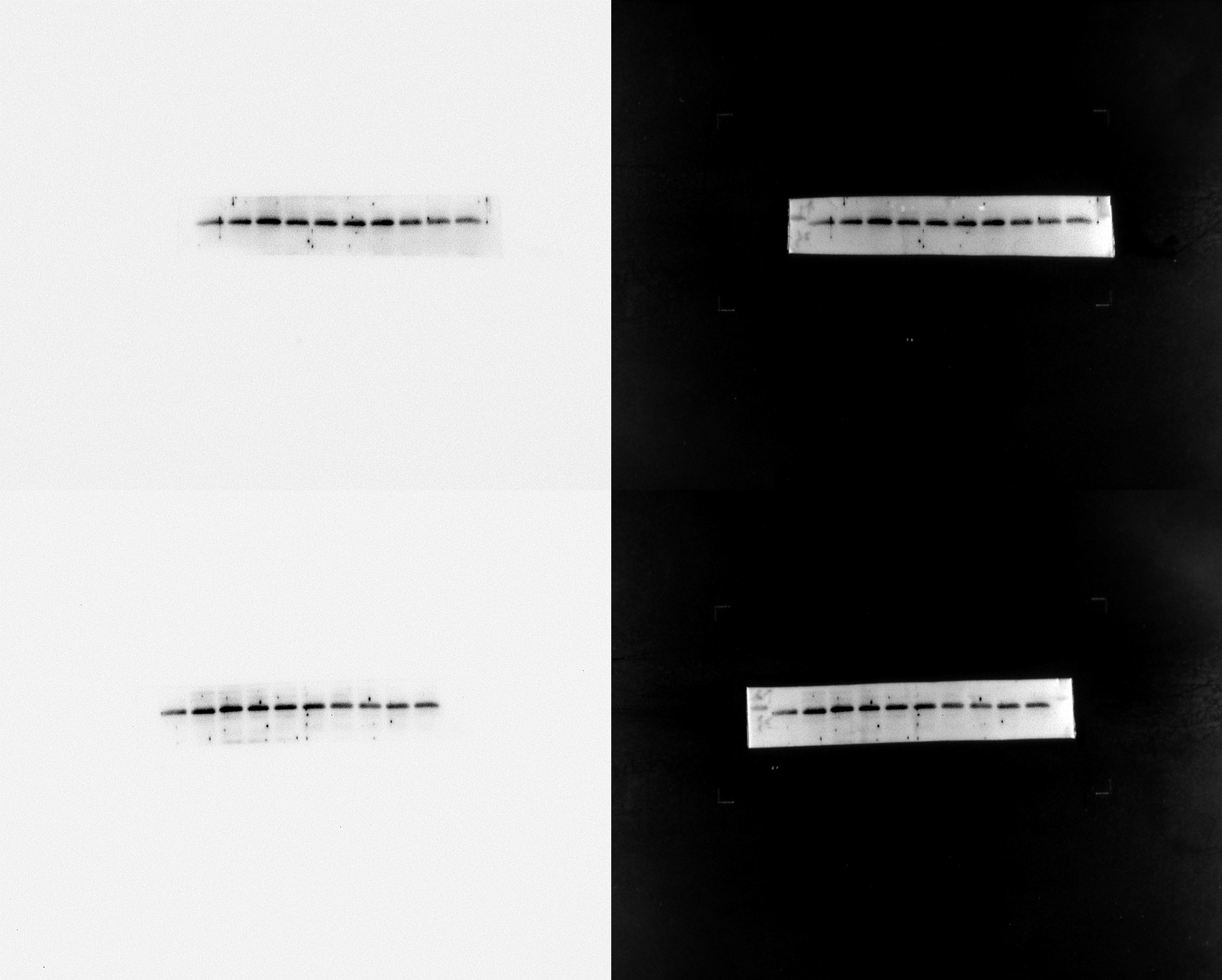

Supplement: Figure 2—source data 1. [file elife-96161-fig2-data1.zip › Figure 2-Source data1/Figure2I-Source data1-Claudin-5.png]

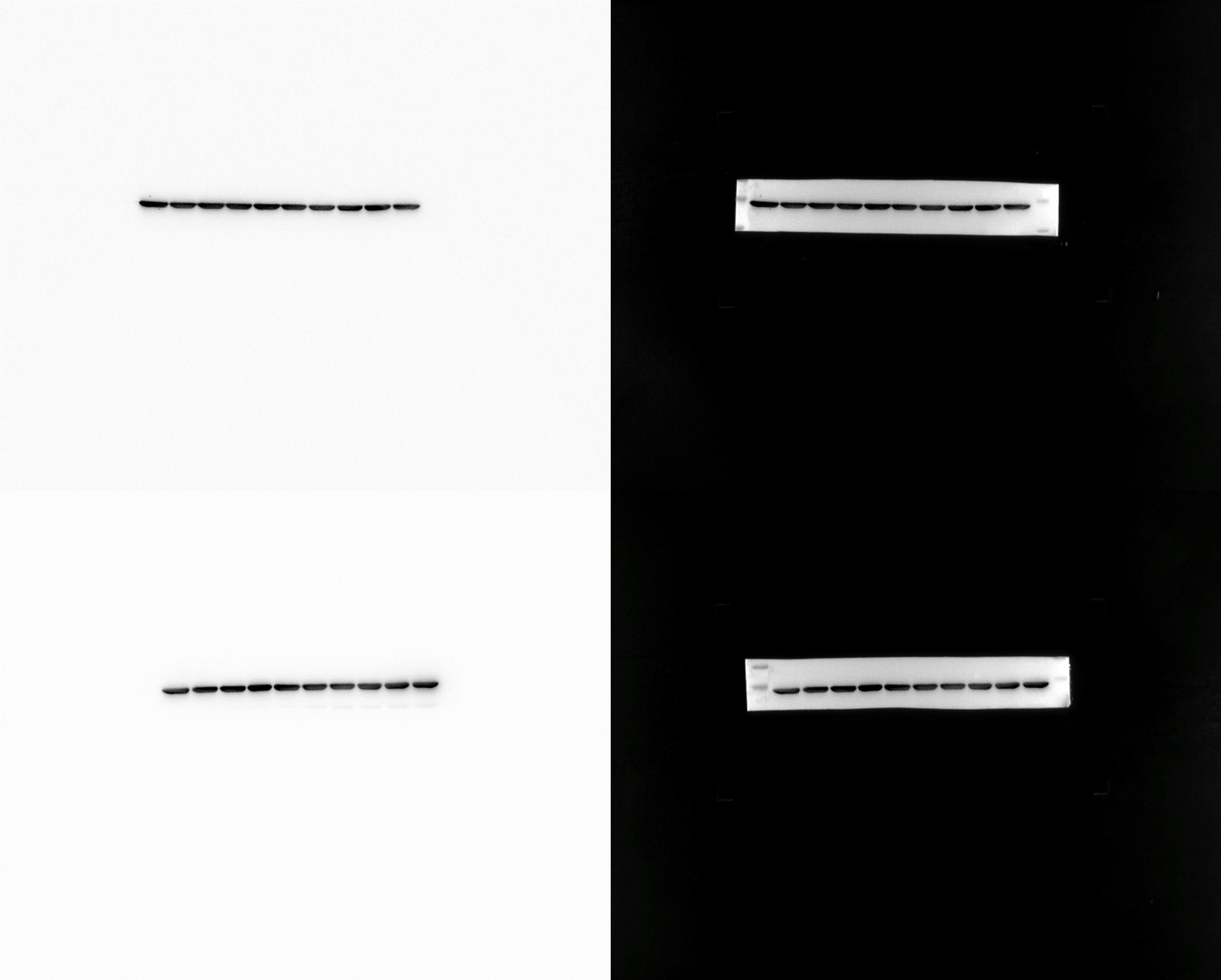

Supplement: Figure 2—source data 1. [file elife-96161-fig2-data1.zip › Figure 2-Source data1/Figure2I-Source data1-a┬-actin.png]

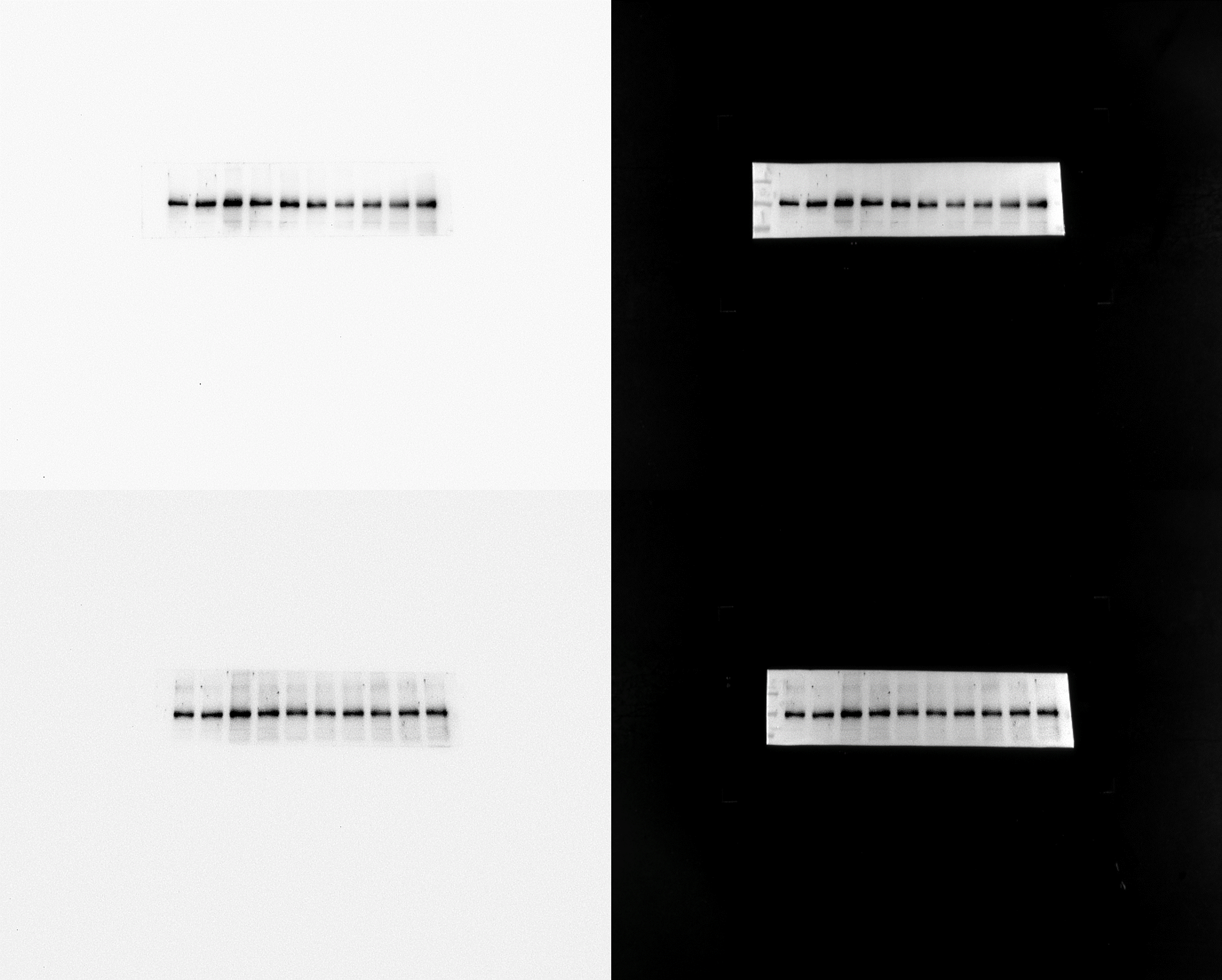

Supplement: Figure 2—source data 1. [file elife-96161-fig2-data1.zip › Figure 2-Source data1/Figure2I-Source data2-VE-Cadherin.png]

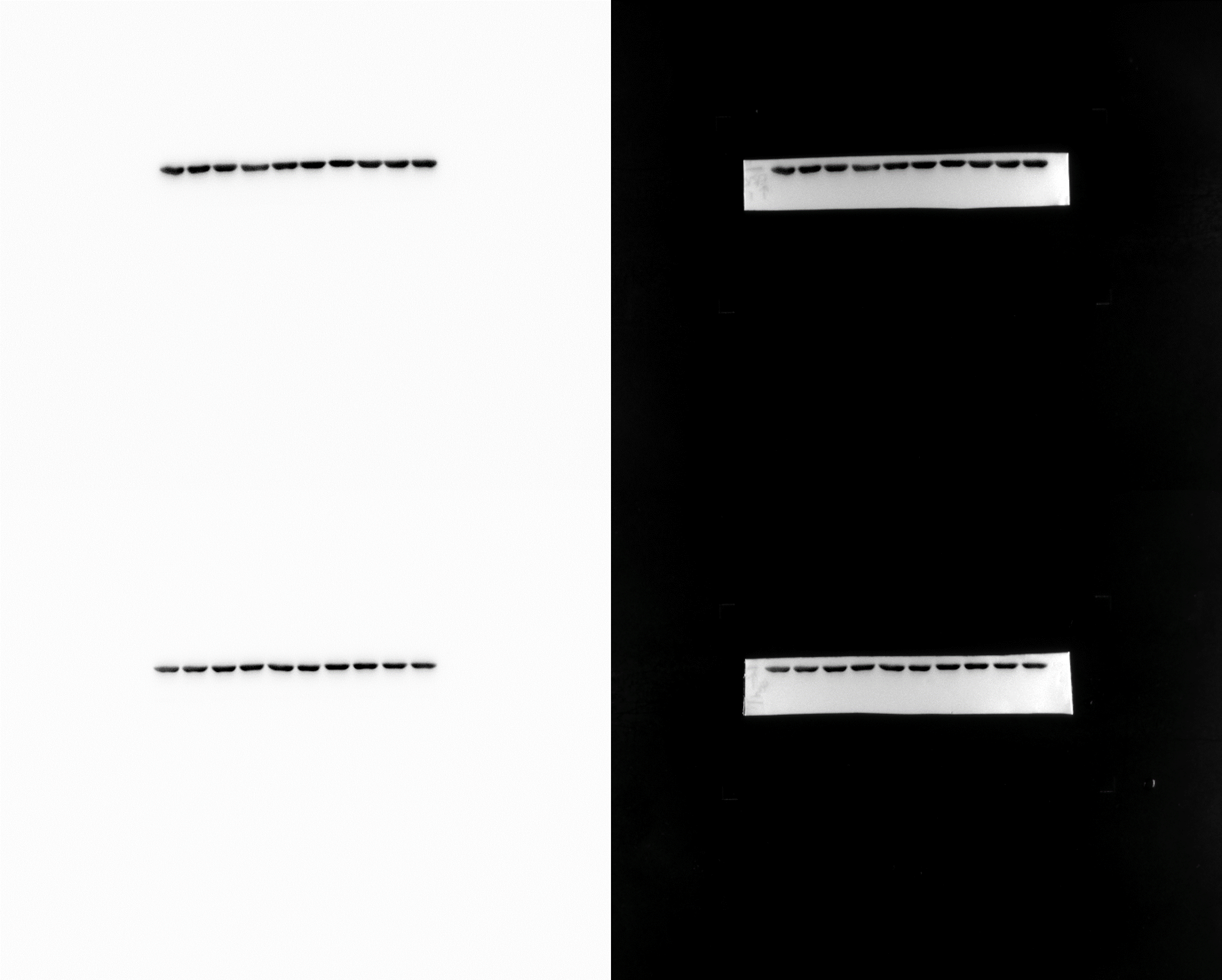

Supplement: Figure 2—source data 1. [file elife-96161-fig2-data1.zip › Figure 2-Source data1/Figure2I-Source data2-a┬-actin.png]

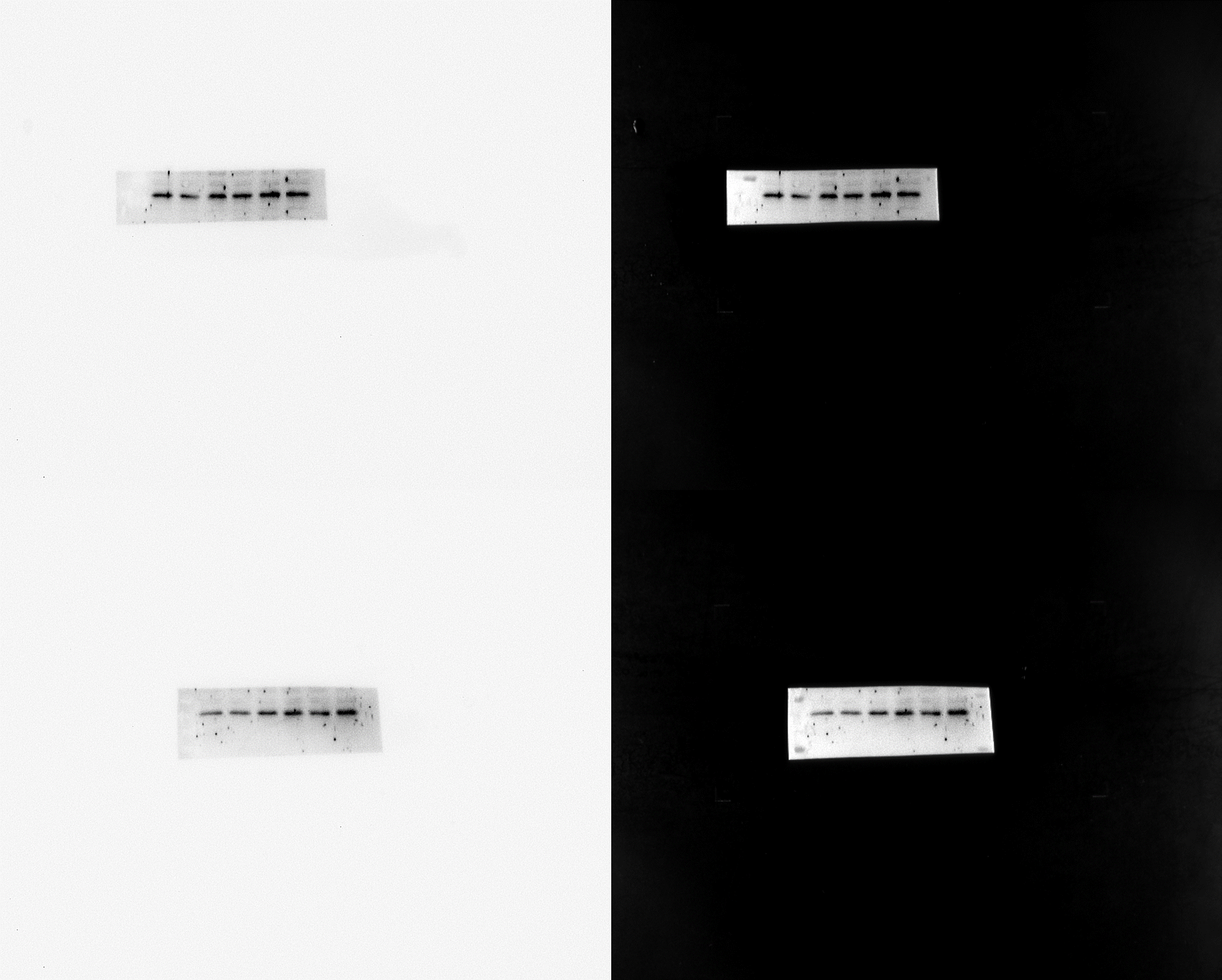

Supplement: Figure 2—source data 1. [file elife-96161-fig2-data1.zip › Figure 2-Source data1/Figure2J-Source data1-Claudin-5.png]

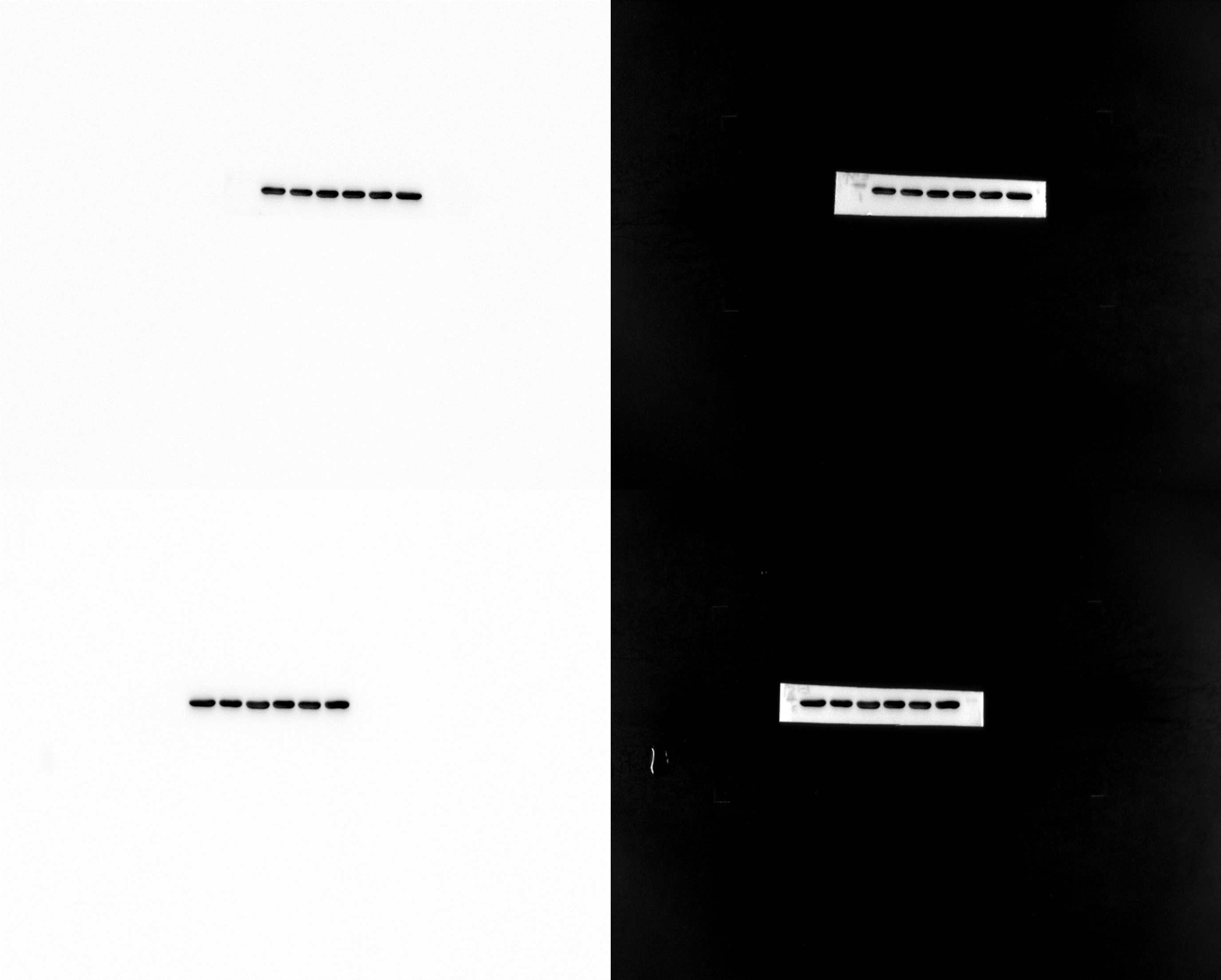

Supplement: Figure 2—source data 1. [file elife-96161-fig2-data1.zip › Figure 2-Source data1/Figure2J-Source data1-a┬-actin.png]

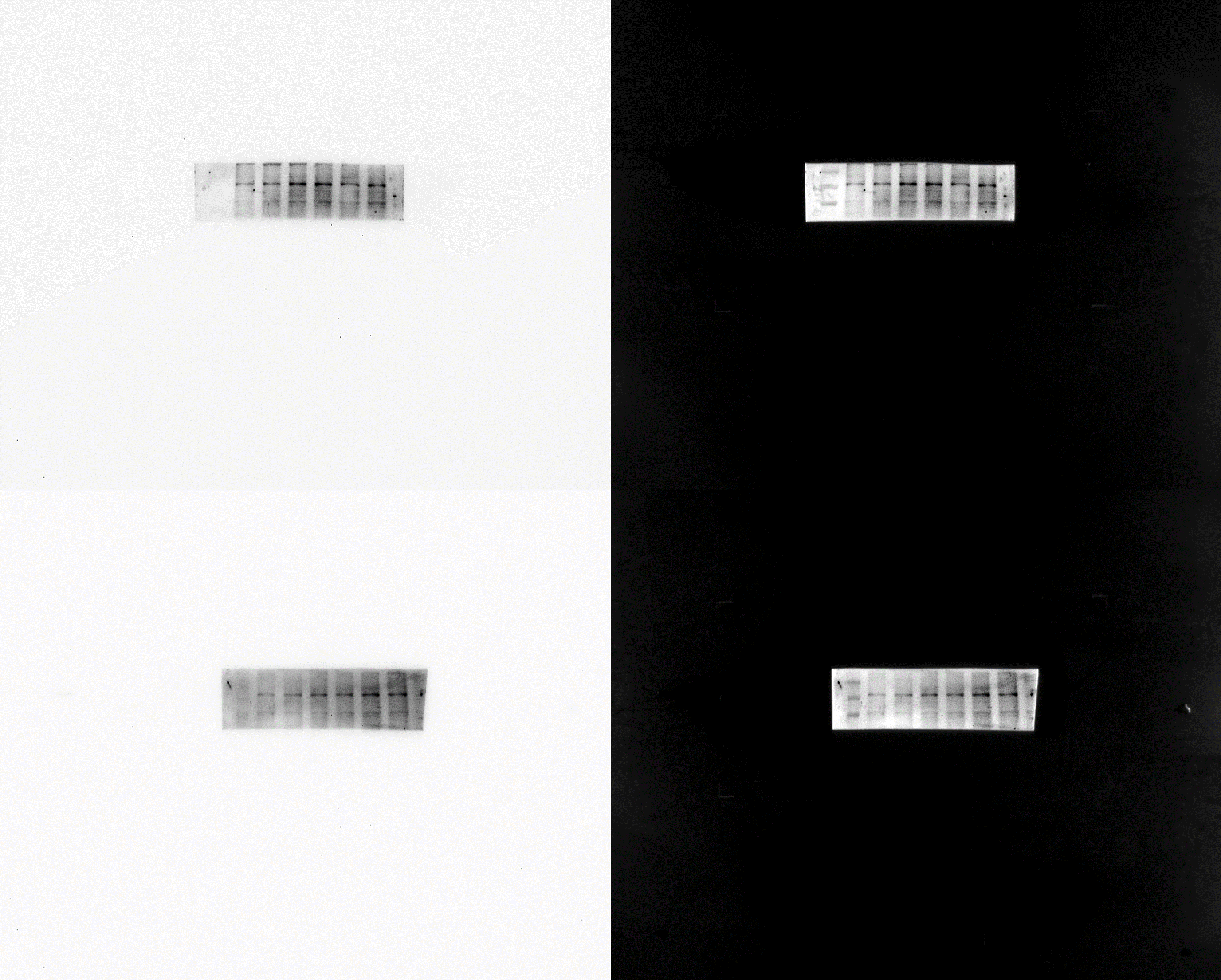

Supplement: Figure 2—source data 1. [file elife-96161-fig2-data1.zip › Figure 2-Source data1/Figure2J-Source data2-VE-Cadherin.png]

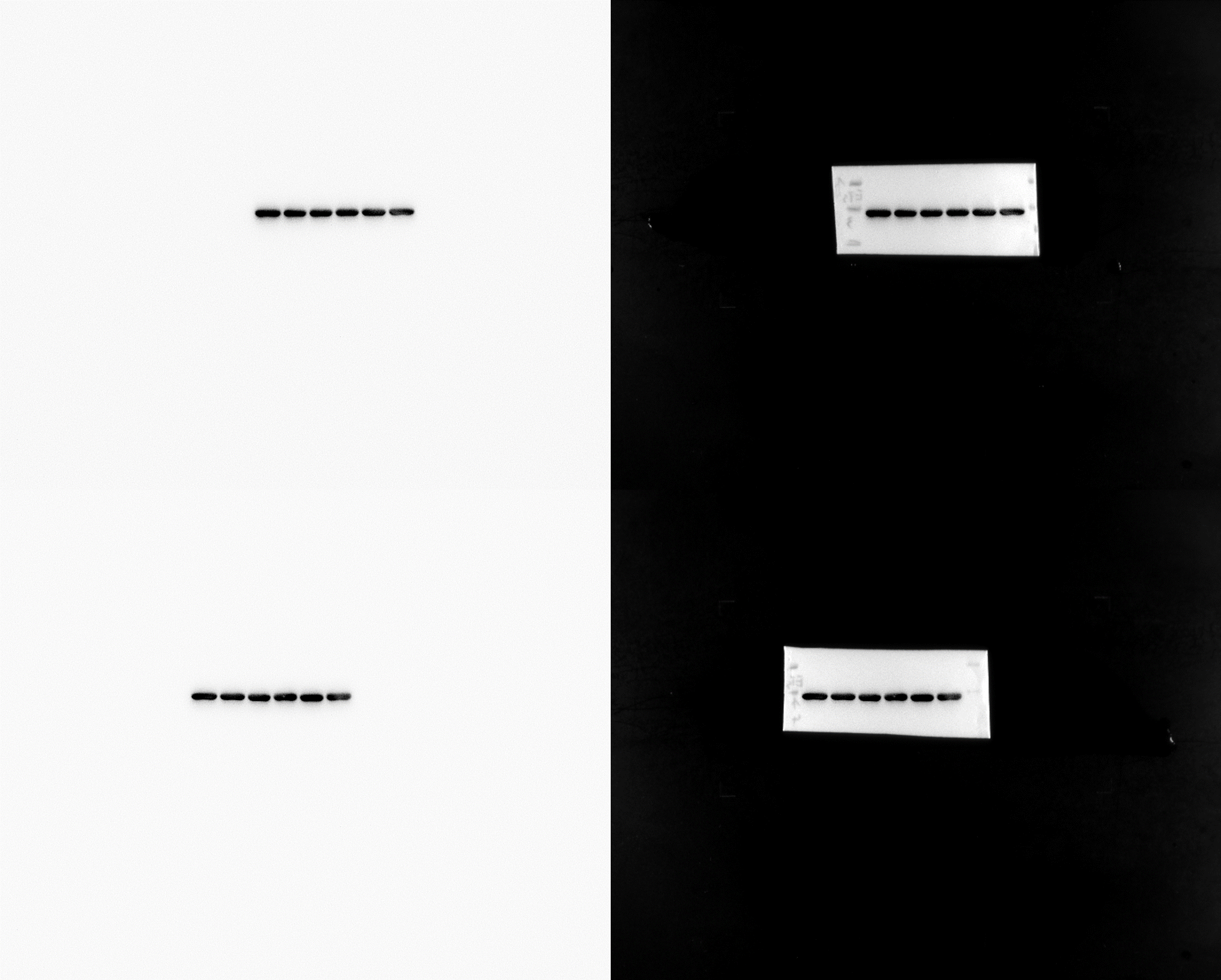

Supplement: Figure 2—source data 1. [file elife-96161-fig2-data1.zip › Figure 2-Source data1/Figure2J-Source data2-a┬-actin.png]

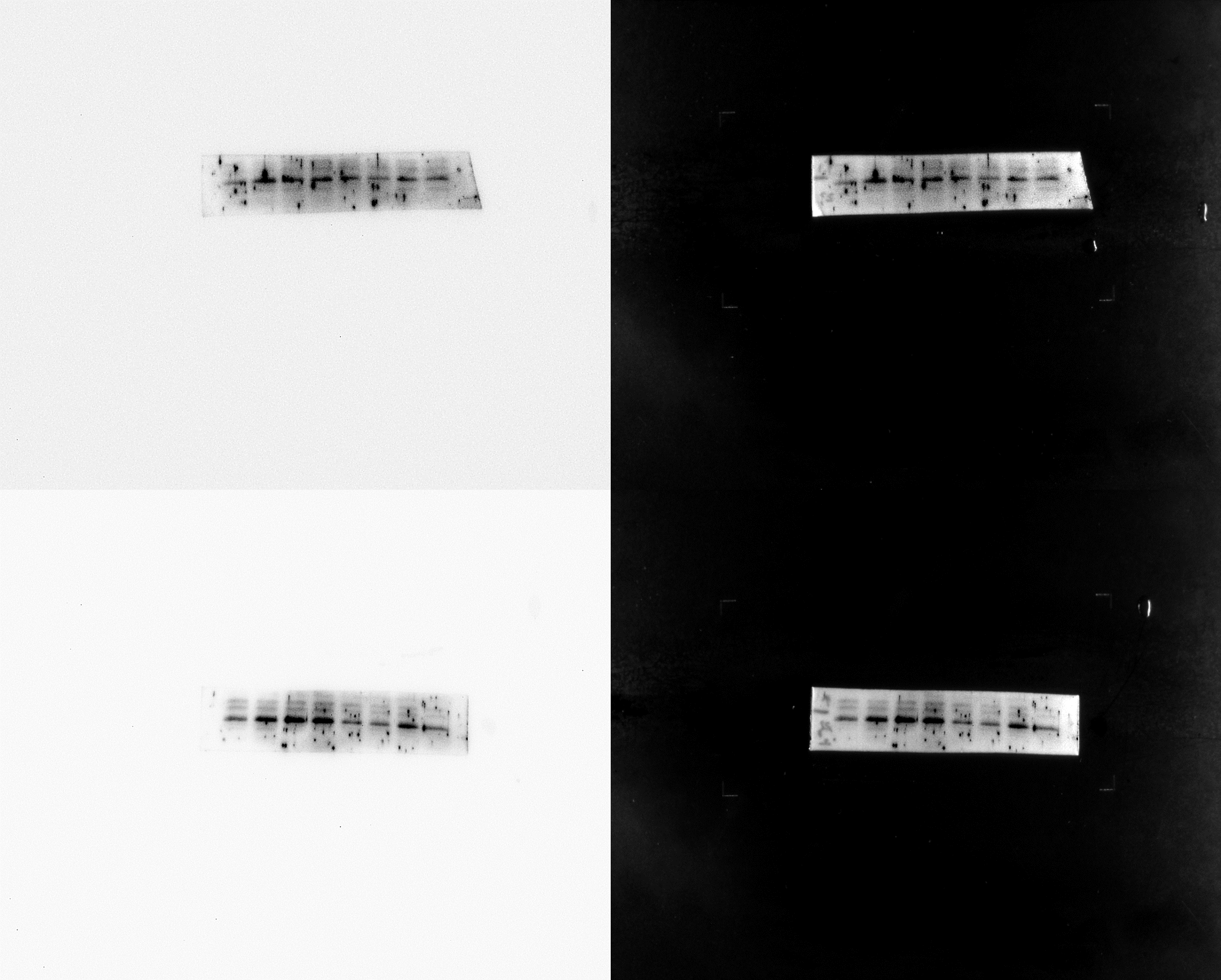

Supplement: Figure 2—source data 1. [file elife-96161-fig2-data1.zip › Figure 2-Source data1/Figure2K-Source data1-Claudin-5.png]

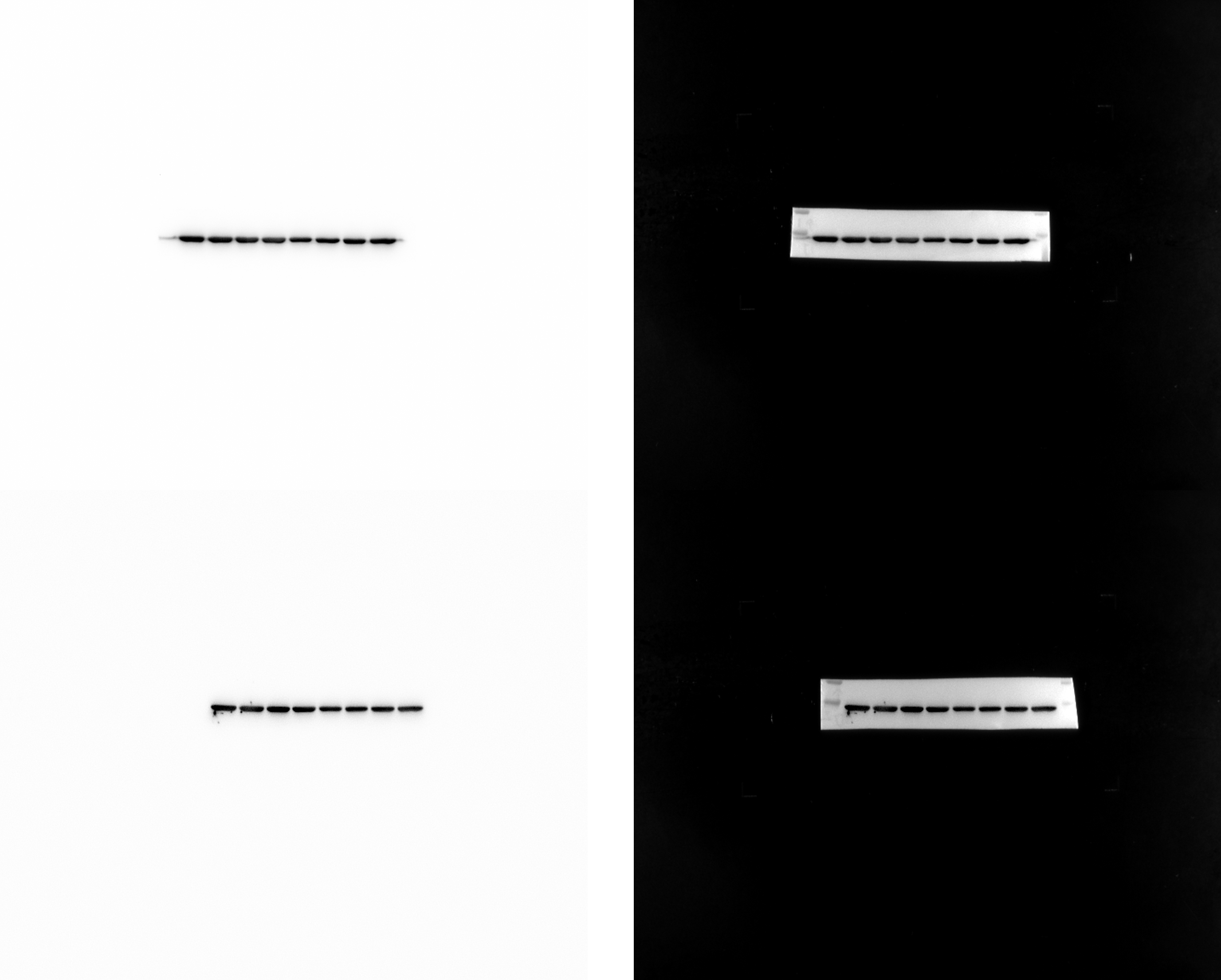

Supplement: Figure 2—source data 1. [file elife-96161-fig2-data1.zip › Figure 2-Source data1/Figure2K-Source data1-a┬-actin.png]

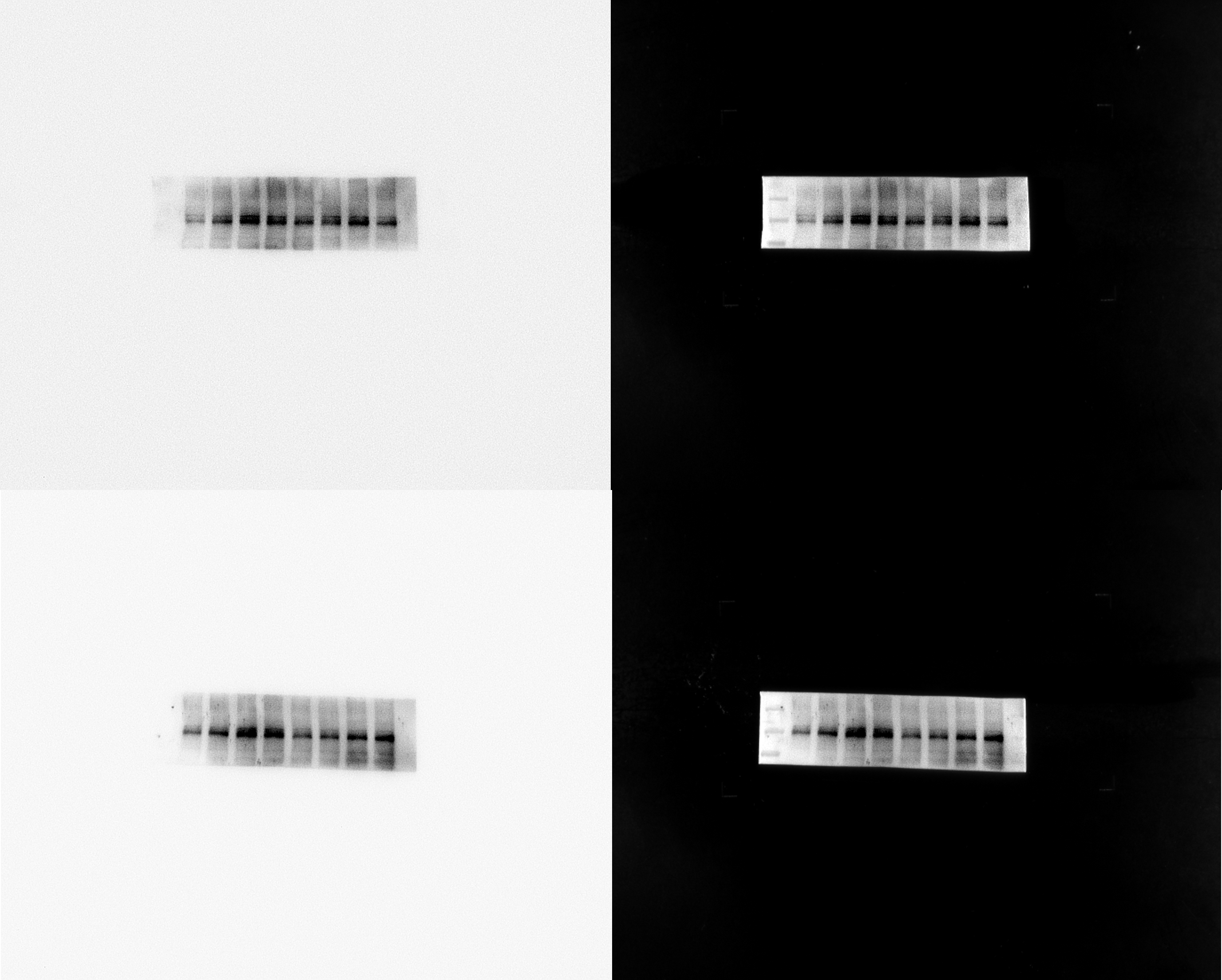

Supplement: Figure 2—source data 1. [file elife-96161-fig2-data1.zip › Figure 2-Source data1/Figure2K-Source data2-VE-Cadherin.png]

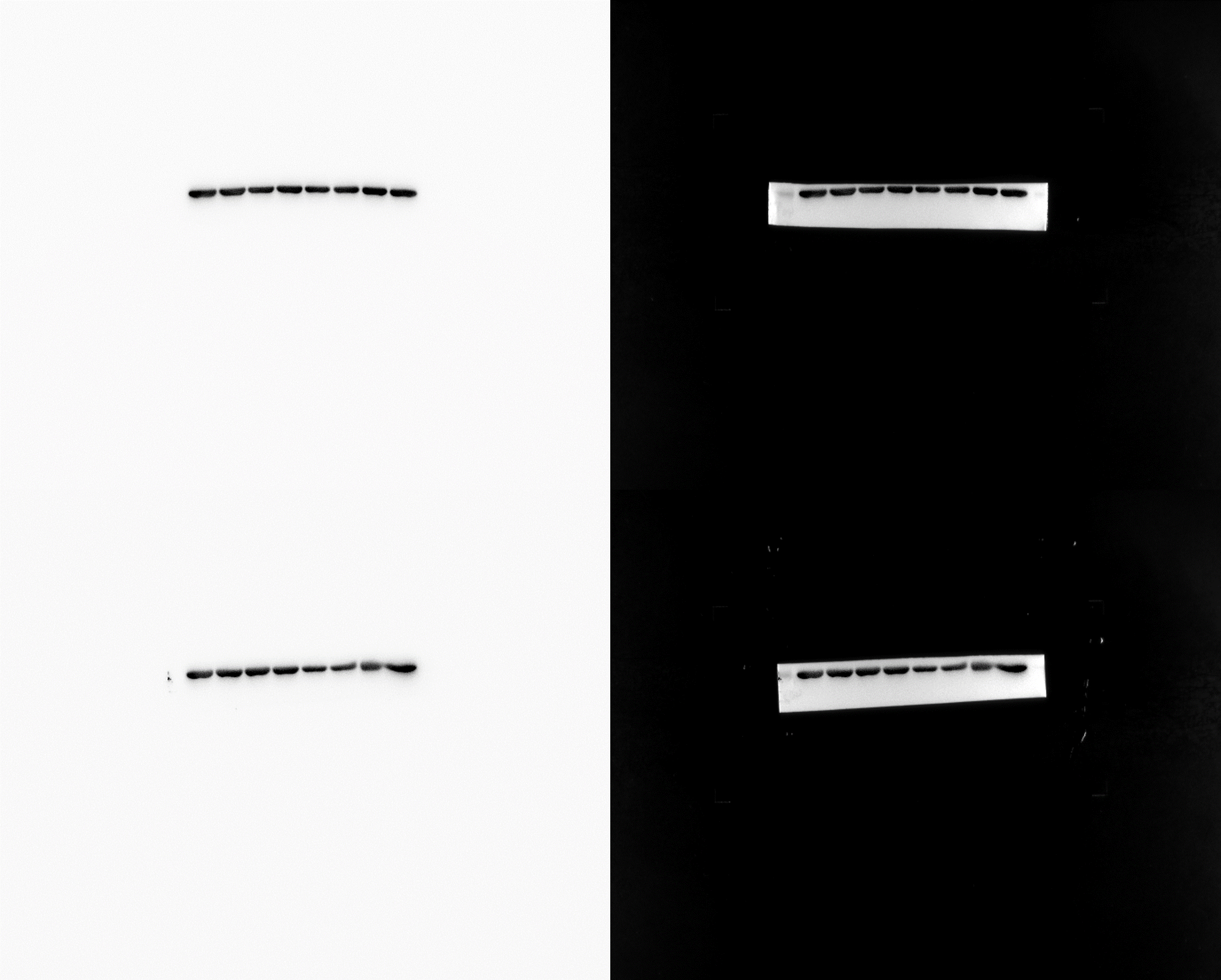

Supplement: Figure 2—source data 1. [file elife-96161-fig2-data1.zip › Figure 2-Source data1/Figure2K-Source data2-a┬-actin.png]

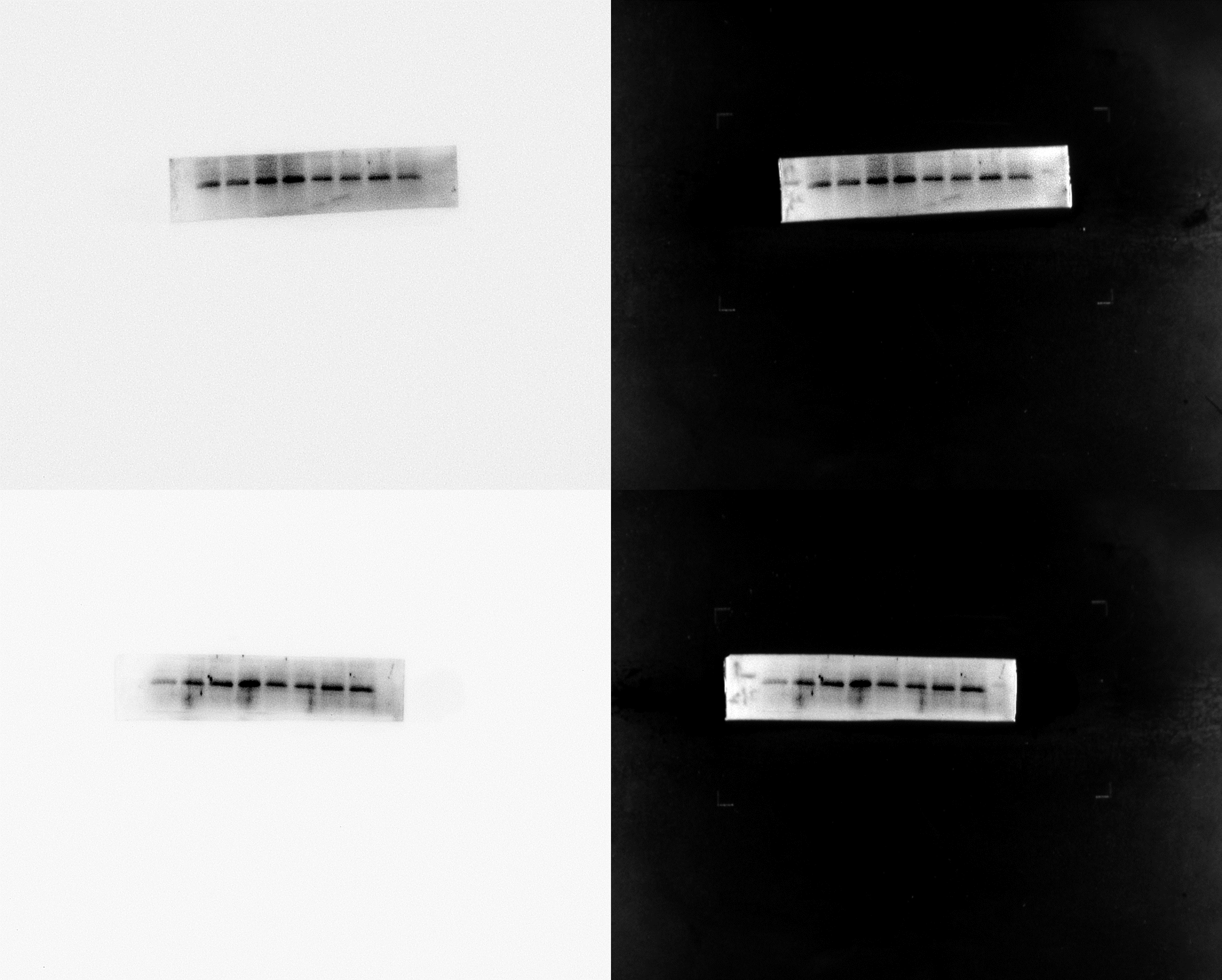

Supplement: Figure 2—source data 1. [file elife-96161-fig2-data1.zip › Figure 2-Source data1/Figure2L-Source data1-Claudin-5.png]

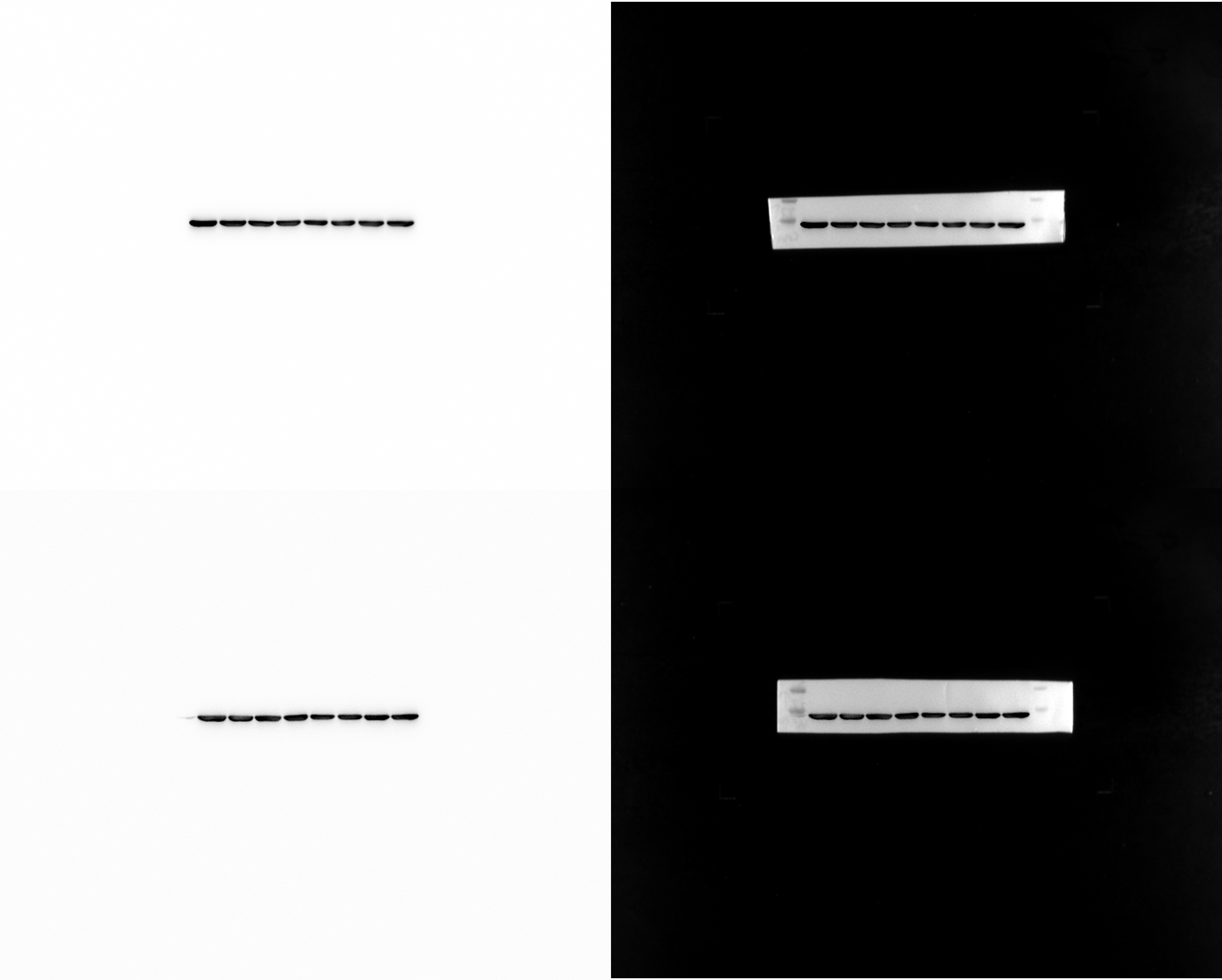

Supplement: Figure 2—source data 1. [file elife-96161-fig2-data1.zip › Figure 2-Source data1/Figure2L-Source data1-a┬-actin.png]

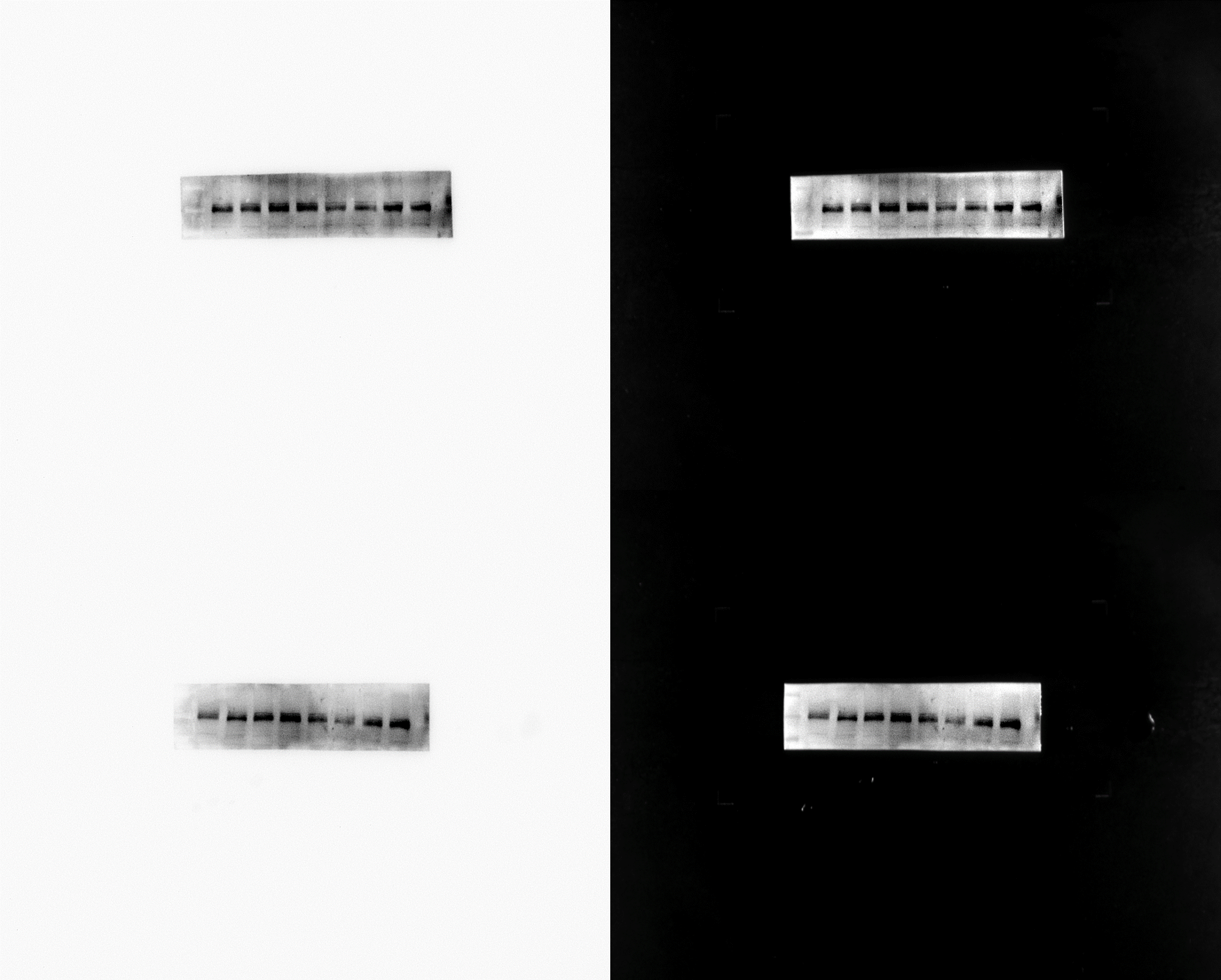

Supplement: Figure 2—source data 1. [file elife-96161-fig2-data1.zip › Figure 2-Source data1/Figure2L-Source data2-VE-Cadherin.png]

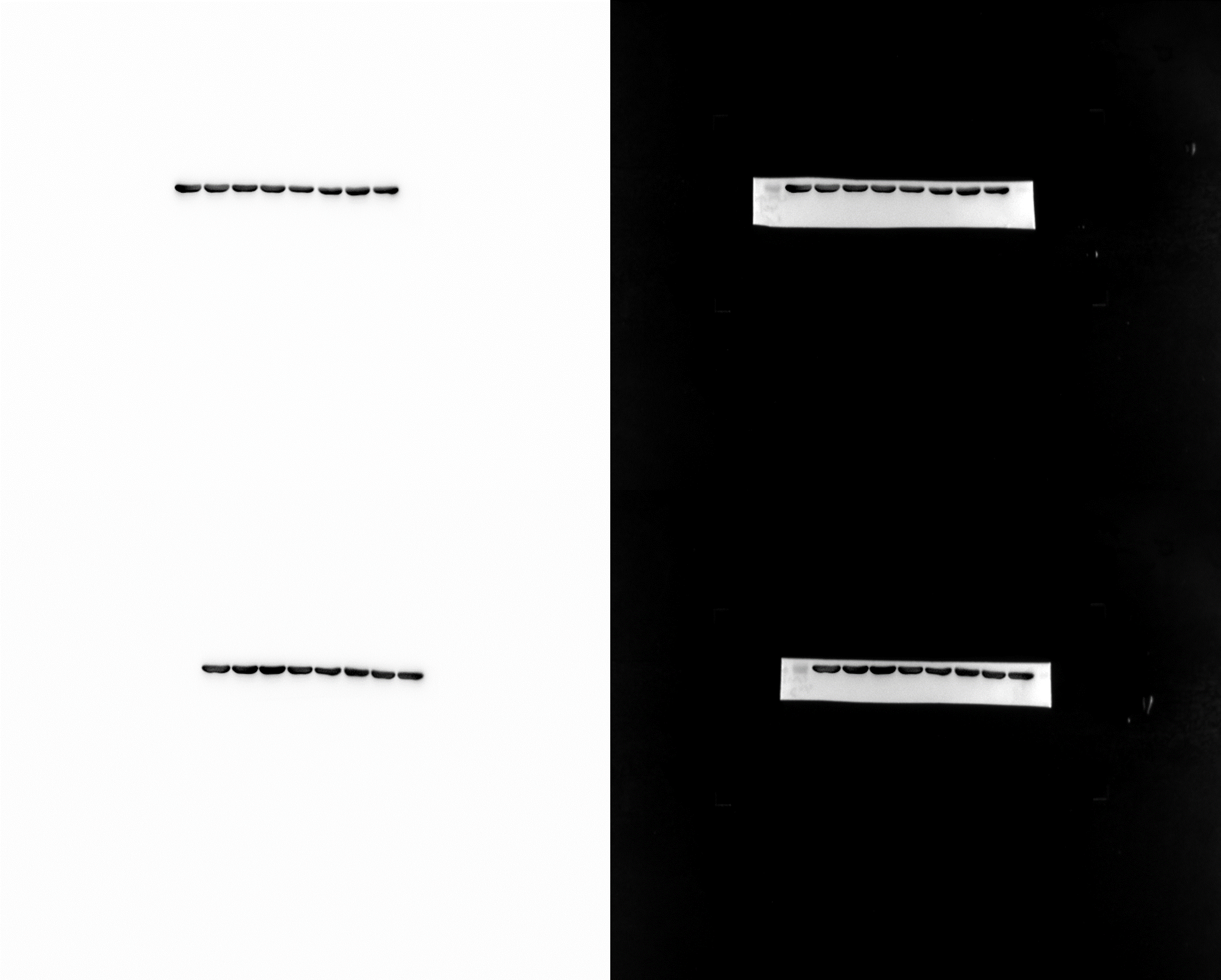

Supplement: Figure 2—source data 1. [file elife-96161-fig2-data1.zip › Figure 2-Source data1/Figure2L-Source data2-a┬-actin.png]

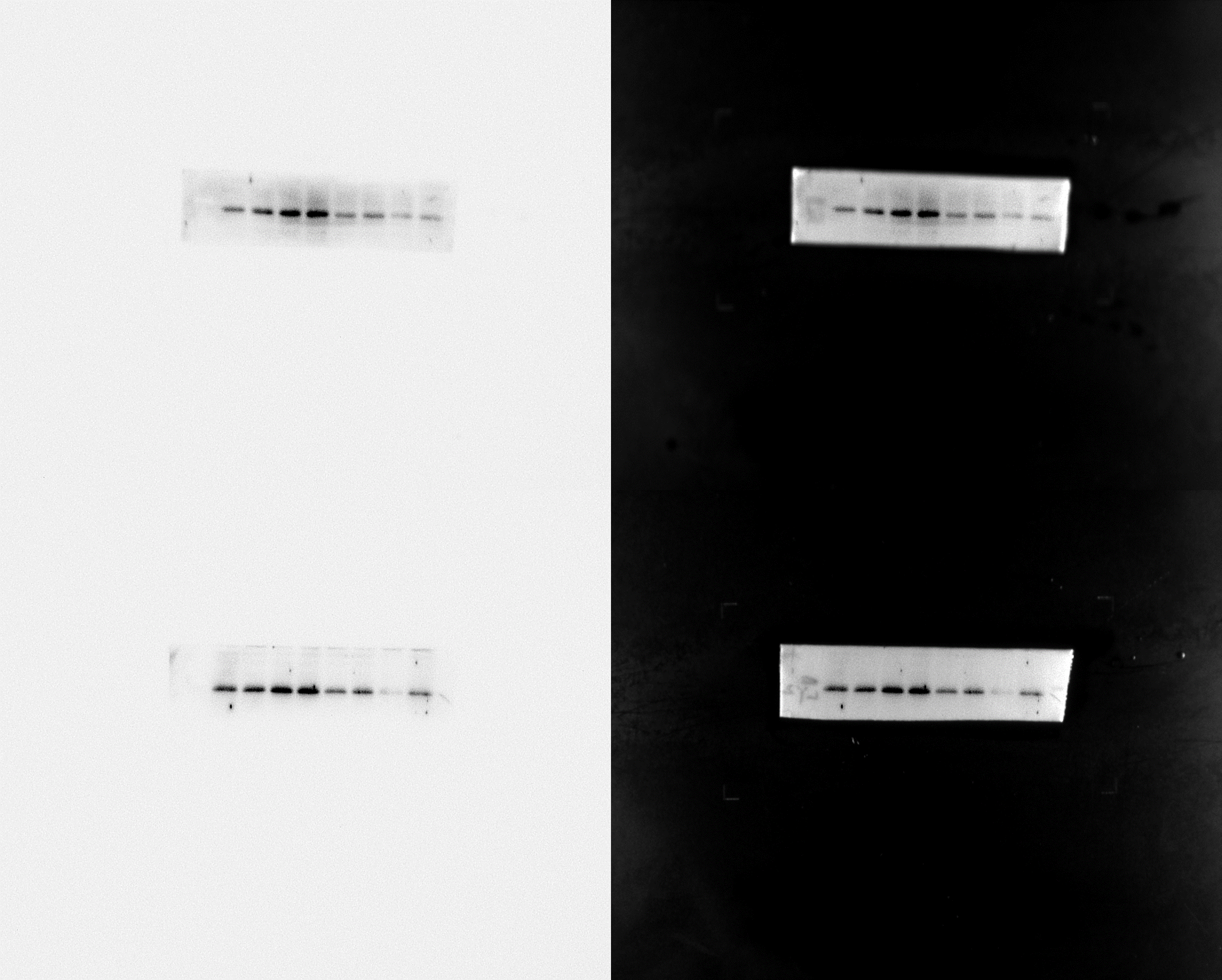

Supplement: Figure 3—source data 1. [file elife-96161-fig3-data1.zip › Figure 3-Source data1/Figure3A-Source data1-Claudin-5.png]

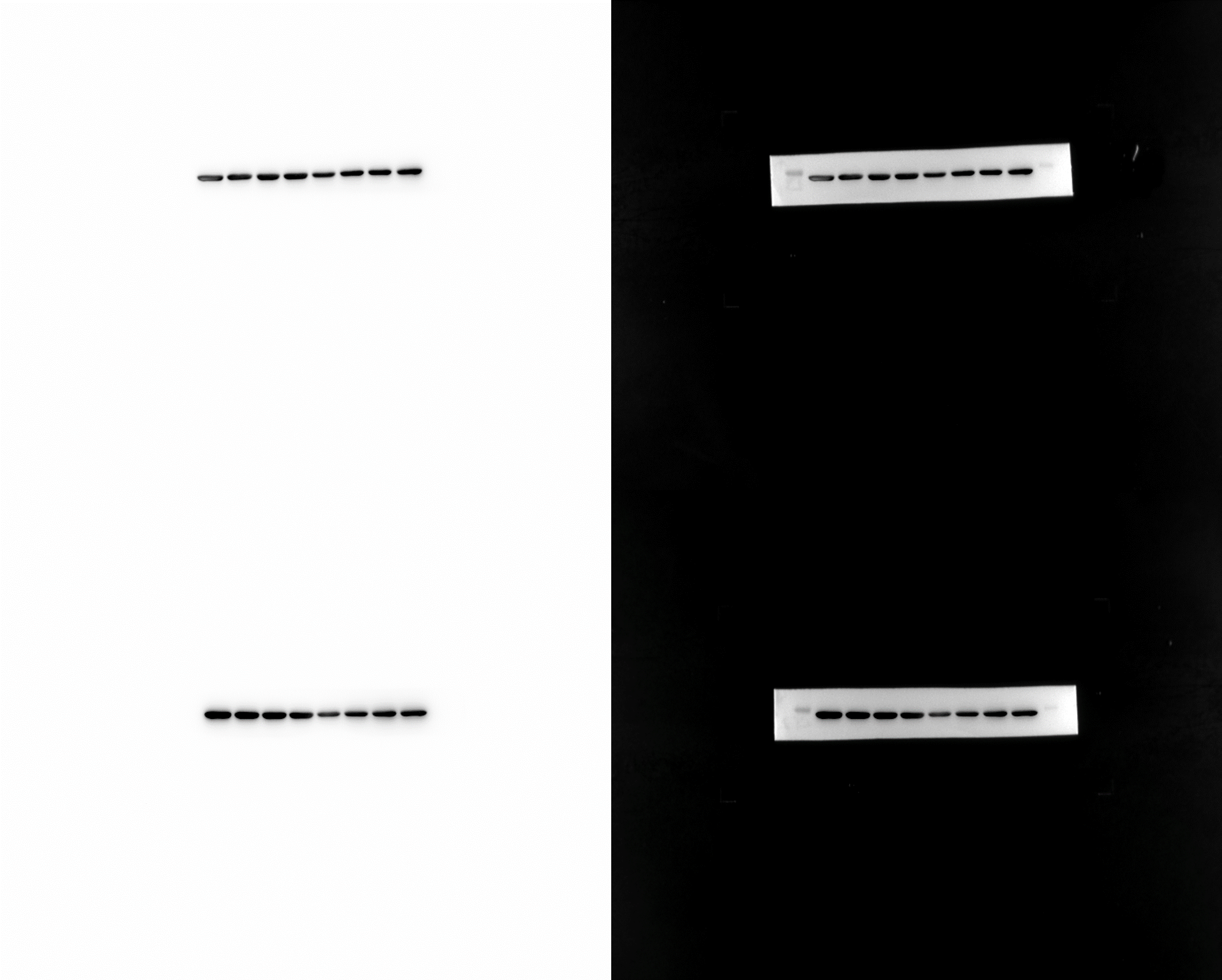

Supplement: Figure 3—source data 1. [file elife-96161-fig3-data1.zip › Figure 3-Source data1/Figure3A-Source data1-a┬-actin.png]

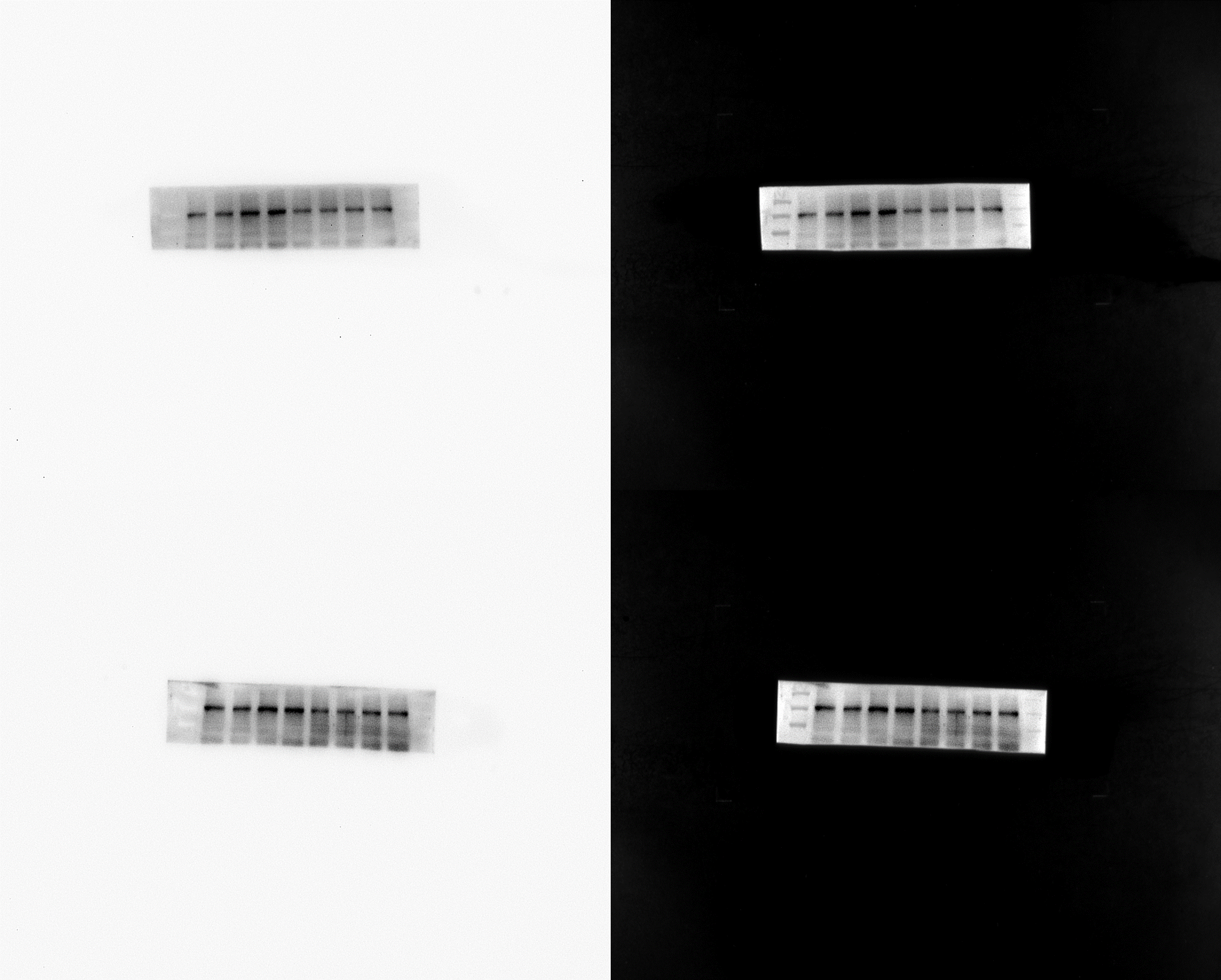

Supplement: Figure 3—source data 1. [file elife-96161-fig3-data1.zip › Figure 3-Source data1/Figure3A-Source data2-VE-Cadherin.png]

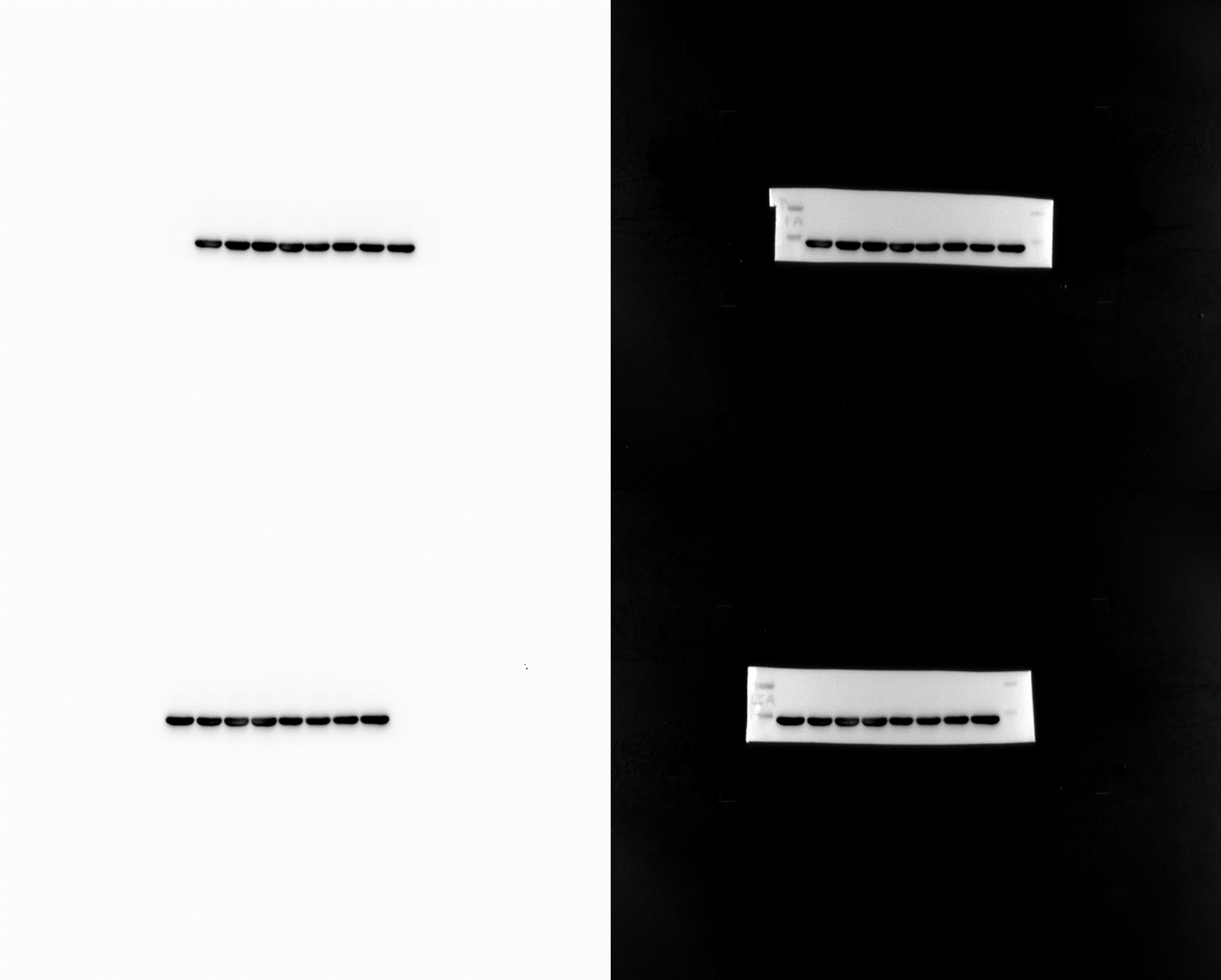

Supplement: Figure 3—source data 1. [file elife-96161-fig3-data1.zip › Figure 3-Source data1/Figure3A-Source data2-a┬-actin.png]

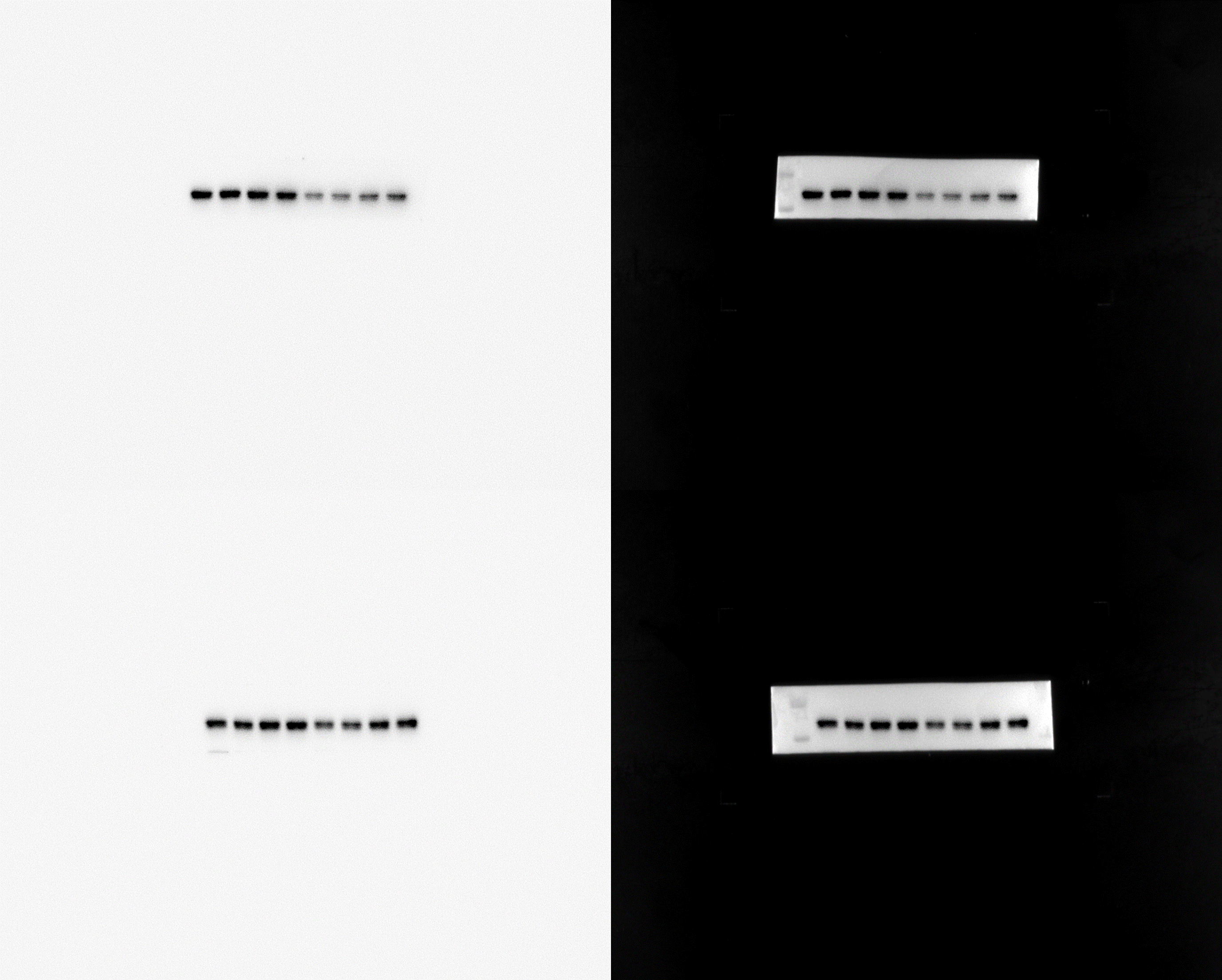

Supplement: Figure 3—source data 1. [file elife-96161-fig3-data1.zip › Figure 3-Source data1/Figure3A-Source data3-AKT.png]

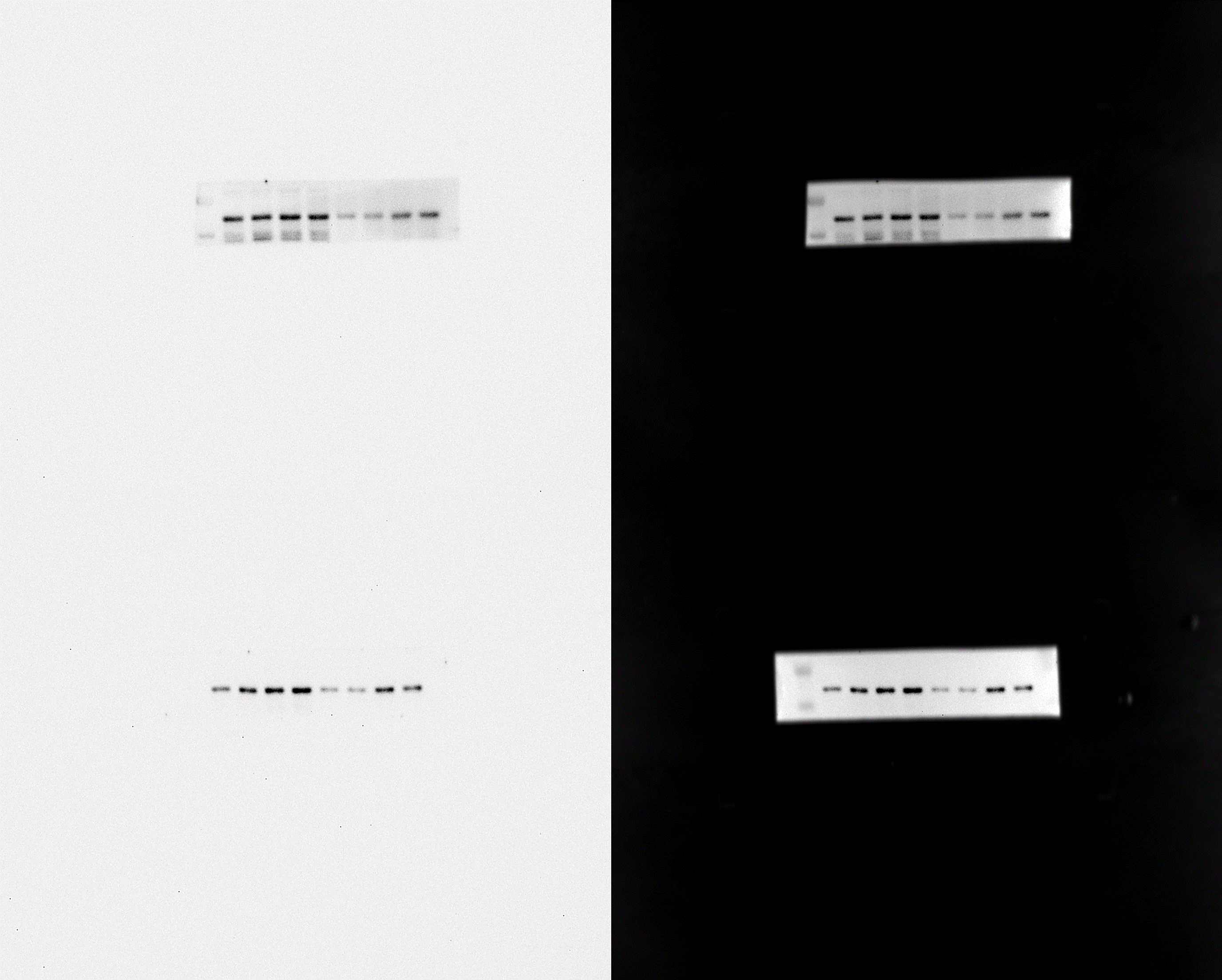

Supplement: Figure 3—source data 1. [file elife-96161-fig3-data1.zip › Figure 3-Source data1/Figure3A-Source data3-p-AKT.png]

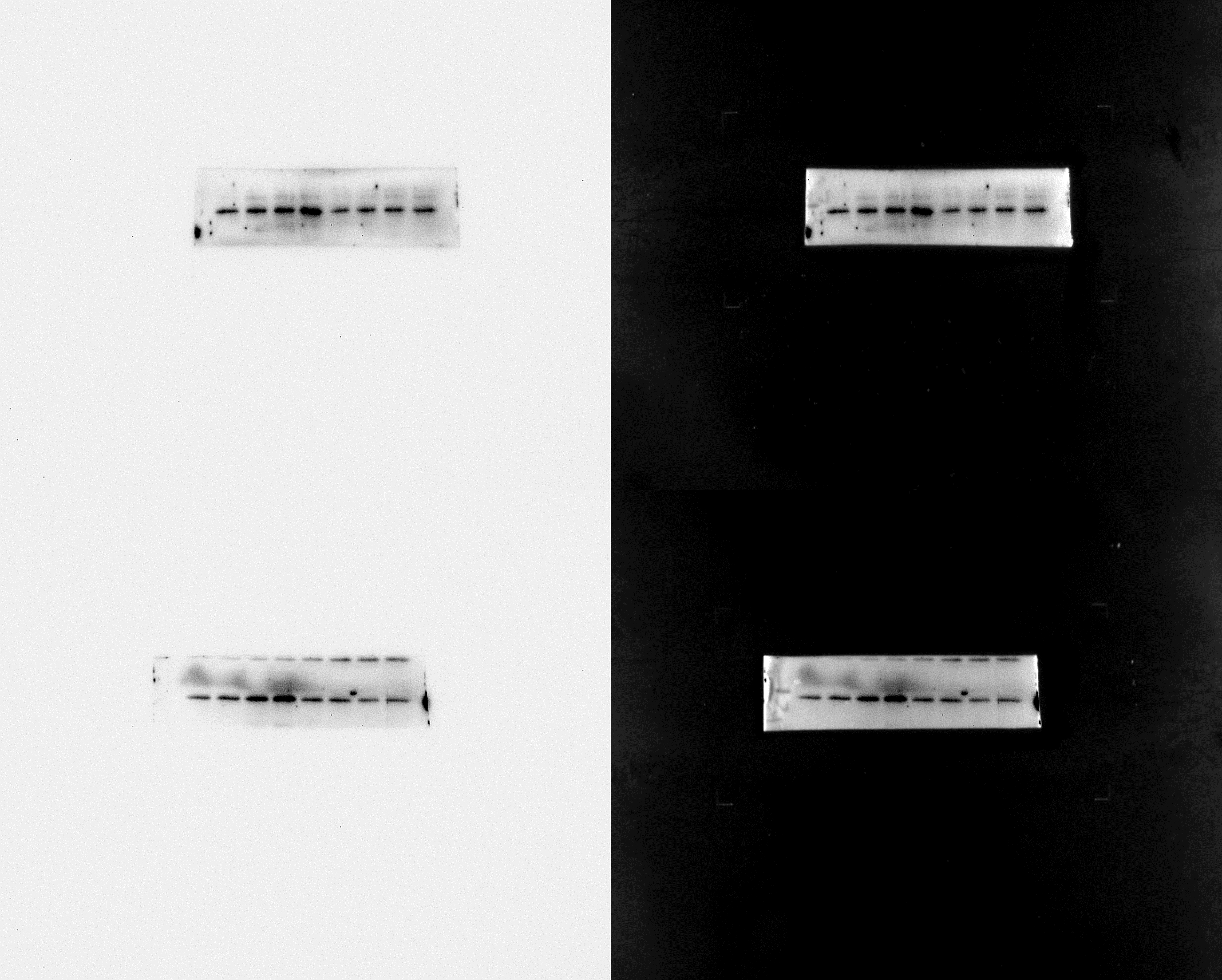

Supplement: Figure 3—source data 1. [file elife-96161-fig3-data1.zip › Figure 3-Source data1/Figure3B-Source data1-Claudin-5.png]

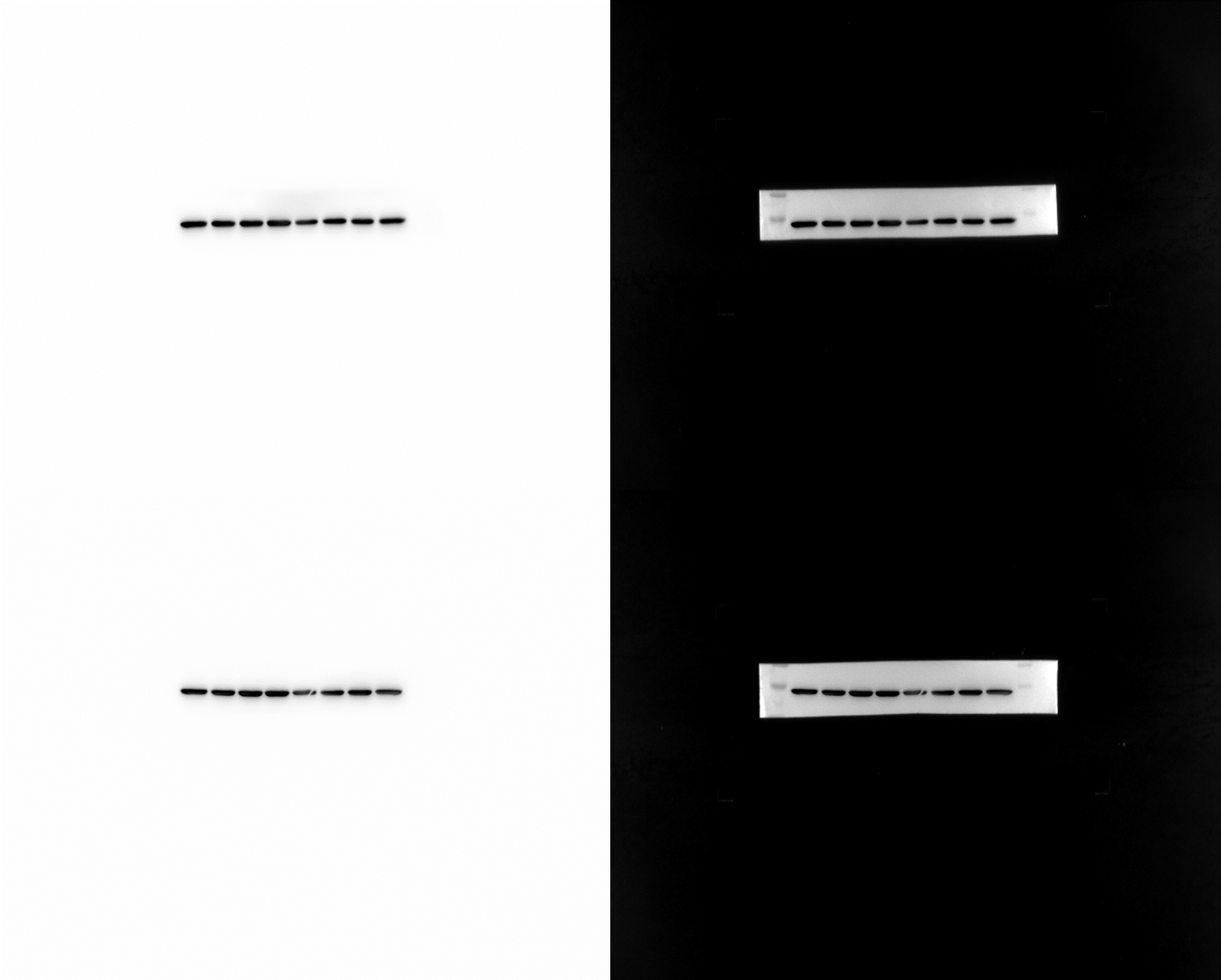

Supplement: Figure 3—source data 1. [file elife-96161-fig3-data1.zip › Figure 3-Source data1/Figure3B-Source data1-a┬-actin.png]

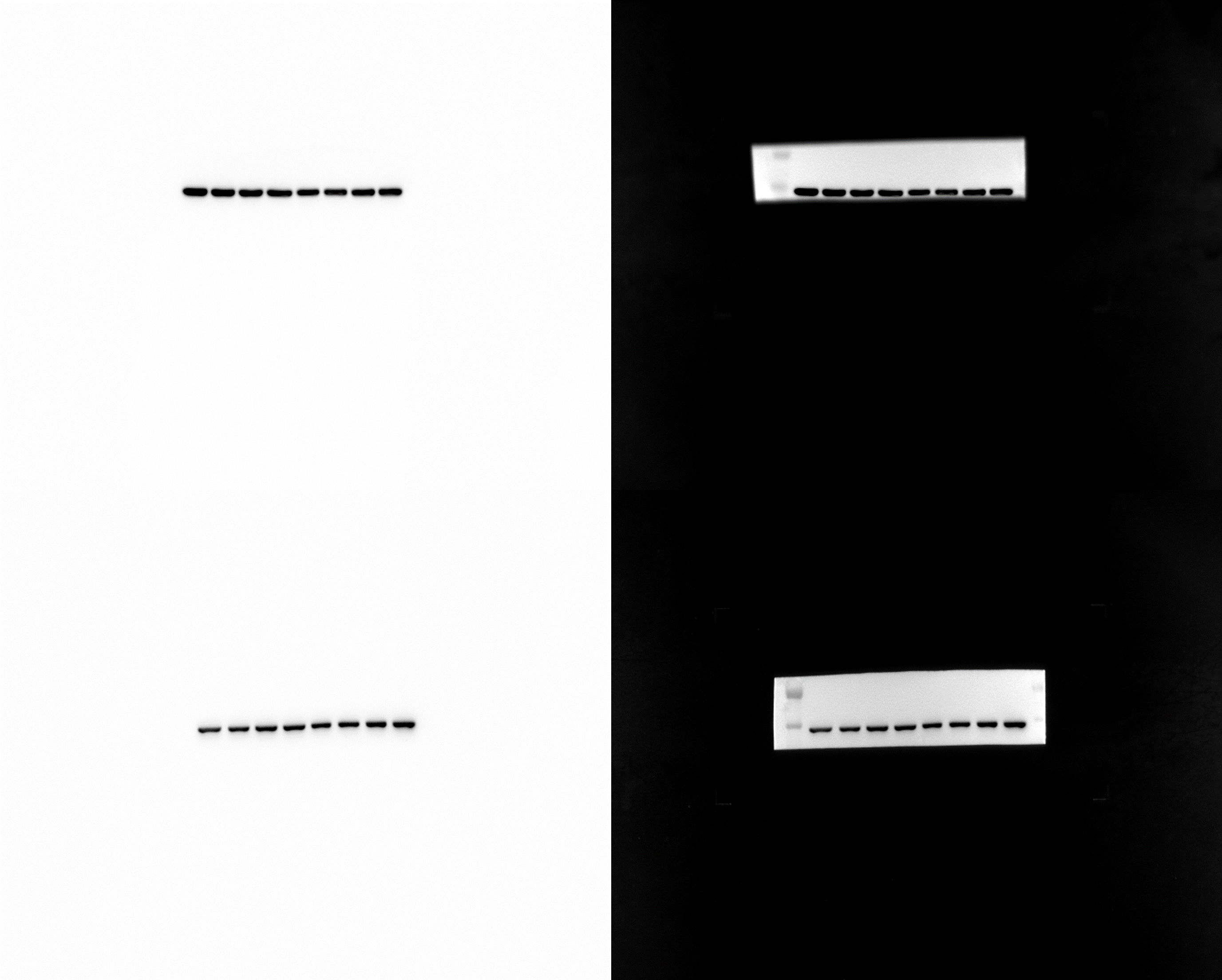

Supplement: Figure 3—source data 1. [file elife-96161-fig3-data1.zip › Figure 3-Source data1/Figure3B-Source data2-Tubulin.png]

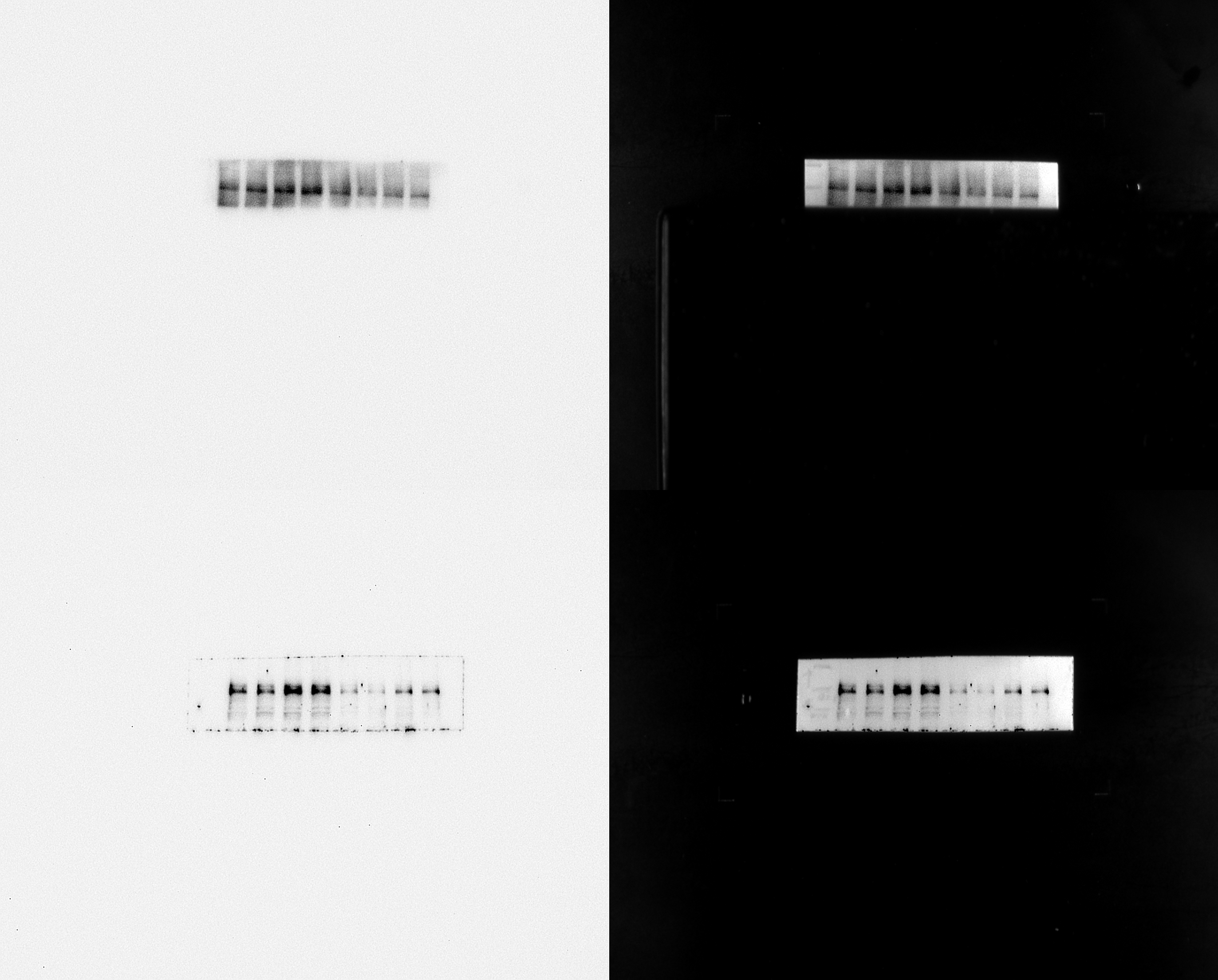

Supplement: Figure 3—source data 1. [file elife-96161-fig3-data1.zip › Figure 3-Source data1/Figure3B-Source data2-VE-Cadherin.png]

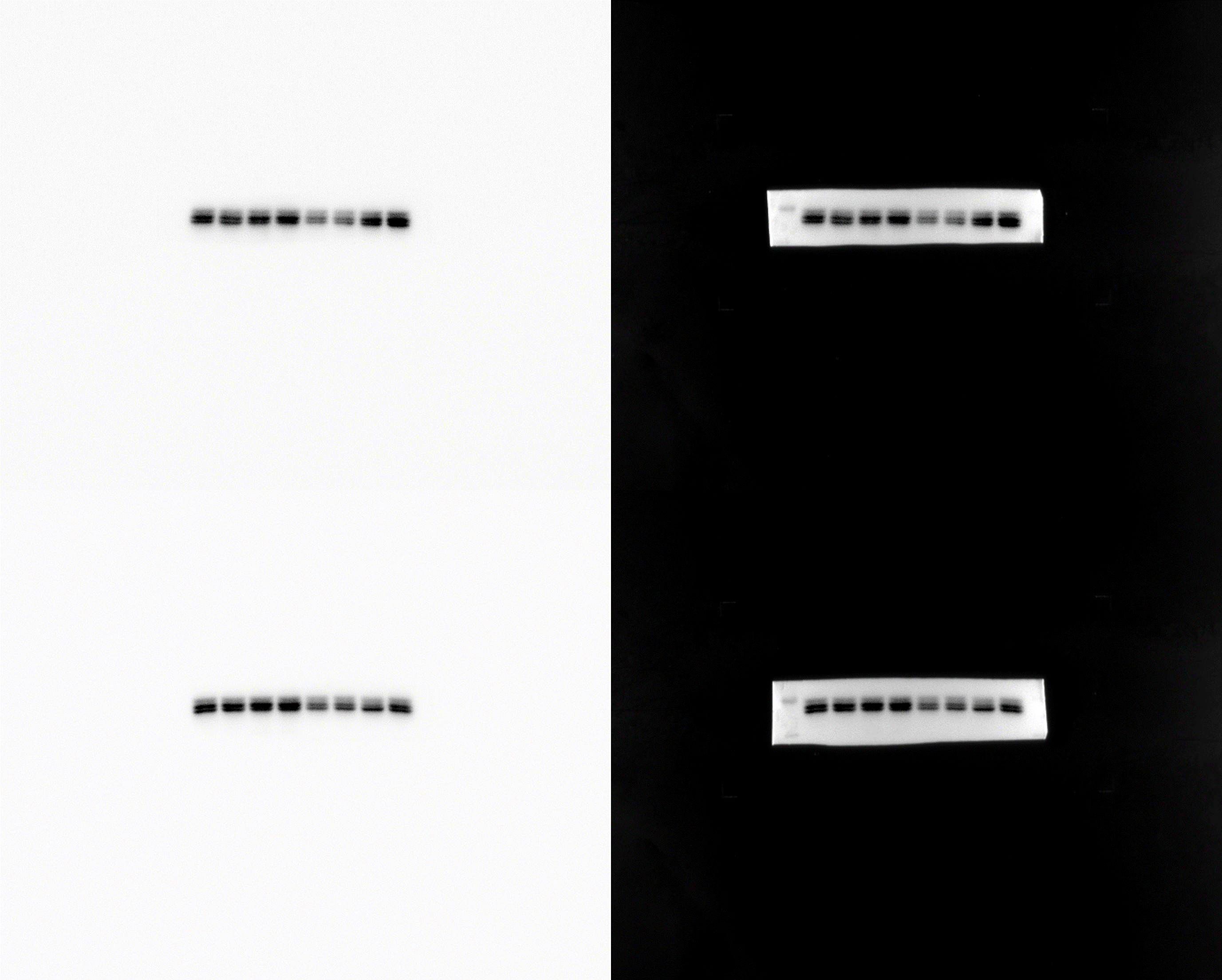

Supplement: Figure 3—source data 1. [file elife-96161-fig3-data1.zip › Figure 3-Source data1/Figure3B-Source data3-ERK.png]

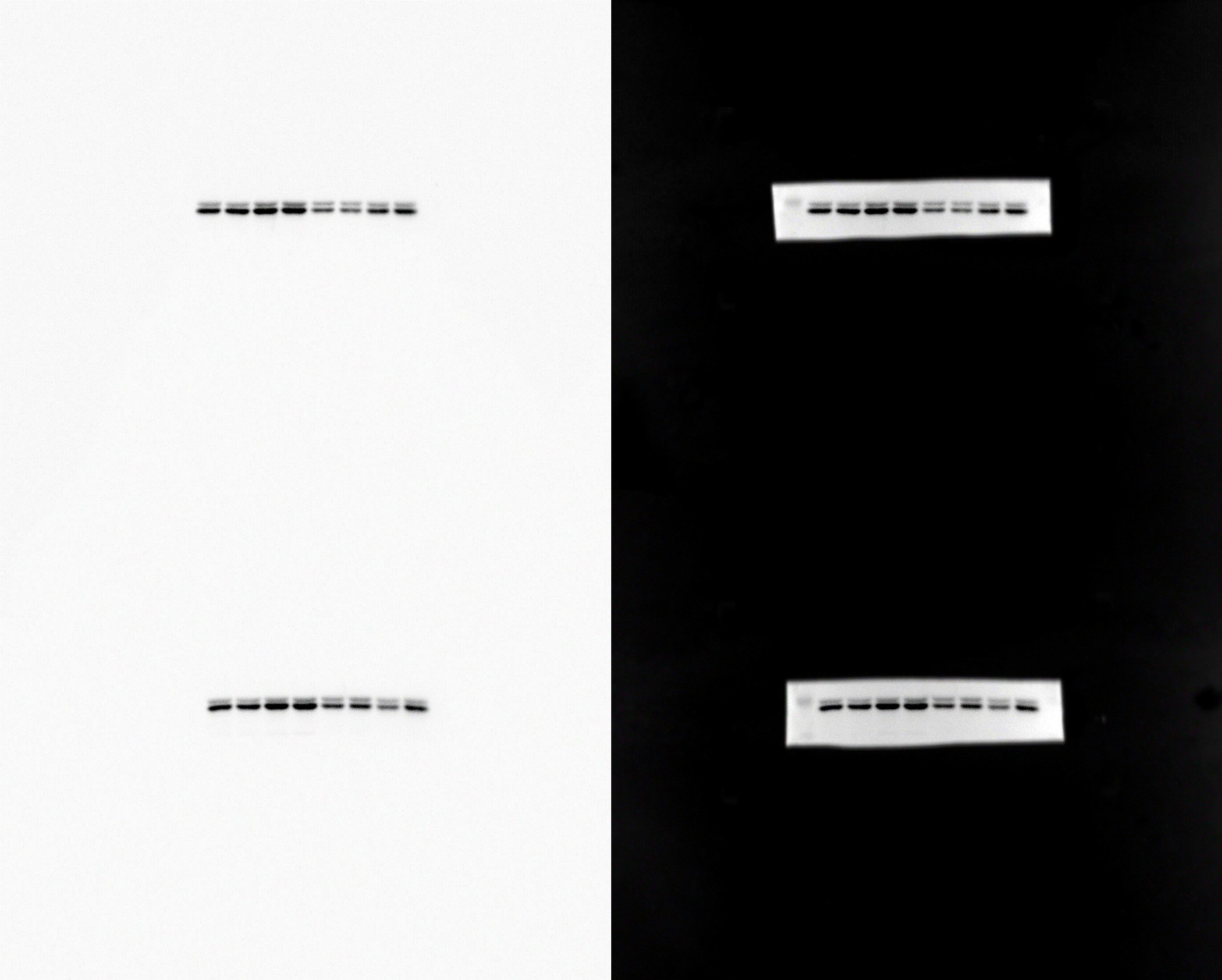

Supplement: Figure 3—source data 1. [file elife-96161-fig3-data1.zip › Figure 3-Source data1/Figure3B-Source data3-p-ERK.png]

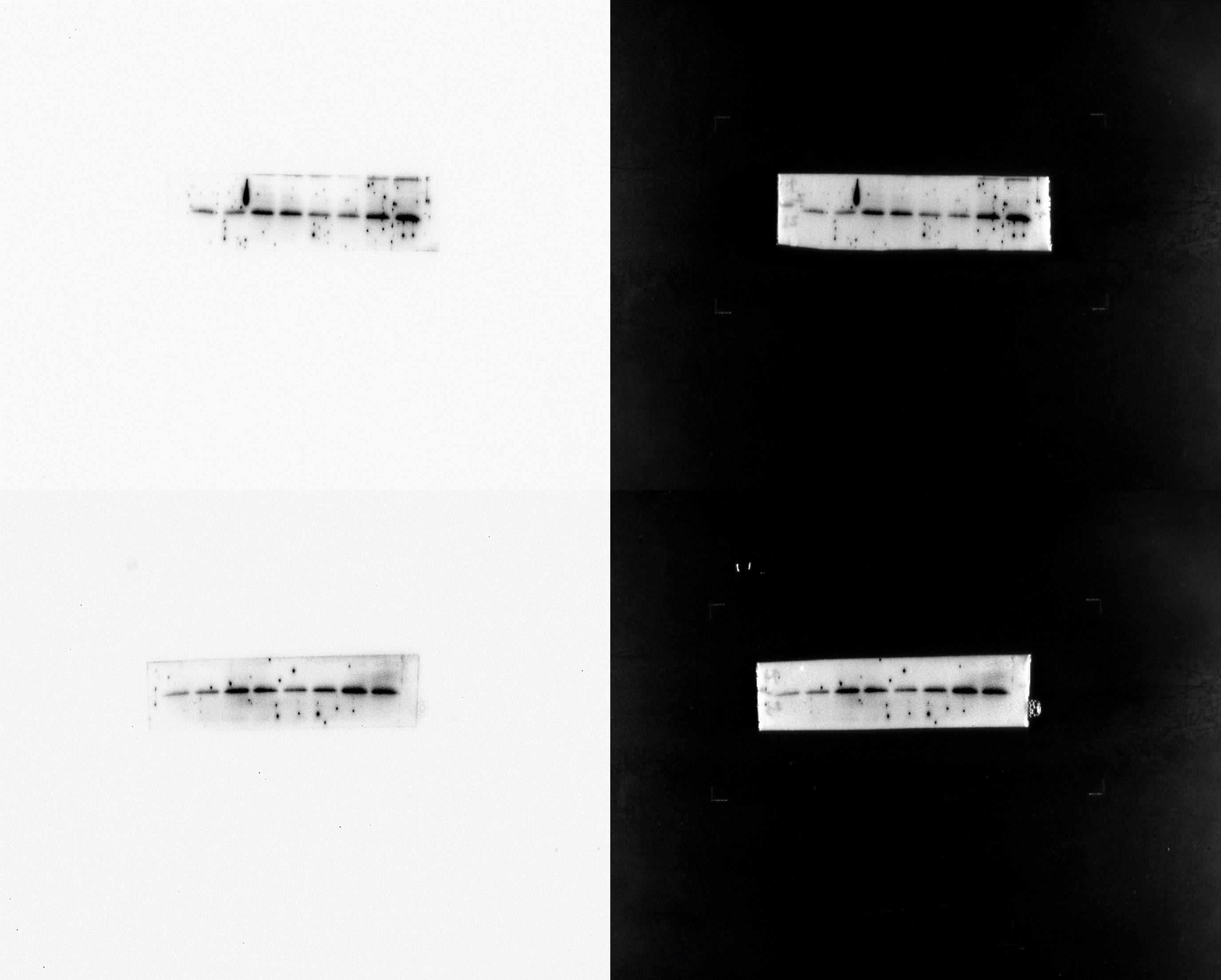

Supplement: Figure 3—source data 1. [file elife-96161-fig3-data1.zip › Figure 3-Source data1/Figure3C-Source data1-Claudin-5.png]

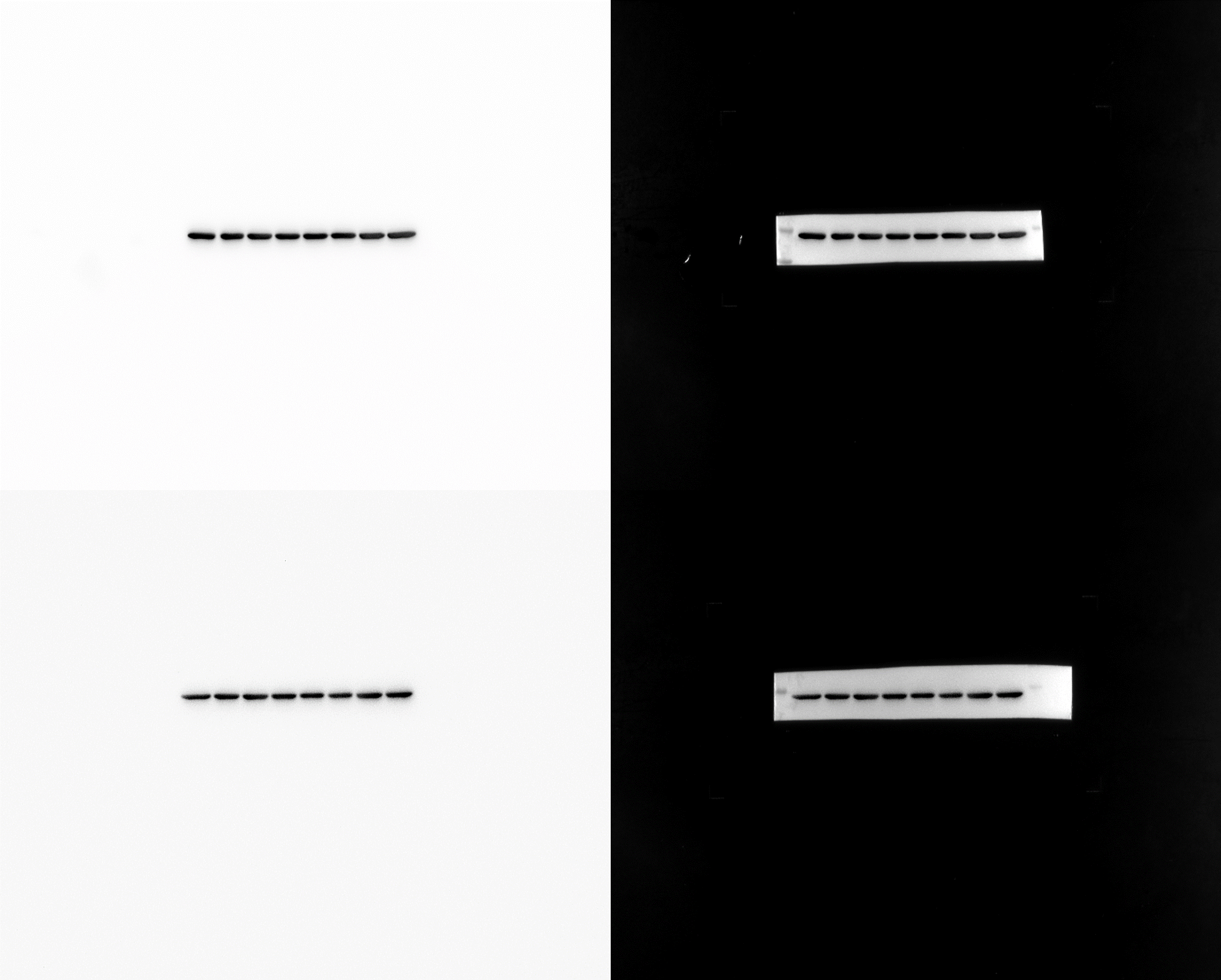

Supplement: Figure 3—source data 1. [file elife-96161-fig3-data1.zip › Figure 3-Source data1/Figure3C-Source data1-a┬-actin.png]

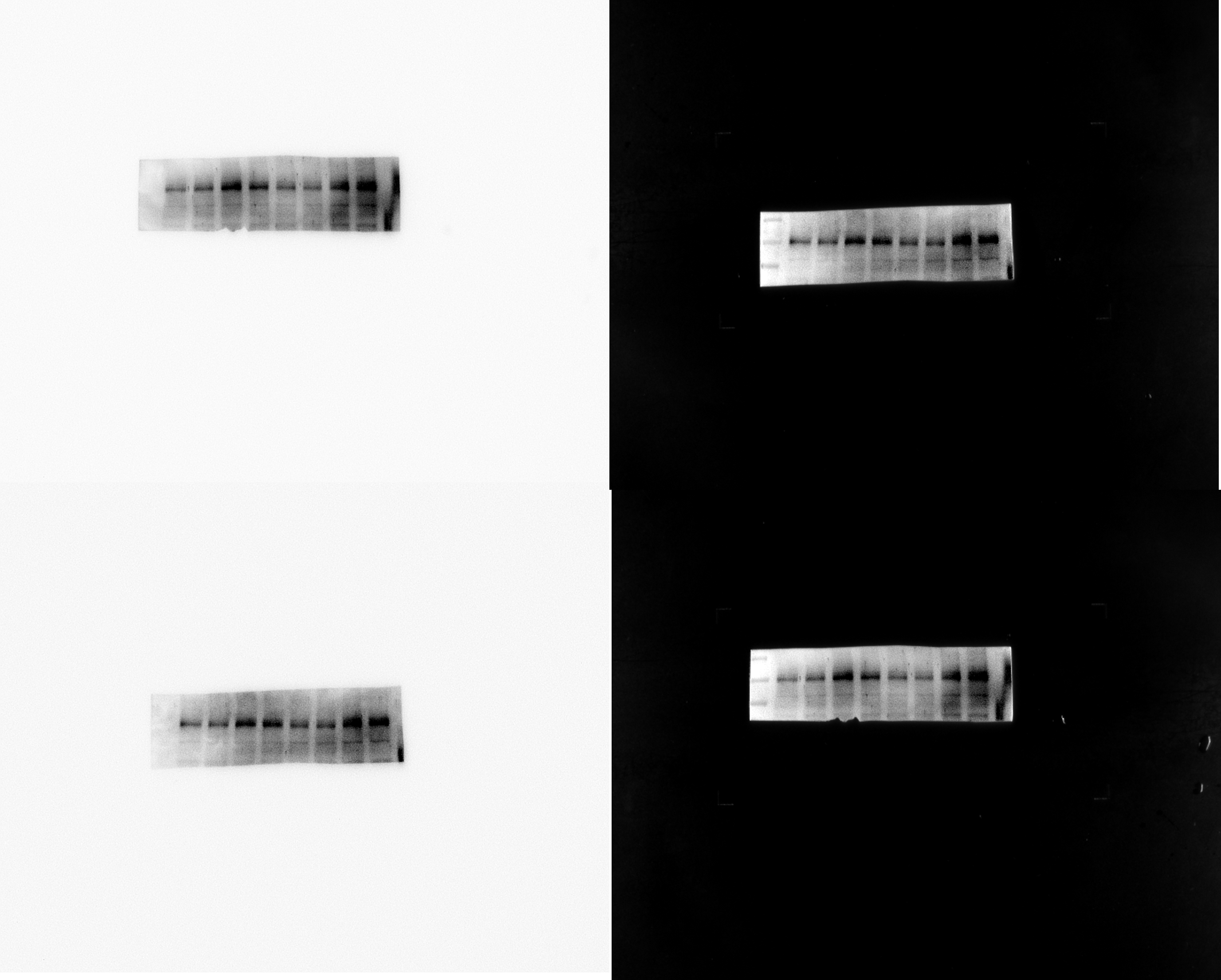

Supplement: Figure 3—source data 1. [file elife-96161-fig3-data1.zip › Figure 3-Source data1/Figure3C-Source data2-VE-Cadherin.png]

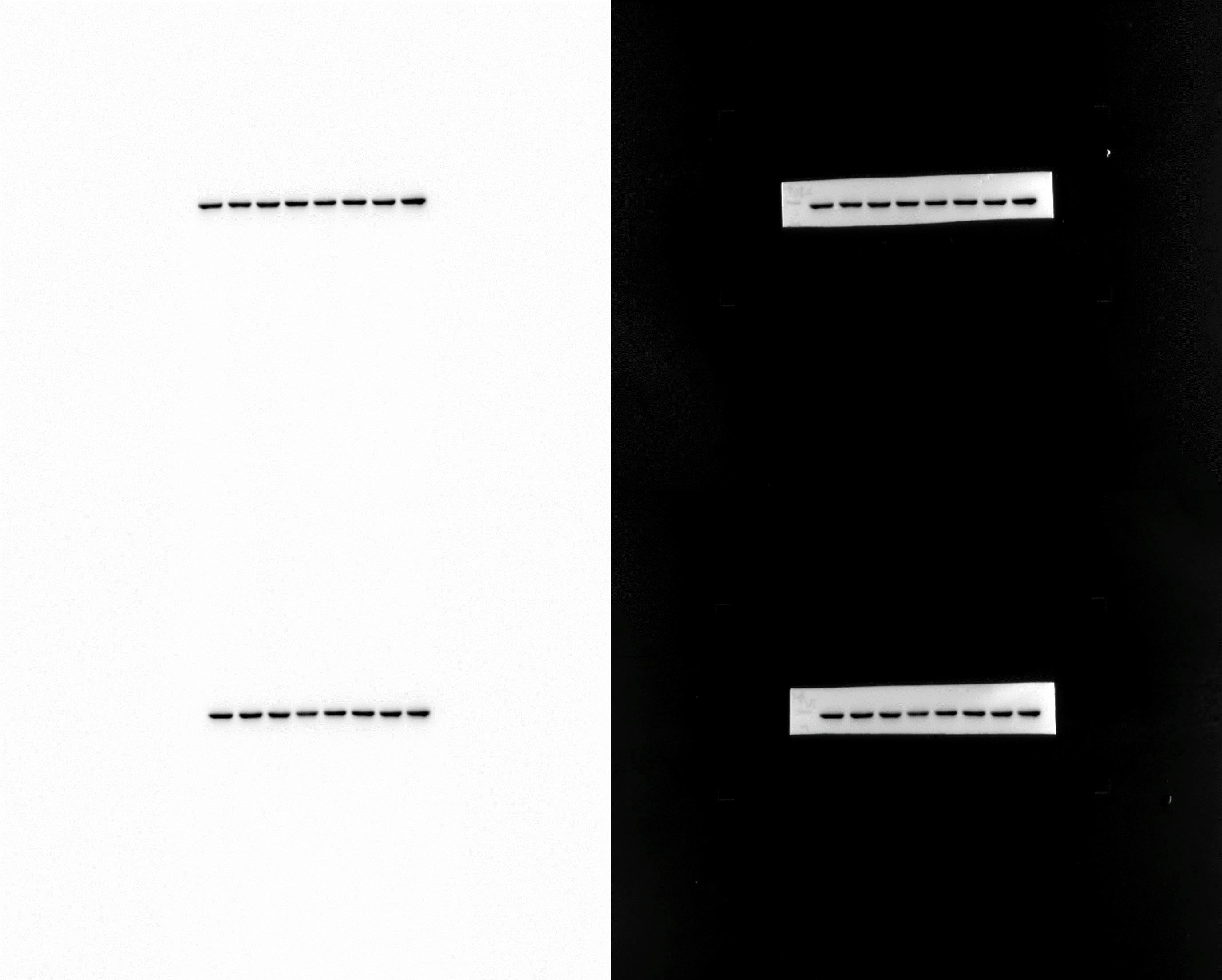

Supplement: Figure 3—source data 1. [file elife-96161-fig3-data1.zip › Figure 3-Source data1/Figure3C-Source data2-a┬-actin.png]

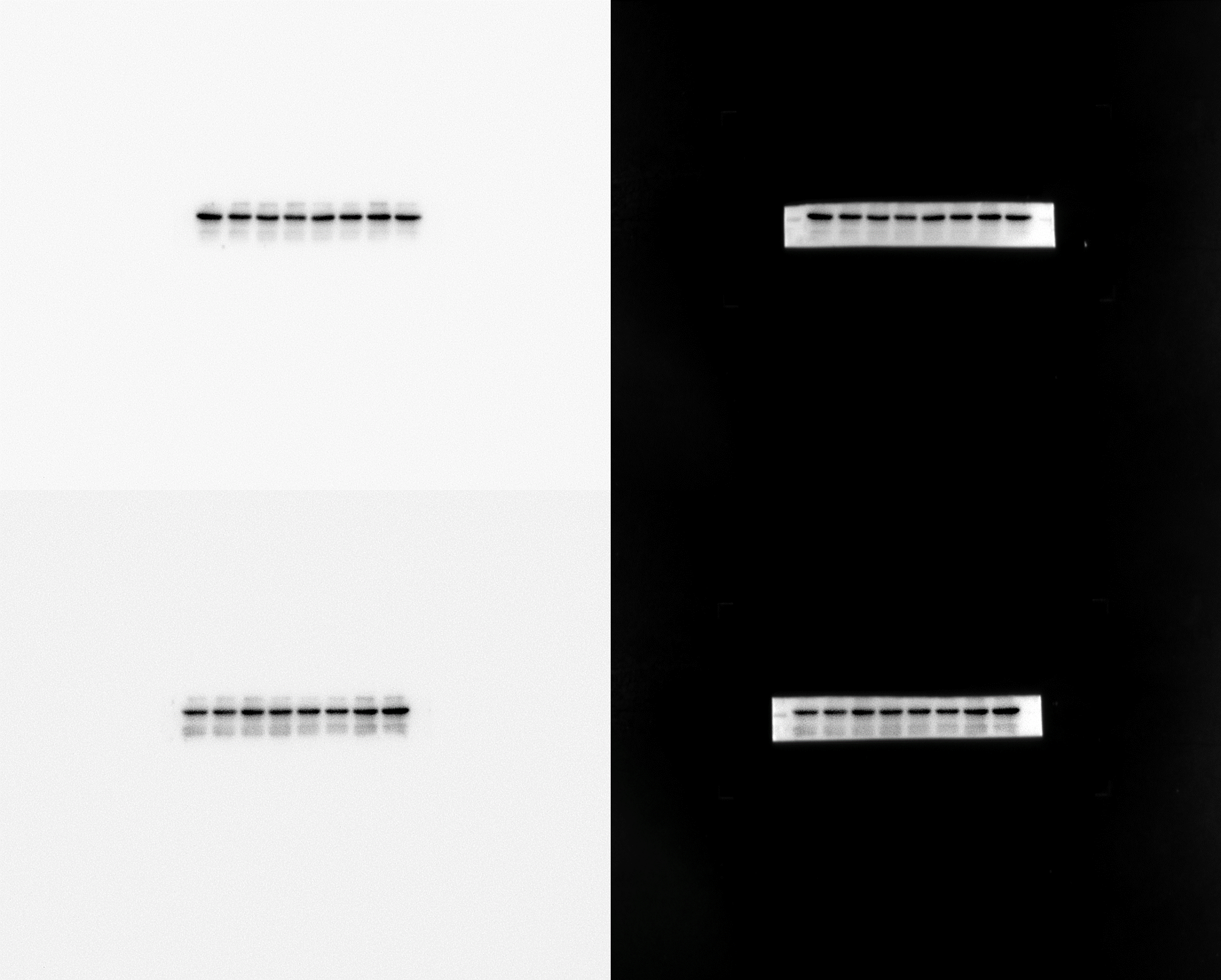

Supplement: Figure 3—source data 1. [file elife-96161-fig3-data1.zip › Figure 3-Source data1/Figure3C-Source data3-JNK.png]

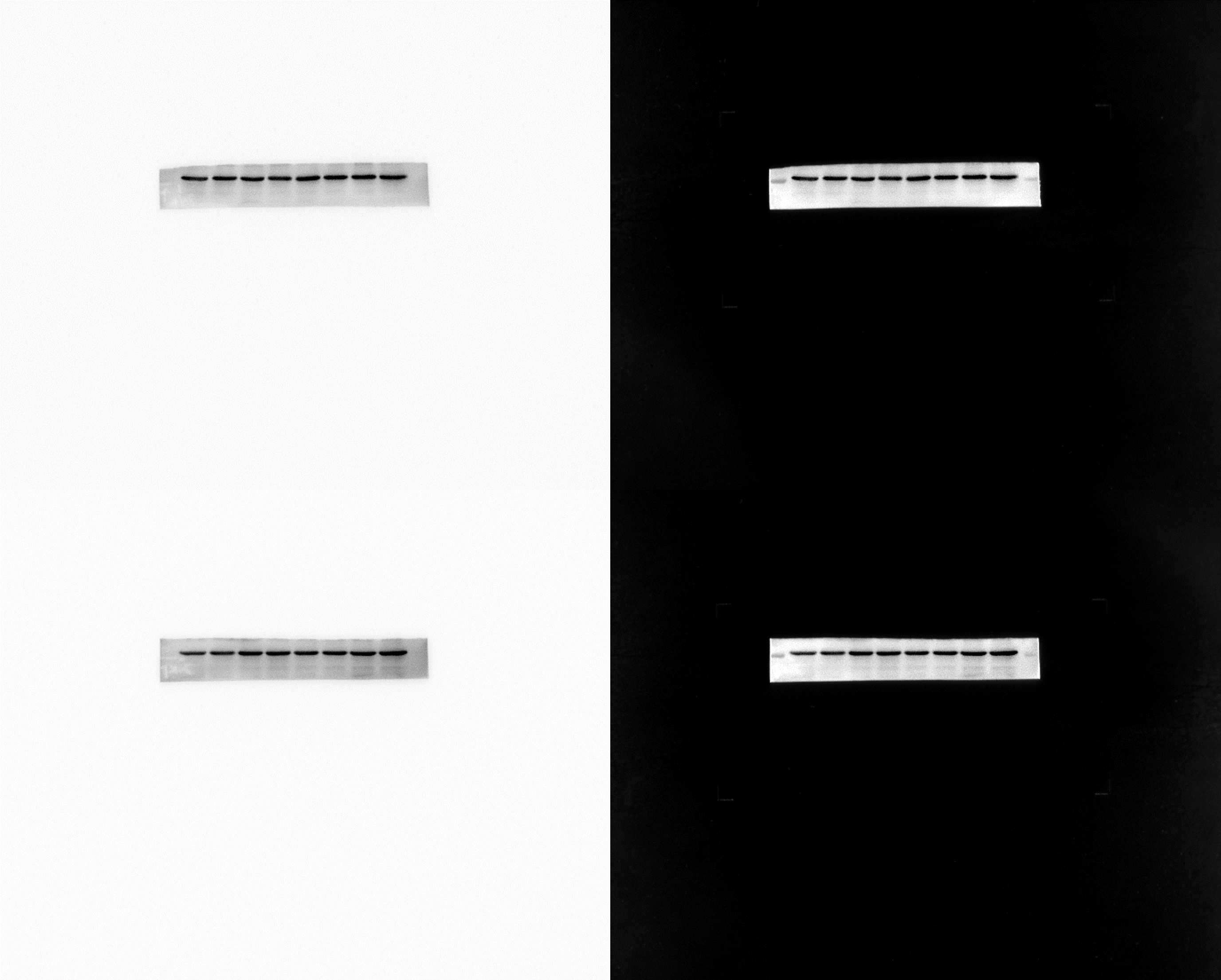

Supplement: Figure 3—source data 1. [file elife-96161-fig3-data1.zip › Figure 3-Source data1/Figure3C-Source data3-p-JNK.png]

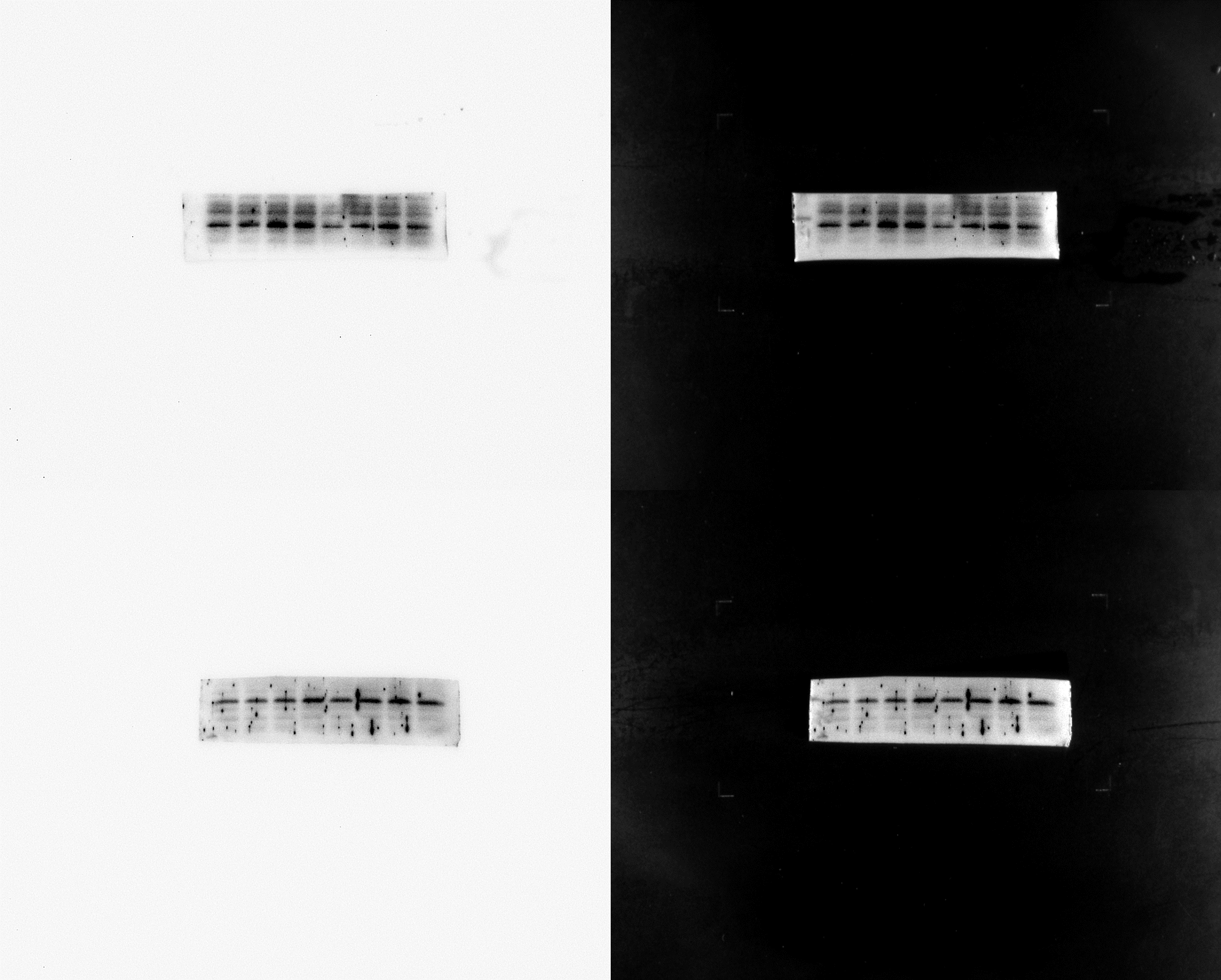

Supplement: Figure 3—source data 1. [file elife-96161-fig3-data1.zip › Figure 3-Source data1/Figure3D-Source data1-Claudin-5.png]

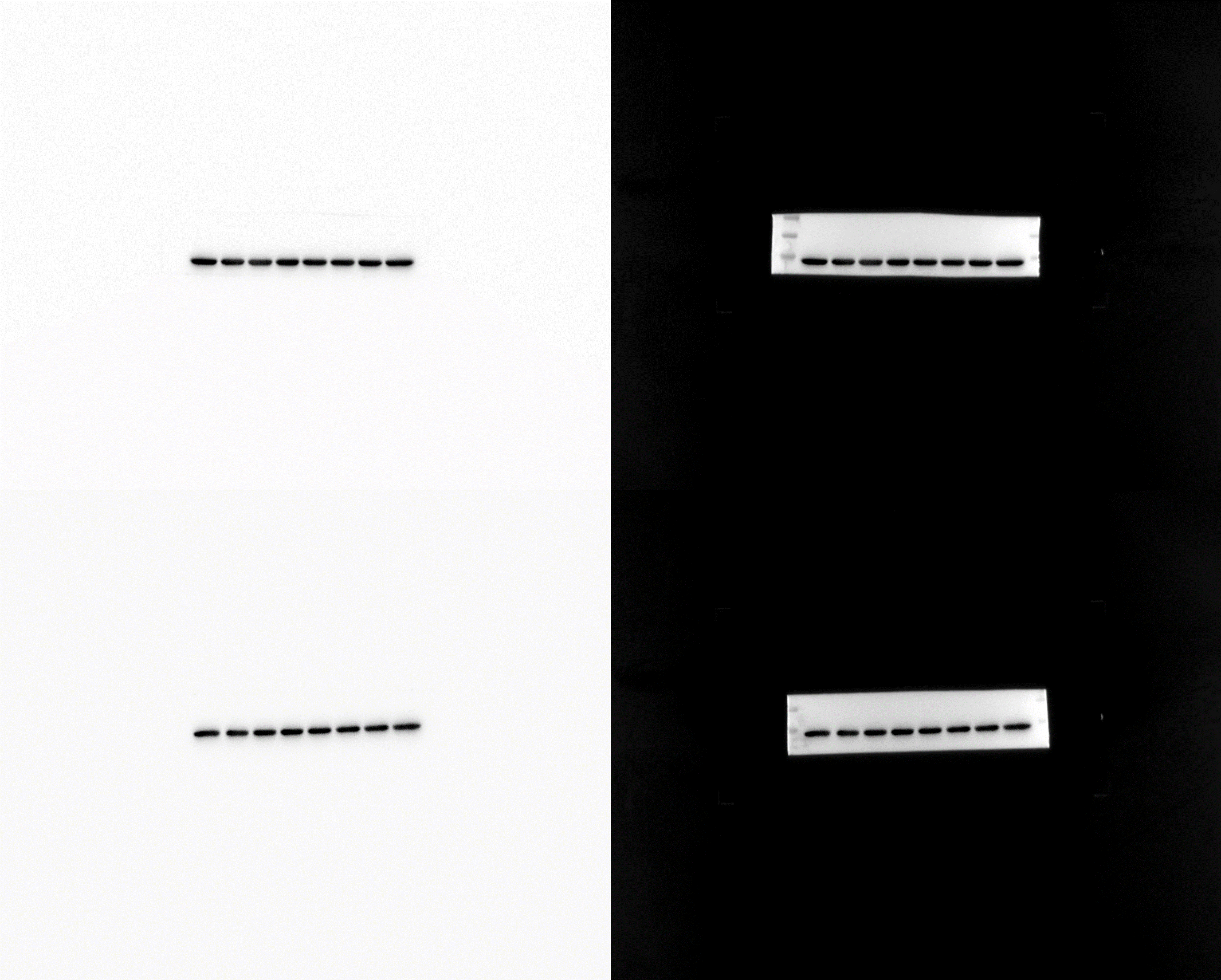

Supplement: Figure 3—source data 1. [file elife-96161-fig3-data1.zip › Figure 3-Source data1/Figure3D-Source data1-a┬-actin.png]

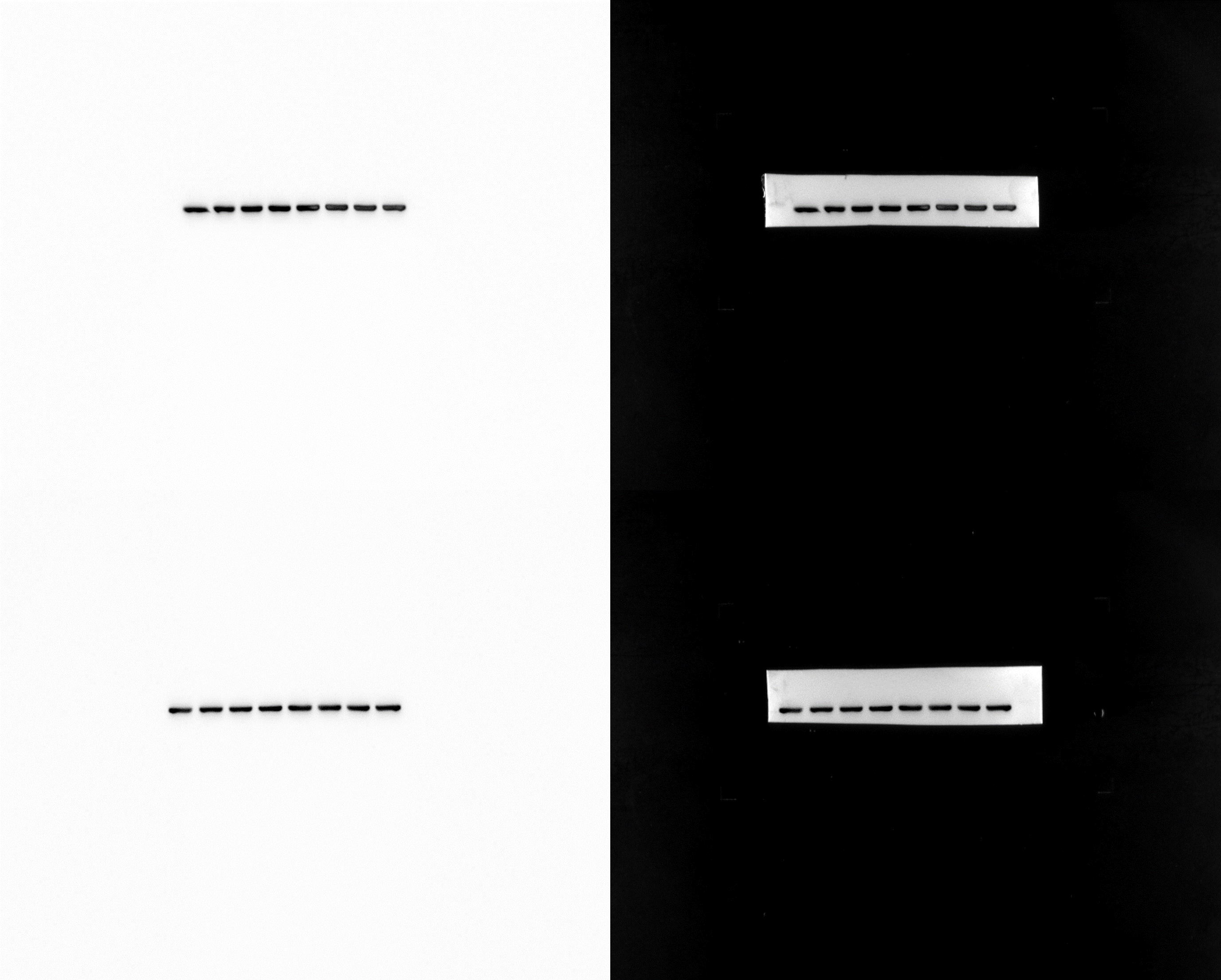

Supplement: Figure 3—source data 1. [file elife-96161-fig3-data1.zip › Figure 3-Source data1/Figure3D-Source data2-Tubulin.png]

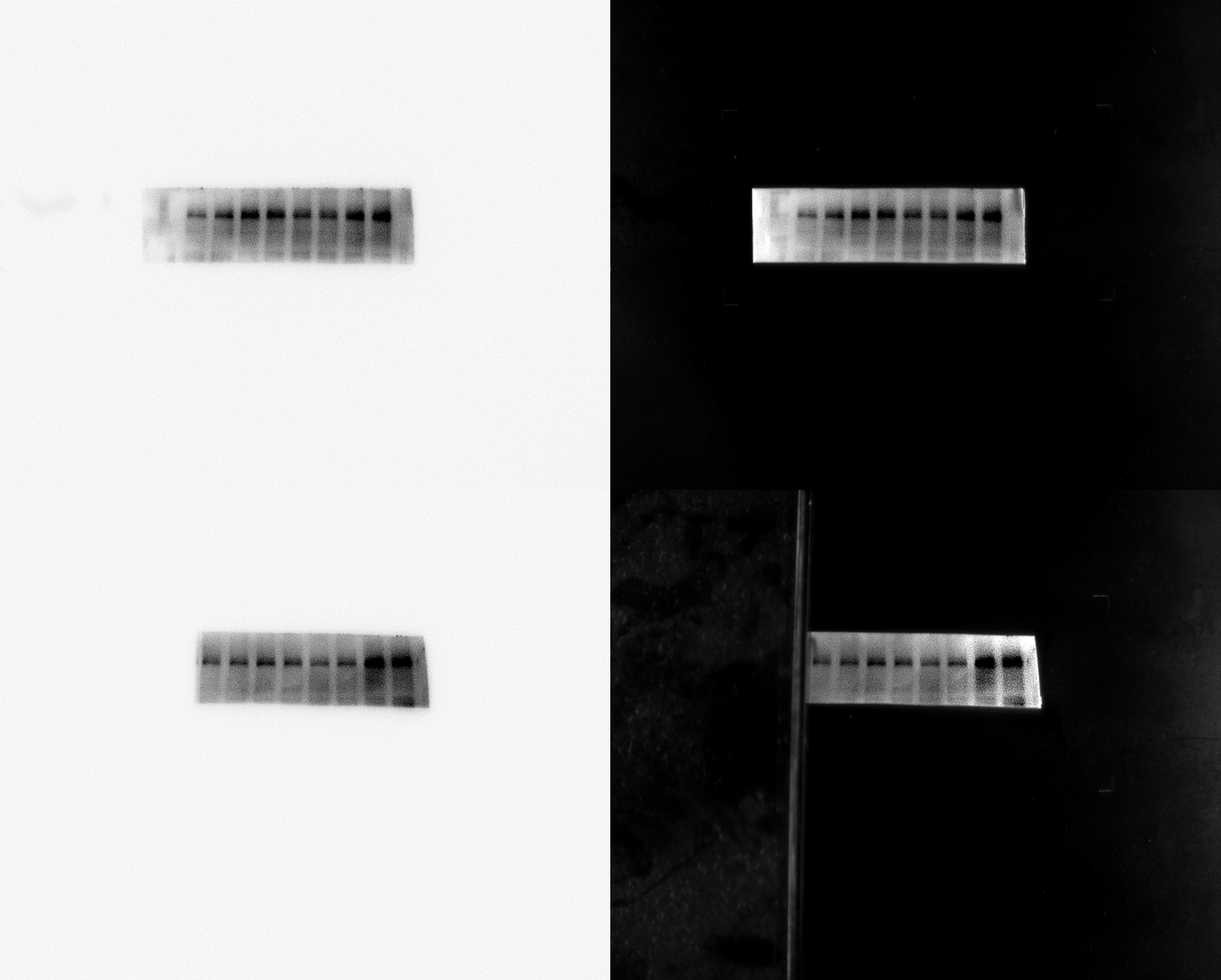

Supplement: Figure 3—source data 1. [file elife-96161-fig3-data1.zip › Figure 3-Source data1/Figure3D-Source data2-VE-Cadherin.png]

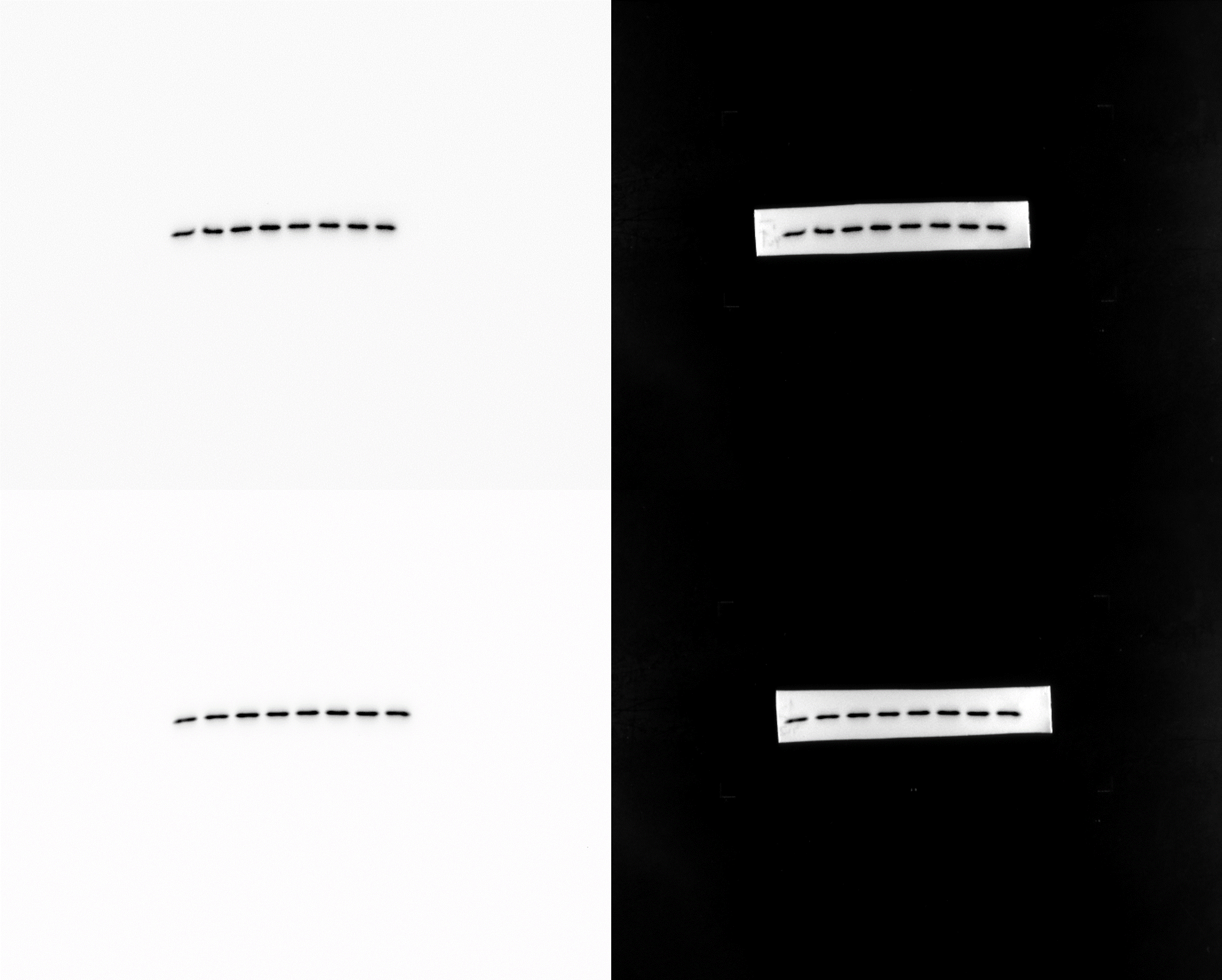

Supplement: Figure 3—source data 1. [file elife-96161-fig3-data1.zip › Figure 3-Source data1/Figure3D-Source data3-p38.png]

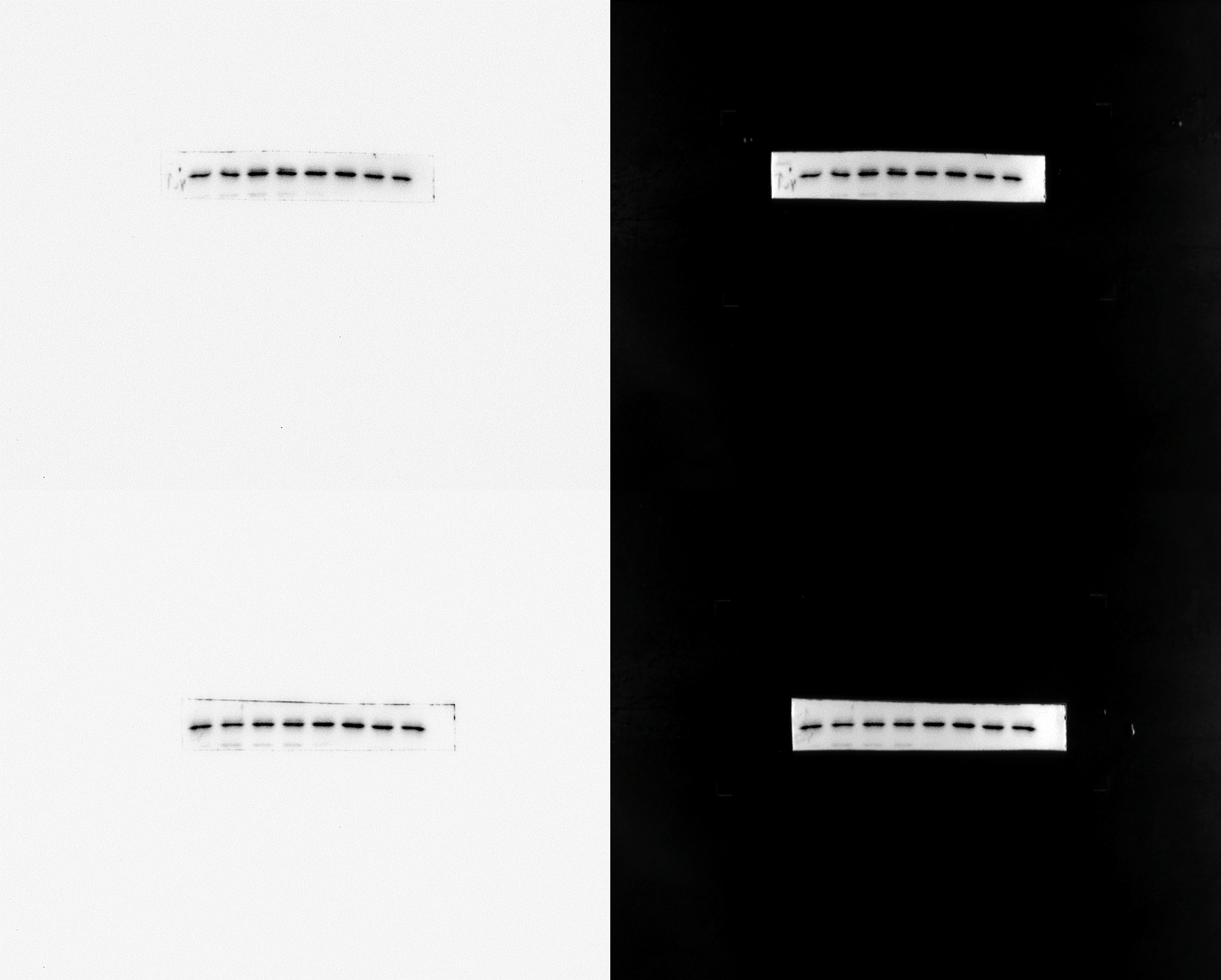

Supplement: Figure 3—source data 1. [file elife-96161-fig3-data1.zip › Figure 3-Source data1/Figure3D-Source data3-p-p38.png]

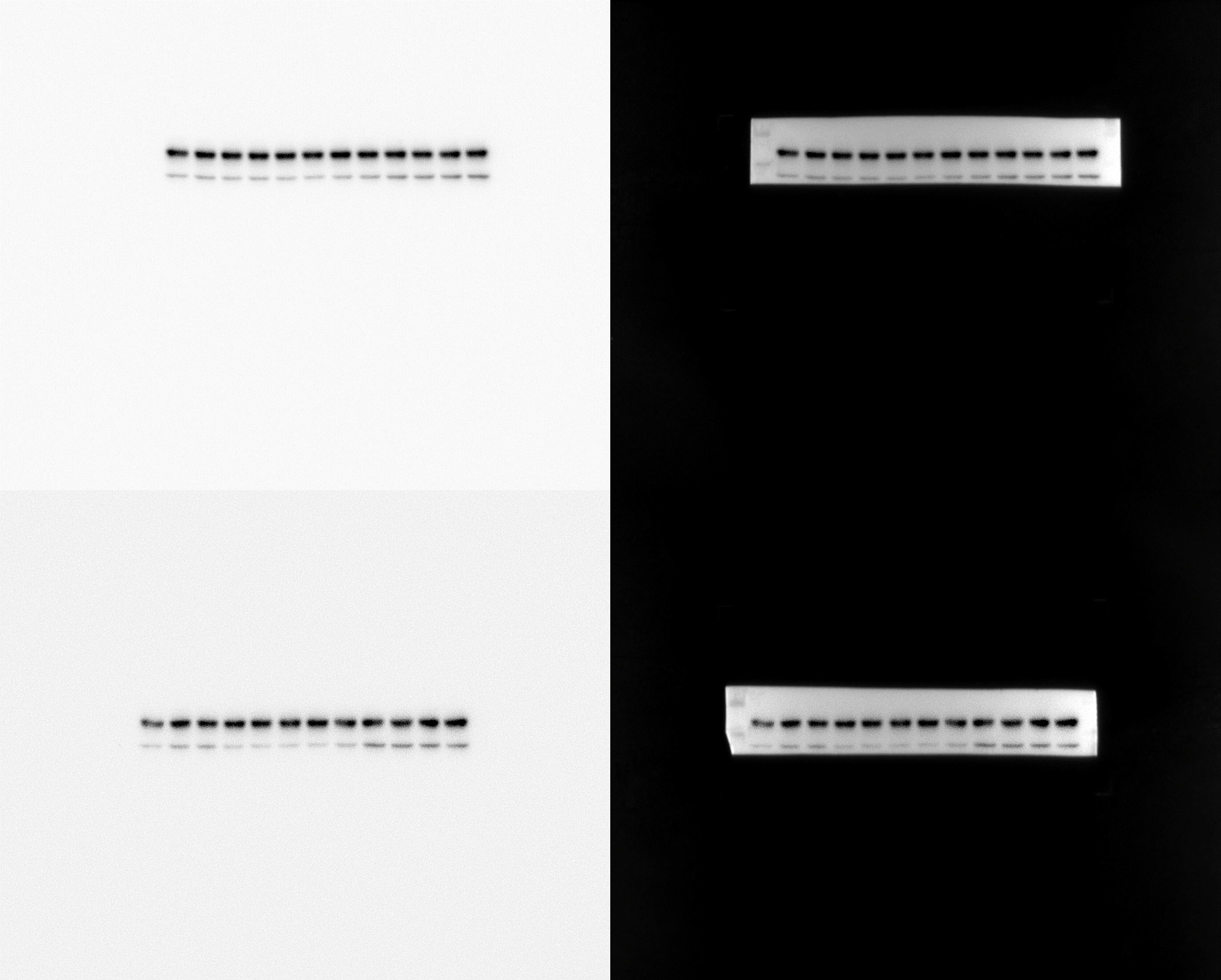

Supplement: Figure 3—source data 1. [file elife-96161-fig3-data1.zip › Figure 3-Source data1/Figure3E-Source data1-AKT.png]

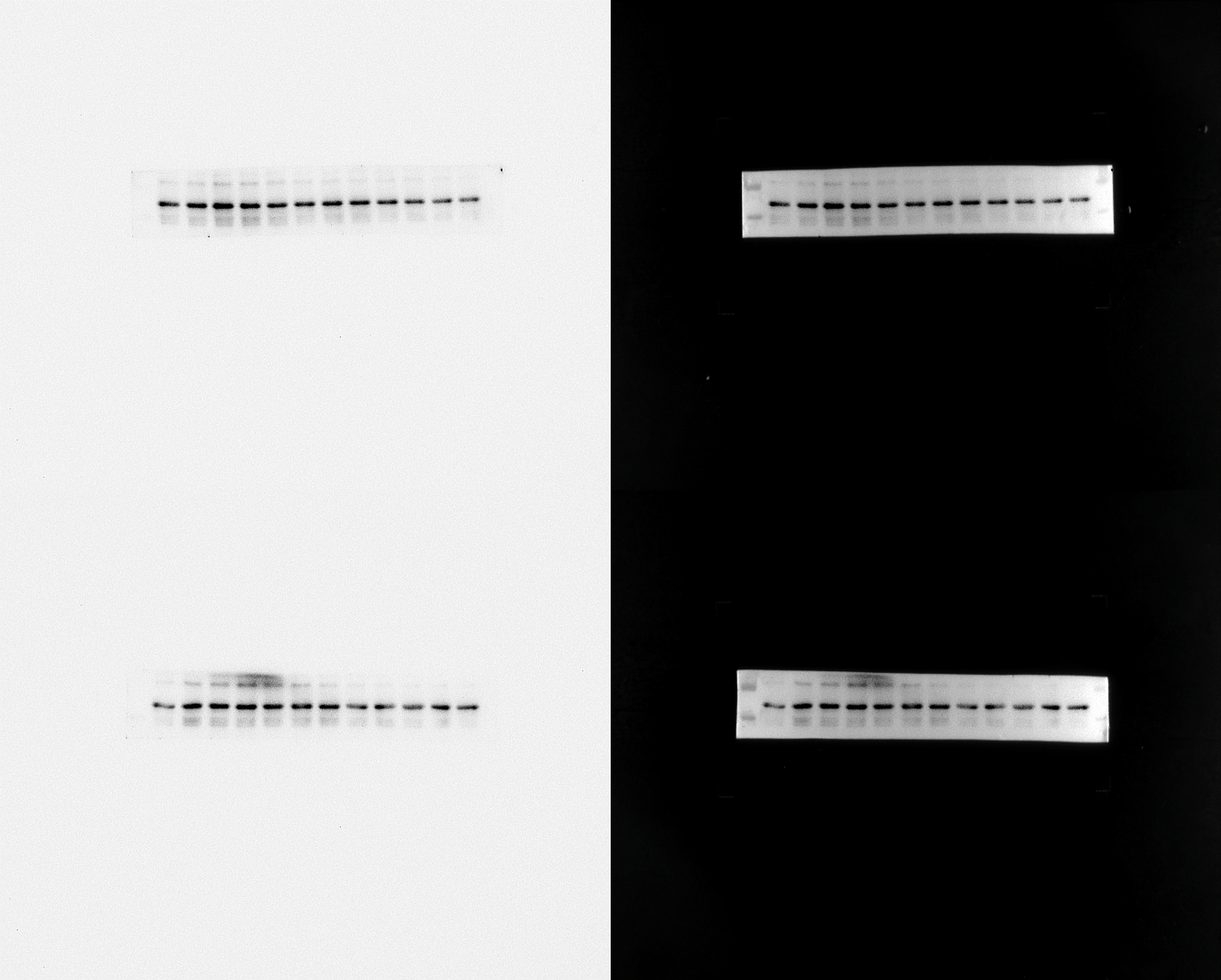

Supplement: Figure 3—source data 1. [file elife-96161-fig3-data1.zip › Figure 3-Source data1/Figure3E-Source data1-p-AKT.png]

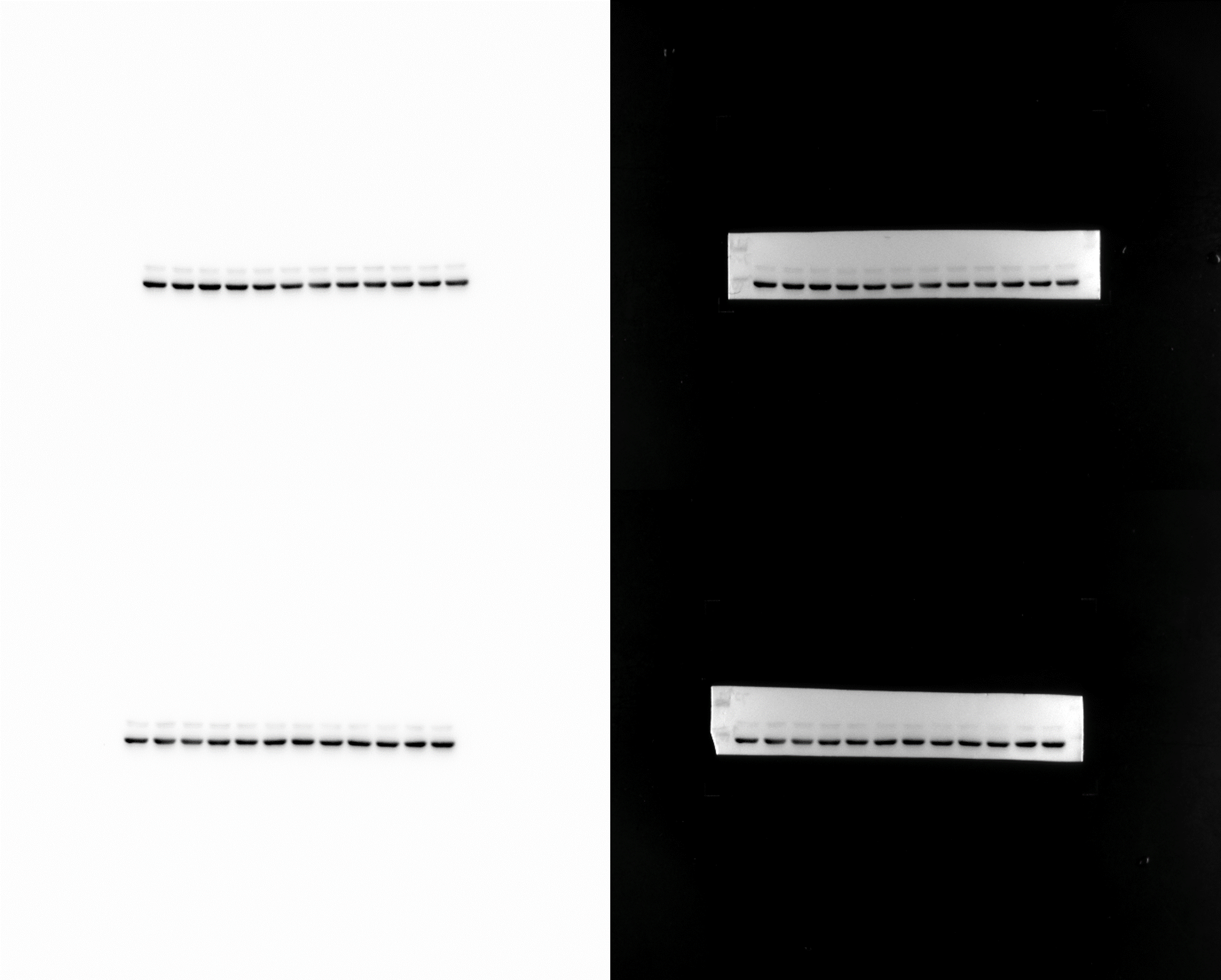

Supplement: Figure 3—source data 1. [file elife-96161-fig3-data1.zip › Figure 3-Source data1/Figure3E-Source data1-Tubulin.png]

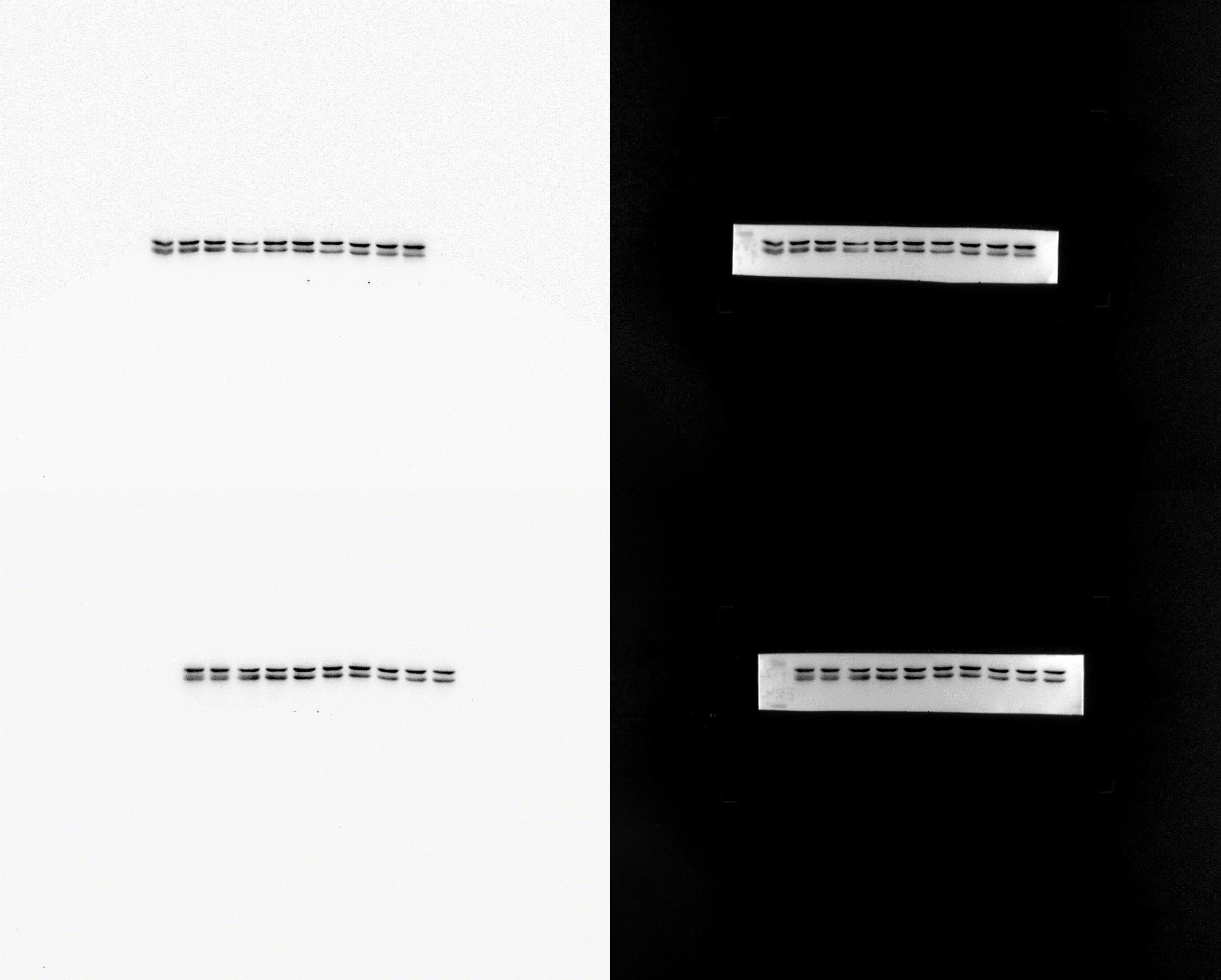

Supplement: Figure 3—source data 1. [file elife-96161-fig3-data1.zip › Figure 3-Source data1/Figure3E-Source data2-ERK.png]

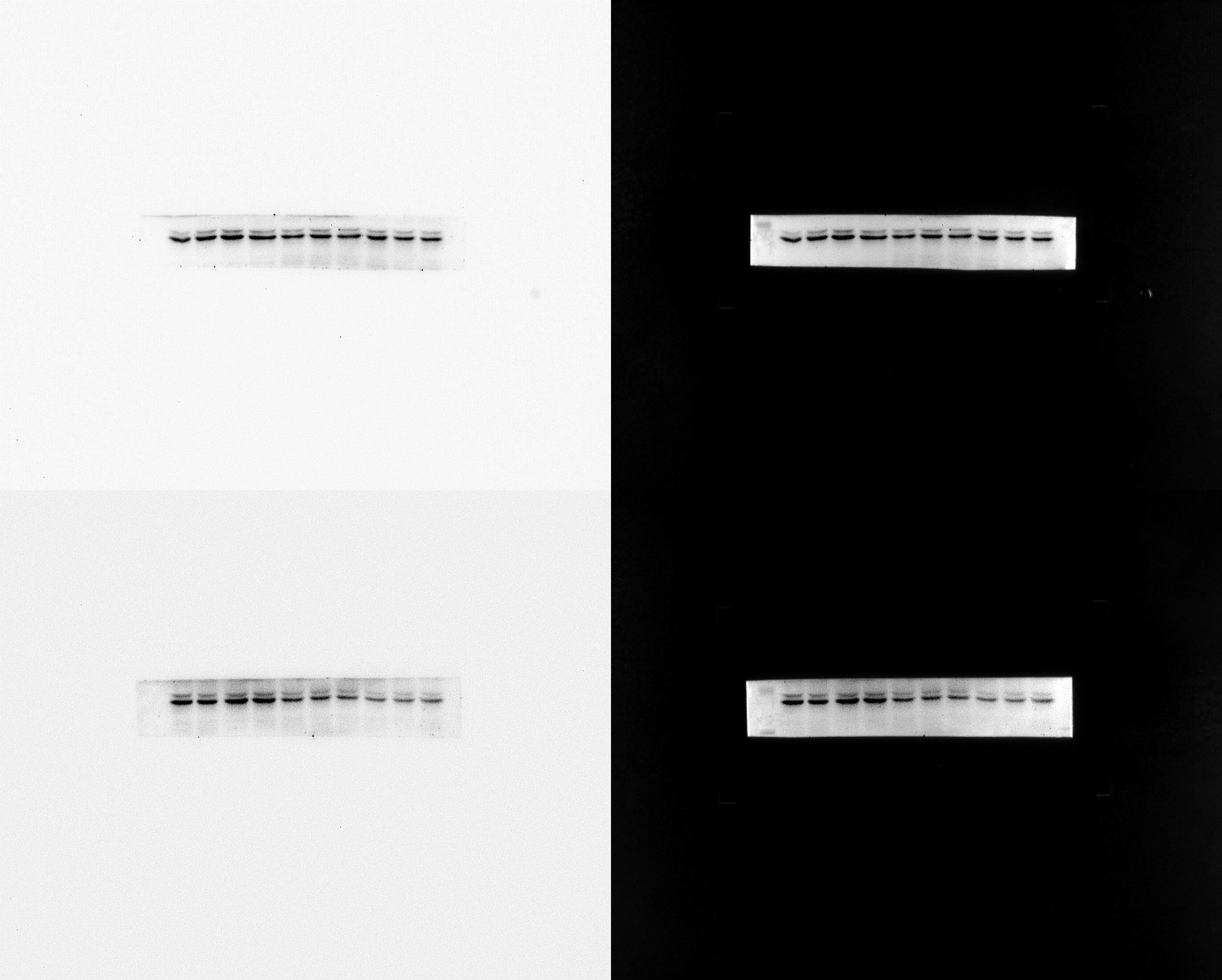

Supplement: Figure 3—source data 1. [file elife-96161-fig3-data1.zip › Figure 3-Source data1/Figure3E-Source data2-p-ERK.png]

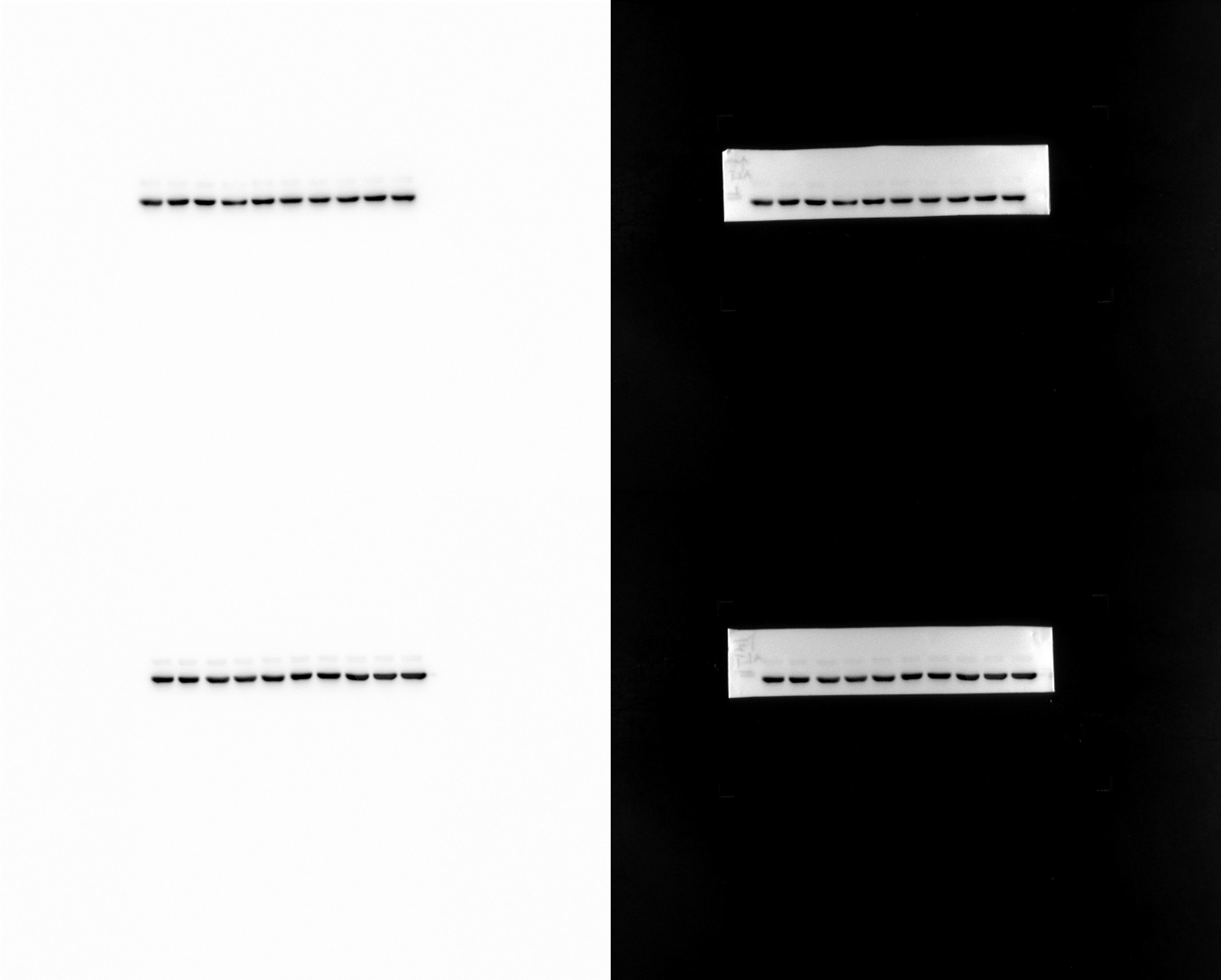

Supplement: Figure 3—source data 1. [file elife-96161-fig3-data1.zip › Figure 3-Source data1/Figure3E-Source data2-Tubulin.png]

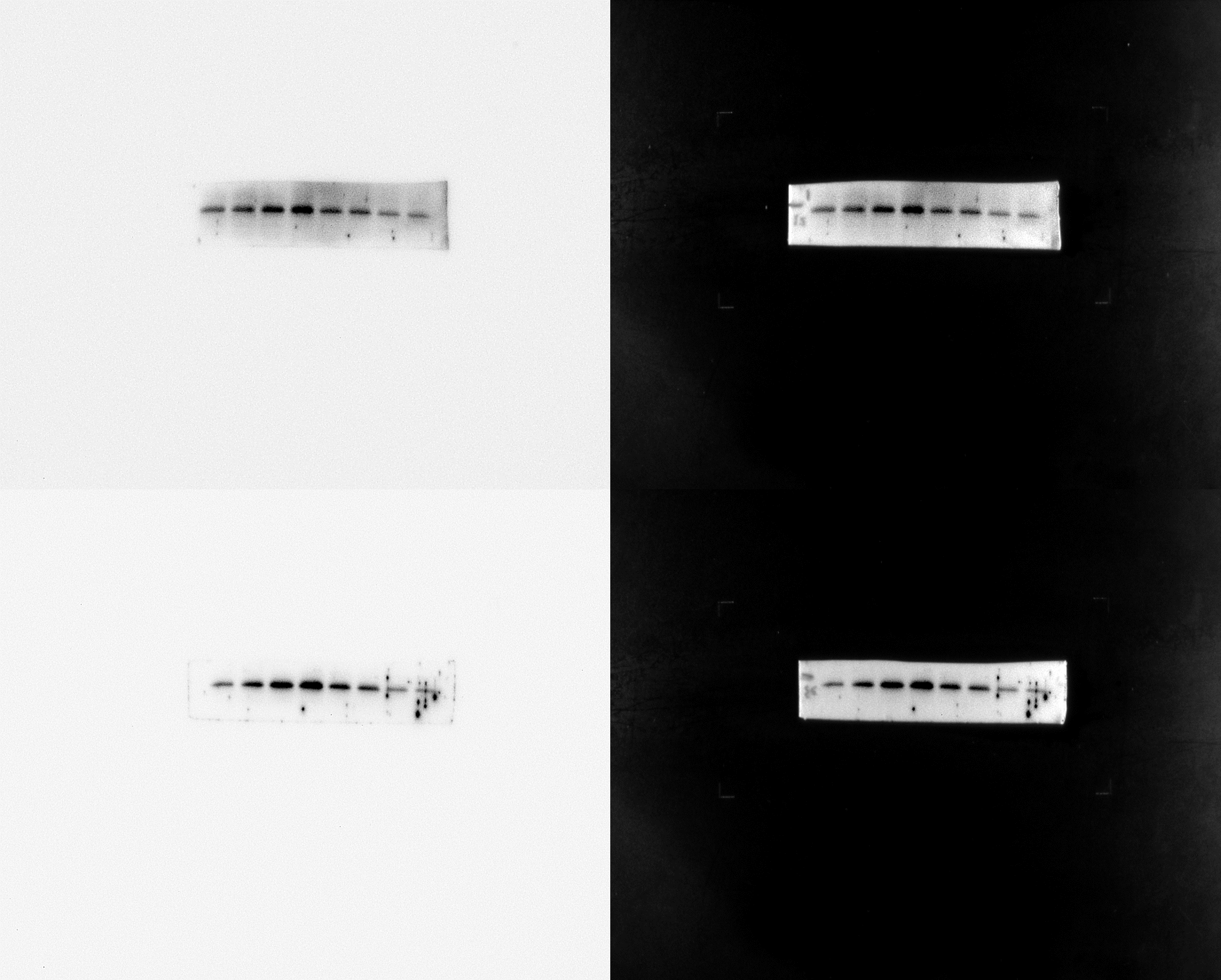

Supplement: Figure 3—source data 1. [file elife-96161-fig3-data1.zip › Figure 3-Source data1/Figure3F-Source data1-Claudin-5.png]

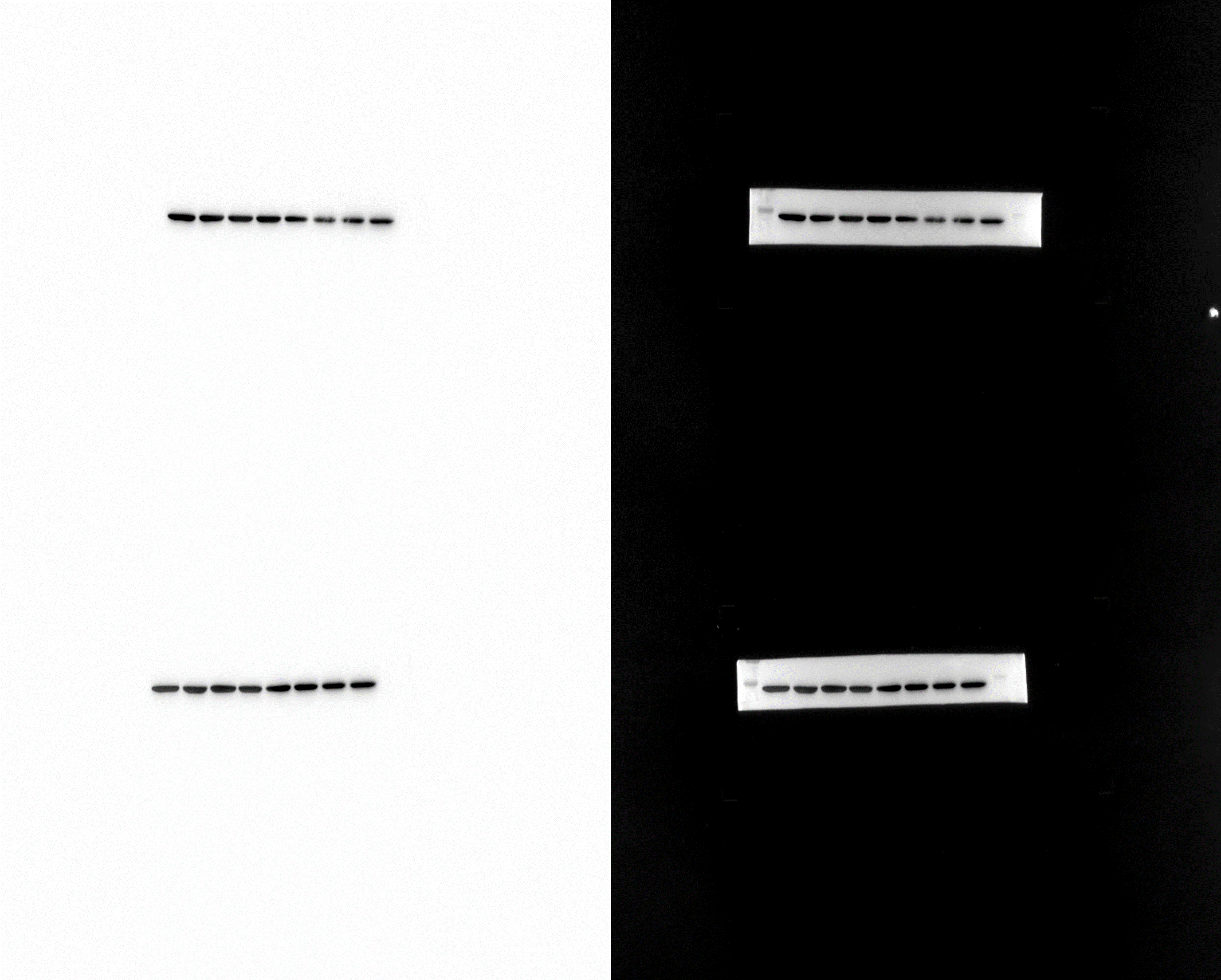

Supplement: Figure 3—source data 1. [file elife-96161-fig3-data1.zip › Figure 3-Source data1/Figure3F-Source data1-a┬-actin.png]

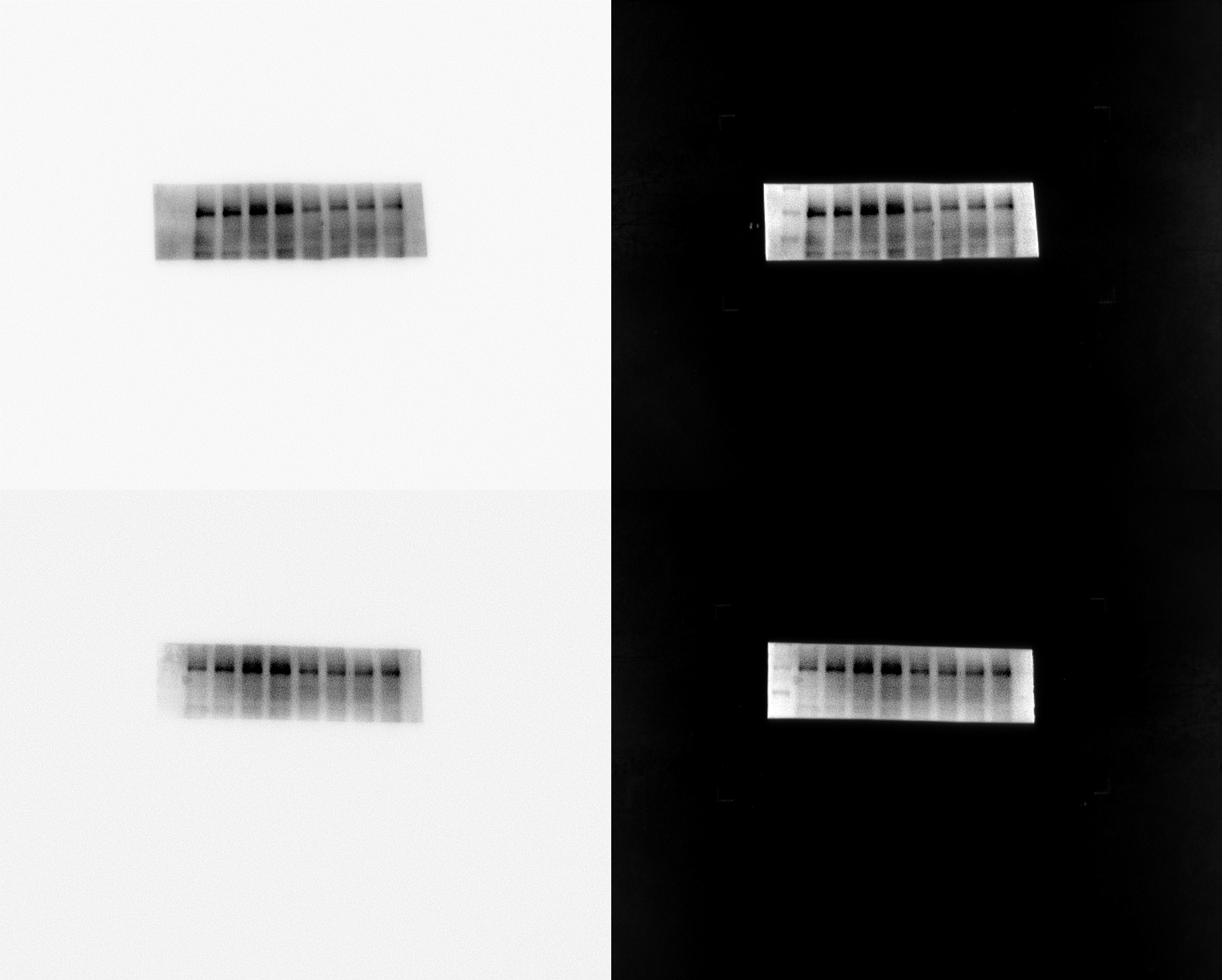

Supplement: Figure 3—source data 1. [file elife-96161-fig3-data1.zip › Figure 3-Source data1/Figure3F-Source data2-VE-Cadherin.png]

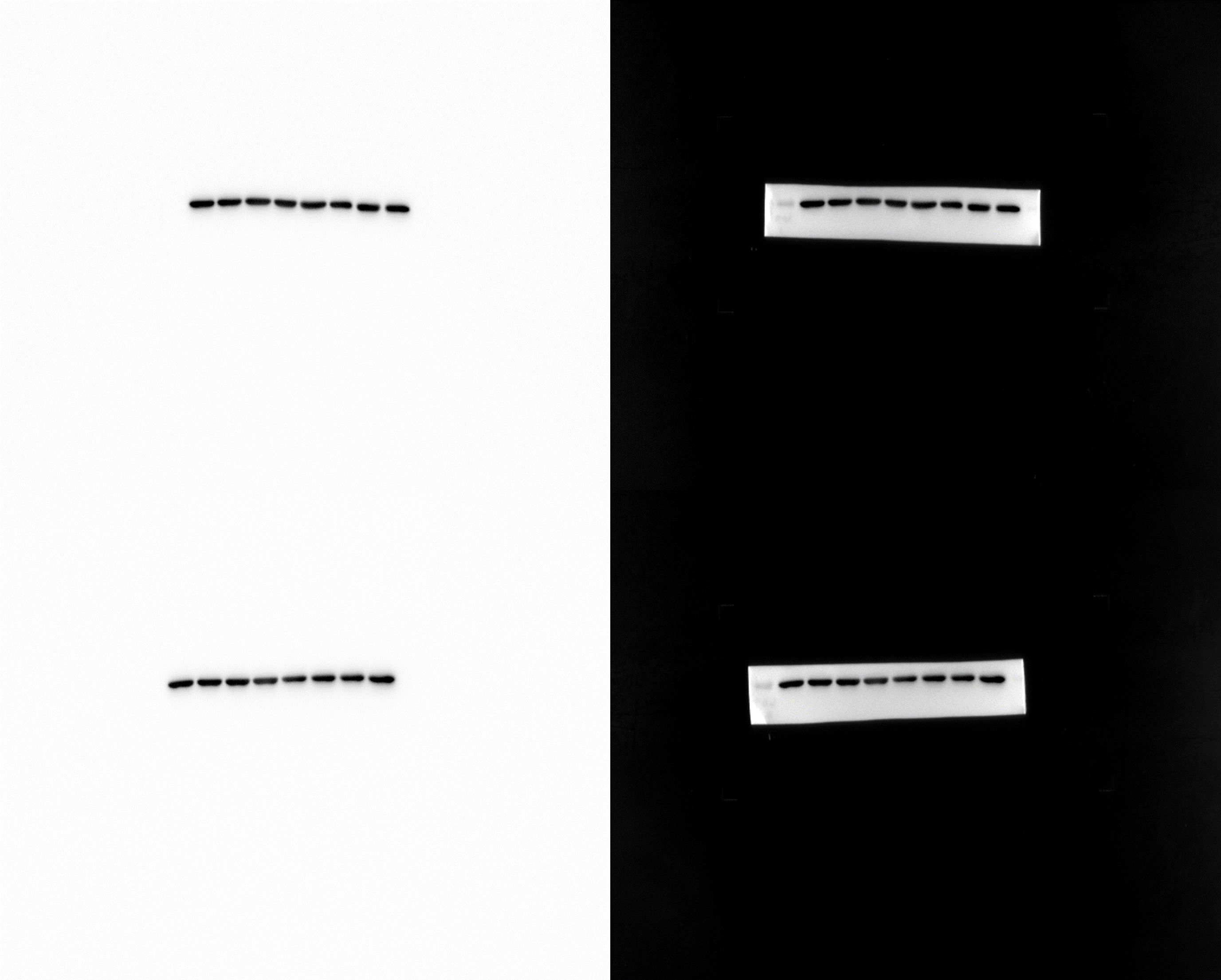

Supplement: Figure 3—source data 1. [file elife-96161-fig3-data1.zip › Figure 3-Source data1/Figure3F-Source data2-a┬-actin.png]

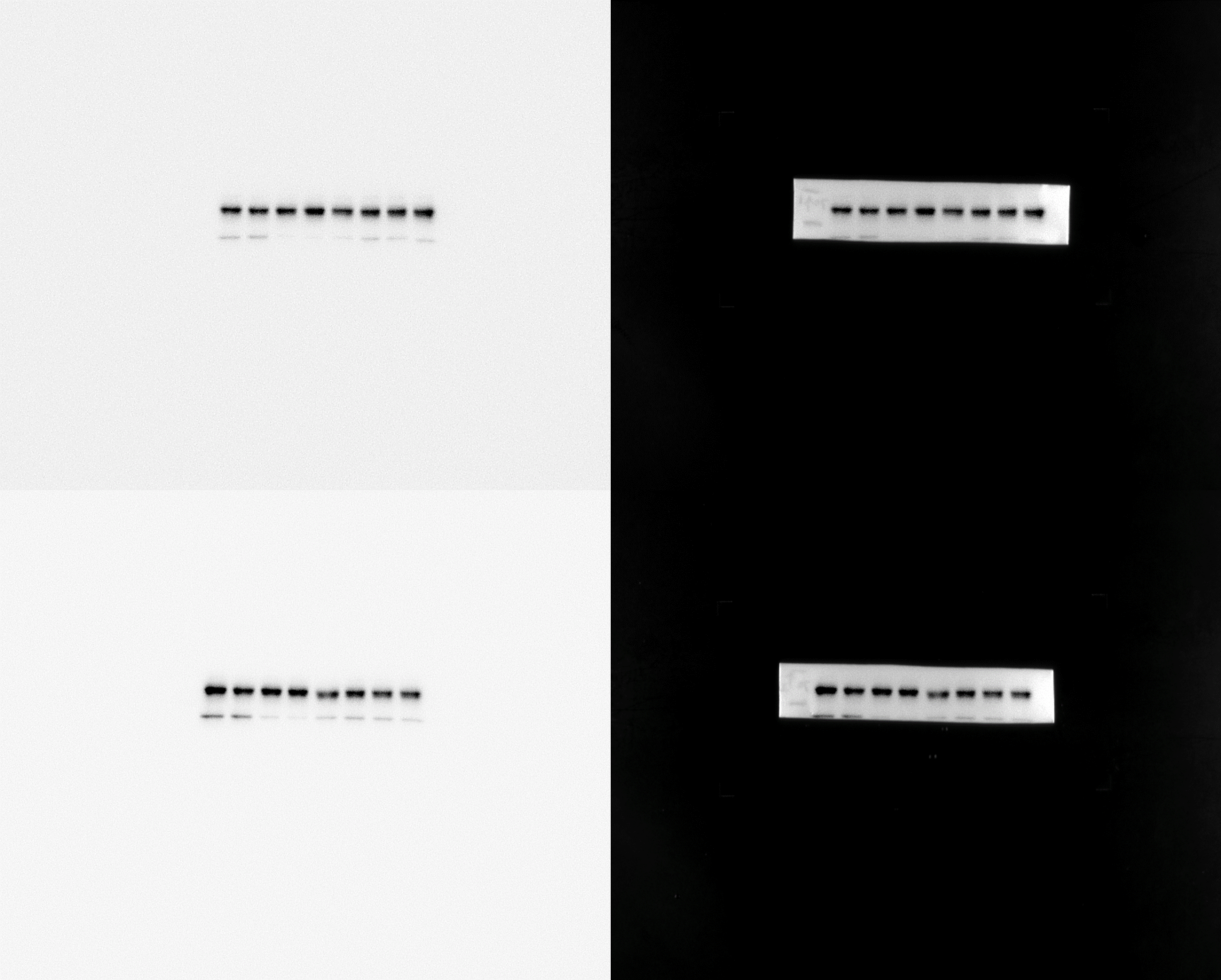

Supplement: Figure 3—source data 1. [file elife-96161-fig3-data1.zip › Figure 3-Source data1/Figure3F-Source data3-AKT.png]

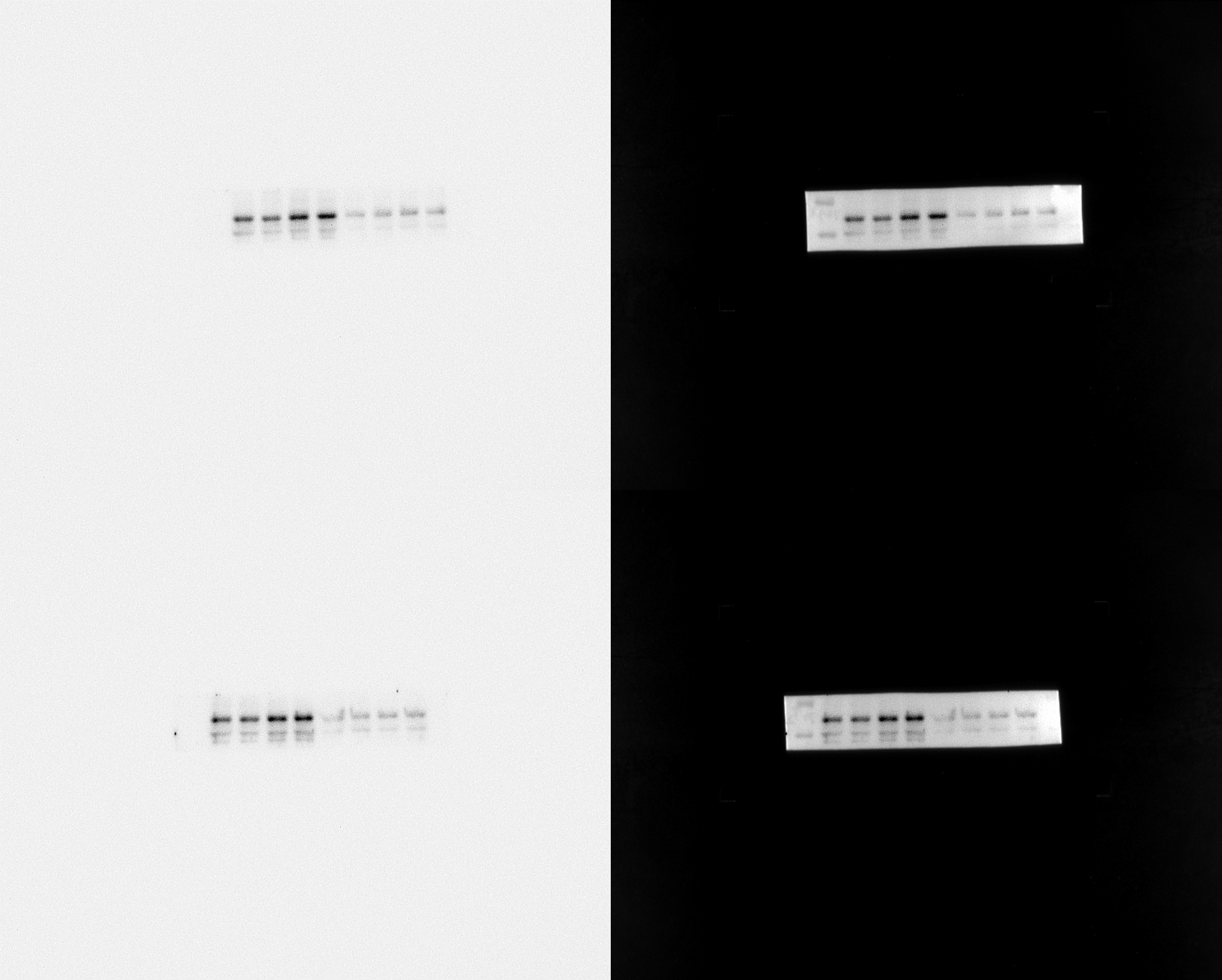

Supplement: Figure 3—source data 1. [file elife-96161-fig3-data1.zip › Figure 3-Source data1/Figure3F-Source data3-p-AKT.png]

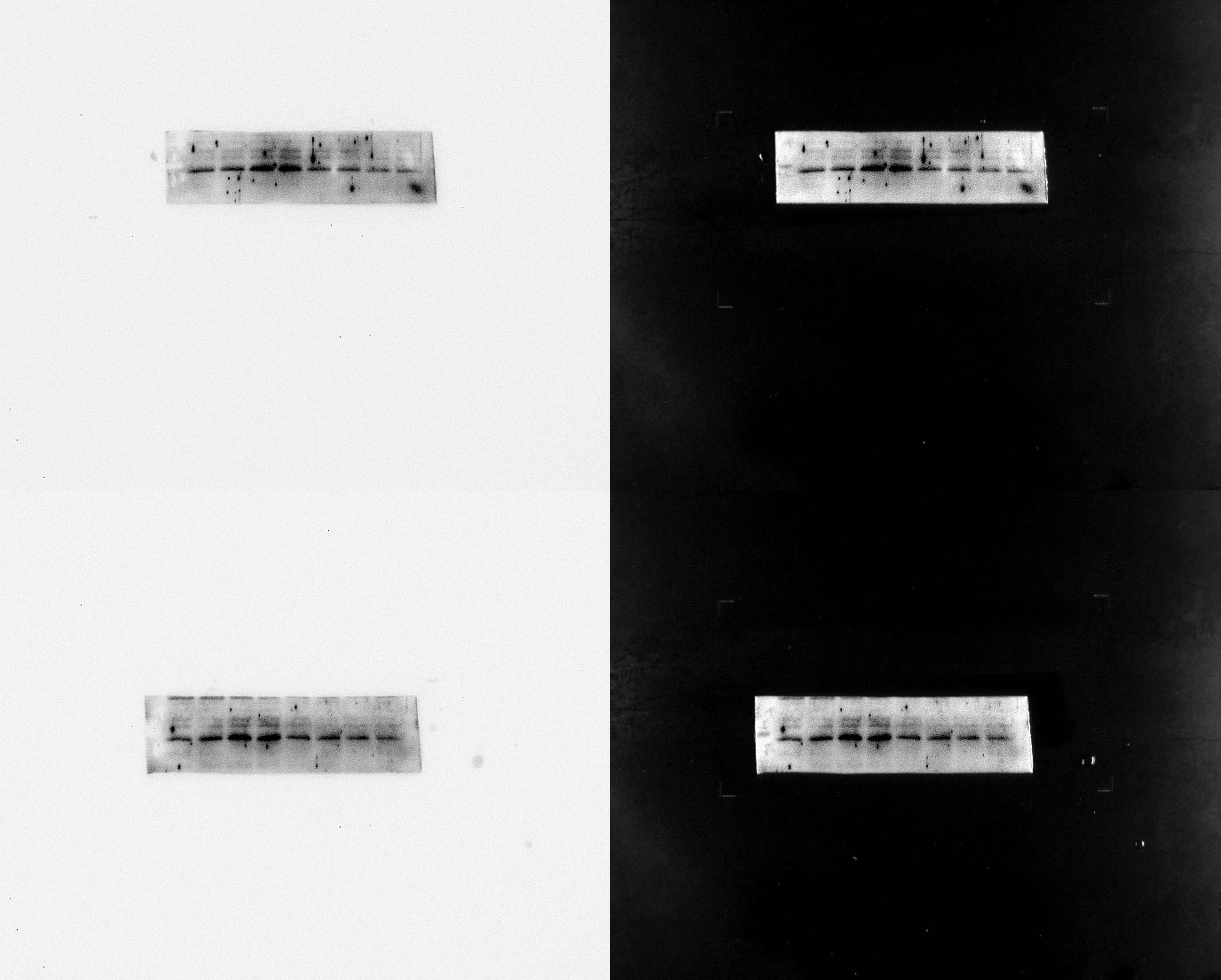

Supplement: Figure 3—source data 1. [file elife-96161-fig3-data1.zip › Figure 3-Source data1/Figure3G-Source data1-Claudin-5.png]

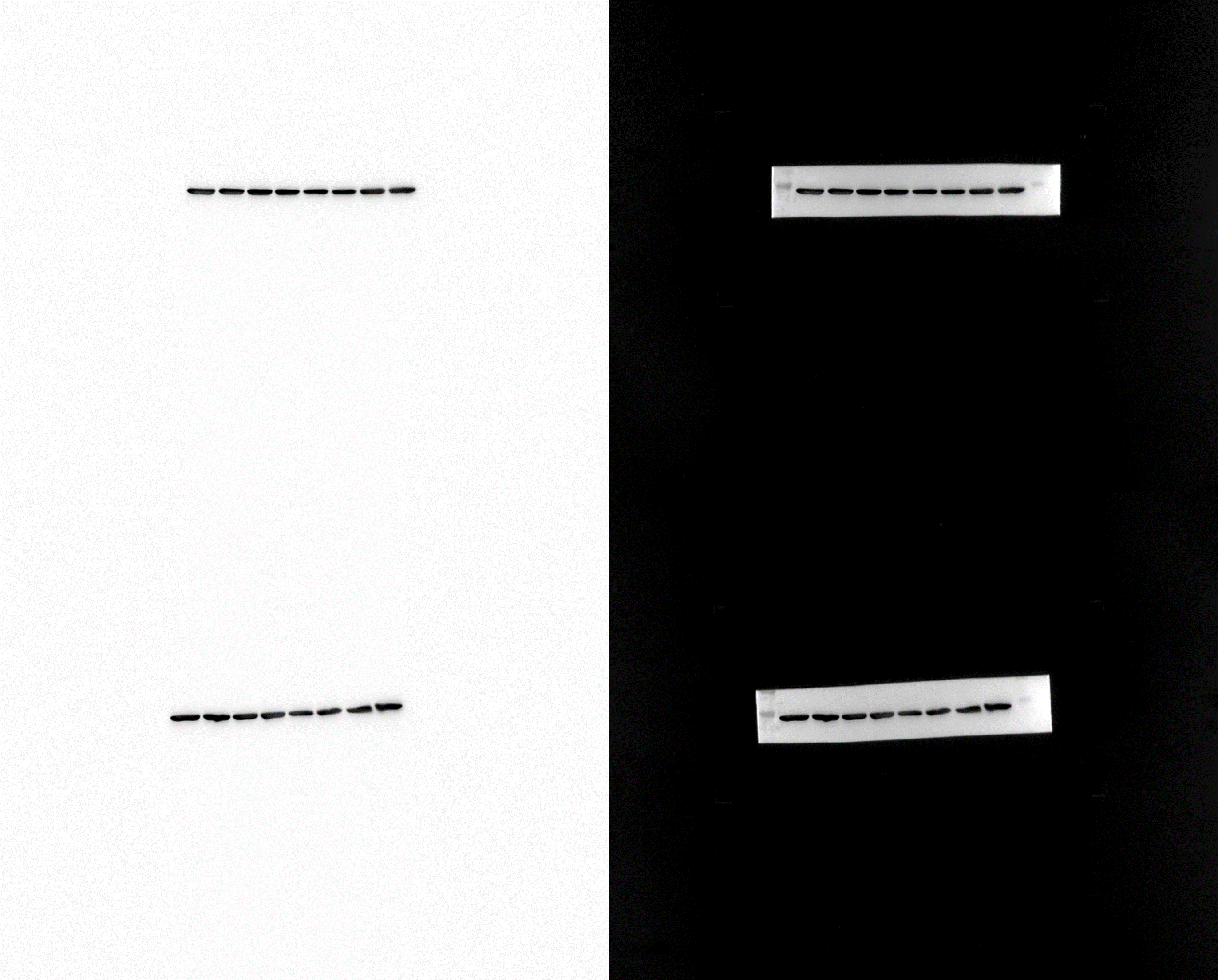

Supplement: Figure 3—source data 1. [file elife-96161-fig3-data1.zip › Figure 3-Source data1/Figure3G-Source data1-a┬-actin.png]

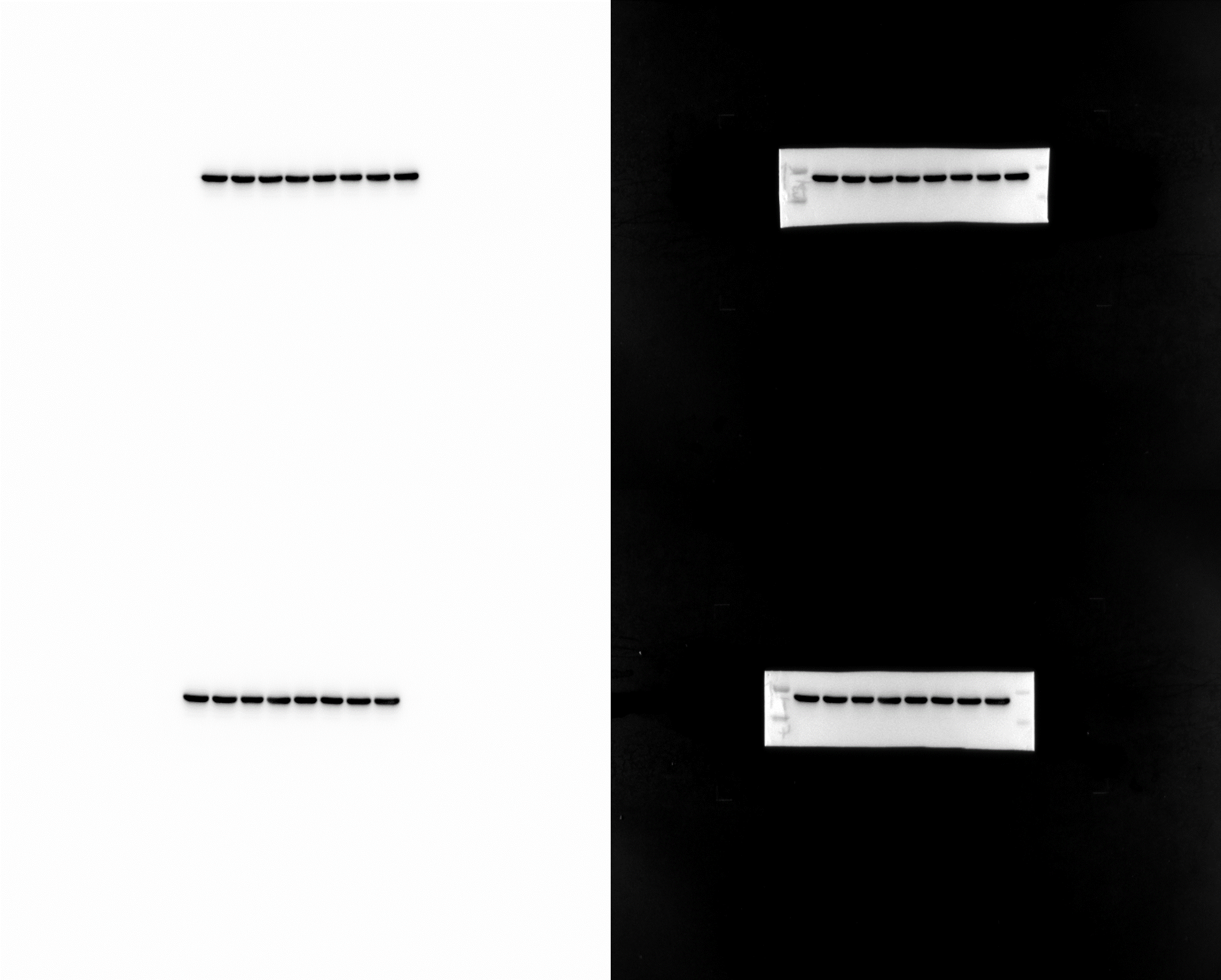

Supplement: Figure 3—source data 1. [file elife-96161-fig3-data1.zip › Figure 3-Source data1/Figure3G-Source data2-Tubulin.png]

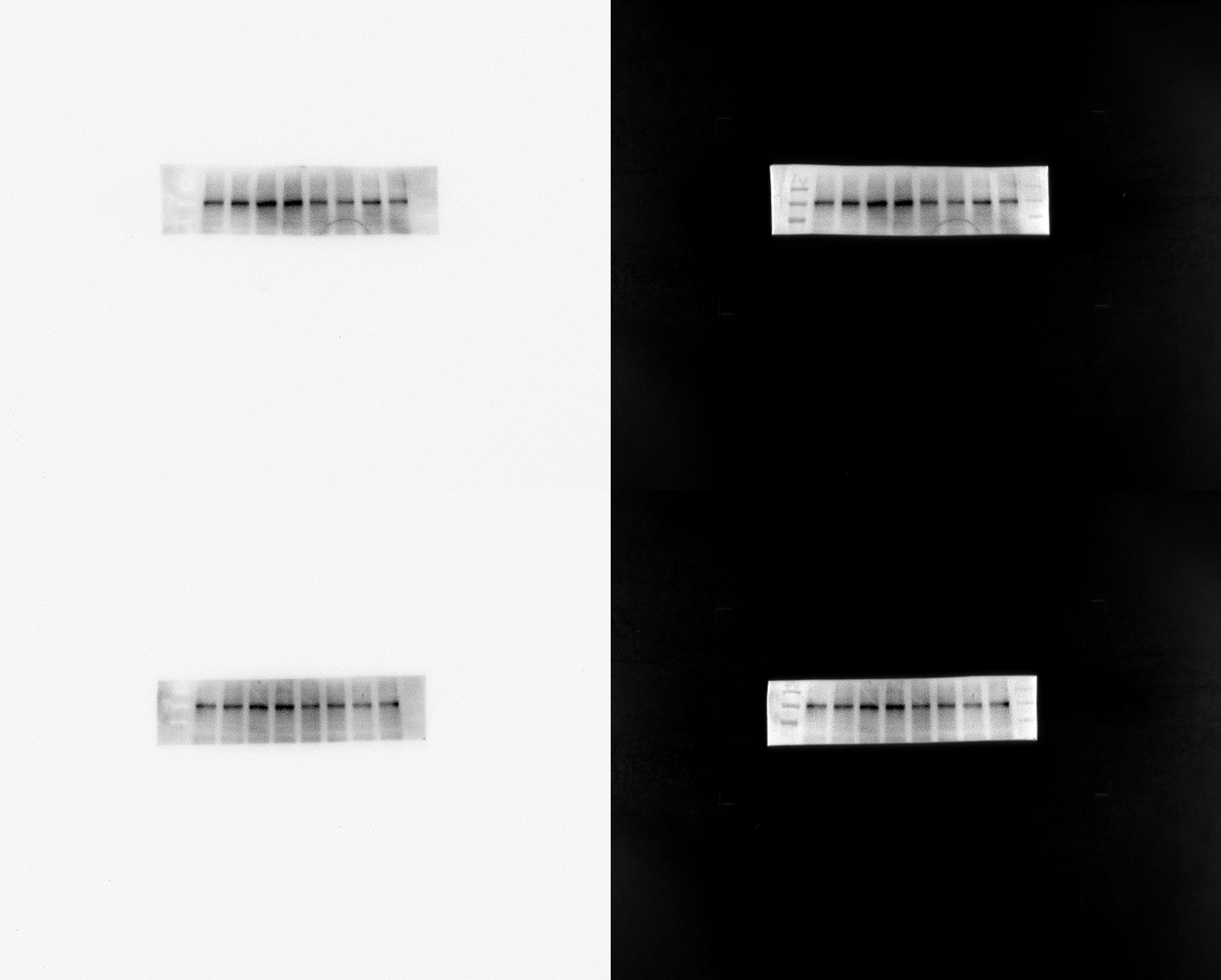

Supplement: Figure 3—source data 1. [file elife-96161-fig3-data1.zip › Figure 3-Source data1/Figure3G-Source data2-VE-Cadherin.png]

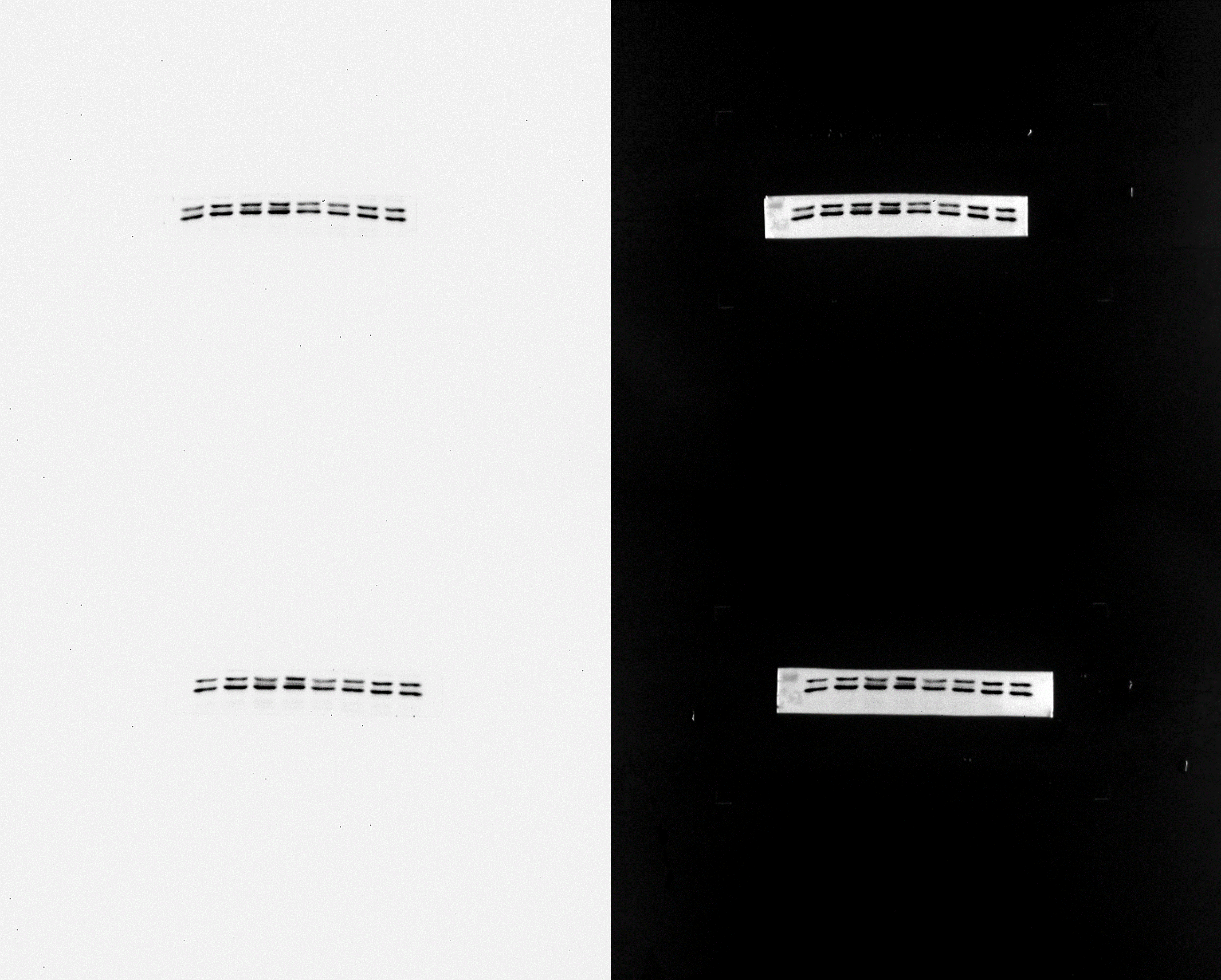

Supplement: Figure 3—source data 1. [file elife-96161-fig3-data1.zip › Figure 3-Source data1/Figure3G-Source data3-ERK.png]

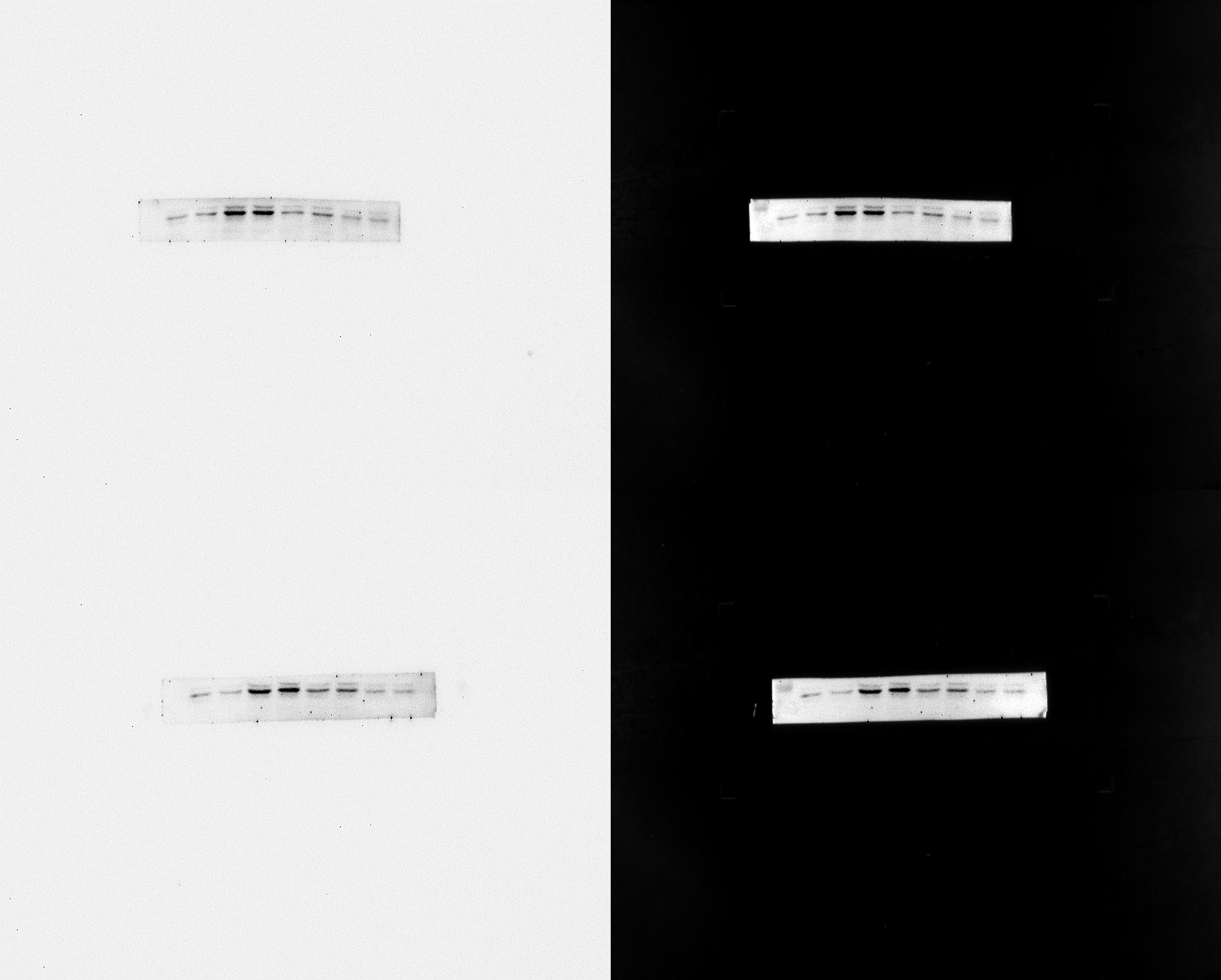

Supplement: Figure 3—source data 1. [file elife-96161-fig3-data1.zip › Figure 3-Source data1/Figure3G-Source data3-p-ERK.png]

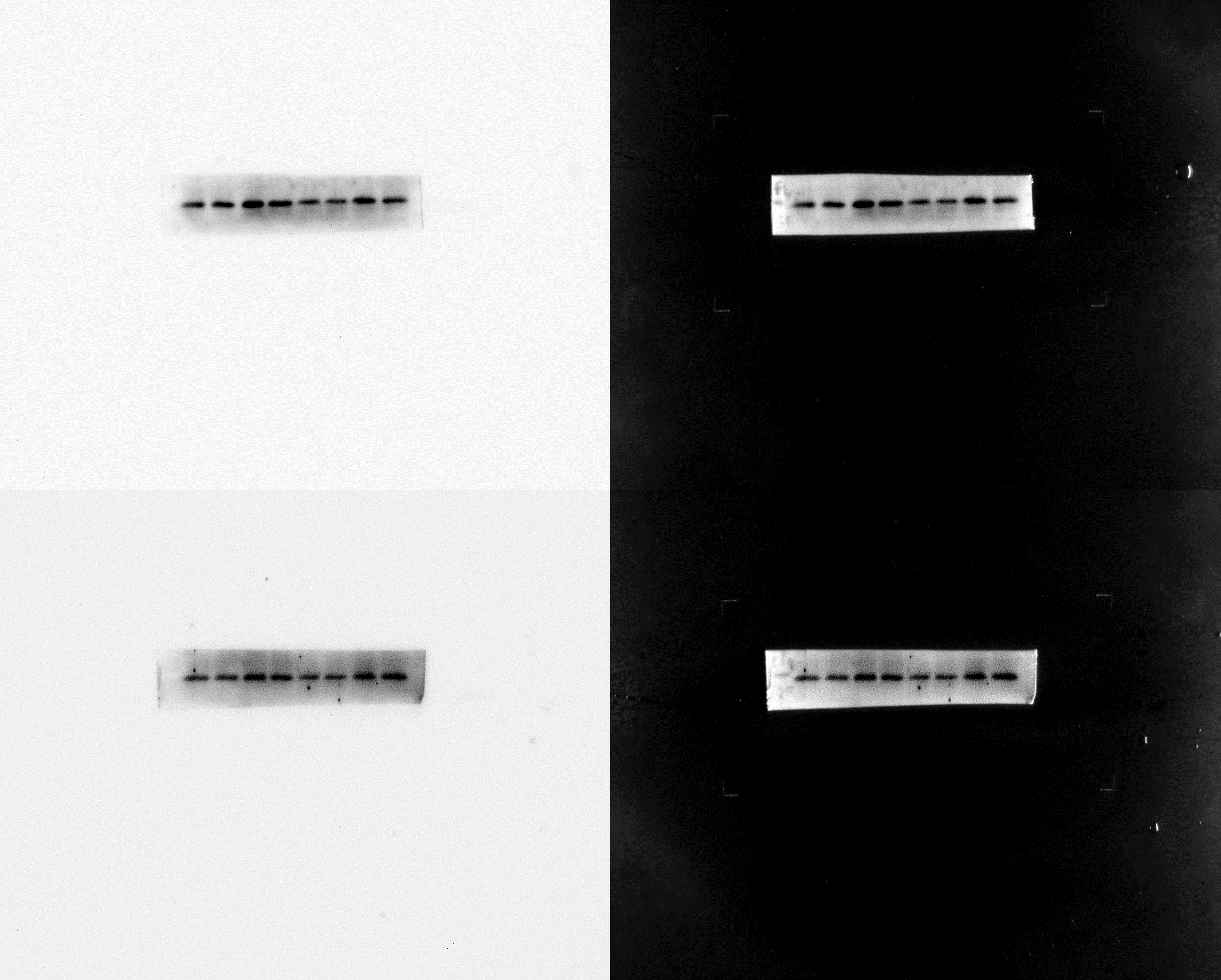

Supplement: Figure 3—source data 1. [file elife-96161-fig3-data1.zip › Figure 3-Source data1/Figure3H-Source data1-Claudin-5.png]

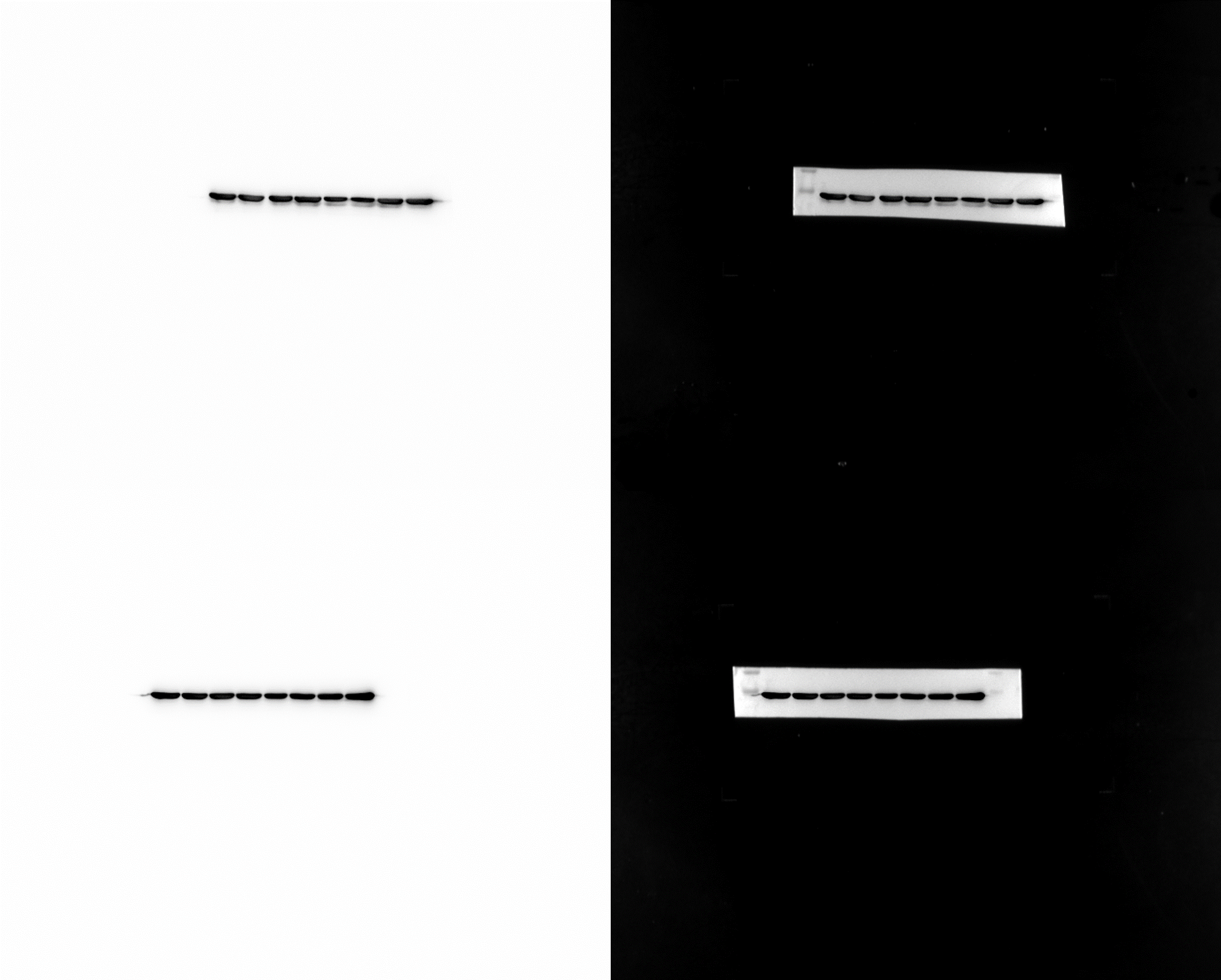

Supplement: Figure 3—source data 1. [file elife-96161-fig3-data1.zip › Figure 3-Source data1/Figure3H-Source data1-a┬-actin.png]

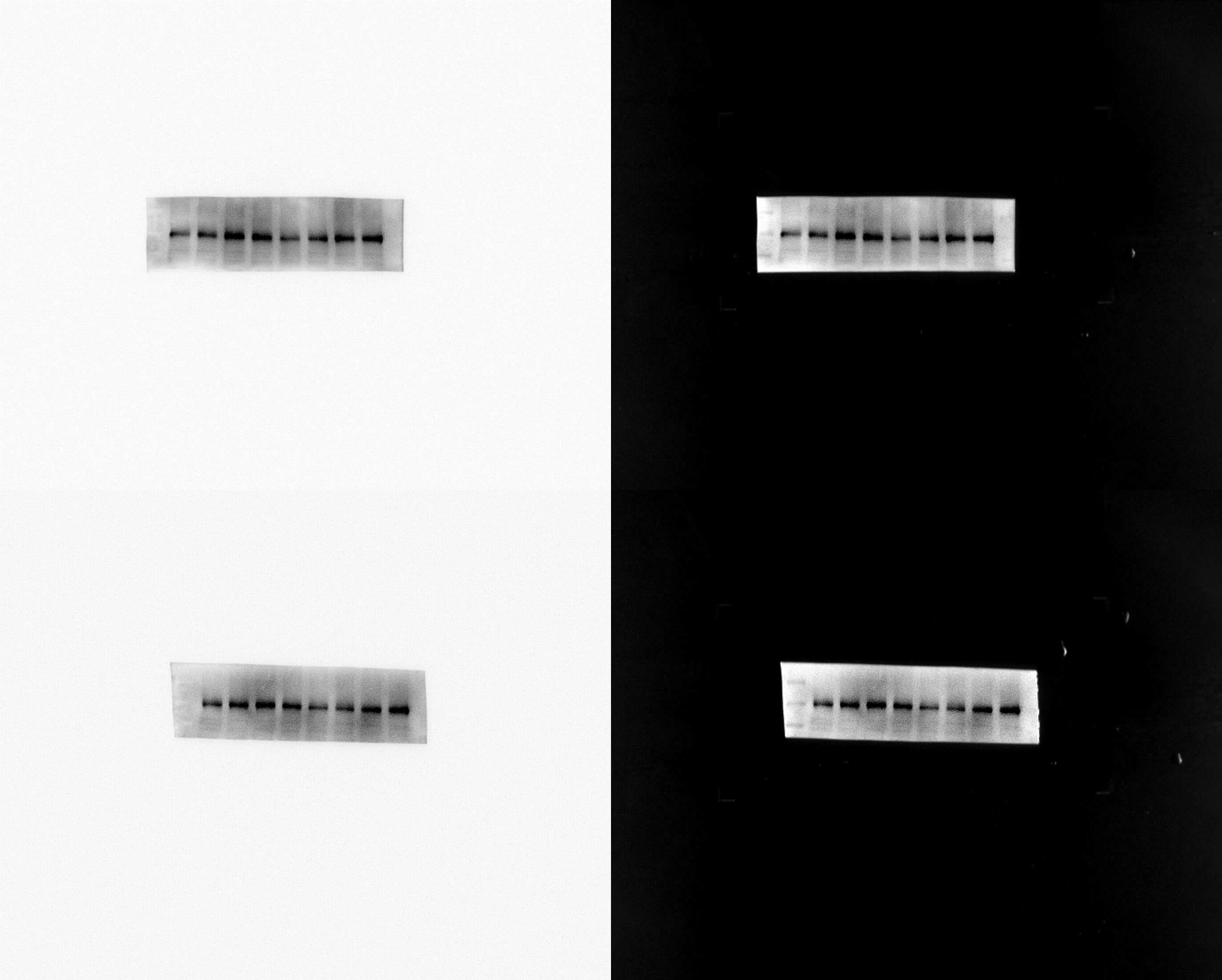

Supplement: Figure 3—source data 1. [file elife-96161-fig3-data1.zip › Figure 3-Source data1/Figure3H-Source data2-VE-Cadherin.png]

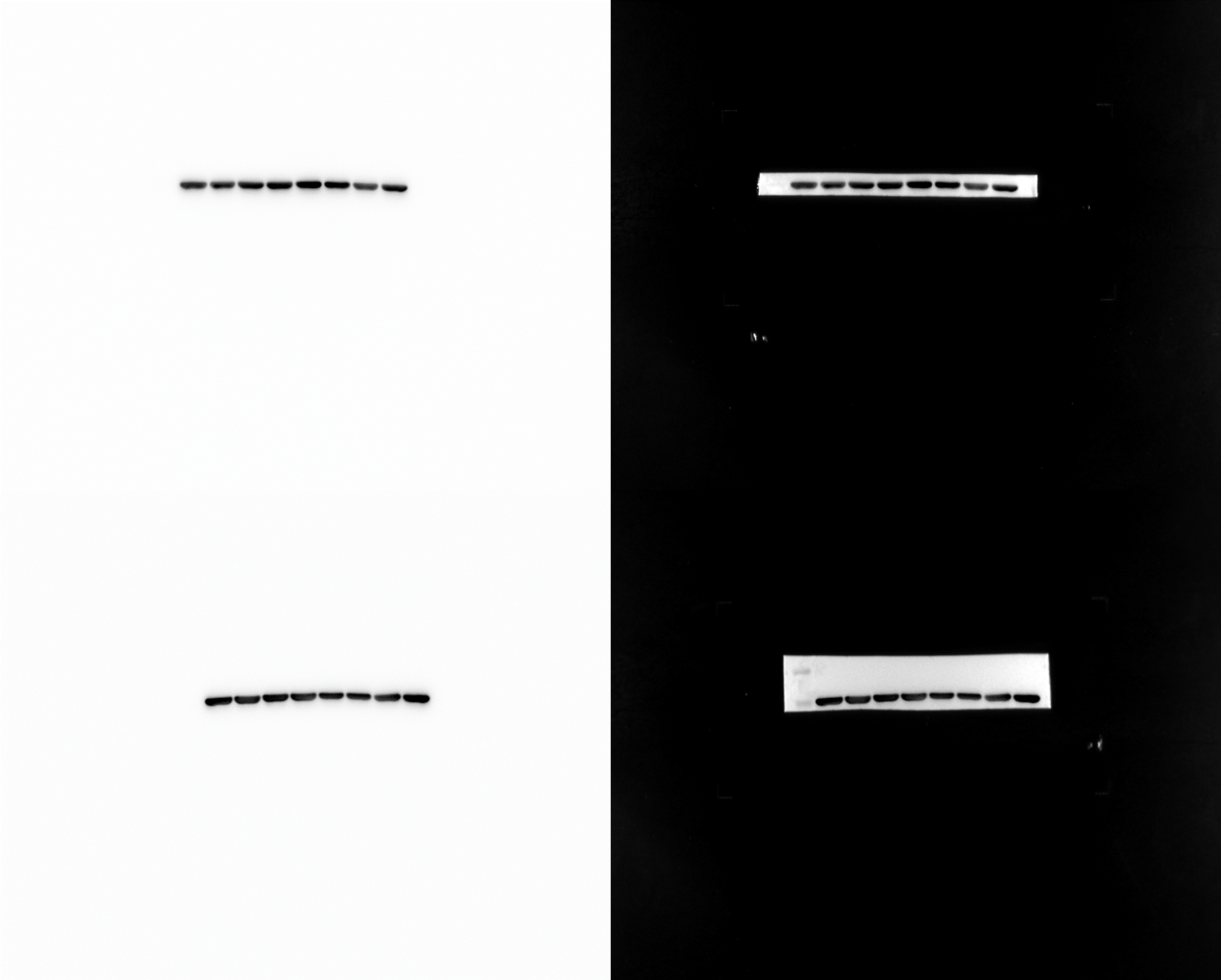

Supplement: Figure 3—source data 1. [file elife-96161-fig3-data1.zip › Figure 3-Source data1/Figure3H-Source data2-a┬-actin.png]

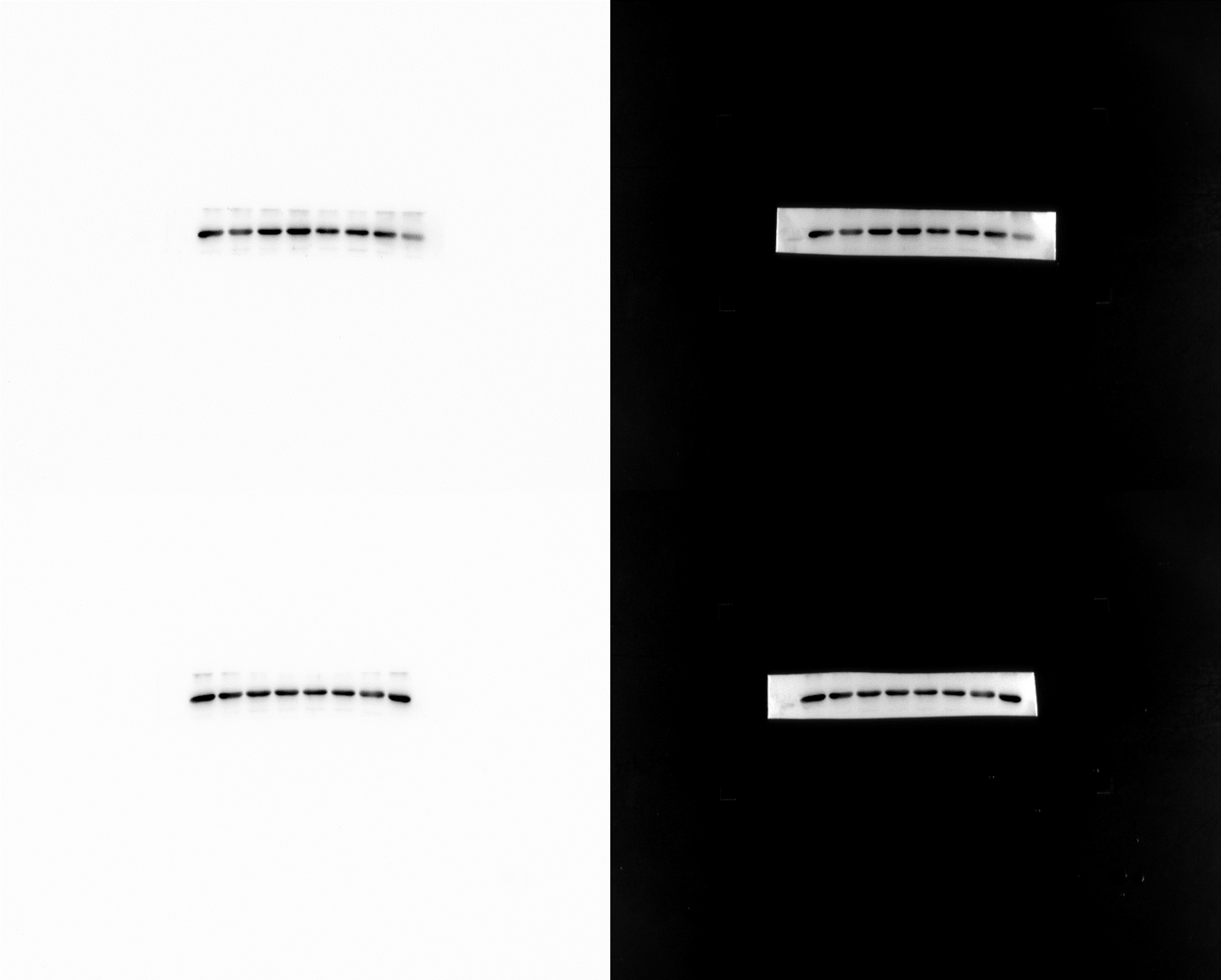

Supplement: Figure 3—source data 1. [file elife-96161-fig3-data1.zip › Figure 3-Source data1/Figure3H-Source data3-JNK.png]

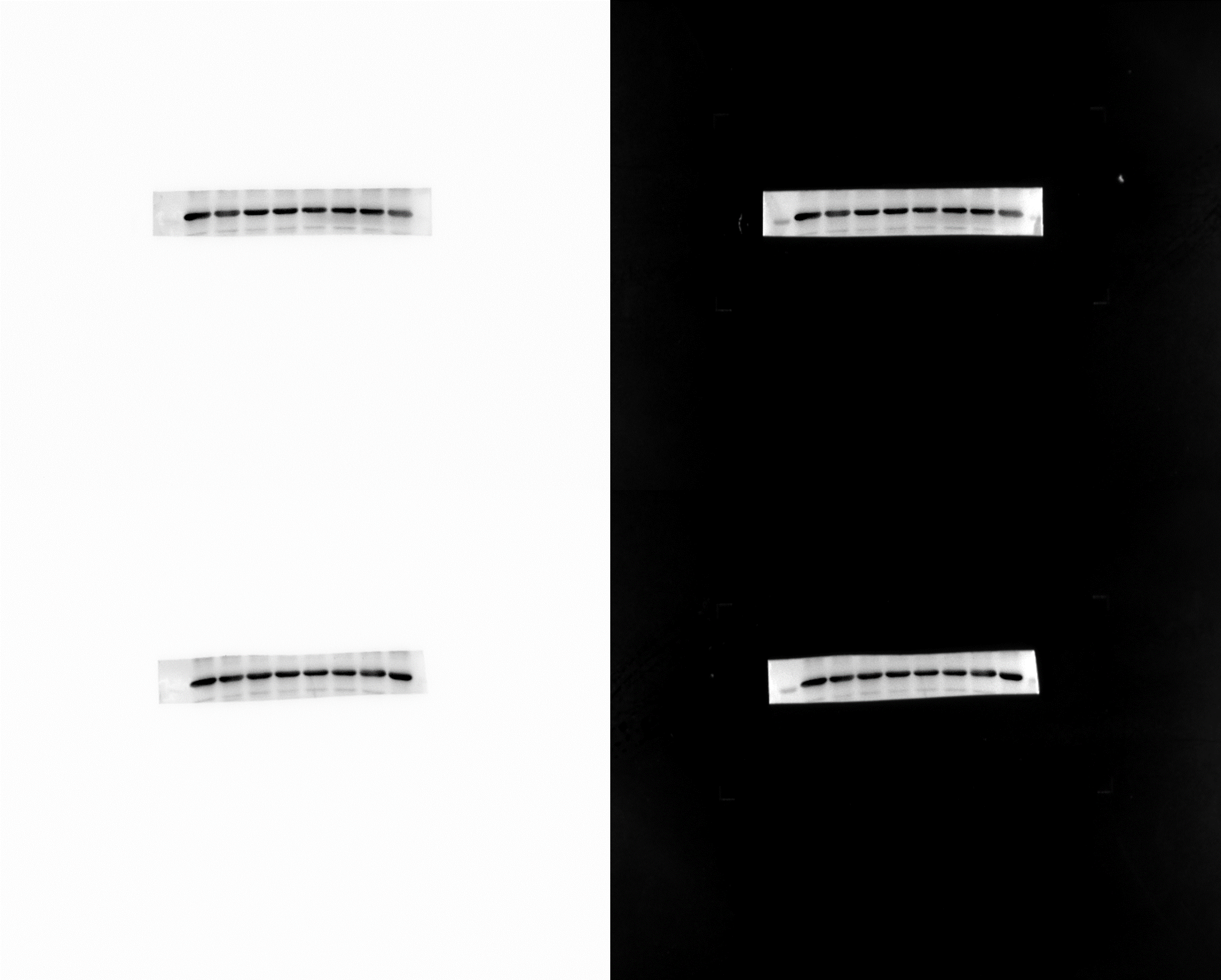

Supplement: Figure 3—source data 1. [file elife-96161-fig3-data1.zip › Figure 3-Source data1/Figure3H-Source data3-p-JNK.png]
