# Supplementary material for: Neurons enhance blood–brain barrier function via upregulating claudin-5 and VE-cadherin expression due to glial cell line-derived neurotrophic factor secretion
Source: eLife. 2024 Oct 30;13:RP96161. doi: 10.7554/eLife.96161 (PMC11524583; doi:10.7554/eLife.96161)

Figure 1K-Claudin-5

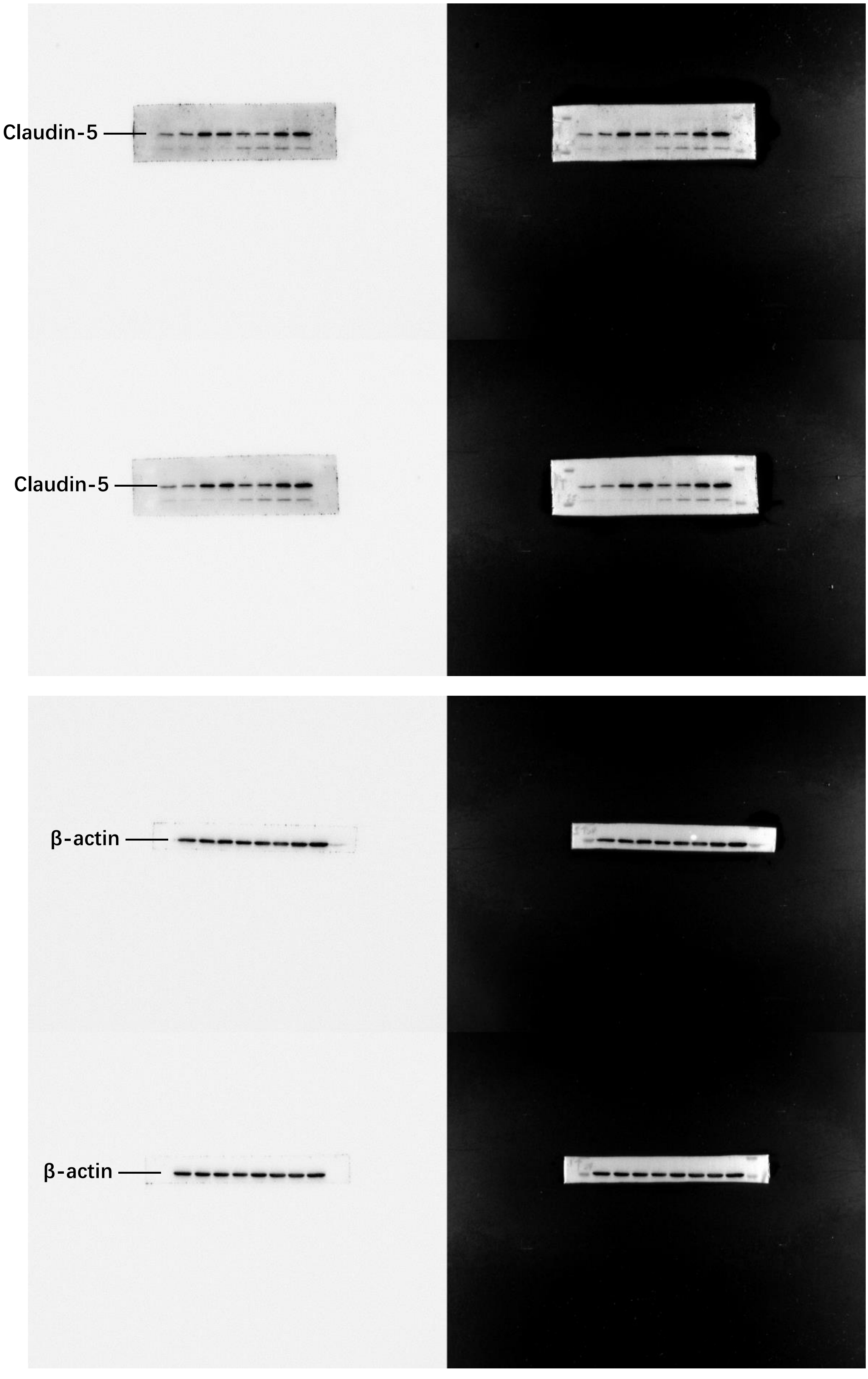

Figure 1K-ZO-1

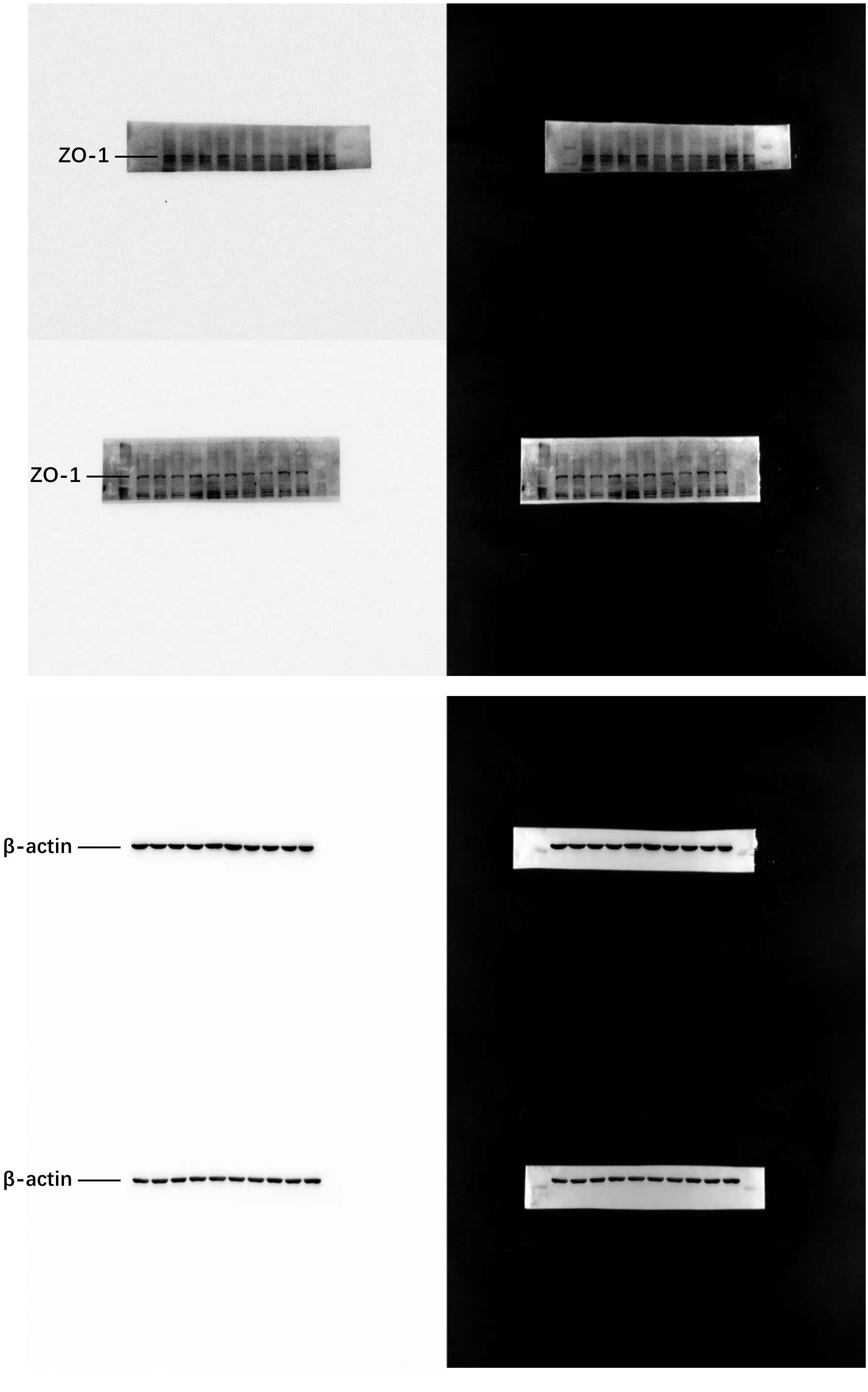

Figure 1K-Occludin

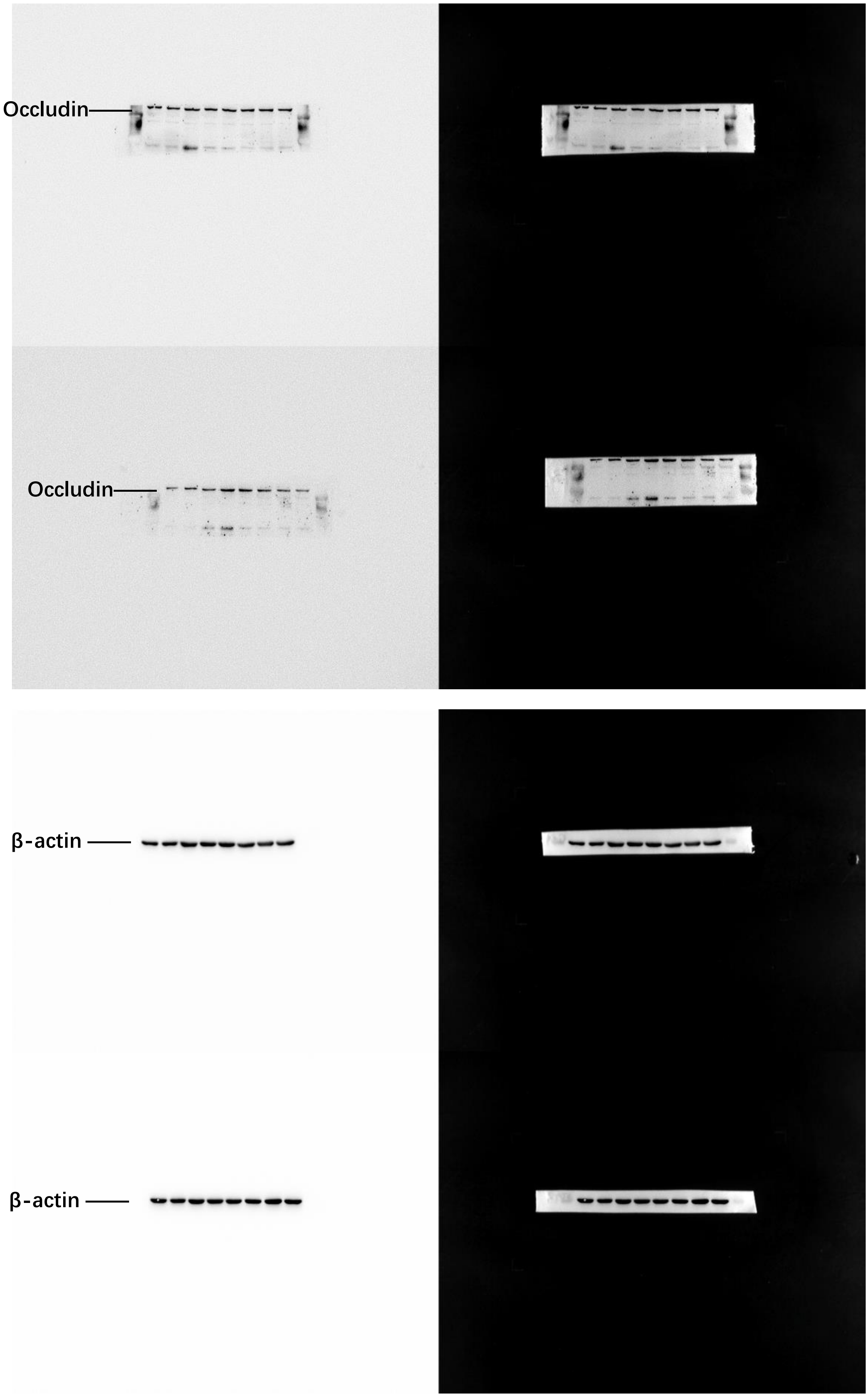

Figure 1M -VE-Cadherin

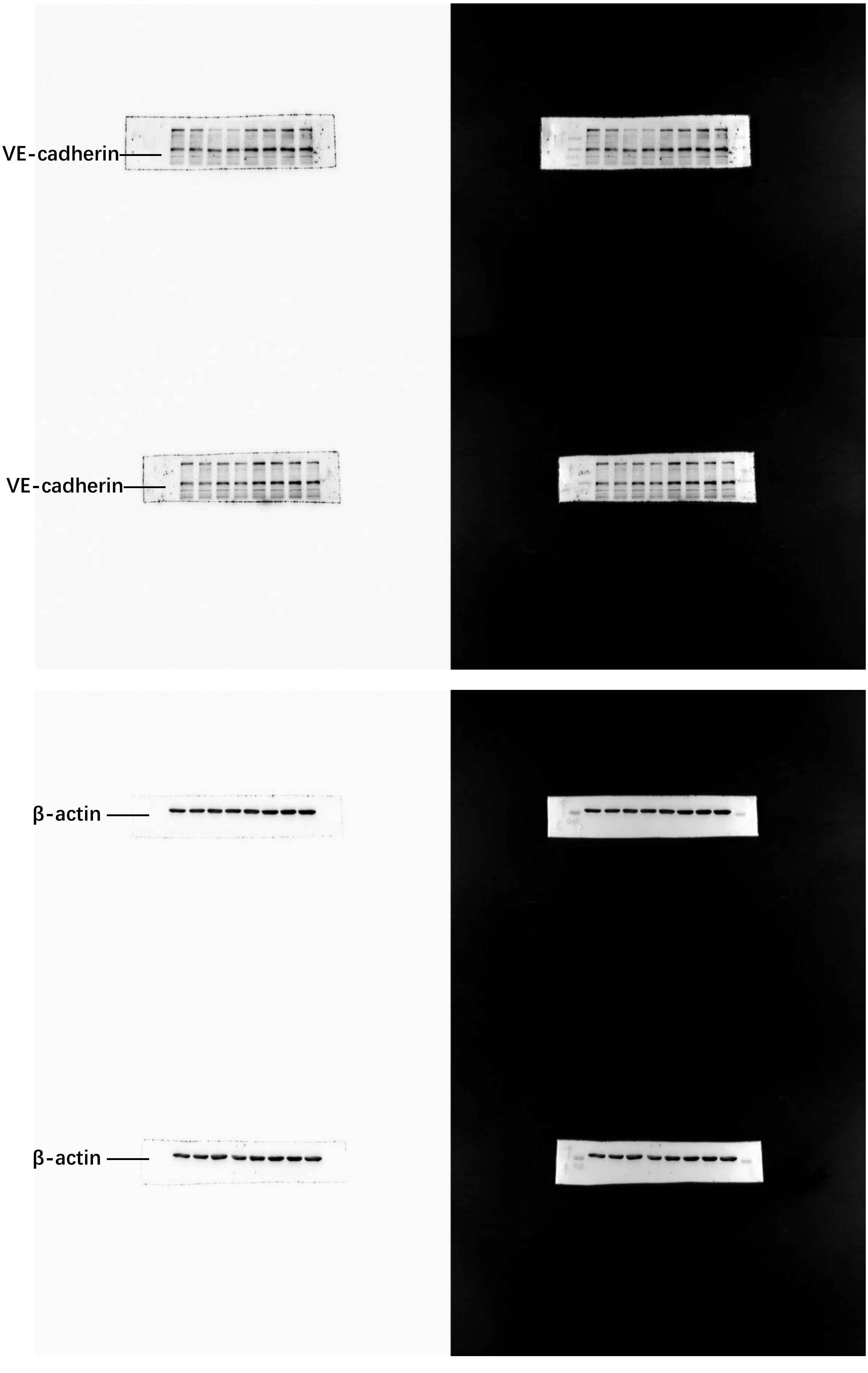

Figure 1M-β-catenin

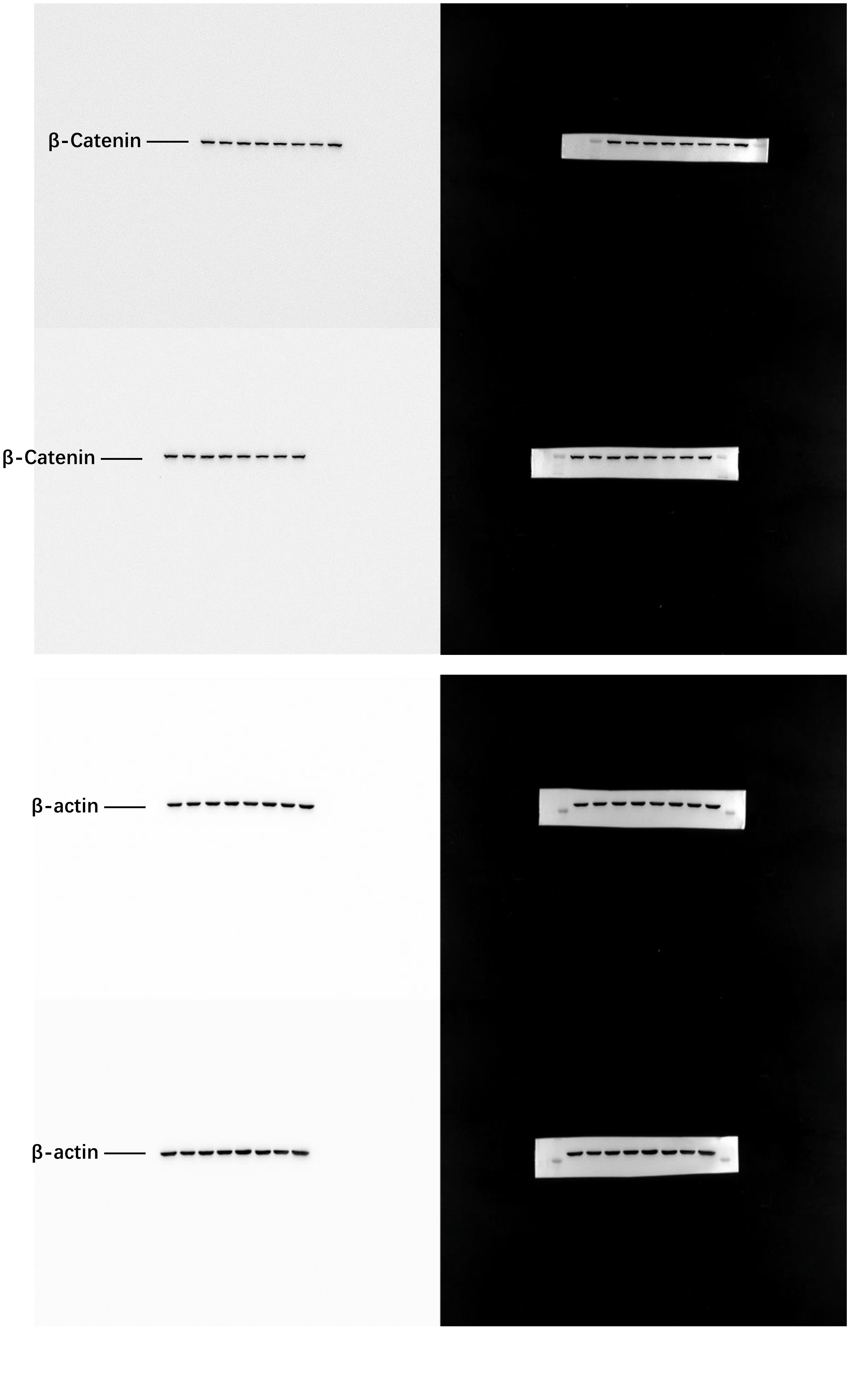

Figure 1M-BCRP

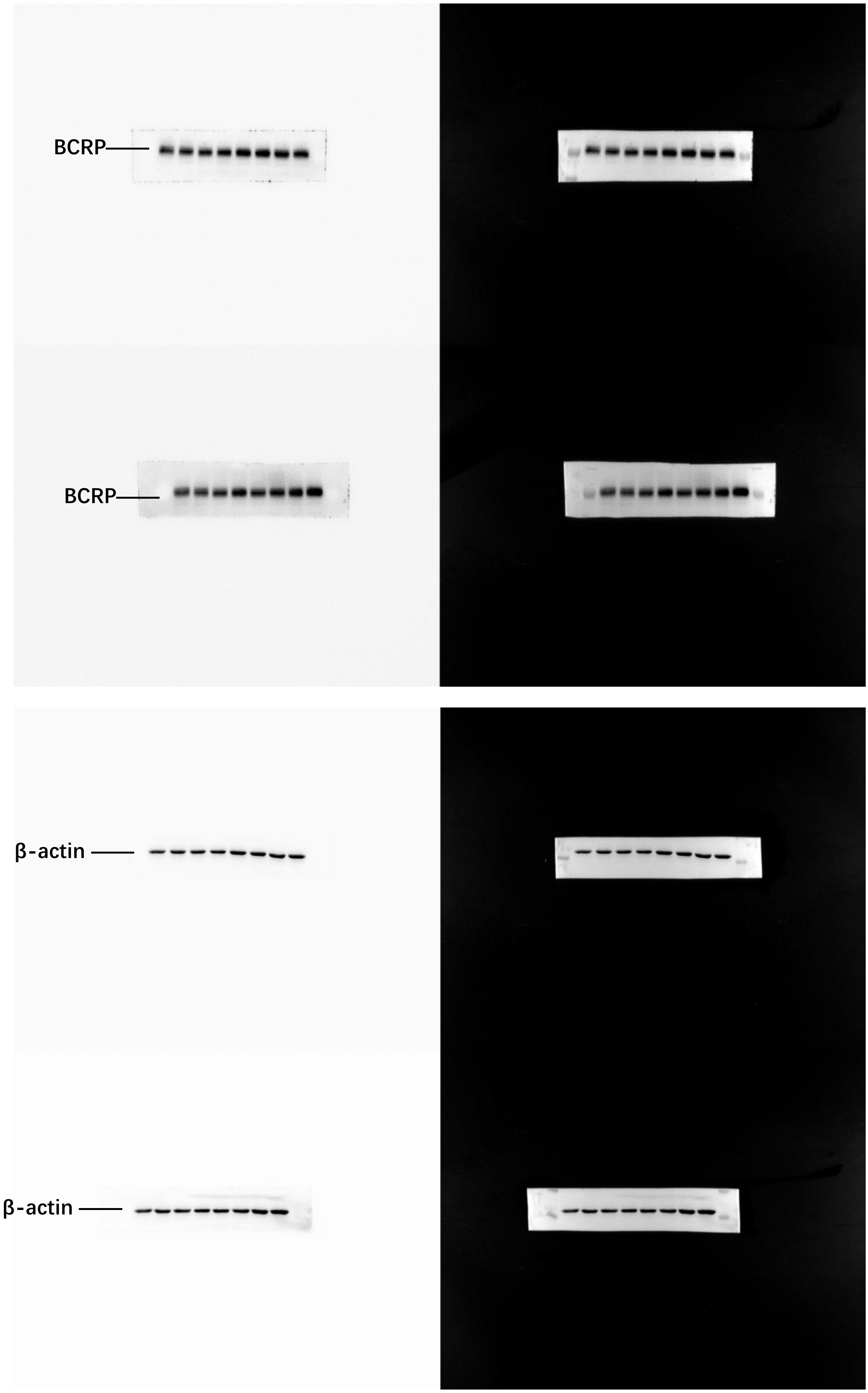

Supplement: Figure 1—source data 2. [file elife-96161-fig1-data2.zip › Figure 1-Source data2/Figure 1-Annotated western blots.pdf]
