# Supplementary material for: Neurons enhance blood–brain barrier function via upregulating claudin-5 and VE-cadherin expression due to glial cell line-derived neurotrophic factor secretion
Source: eLife. 2024 Oct 30;13:RP96161. doi: 10.7554/eLife.96161 (PMC11524583; doi:10.7554/eLife.96161)

Figure 2A-Claudin-5

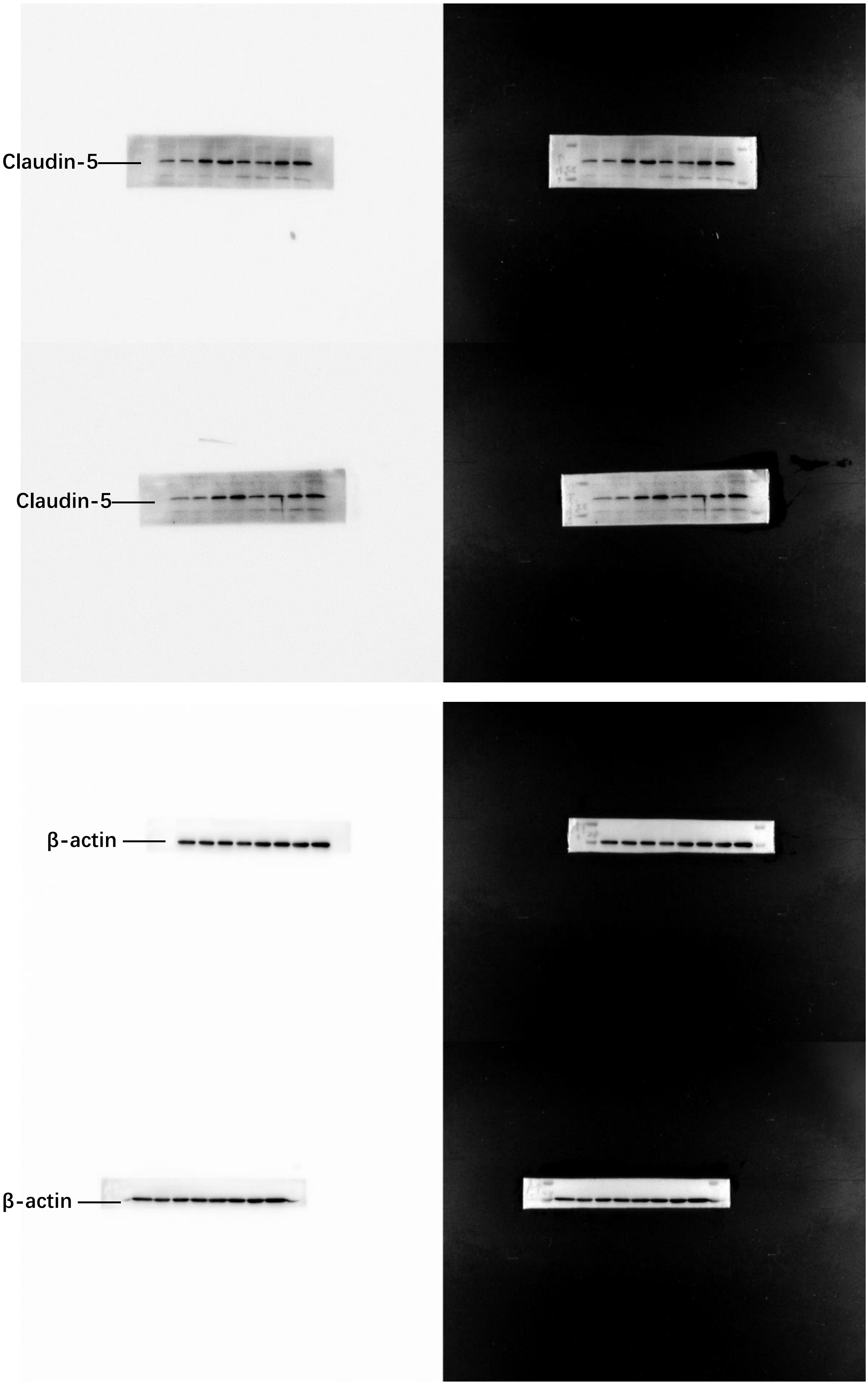

Figure 2A-VE-cadherin

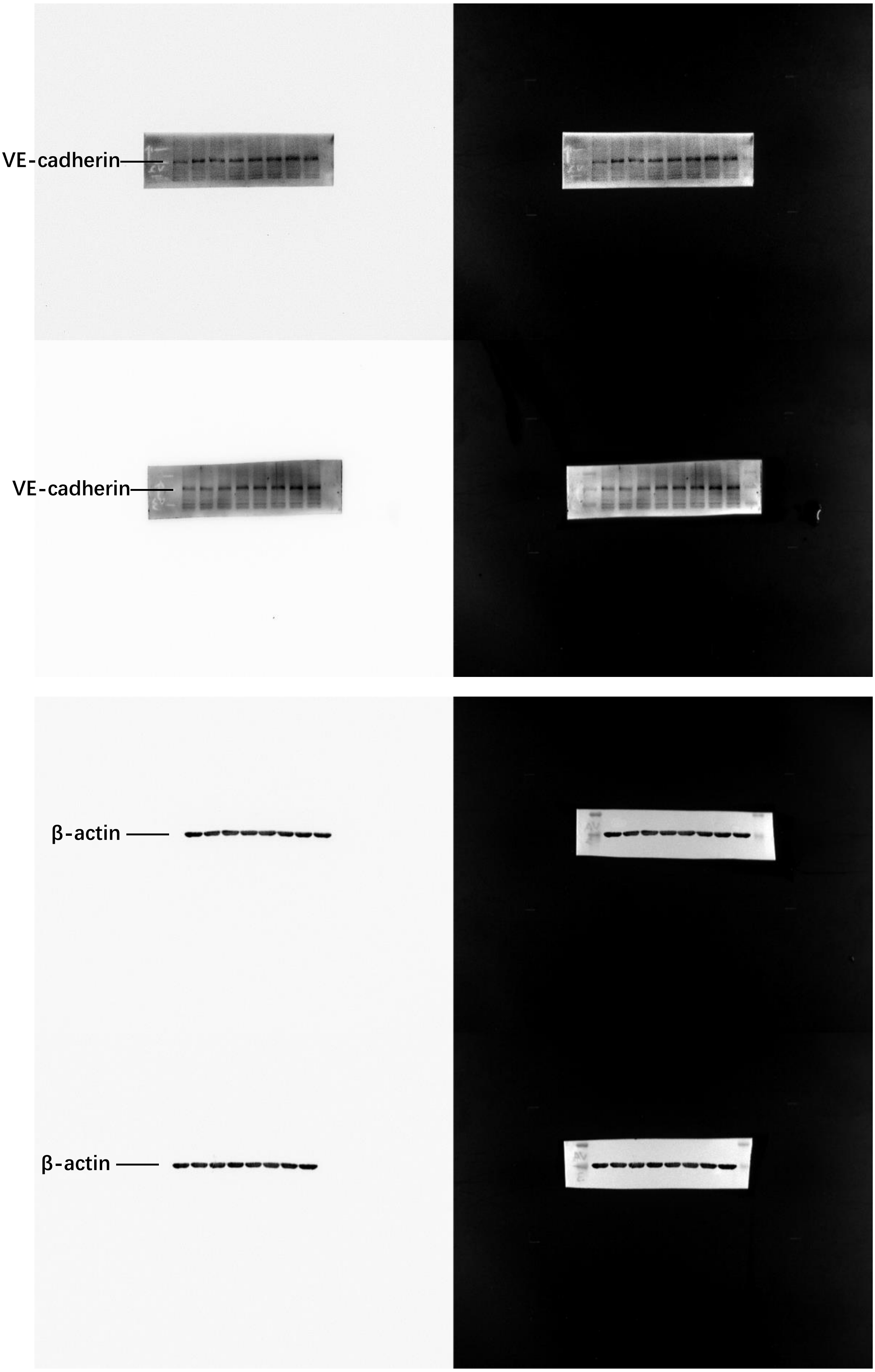

Figure 2D-Claudin-5

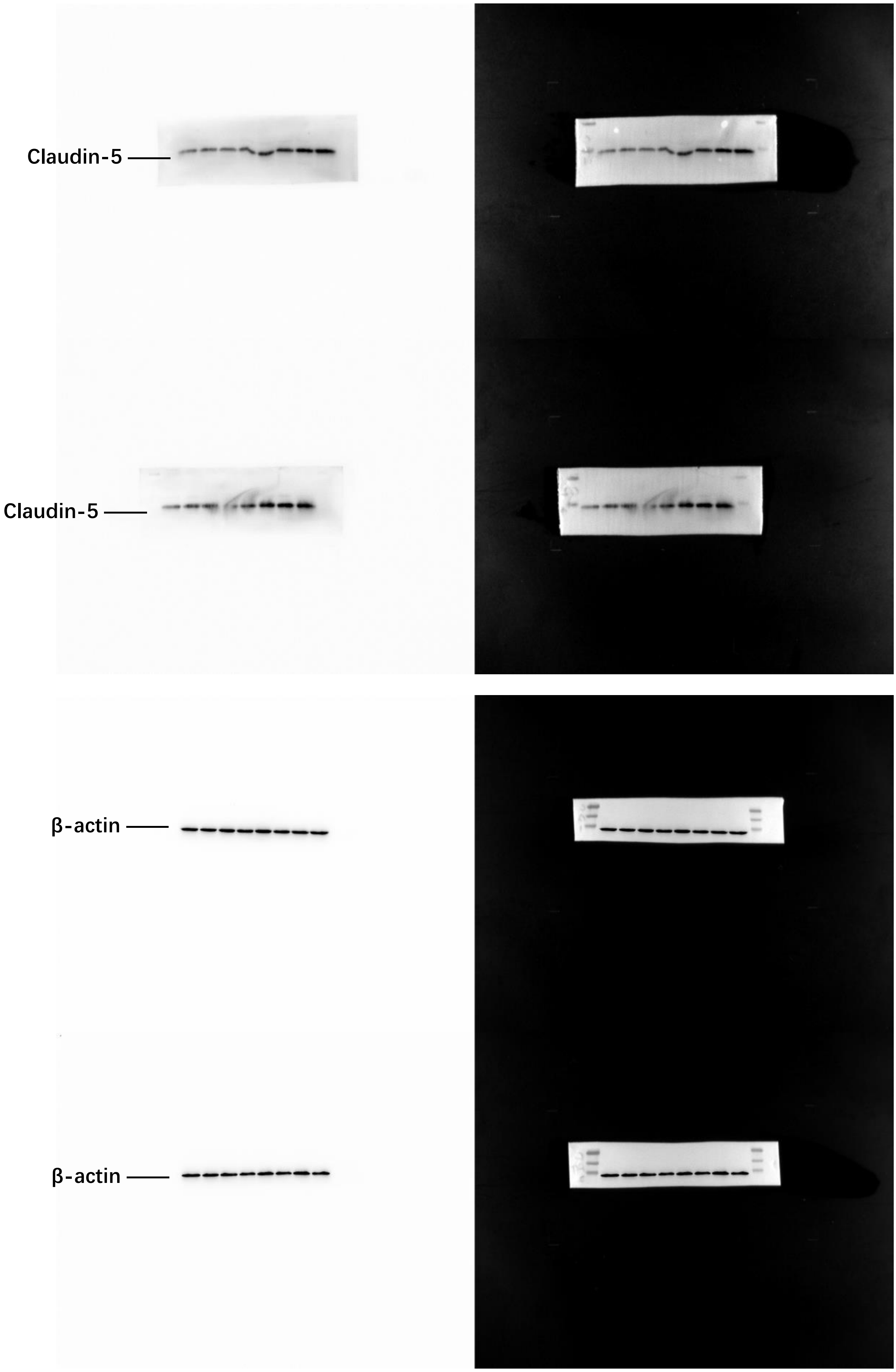

Figure 2D-VE-cadherin

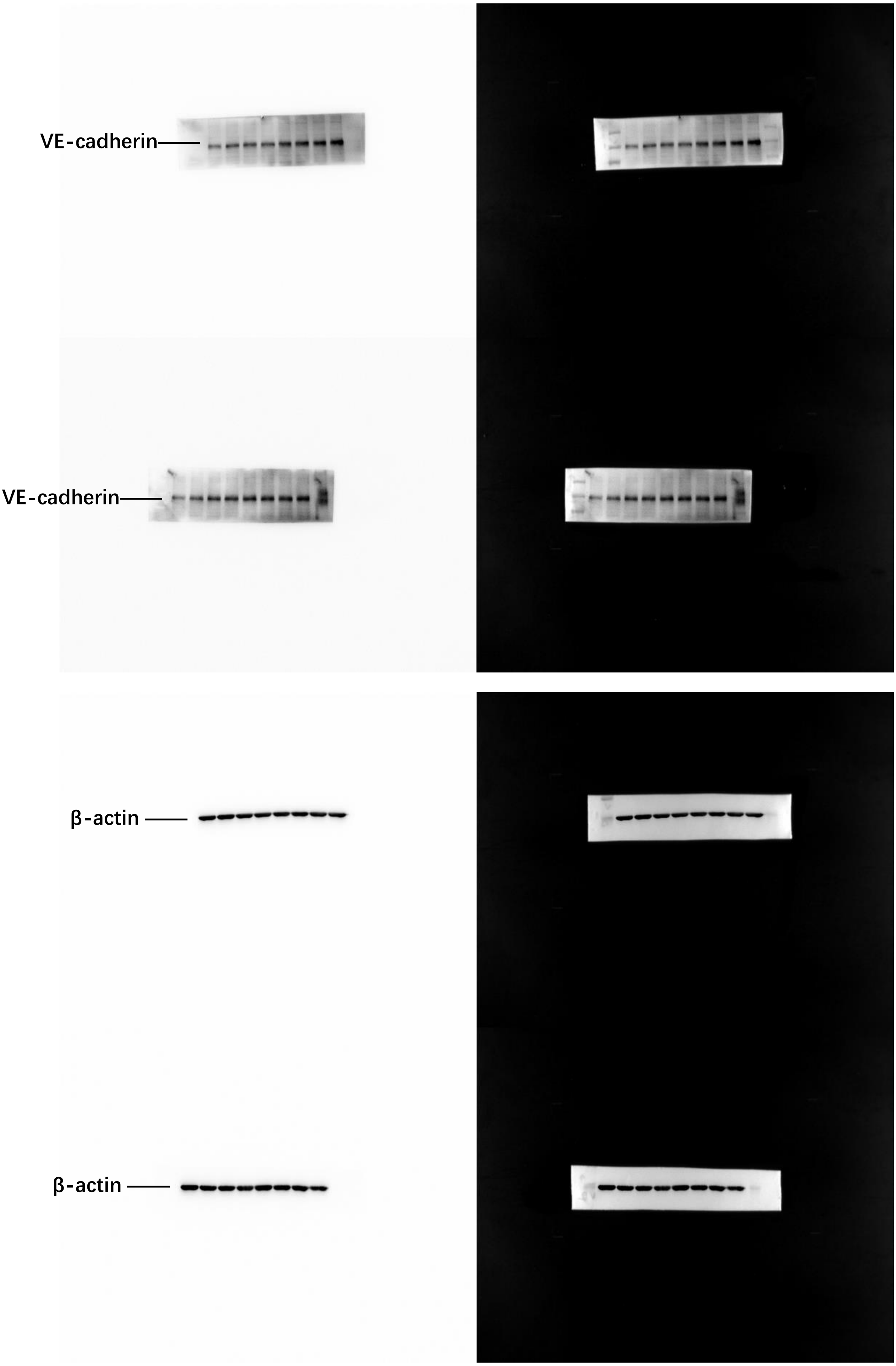

Figure 2E-Claudin-5

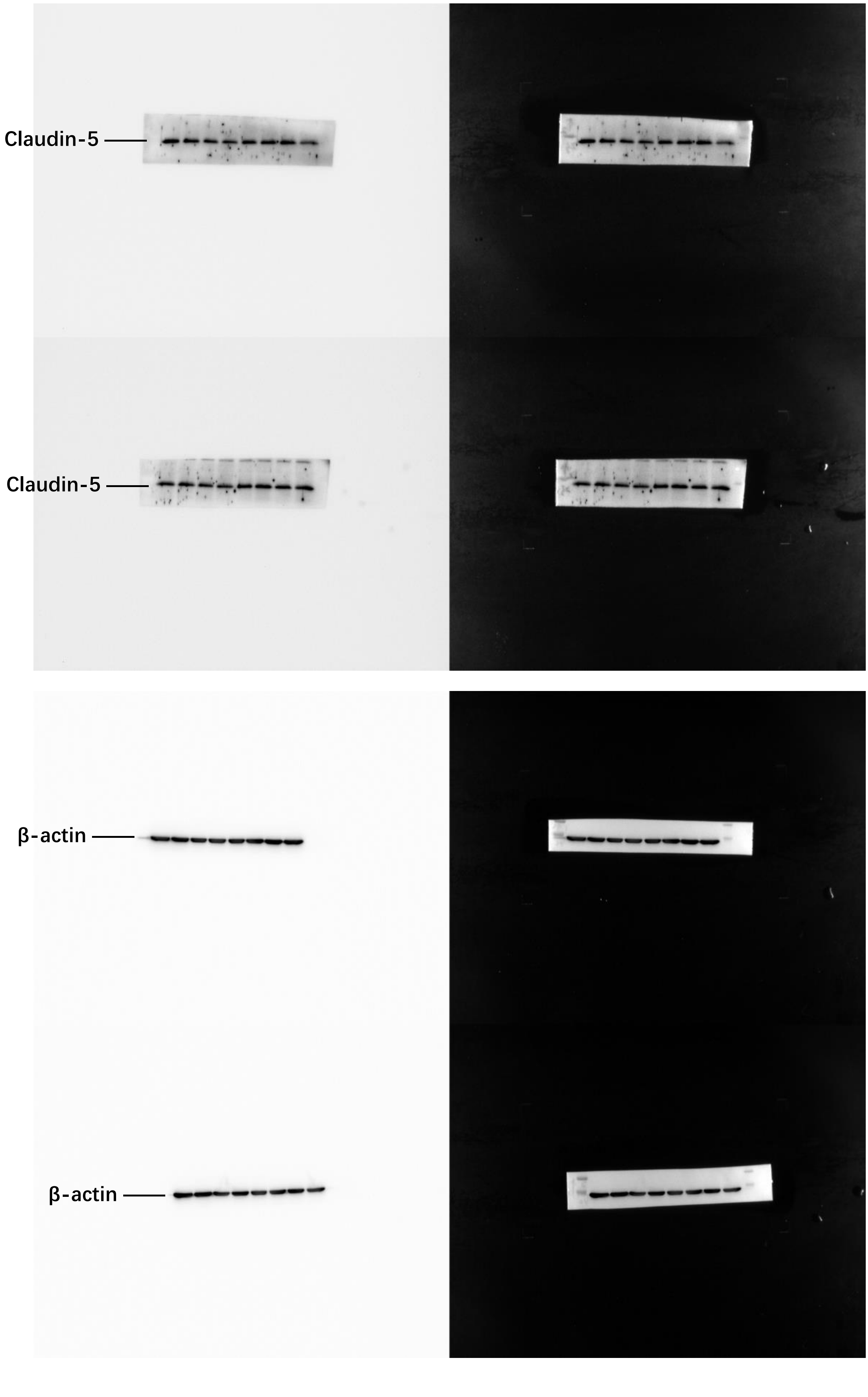

Figure 2E-VE-cadherin

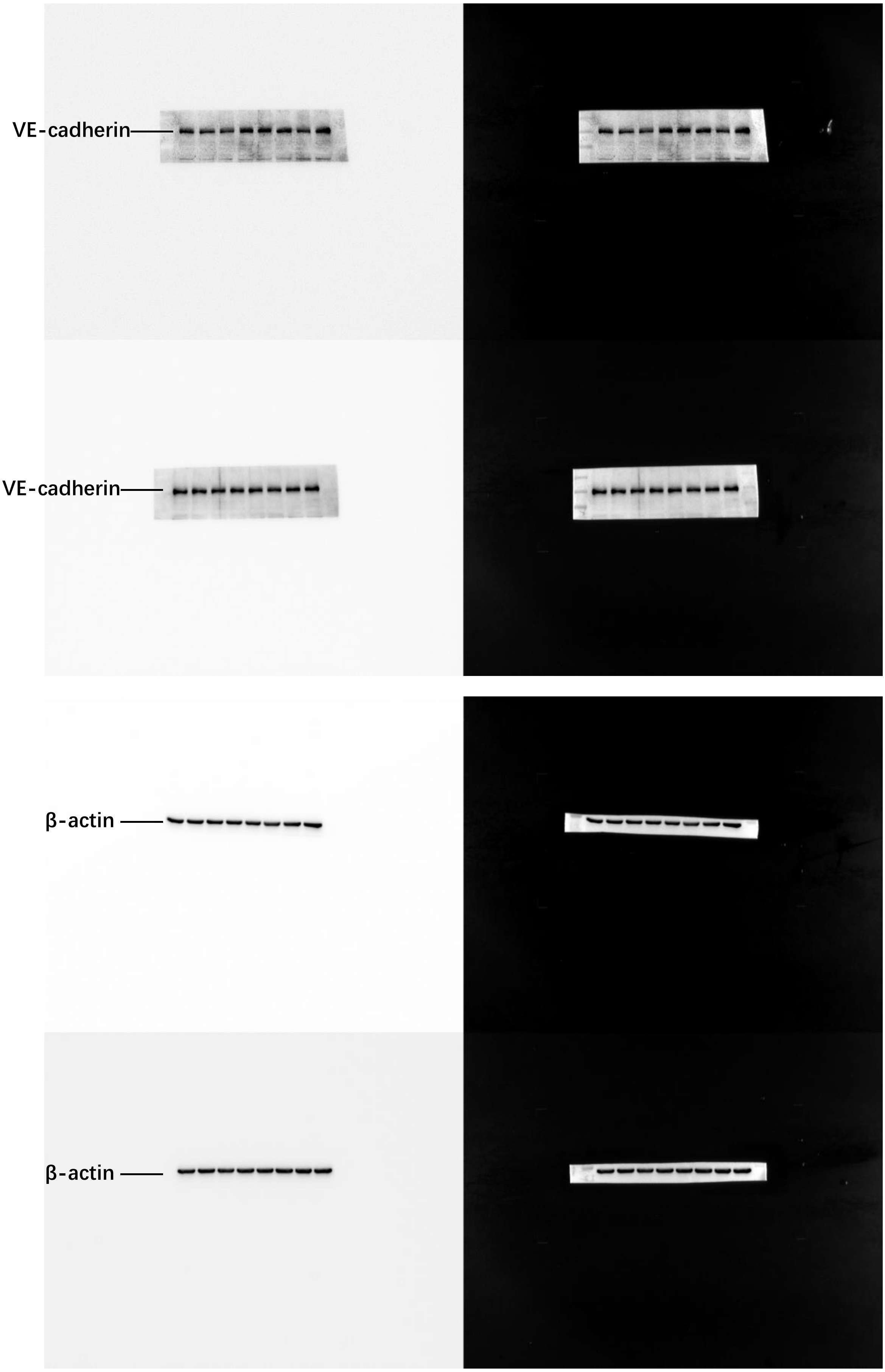

Figure 2F-Claudin-5

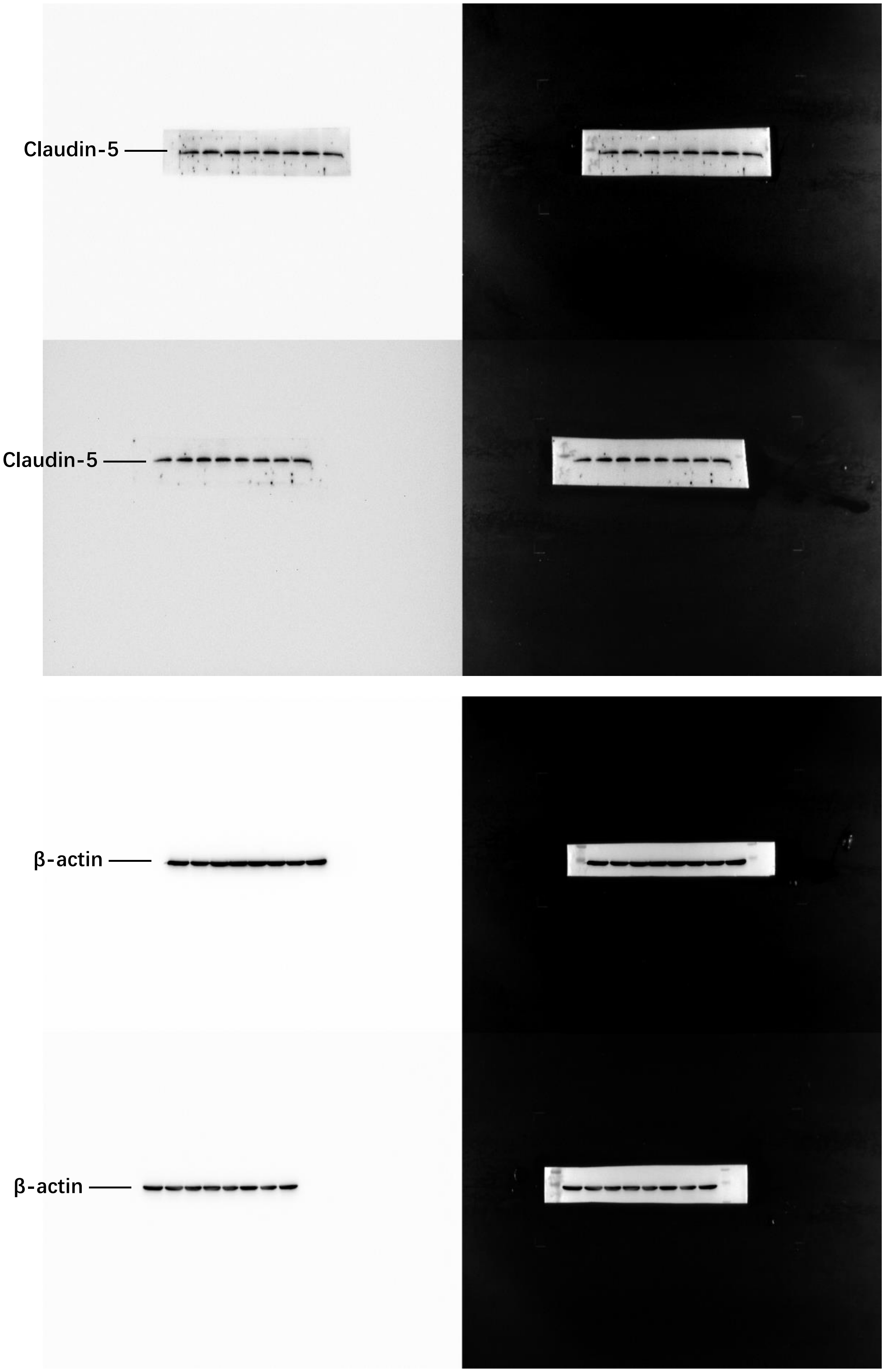

Figure 2F-VE-cadherin

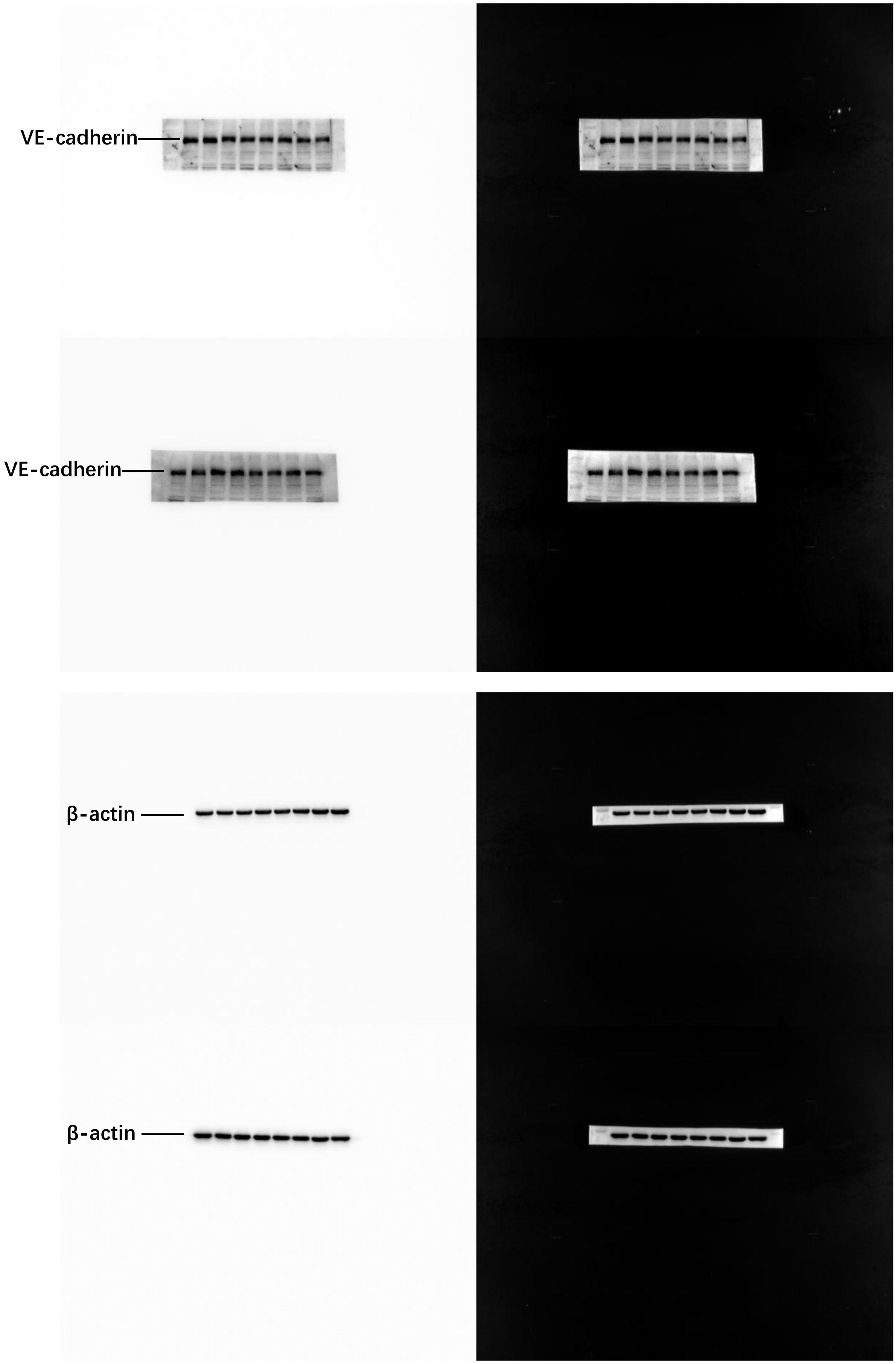

Figure 2G-Claudin-5

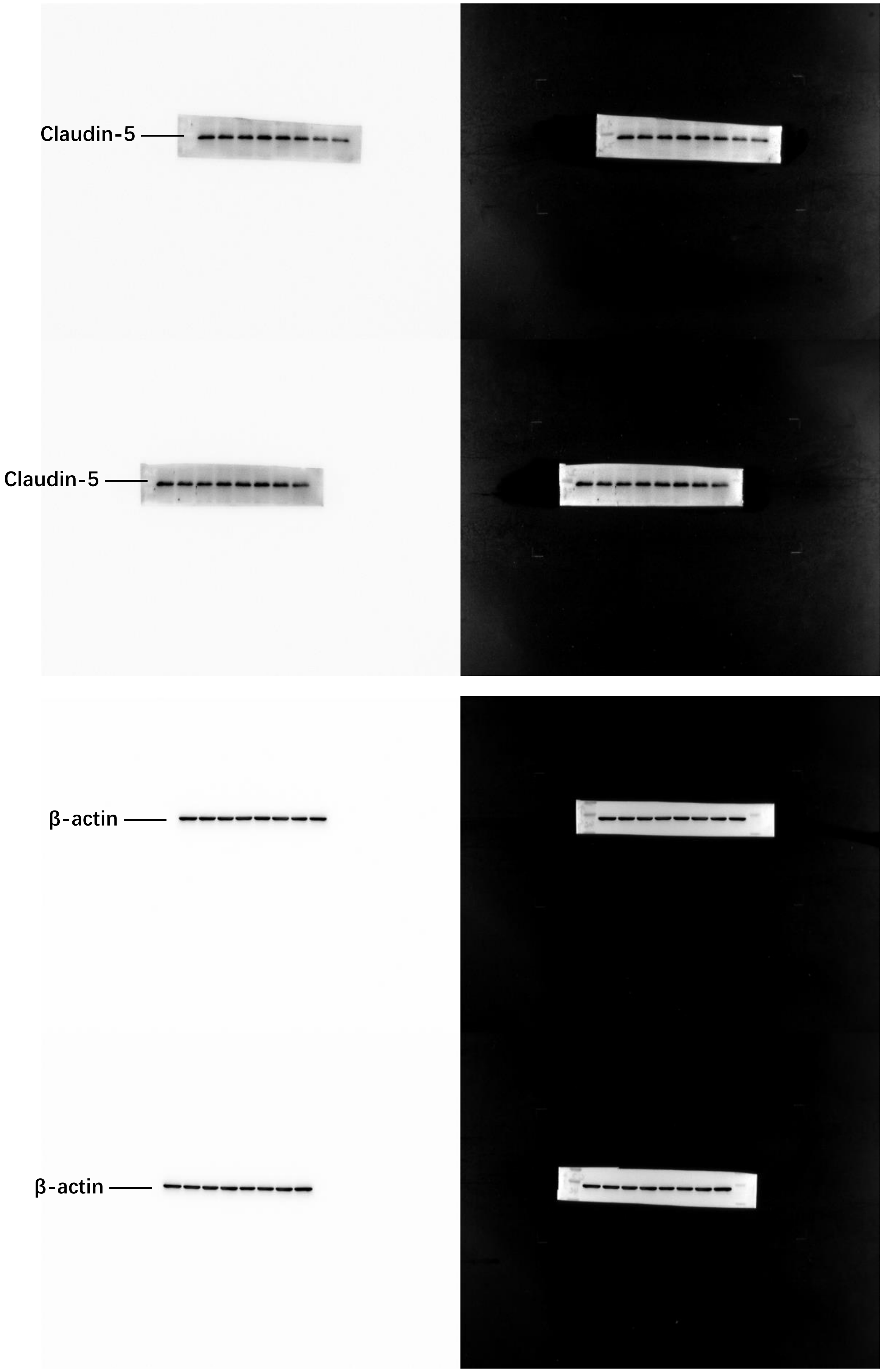

Figure 2G-VE-cadherin

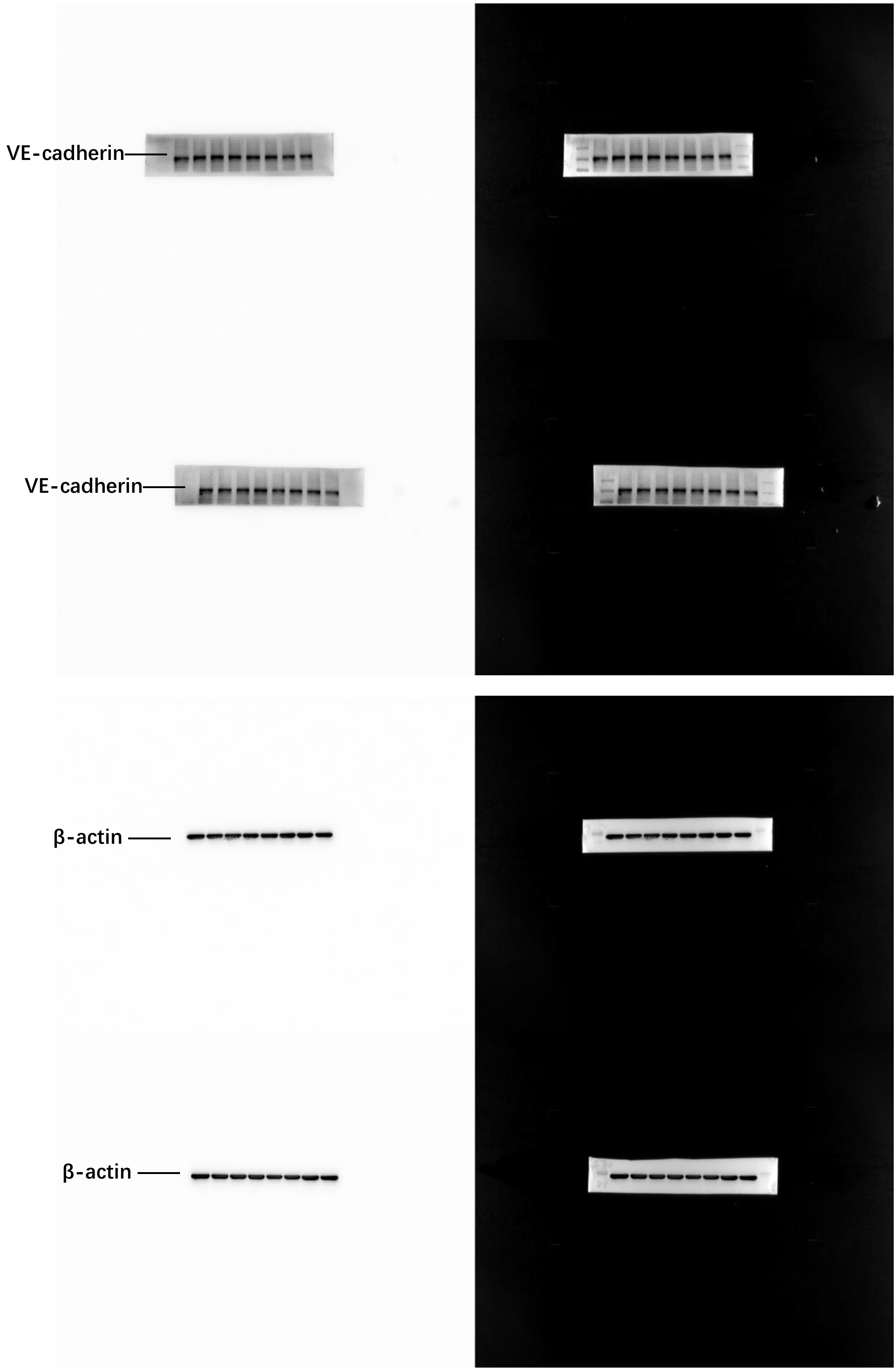

Figure 2H-Claudin-5

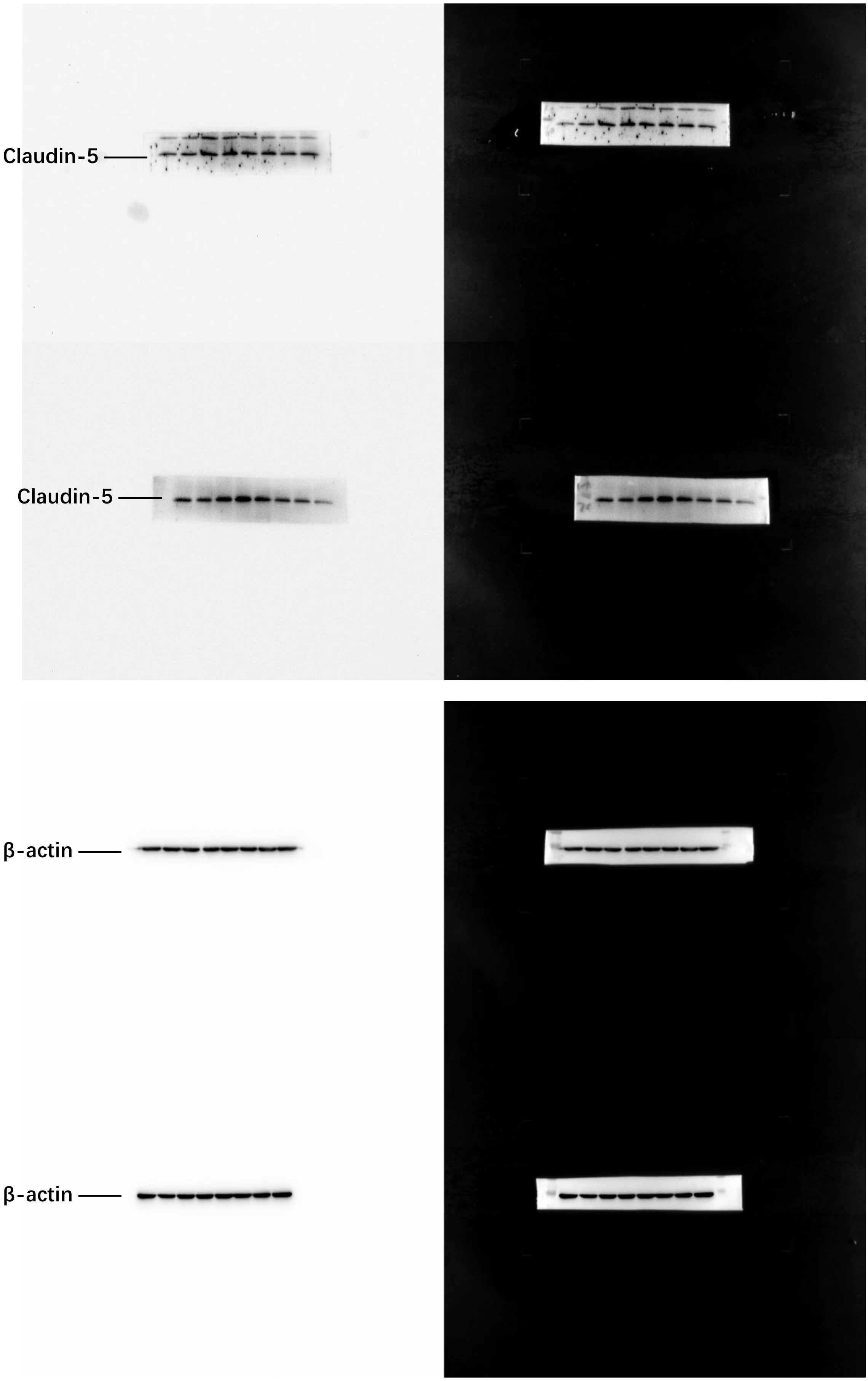

Figure 2H-VE-cadherin

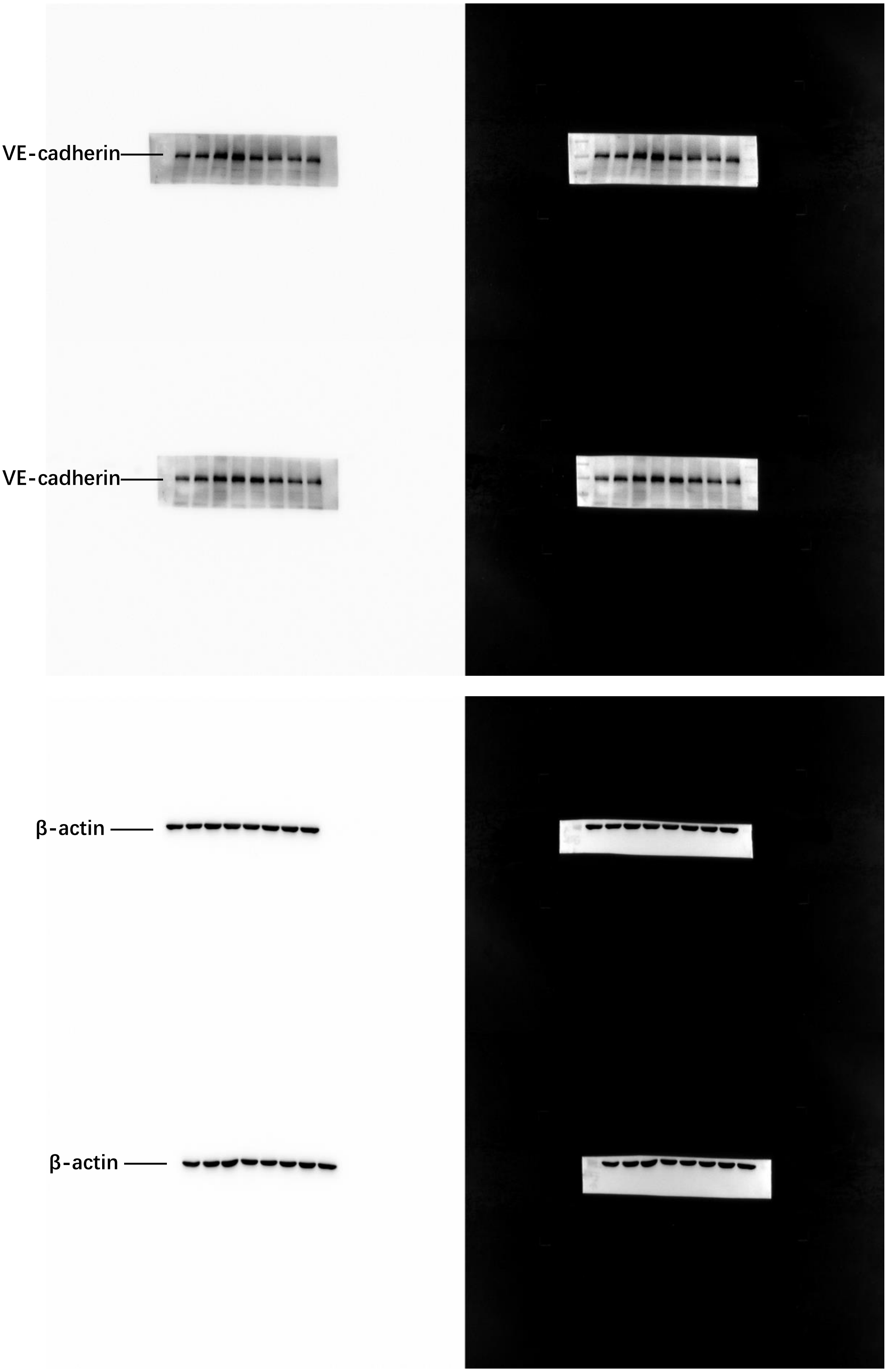

Figure 2I-Claudin-5

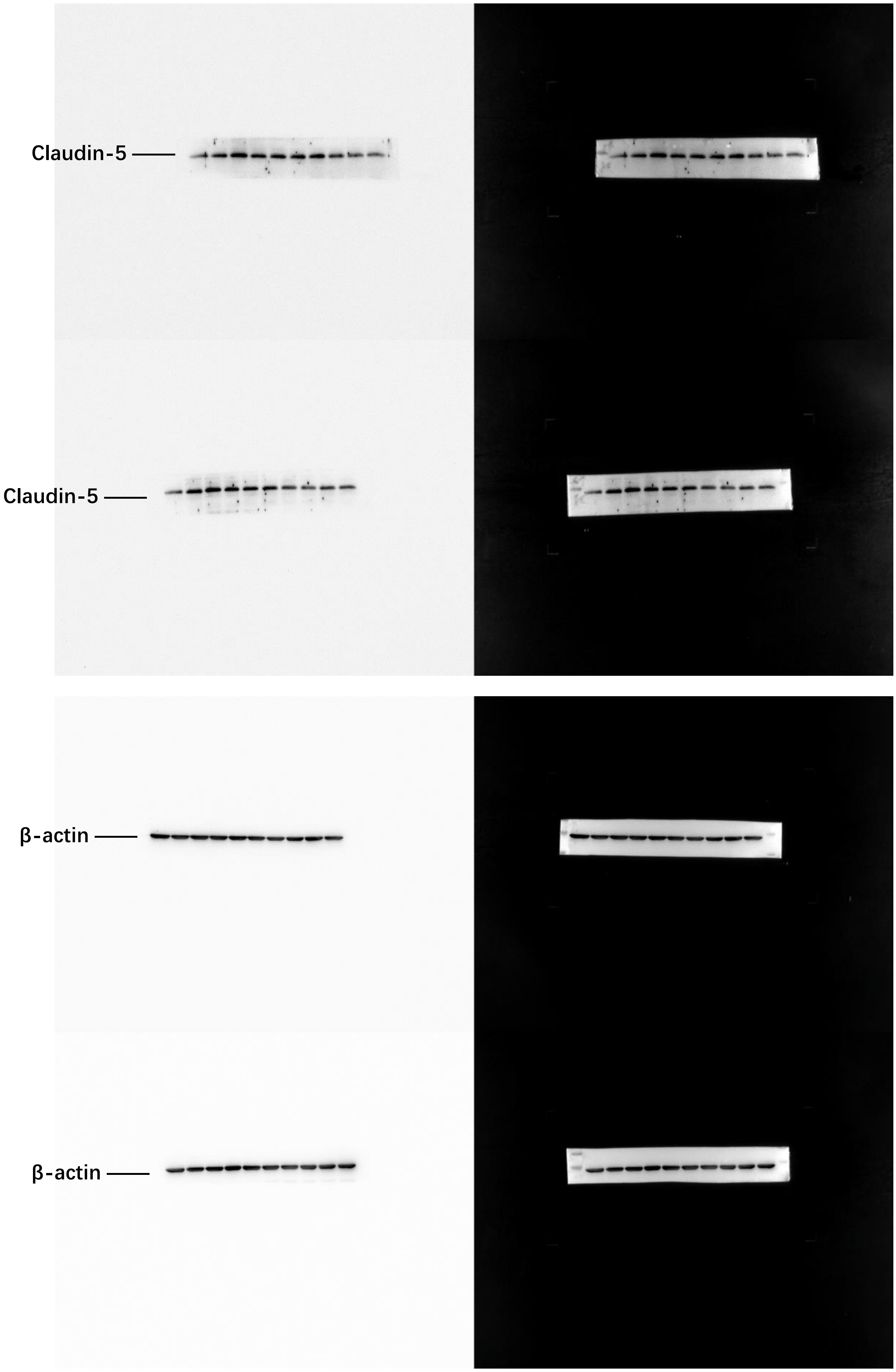

Figure 2I-VE-cadherin

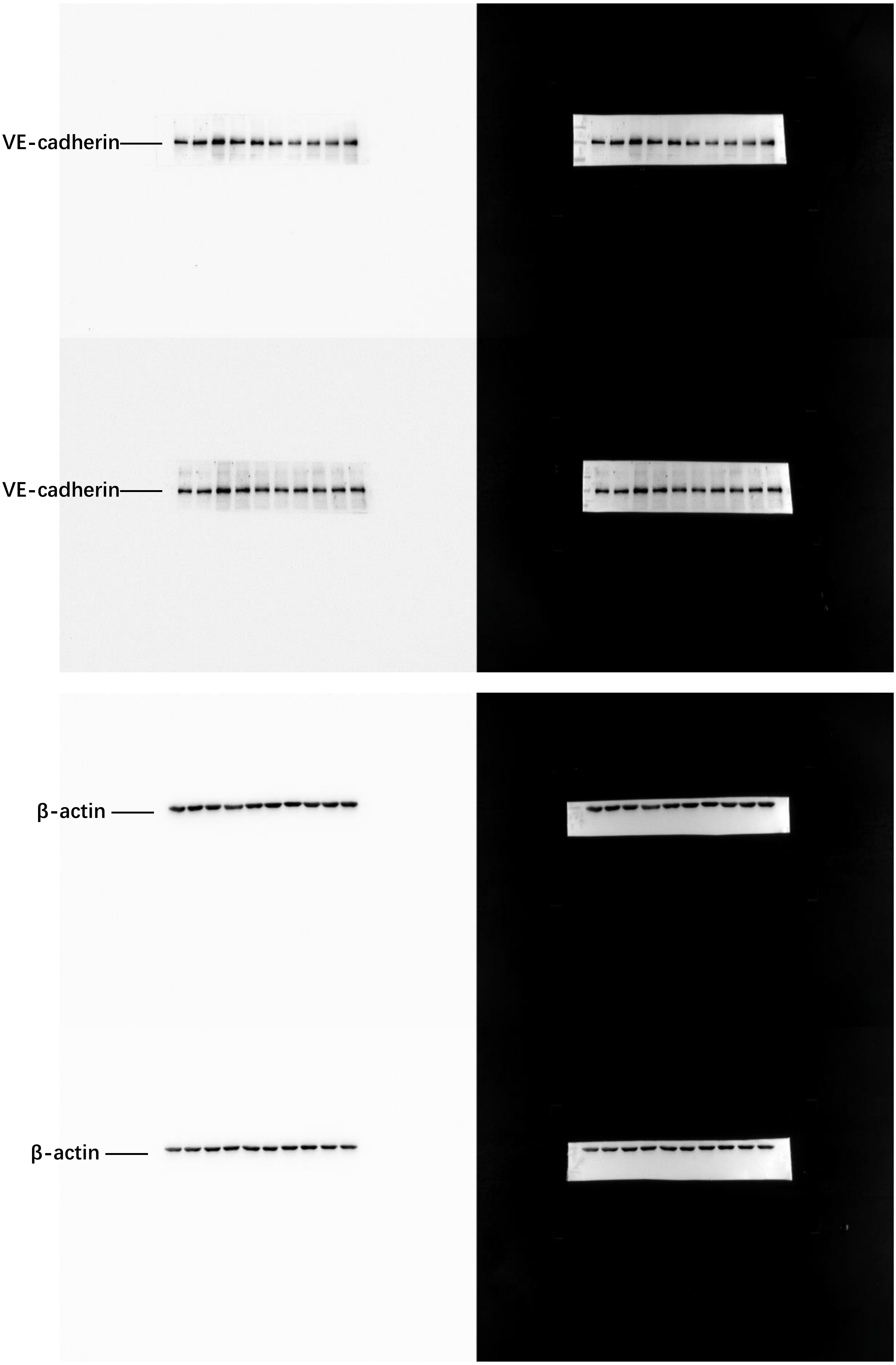

Figure 2J-Claudin-5

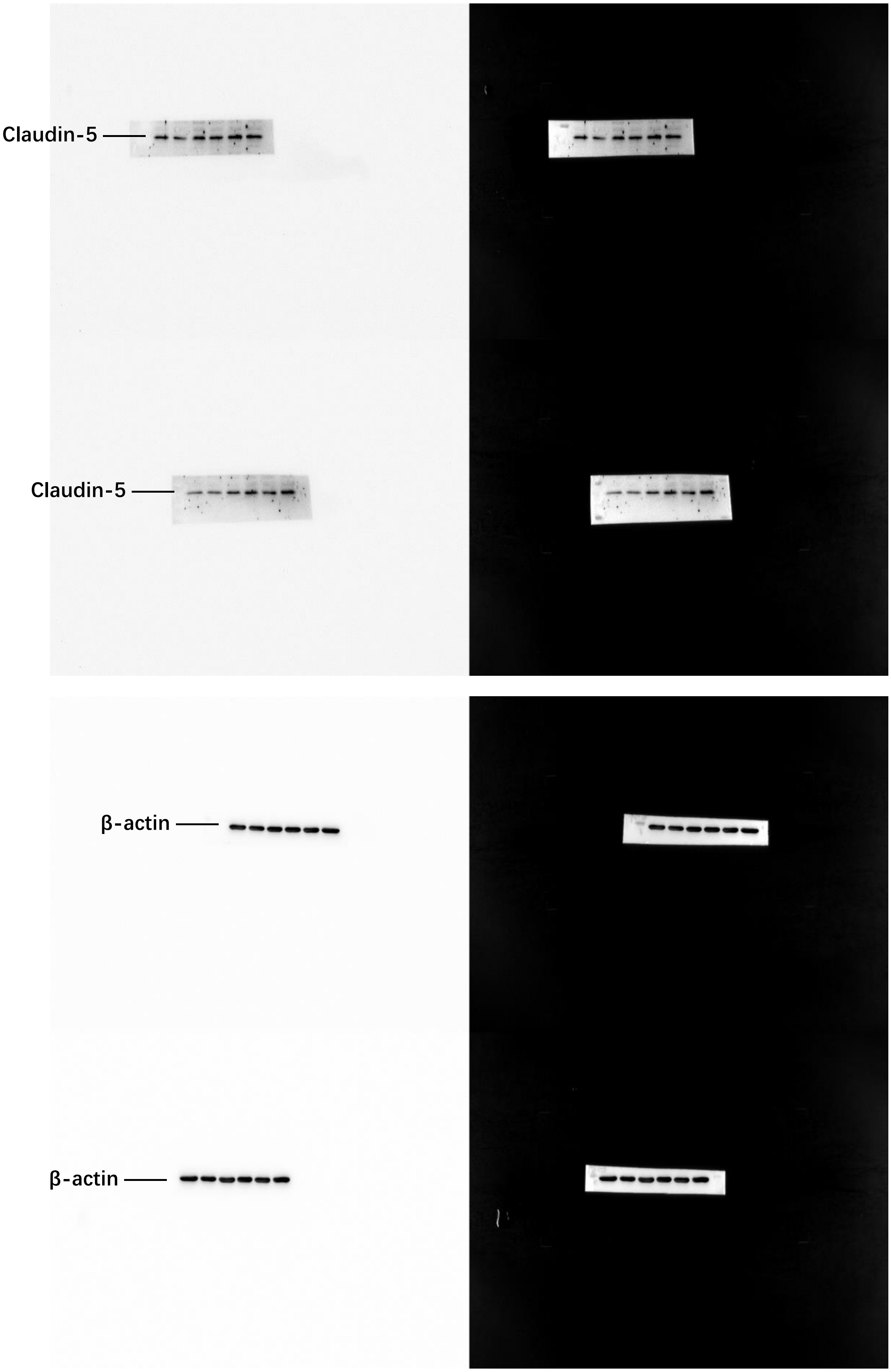

Figure 2J-VE-cadherin

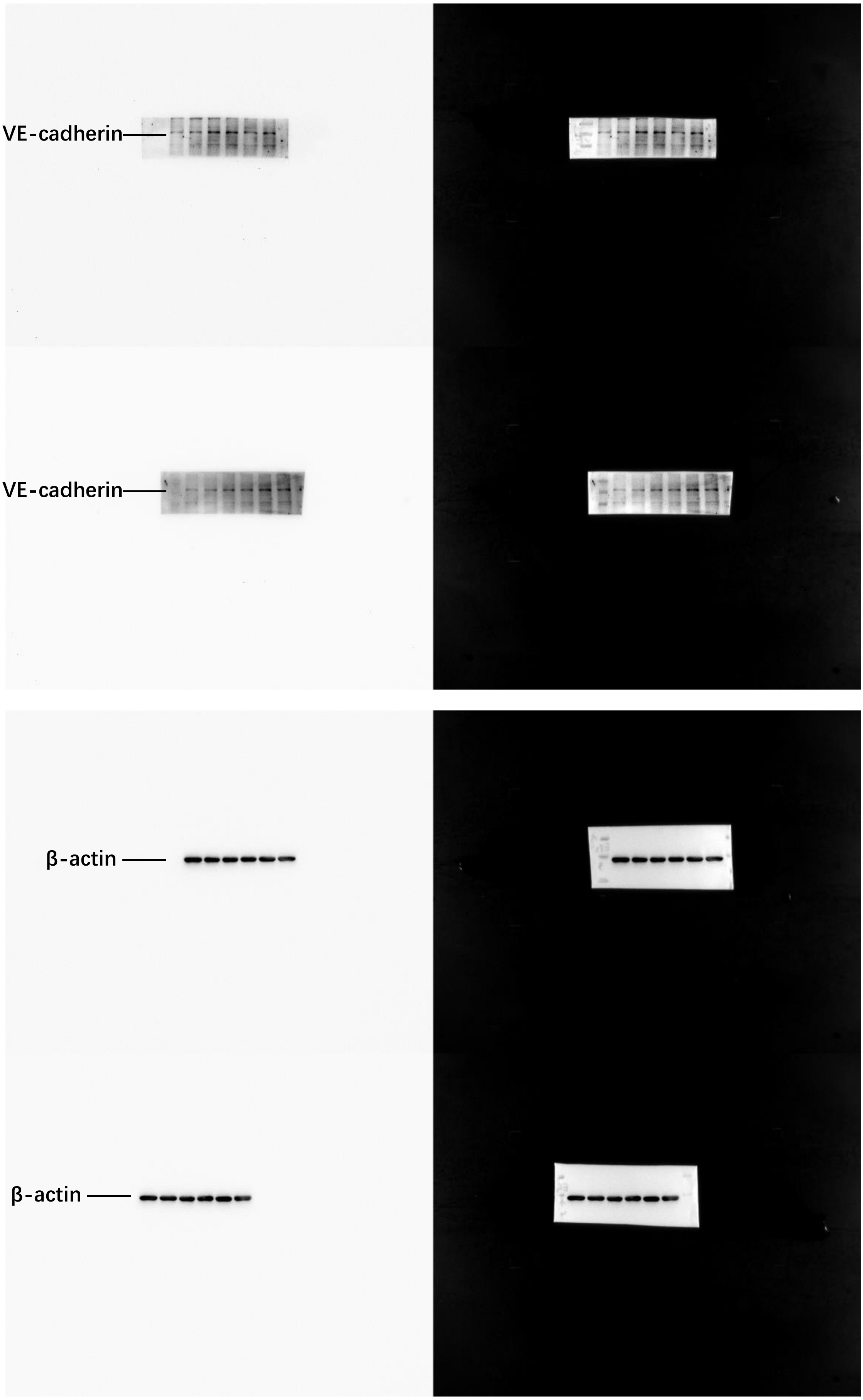

Figure 2K-Claudin-5

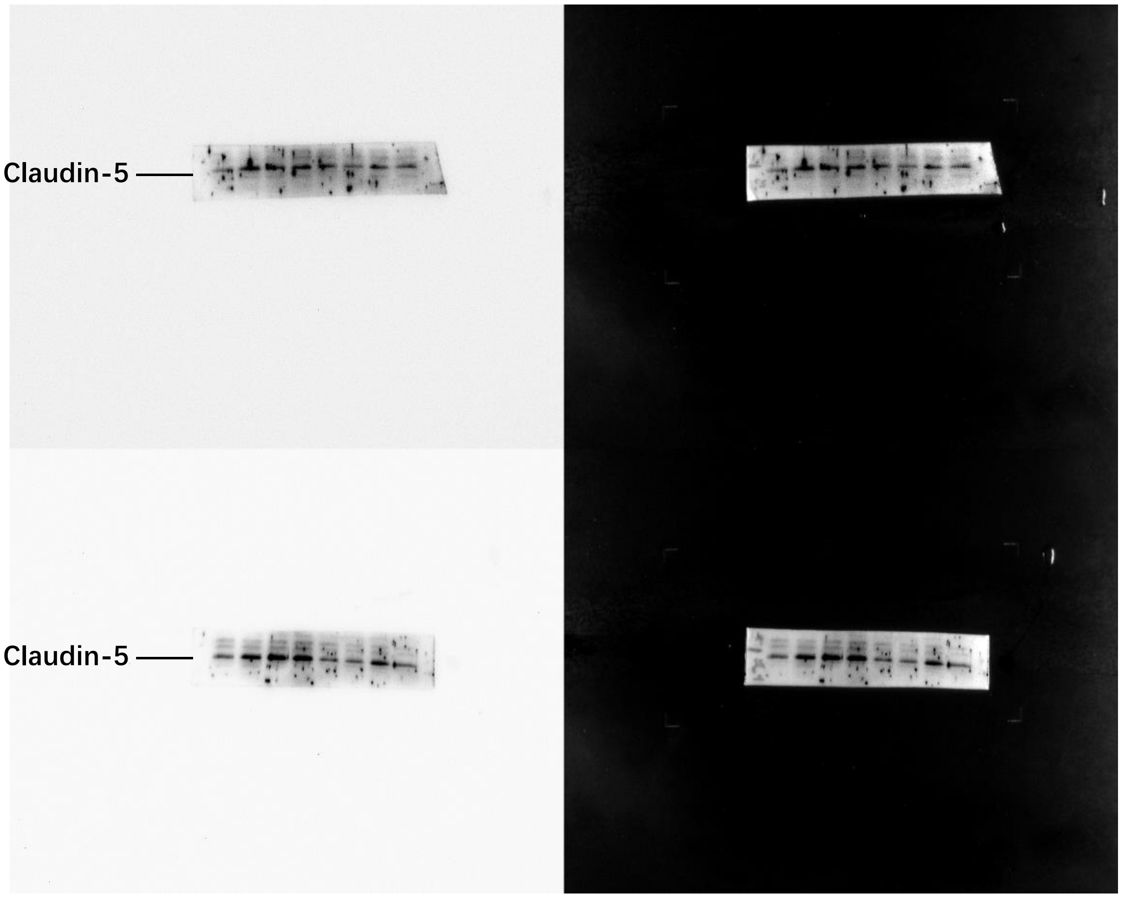

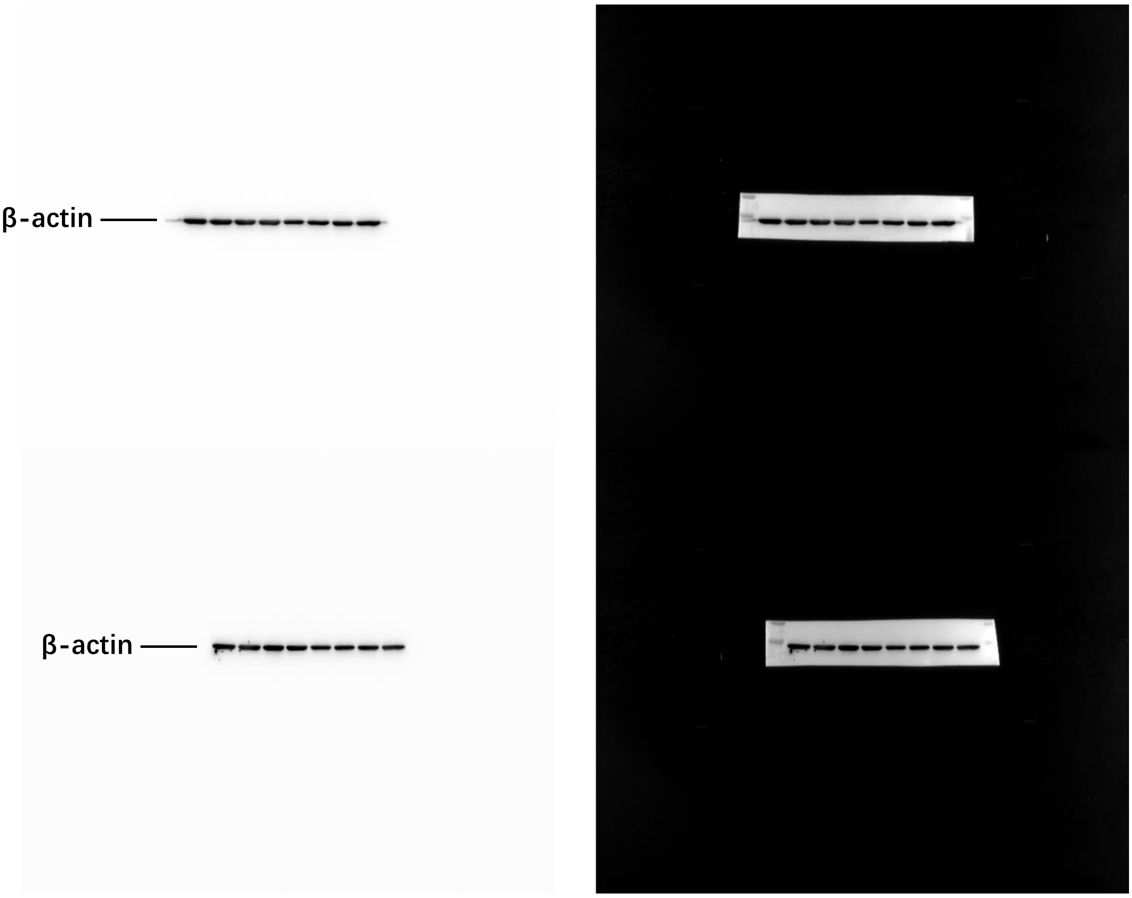

Figure 2K-VE-cadherin

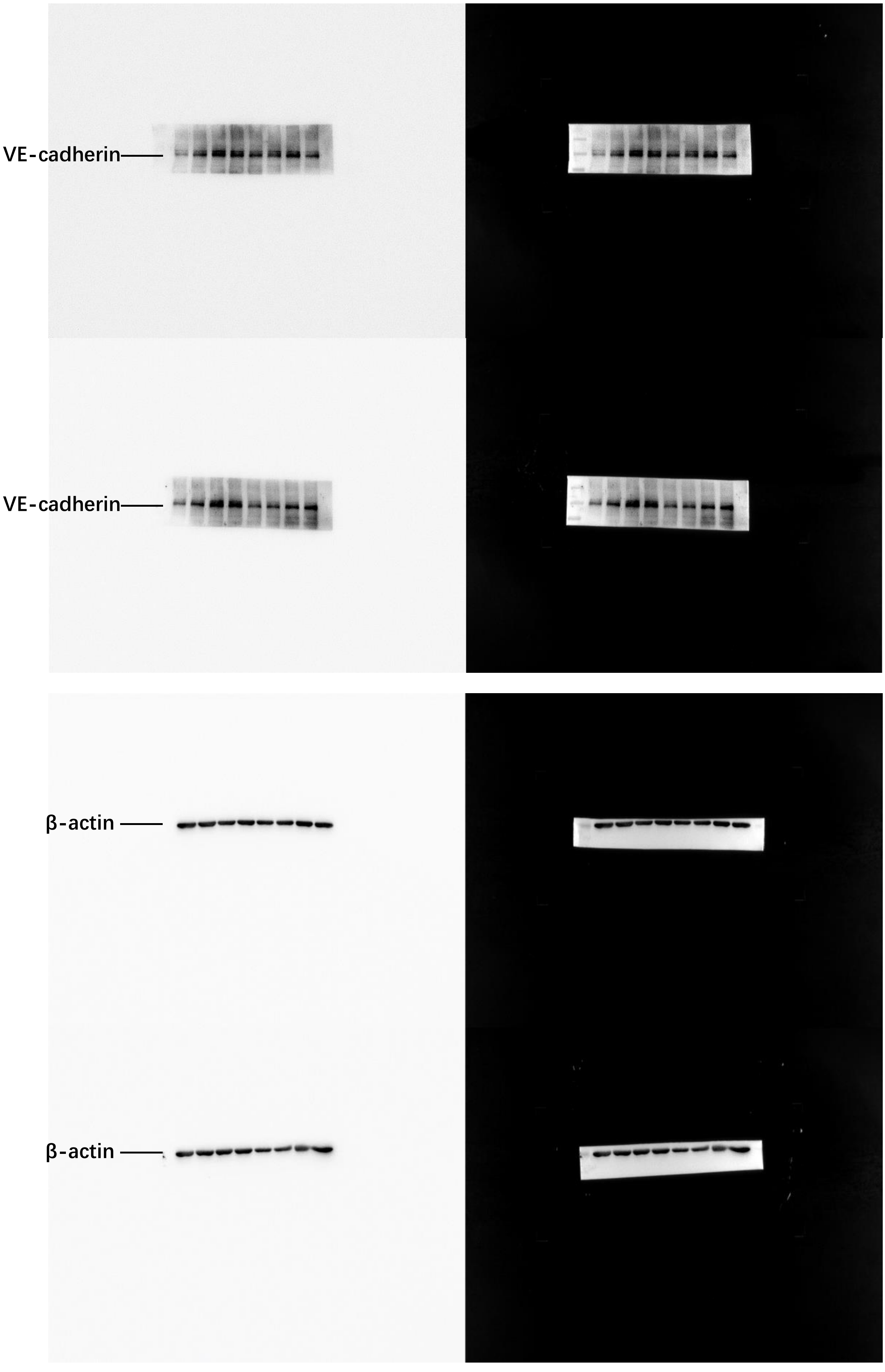

Figure 2L-Claudin-5

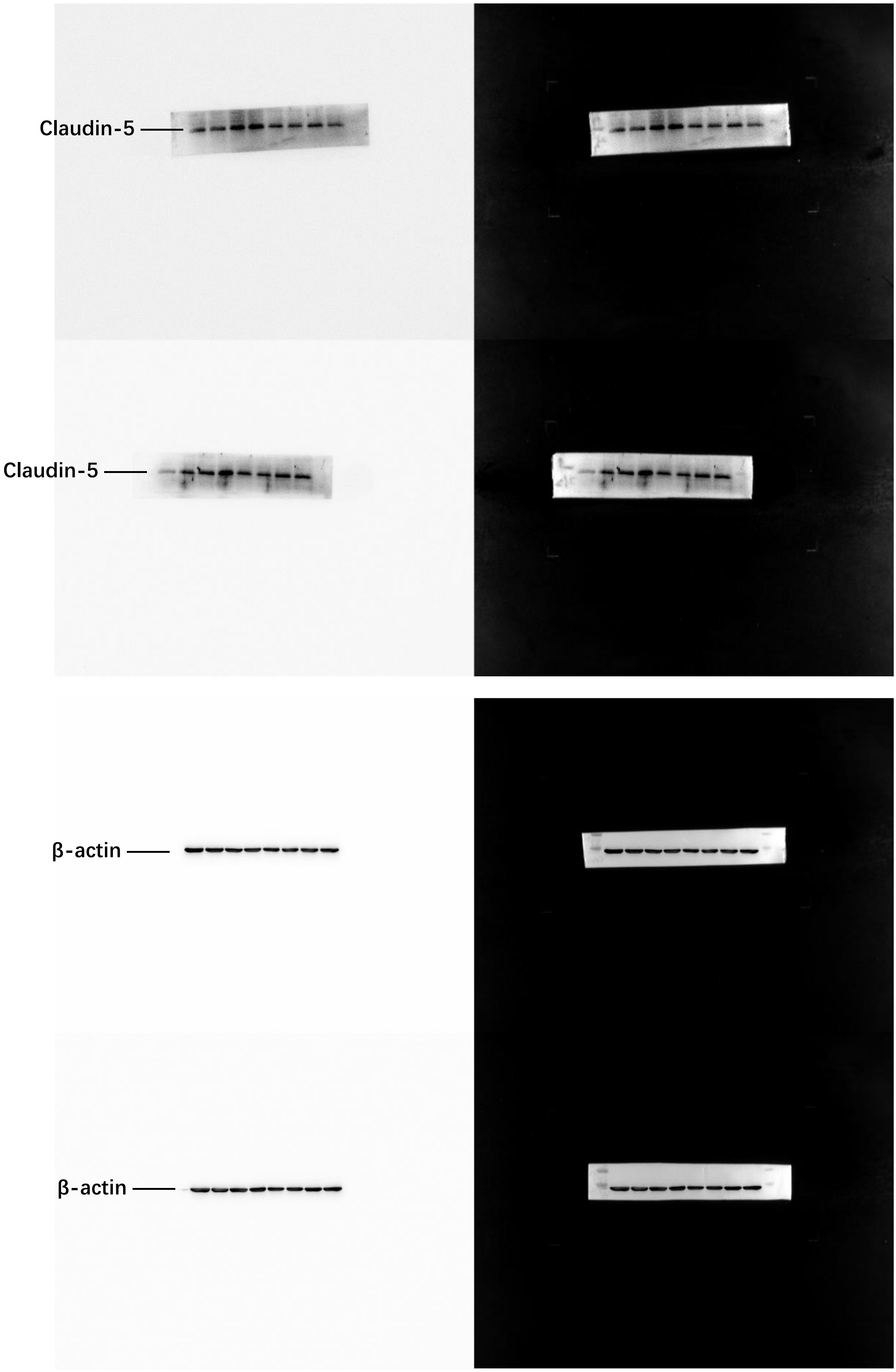

Figure 2L-VE-cadherin

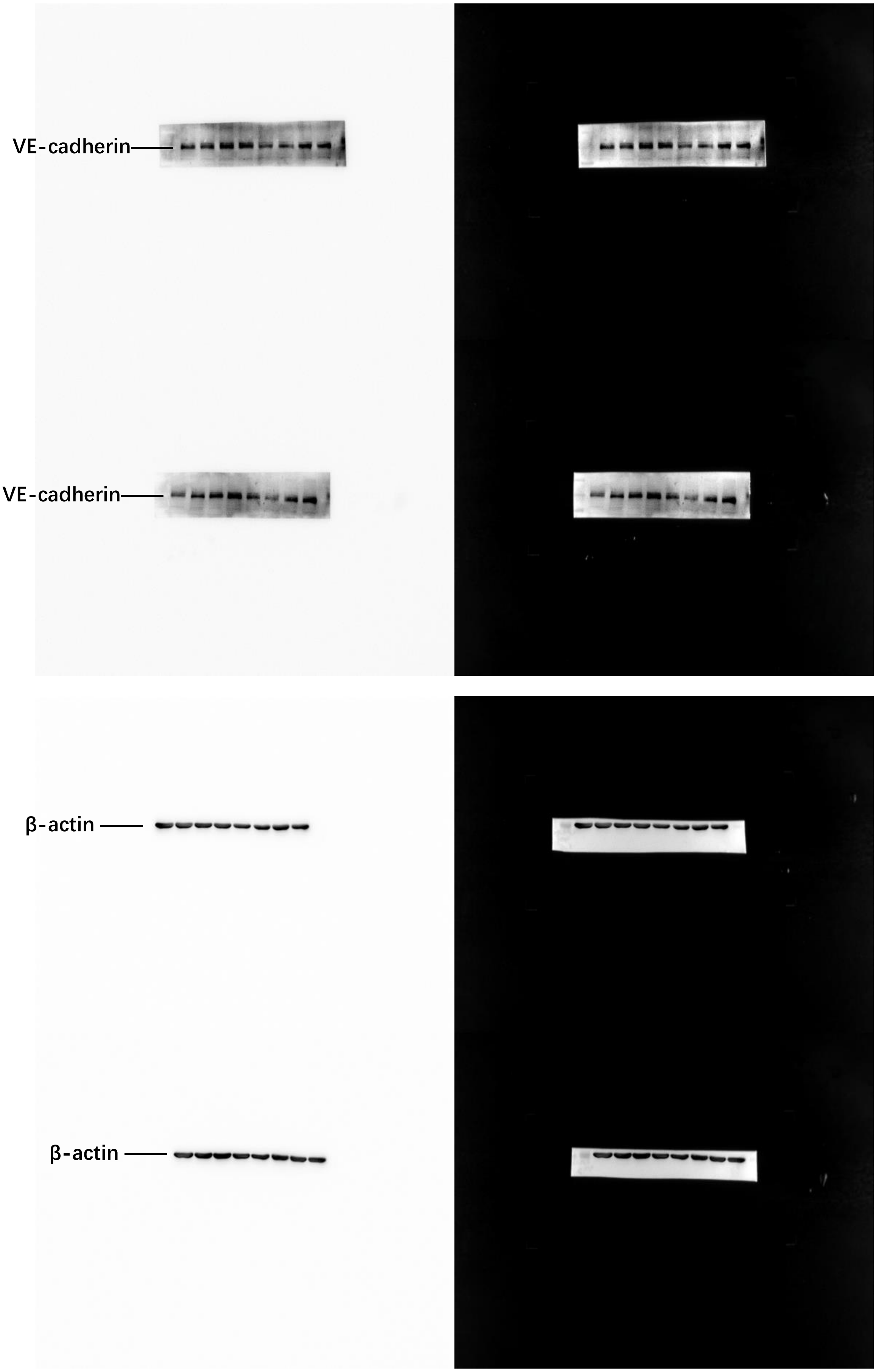

Supplement: Figure 2—source data 2. [file elife-96161-fig2-data2.zip › Figure 2-Source data2/Figure 2-Annotated western blots.pdf]
