# Supplementary figures and images for: Neurons enhance blood–brain barrier function via upregulating claudin-5 and VE-cadherin expression due to glial cell line-derived neurotrophic factor secretion (part 2 of 2)
Source: eLife. 2024 Oct 30;13:RP96161. doi: 10.7554/eLife.96161 (PMC11524583; doi:10.7554/eLife.96161)

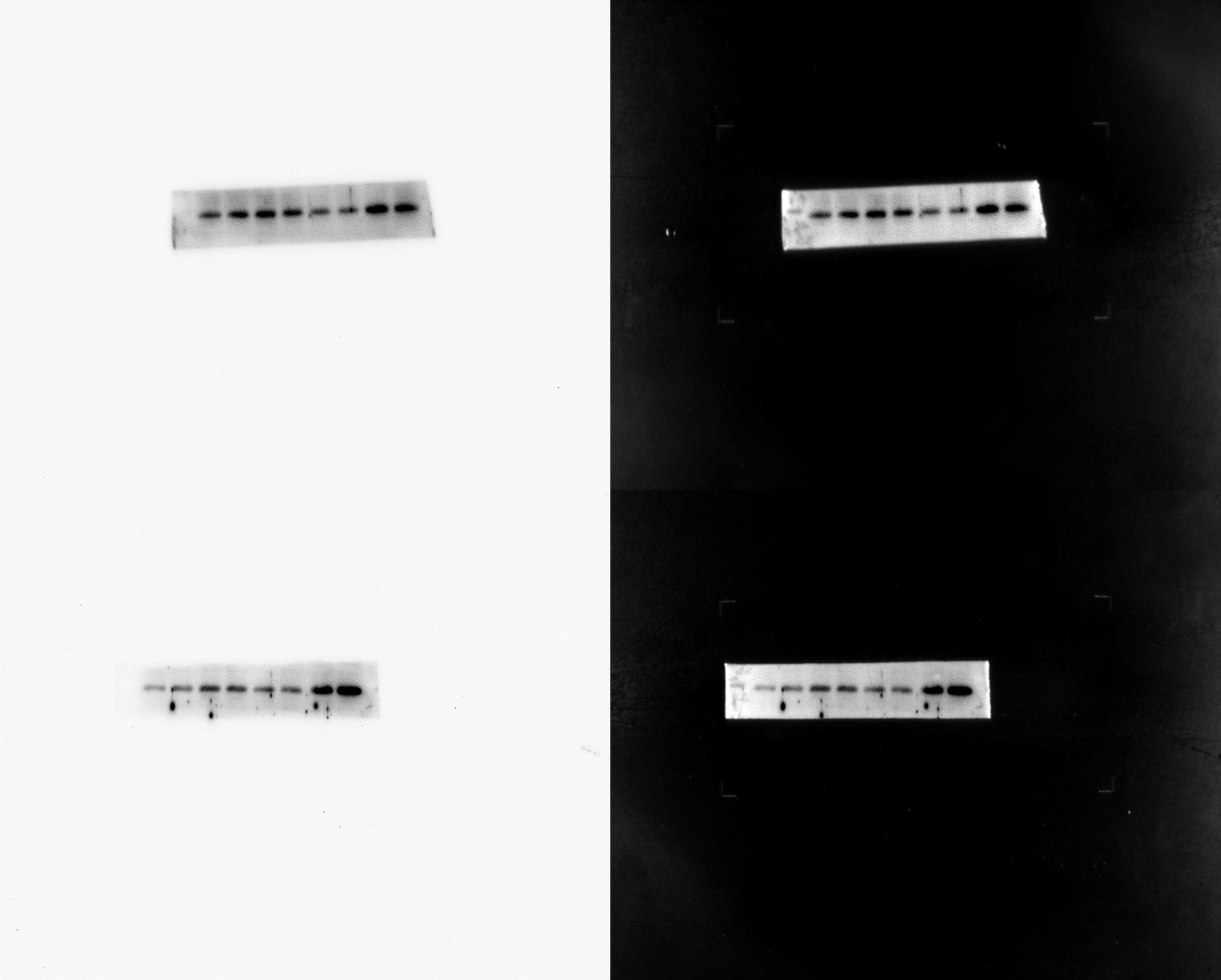

Supplement: Figure 3—source data 1. [file elife-96161-fig3-data1.zip › Figure 3-Source data1/Figure3I-Source data1-Claudin-5.png]

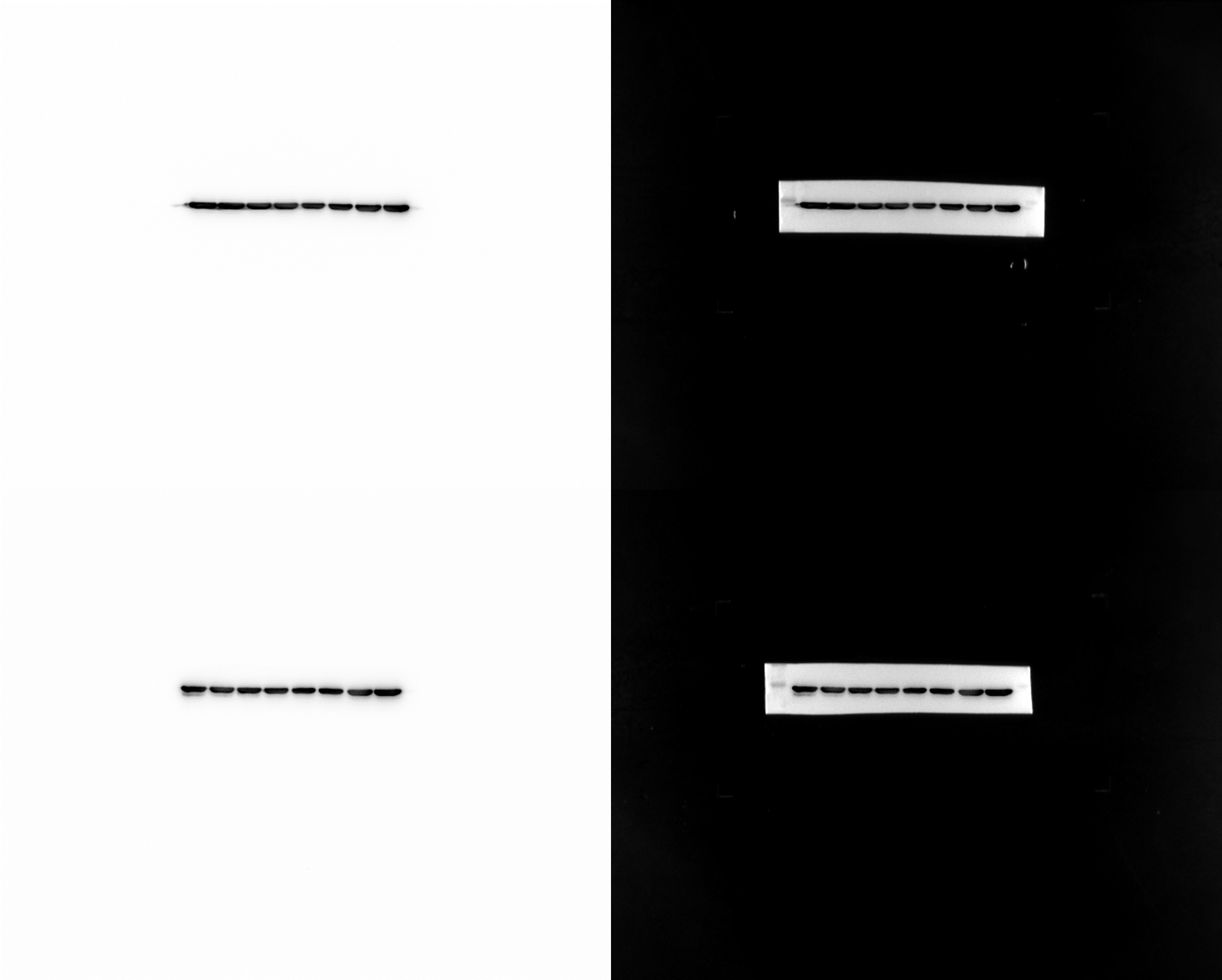

Supplement: Figure 3—source data 1. [file elife-96161-fig3-data1.zip › Figure 3-Source data1/Figure3I-Source data1-a┬-actin.png]

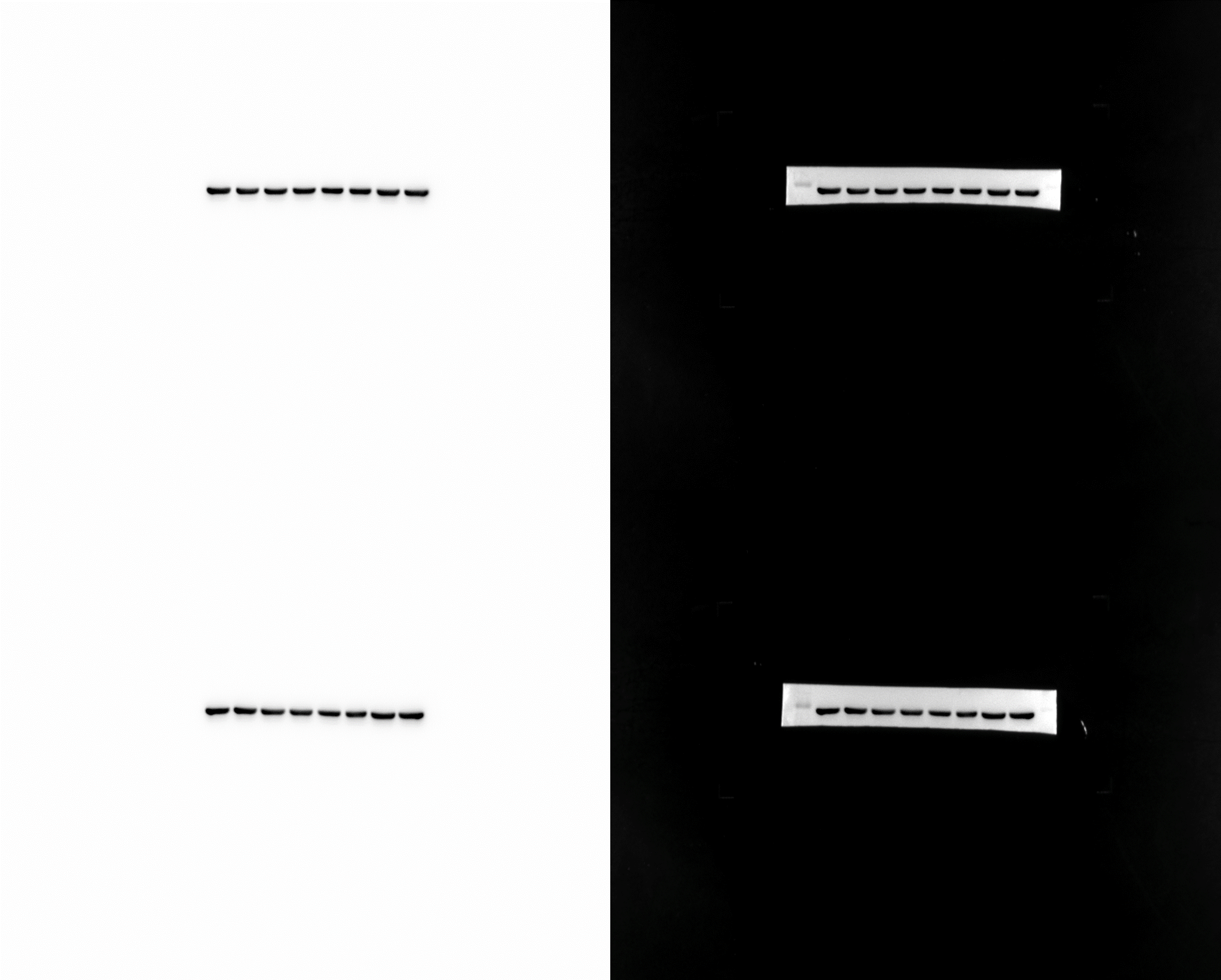

Supplement: Figure 3—source data 1. [file elife-96161-fig3-data1.zip › Figure 3-Source data1/Figure3I-Source data2-Tubulin.png]

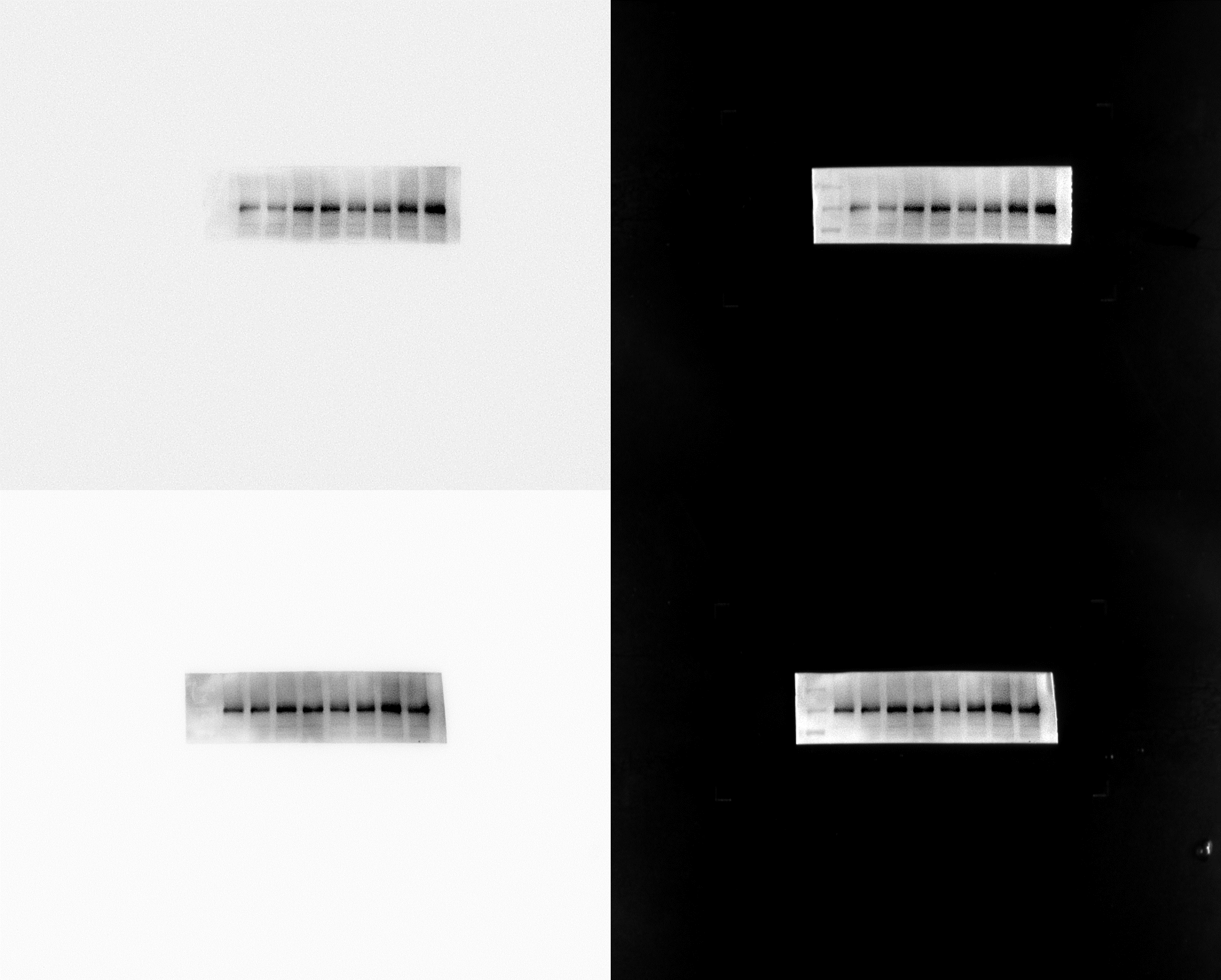

Supplement: Figure 3—source data 1. [file elife-96161-fig3-data1.zip › Figure 3-Source data1/Figure3I-Source data2-VE-Cadherin.png]

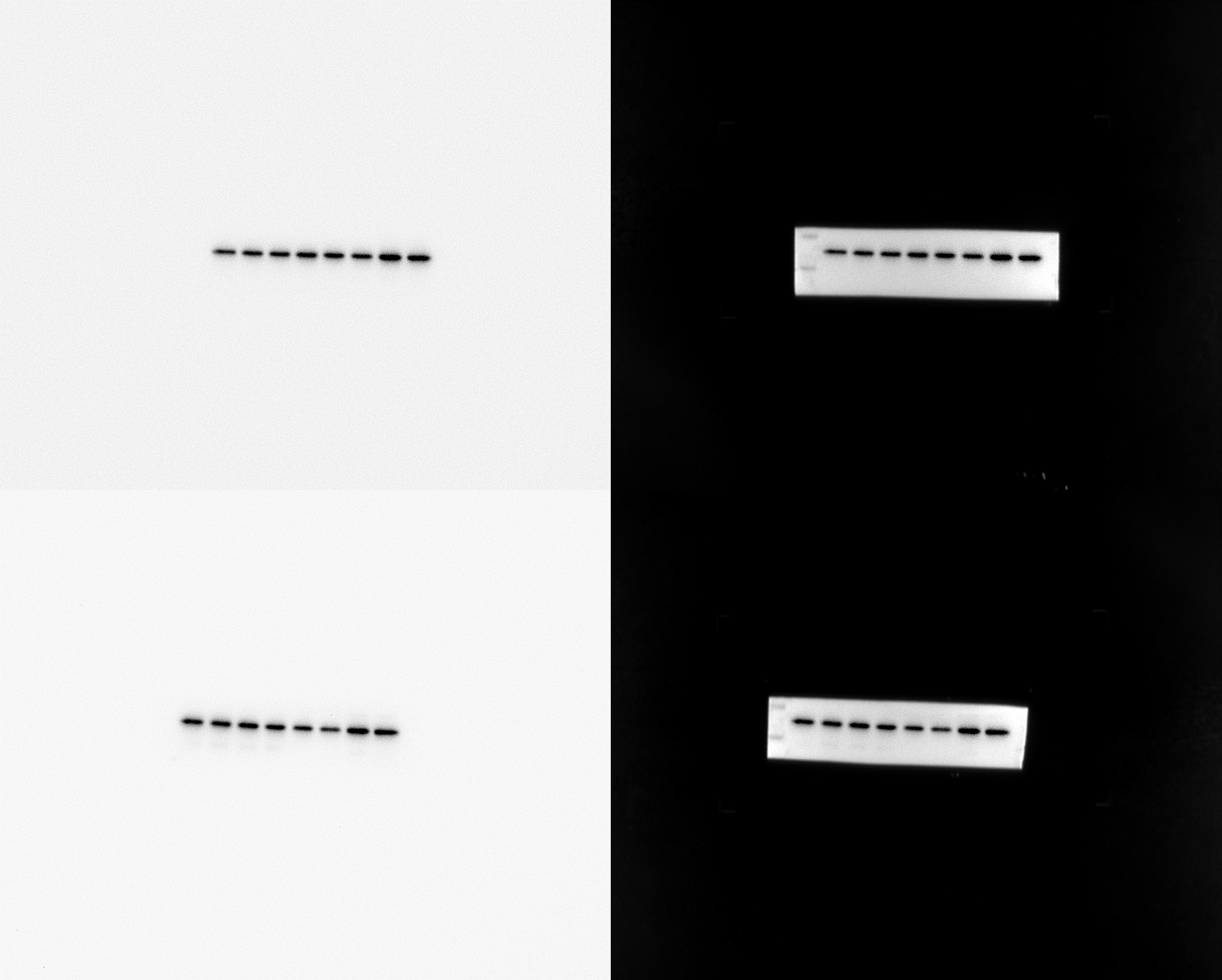

Supplement: Figure 3—source data 1. [file elife-96161-fig3-data1.zip › Figure 3-Source data1/Figure3I-Source data3-p38.png]

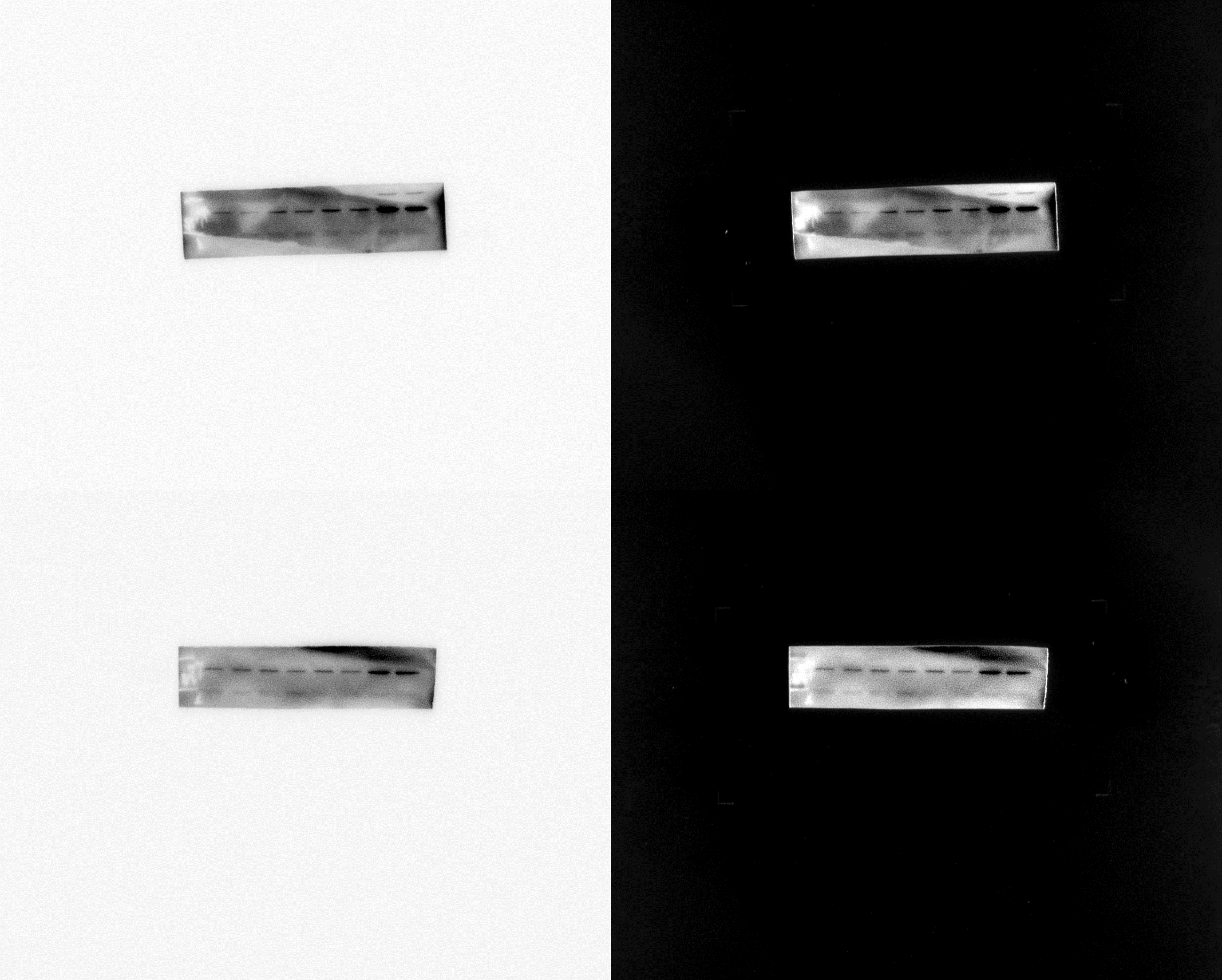

Supplement: Figure 3—source data 1. [file elife-96161-fig3-data1.zip › Figure 3-Source data1/Figure3I-Source data3-p-p38.png]

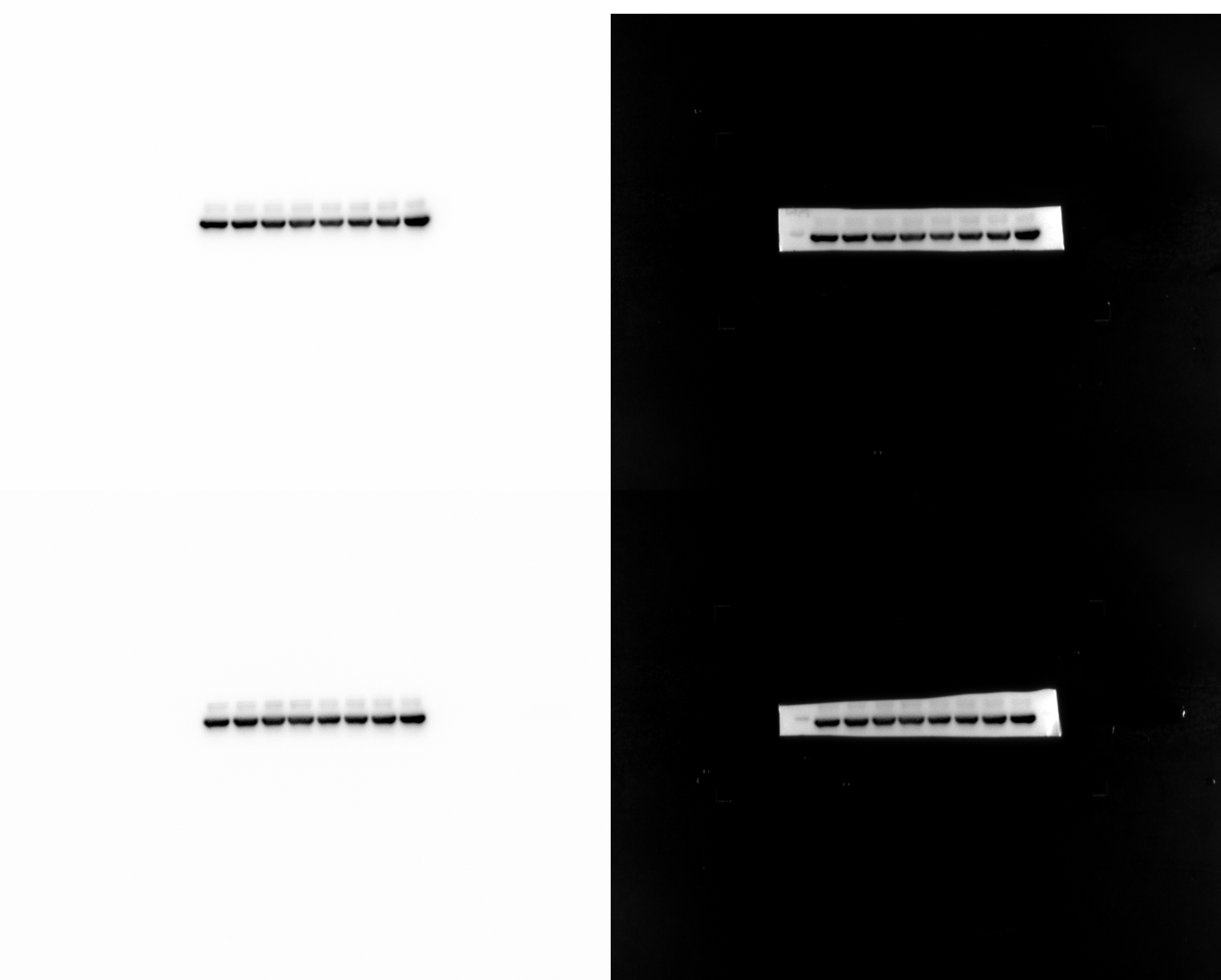

Supplement: Figure 3—source data 1. [file elife-96161-fig3-data1.zip › Figure 3-Source data1/Figure3J-Source data1,2-Tubulin.png]

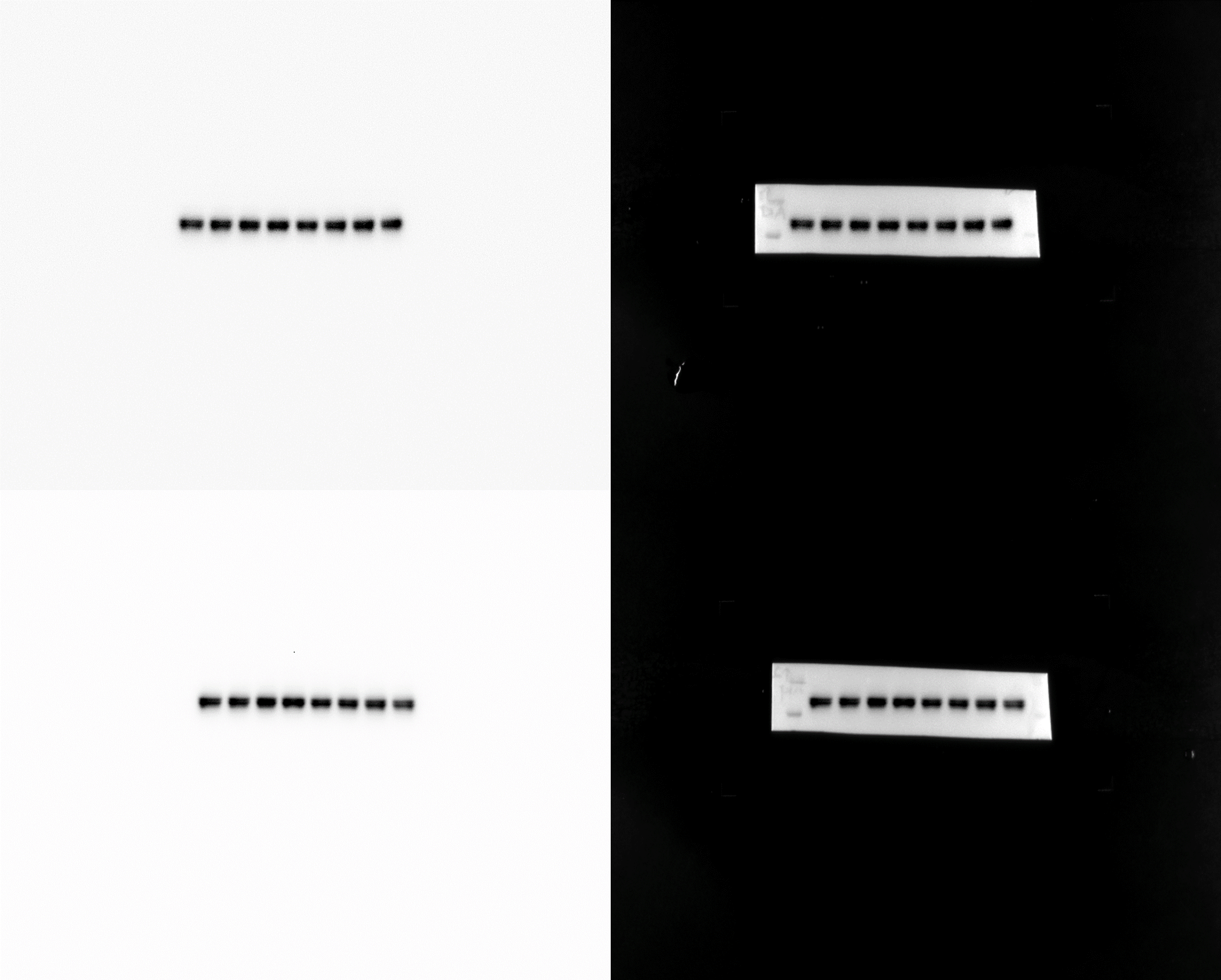

Supplement: Figure 3—source data 1. [file elife-96161-fig3-data1.zip › Figure 3-Source data1/Figure3J-Source data1-AKT.png]

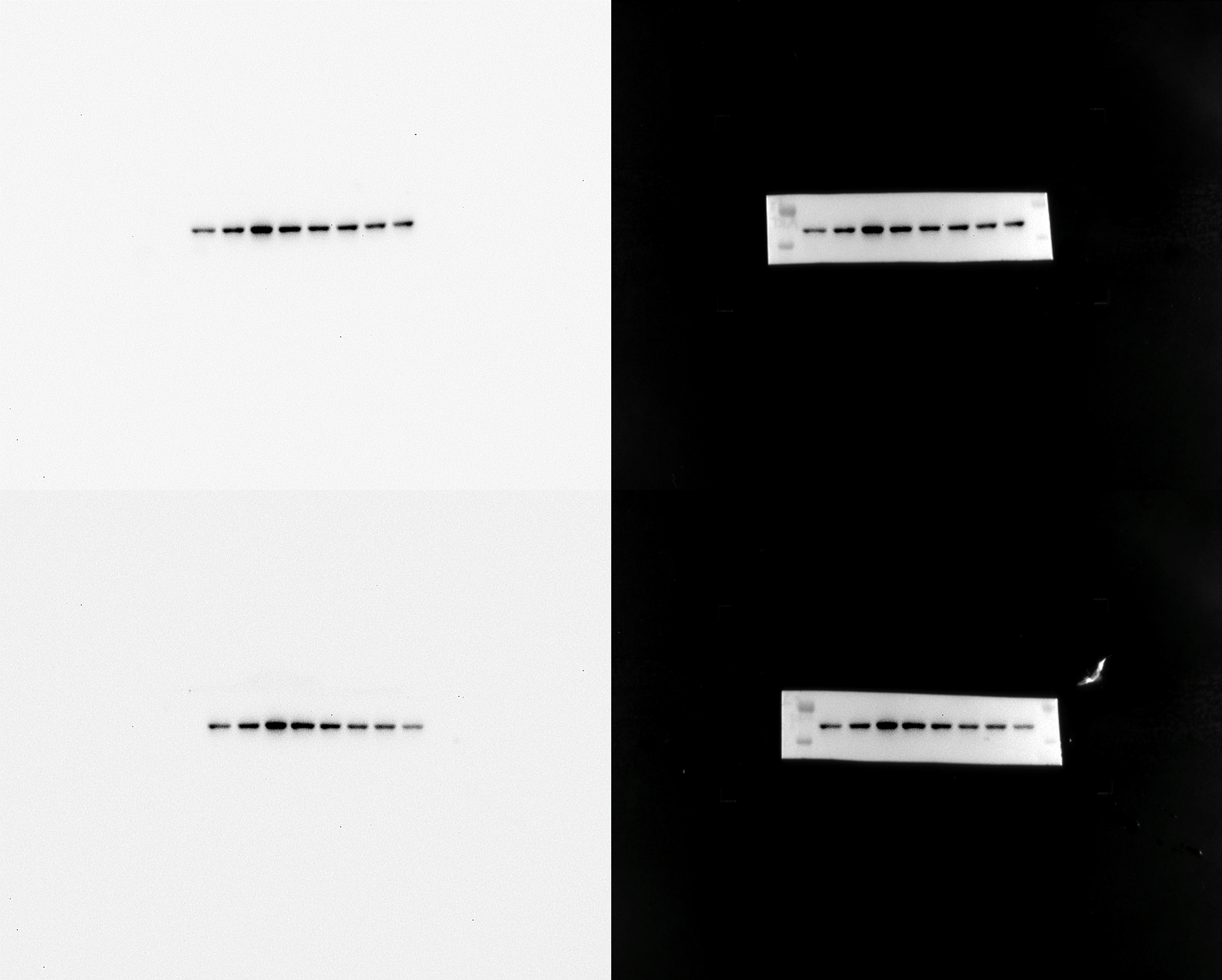

Supplement: Figure 3—source data 1. [file elife-96161-fig3-data1.zip › Figure 3-Source data1/Figure3J-Source data1-p-AKT.png]

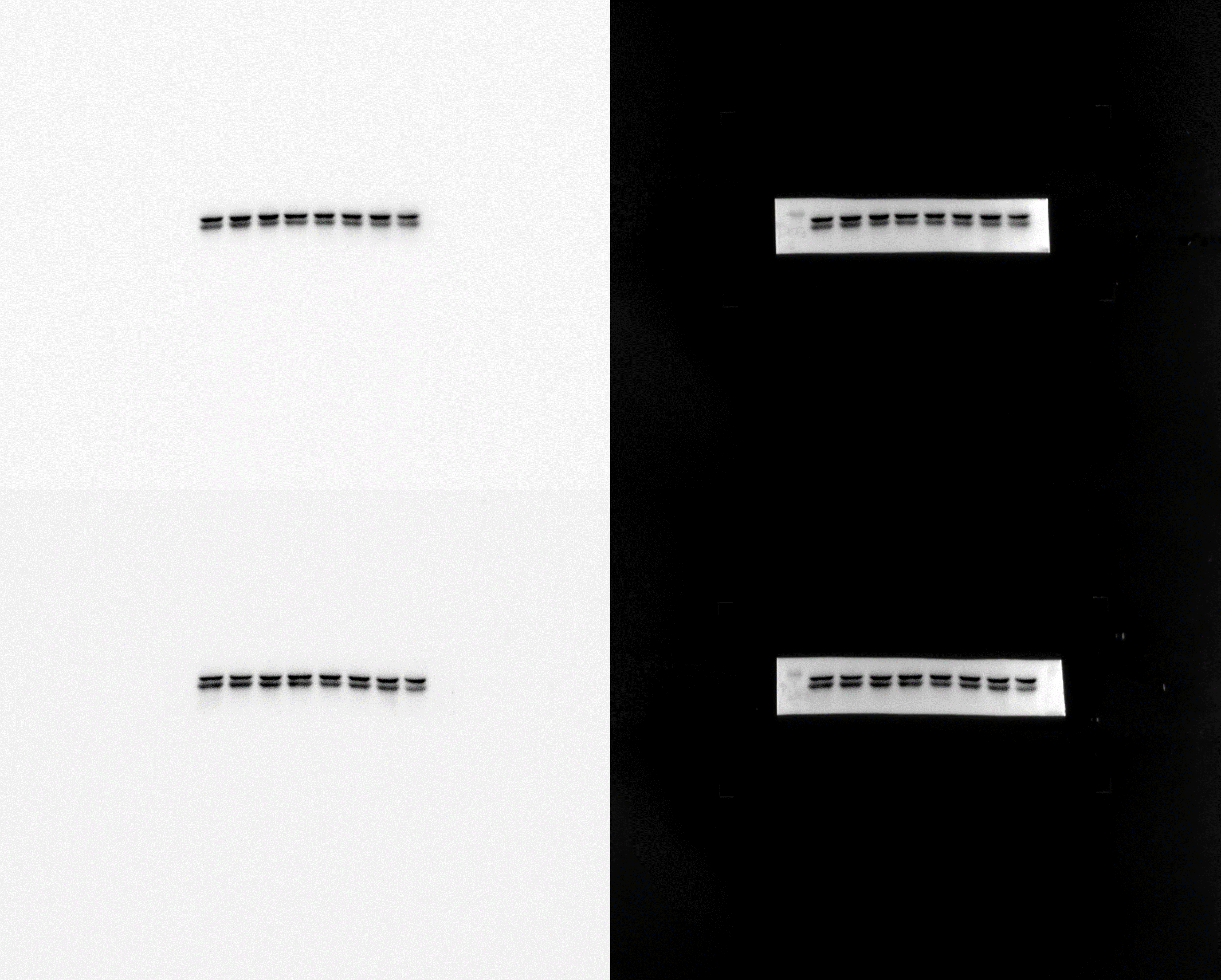

Supplement: Figure 3—source data 1. [file elife-96161-fig3-data1.zip › Figure 3-Source data1/Figure3J-Source data2-ERK.png]

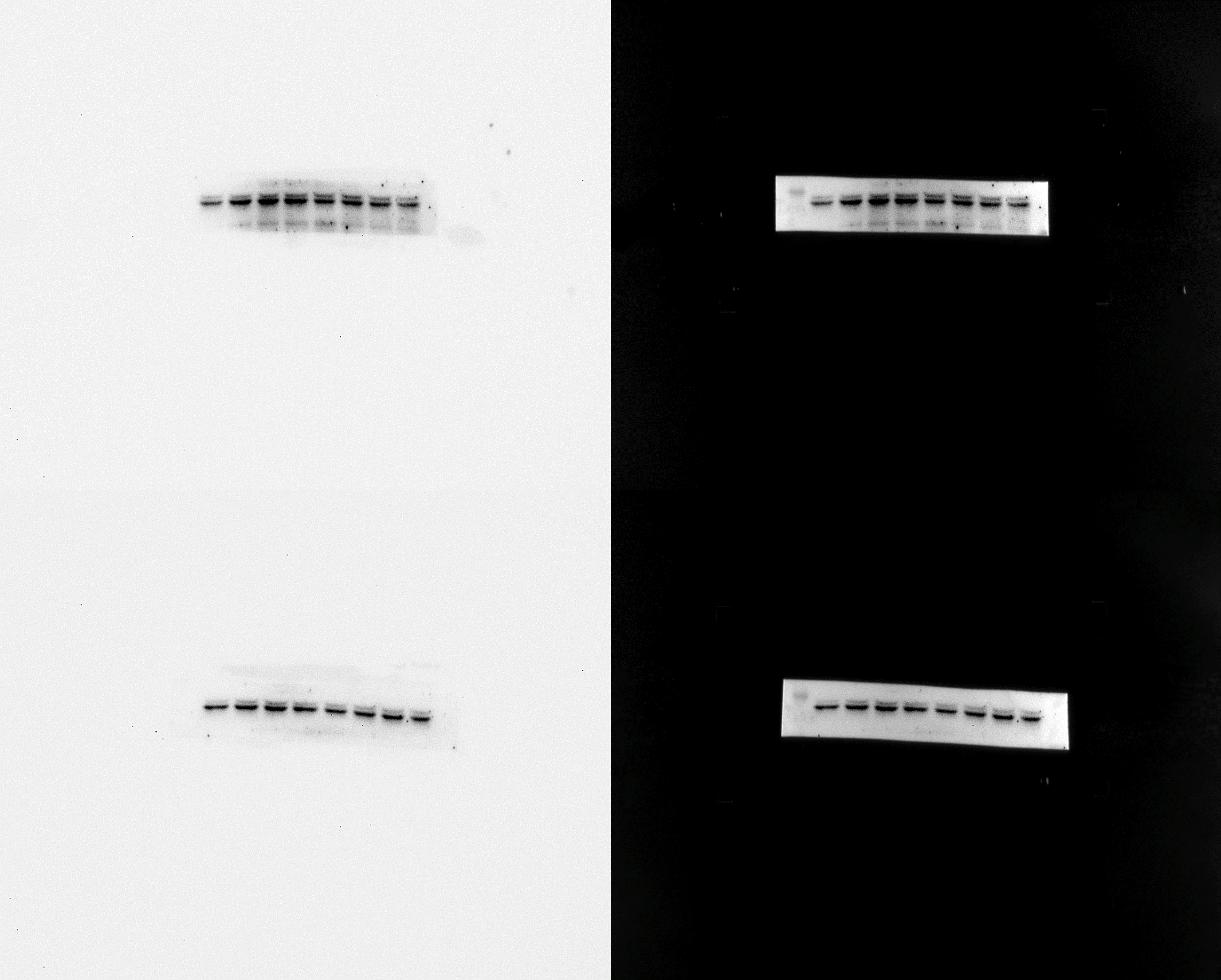

Supplement: Figure 3—source data 1. [file elife-96161-fig3-data1.zip › Figure 3-Source data1/Figure3J-Source data2-p-ERK.png]

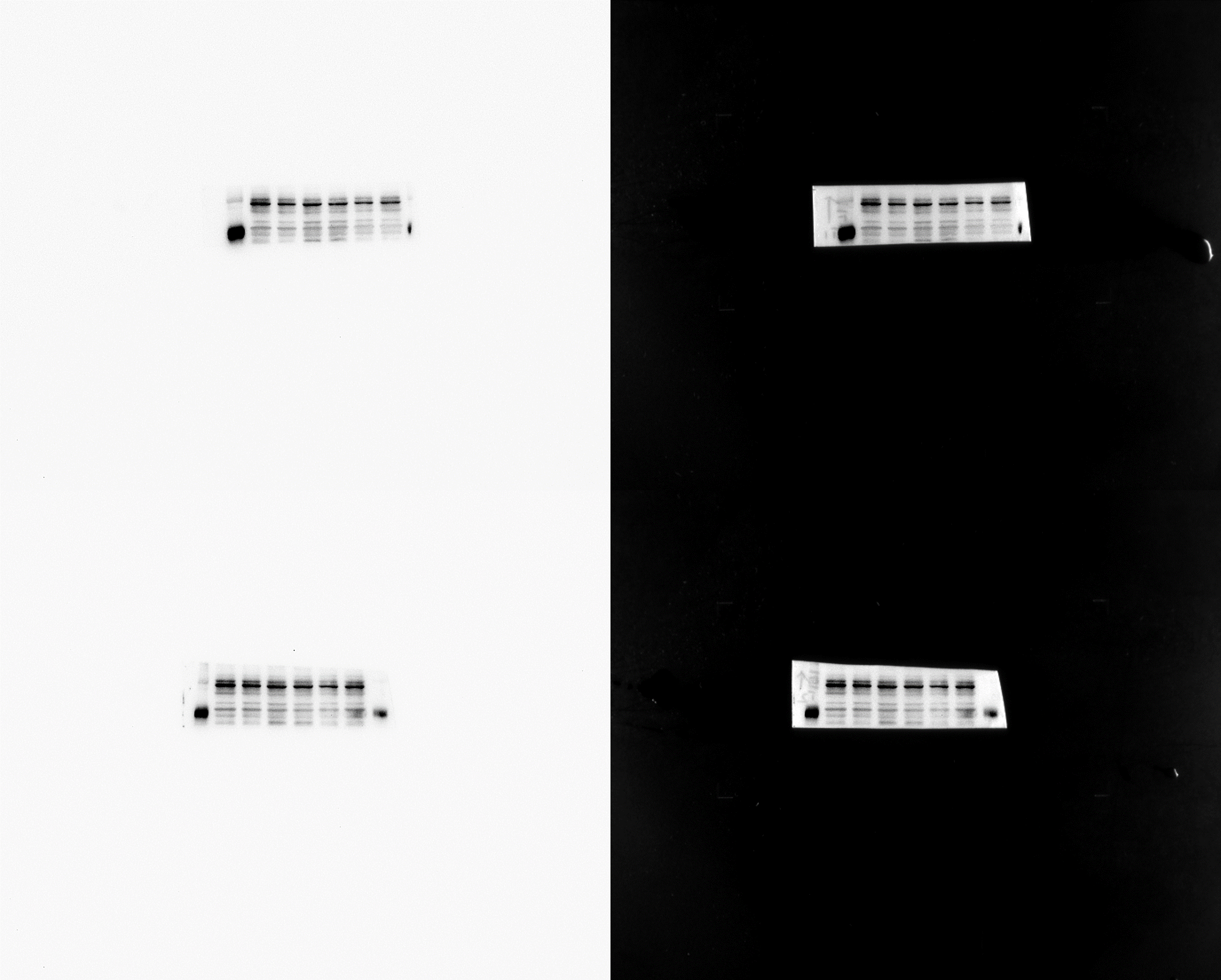

Supplement: Figure 4—source data 1. [file elife-96161-fig4-data1.zip › Figure 4-Source data1/Figure4A-Source data-FOXO1.png]

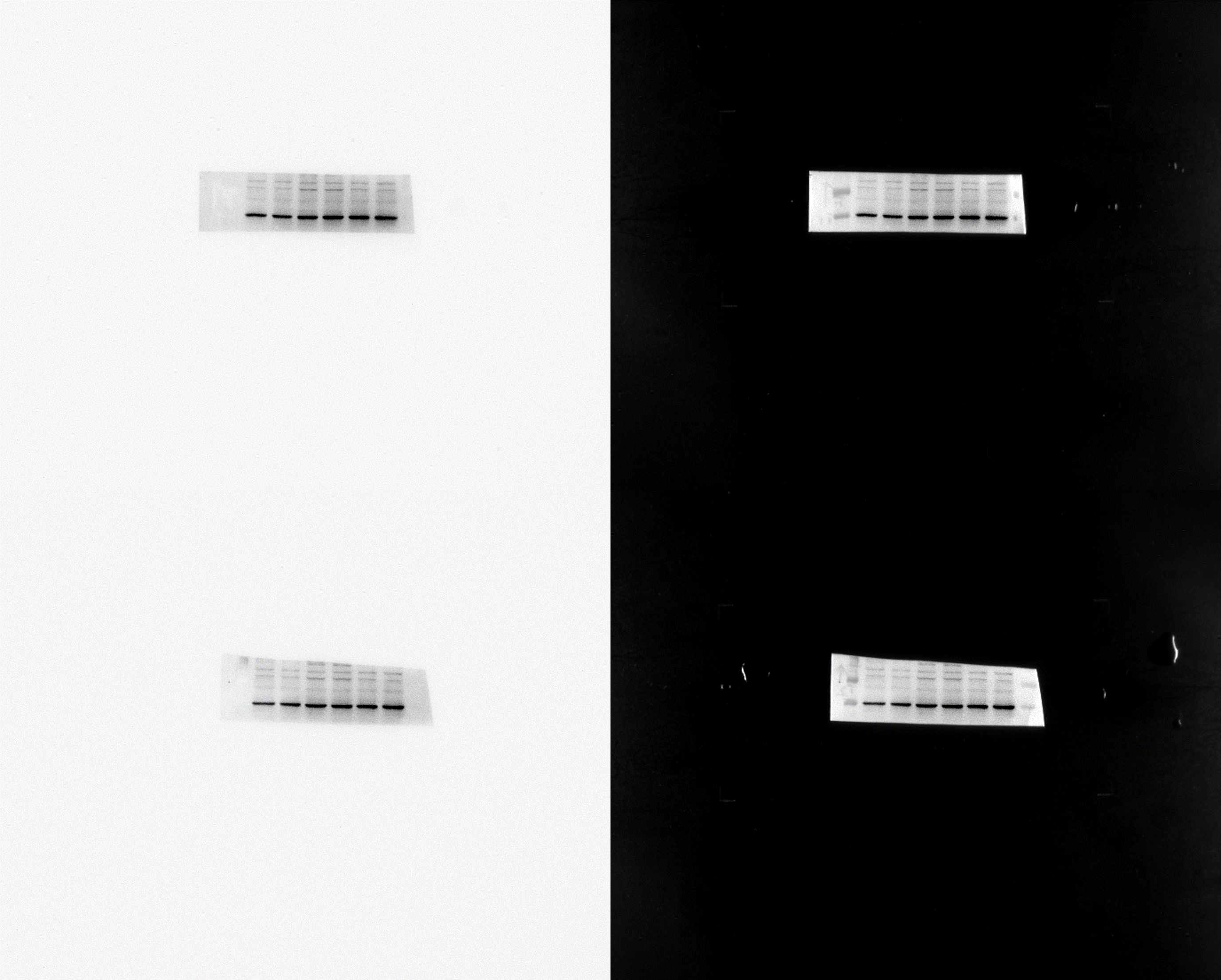

Supplement: Figure 4—source data 1. [file elife-96161-fig4-data1.zip › Figure 4-Source data1/Figure4A-Source data-p-FOXO1.png]

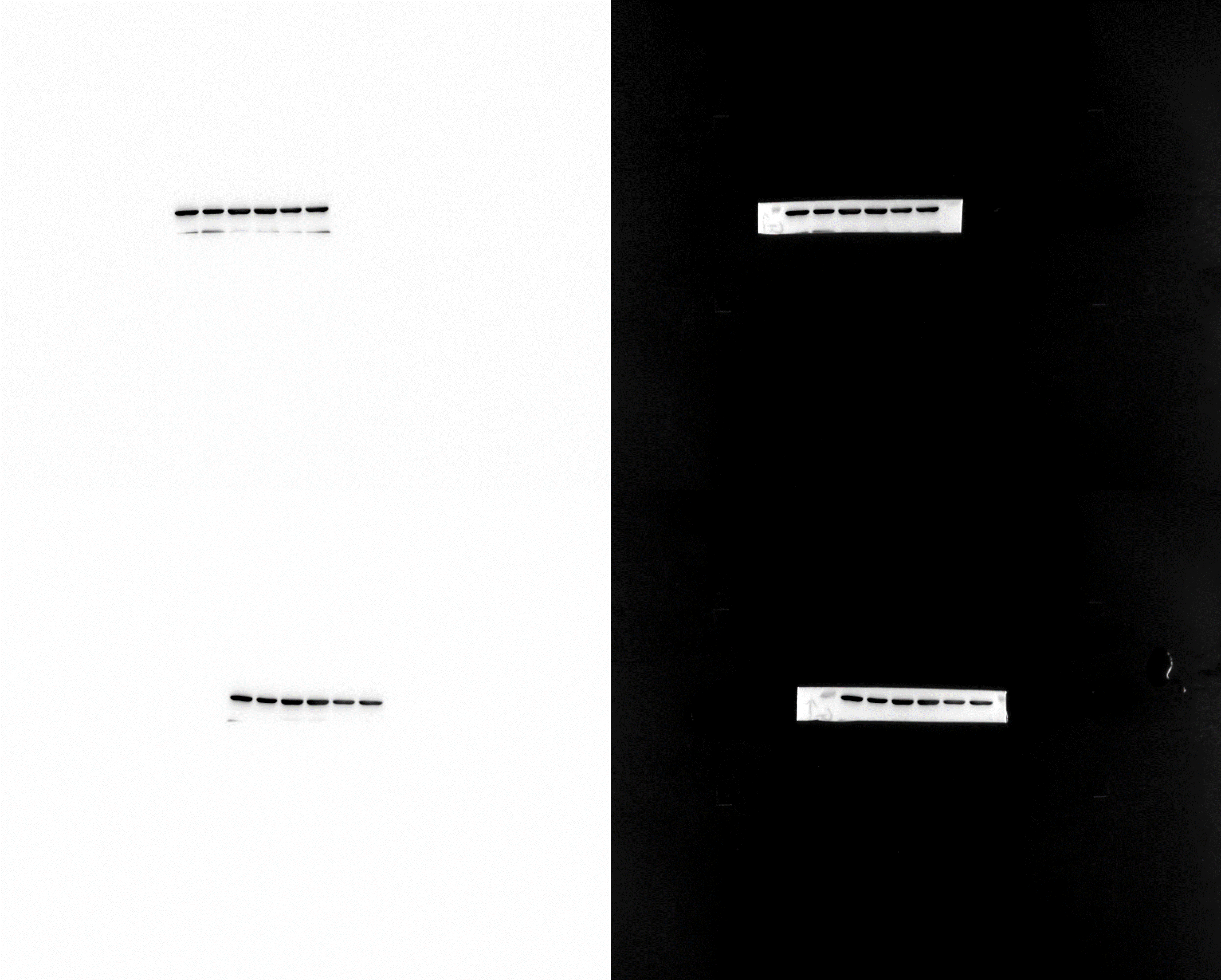

Supplement: Figure 4—source data 1. [file elife-96161-fig4-data1.zip › Figure 4-Source data1/Figure4A-Source data-a┬-actin.png]

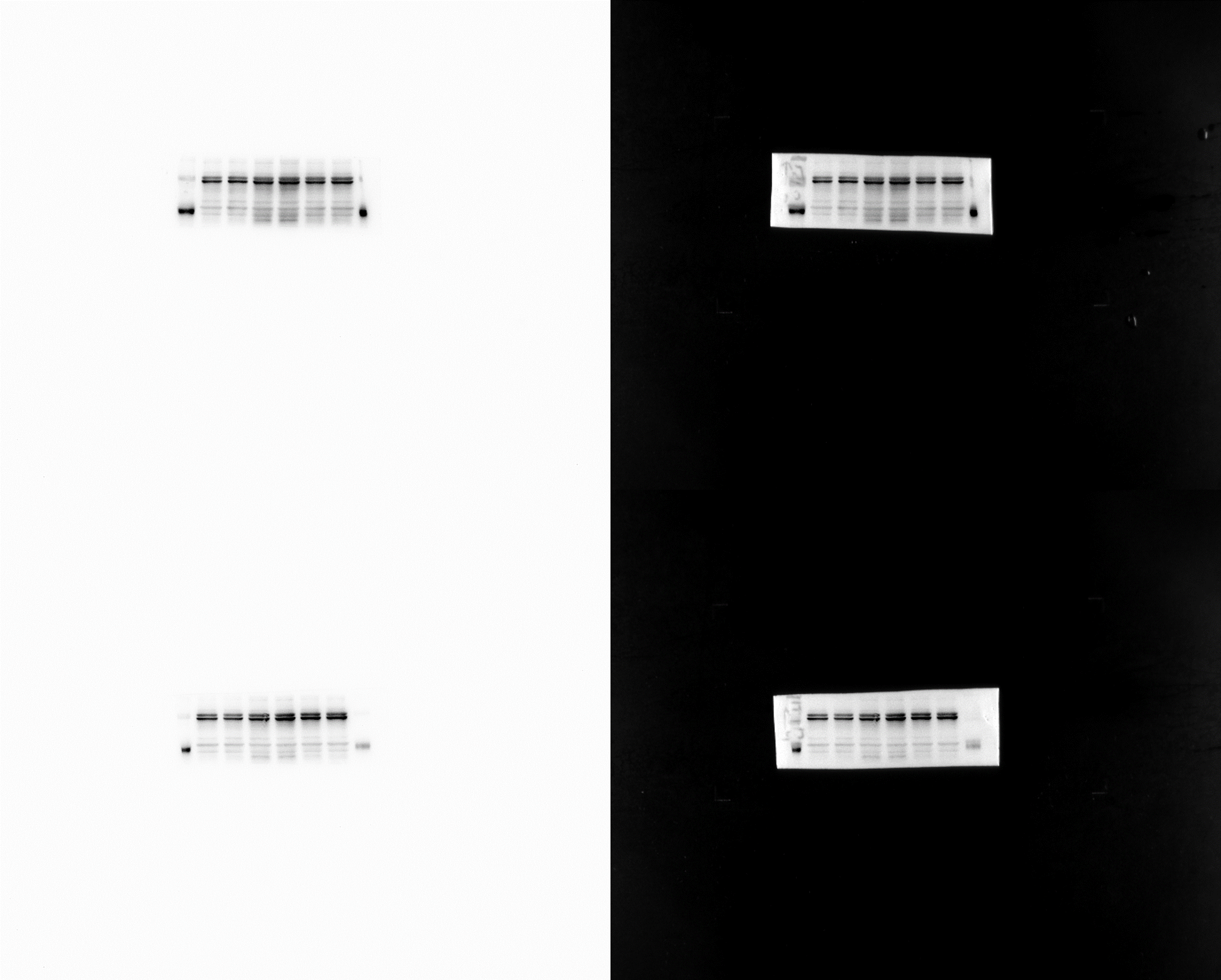

Supplement: Figure 4—source data 1. [file elife-96161-fig4-data1.zip › Figure 4-Source data1/Figure4B-Source data1-FOXO1.png]

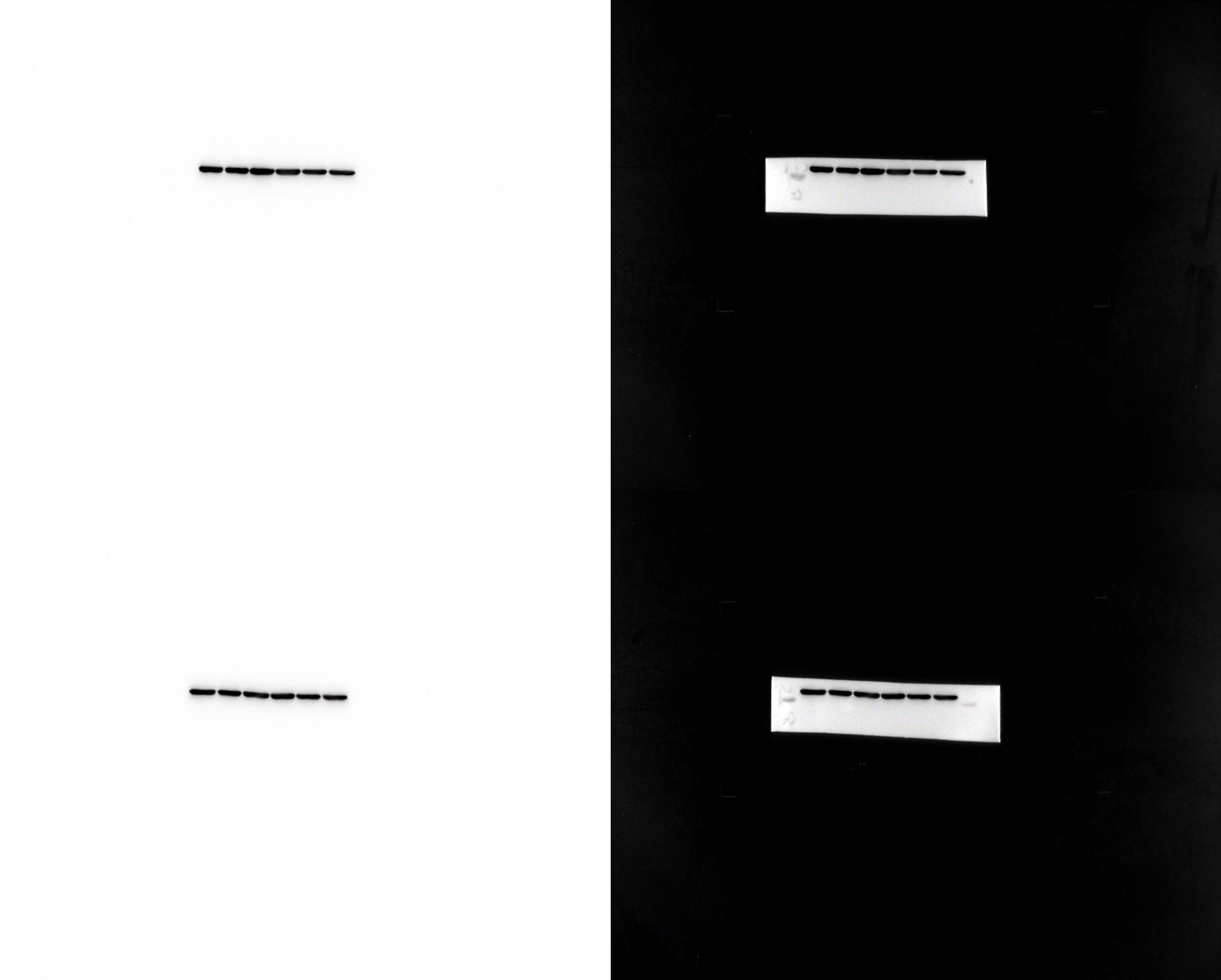

Supplement: Figure 4—source data 1. [file elife-96161-fig4-data1.zip › Figure 4-Source data1/Figure4B-Source data1-GAPDH.png]

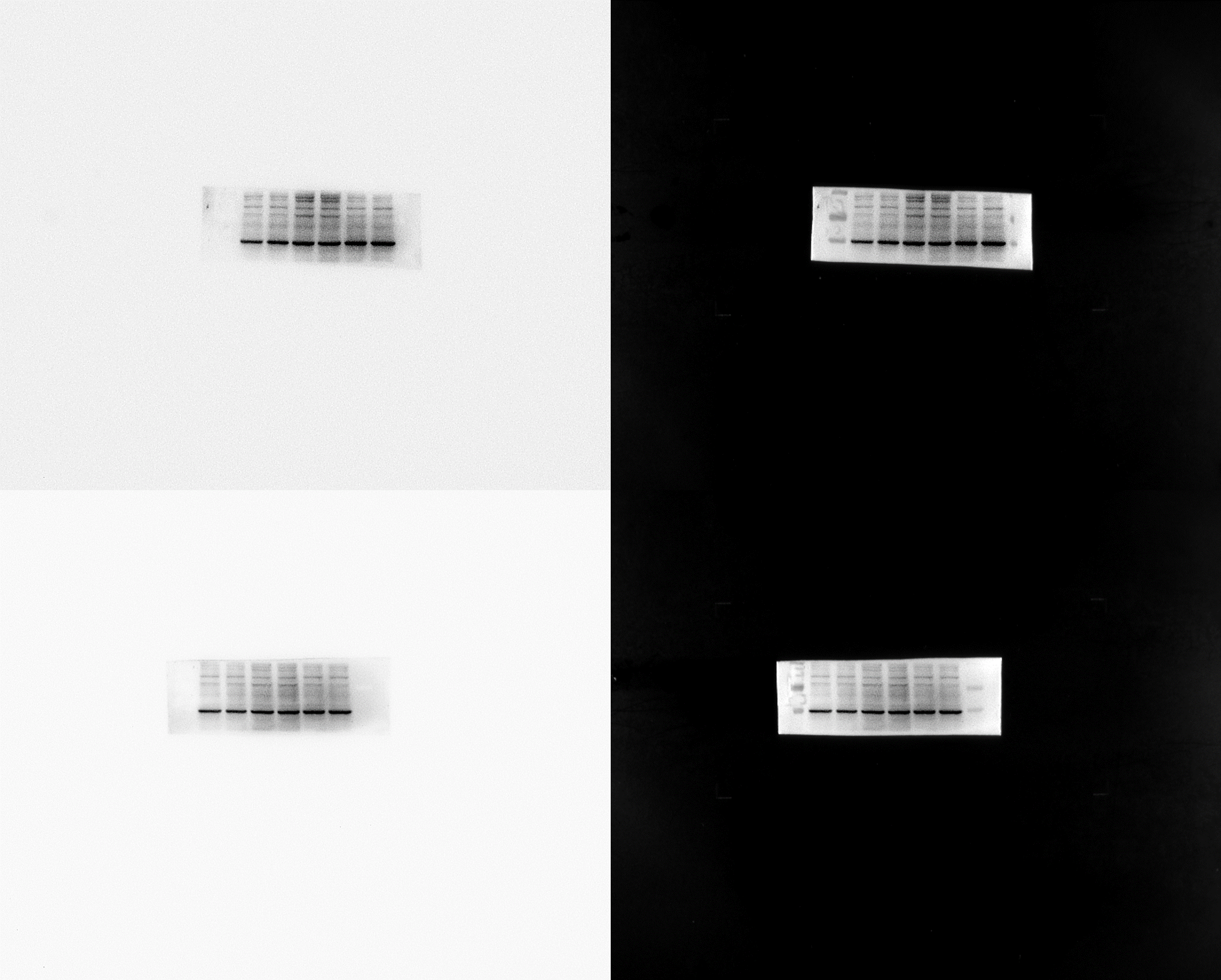

Supplement: Figure 4—source data 1. [file elife-96161-fig4-data1.zip › Figure 4-Source data1/Figure4B-Source data1-p-FOXO1.png]

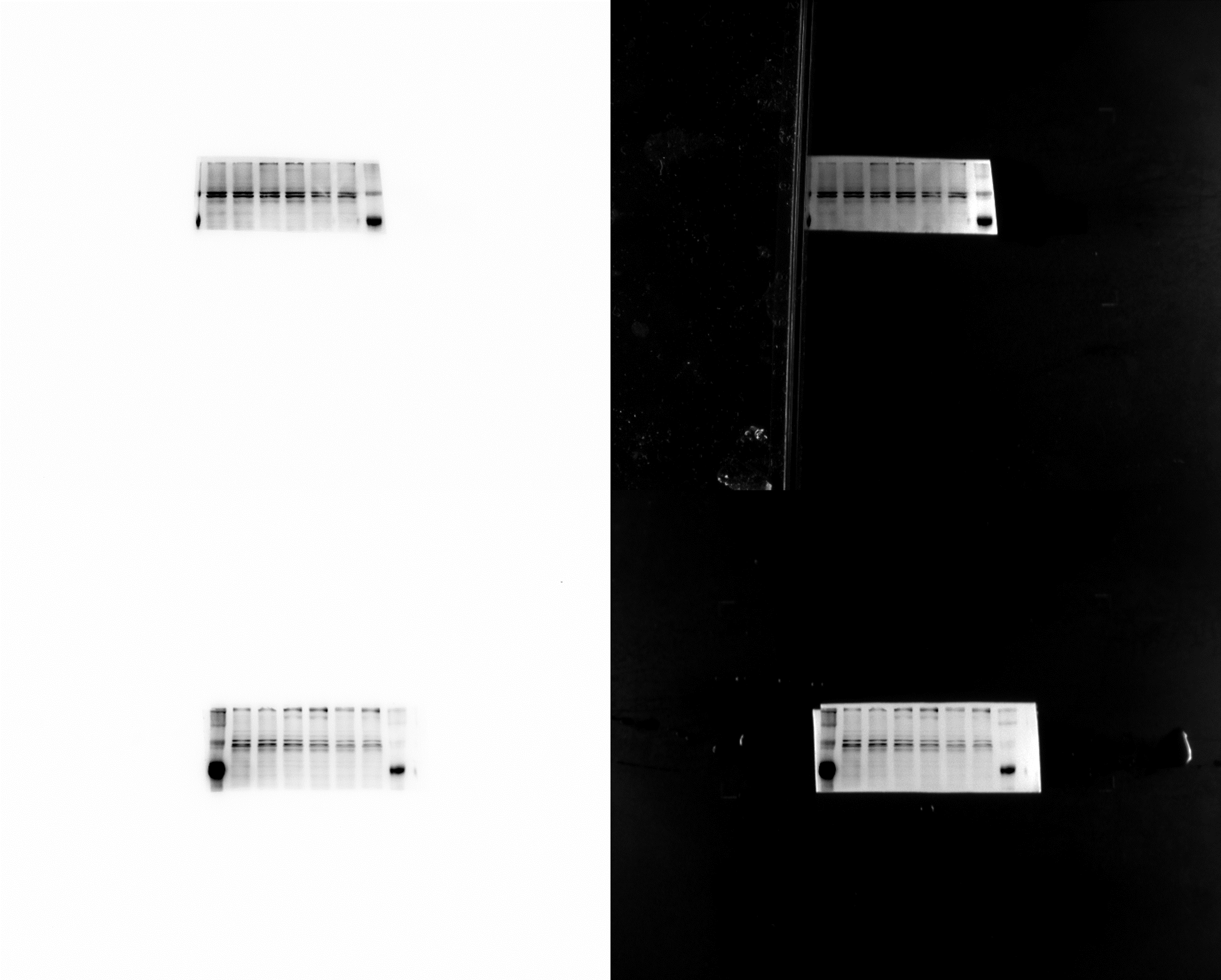

Supplement: Figure 4—source data 1. [file elife-96161-fig4-data1.zip › Figure 4-Source data1/Figure4B-Source data2-FOXO1.png]

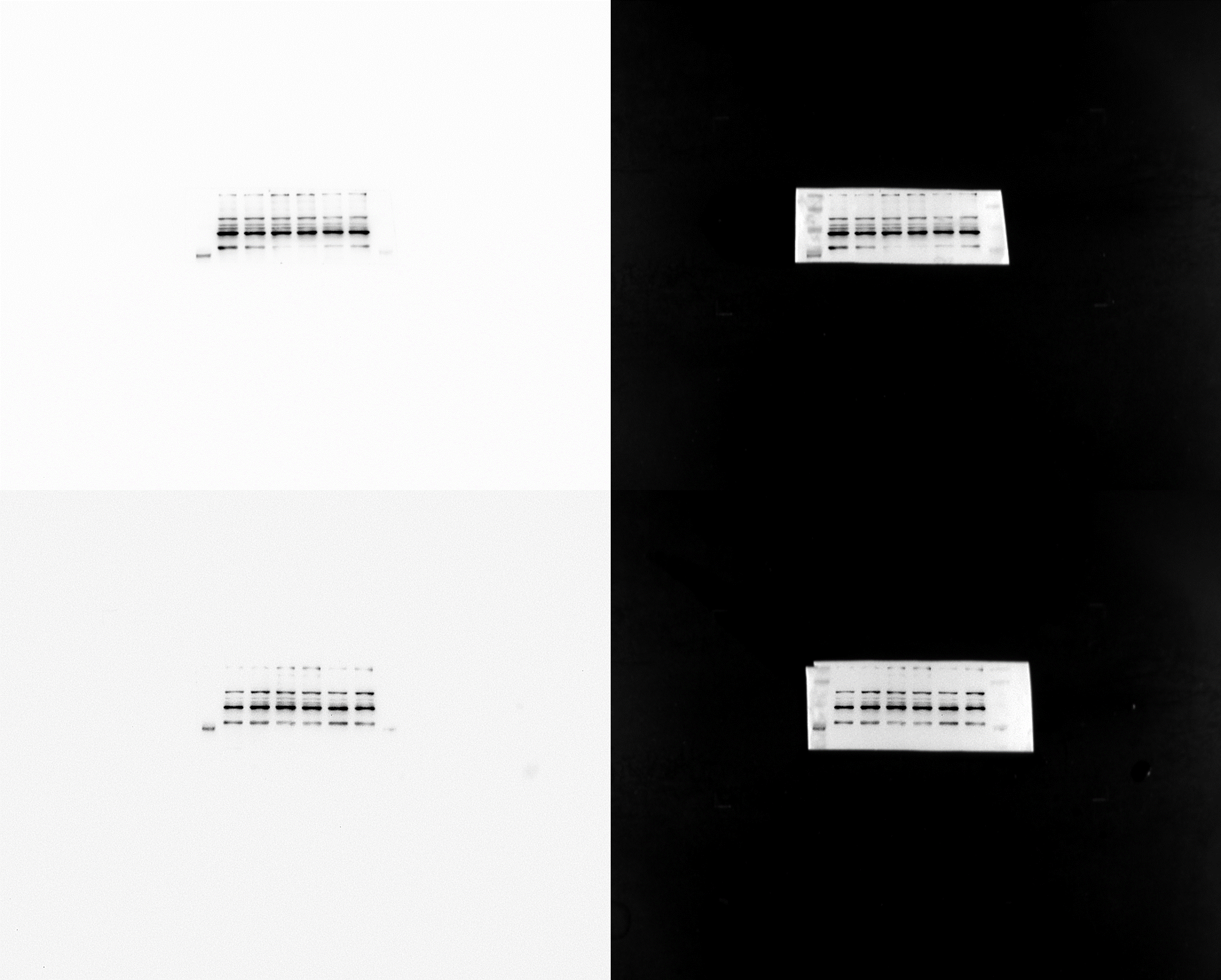

Supplement: Figure 4—source data 1. [file elife-96161-fig4-data1.zip › Figure 4-Source data1/Figure4B-Source data2-Lamin B.png]

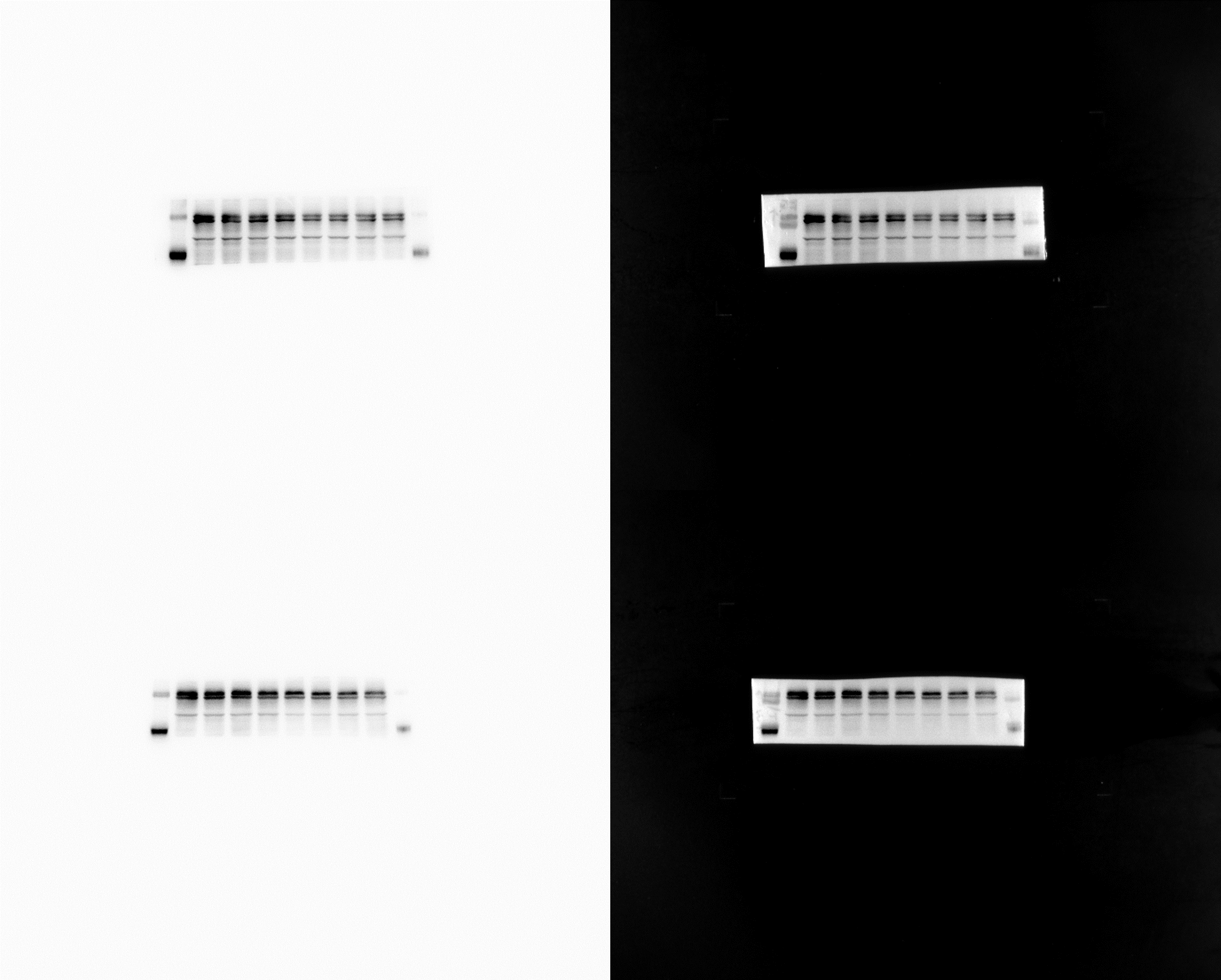

Supplement: Figure 4—source data 1. [file elife-96161-fig4-data1.zip › Figure 4-Source data1/Figure4C-Source data1-FOXO1.png]

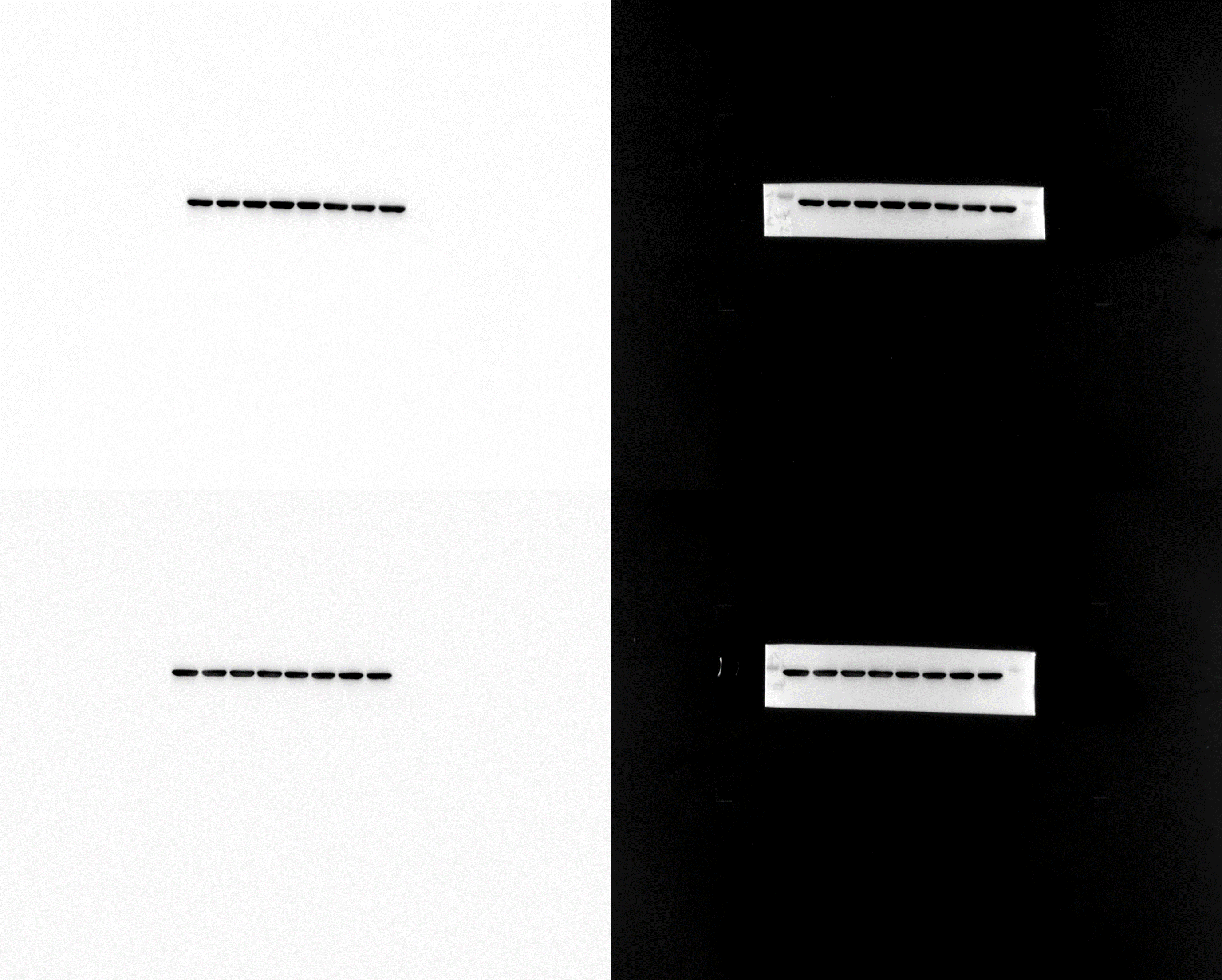

Supplement: Figure 4—source data 1. [file elife-96161-fig4-data1.zip › Figure 4-Source data1/Figure4C-Source data1-a┬-actin.png]

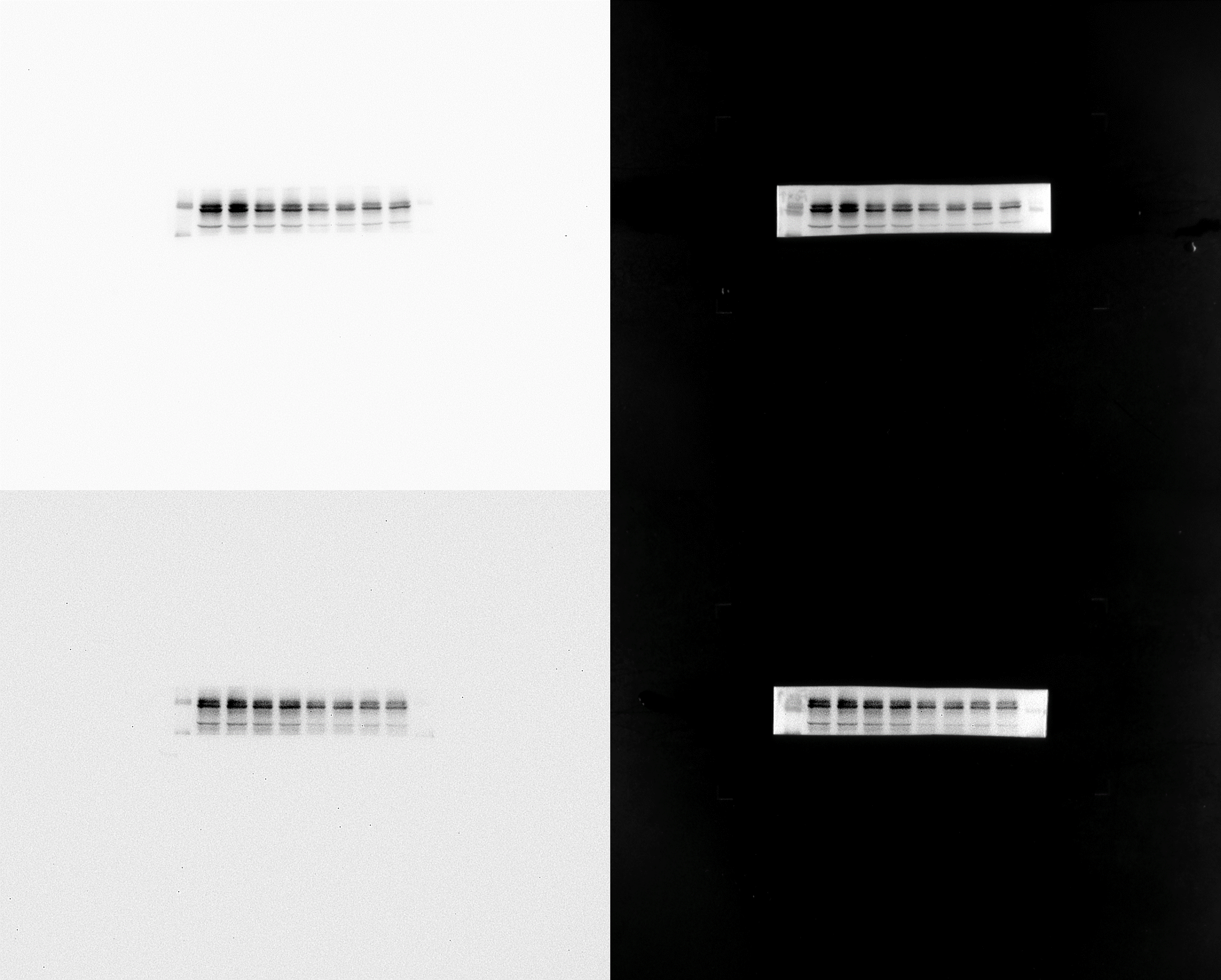

Supplement: Figure 4—source data 1. [file elife-96161-fig4-data1.zip › Figure 4-Source data1/Figure4C-Source data2-FOXO1.png]

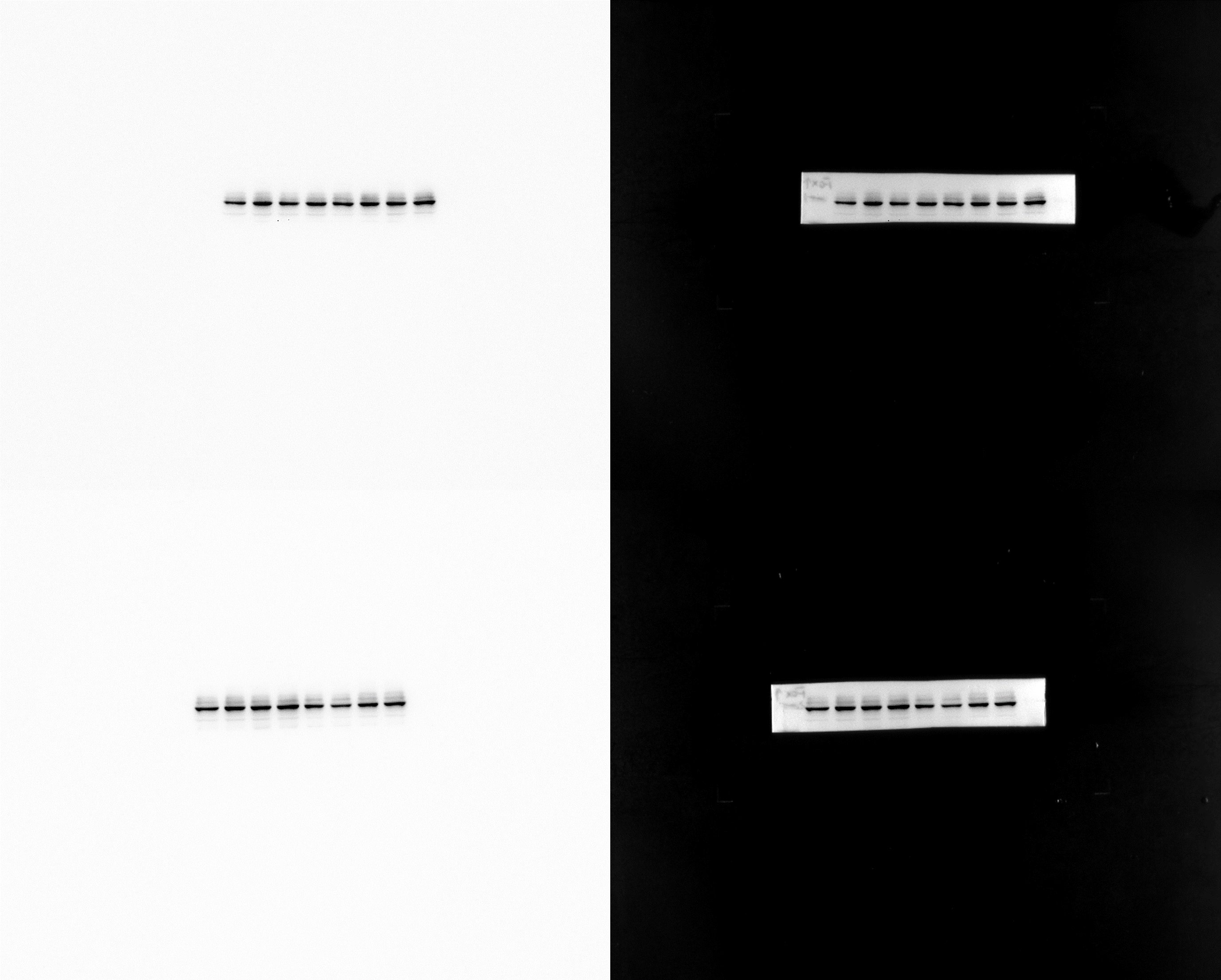

Supplement: Figure 4—source data 1. [file elife-96161-fig4-data1.zip › Figure 4-Source data1/Figure4C-Source data2-Lamin B.png]

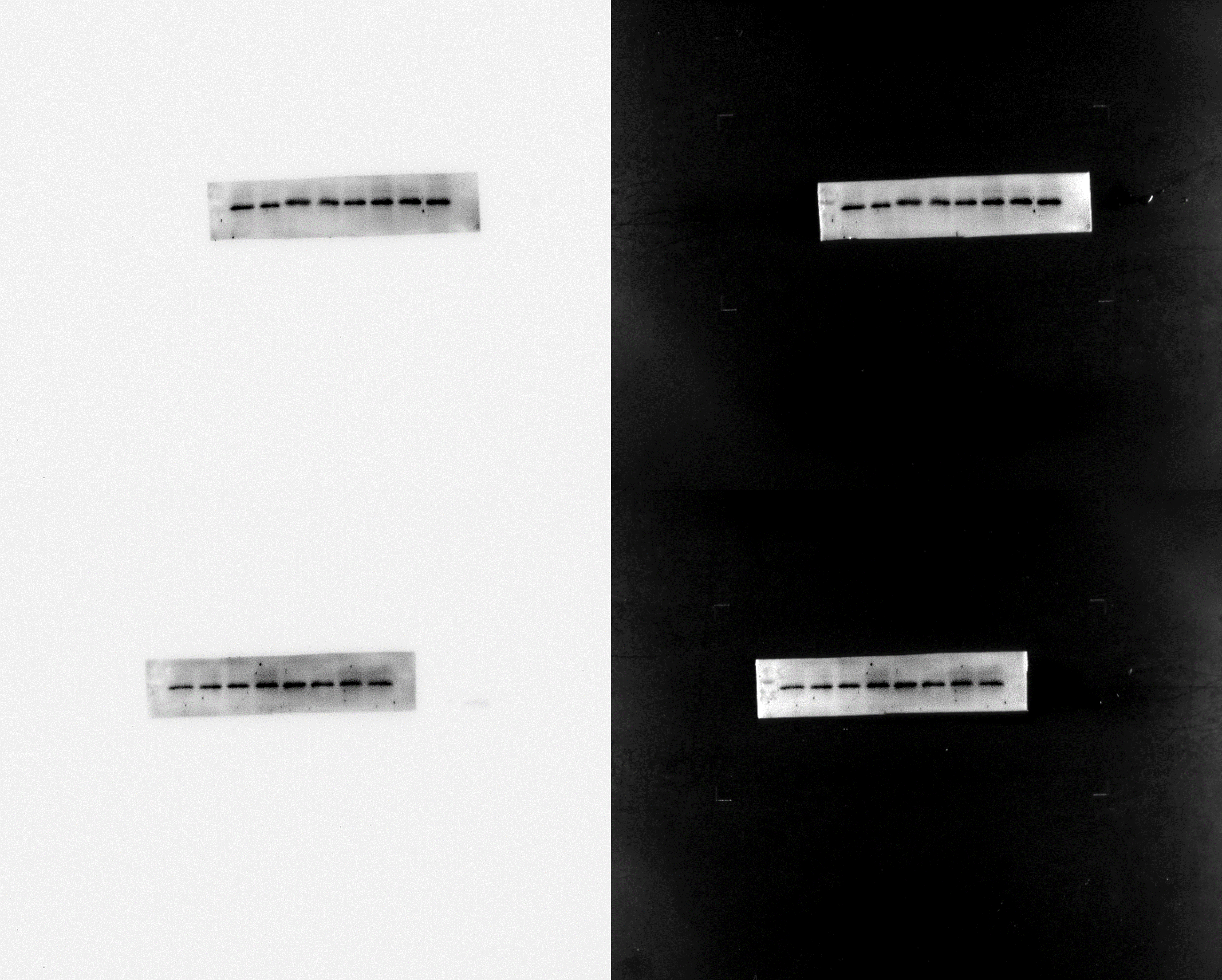

Supplement: Figure 4—source data 1. [file elife-96161-fig4-data1.zip › Figure 4-Source data1/Figure4D-Source data1-Claudin-5.png]

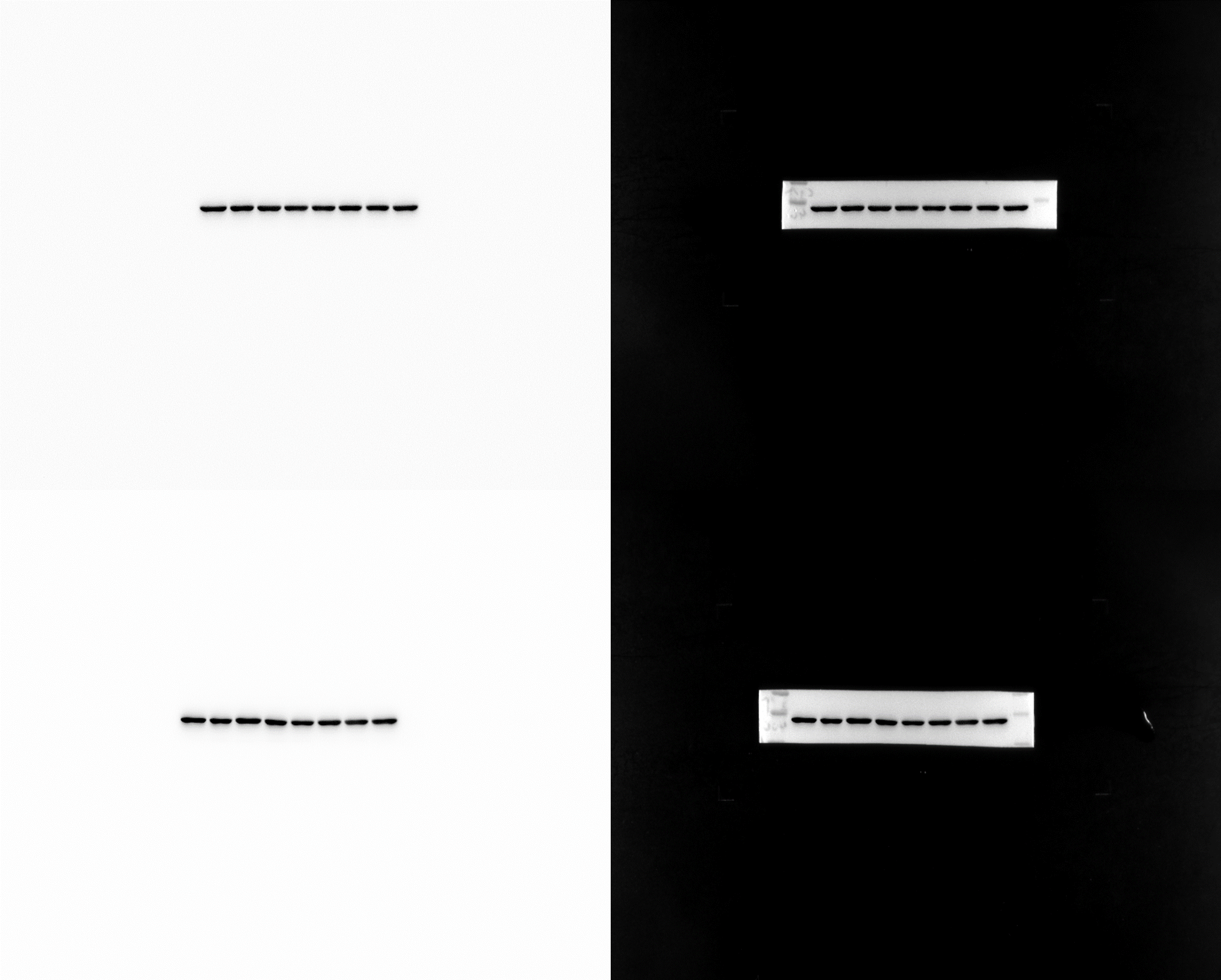

Supplement: Figure 4—source data 1. [file elife-96161-fig4-data1.zip › Figure 4-Source data1/Figure4D-Source data1-a┬-actin.png]

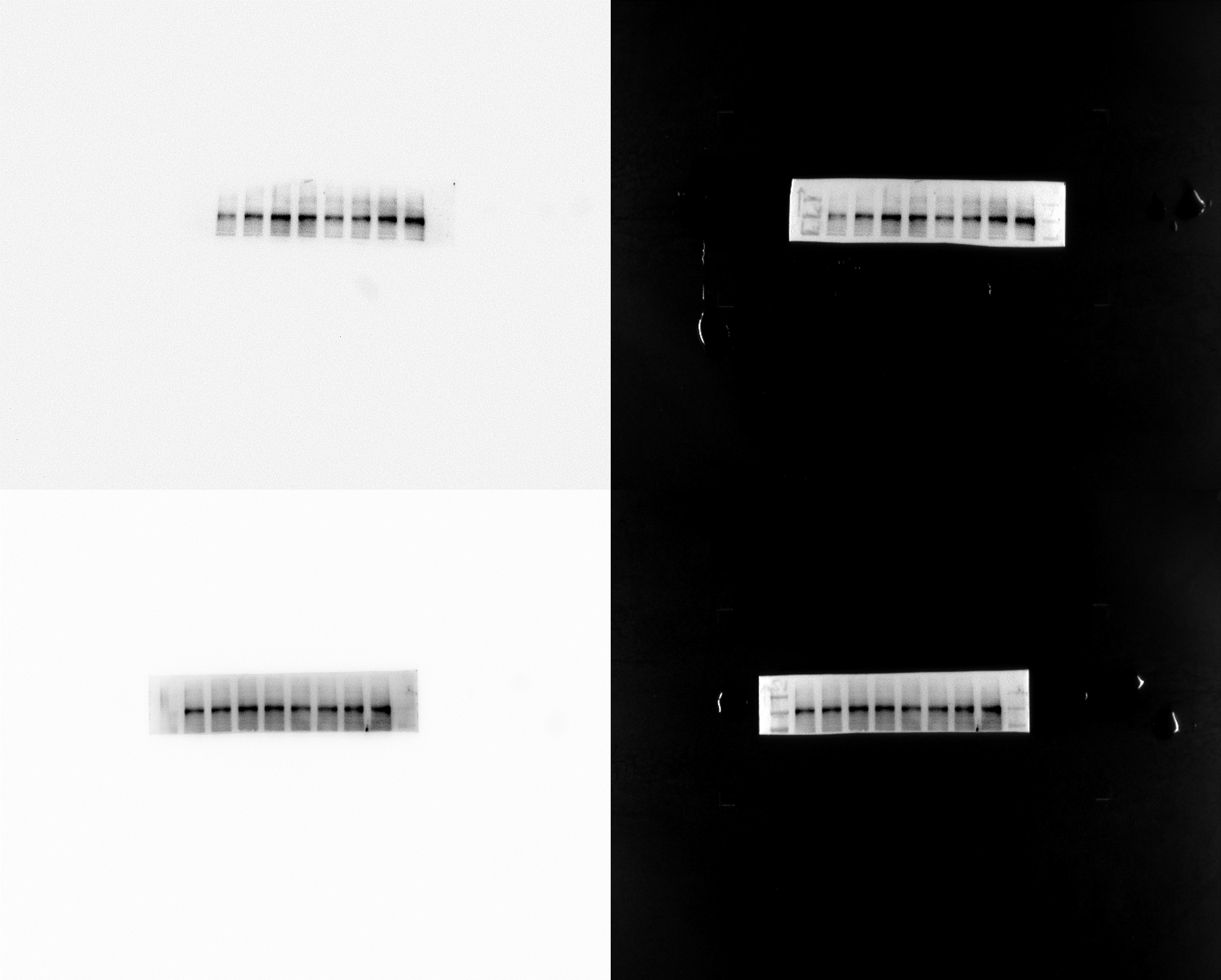

Supplement: Figure 4—source data 1. [file elife-96161-fig4-data1.zip › Figure 4-Source data1/Figure4D-Source data2-VE-Cadherin.png]

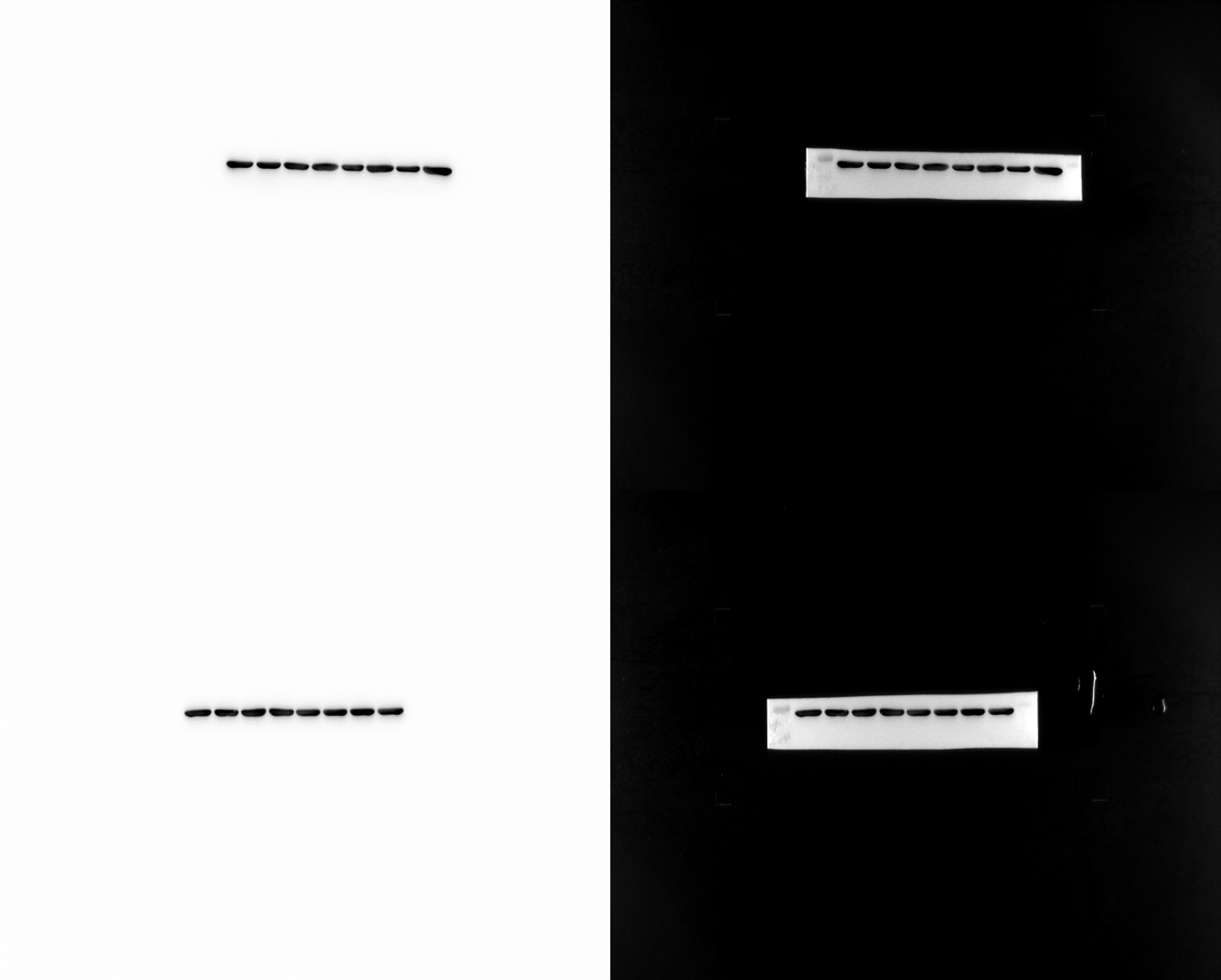

Supplement: Figure 4—source data 1. [file elife-96161-fig4-data1.zip › Figure 4-Source data1/Figure4D-Source data2-a┬-actin.png]

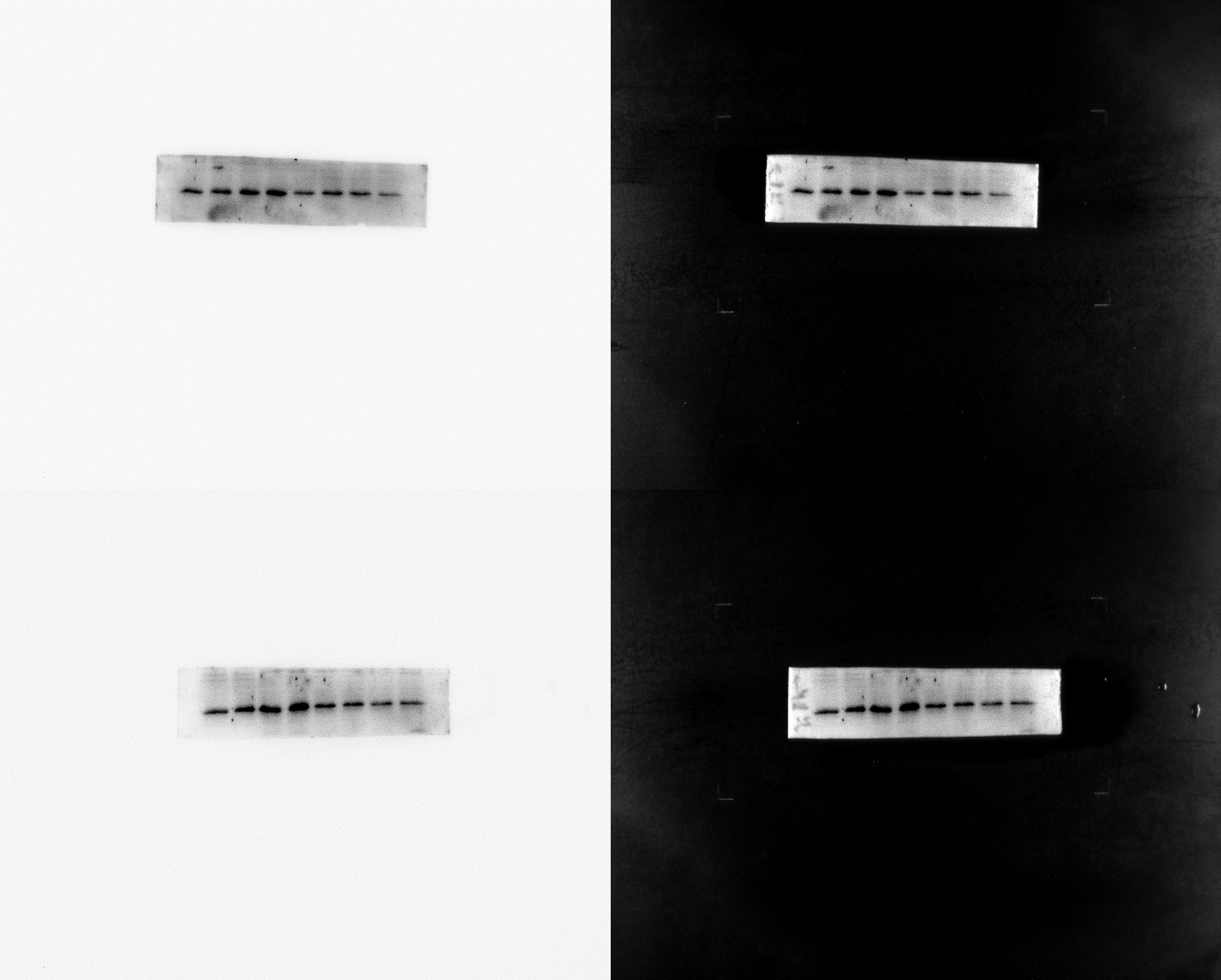

Supplement: Figure 4—source data 1. [file elife-96161-fig4-data1.zip › Figure 4-Source data1/Figure4E-Source data1-Claudin-5.png]

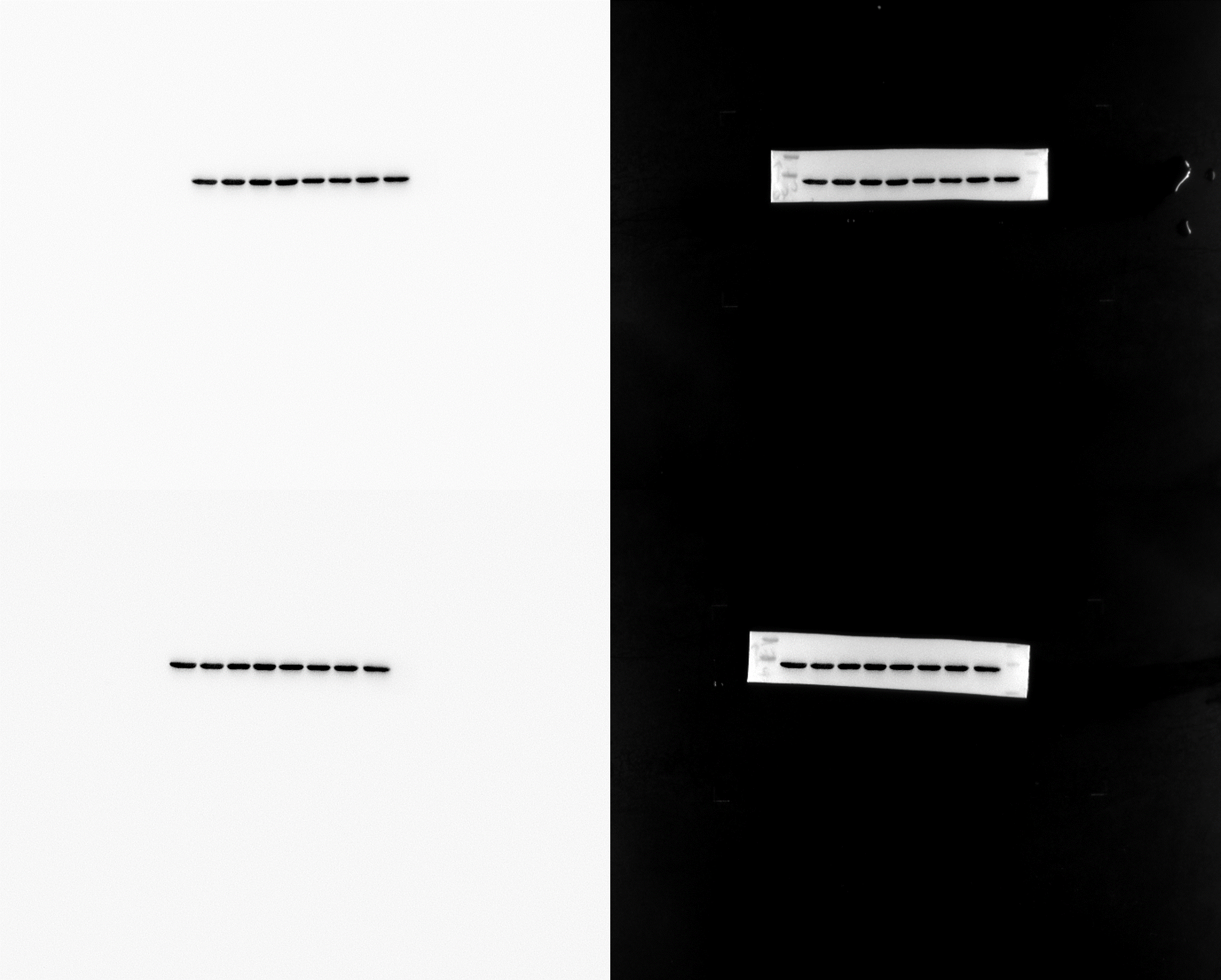

Supplement: Figure 4—source data 1. [file elife-96161-fig4-data1.zip › Figure 4-Source data1/Figure4E-Source data1-a┬-actin.png]

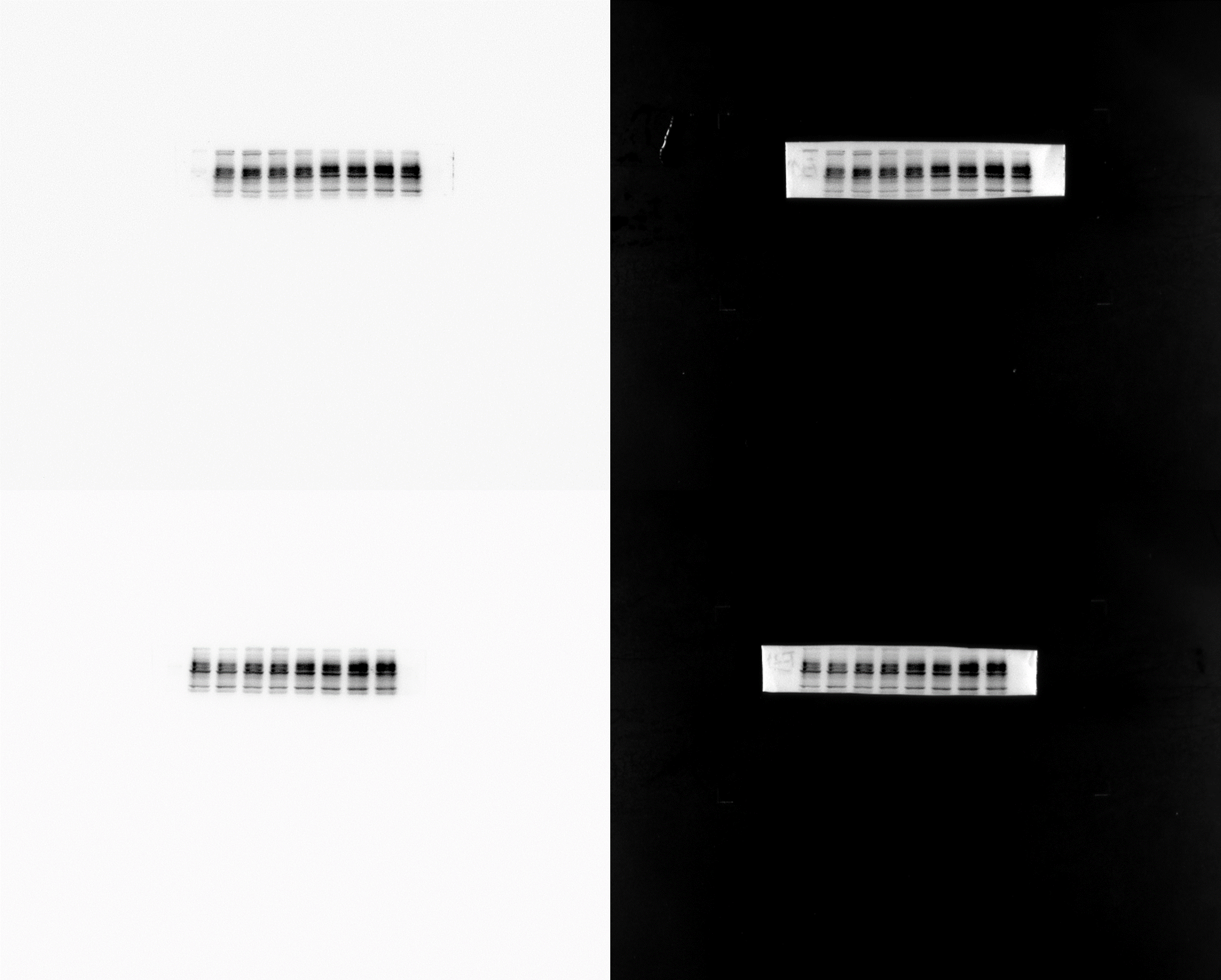

Supplement: Figure 4—source data 1. [file elife-96161-fig4-data1.zip › Figure 4-Source data1/Figure4E-Source data2-FOXO1.png]

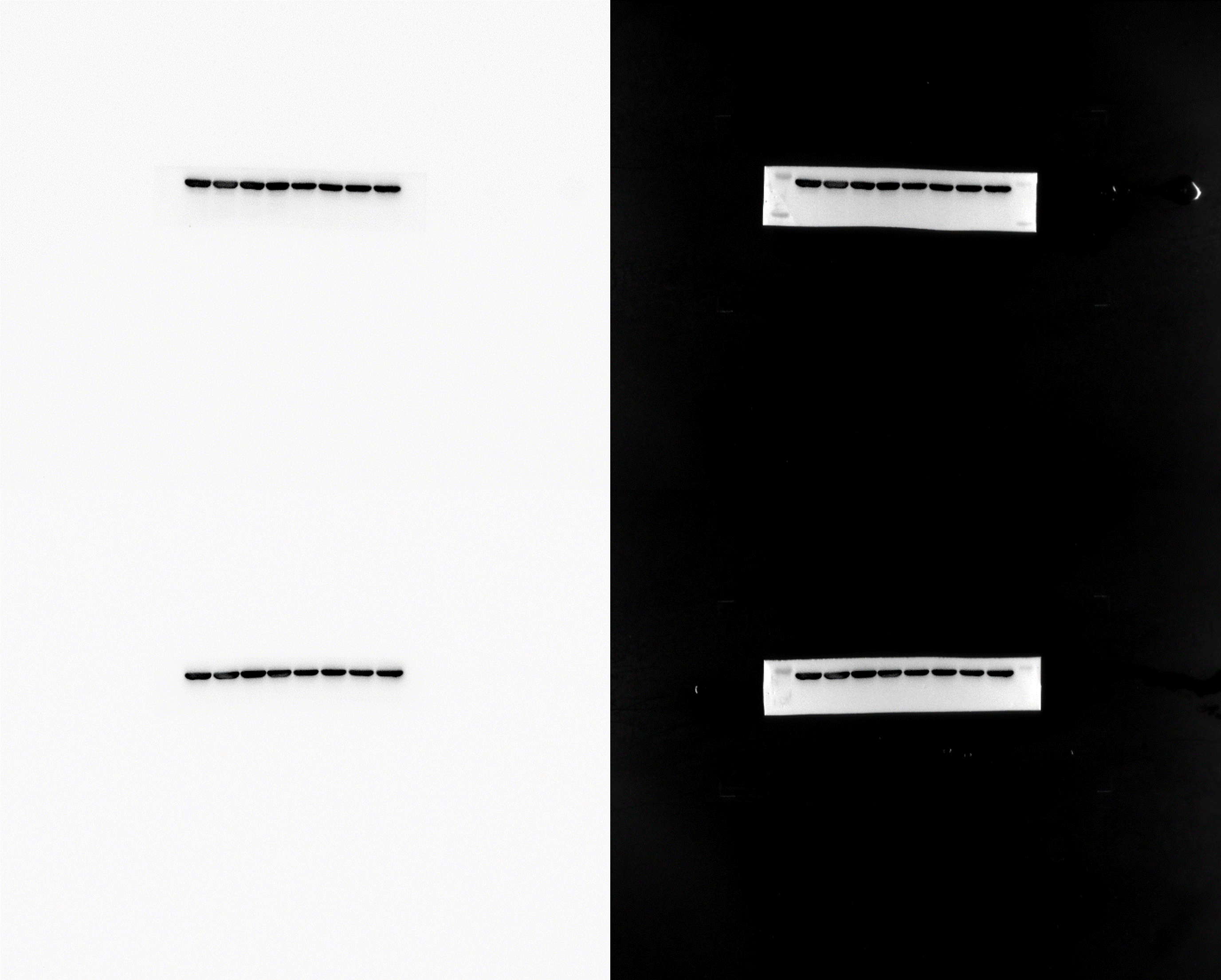

Supplement: Figure 4—source data 1. [file elife-96161-fig4-data1.zip › Figure 4-Source data1/Figure4E-Source data2-a┬-actin.png]

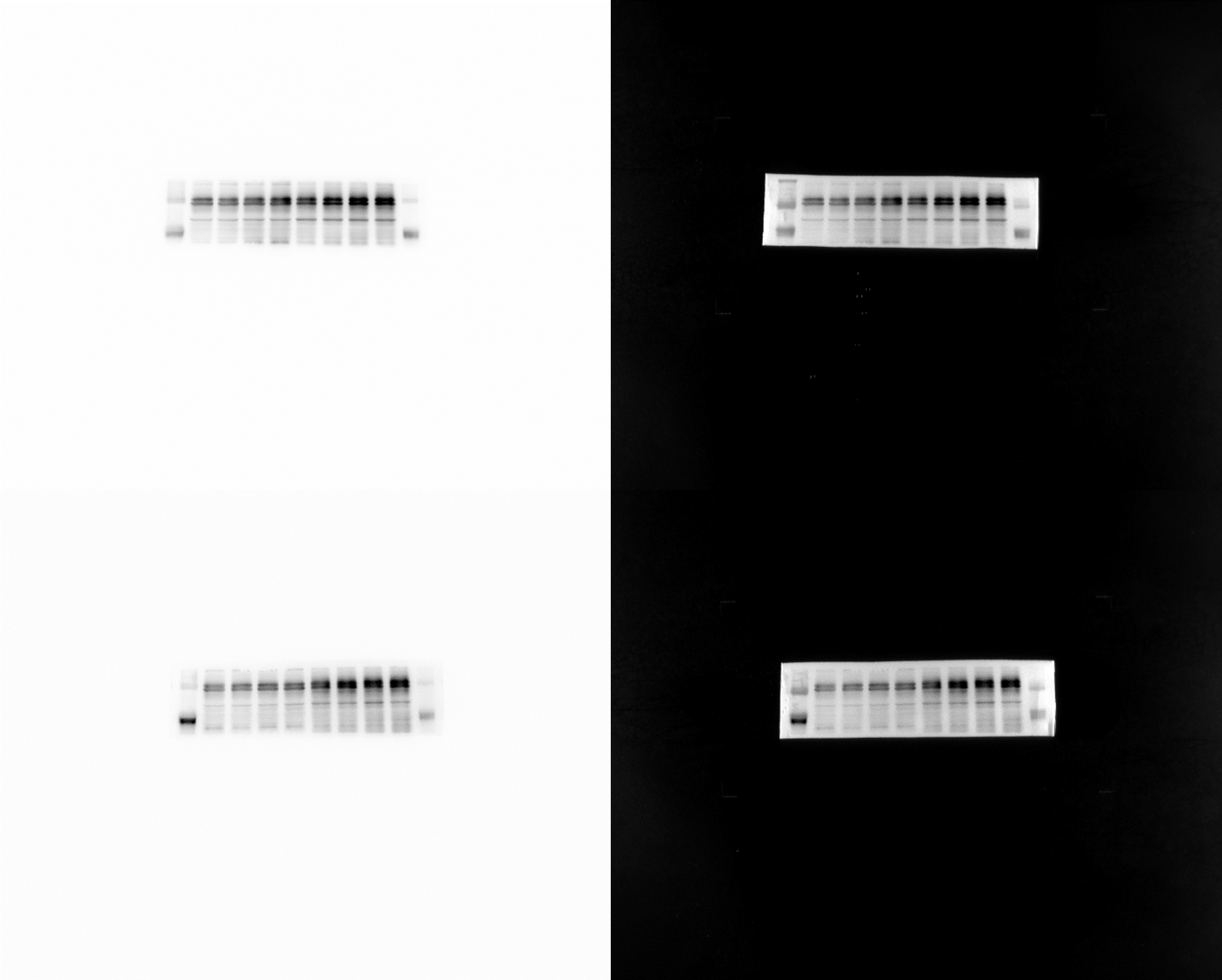

Supplement: Figure 4—source data 1. [file elife-96161-fig4-data1.zip › Figure 4-Source data1/Figure4E-Source data3-FOXO1.png]

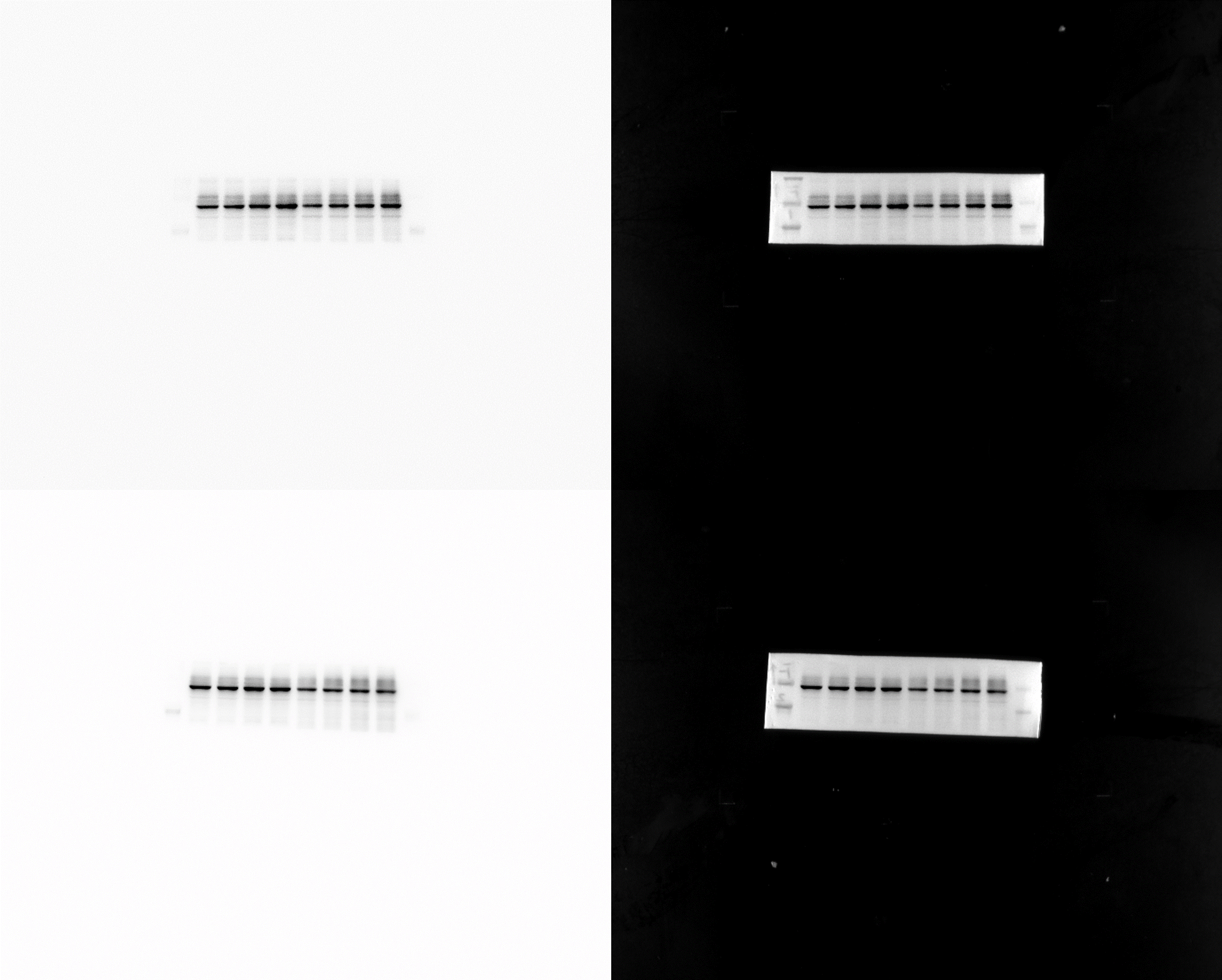

Supplement: Figure 4—source data 1. [file elife-96161-fig4-data1.zip › Figure 4-Source data1/Figure4E-Source data3-Lamin B.png]

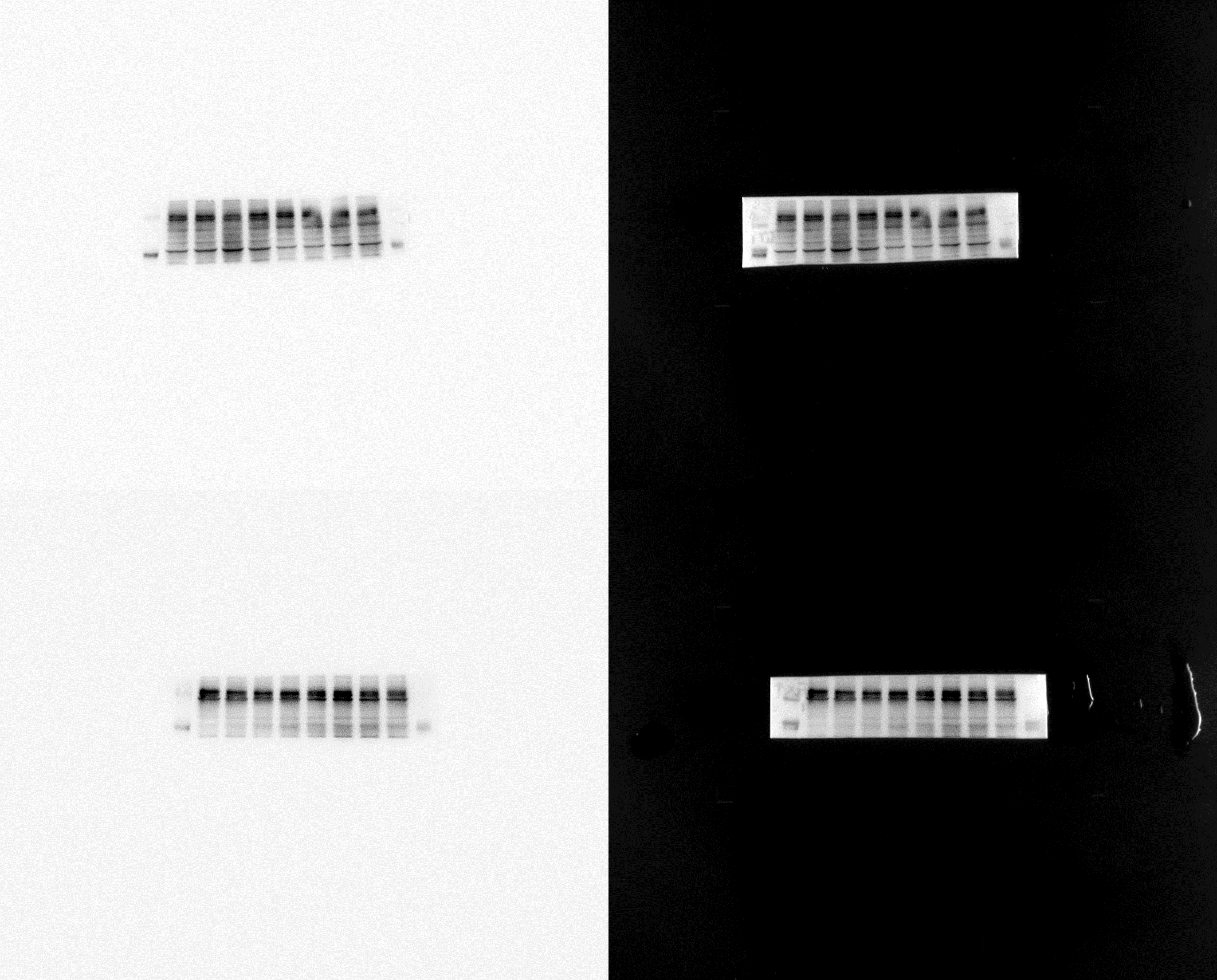

Supplement: Figure 4—source data 1. [file elife-96161-fig4-data1.zip › Figure 4-Source data1/Figure4F-Source data1-FOXO1.png]

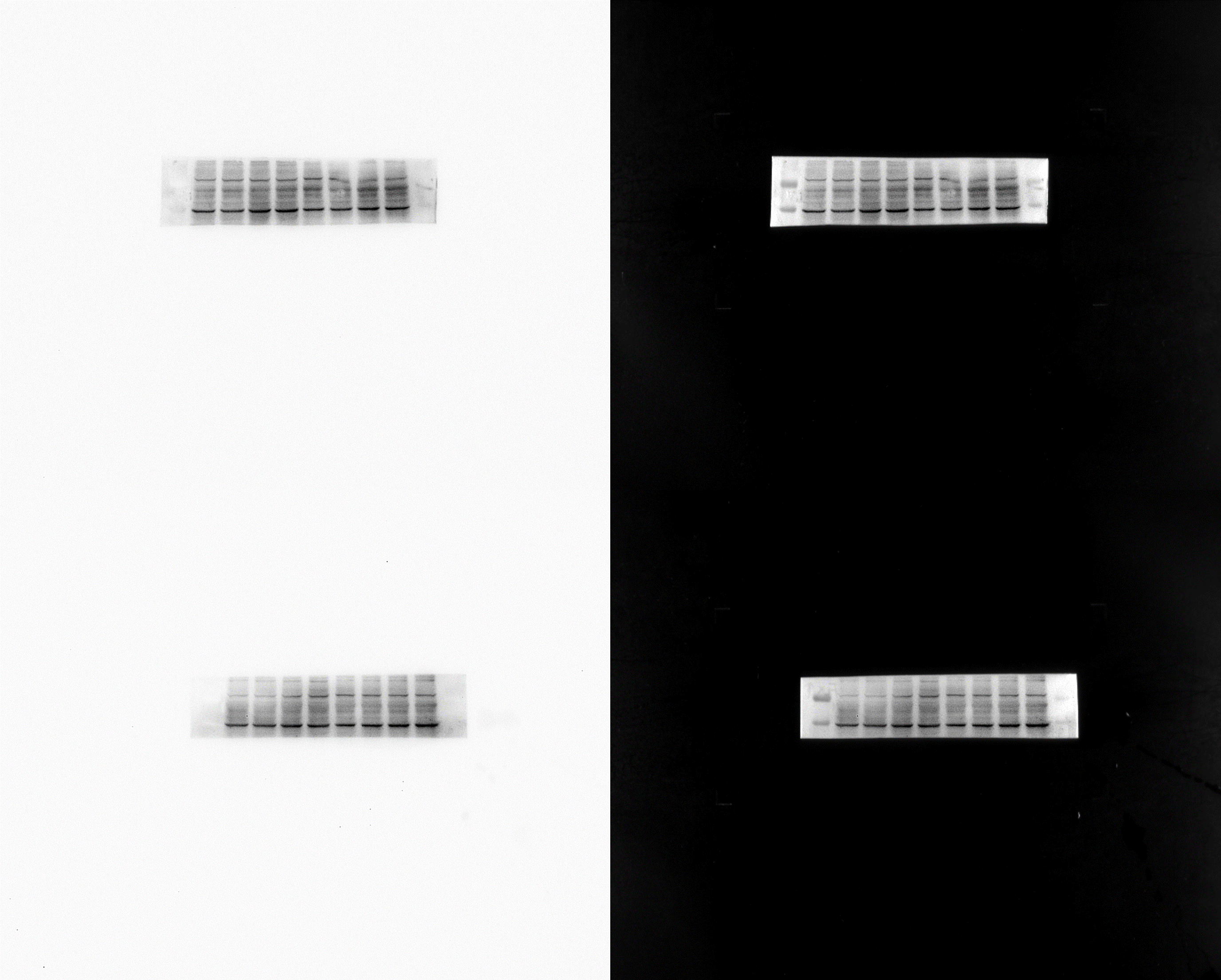

Supplement: Figure 4—source data 1. [file elife-96161-fig4-data1.zip › Figure 4-Source data1/Figure4F-Source data1-p-FOXO1.png]

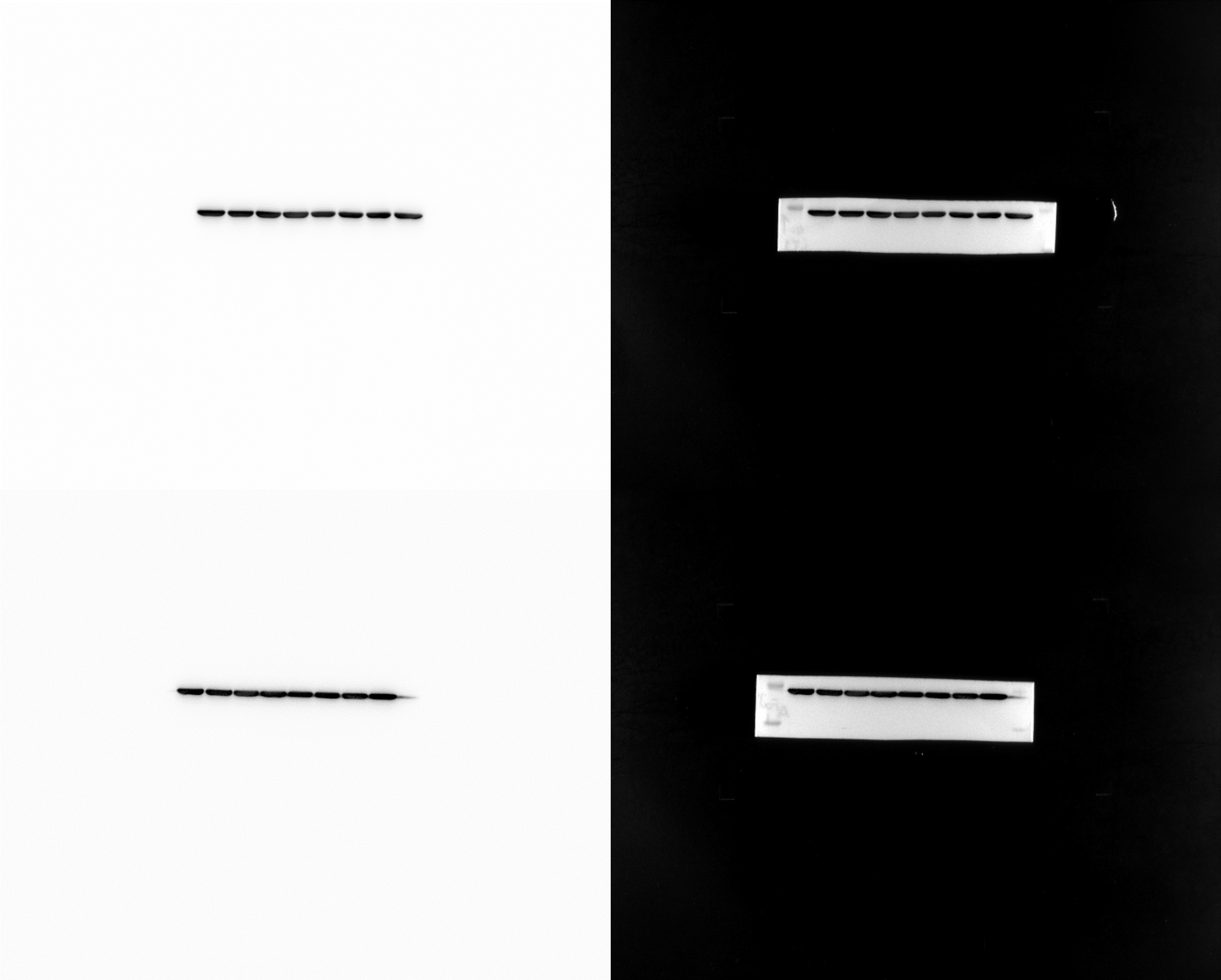

Supplement: Figure 4—source data 1. [file elife-96161-fig4-data1.zip › Figure 4-Source data1/Figure4F-Source data1-a┬-actin.png]

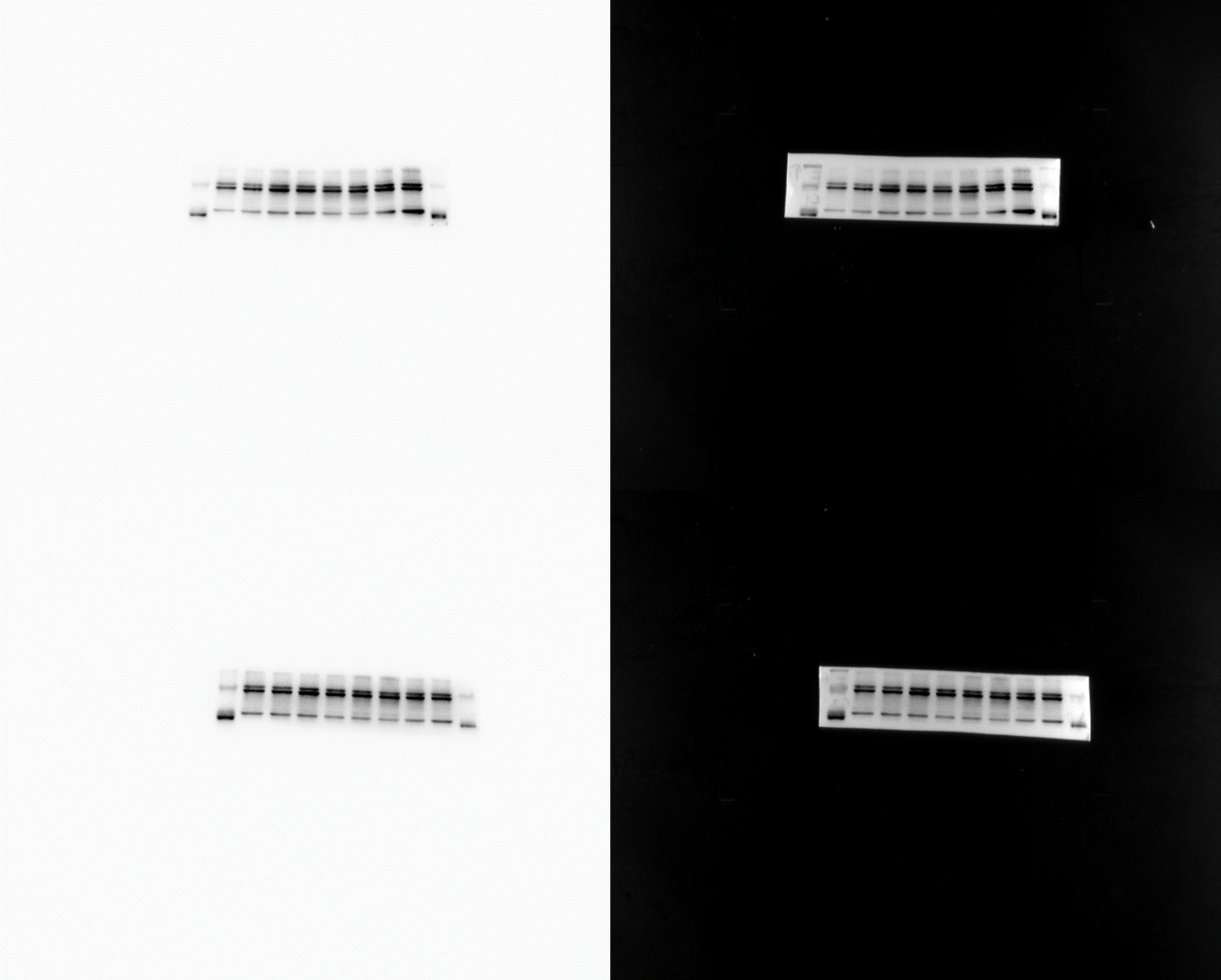

Supplement: Figure 4—source data 1. [file elife-96161-fig4-data1.zip › Figure 4-Source data1/Figure4F-Source data2-FOXO1.png]

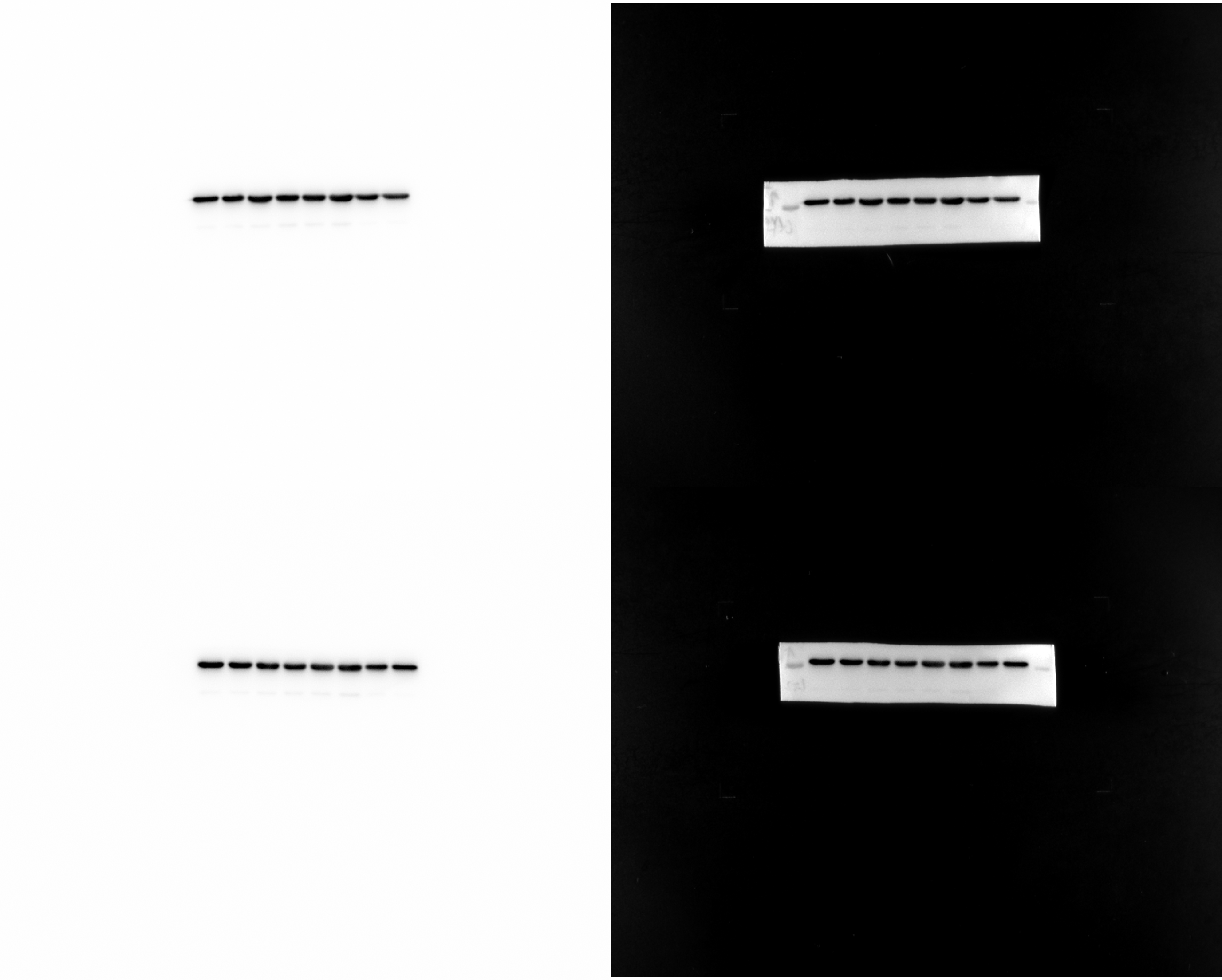

Supplement: Figure 4—source data 1. [file elife-96161-fig4-data1.zip › Figure 4-Source data1/Figure4F-Source data2-GAPDH.png]

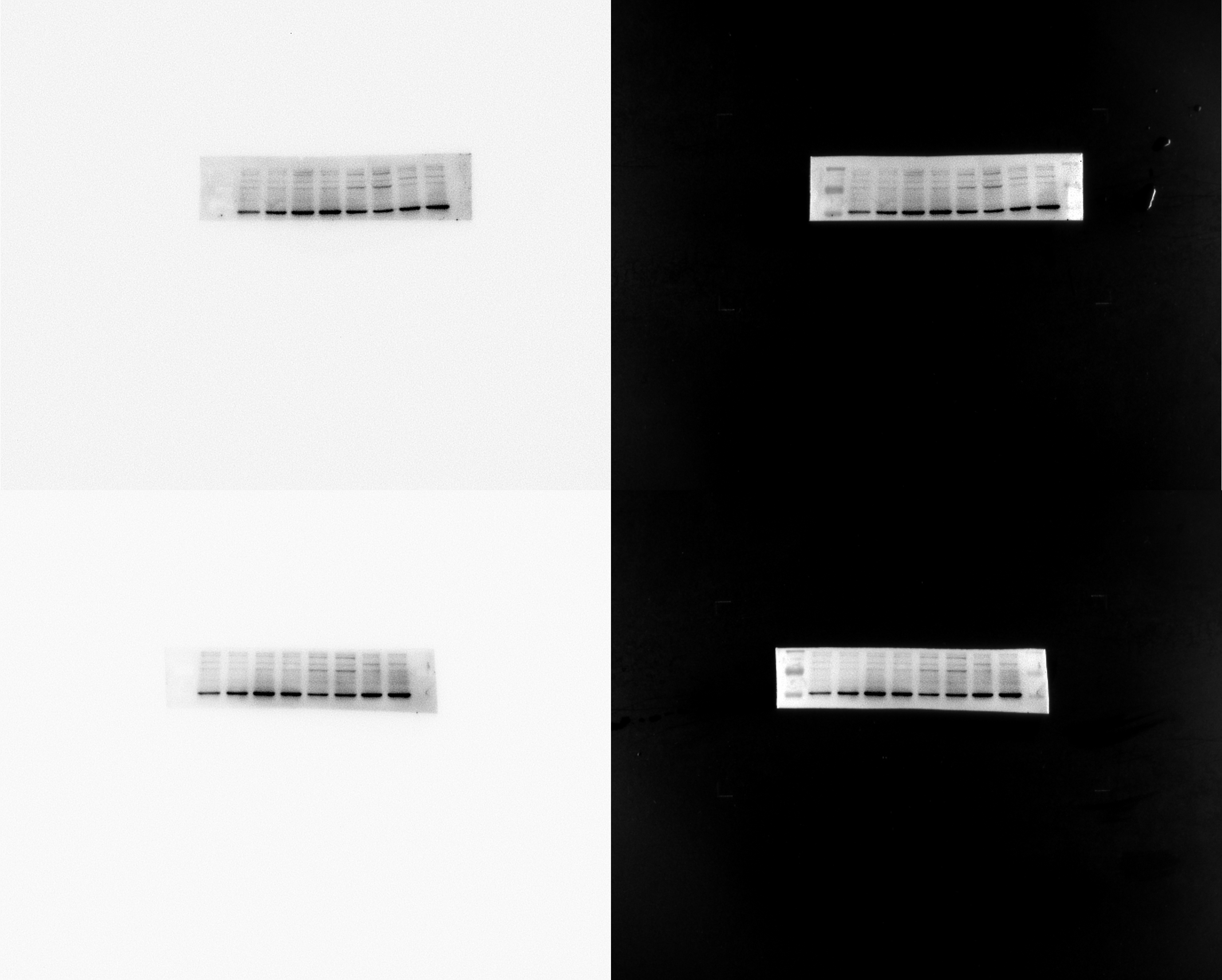

Supplement: Figure 4—source data 1. [file elife-96161-fig4-data1.zip › Figure 4-Source data1/Figure4F-Source data2-p-FOXO1.png]

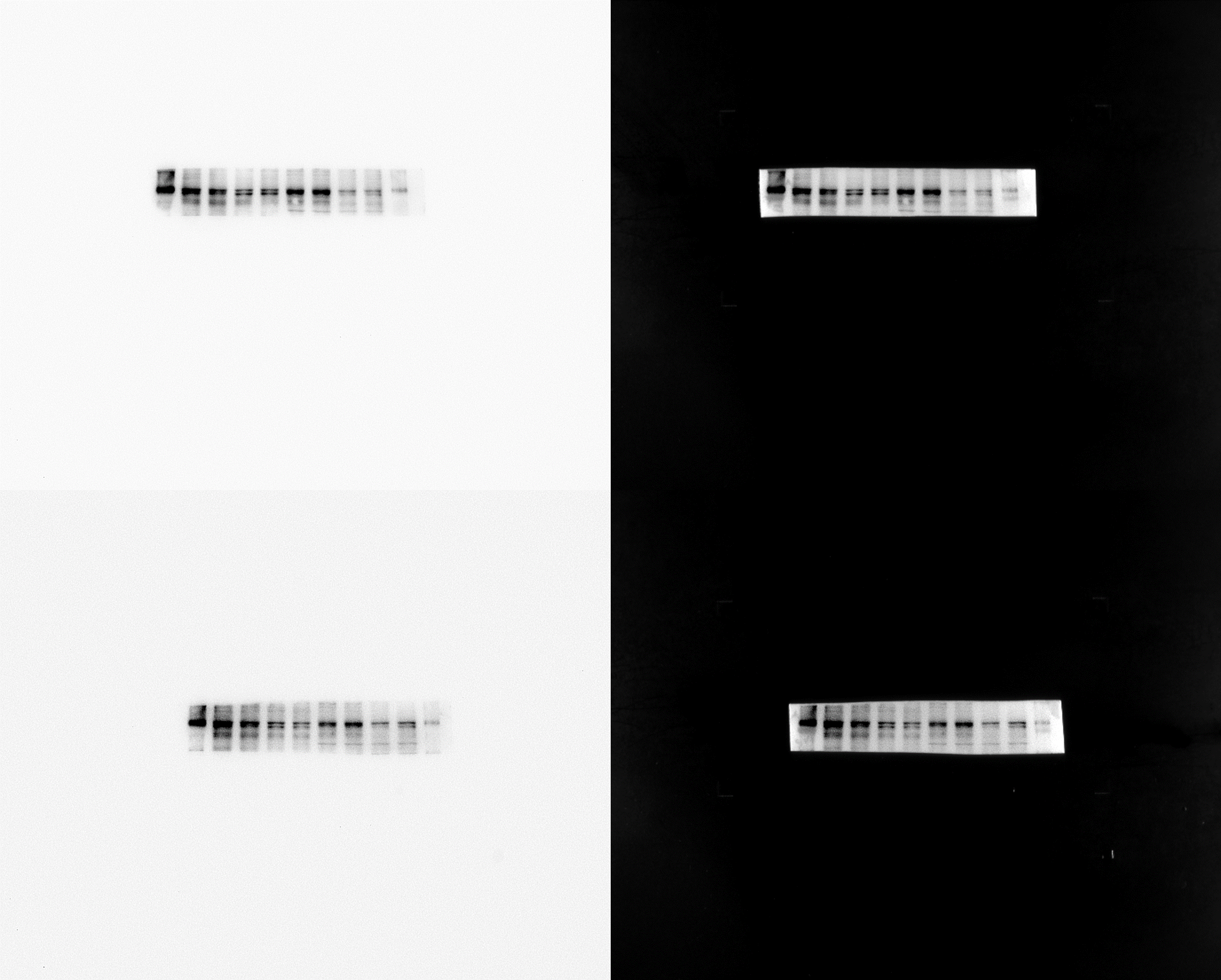

Supplement: Figure 4—source data 1. [file elife-96161-fig4-data1.zip › Figure 4-Source data1/Figure4F-Source data3-FOXO1.png]

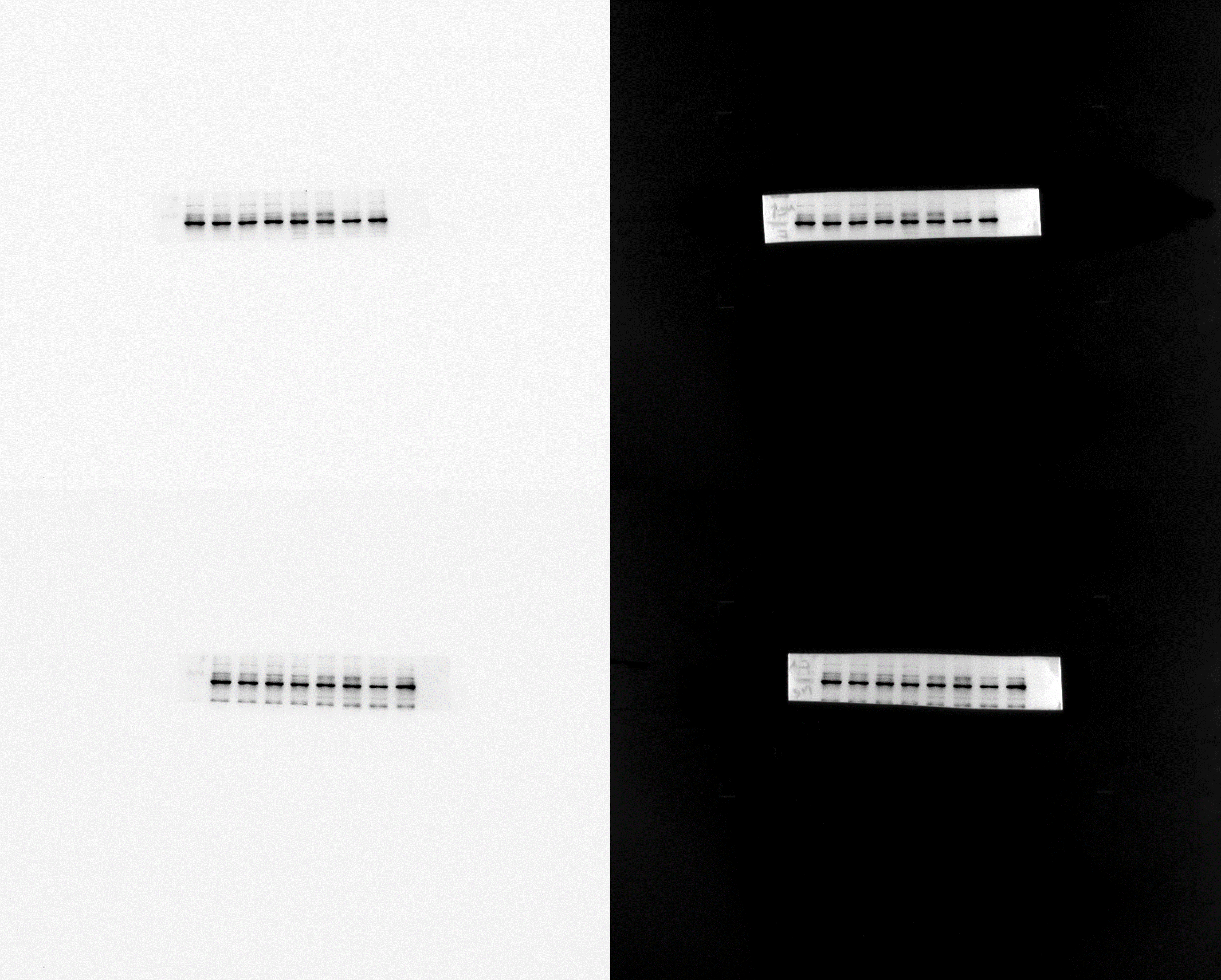

Supplement: Figure 4—source data 1. [file elife-96161-fig4-data1.zip › Figure 4-Source data1/Figure4F-Source data3-Lamin B.png]

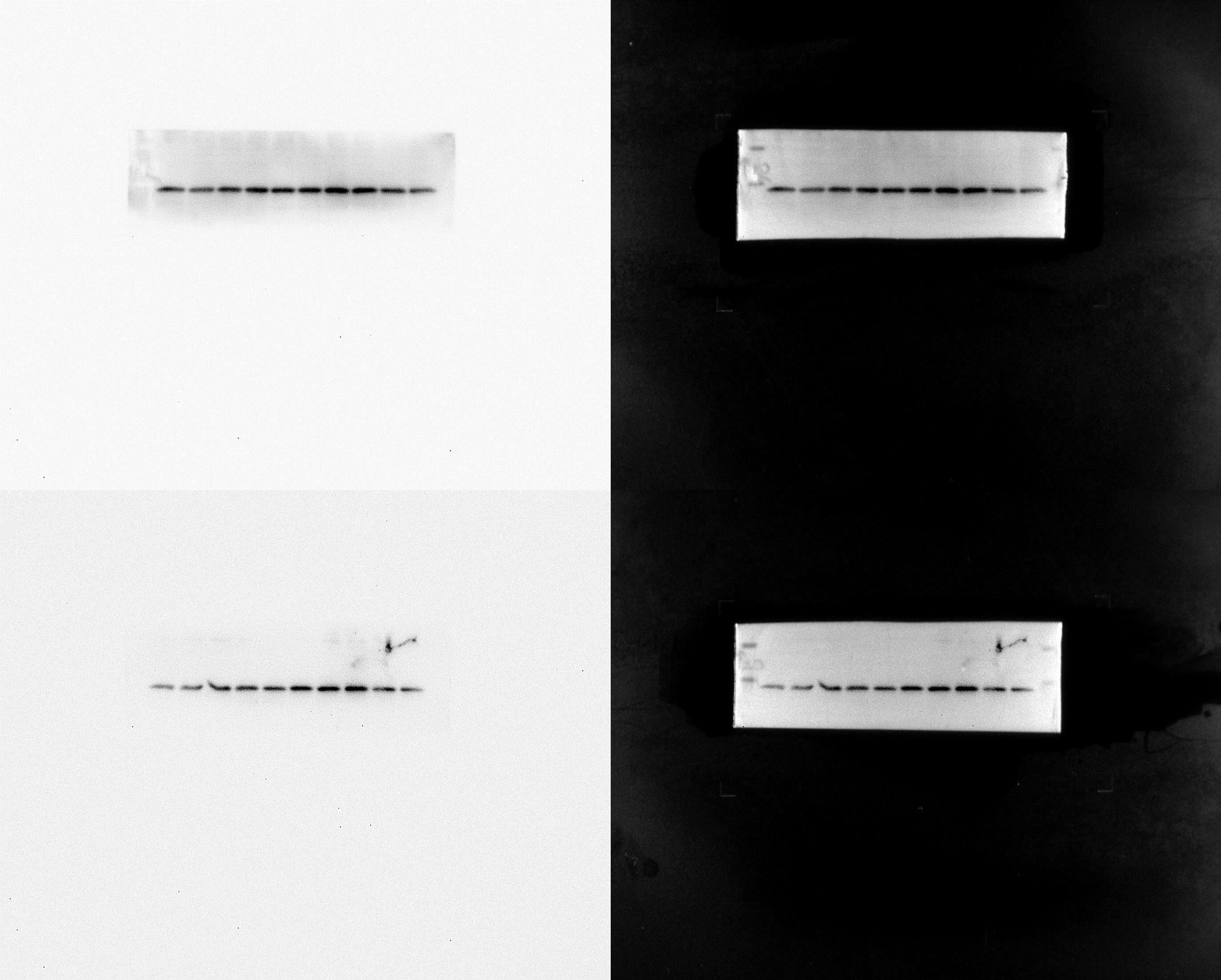

Supplement: Figure 4—source data 1. [file elife-96161-fig4-data1.zip › Figure 4-Source data1/Figure4G-Source data1-Claudin-5.png]

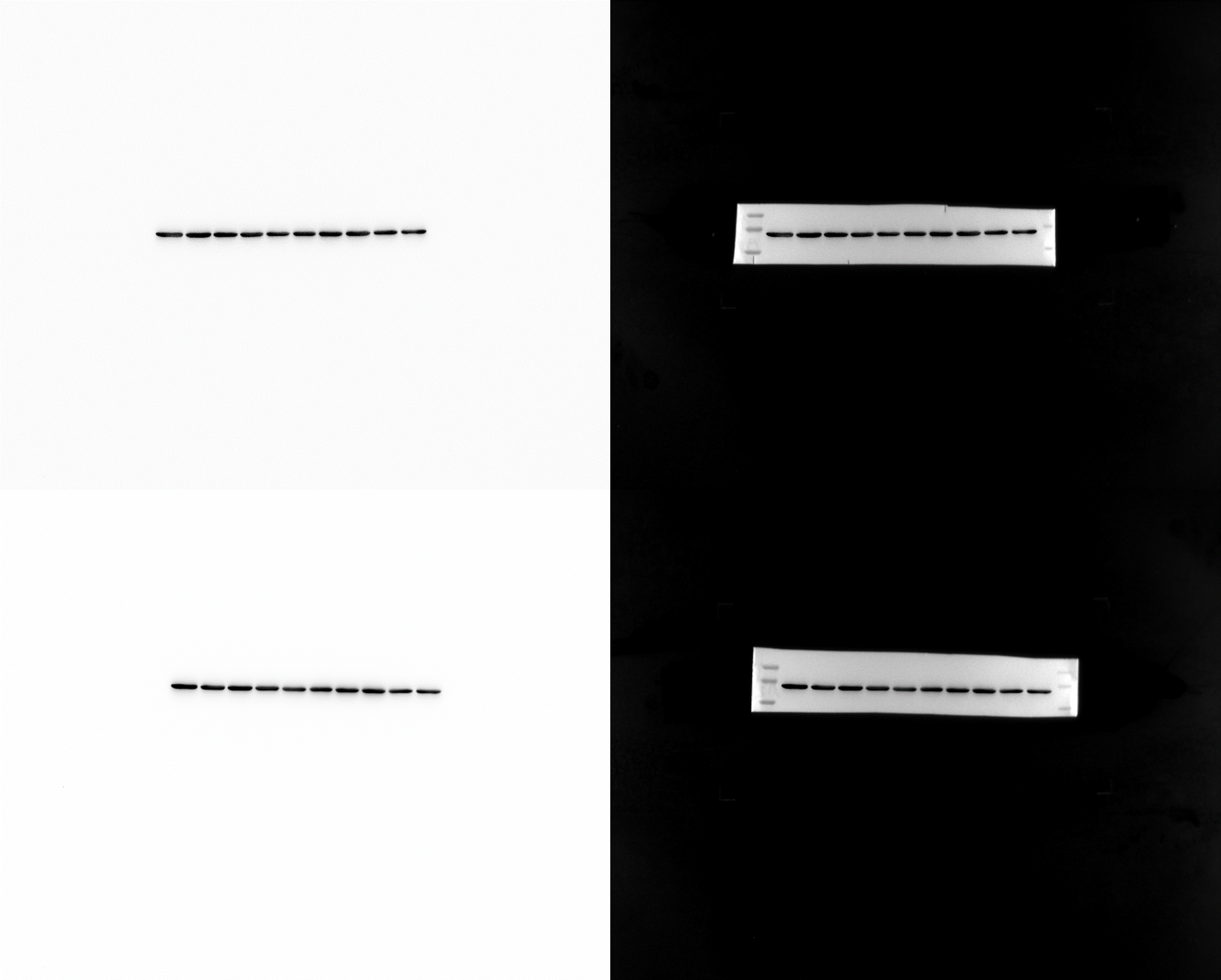

Supplement: Figure 4—source data 1. [file elife-96161-fig4-data1.zip › Figure 4-Source data1/Figure4G-Source data1-a┬-actin.png]

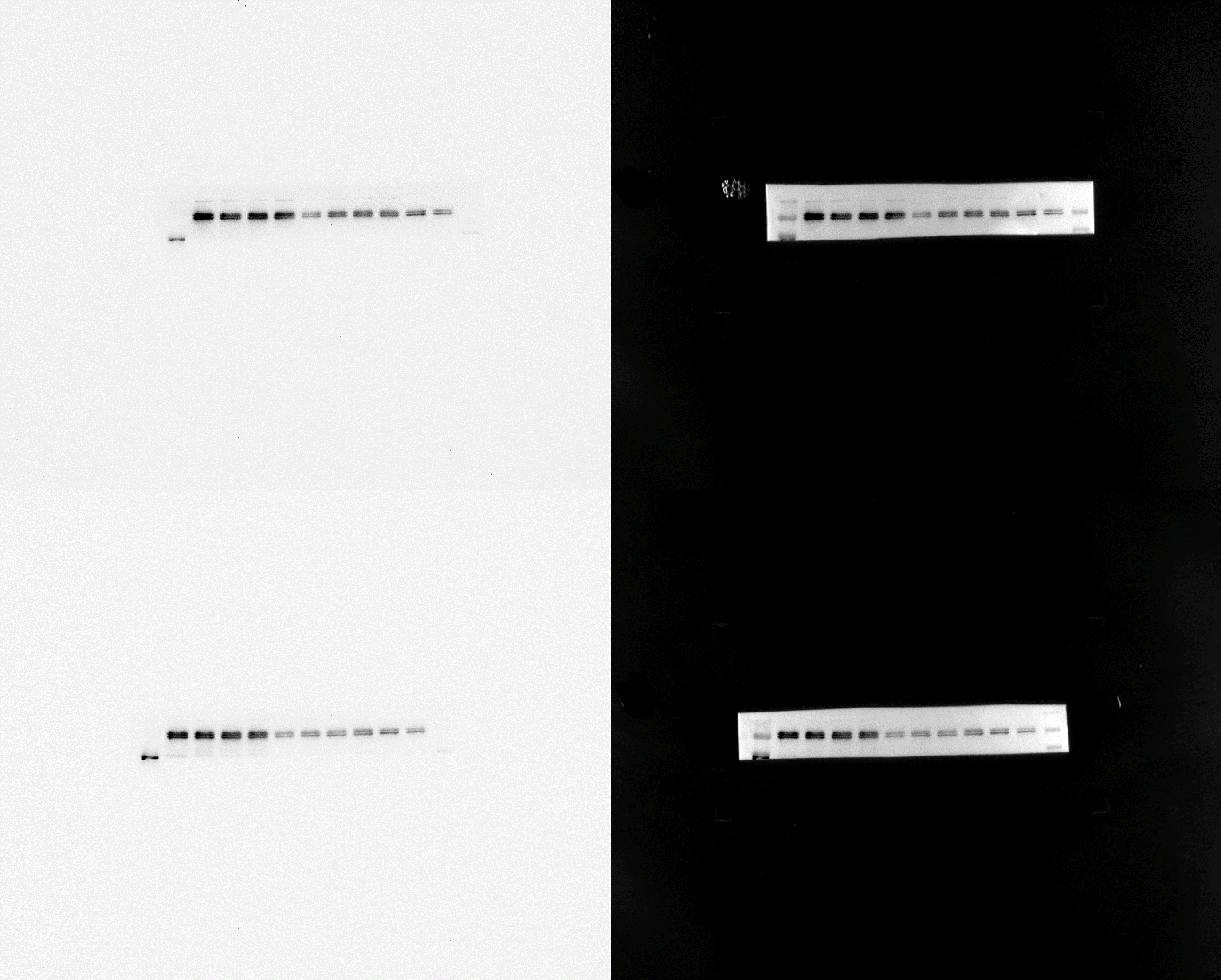

Supplement: Figure 4—source data 1. [file elife-96161-fig4-data1.zip › Figure 4-Source data1/Figure4G-Source data2-FOXO1.png]

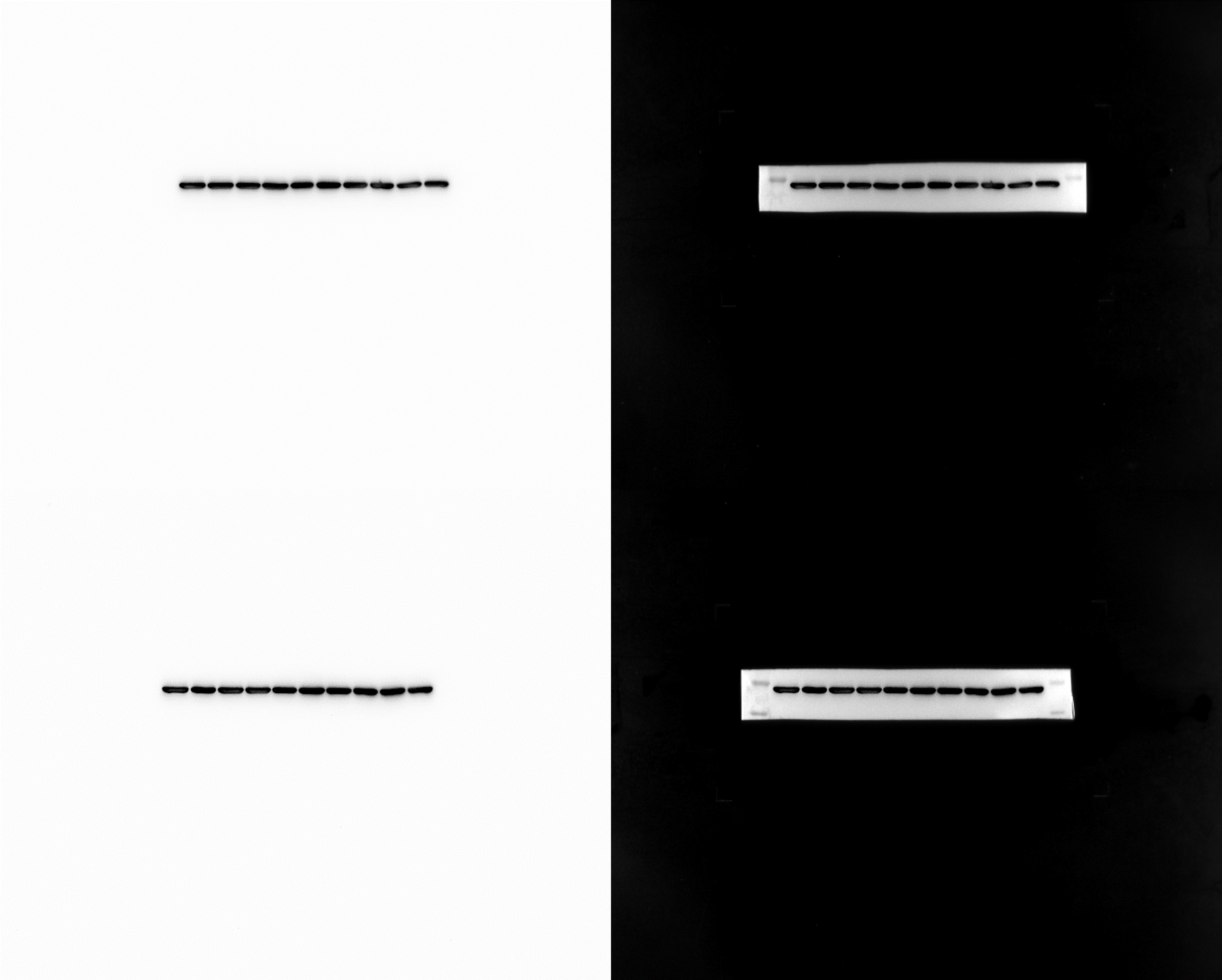

Supplement: Figure 4—source data 1. [file elife-96161-fig4-data1.zip › Figure 4-Source data1/Figure4G-Source data2-a┬-actin.png]

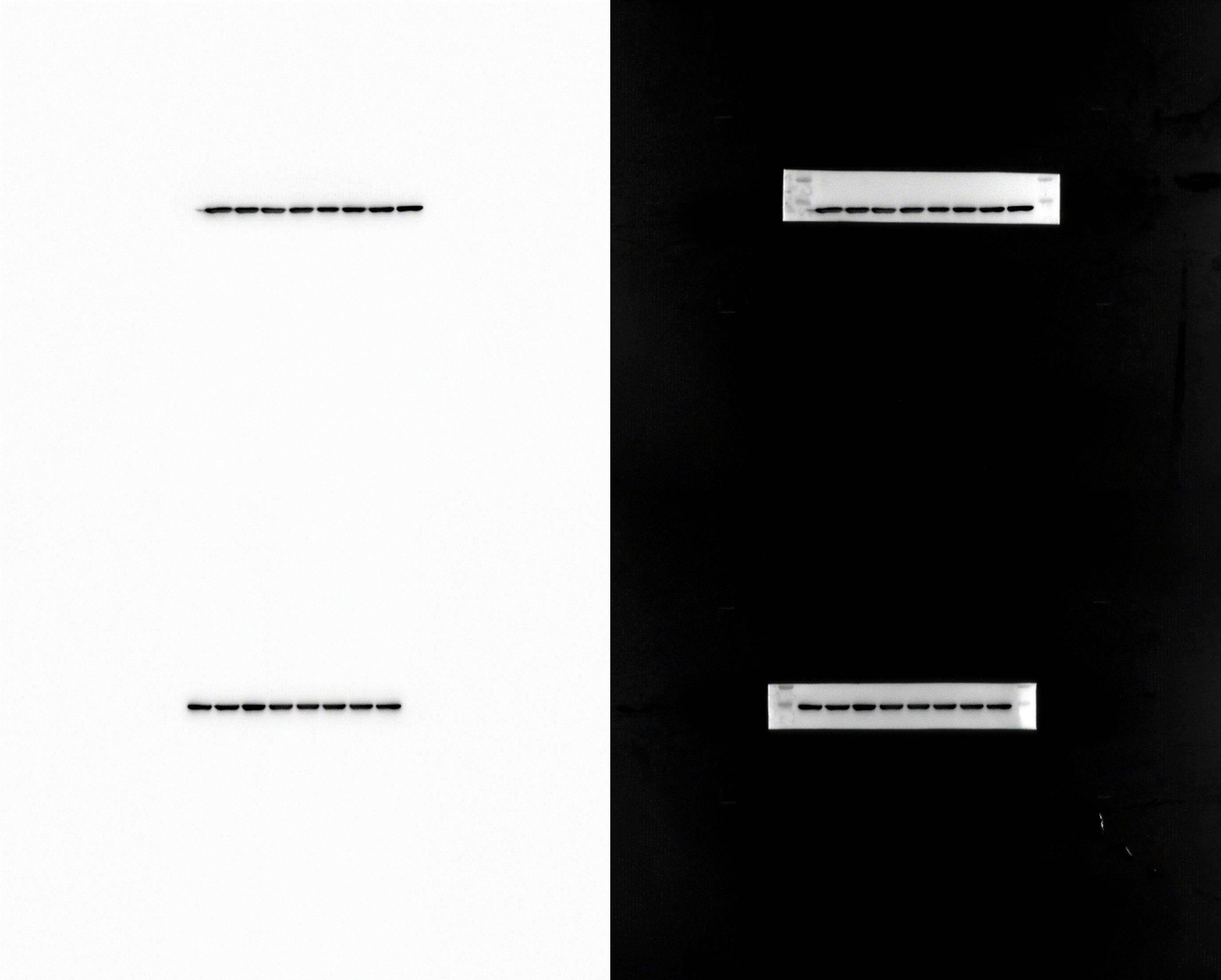

Supplement: Figure 4—figure supplement 1—source data 1. [file elife-96161-fig4-figsupp1-data1.zip › Figure 4α╕üα╕èfigure supplement 1C-Source data1-α╕åα╕ó-actin.png]

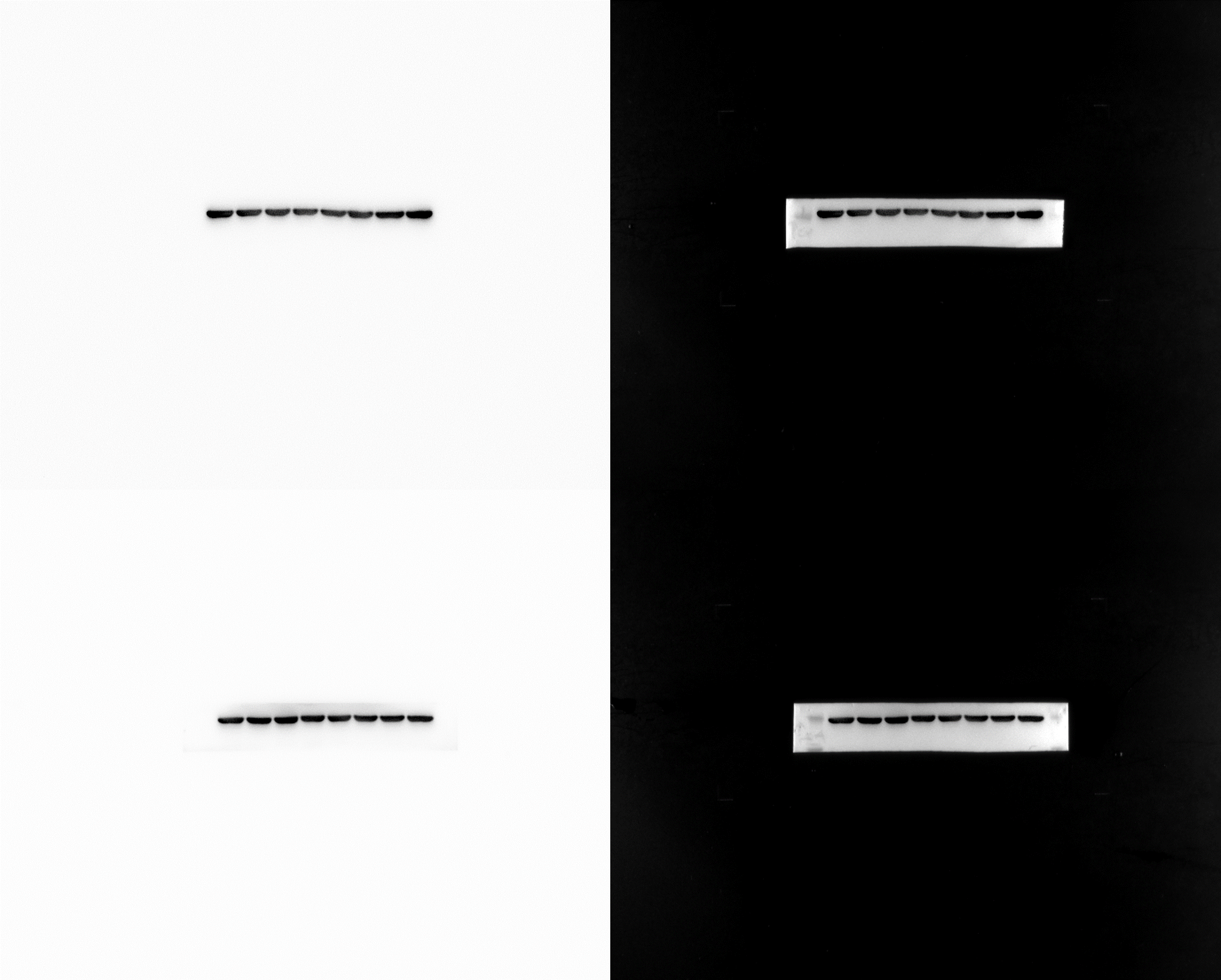

Supplement: Figure 4—figure supplement 1—source data 1. [file elife-96161-fig4-figsupp1-data1.zip › Figure 4α╕üα╕èfigure supplement 1C-Source data2-α╕åα╕ó-actin.png]

Figure 4—figure supplement 1C-Claudin-5

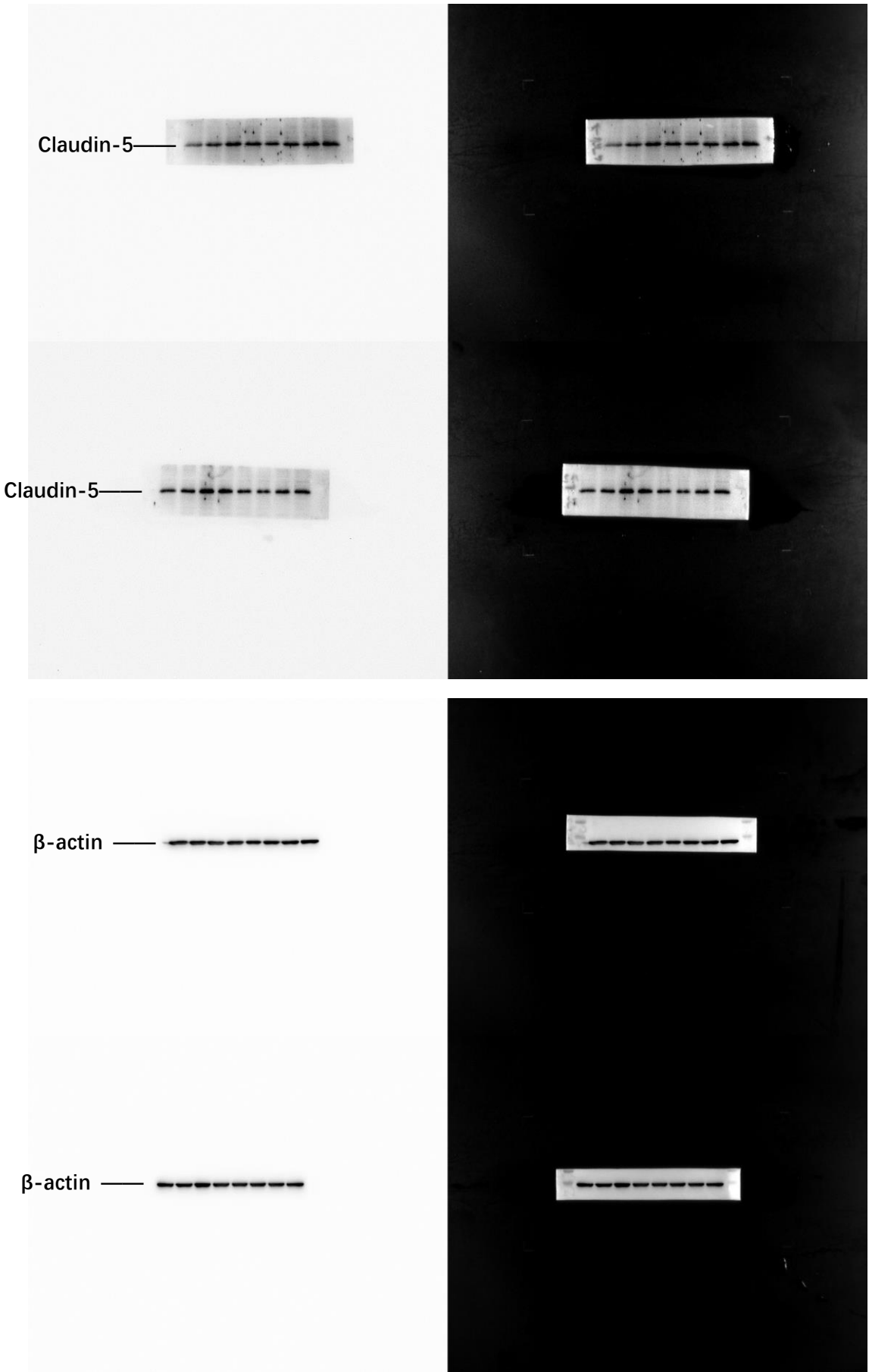

Figure 4—figure supplement 1C-VE-cadherin

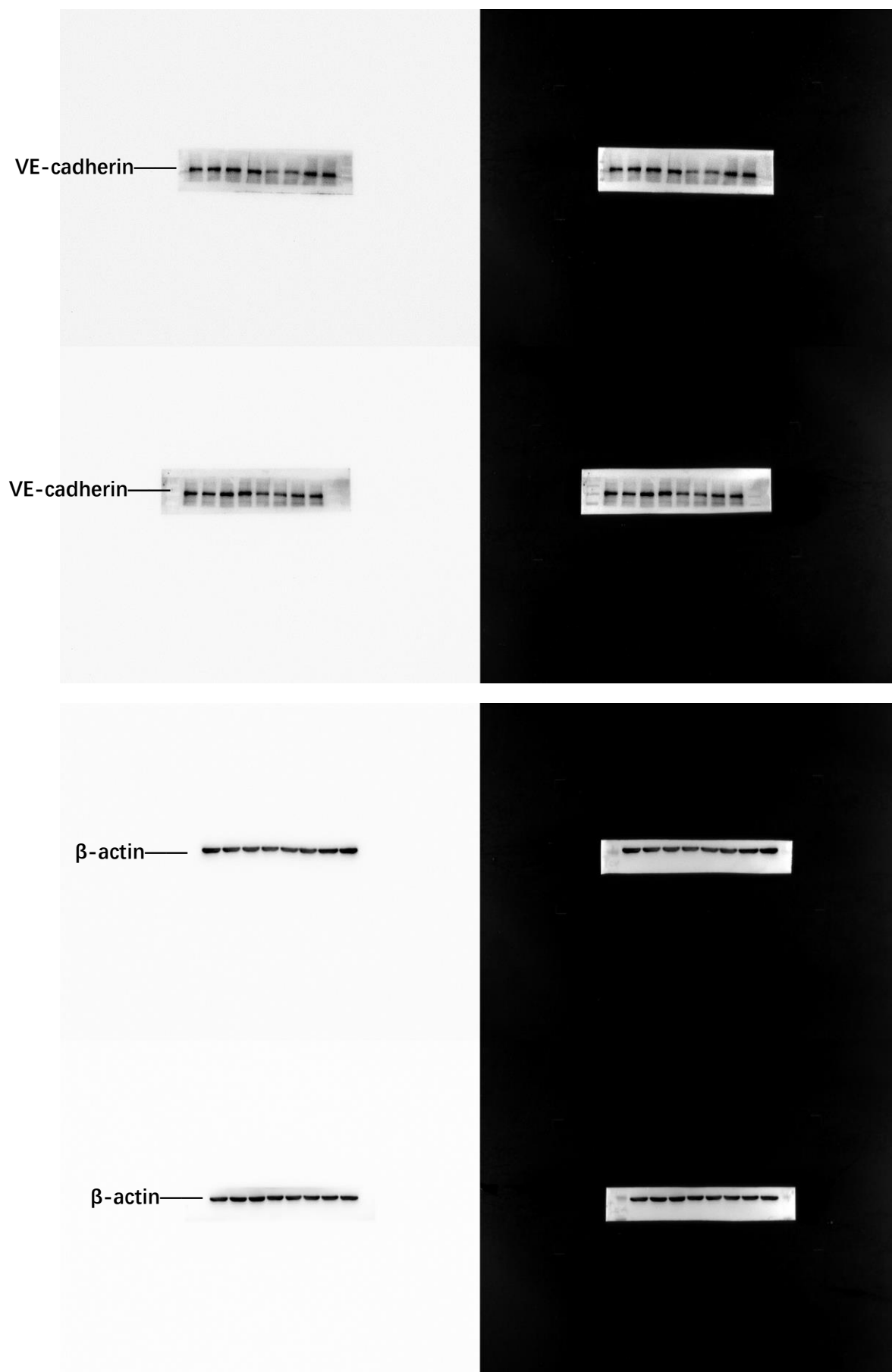

Supplement: Figure 4—figure supplement 1—source data 2. [file elife-96161-fig4-figsupp1-data2.zip › Figure 4í¬figure supplement 1-Source data2/Figure 4í¬figure supplement 1-Annotated western blots.pdf]

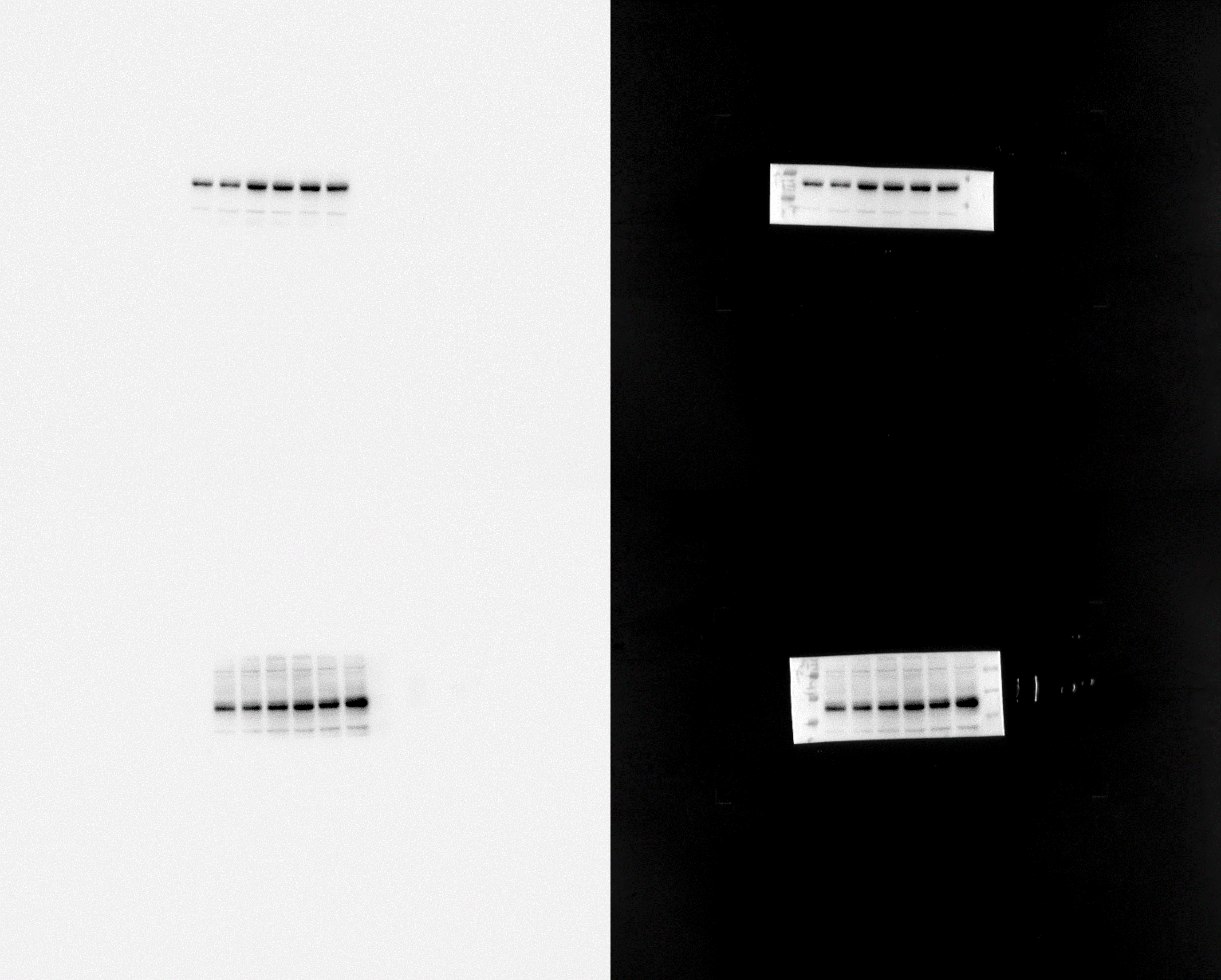

Supplement: Figure 5—source data 1. [file elife-96161-fig5-data1.zip › Figure 5-Source data1/Figure5A-Source data-ETS1.png]

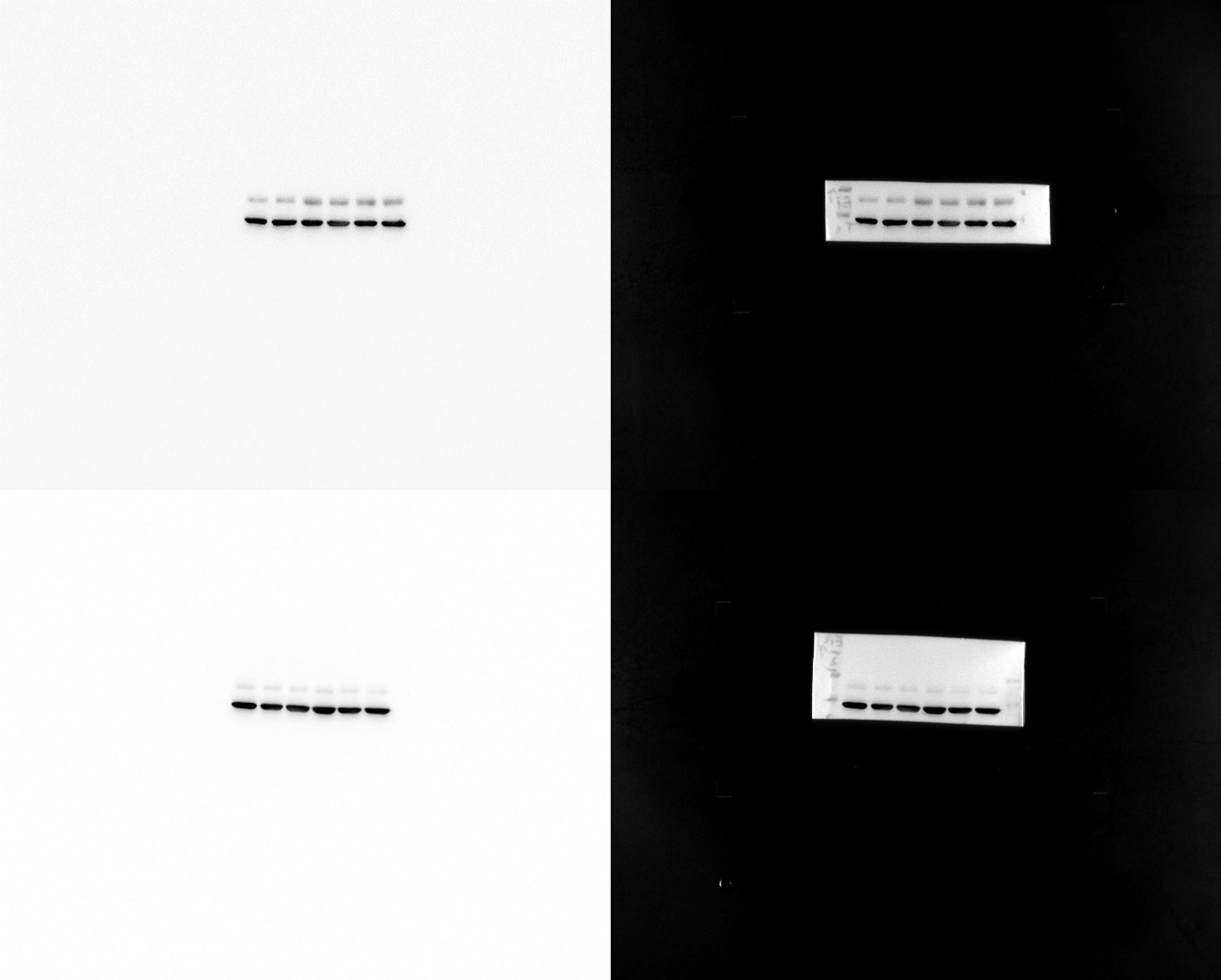

Supplement: Figure 5—source data 1. [file elife-96161-fig5-data1.zip › Figure 5-Source data1/Figure5A-Source data-a┬-actin.png]

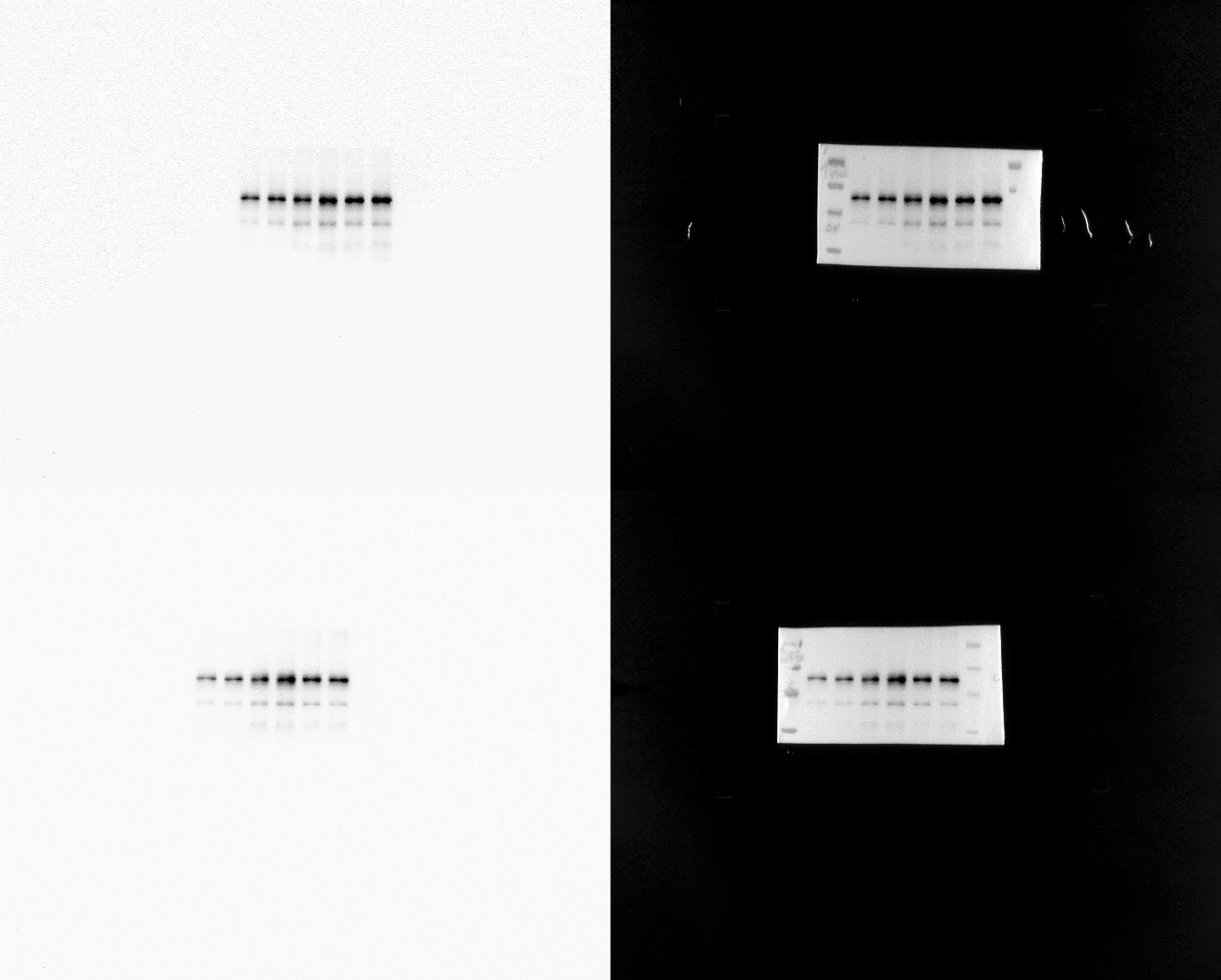

Supplement: Figure 5—source data 1. [file elife-96161-fig5-data1.zip › Figure 5-Source data1/Figure5B-Source data-ETS1.png]

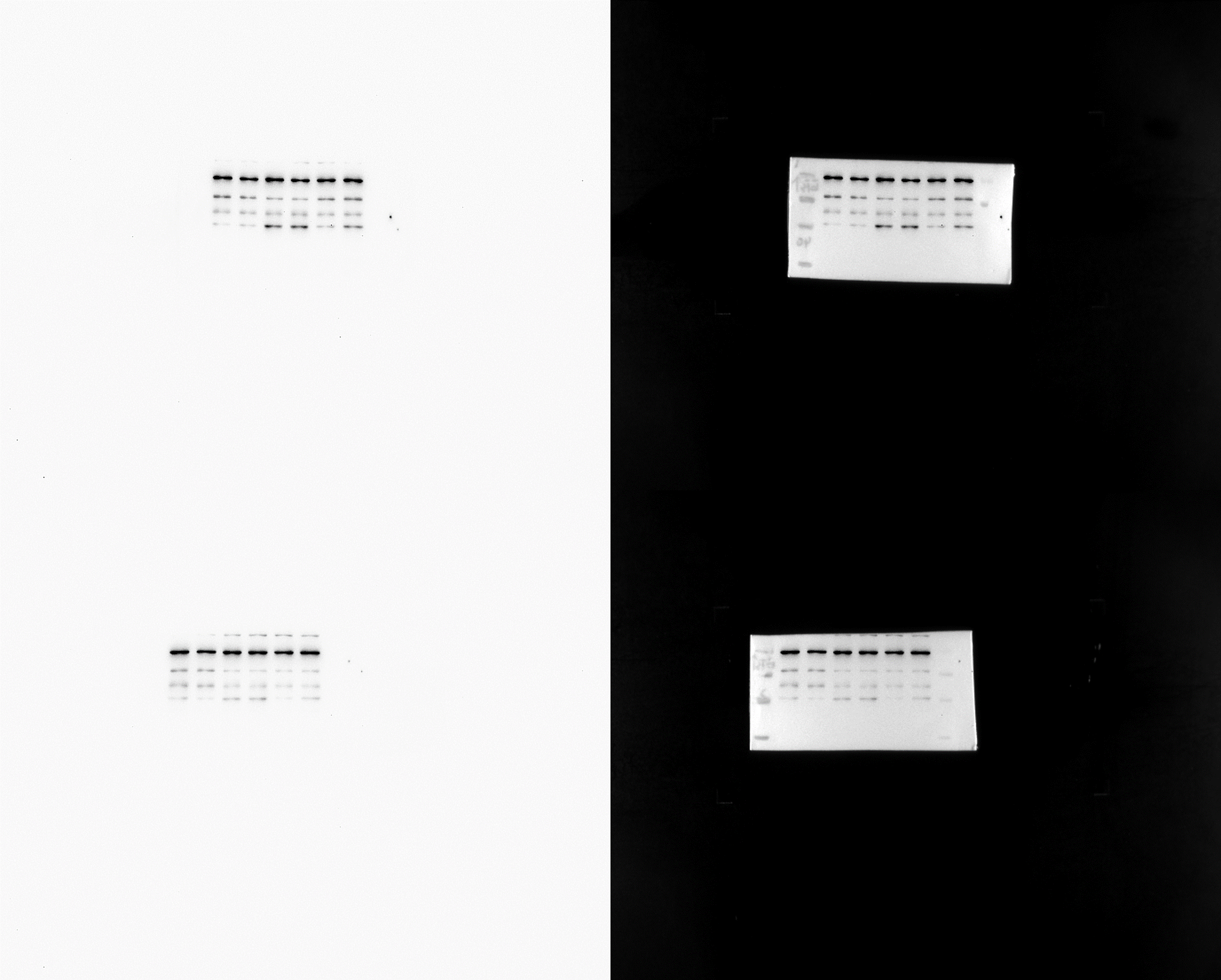

Supplement: Figure 5—source data 1. [file elife-96161-fig5-data1.zip › Figure 5-Source data1/Figure5B-Source data-Lamin B.png]

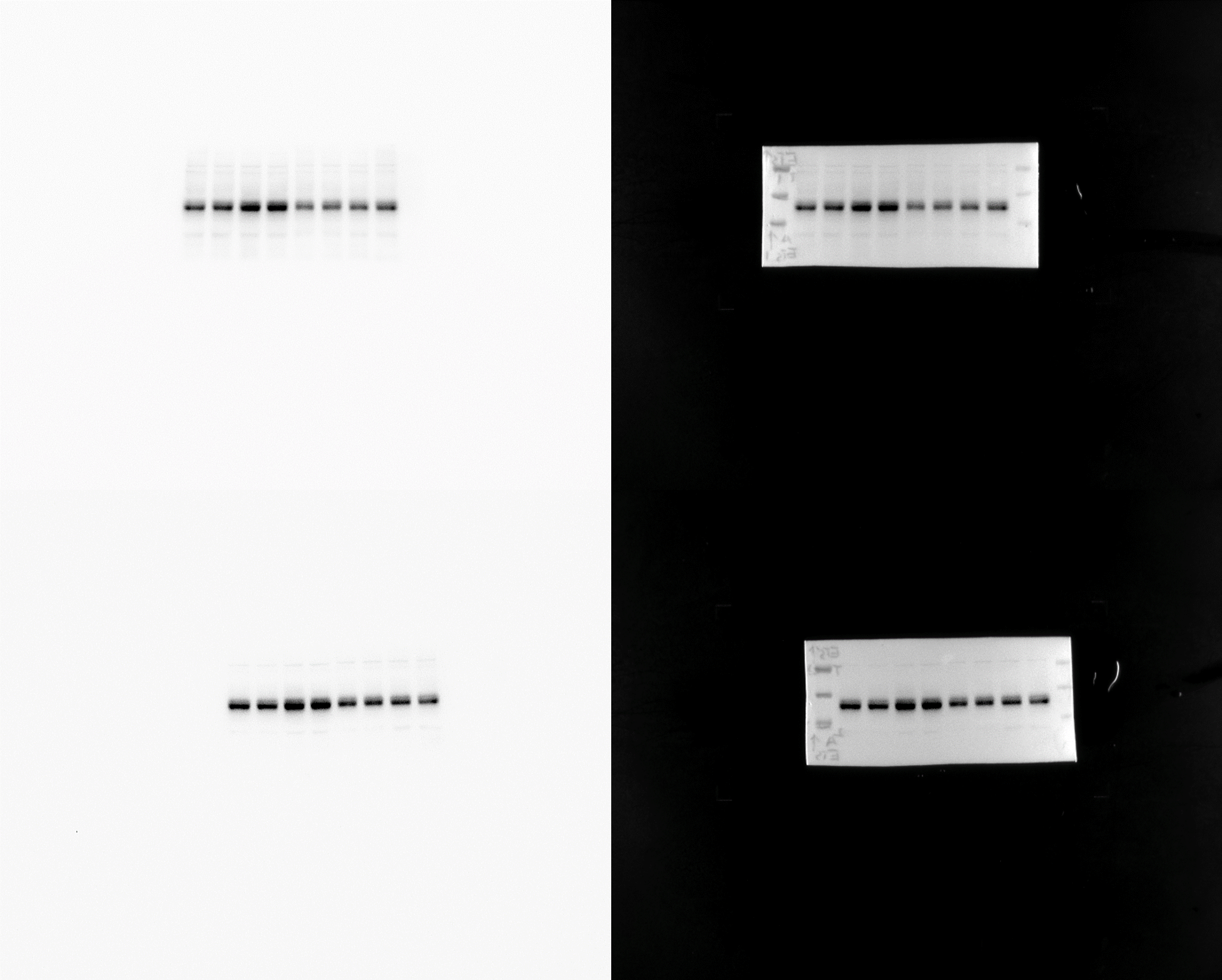

Supplement: Figure 5—source data 1. [file elife-96161-fig5-data1.zip › Figure 5-Source data1/Figure5C-Source data-ETS1.png]

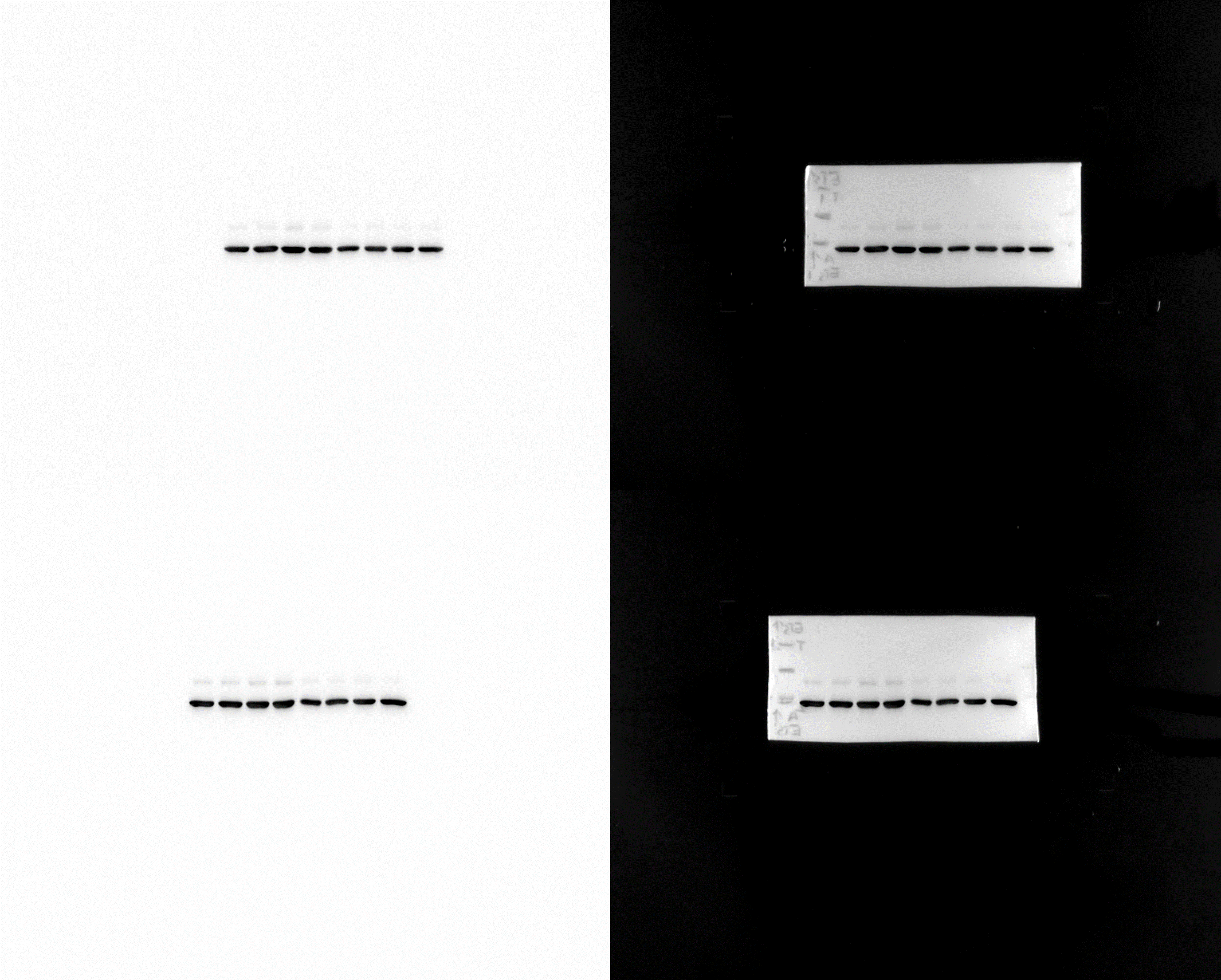

Supplement: Figure 5—source data 1. [file elife-96161-fig5-data1.zip › Figure 5-Source data1/Figure5C-Source data-a┬-actin.png]

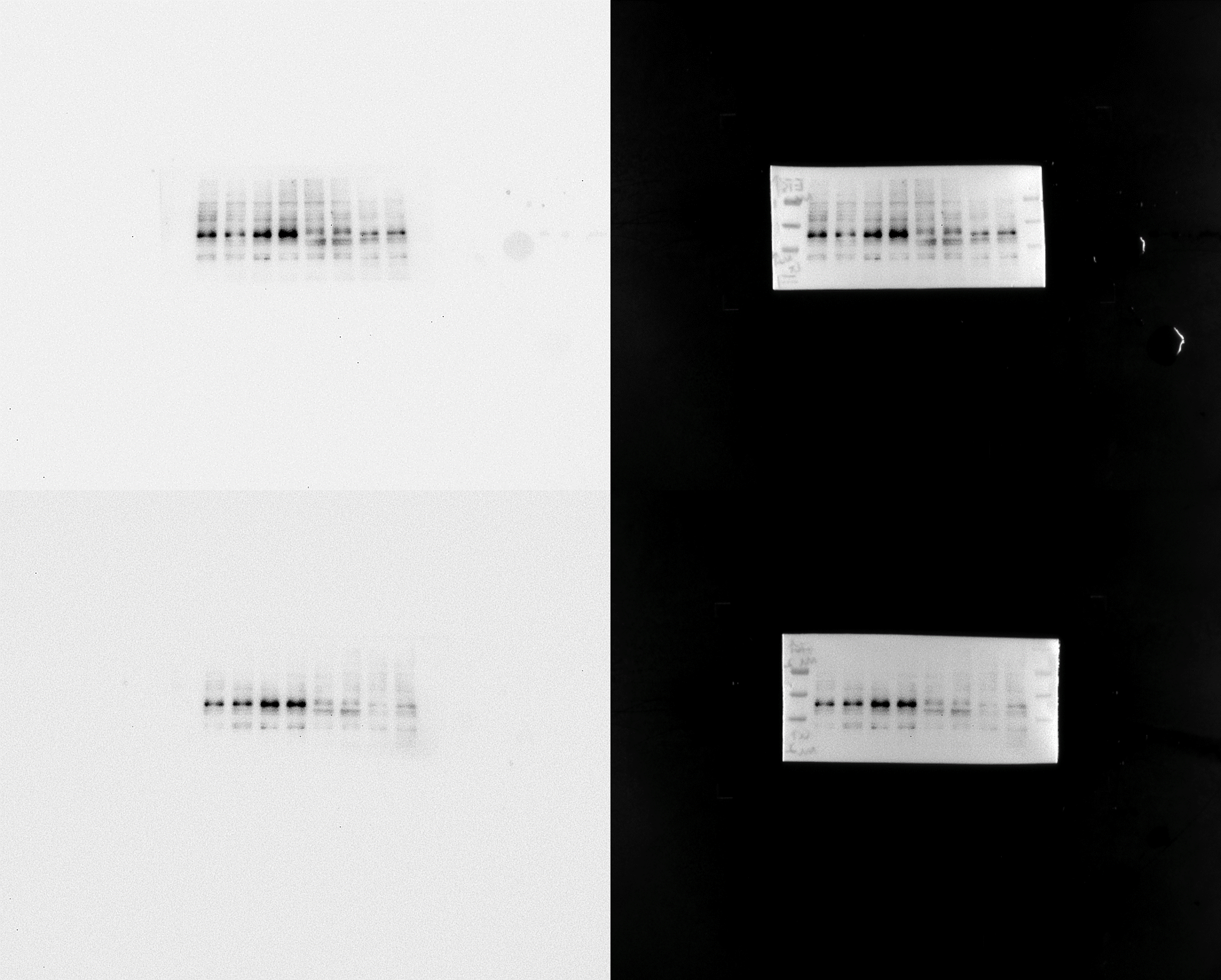

Supplement: Figure 5—source data 1. [file elife-96161-fig5-data1.zip › Figure 5-Source data1/Figure5D-Source data-ETS1.png]

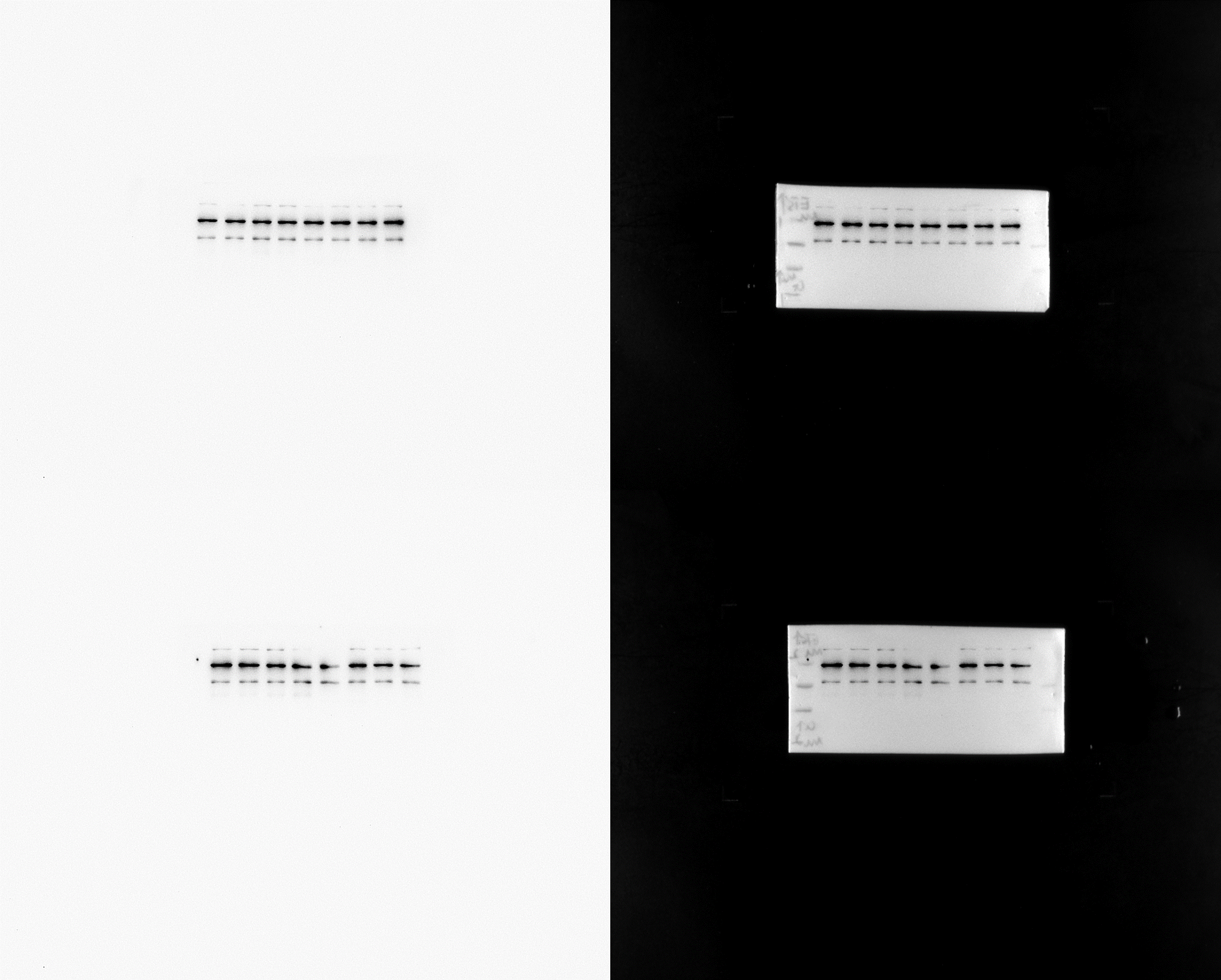

Supplement: Figure 5—source data 1. [file elife-96161-fig5-data1.zip › Figure 5-Source data1/Figure5D-Source data-Lamin B.png]

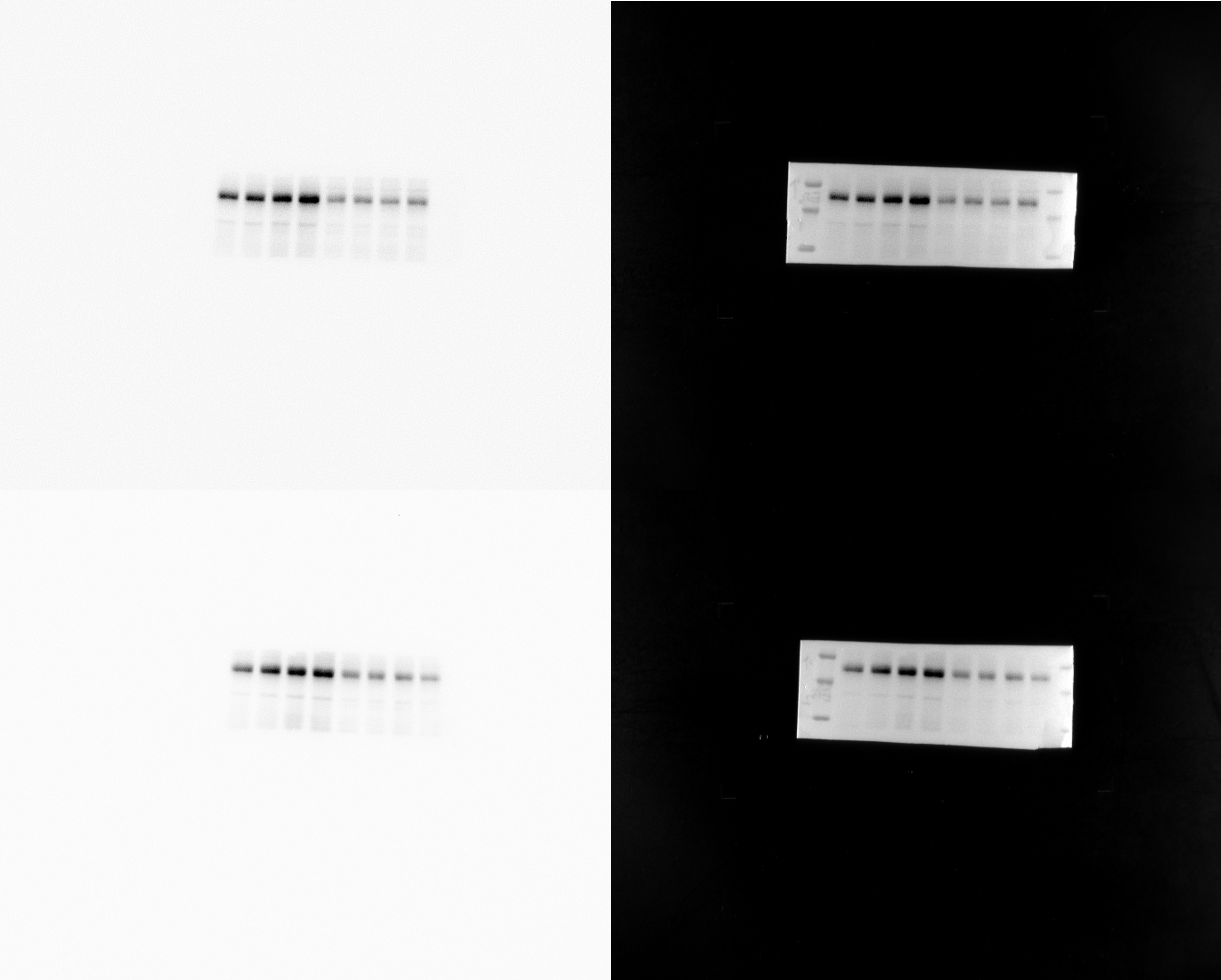

Supplement: Figure 5—source data 1. [file elife-96161-fig5-data1.zip › Figure 5-Source data1/Figure5E-Source data-ETS1.png]

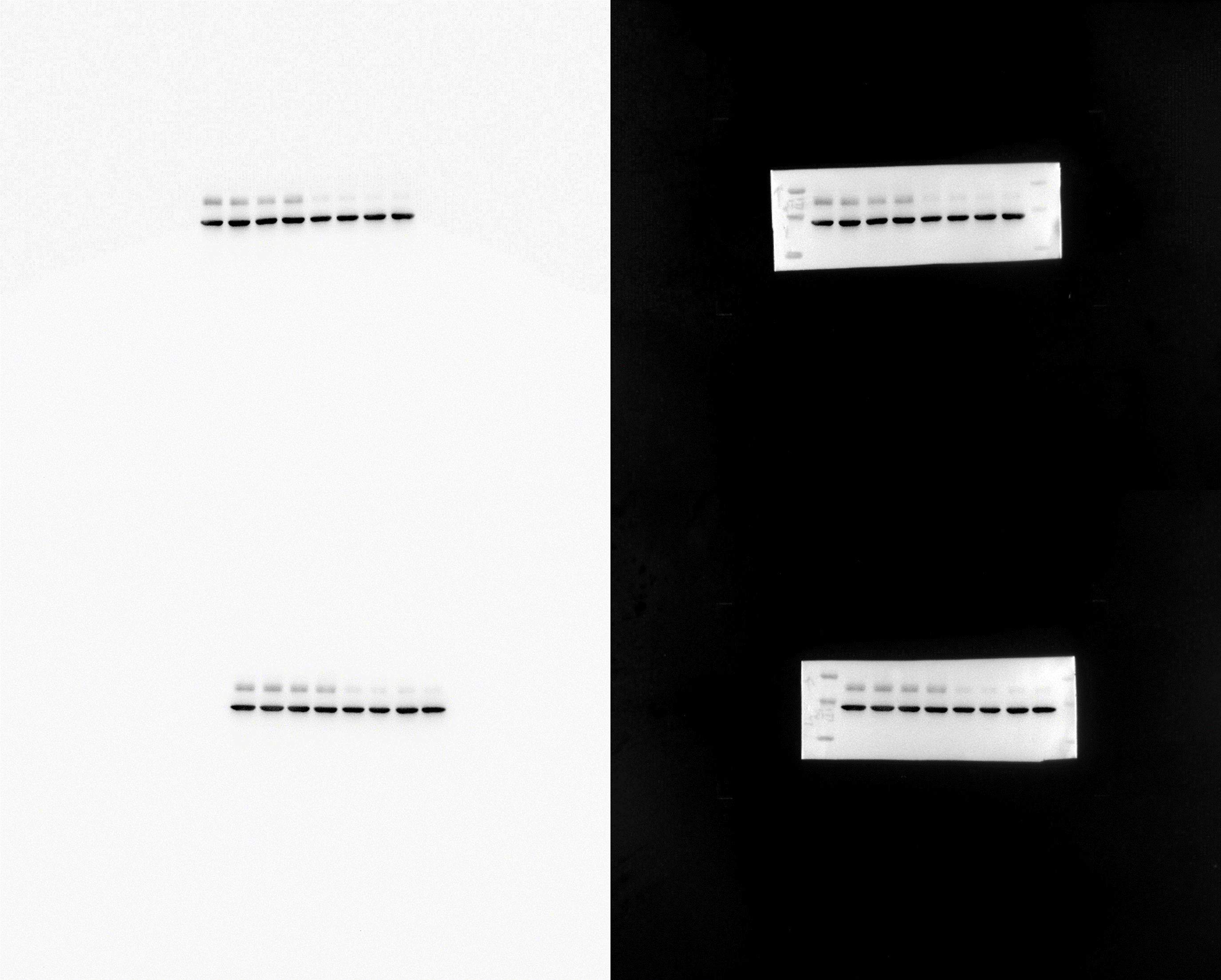

Supplement: Figure 5—source data 1. [file elife-96161-fig5-data1.zip › Figure 5-Source data1/Figure5E-Source data-a┬-actin.png]

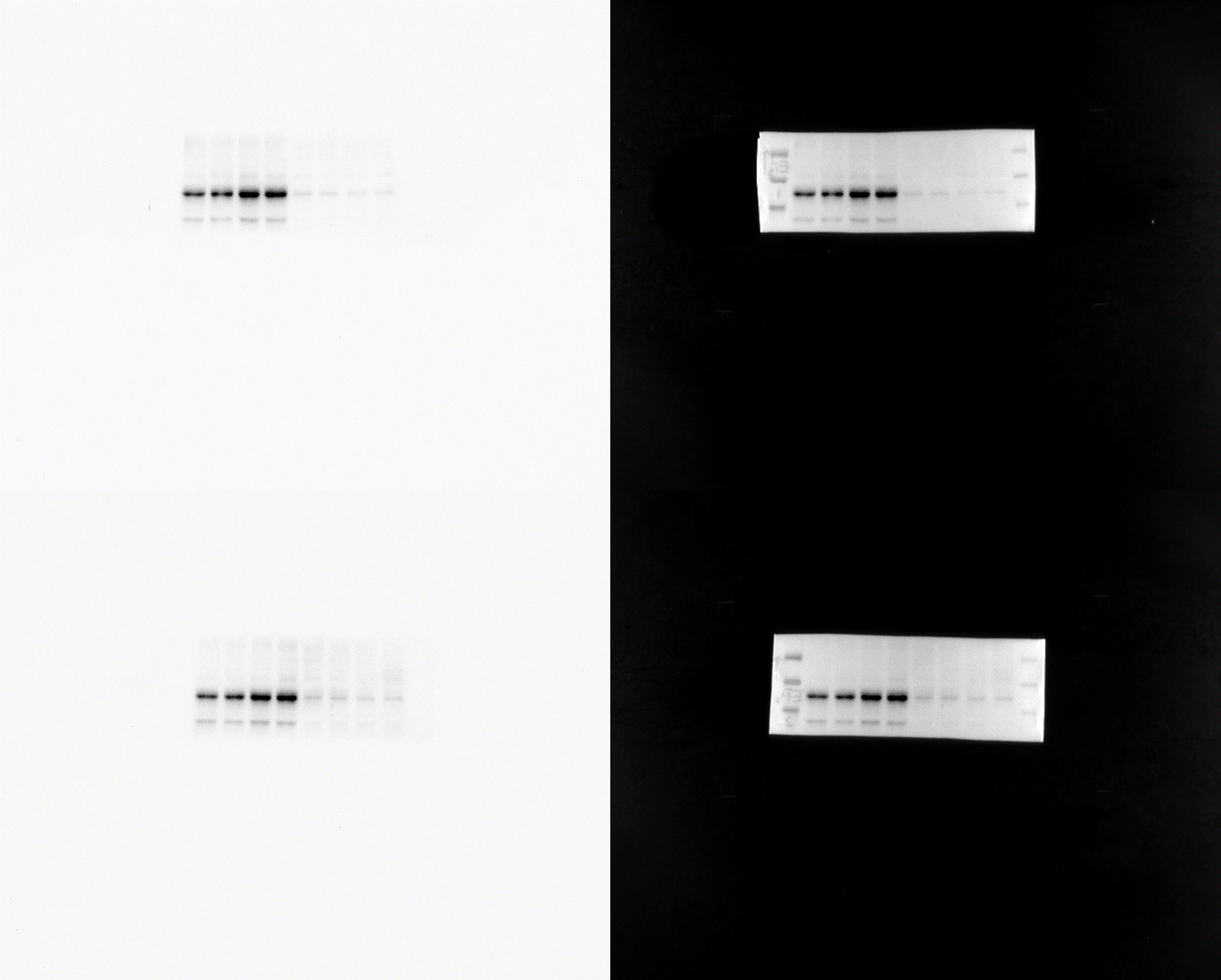

Supplement: Figure 5—source data 1. [file elife-96161-fig5-data1.zip › Figure 5-Source data1/Figure5F-Source data-ETS1.png]

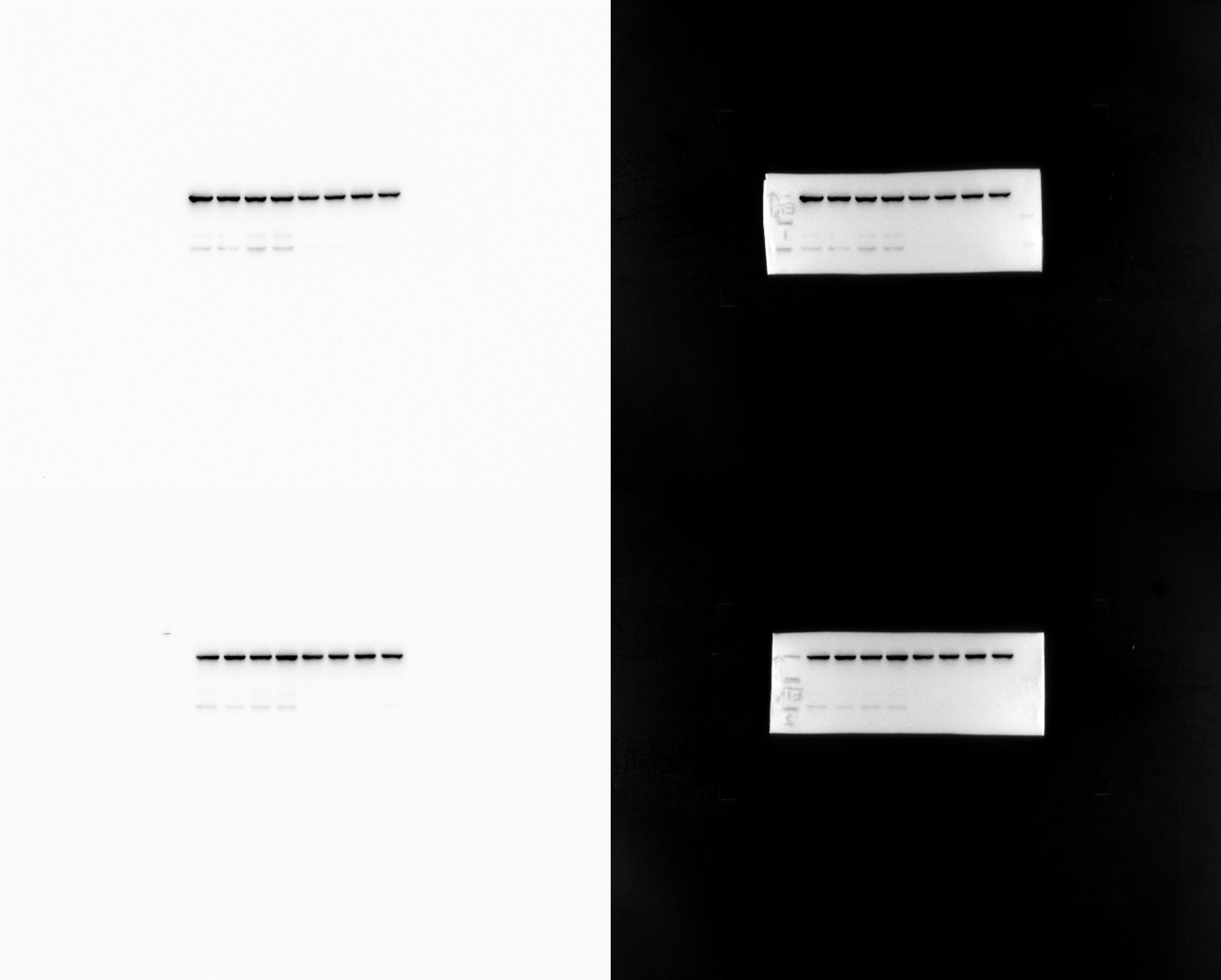

Supplement: Figure 5—source data 1. [file elife-96161-fig5-data1.zip › Figure 5-Source data1/Figure5F-Source data-Lamin B.png]

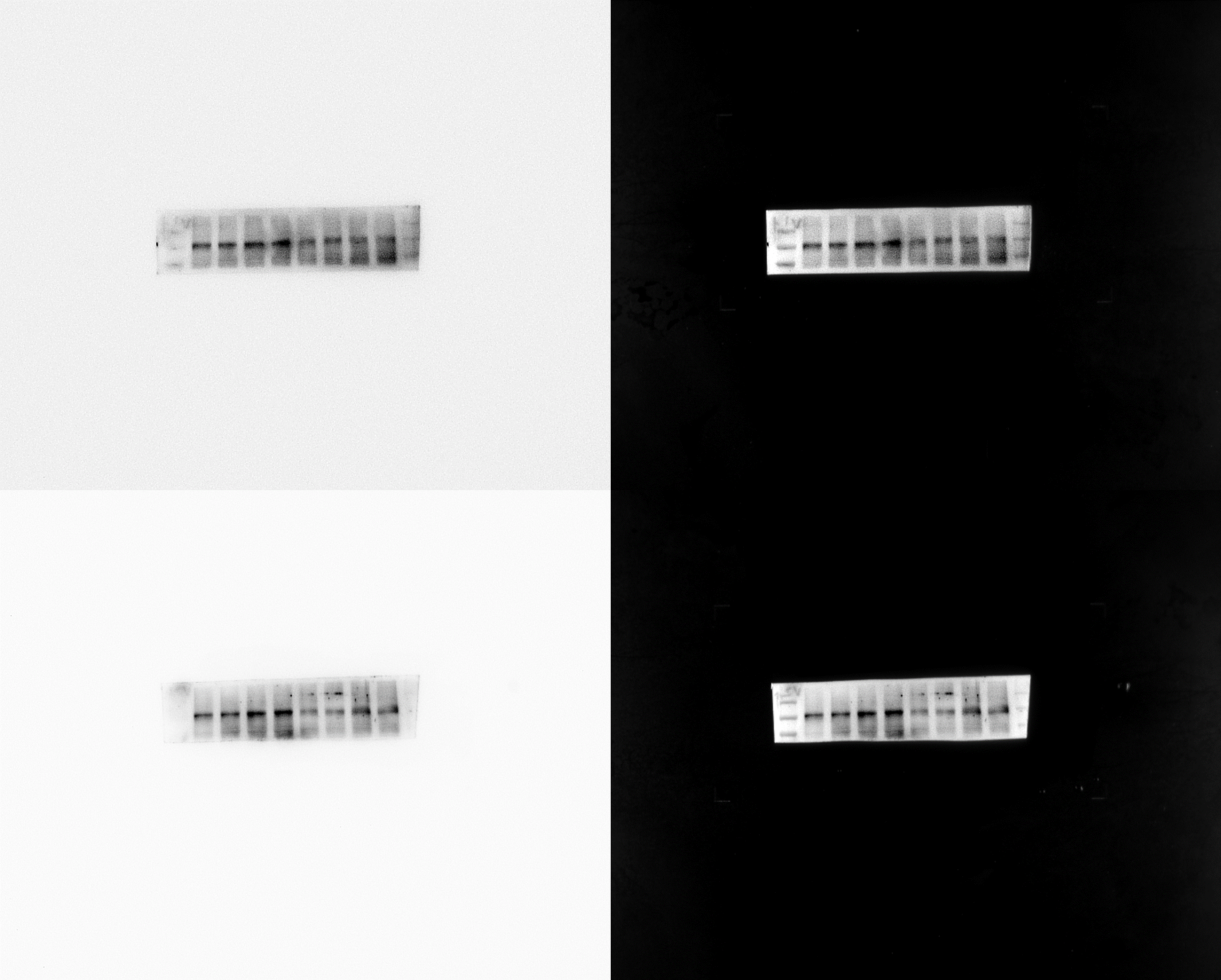

Supplement: Figure 5—source data 1. [file elife-96161-fig5-data1.zip › Figure 5-Source data1/Figure5G-Source data1-VE-Cadherin.png]

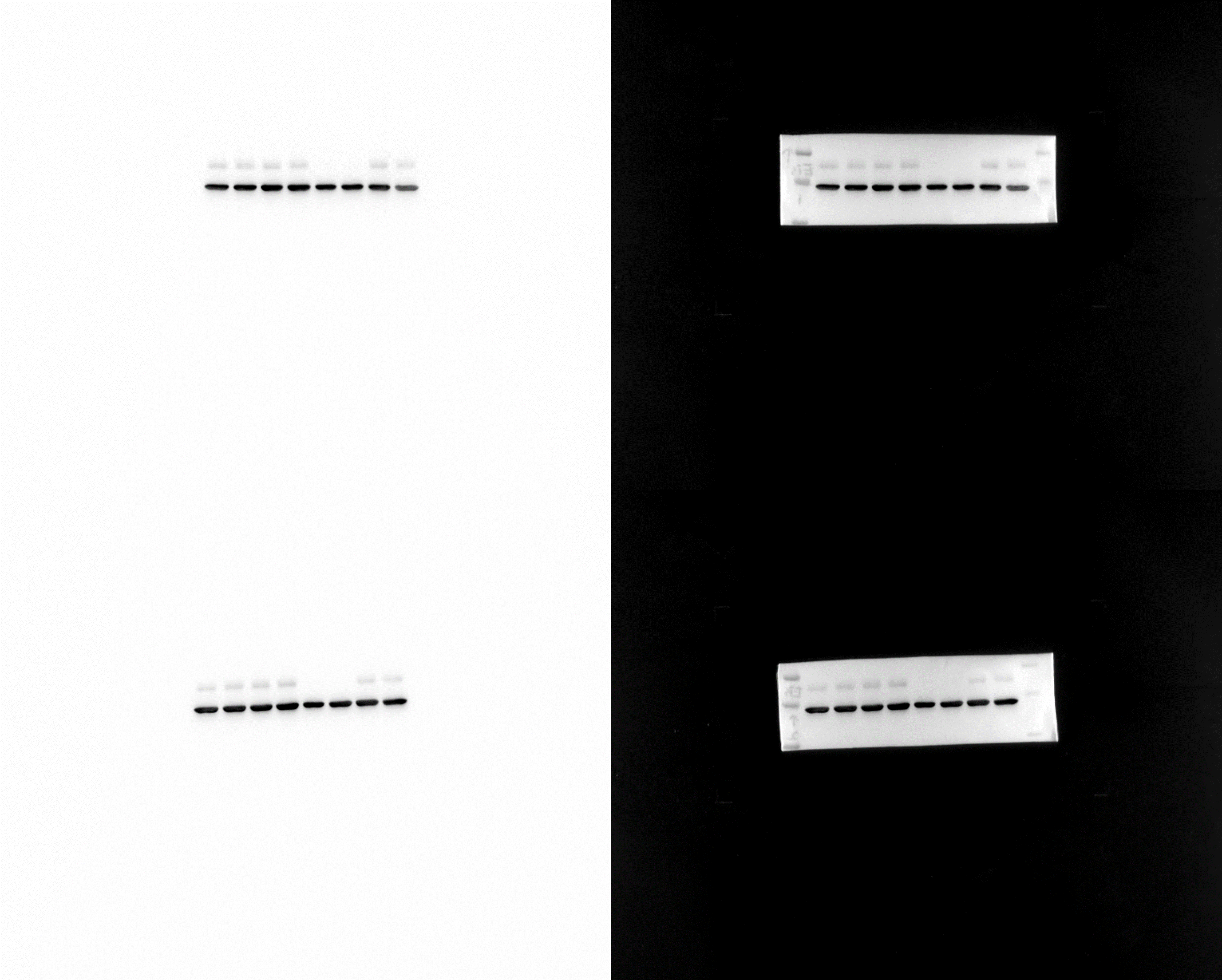

Supplement: Figure 5—source data 1. [file elife-96161-fig5-data1.zip › Figure 5-Source data1/Figure5G-Source data1-a┬-actin.png]

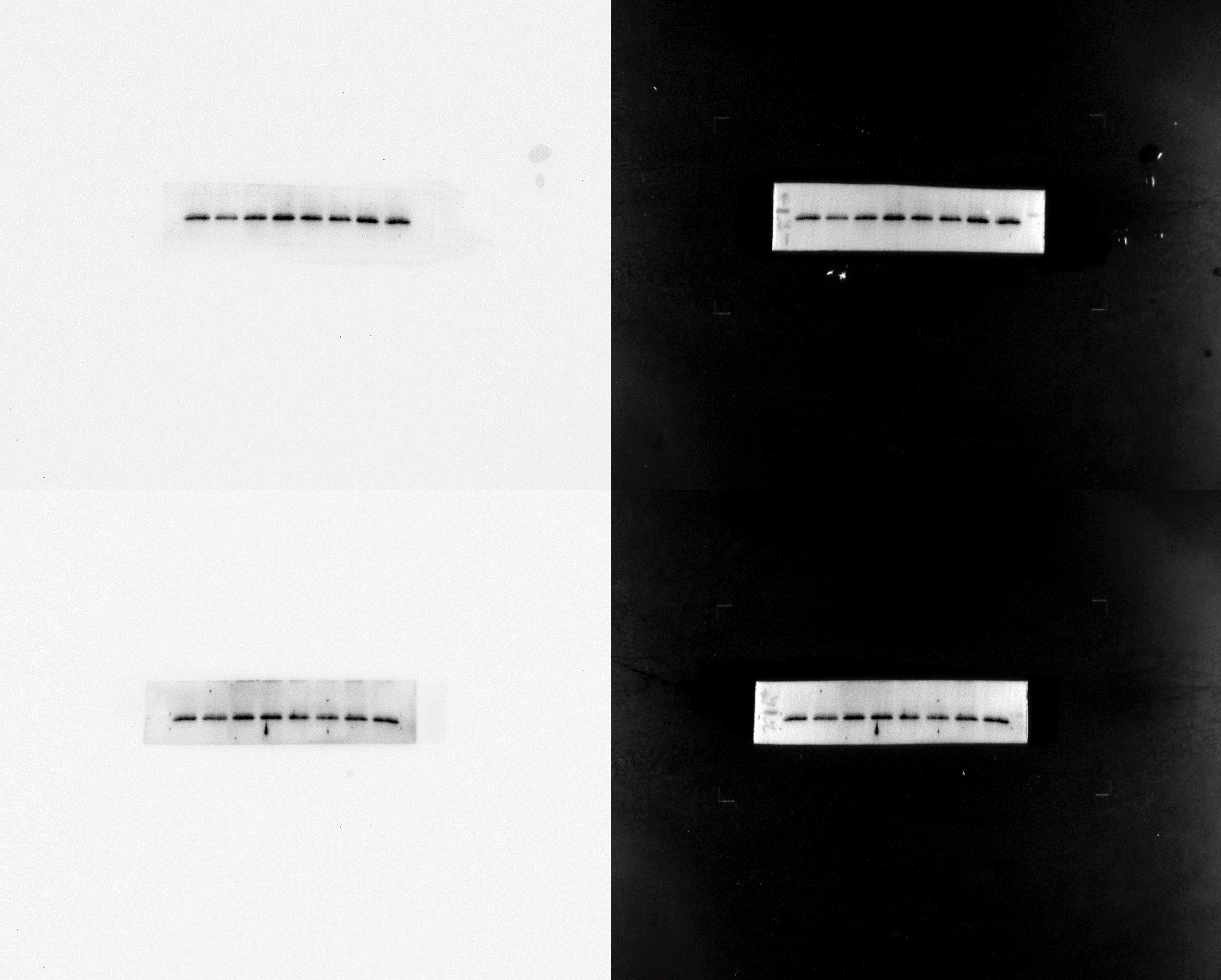

Supplement: Figure 5—source data 1. [file elife-96161-fig5-data1.zip › Figure 5-Source data1/Figure5G-Source data2-Claudin-5.png]

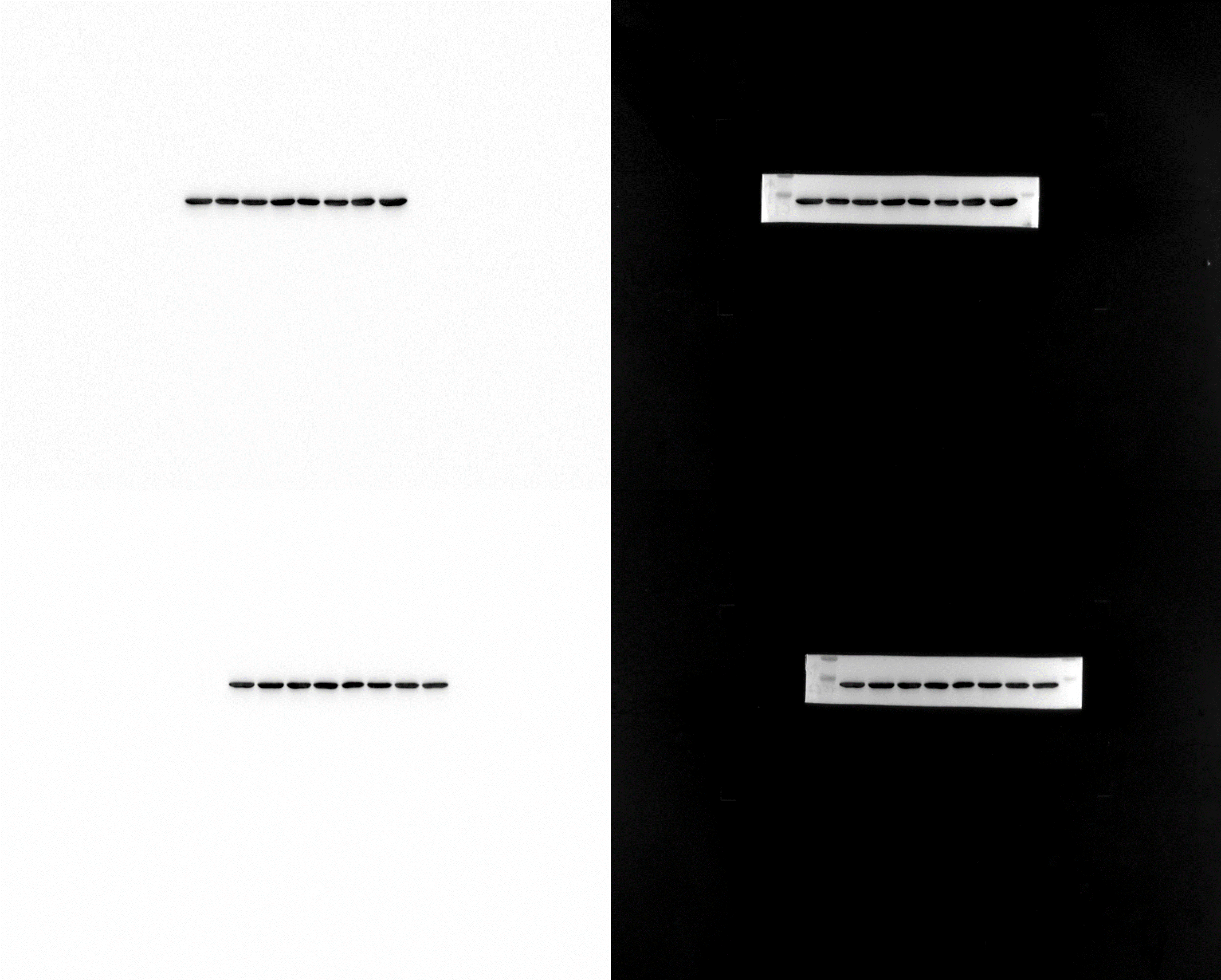

Supplement: Figure 5—source data 1. [file elife-96161-fig5-data1.zip › Figure 5-Source data1/Figure5G-Source data2-a┬-actin.png]

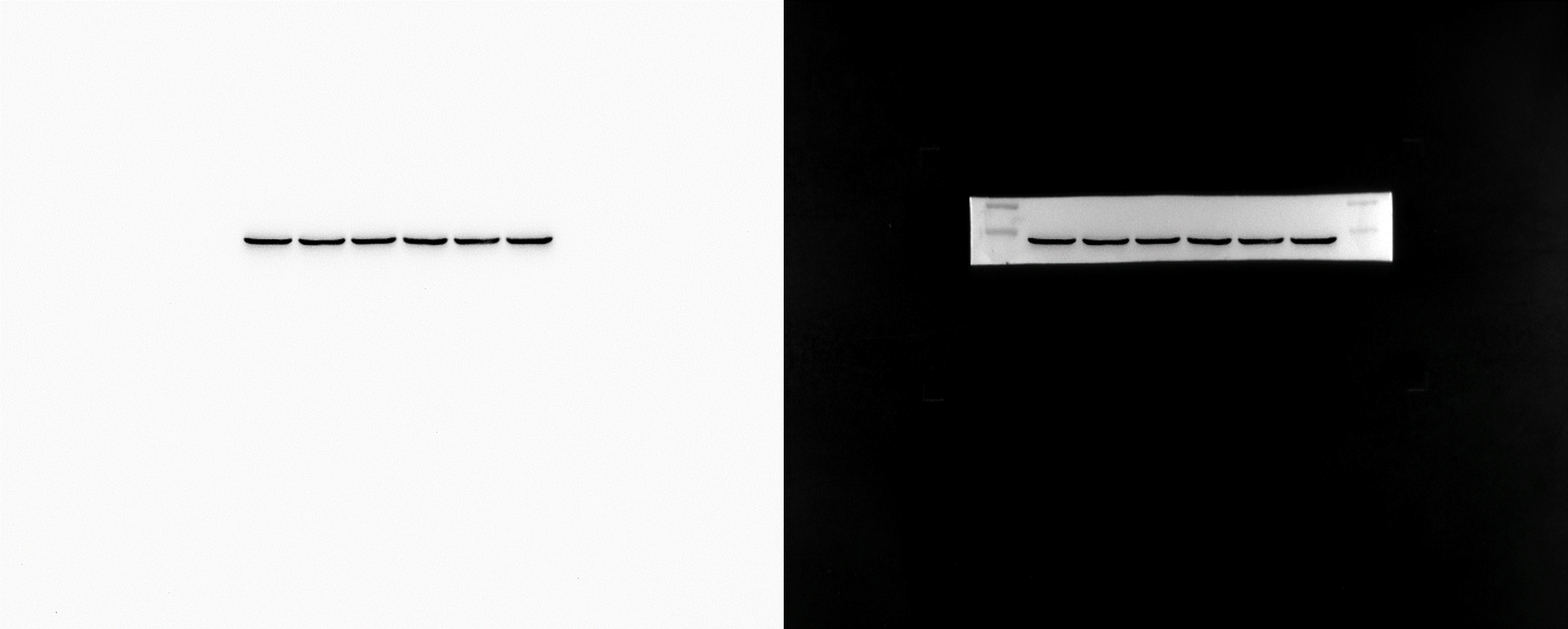

Supplement: Figure 6—source data 1. [file elife-96161-fig6-data1.zip › Figure 6-Source data1/Figure6B-Source data1,2-a┬-actin(Claudin-5,GDNF).png]

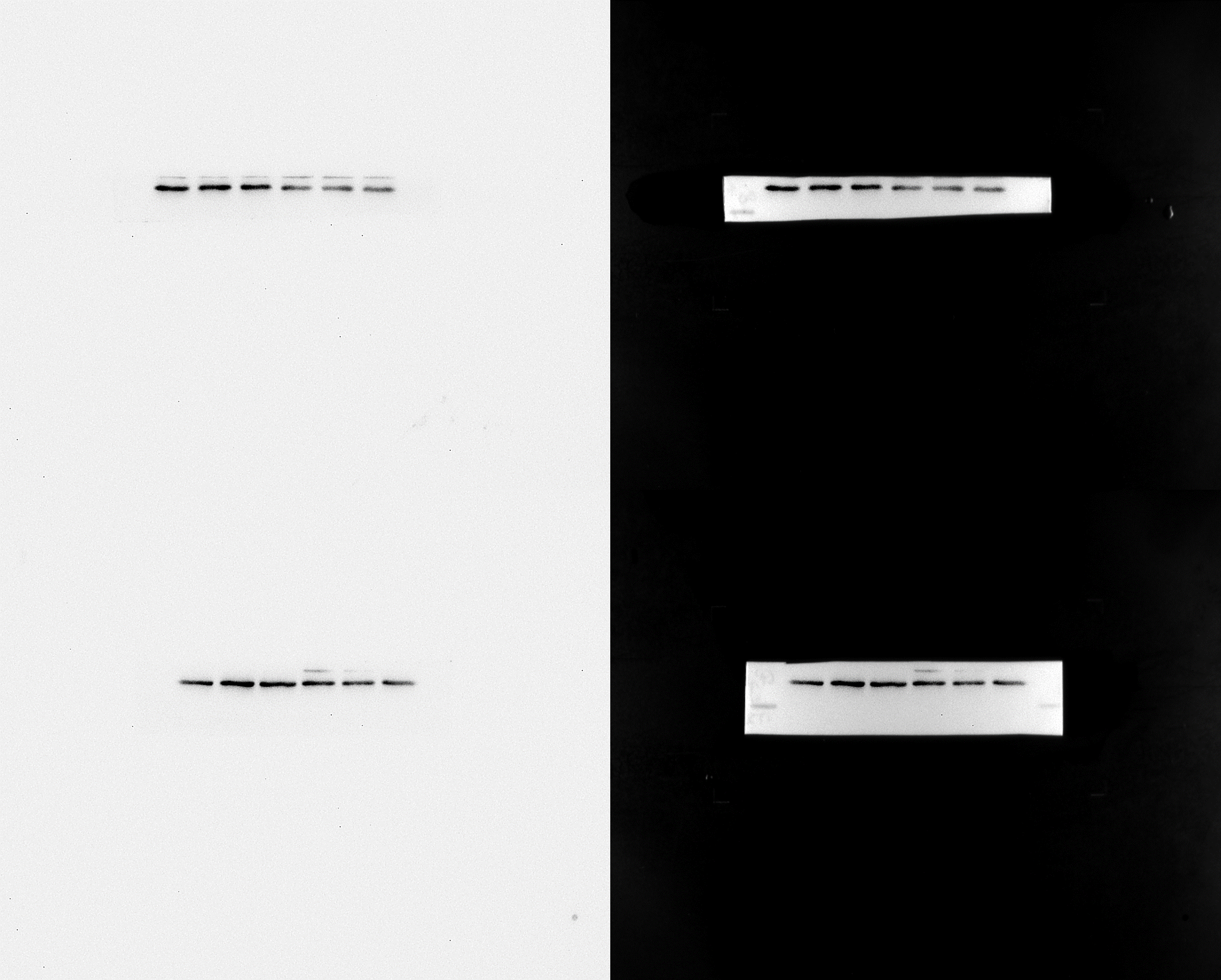

Supplement: Figure 6—source data 1. [file elife-96161-fig6-data1.zip › Figure 6-Source data1/Figure6B-Source data1-GDNF.png]

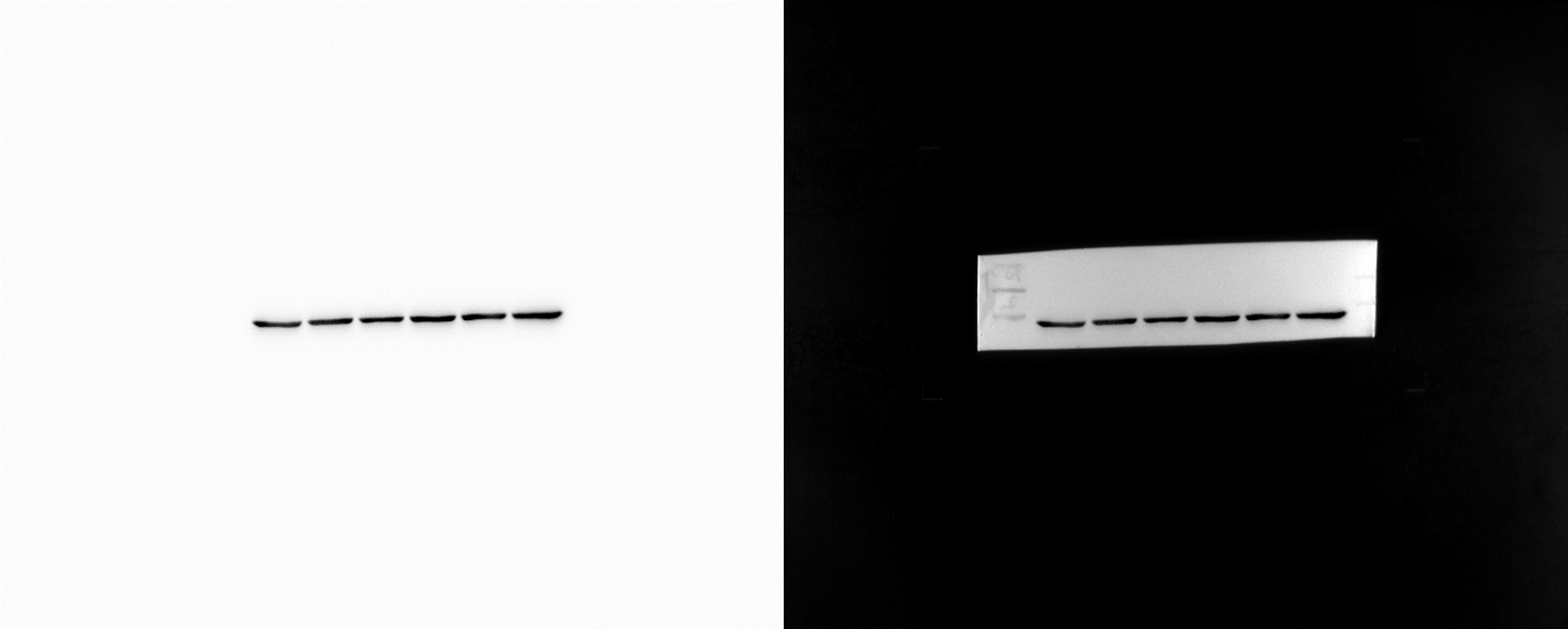

Supplement: Figure 6—source data 1. [file elife-96161-fig6-data1.zip › Figure 6-Source data1/Figure6B-Source data1-a┬-actin(GDNF).png]

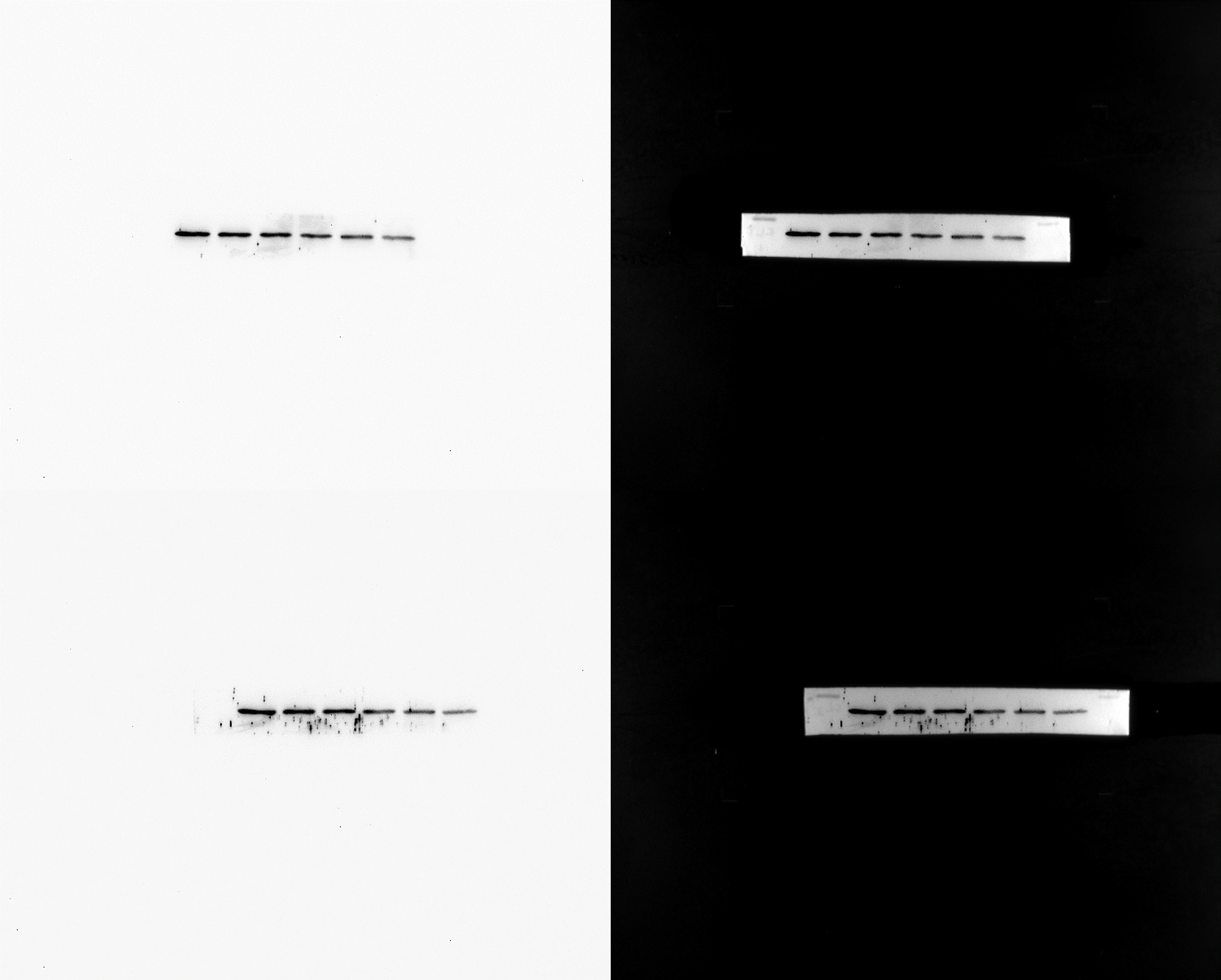

Supplement: Figure 6—source data 1. [file elife-96161-fig6-data1.zip › Figure 6-Source data1/Figure6B-Source data2-Claudin-5.png]

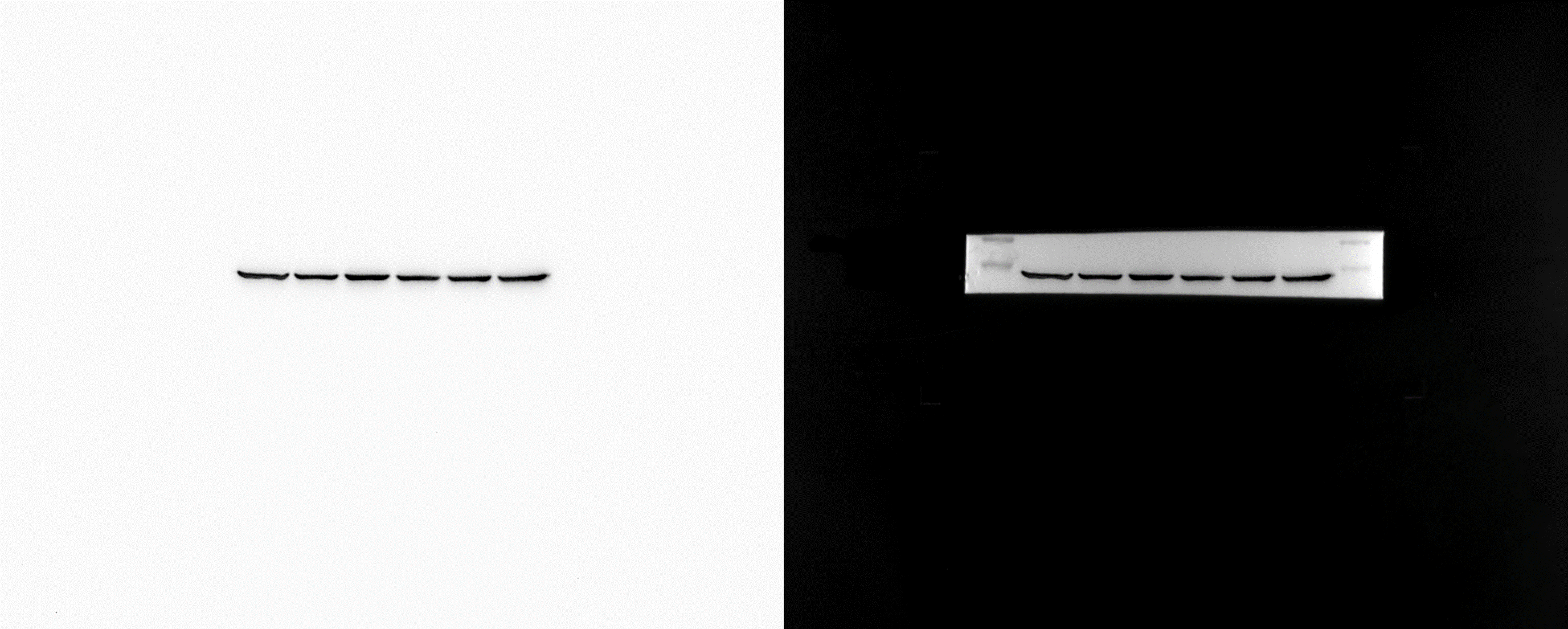

Supplement: Figure 6—source data 1. [file elife-96161-fig6-data1.zip › Figure 6-Source data1/Figure6B-Source data2-a┬-actin(Claudin-5).png]

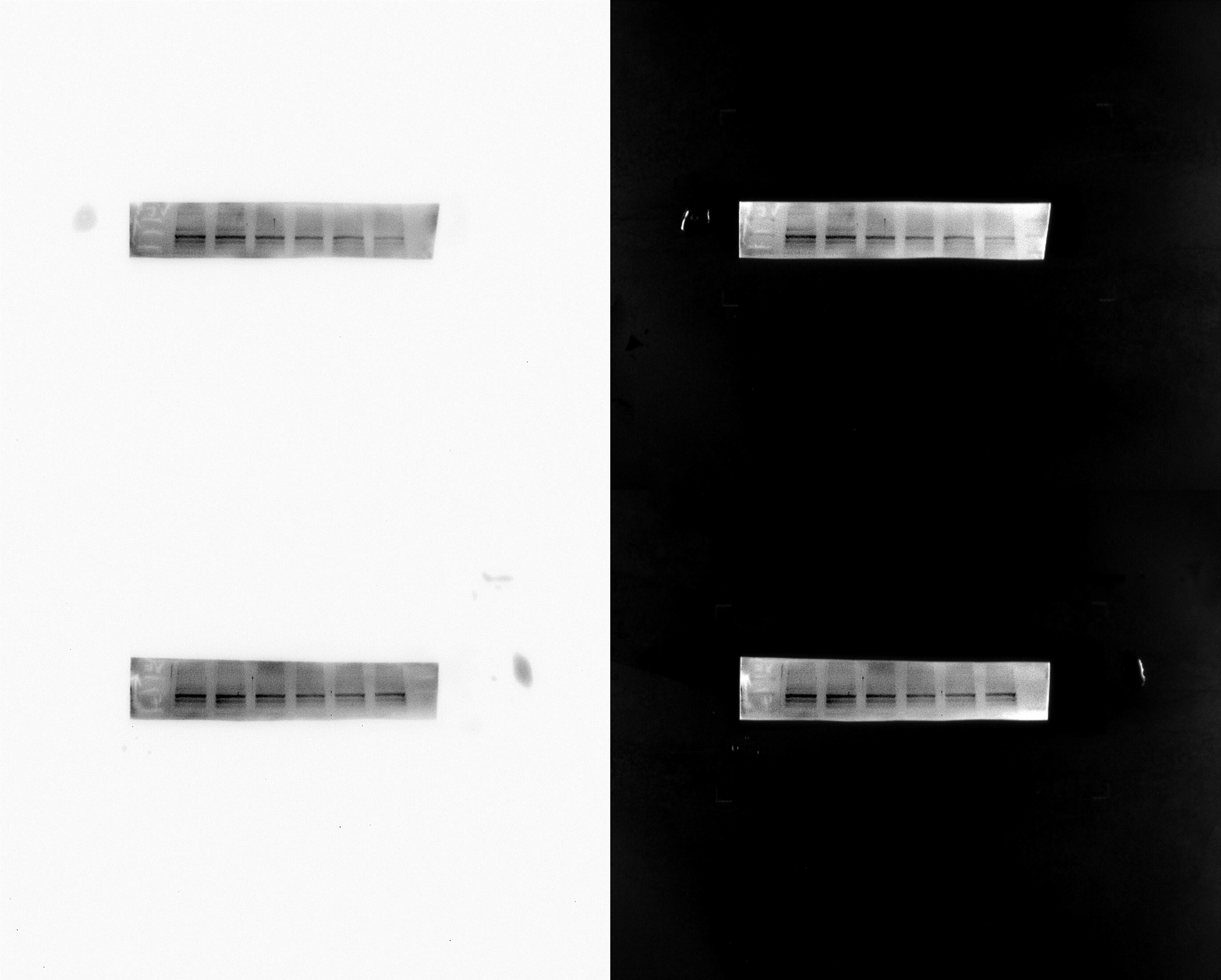

Supplement: Figure 6—source data 1. [file elife-96161-fig6-data1.zip › Figure 6-Source data1/Figure6B-Source data3-VE-Cadherin.png]

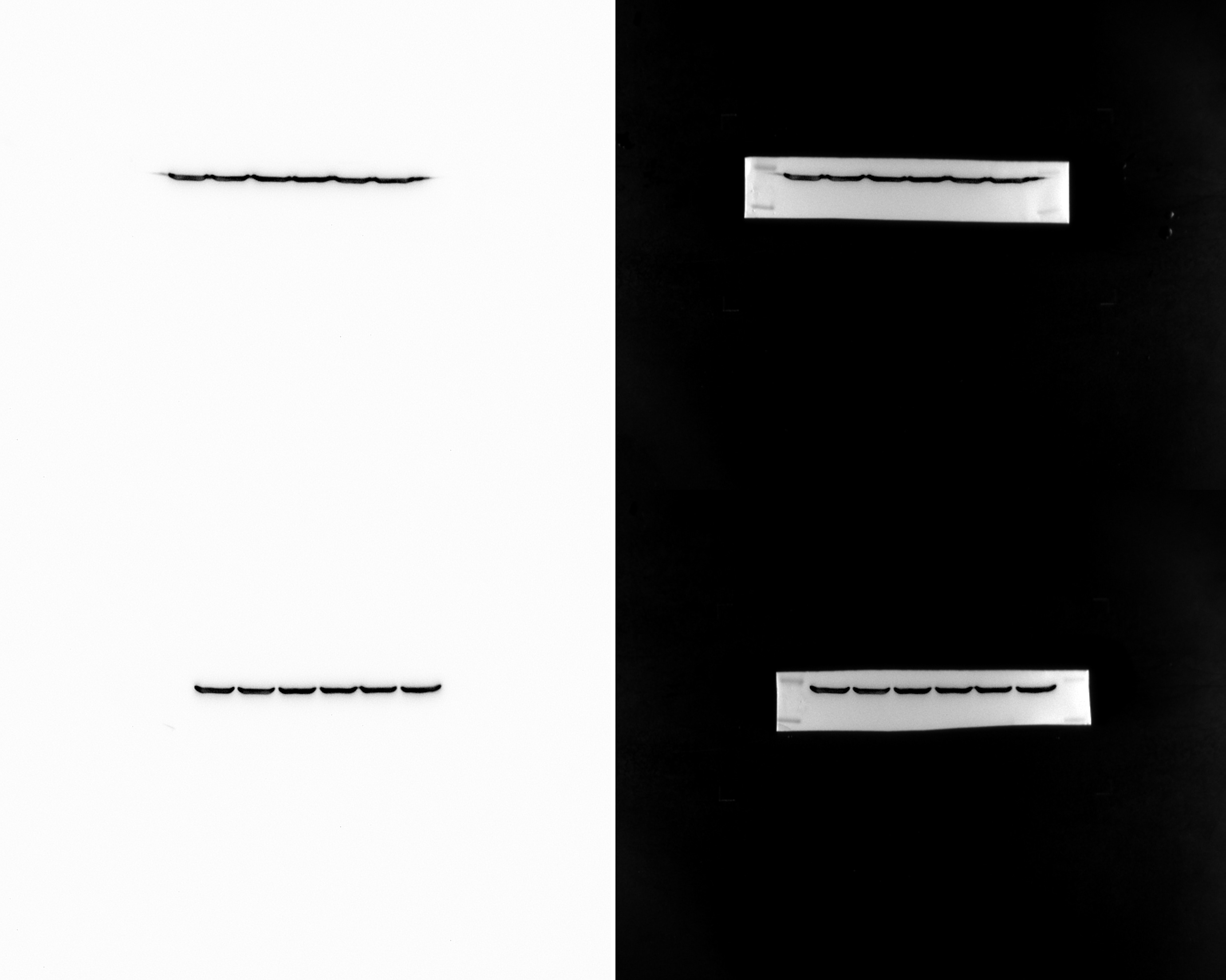

Supplement: Figure 6—source data 1. [file elife-96161-fig6-data1.zip › Figure 6-Source data1/Figure6B-Source data3-a┬-actin(VE-Cadherin).png]

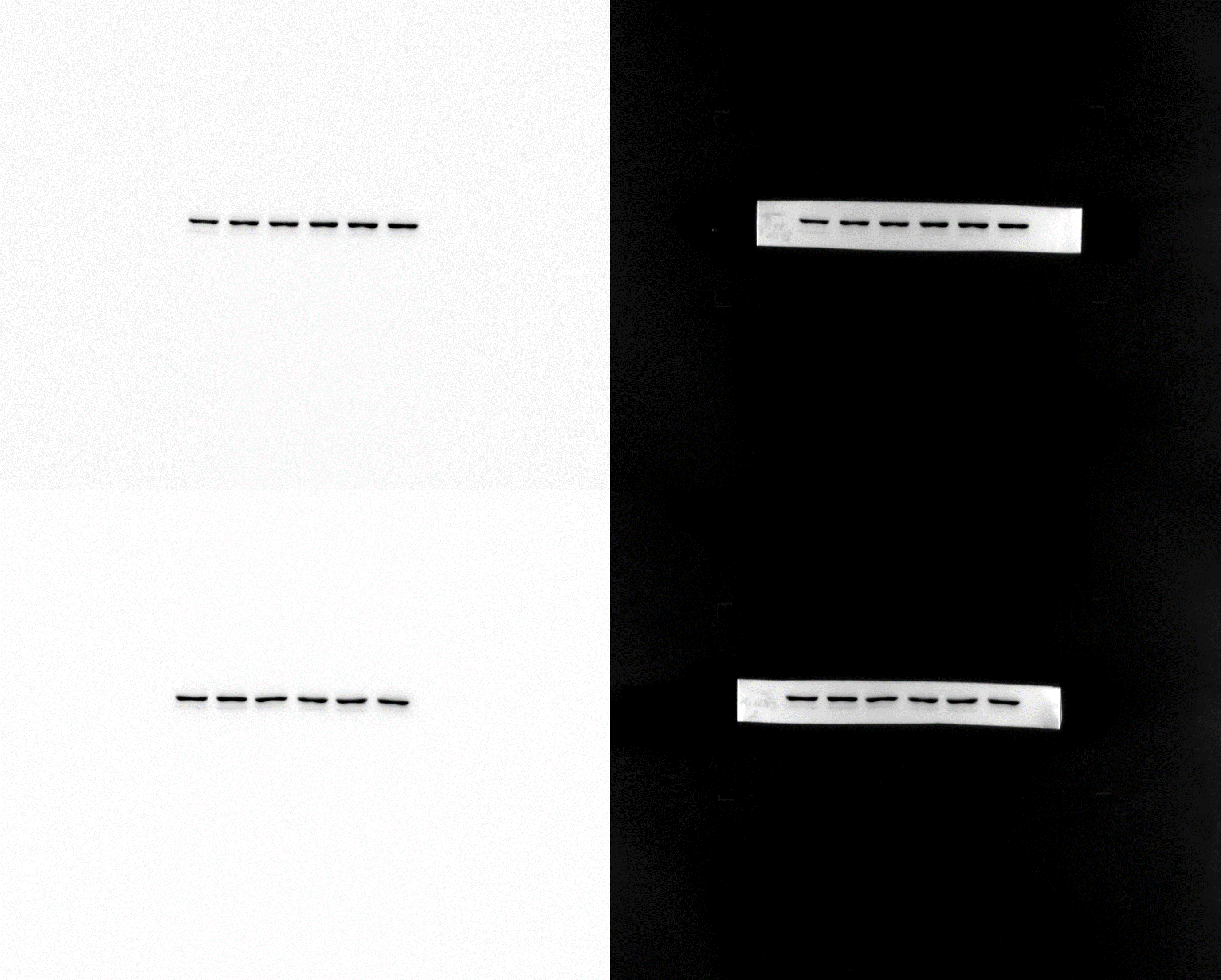

Supplement: Figure 6—source data 1. [file elife-96161-fig6-data1.zip › Figure 6-Source data1/Figure6I,J-Source data-a┬-actin.png]

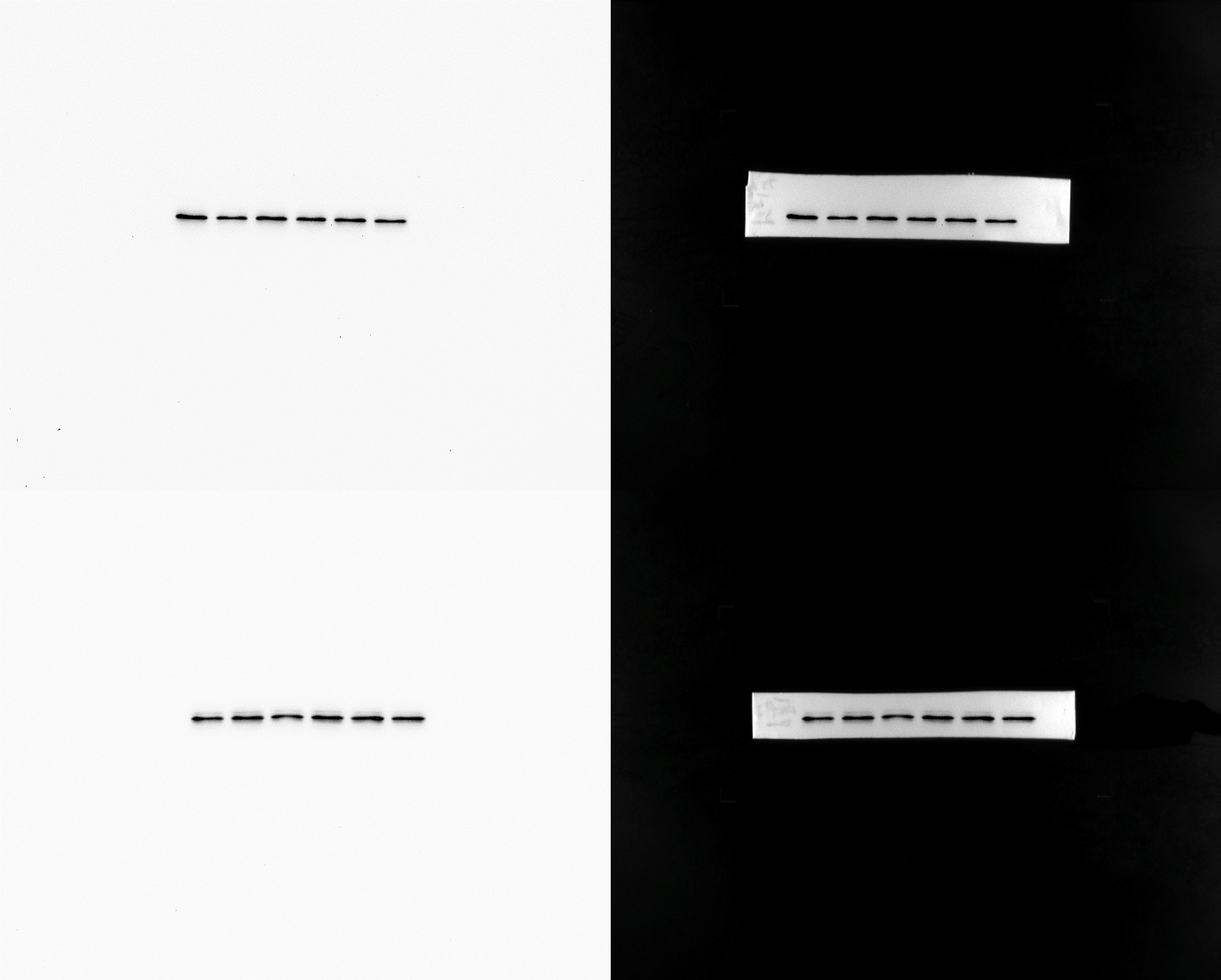

Supplement: Figure 6—source data 1. [file elife-96161-fig6-data1.zip › Figure 6-Source data1/Figure6I-Source data1-AKT.png]

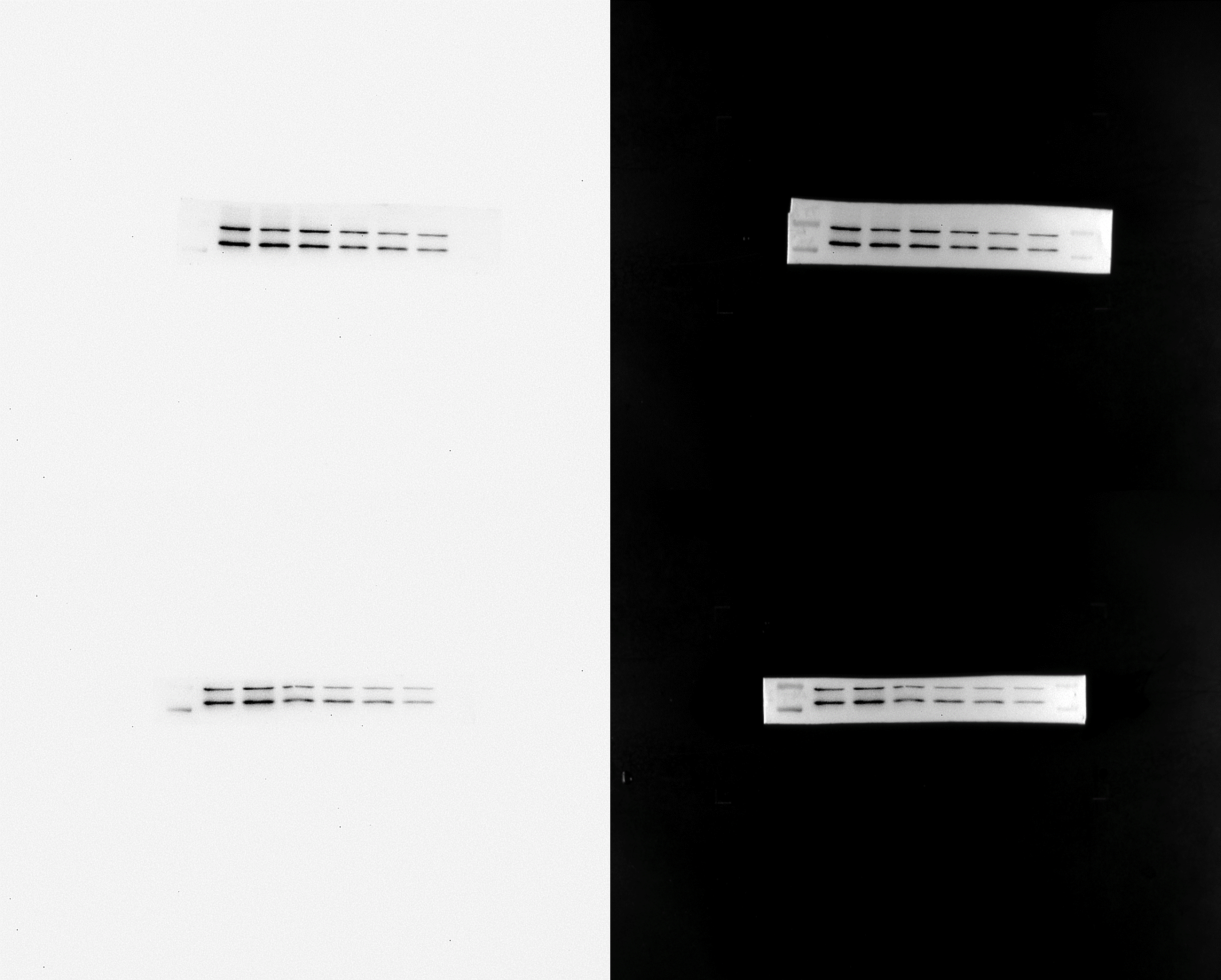

Supplement: Figure 6—source data 1. [file elife-96161-fig6-data1.zip › Figure 6-Source data1/Figure6I-Source data1-p-AKT.png]

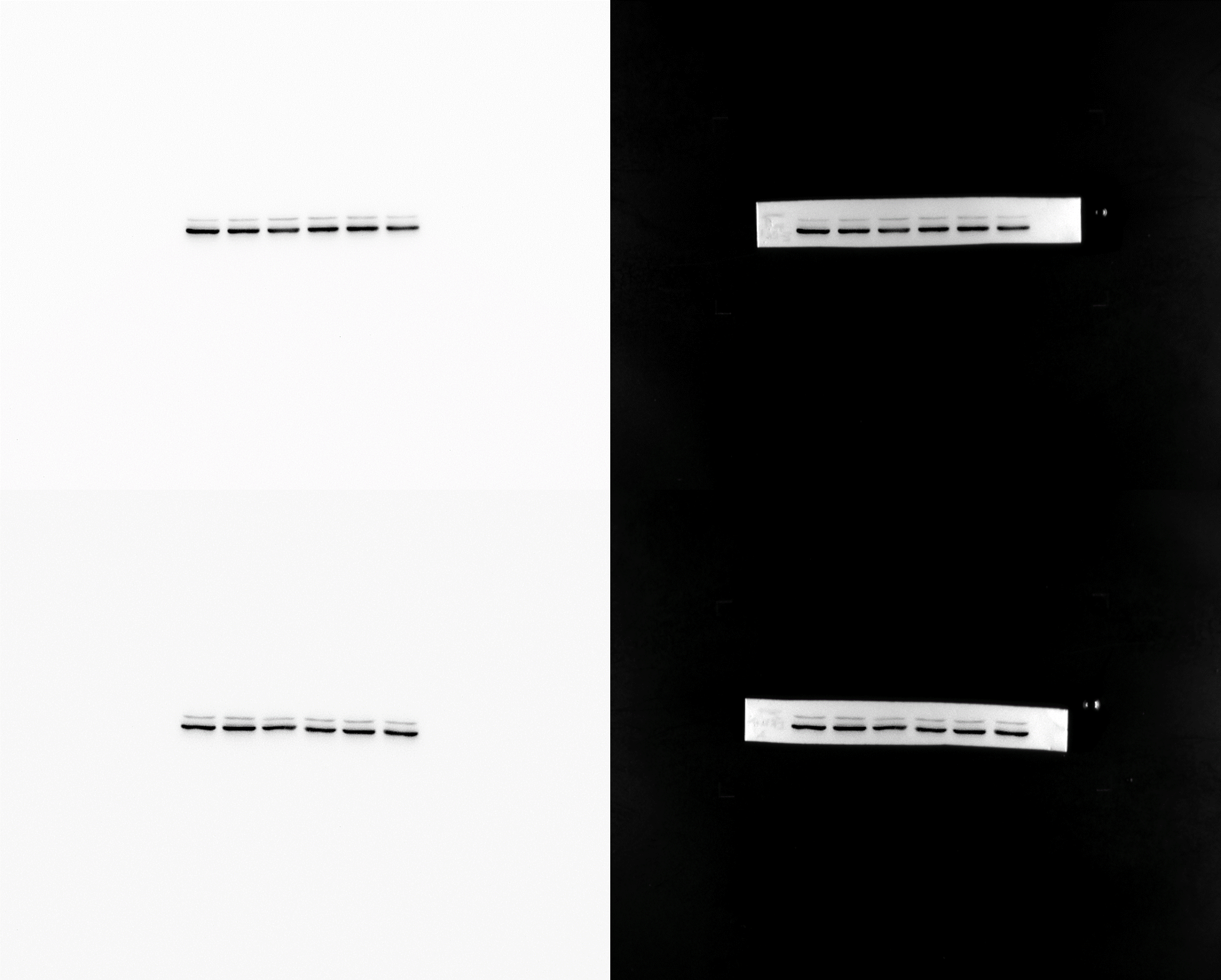

Supplement: Figure 6—source data 1. [file elife-96161-fig6-data1.zip › Figure 6-Source data1/Figure6J-Source data1-ERK.png]

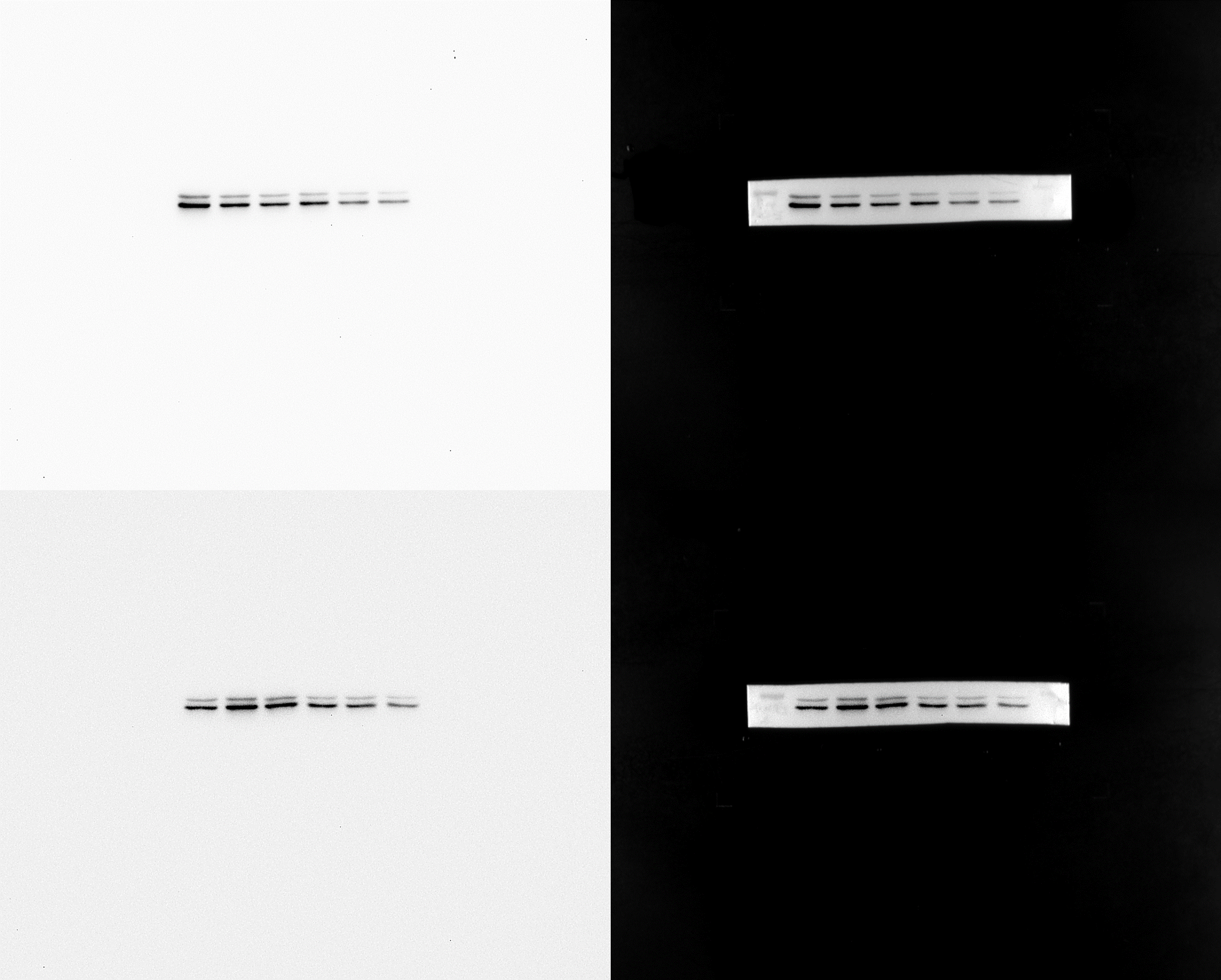

Supplement: Figure 6—source data 1. [file elife-96161-fig6-data1.zip › Figure 6-Source data1/Figure6J-Source data1-p-ERK.png]

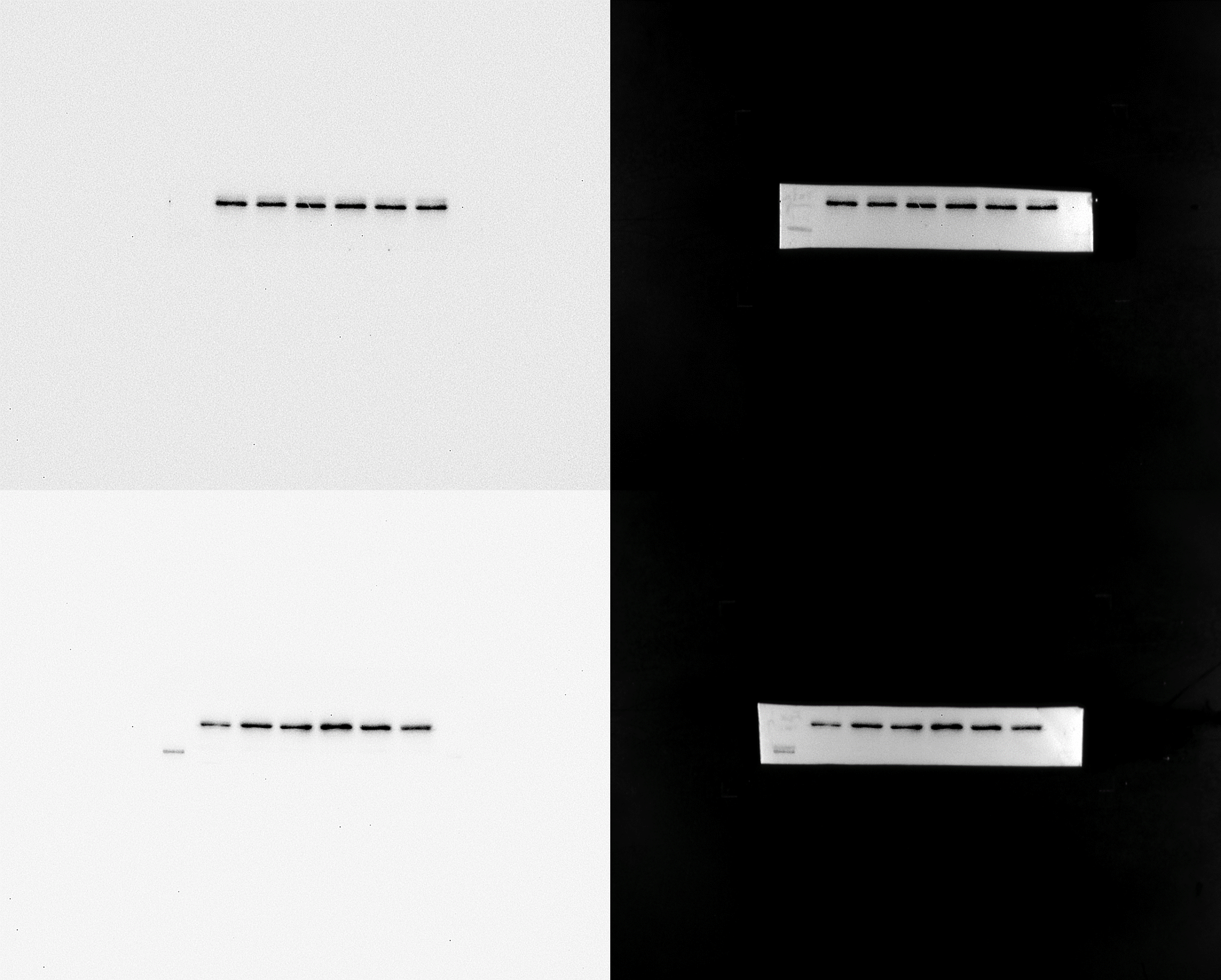

Supplement: Figure 6—source data 1. [file elife-96161-fig6-data1.zip › Figure 6-Source data1/Figure6K-Source data1-FOXO1.png]

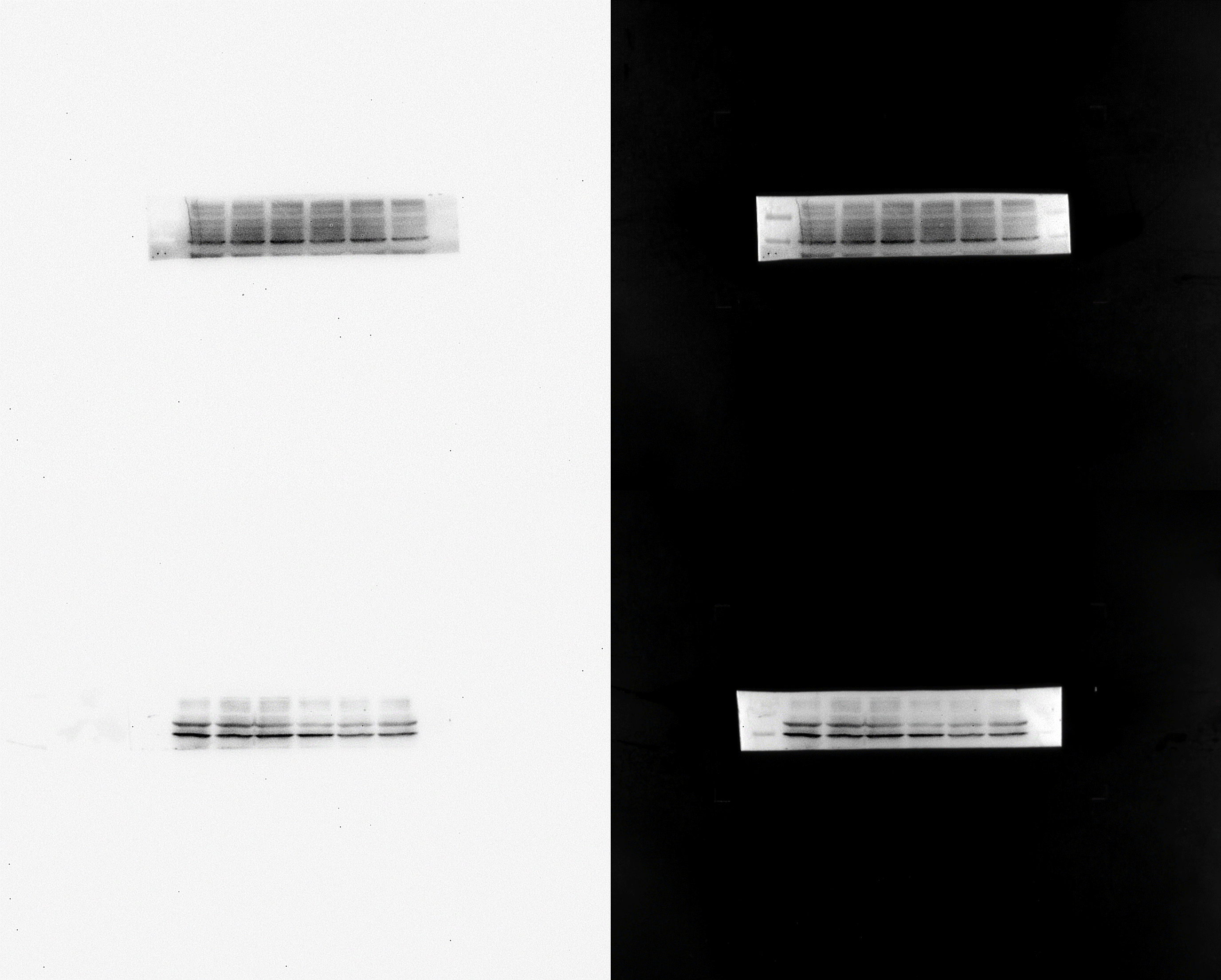

Supplement: Figure 6—source data 1. [file elife-96161-fig6-data1.zip › Figure 6-Source data1/Figure6K-Source data1-p-FOXO1.png]

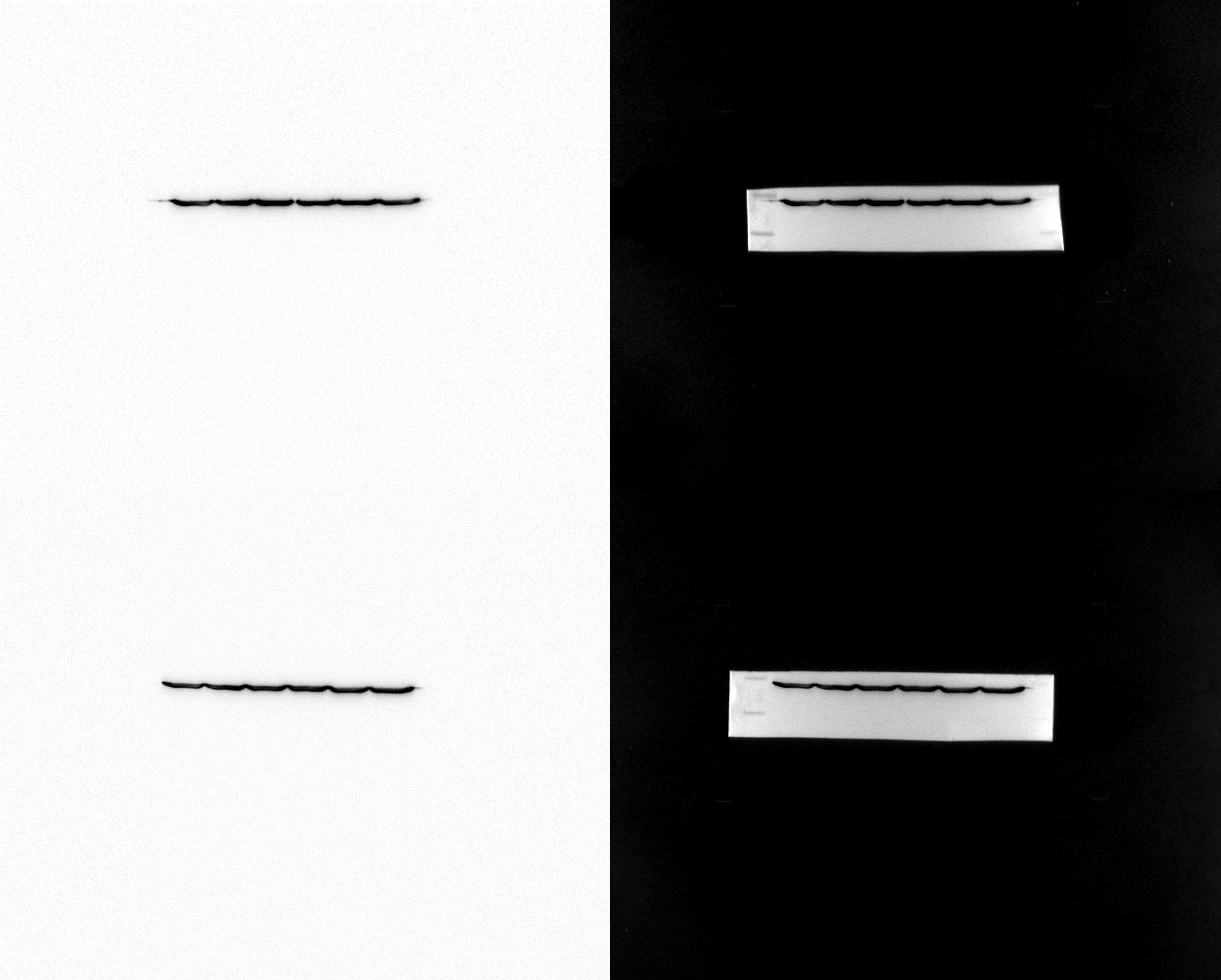

Supplement: Figure 6—source data 1. [file elife-96161-fig6-data1.zip › Figure 6-Source data1/Figure6K-Source data1-a┬-actin.png]

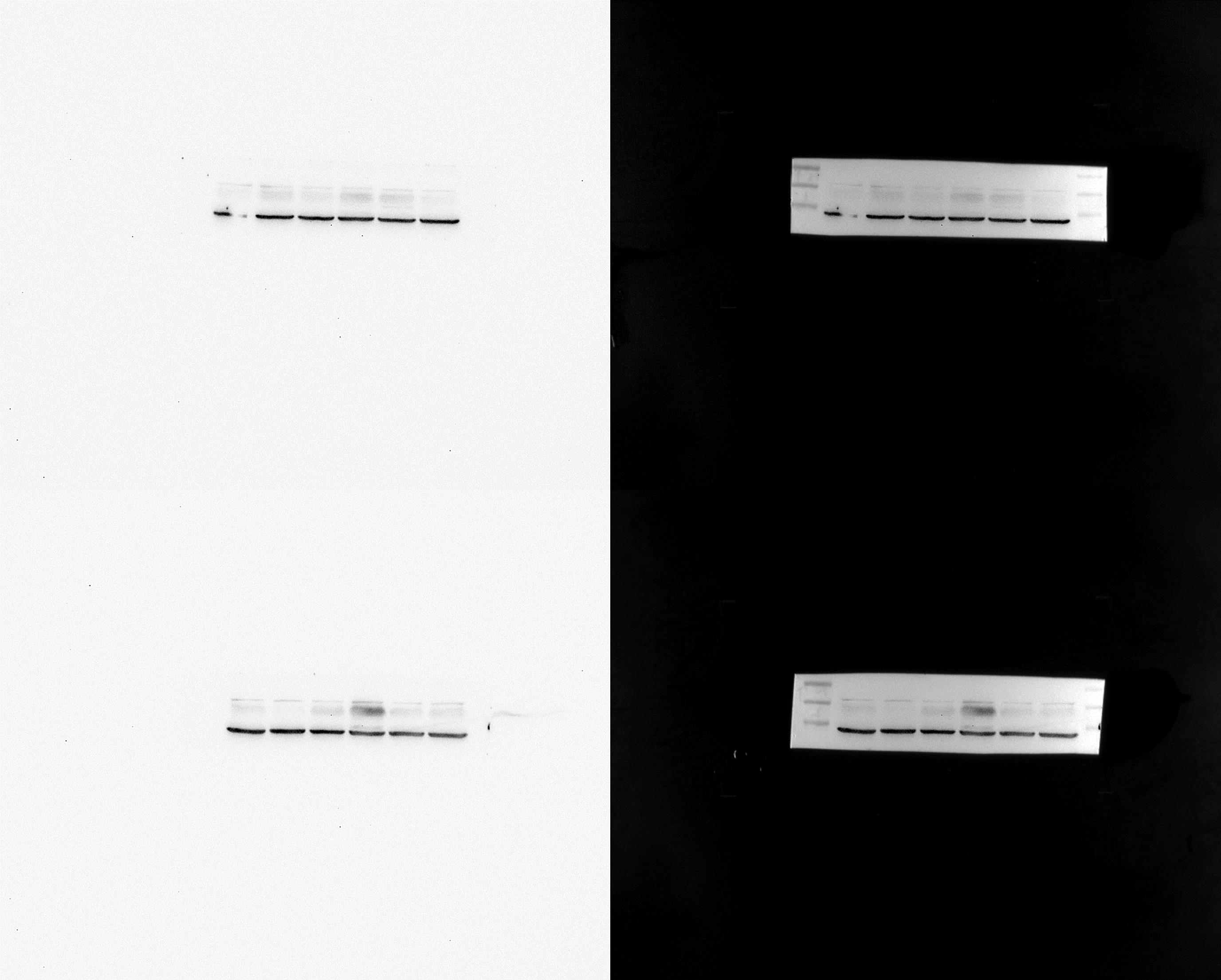

Supplement: Figure 6—source data 1. [file elife-96161-fig6-data1.zip › Figure 6-Source data1/Figure6L-Source data1-ETS-1.png]

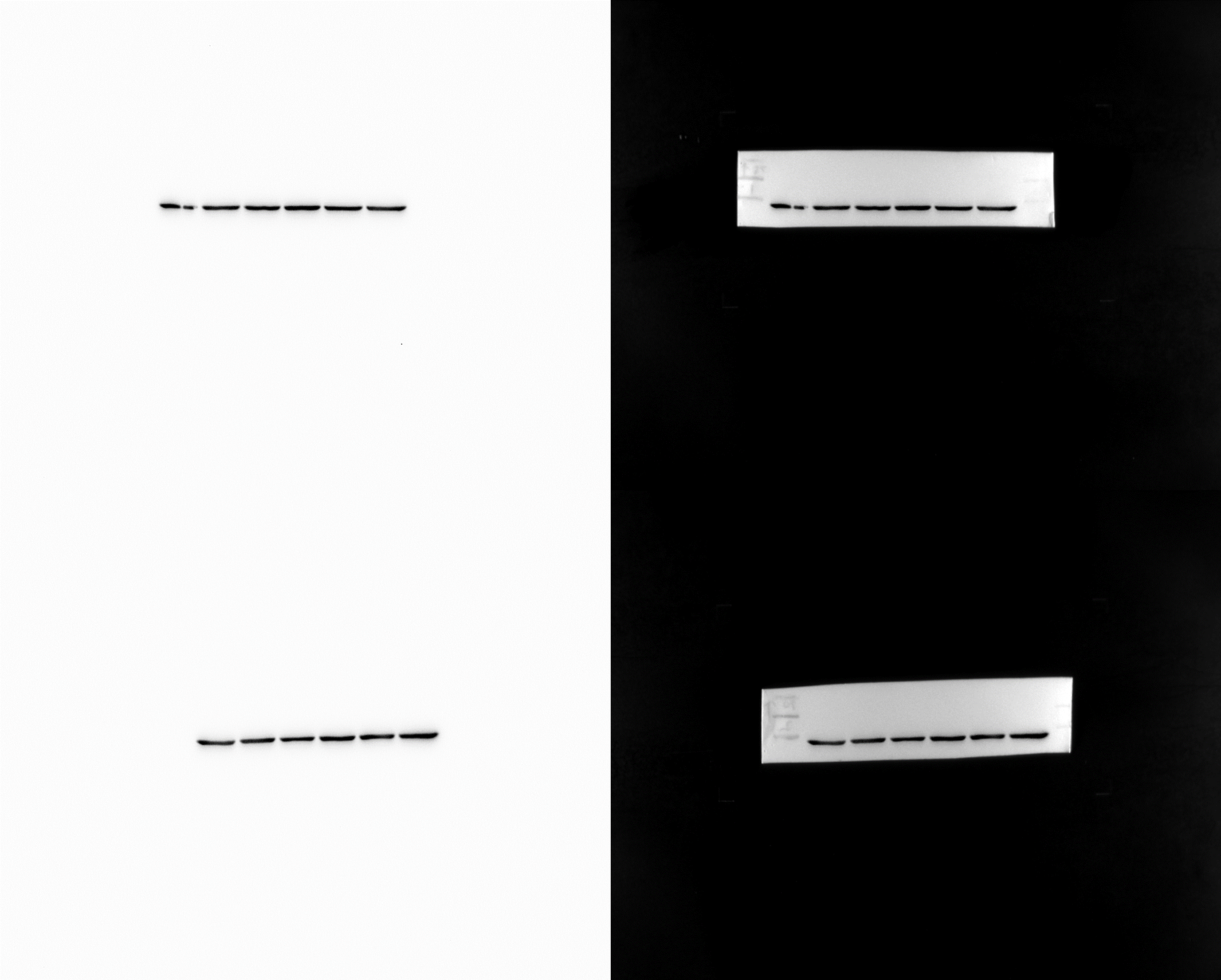

Supplement: Figure 6—source data 1. [file elife-96161-fig6-data1.zip › Figure 6-Source data1/Figure6L-Source data1-a┬-actin.png]
