# Supplementary material for: Neurons enhance blood–brain barrier function via upregulating claudin-5 and VE-cadherin expression due to glial cell line-derived neurotrophic factor secretion
Source: eLife. 2024 Oct 30;13:RP96161. doi: 10.7554/eLife.96161 (PMC11524583; doi:10.7554/eLife.96161)

Figure 3A-Claudin-5

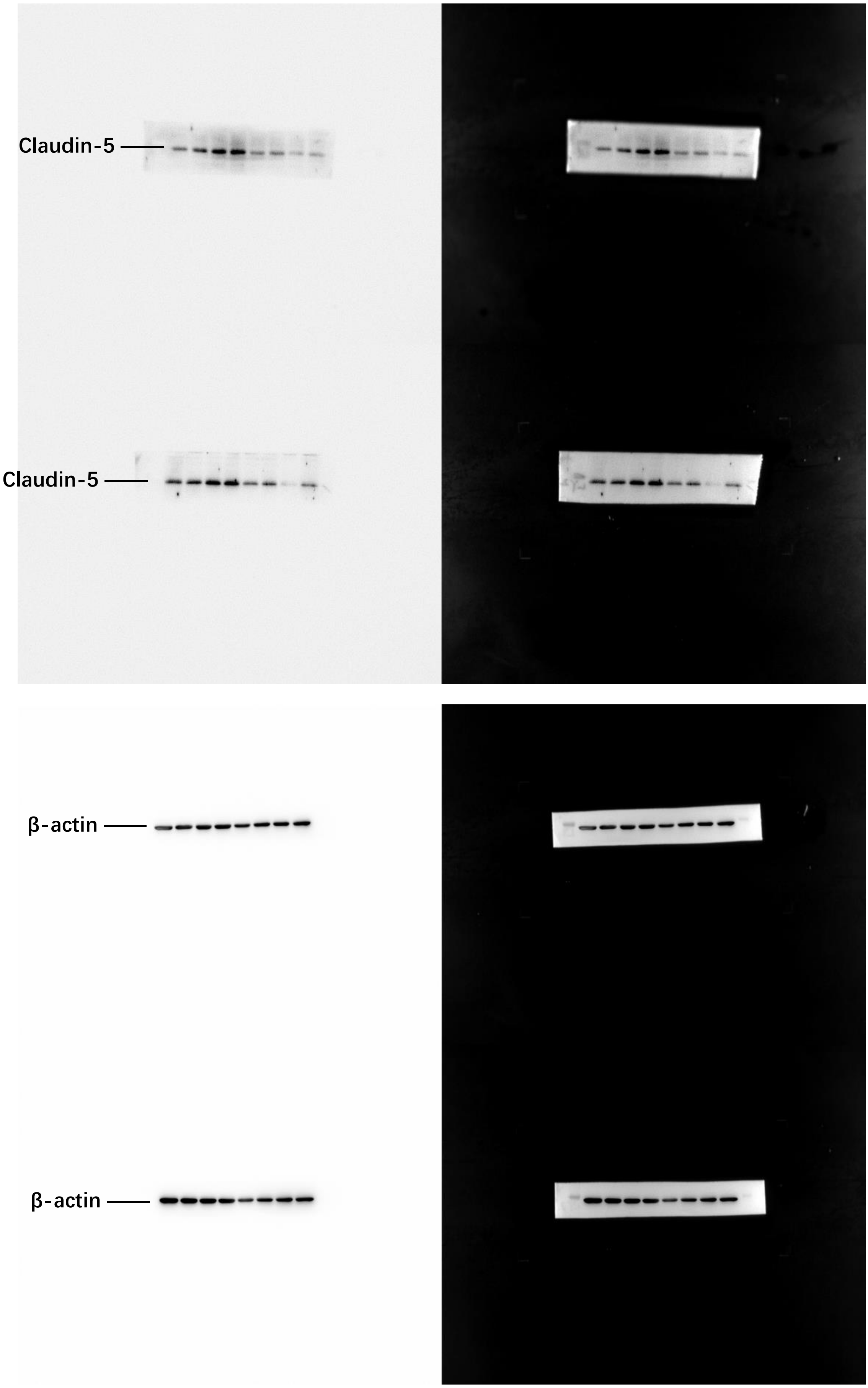

Figure 3A-VE-cadherin

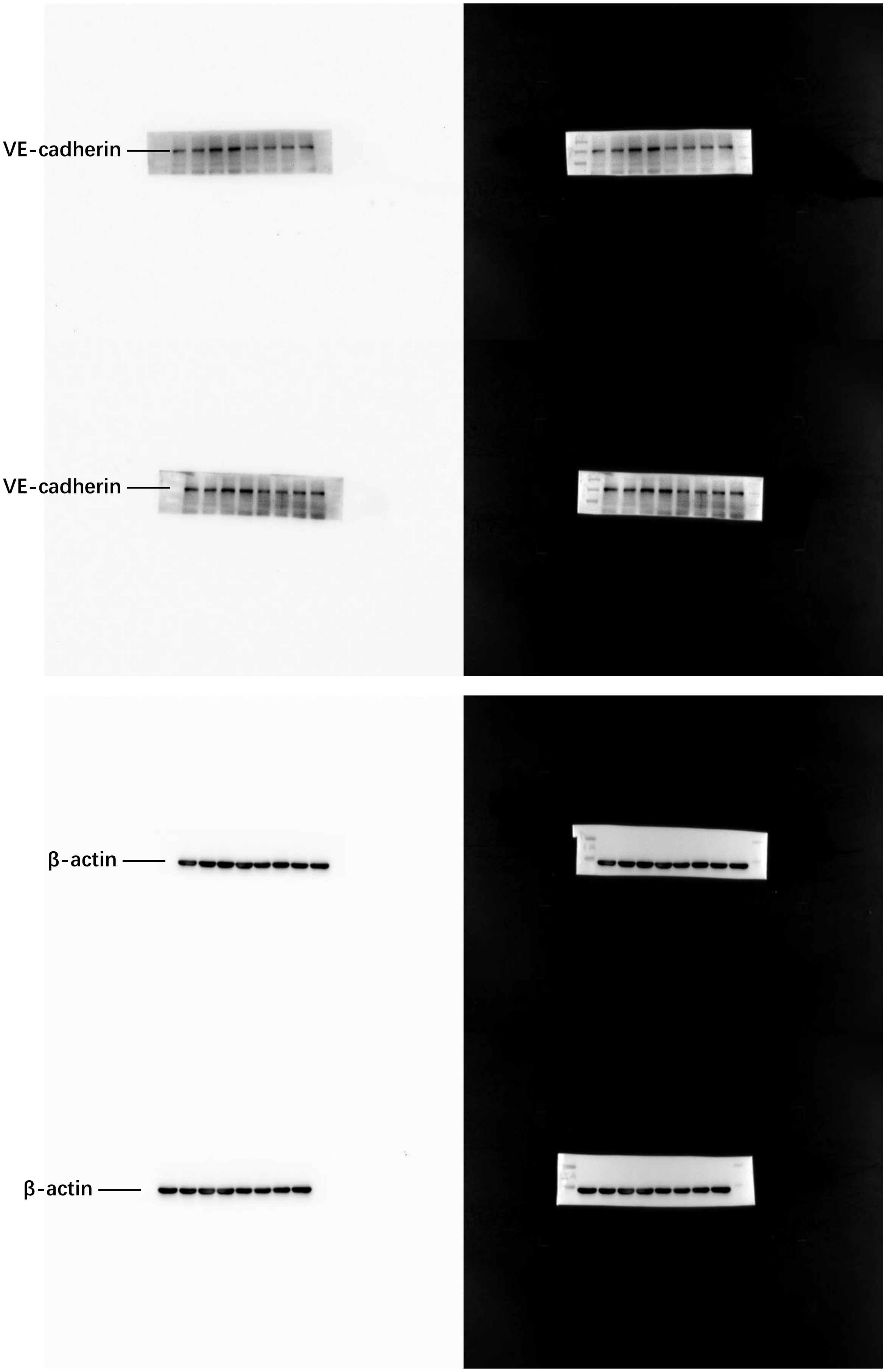

Figure 3A-pAKT/AKT

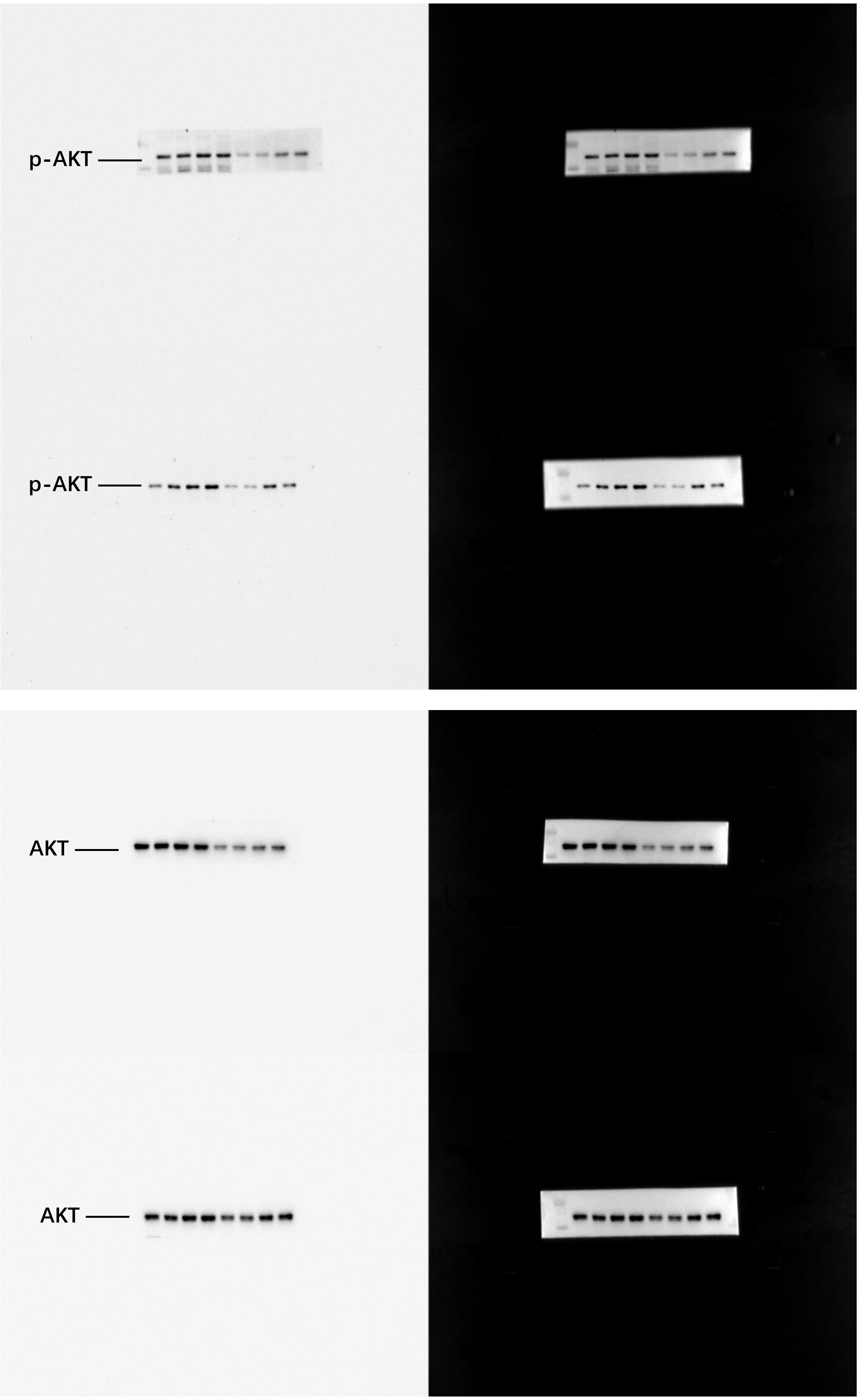

Figure 3B-claudin-5

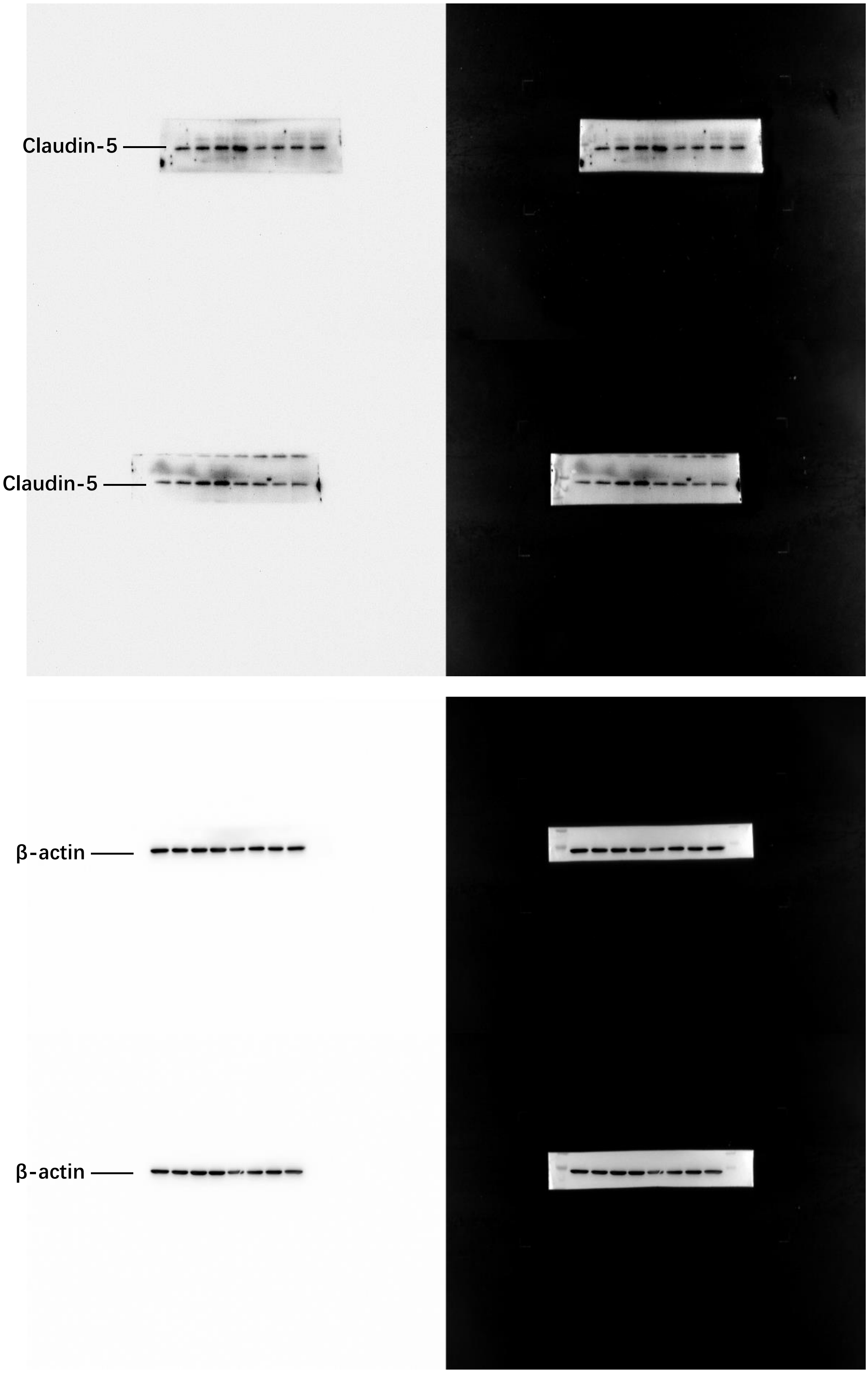

Figure 3B-VE-cadherin

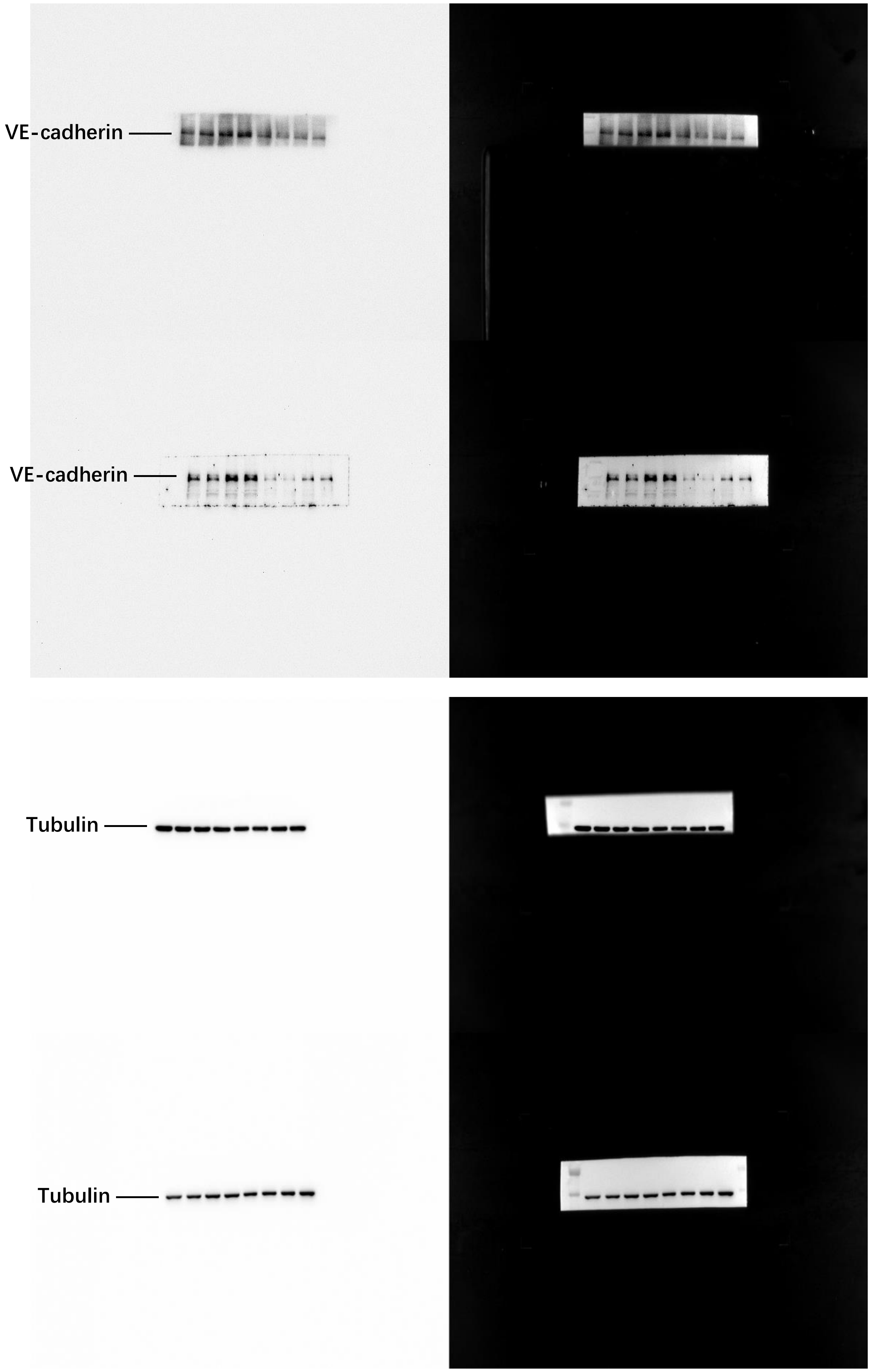

Figure 3B-p-ERK/ERK

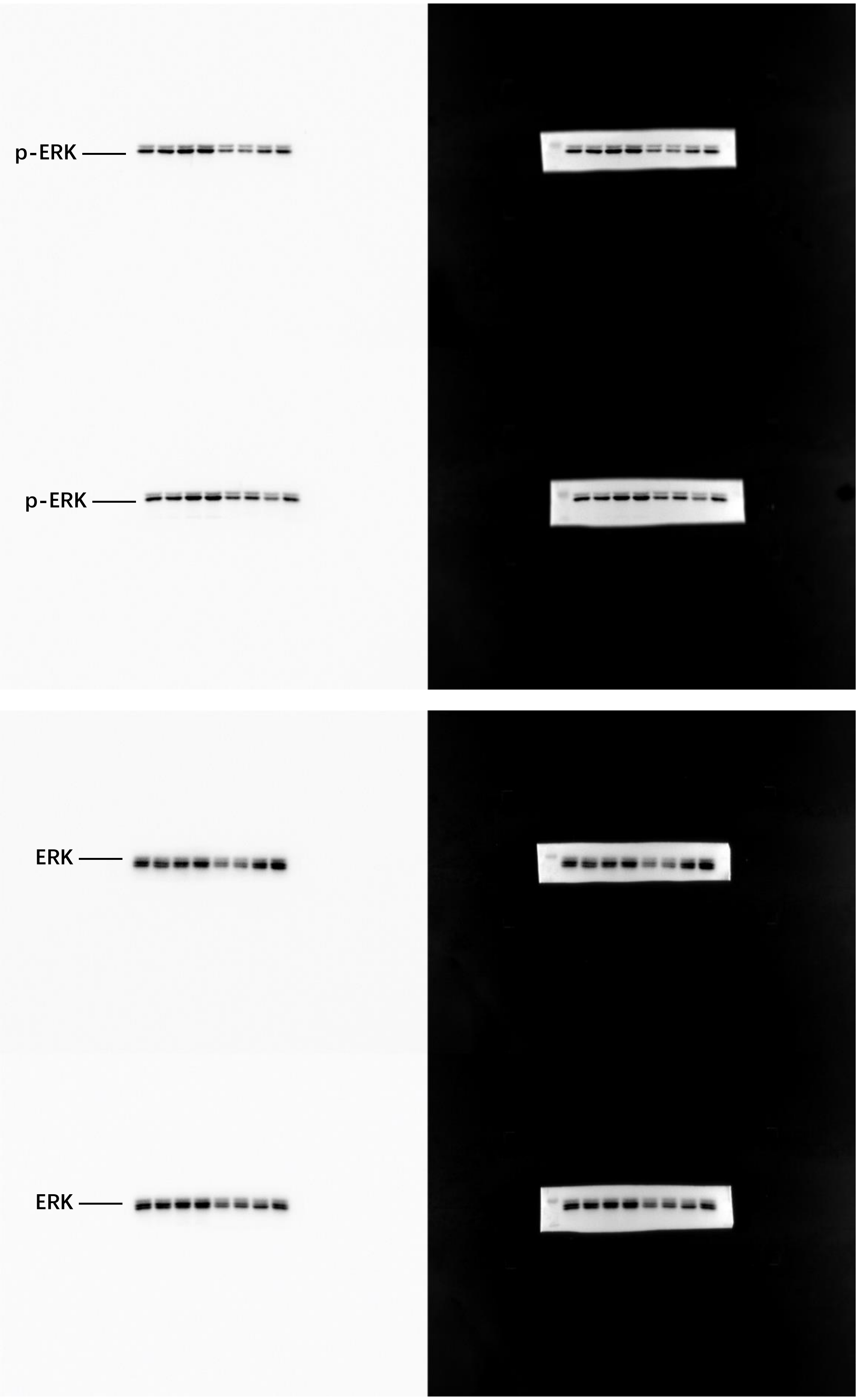

Figure 3C-claudin-5

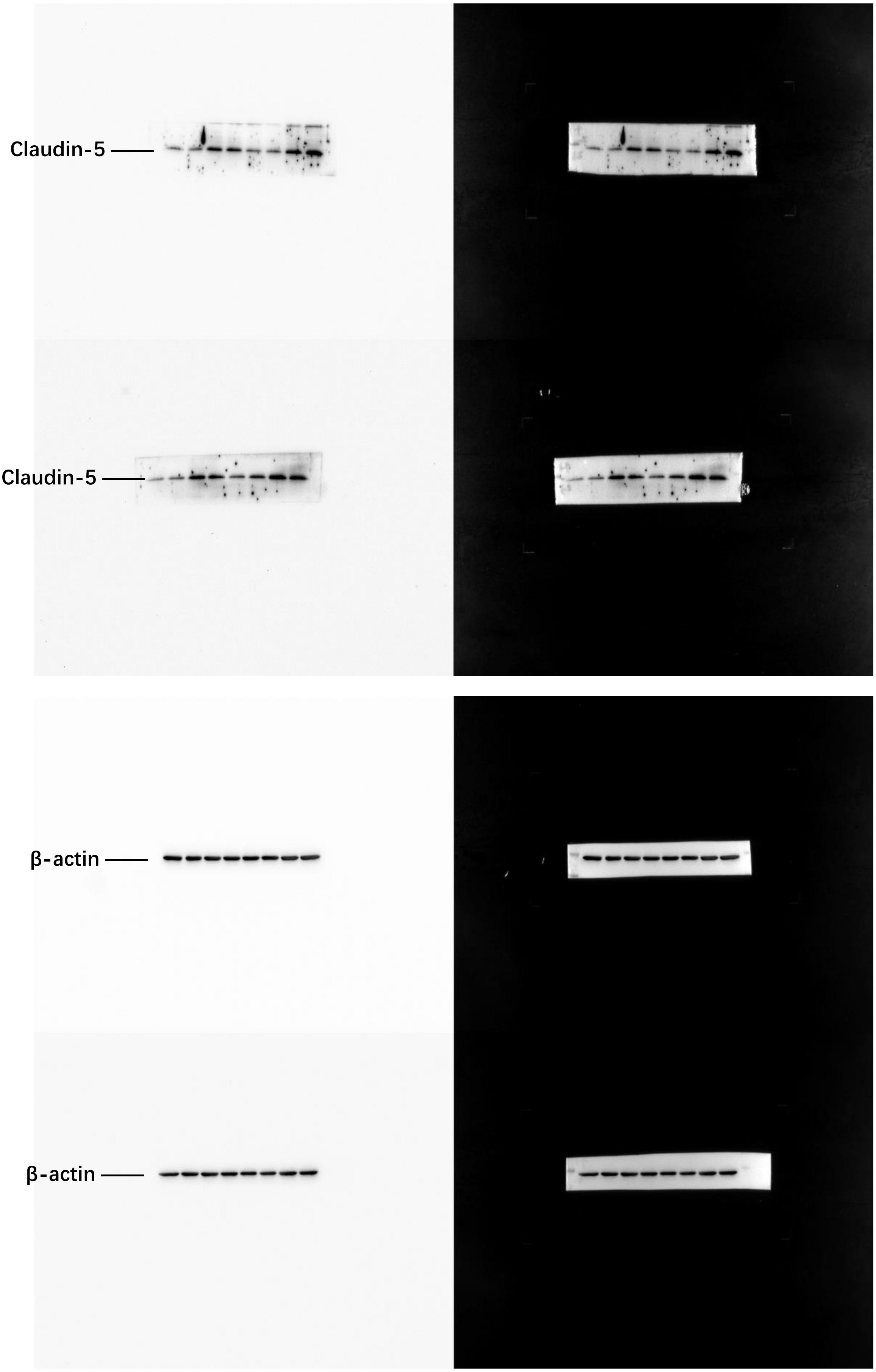

Figure 3C-VE-cadherin

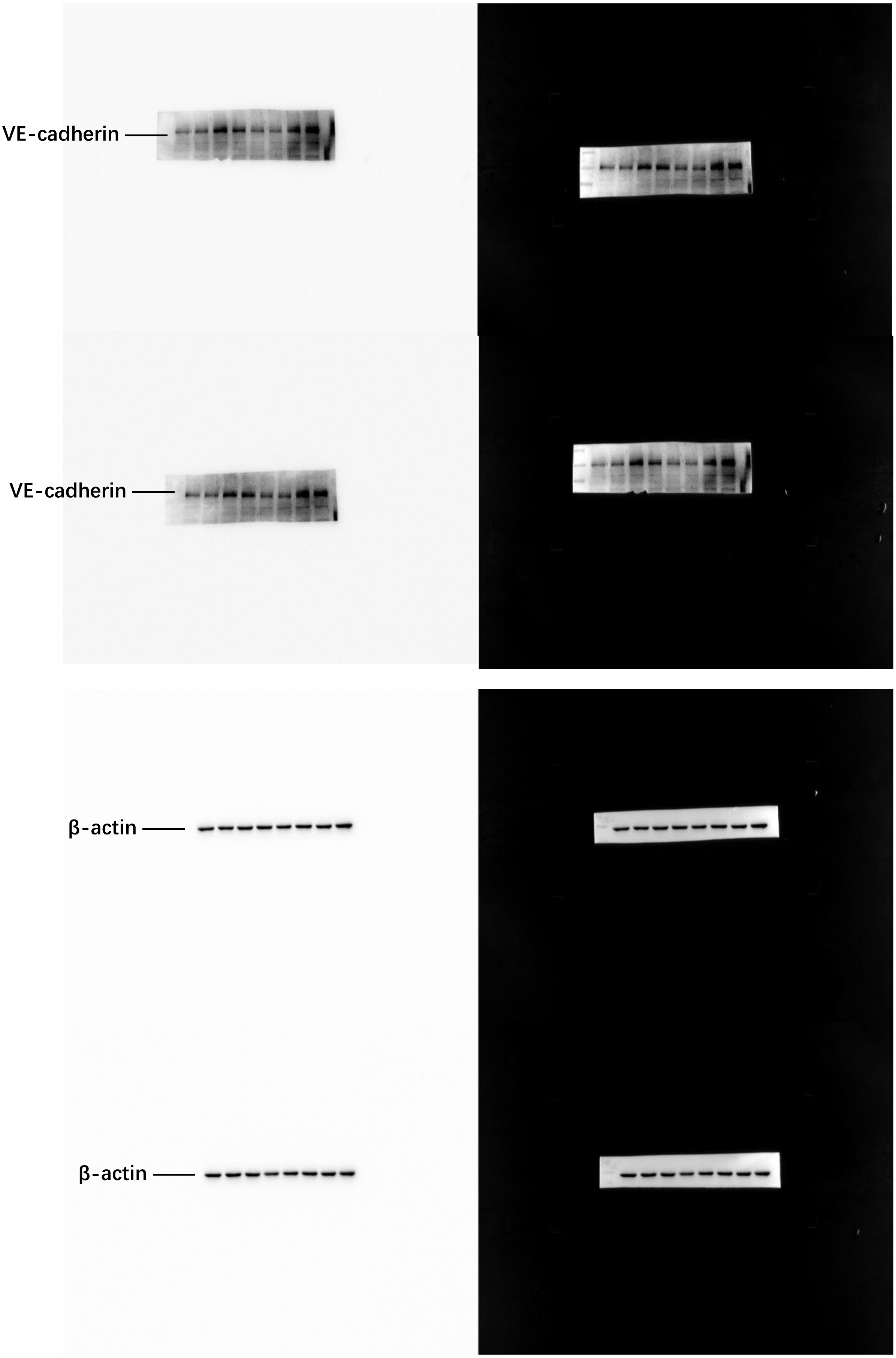

Figure 3C-p-JNK/JNK

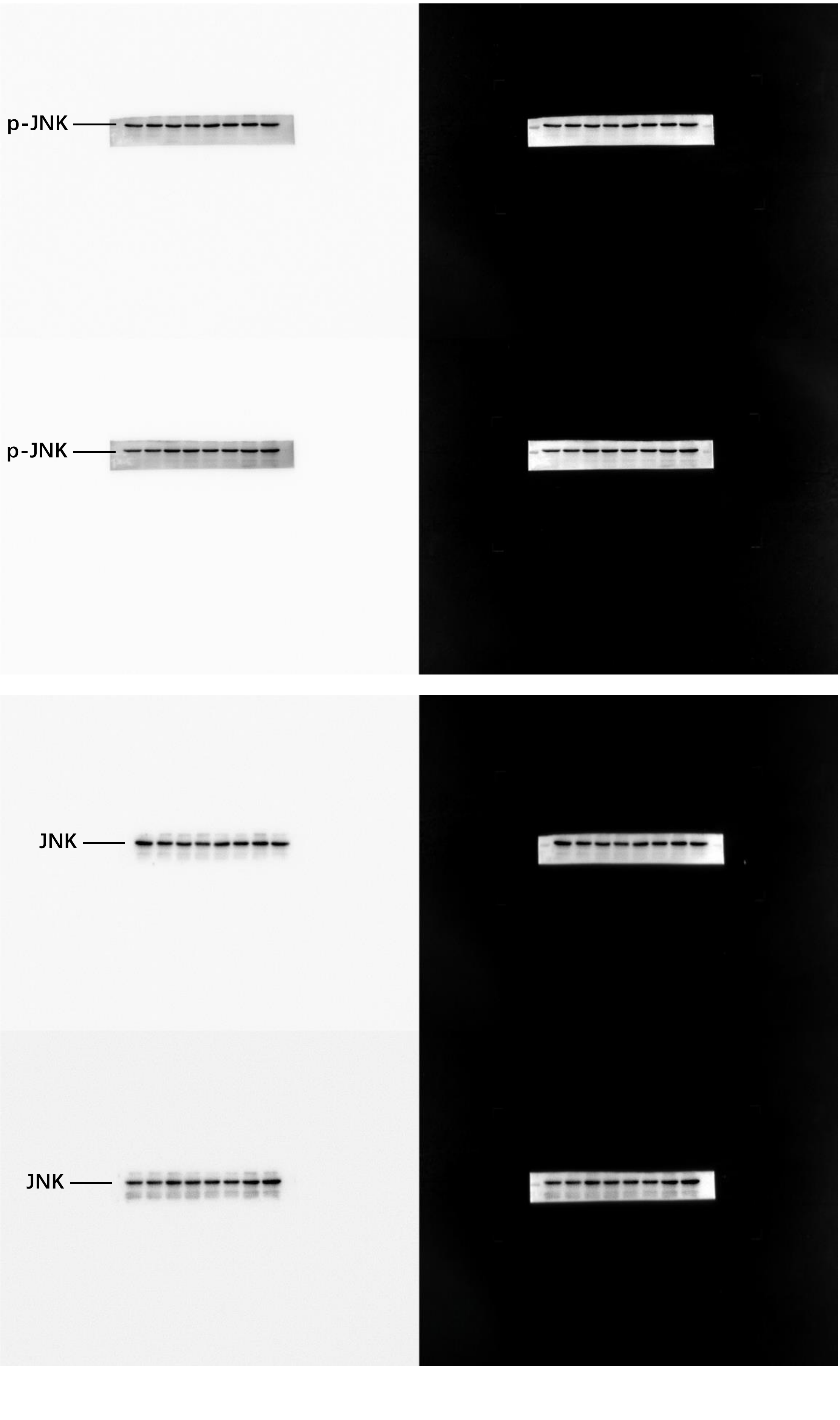

Figure 3D-claudin-5

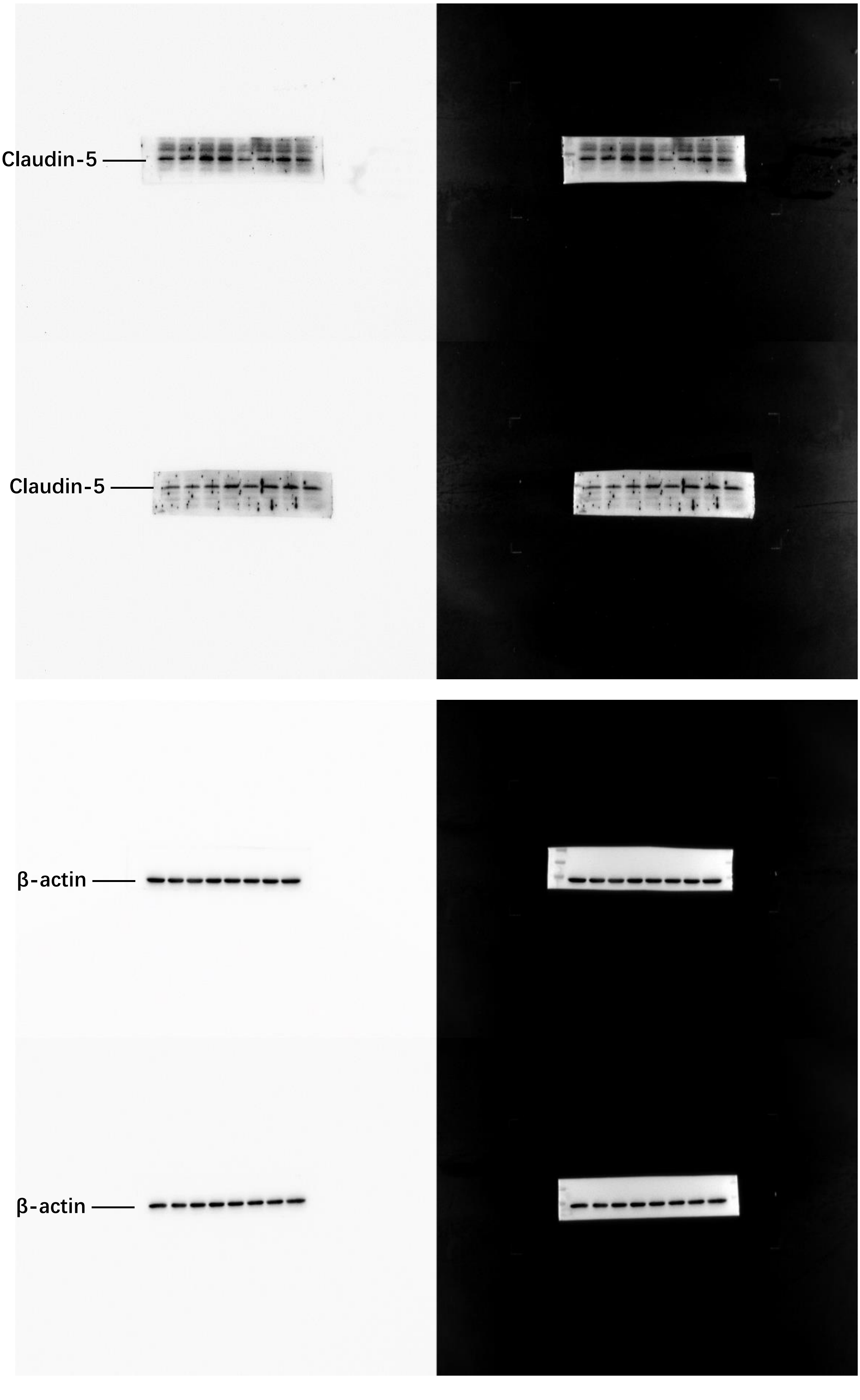

Figure 3D-VE-cadherin

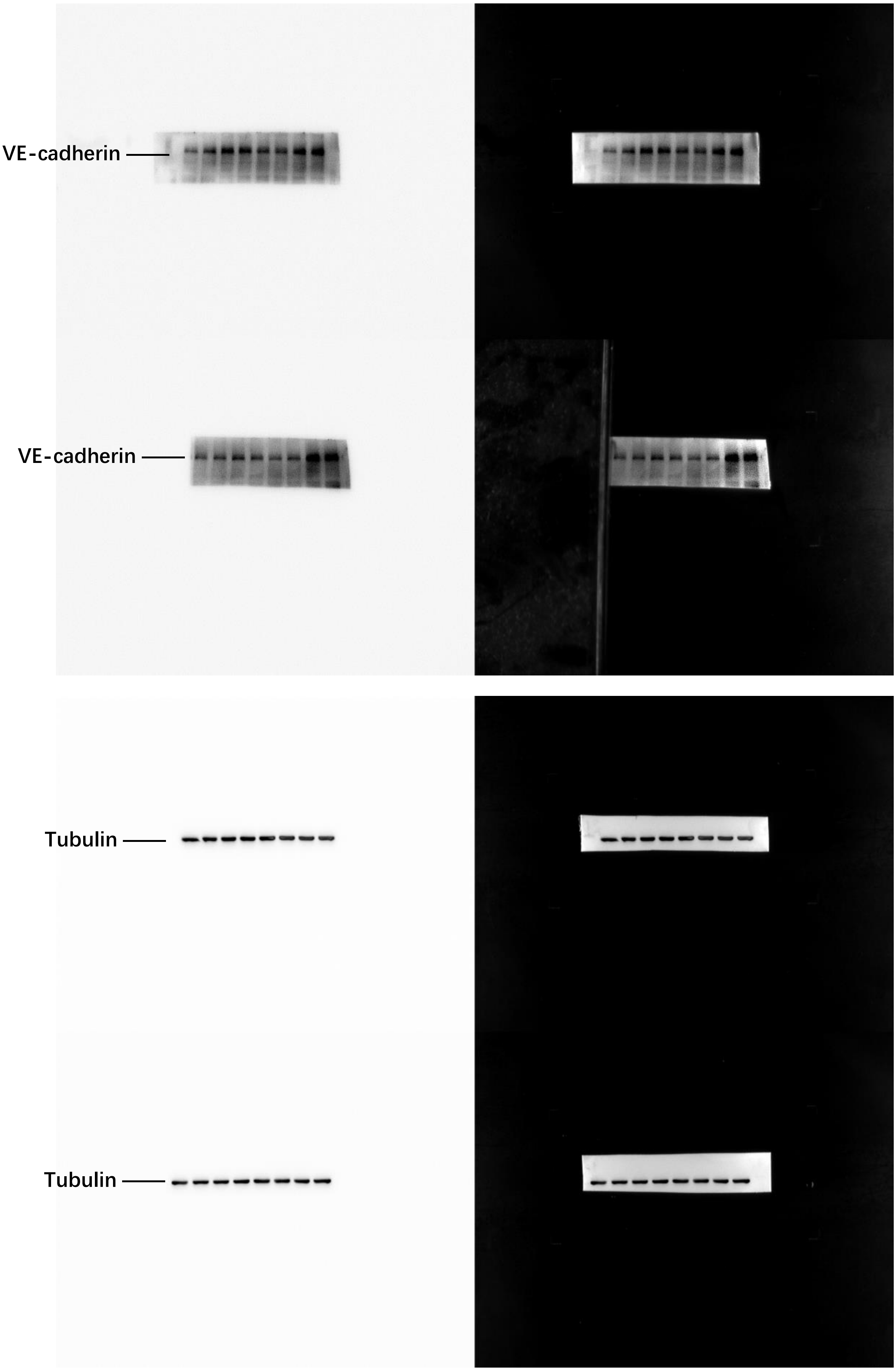

Figure 3D-p-p38/p38

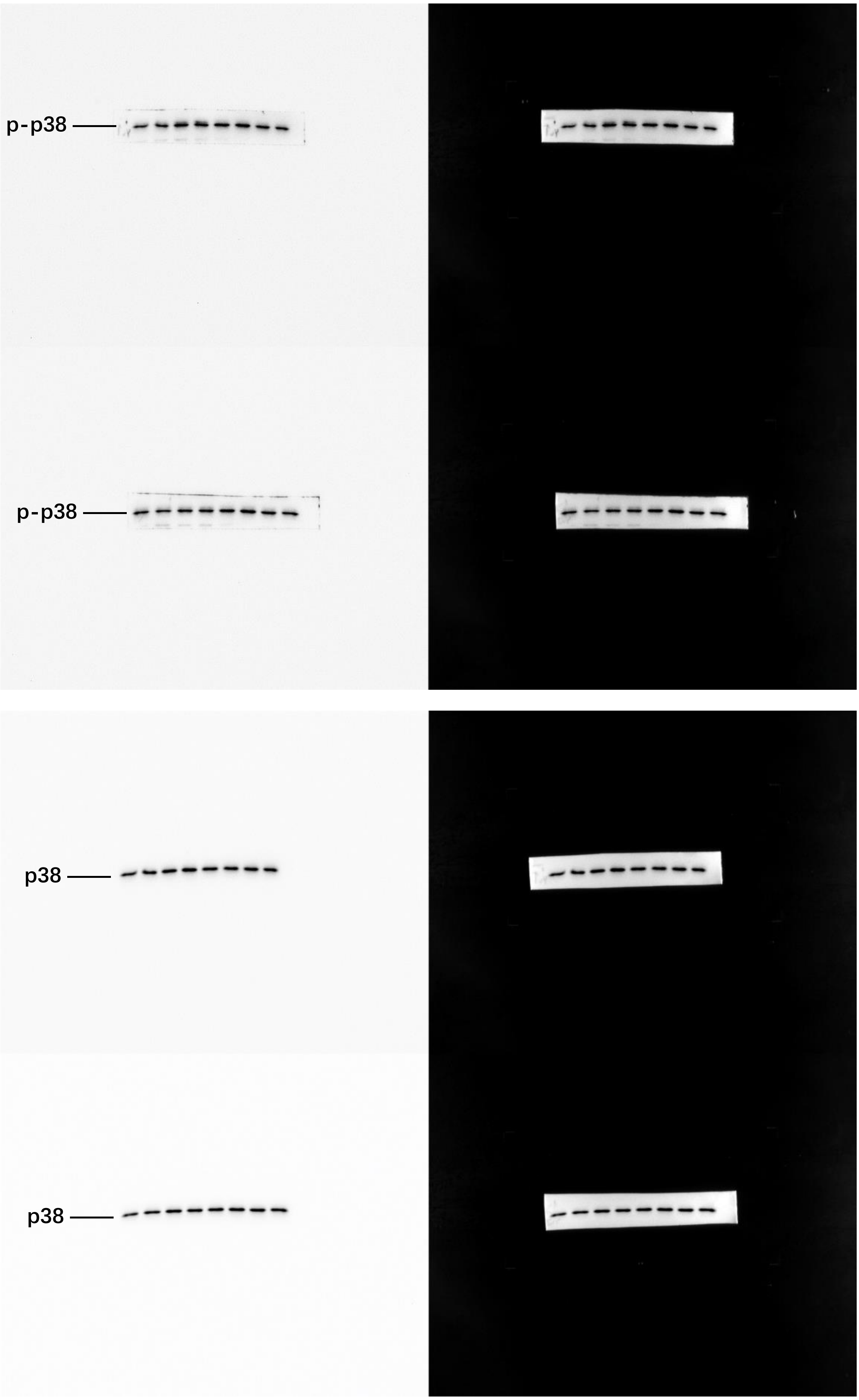

Figure 3E-p-AKT/AKT

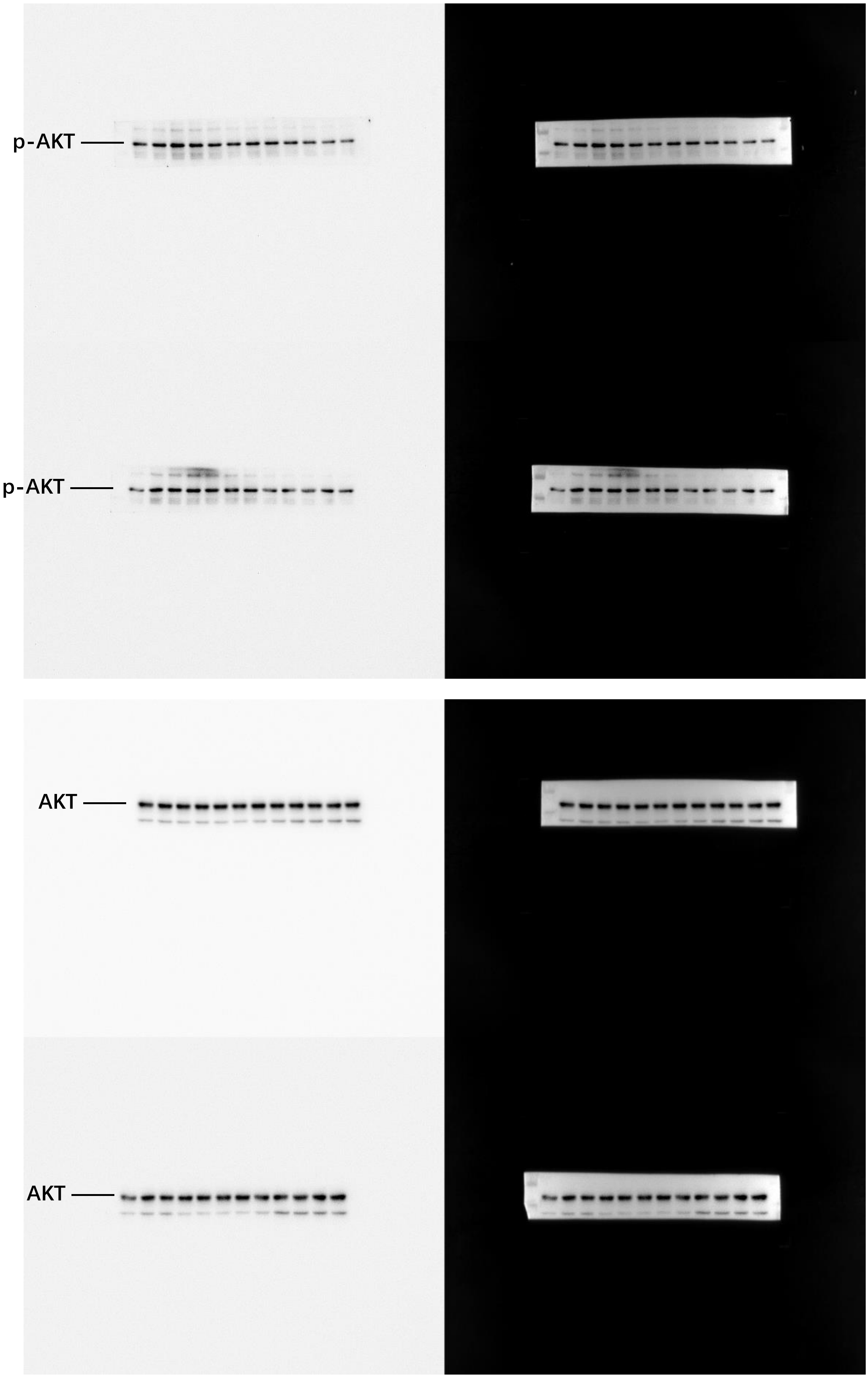

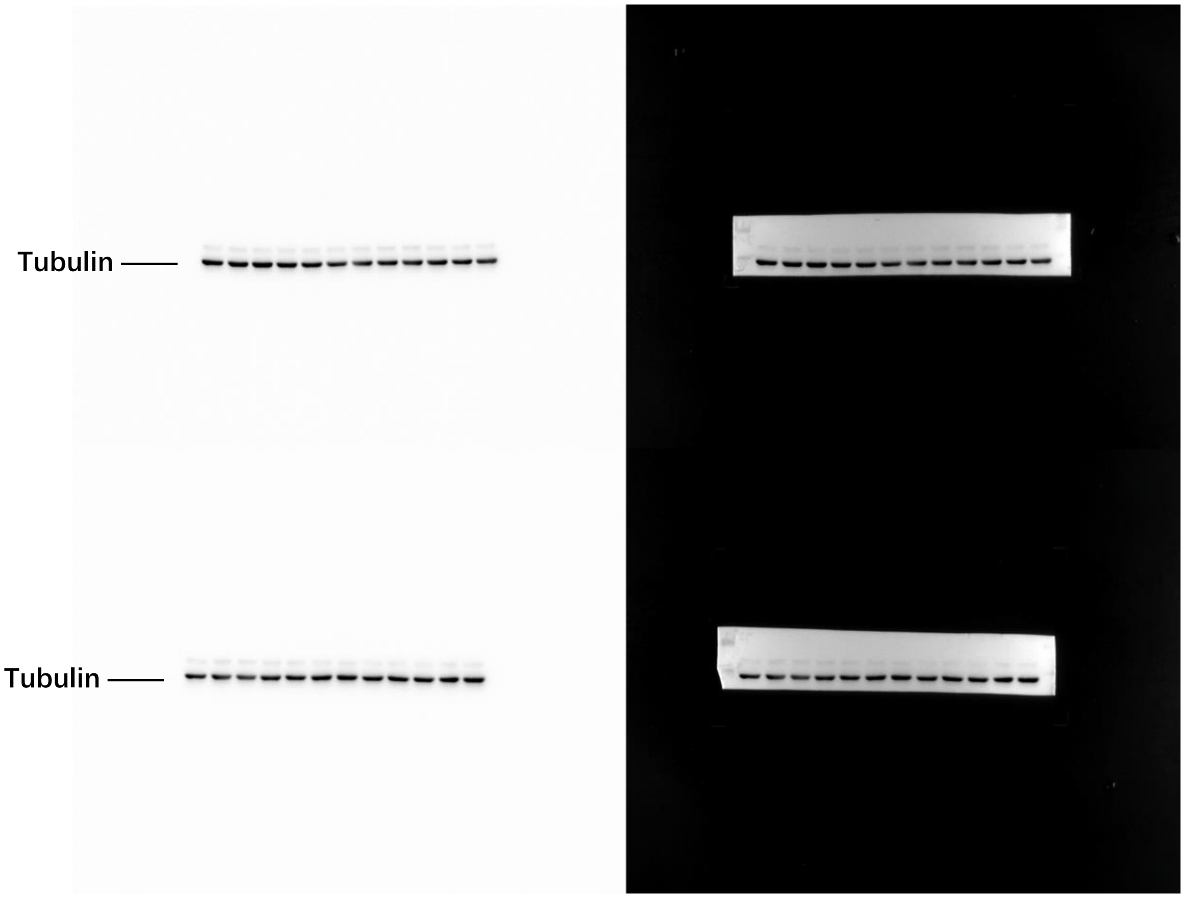
Tubulin
Tubulin

Figure 3E-p-ERK/ERK

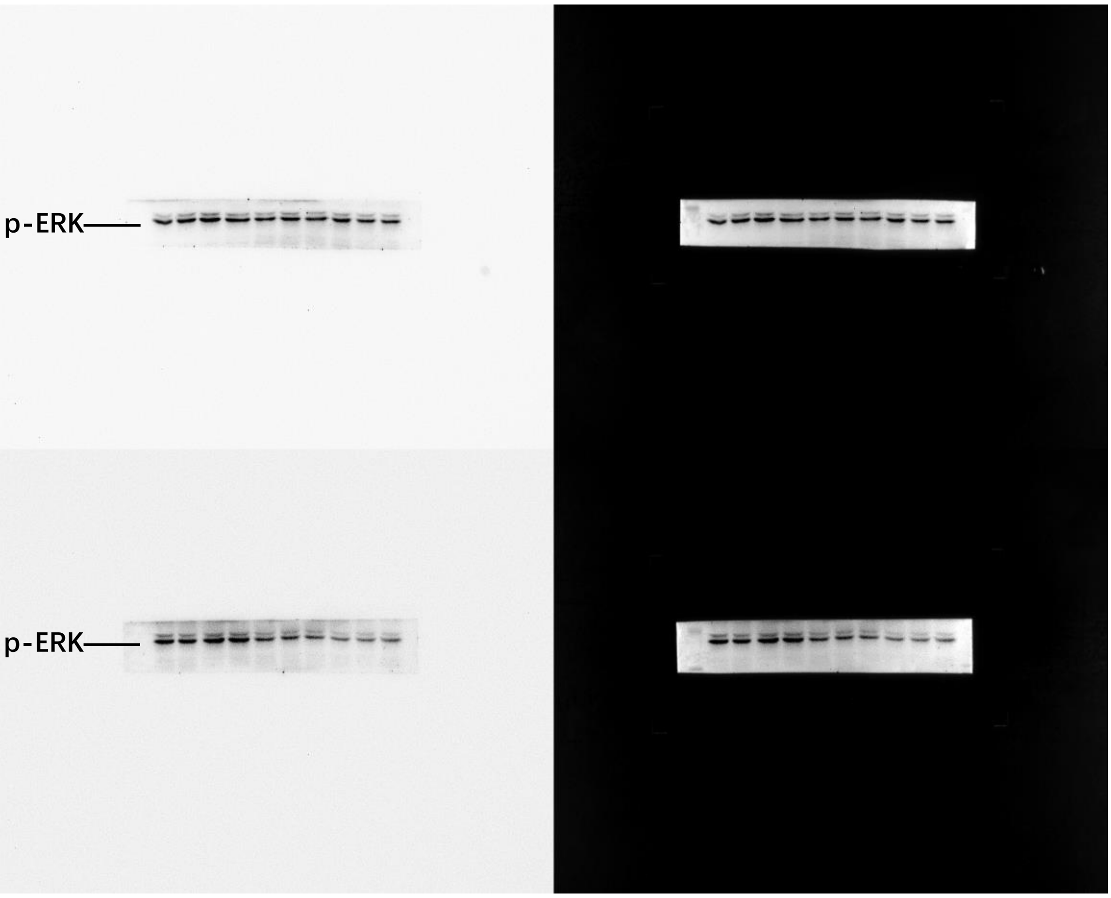

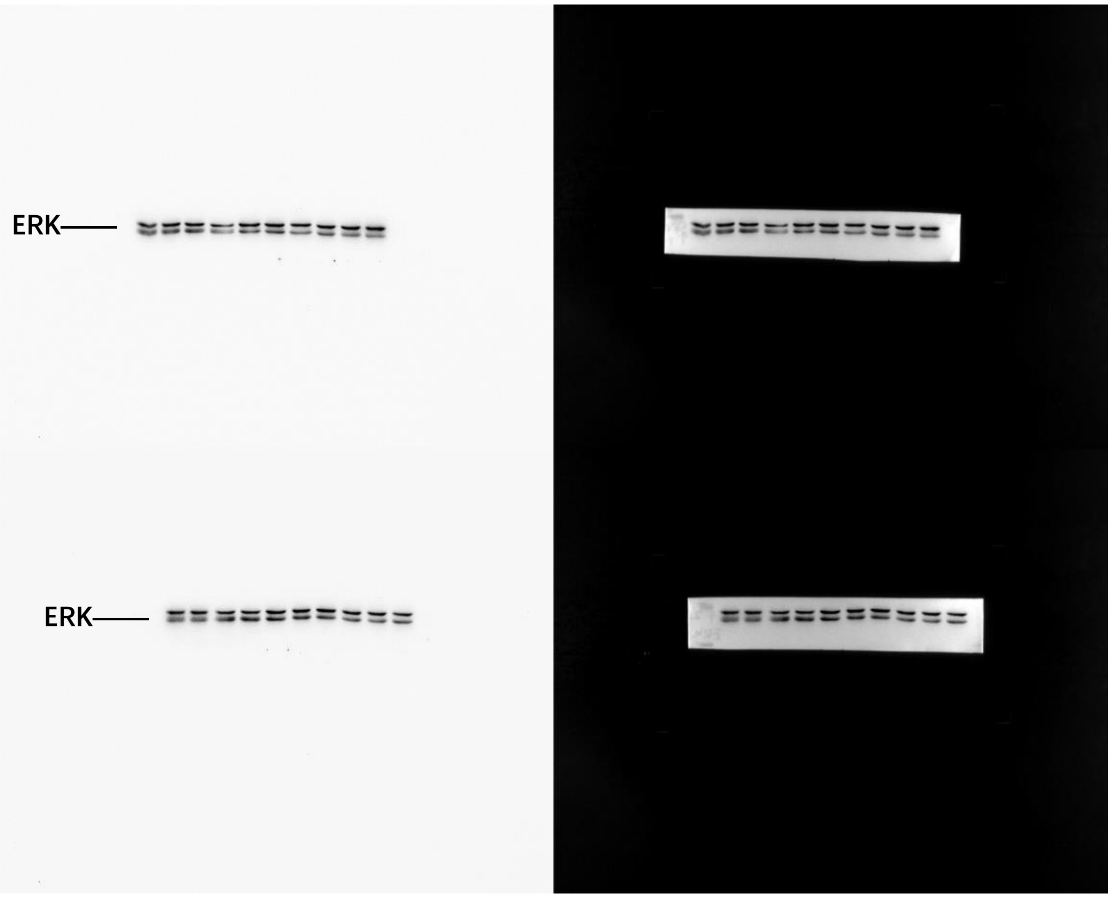

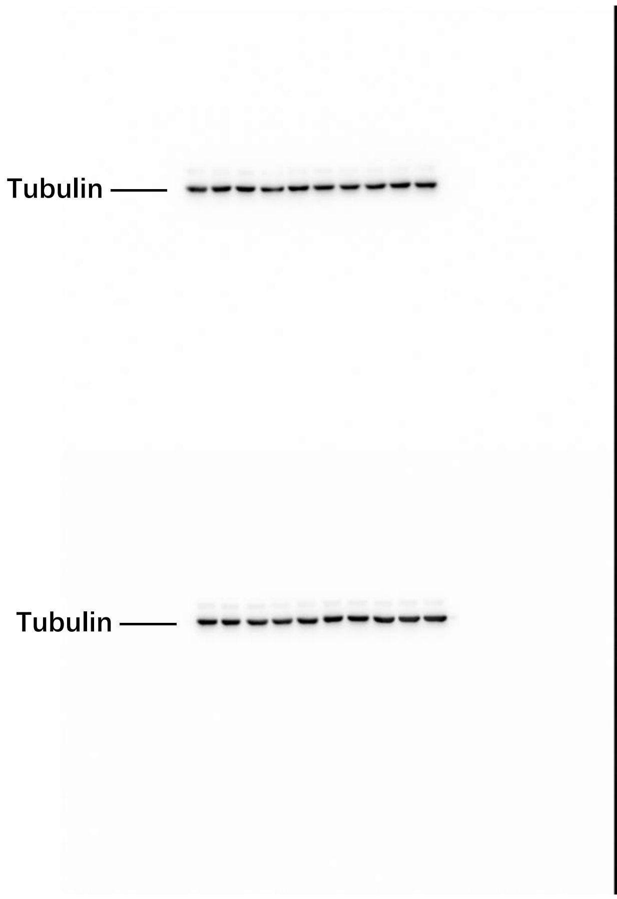
Tubulin
Tubulin

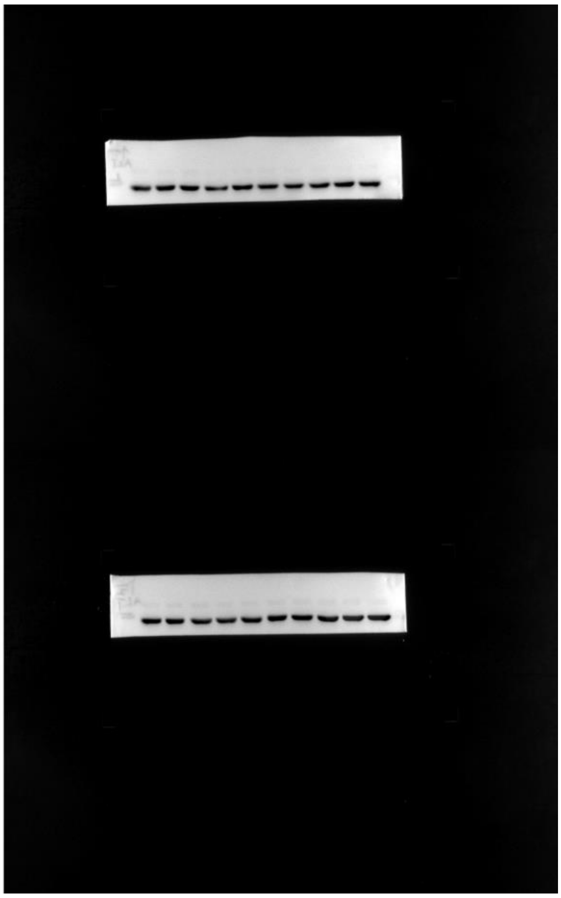

Figure 3F-claudin-5

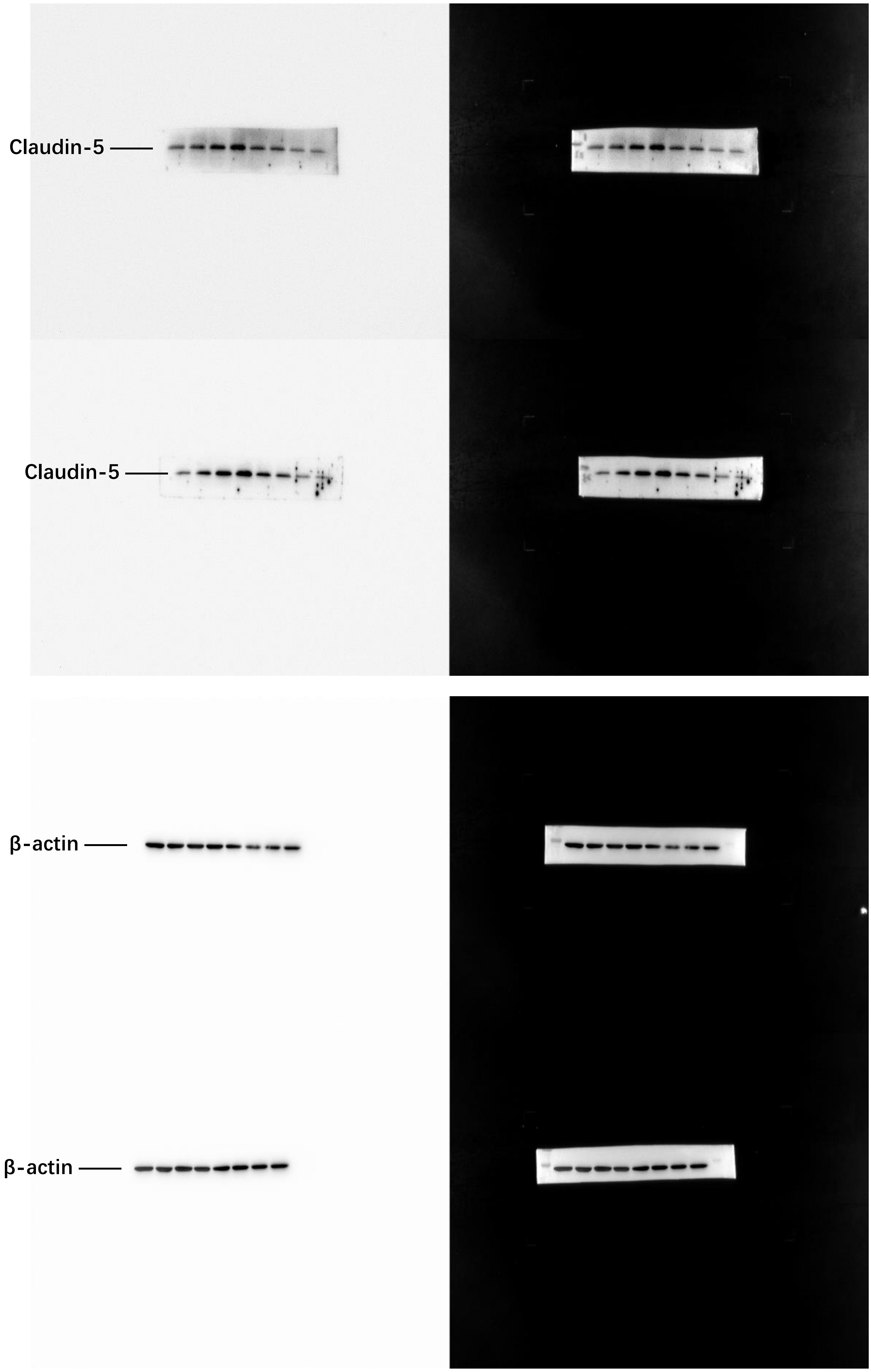

Figure 3F-VE-cadherin

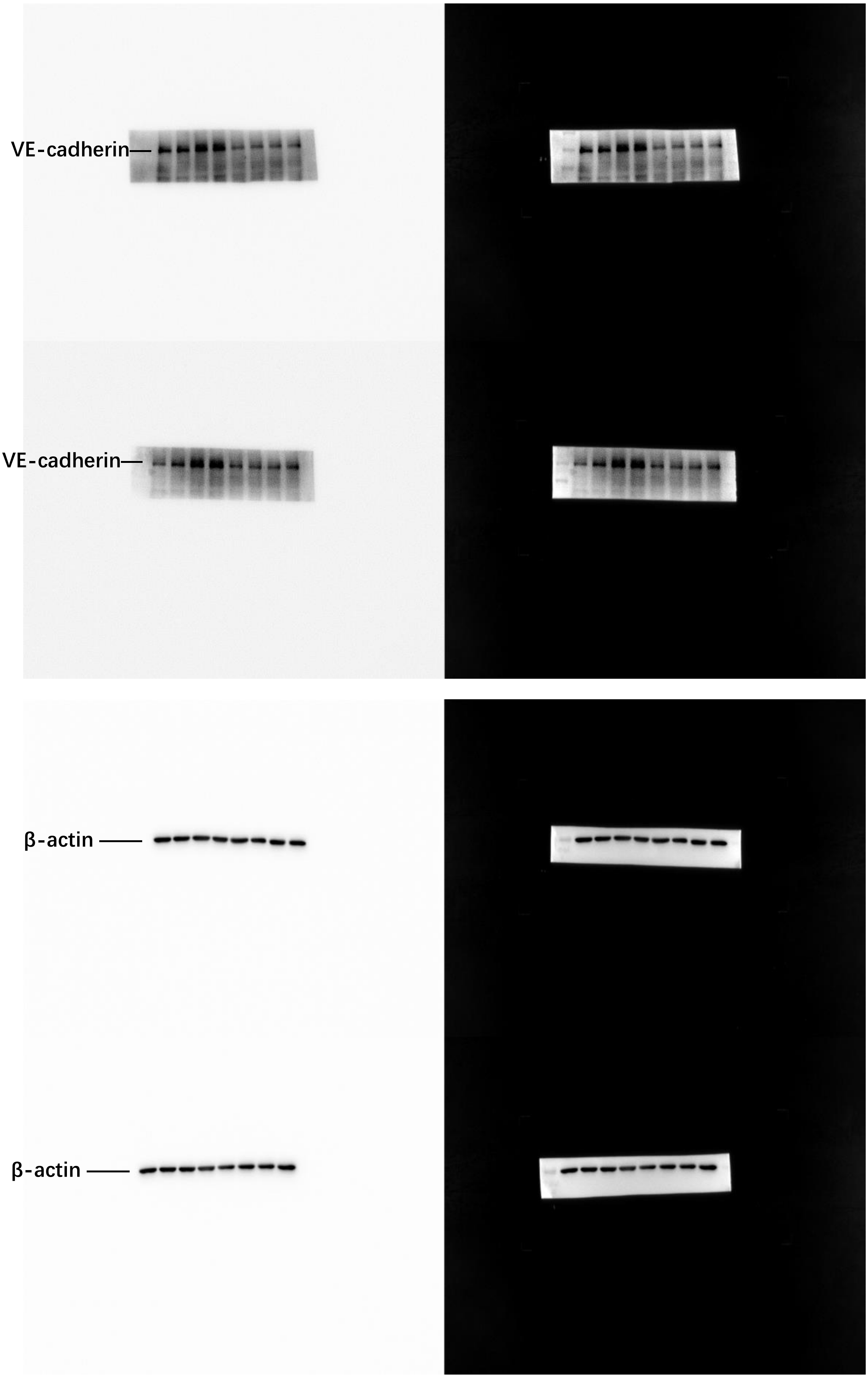

Figure 3F-p-AKT/AKT

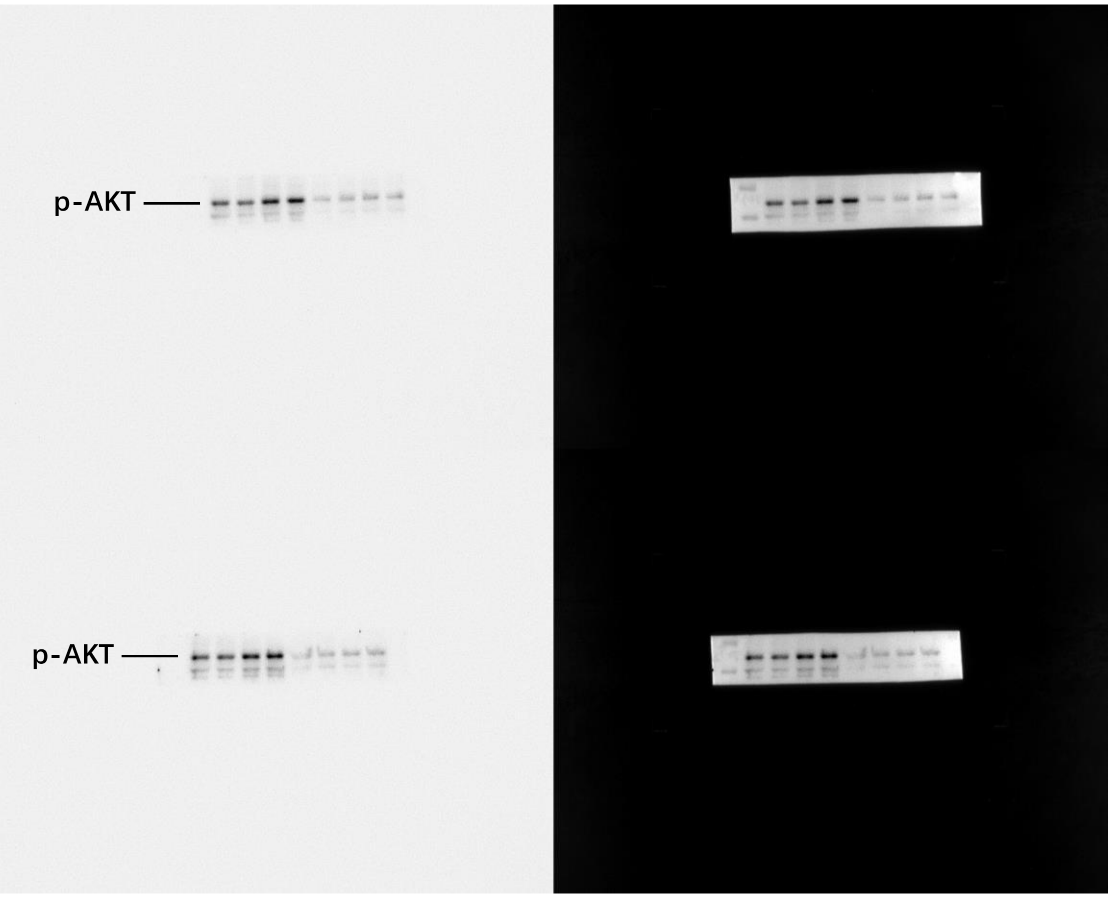

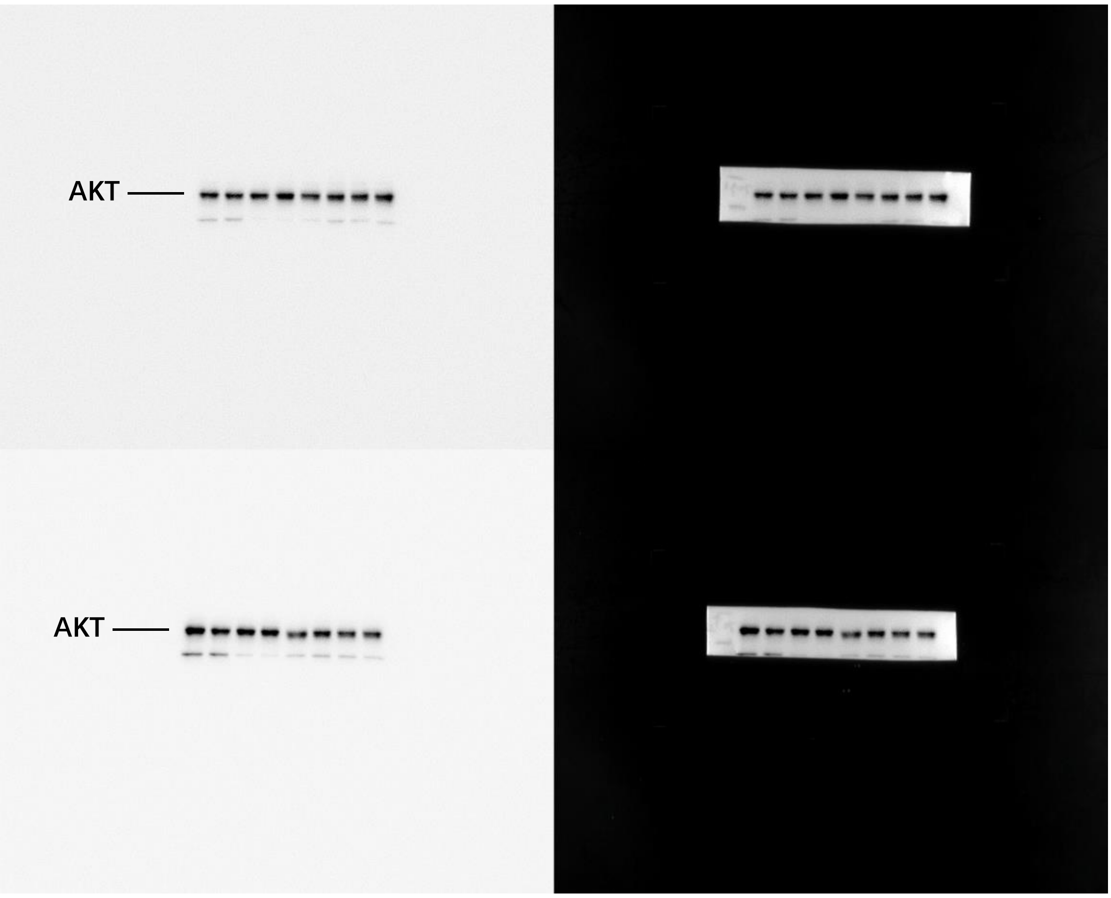

Figure 3G-claudin-5

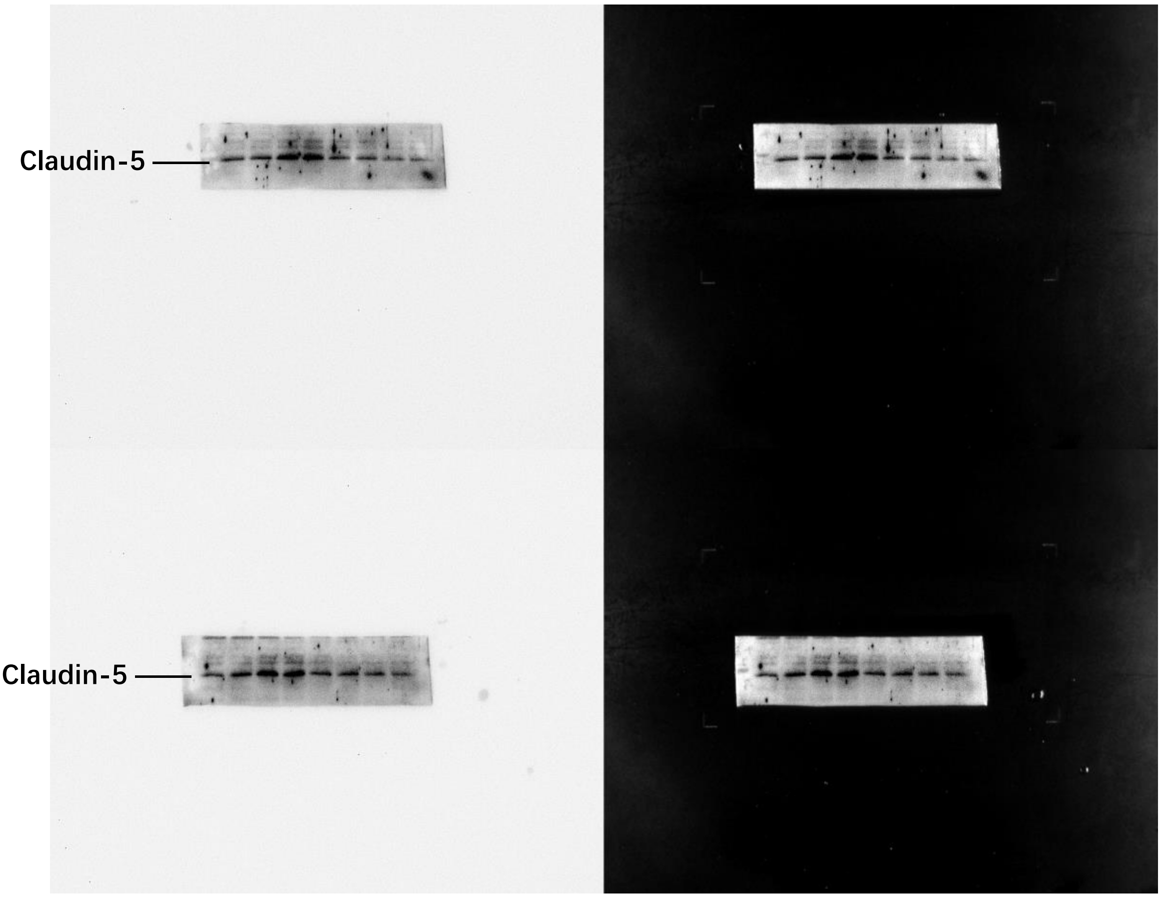

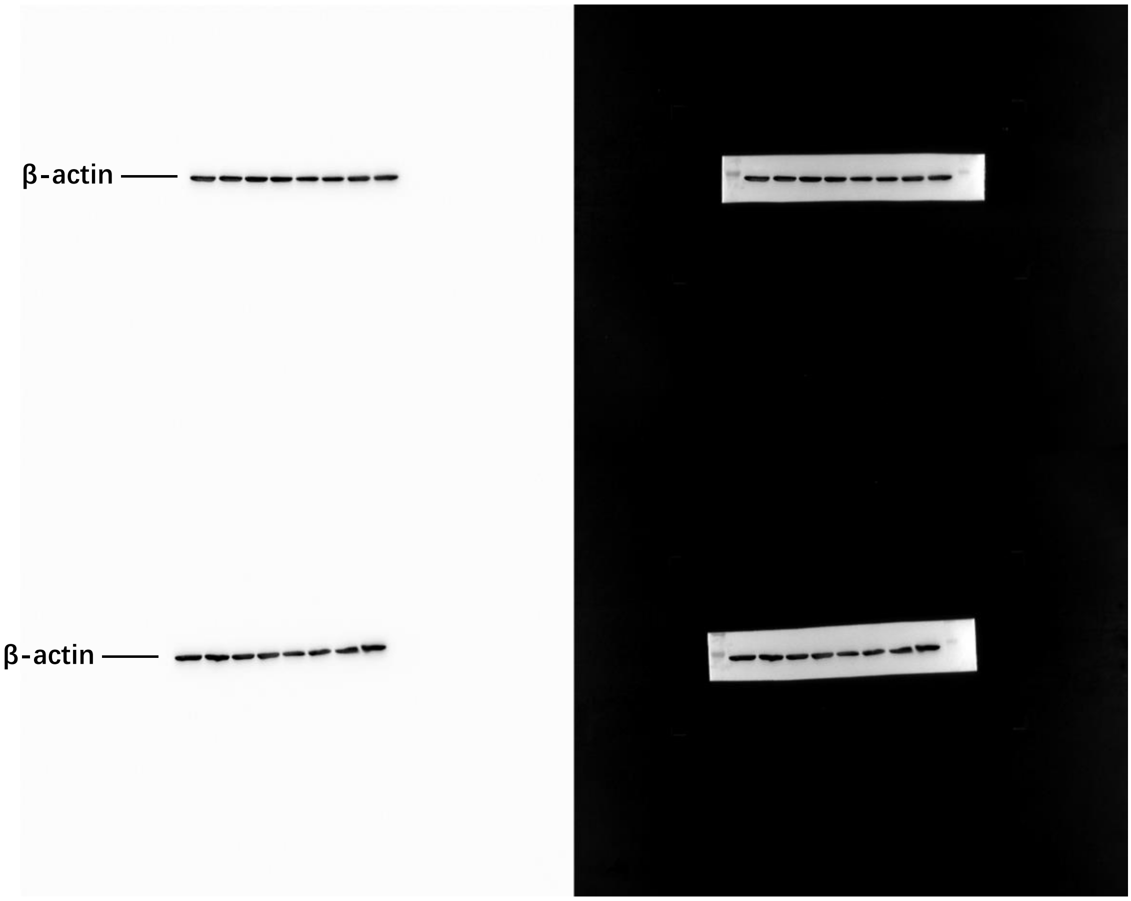

Figure 3G-VE-cadherin

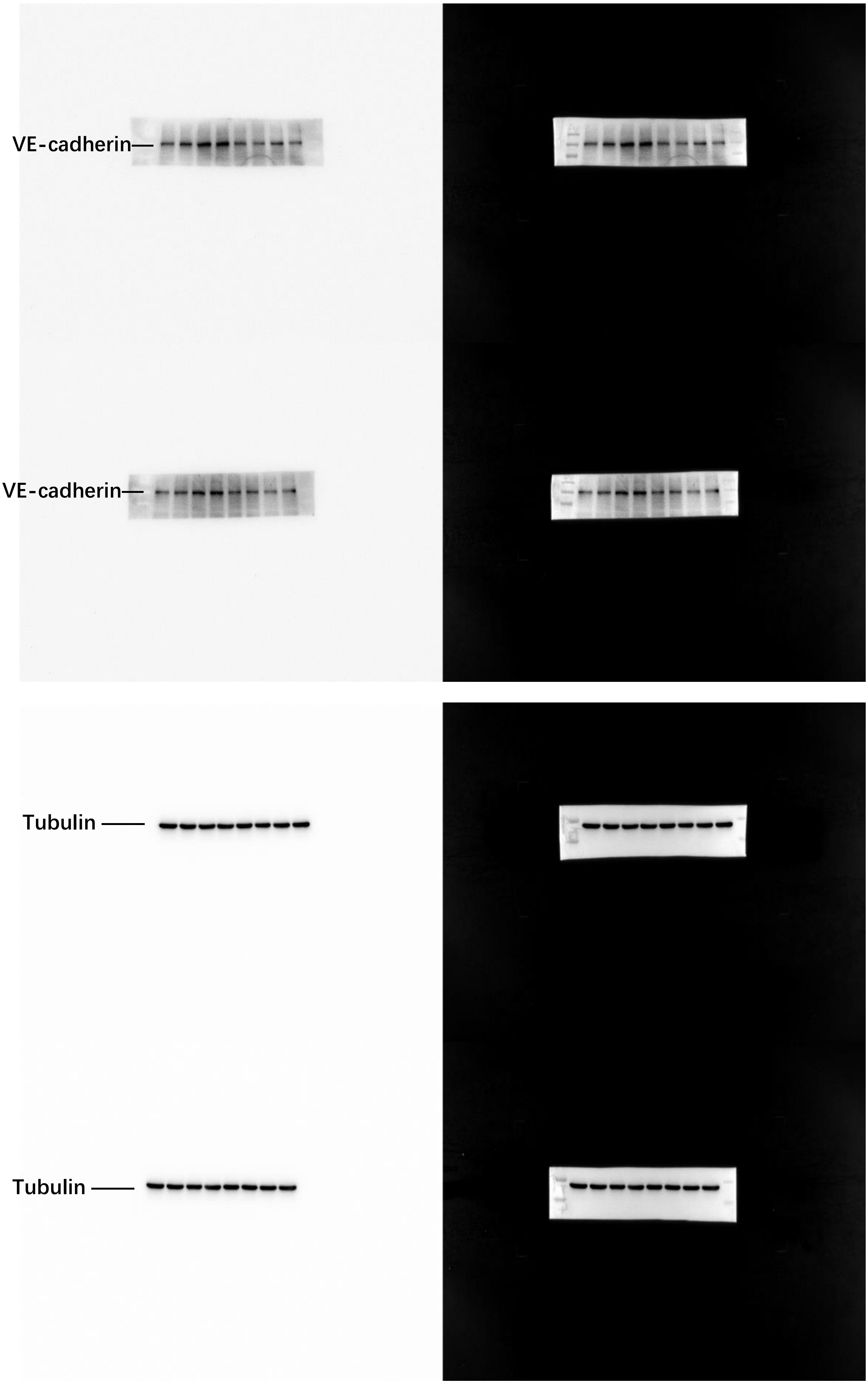

Figure 3G-p-ERK/ERK

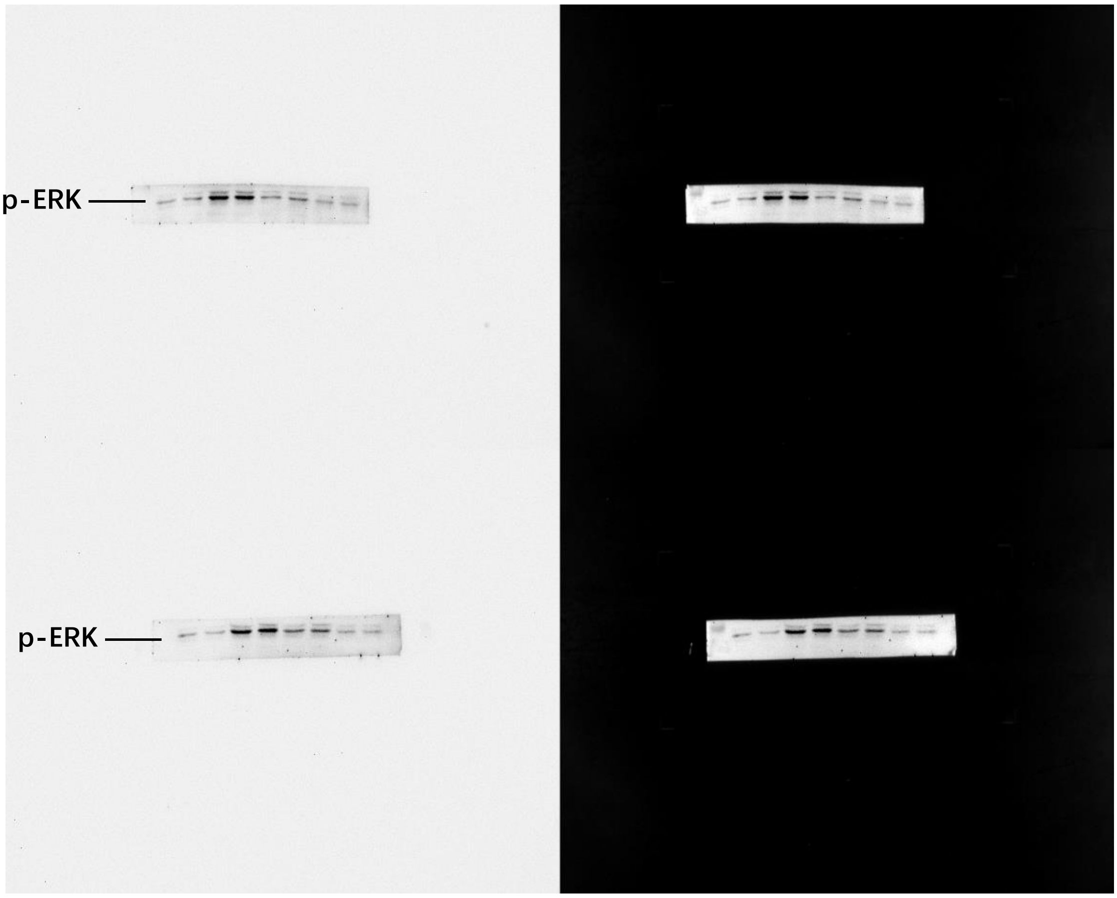

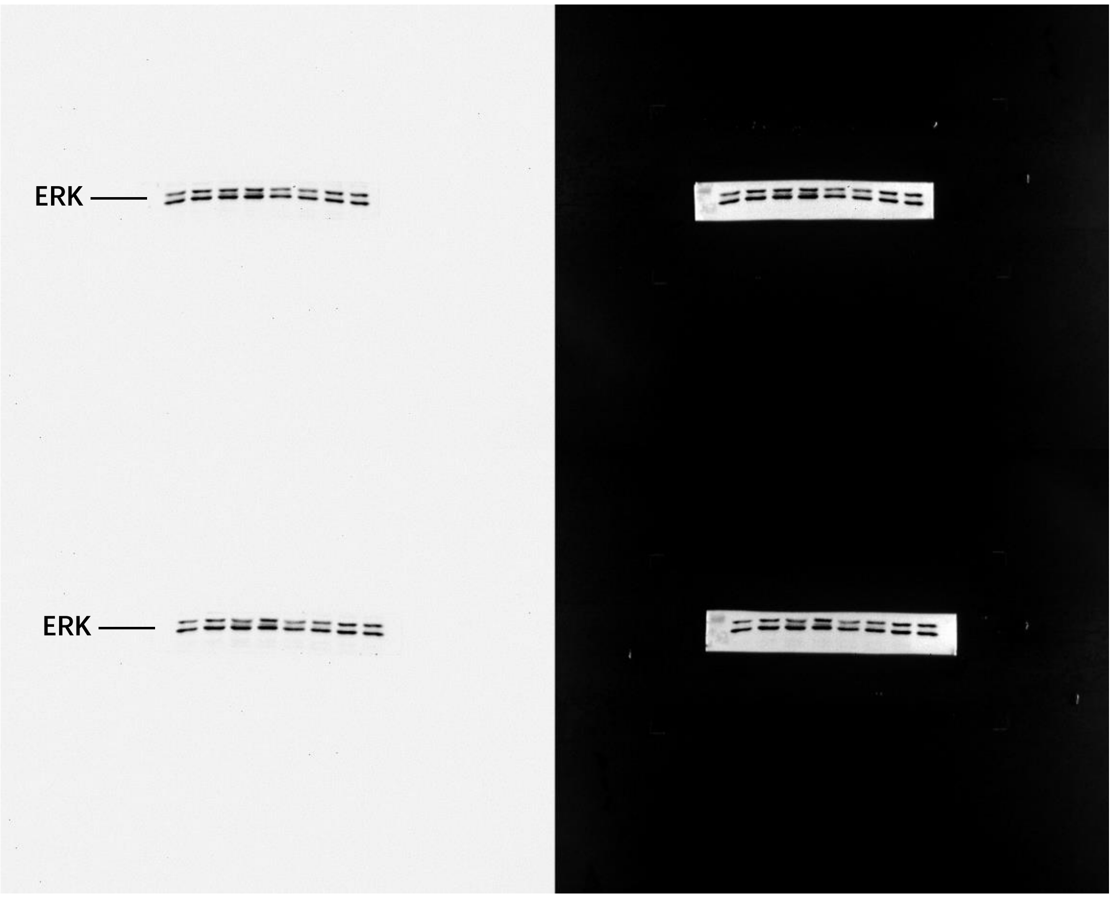

Figure 3H-Claudin-5

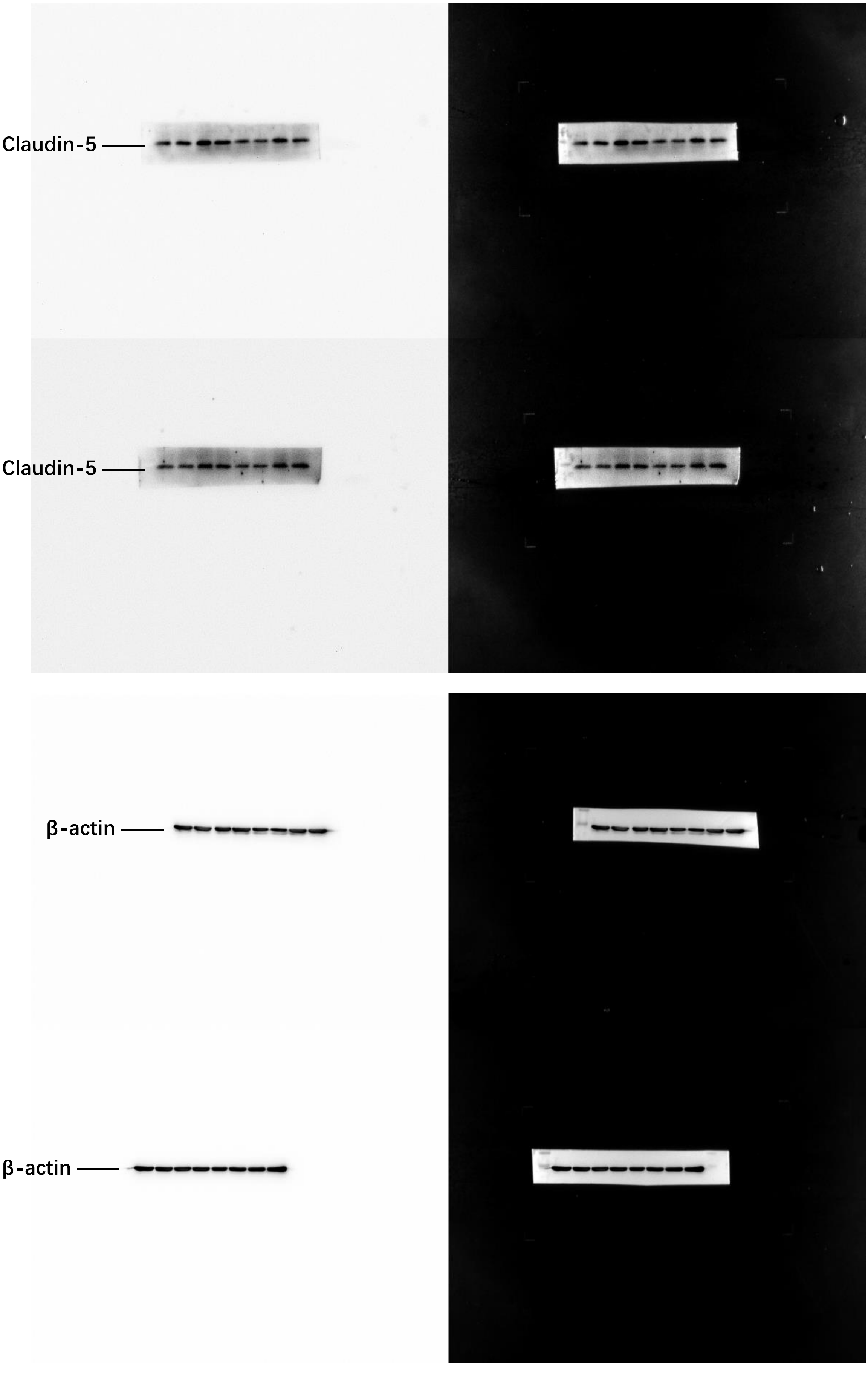

Figure 3H-VE-cadherin

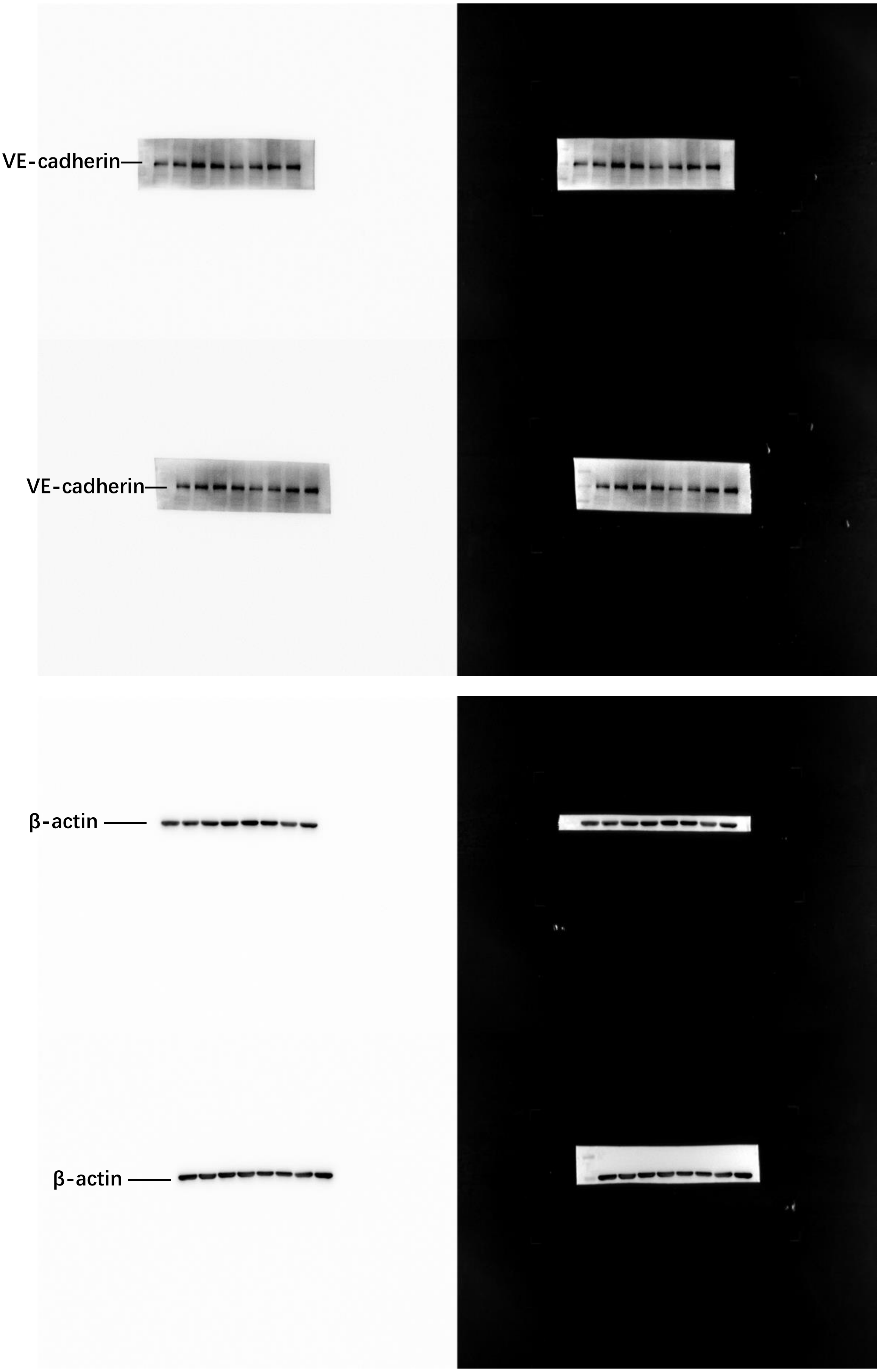

Figure 3H-p-JNK/JNK

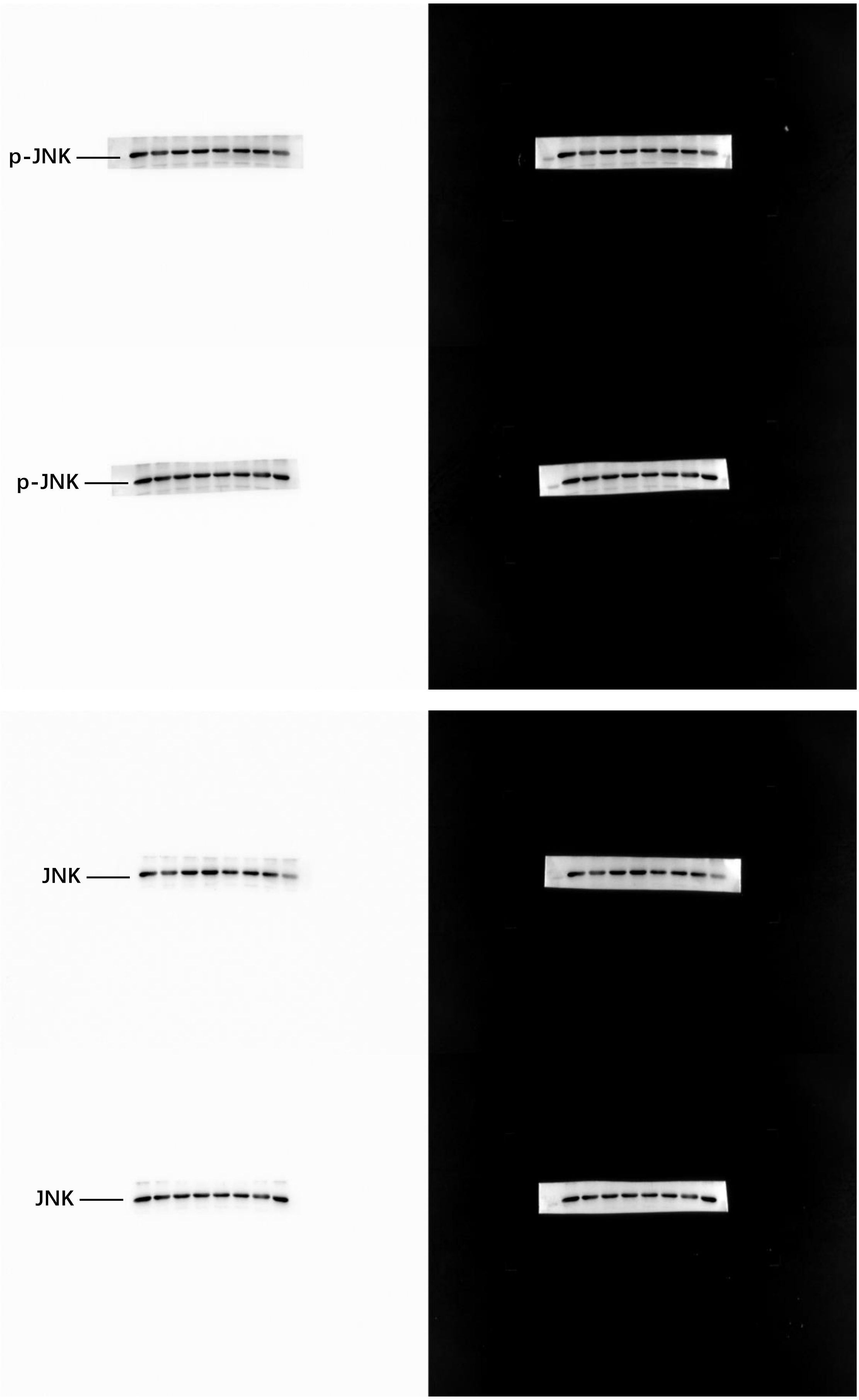

Figure 3I-Claudin-5

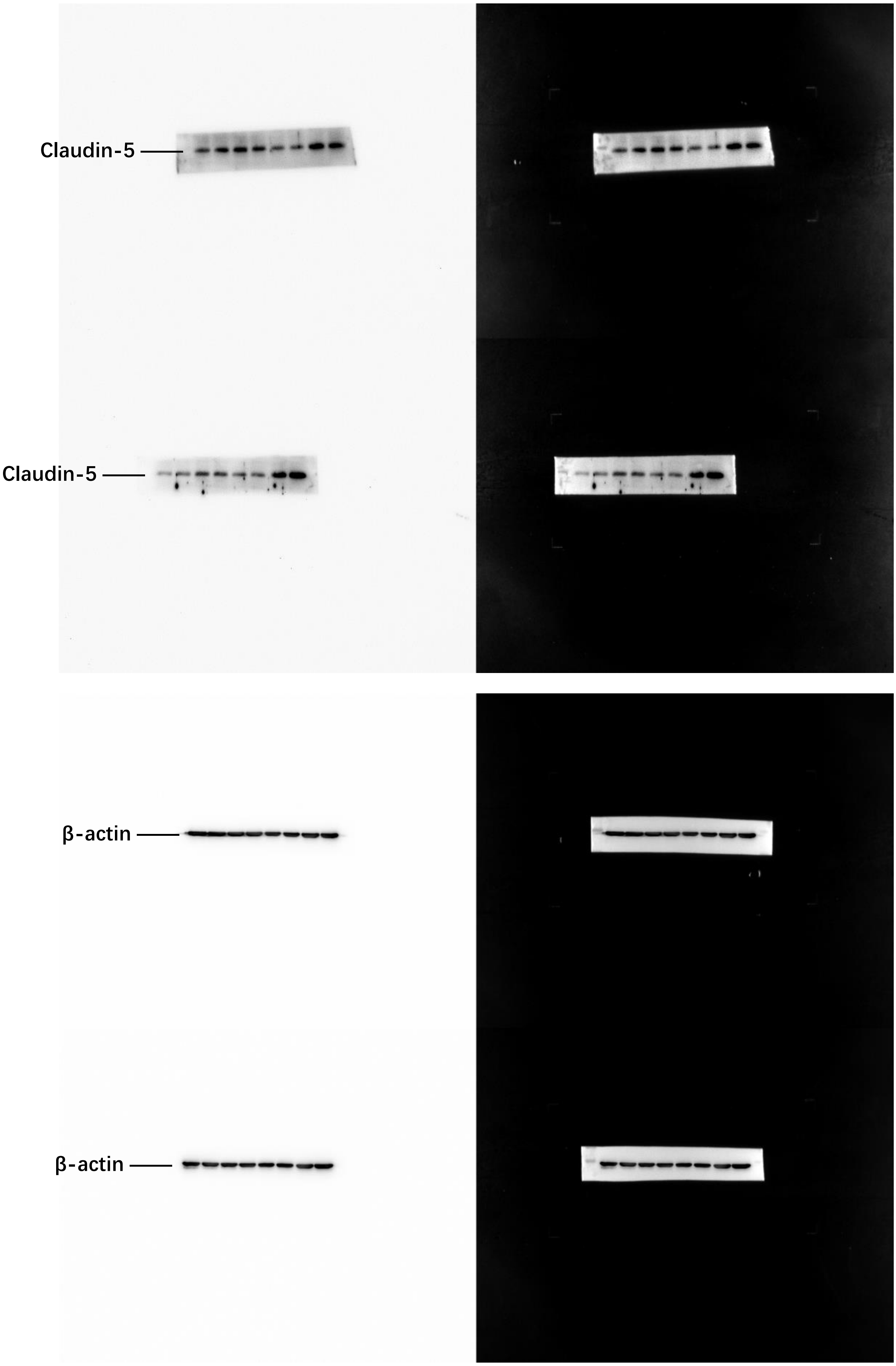

Figure 3I-VE-cadherin

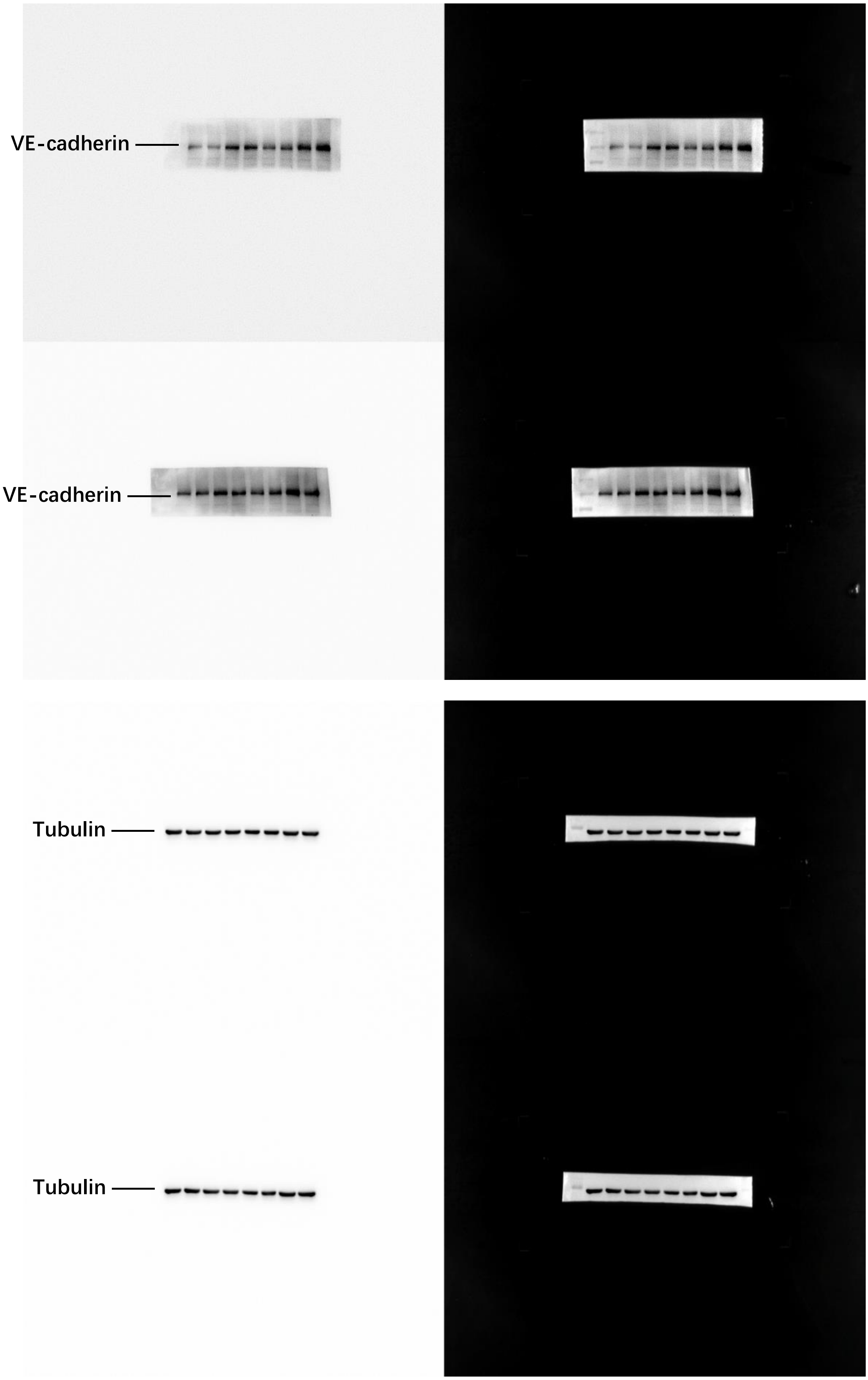

Figure 3I-p-p38/p38

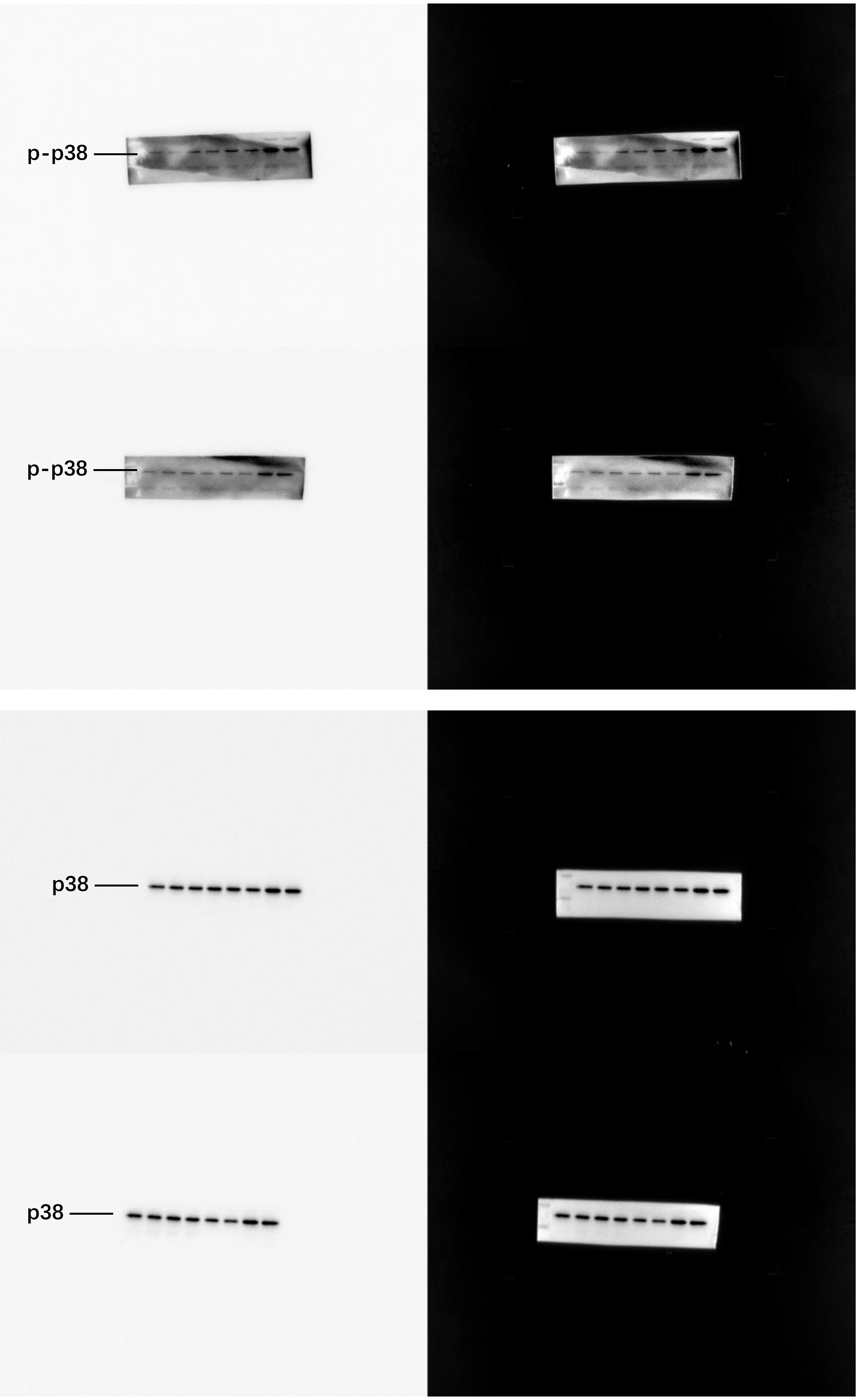

Figure 3J-p-AKT/ATK

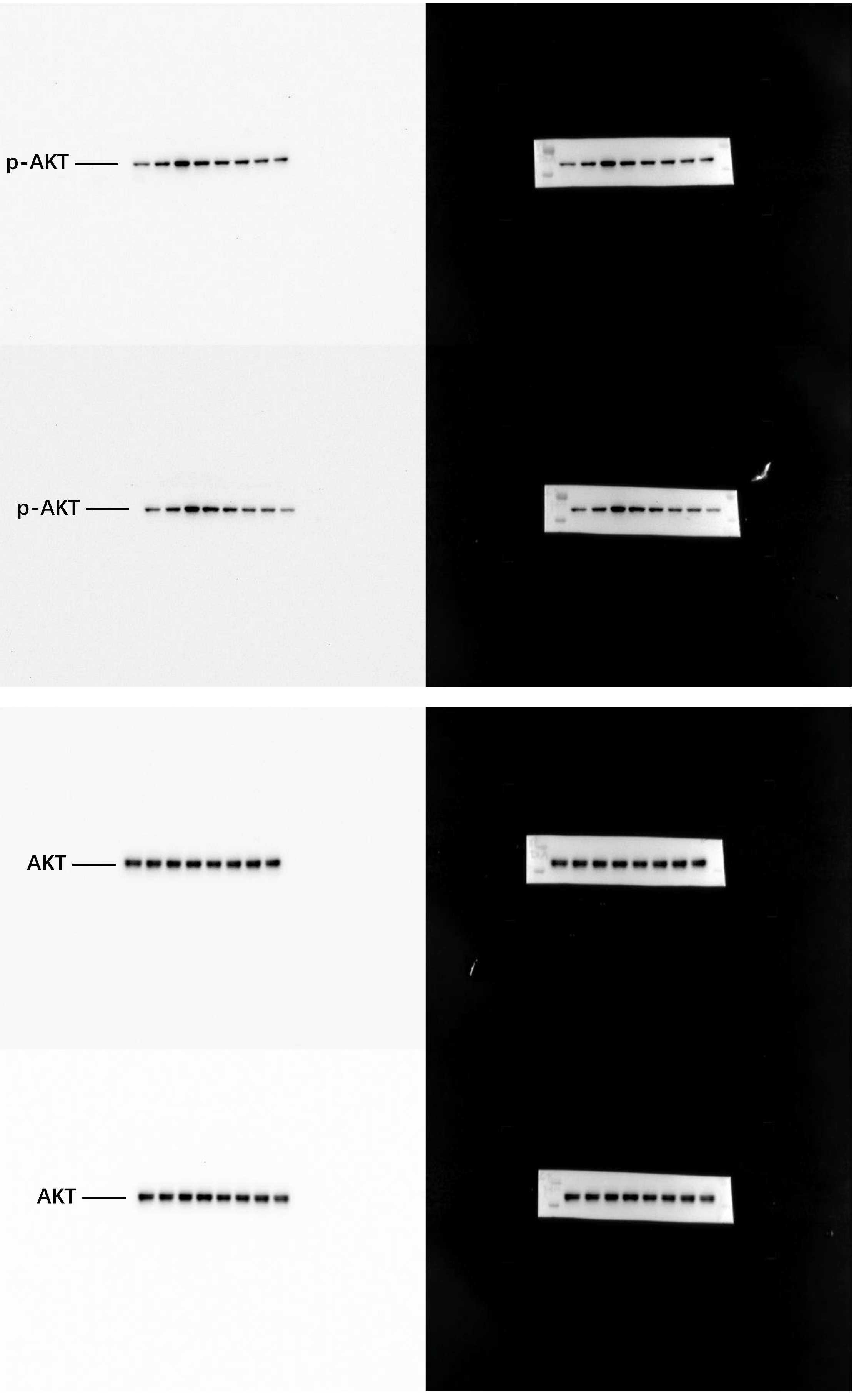

Figure 3J-p-ERK/ERK

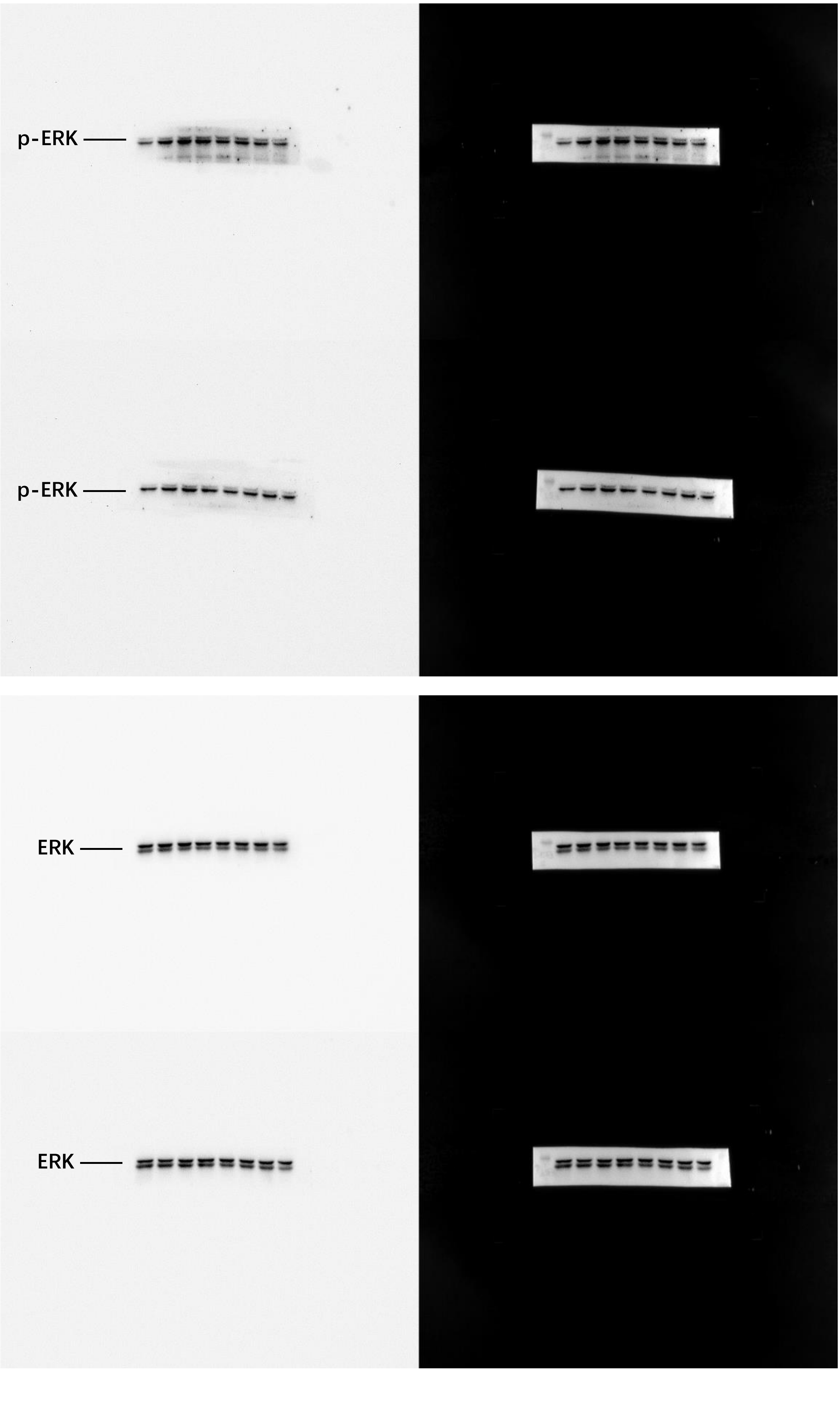

Figure 3J-Tubulin

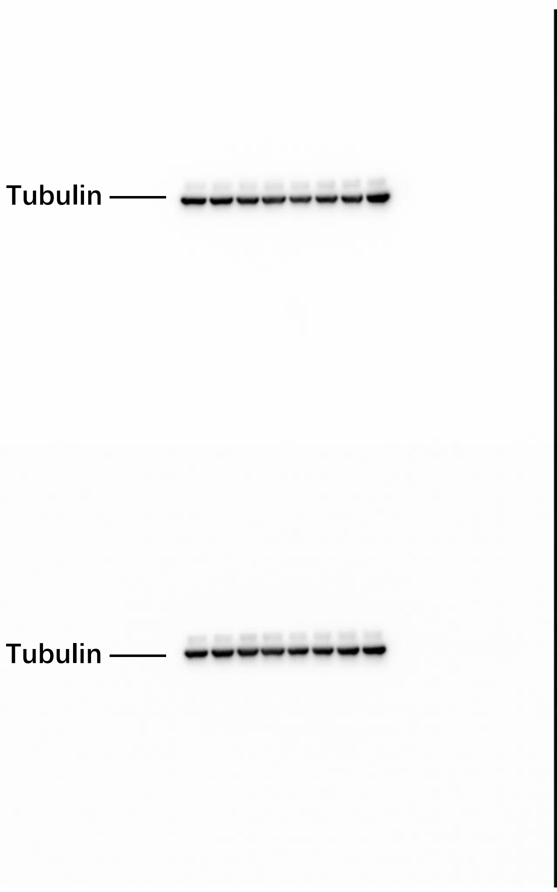

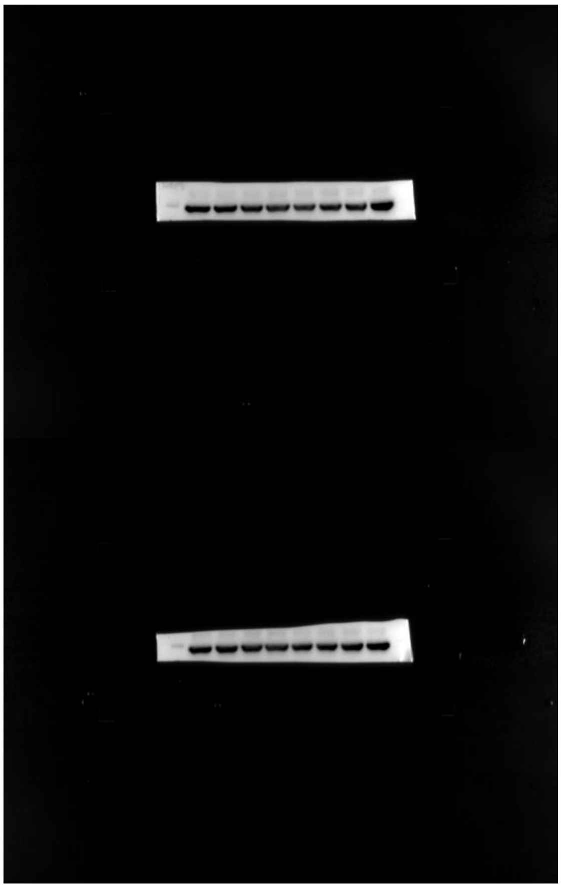

Supplement: Figure 3—source data 2. [file elife-96161-fig3-data2.zip › Figure 3-Source data2/Figure 3-Annotated western blots.pdf]
