# Supplementary material for: Neurons enhance blood–brain barrier function via upregulating claudin-5 and VE-cadherin expression due to glial cell line-derived neurotrophic factor secretion
Source: eLife. 2024 Oct 30;13:RP96161. doi: 10.7554/eLife.96161 (PMC11524583; doi:10.7554/eLife.96161)

Figure 4A-p-FOXO1/FOXO1

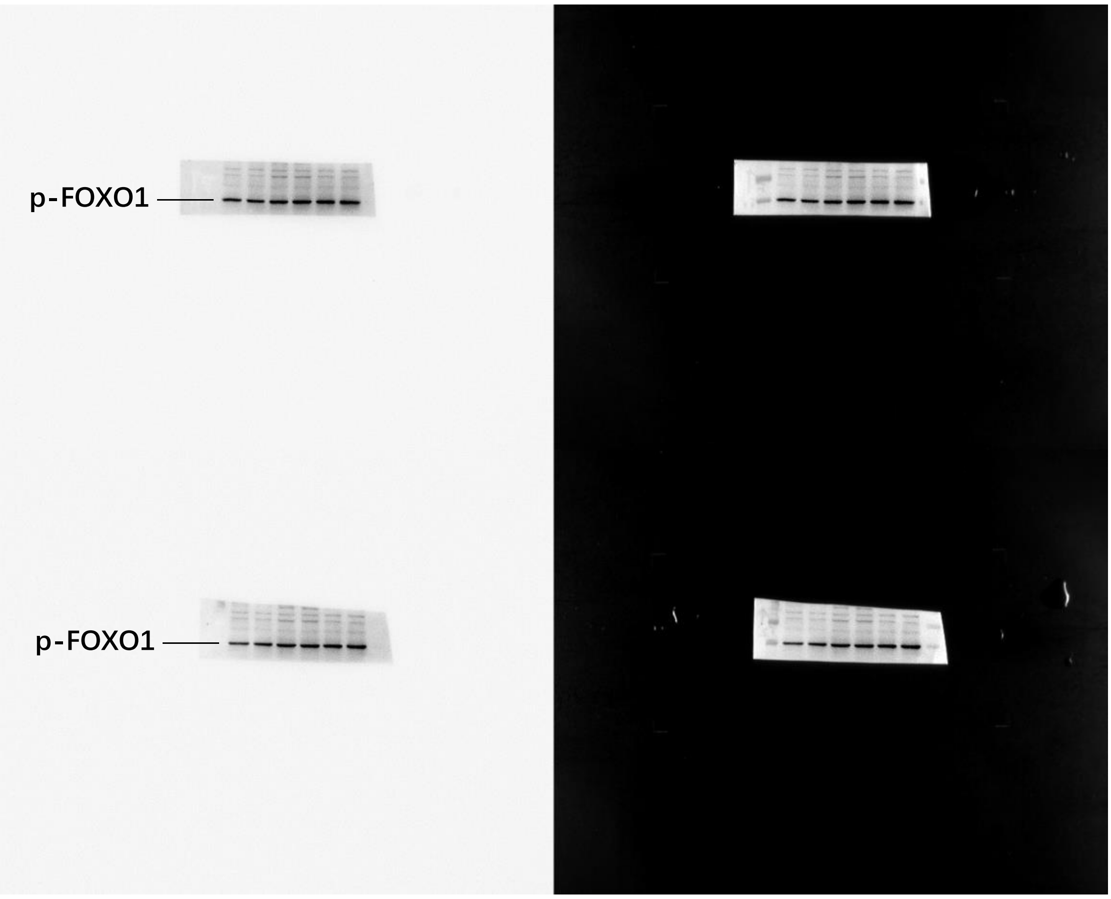

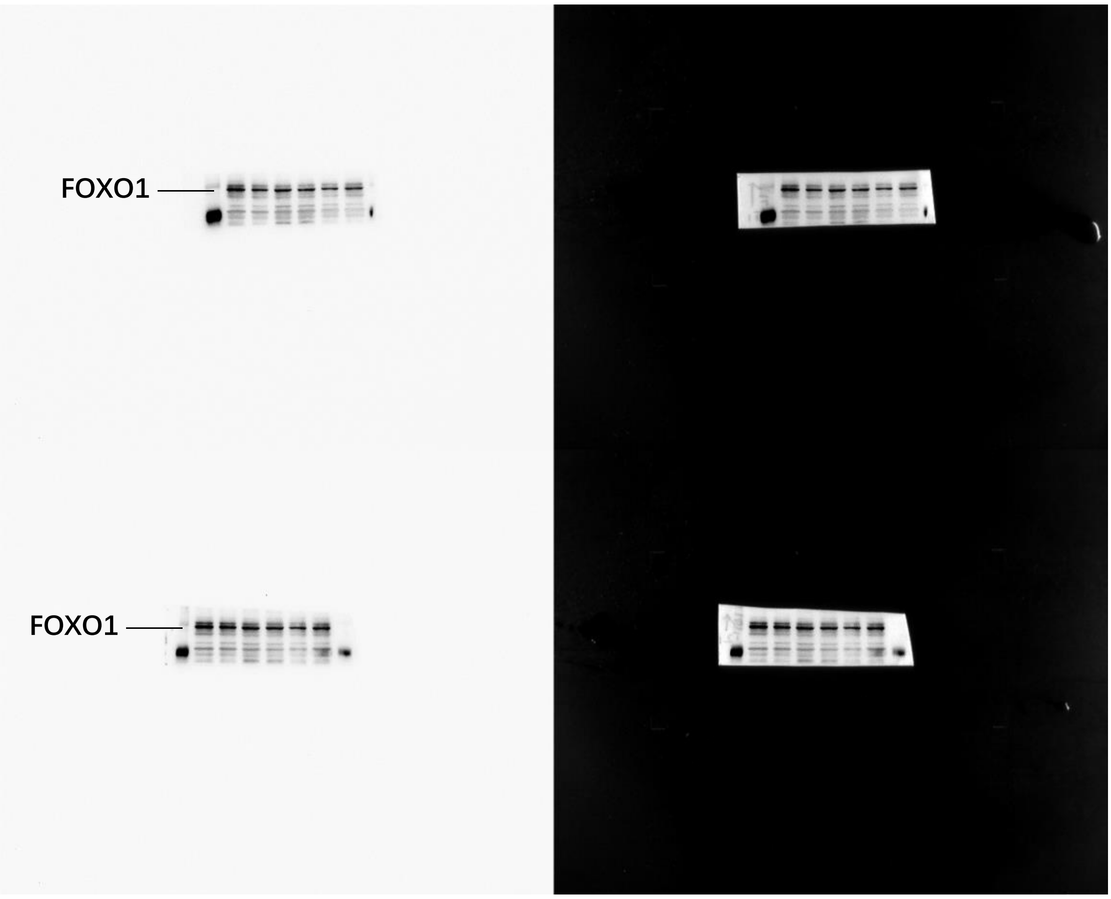

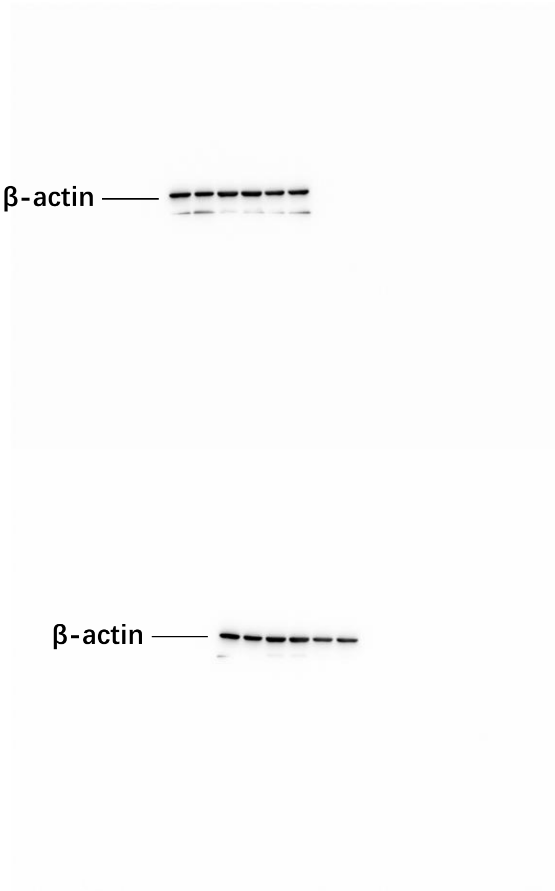
β-actin
β-actin

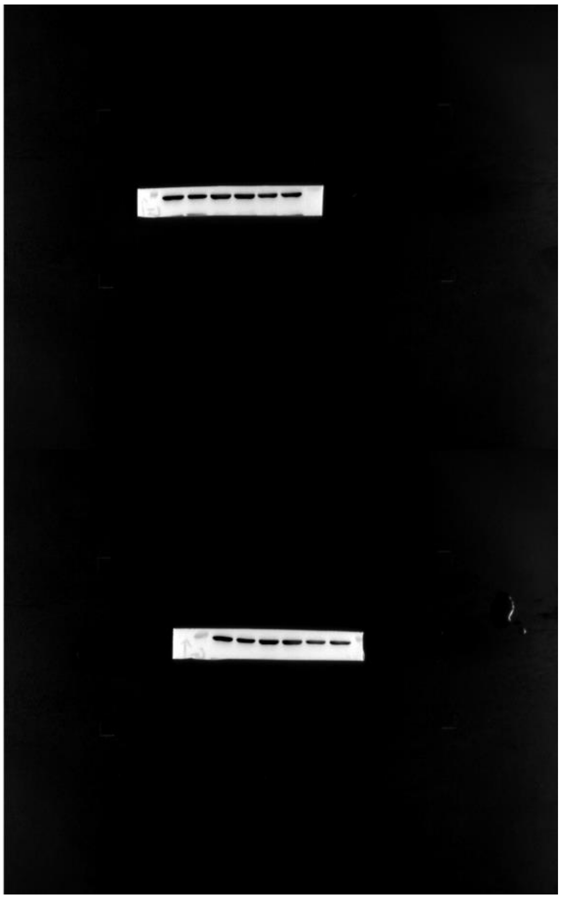

Figure 4B-p-FOXO1, FOXO1, GAPDH

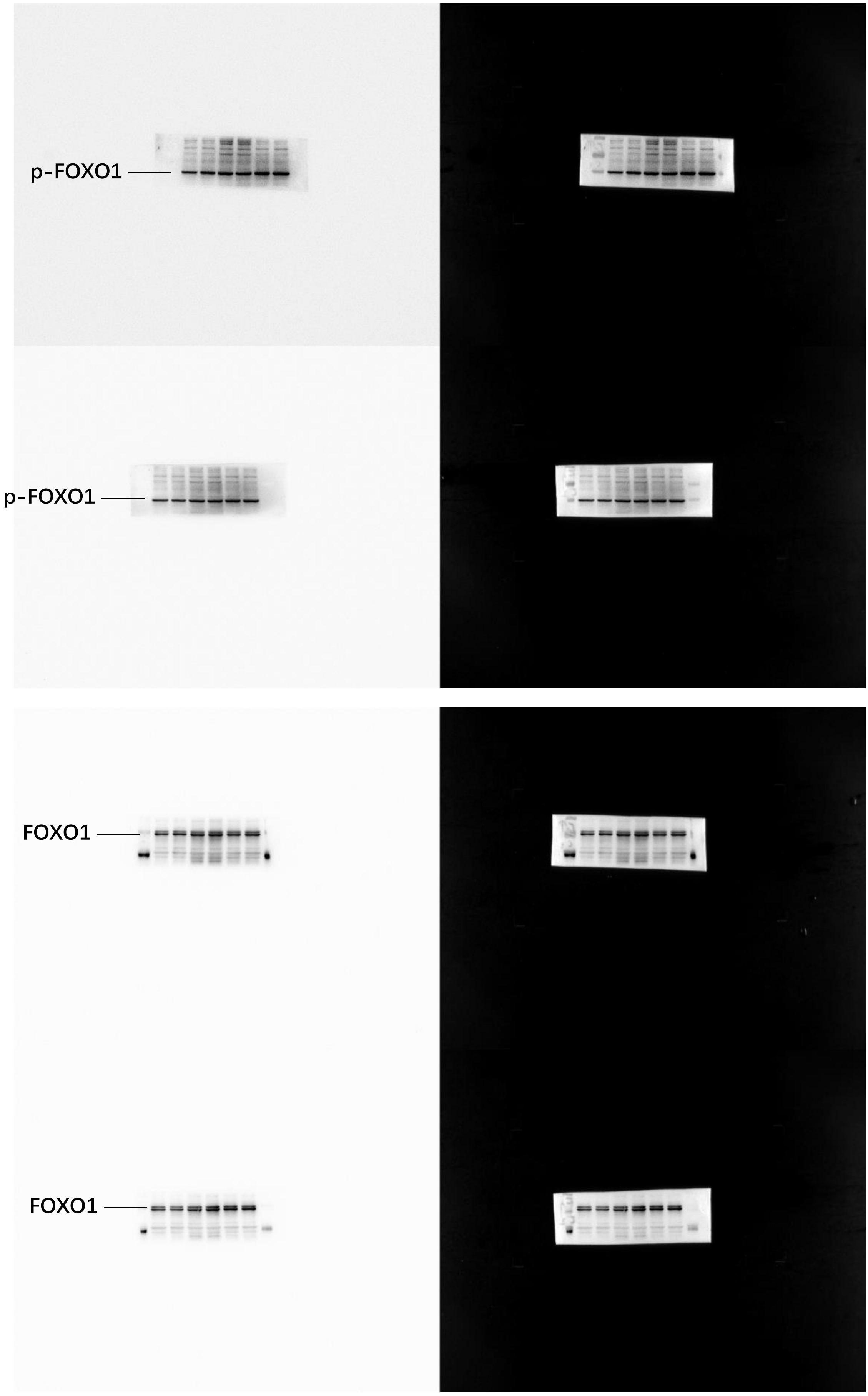

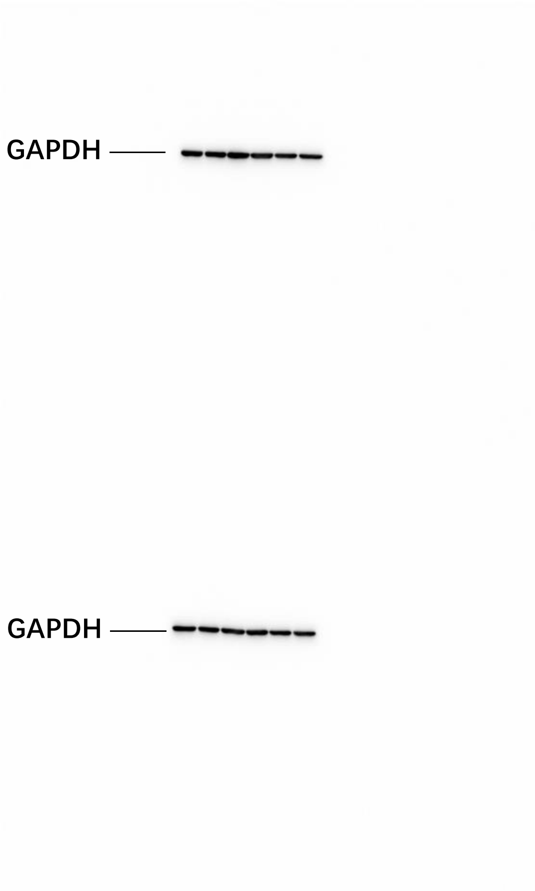
GAPDH
GAPDH
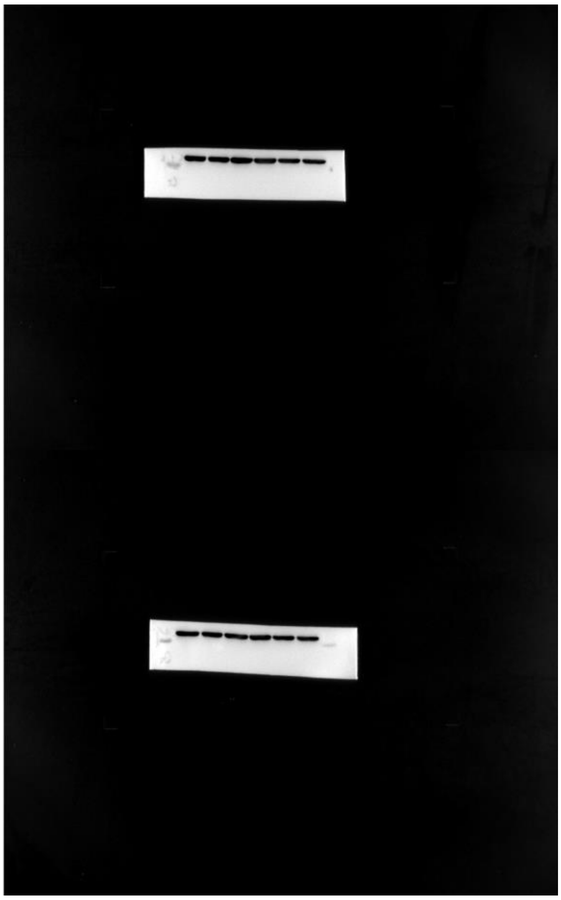

Figure 4B- FOXO1, Lamin B

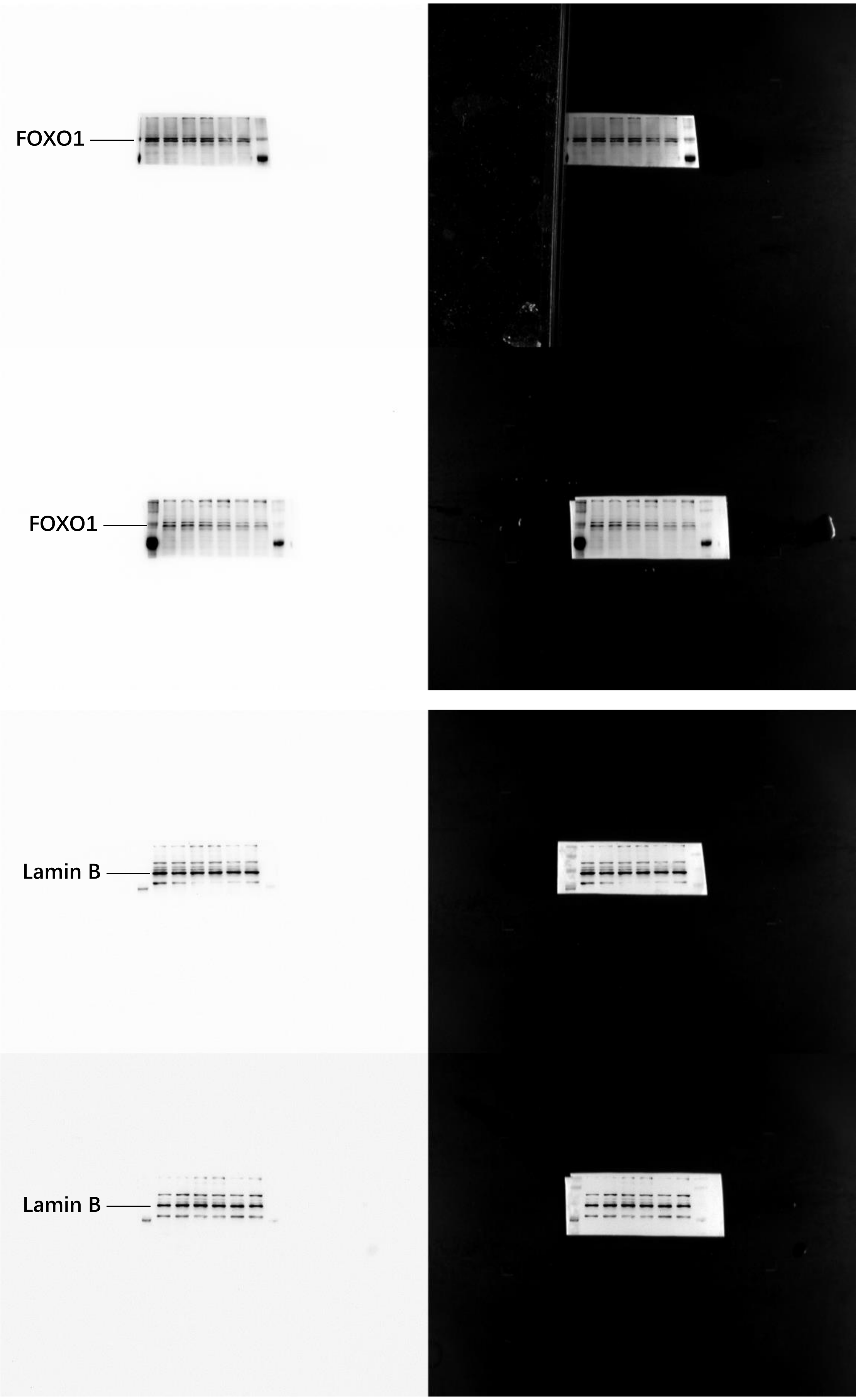

Figure 4C- FOXO1, β-actin

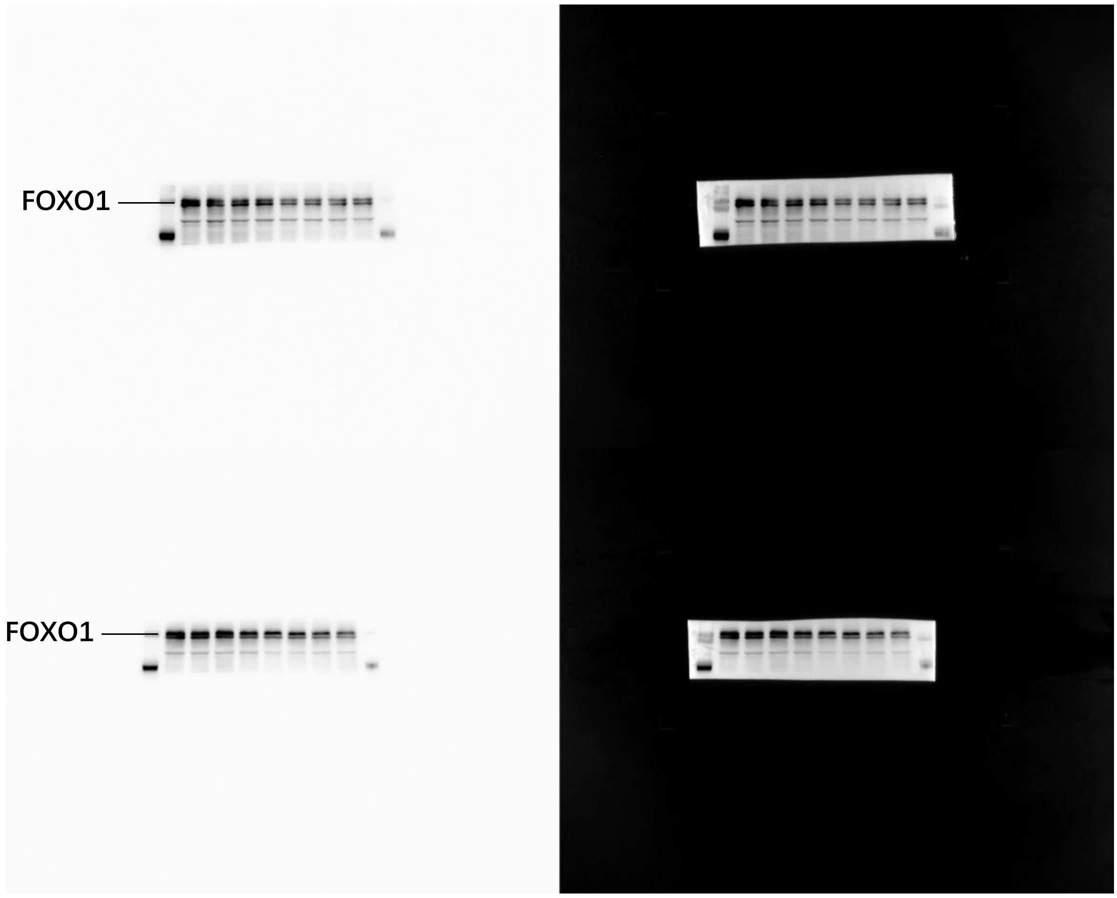

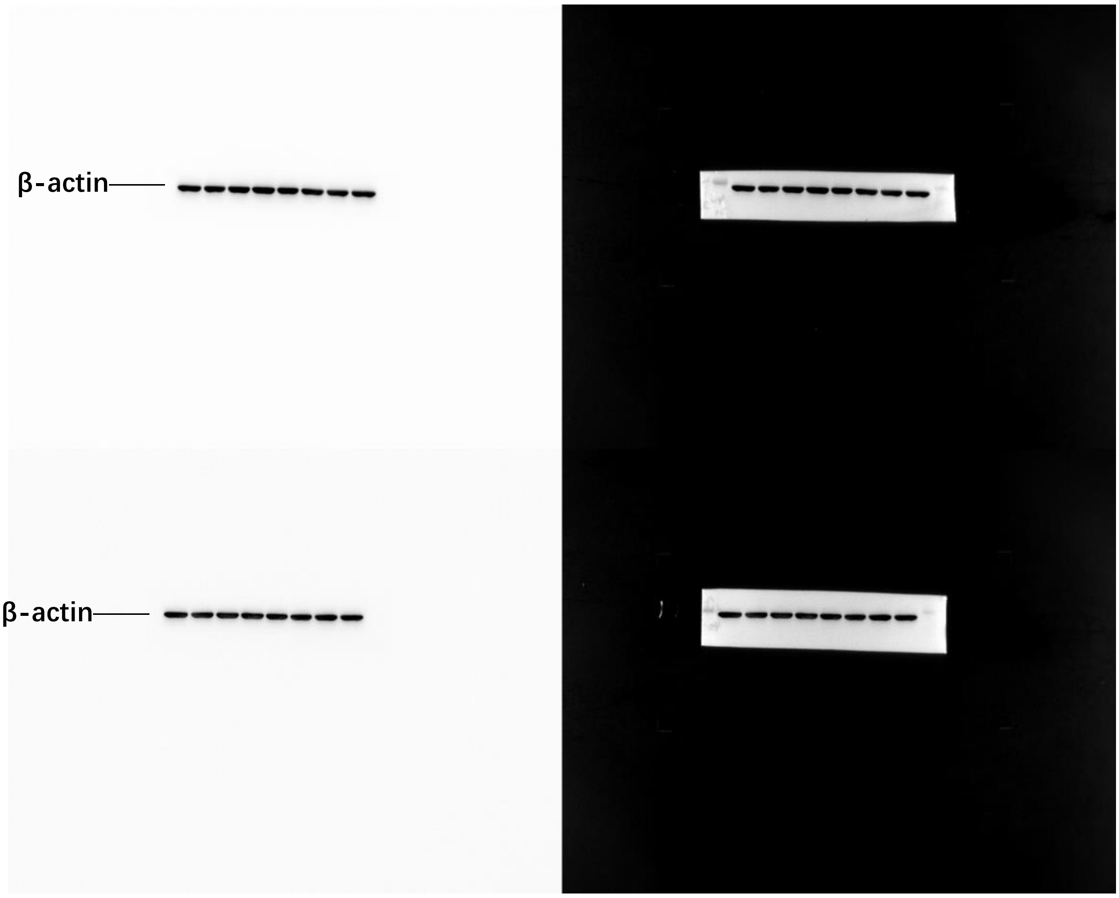

Figure 4C- FOXO1, Lamin B

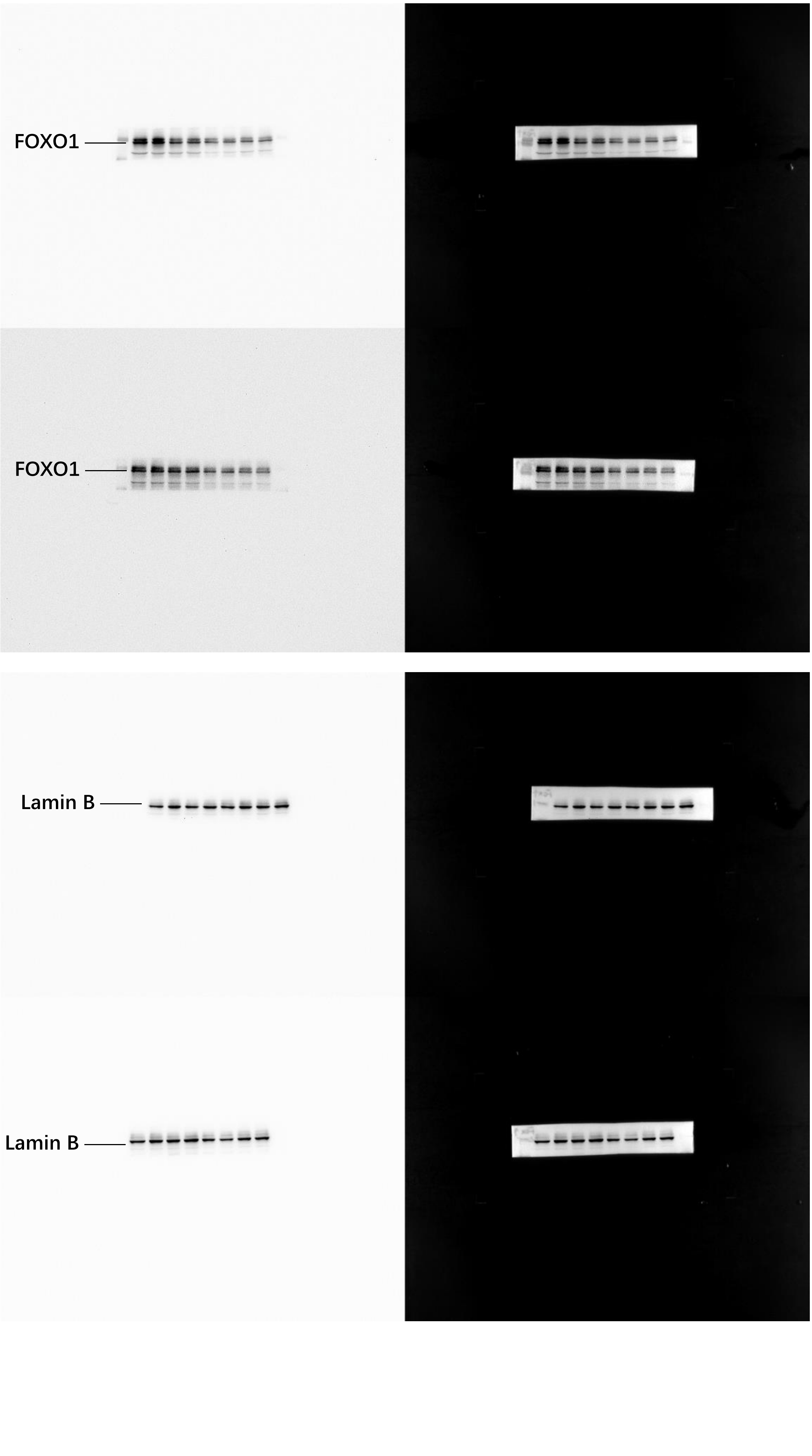

Figure 4D-Claudin-5

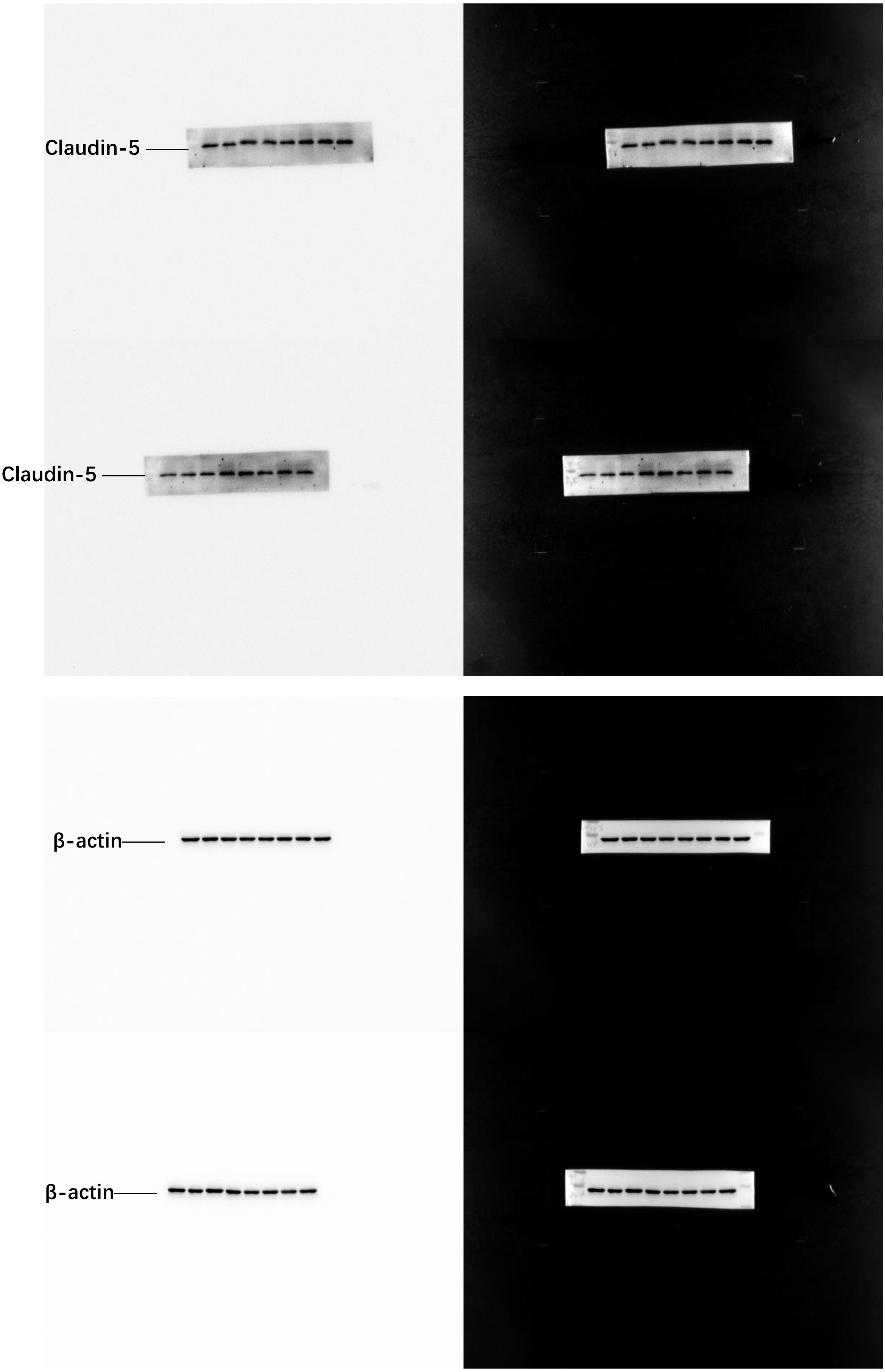

Figure 4D-VE-cadherin

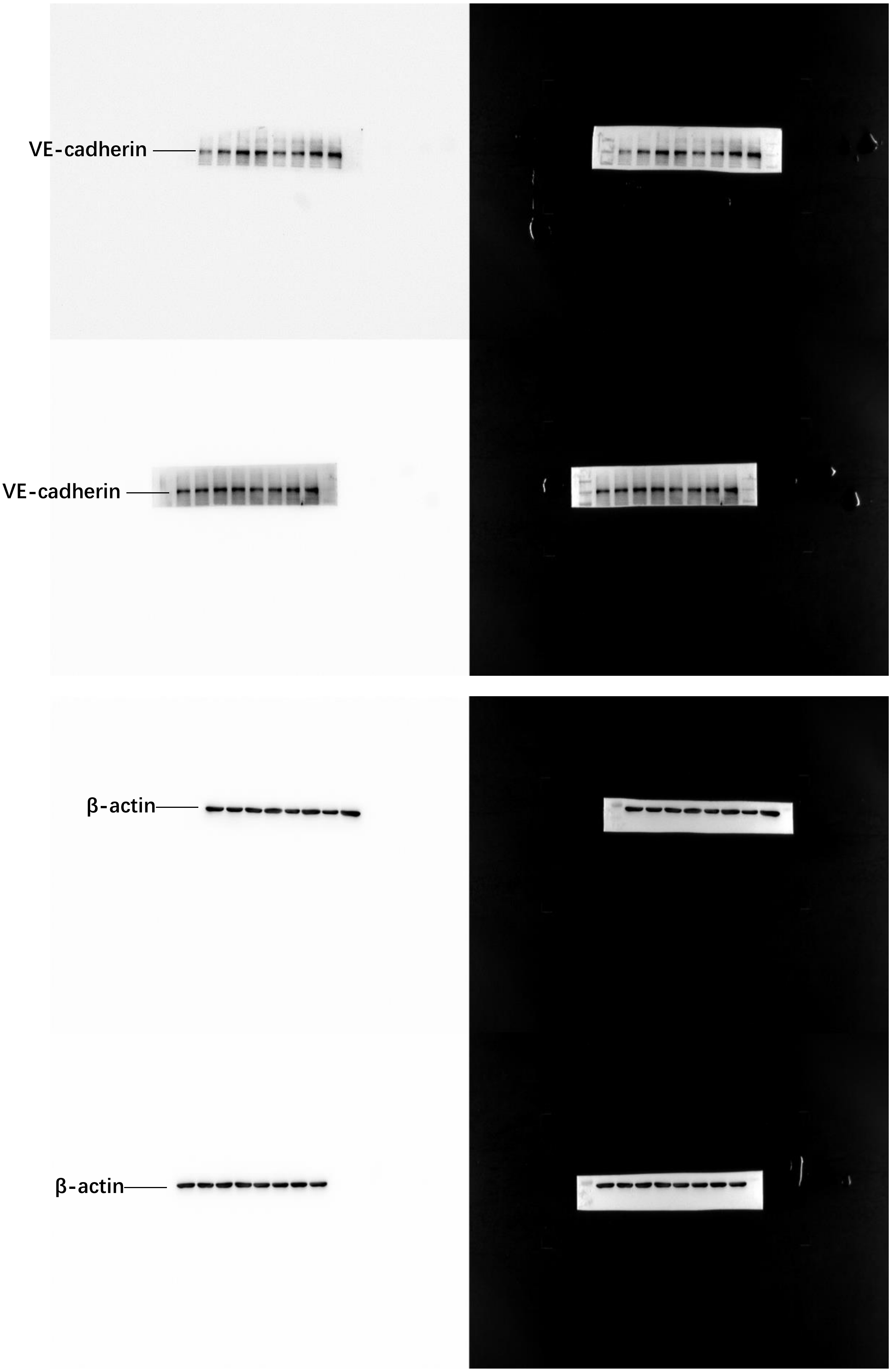

Figure 4E-Claudin-5

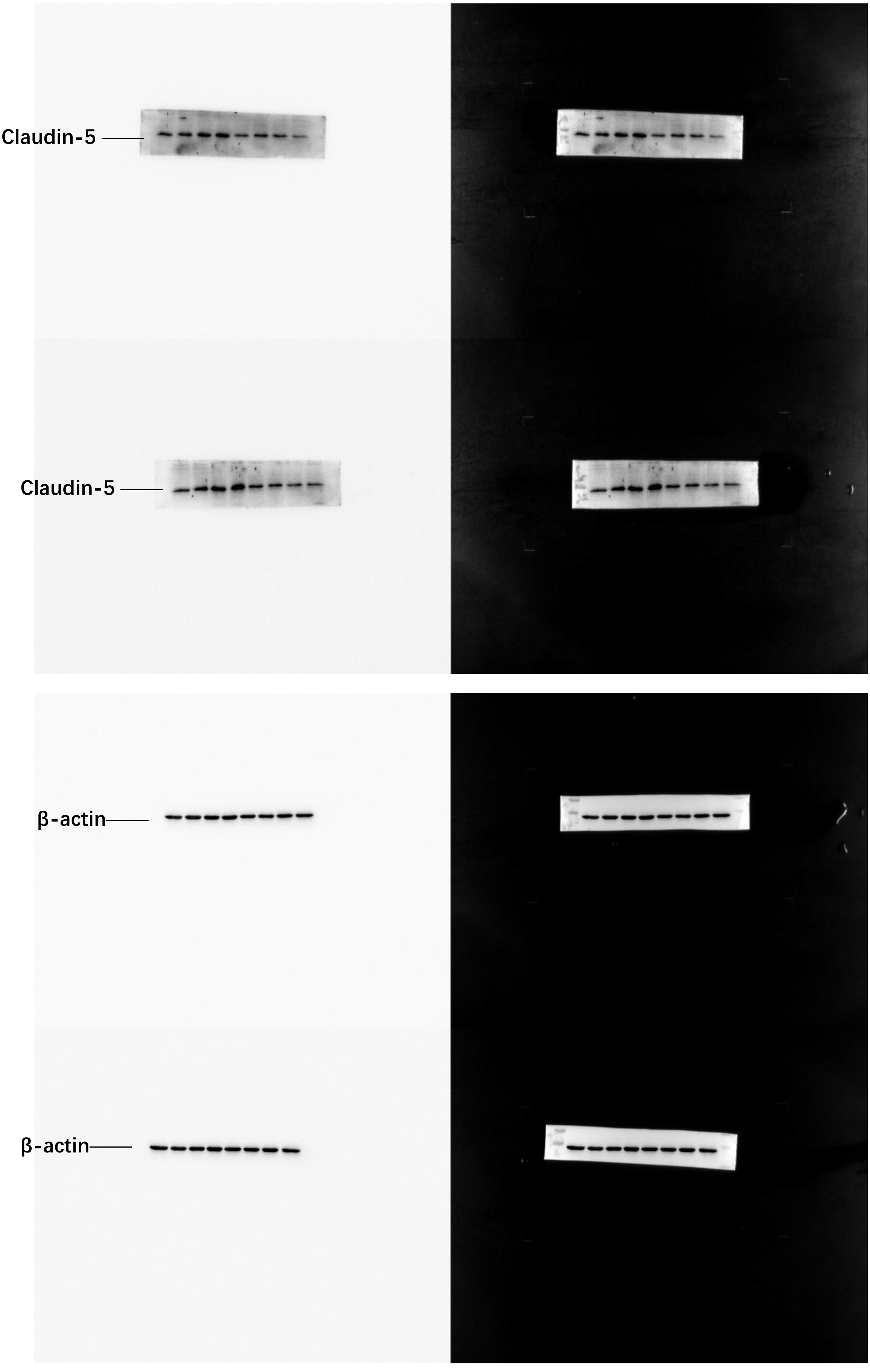

Figure 4E-FOXO1，β-actin

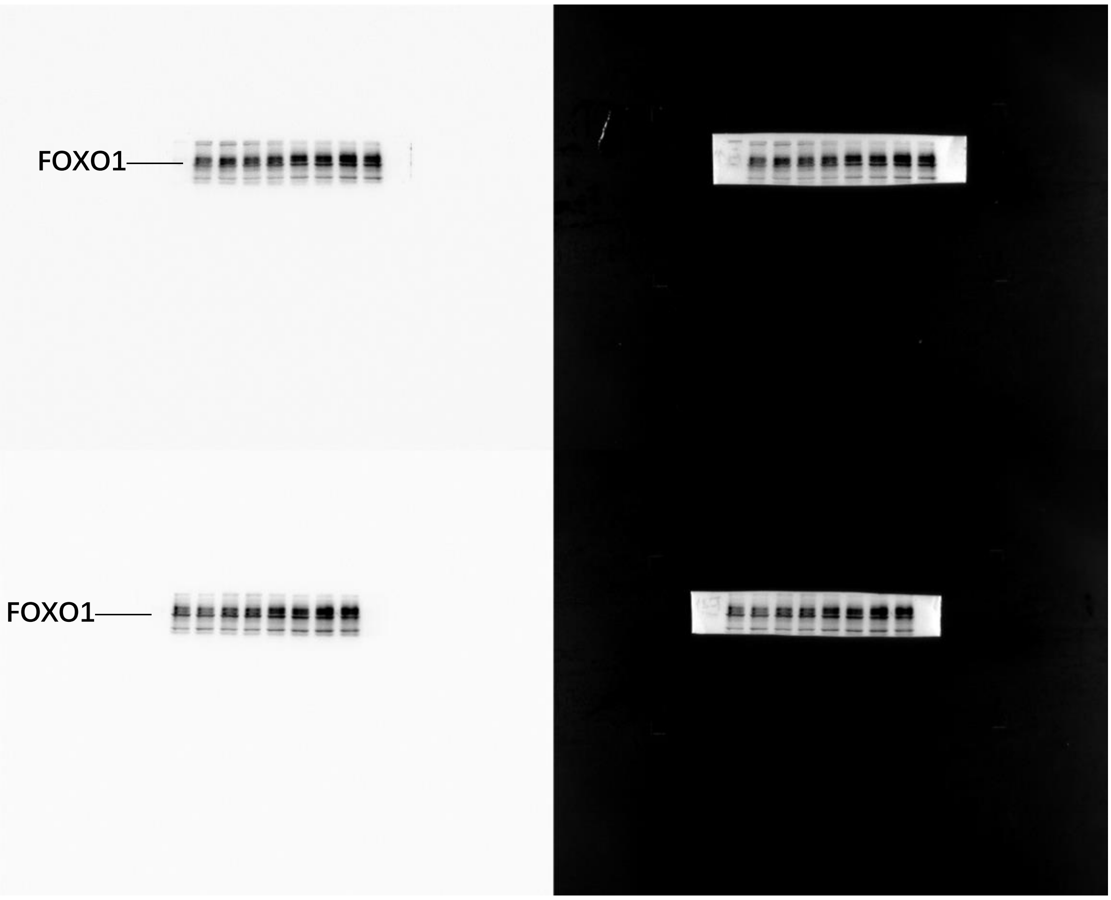

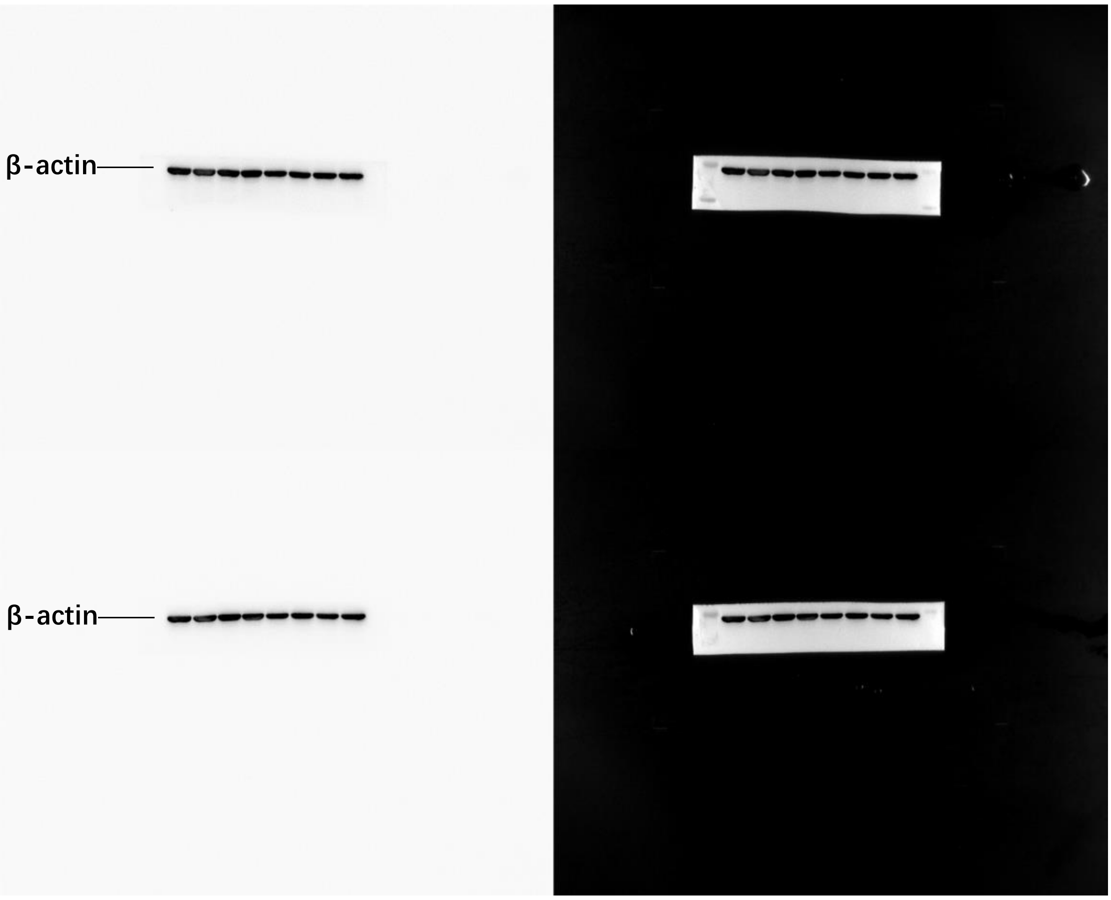

Figure 4E-FOXO1，Lamin B

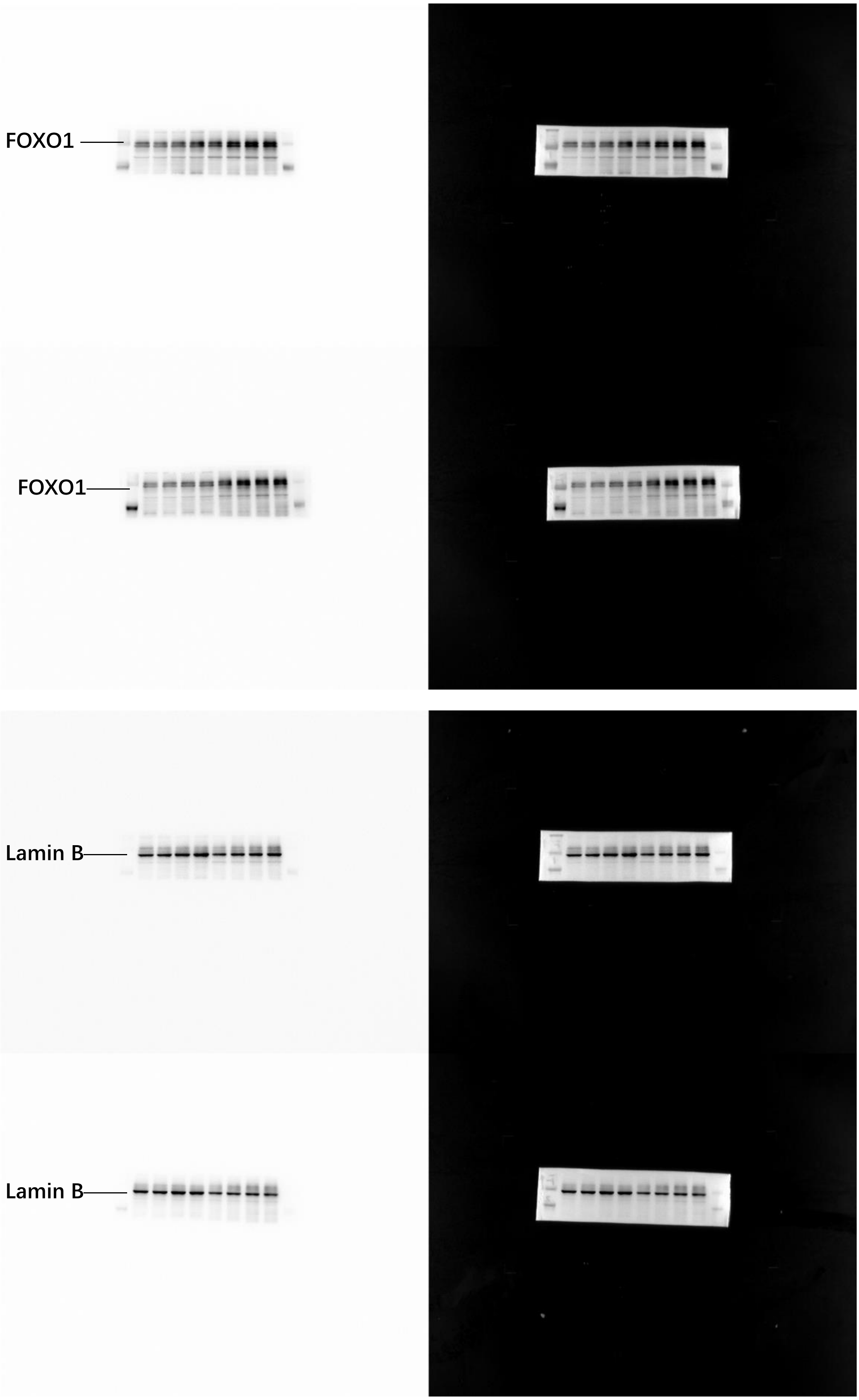

Figure 4F-p-FOXO1/FOXO1

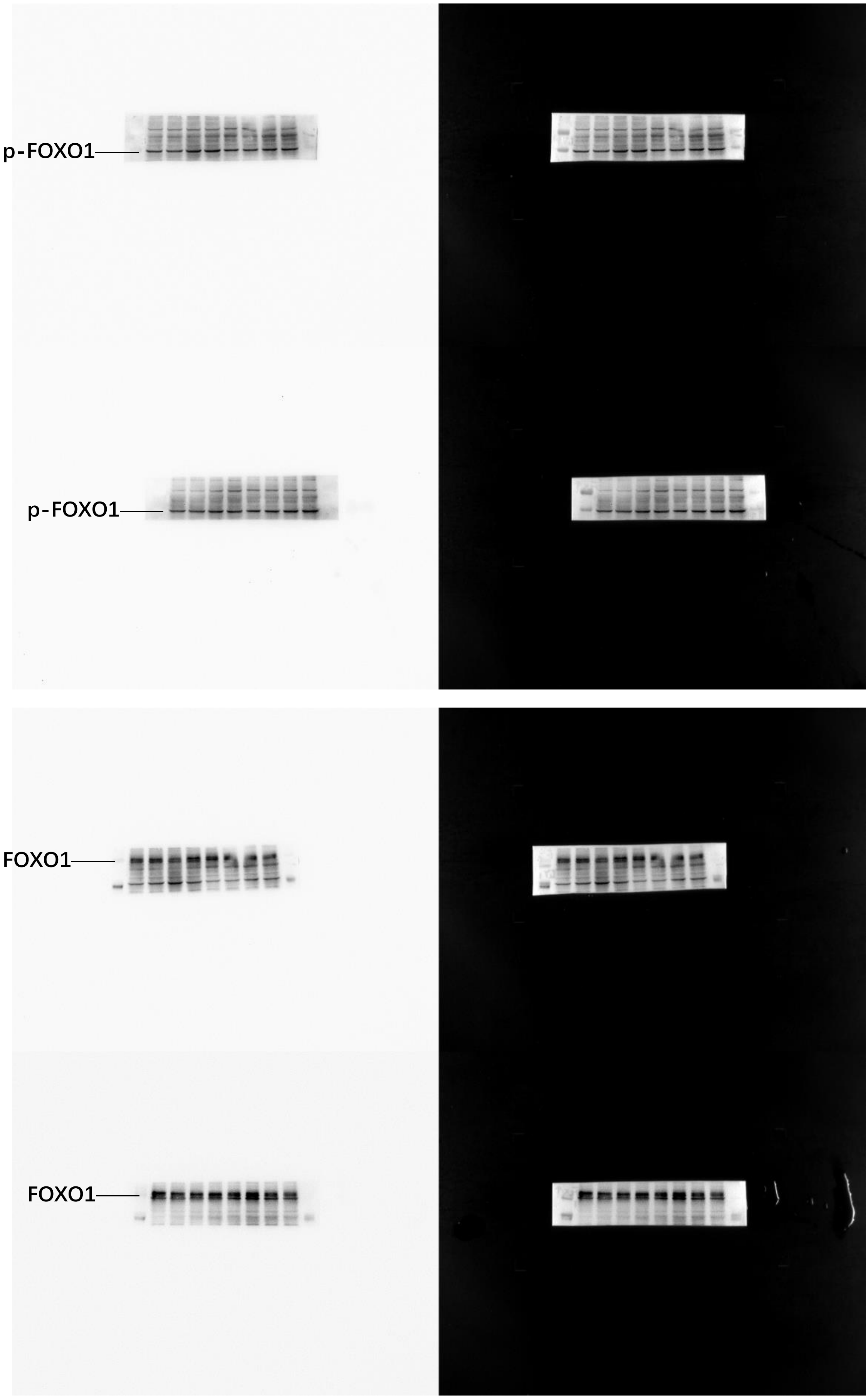

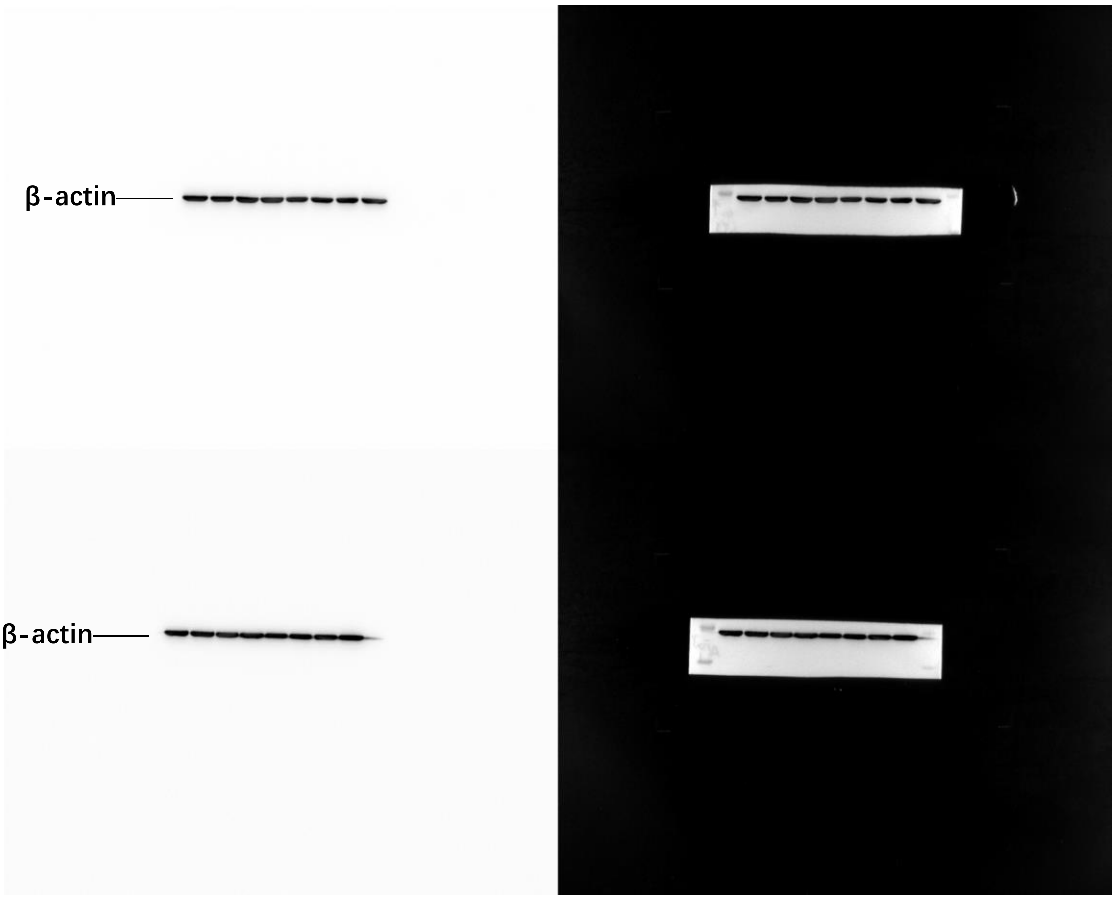
β-actin
β-actin

Figure 4F-p-FOXO1, FOXO1, GAPDH

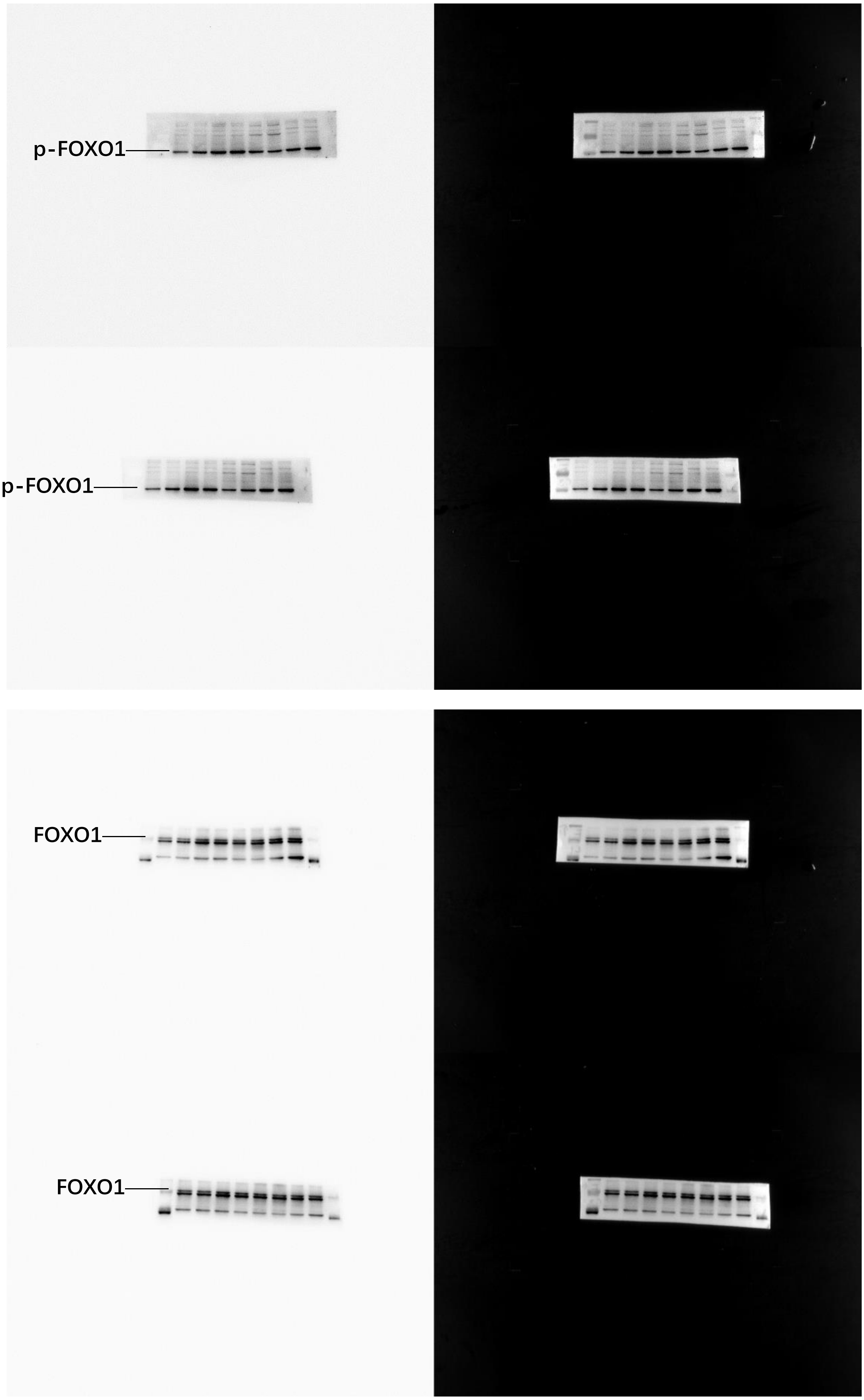

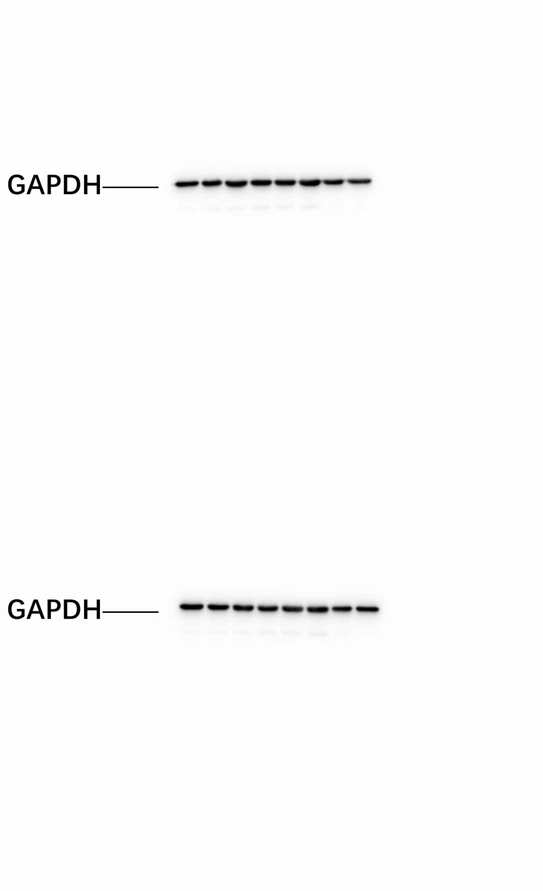
GAPDH
GAPDH

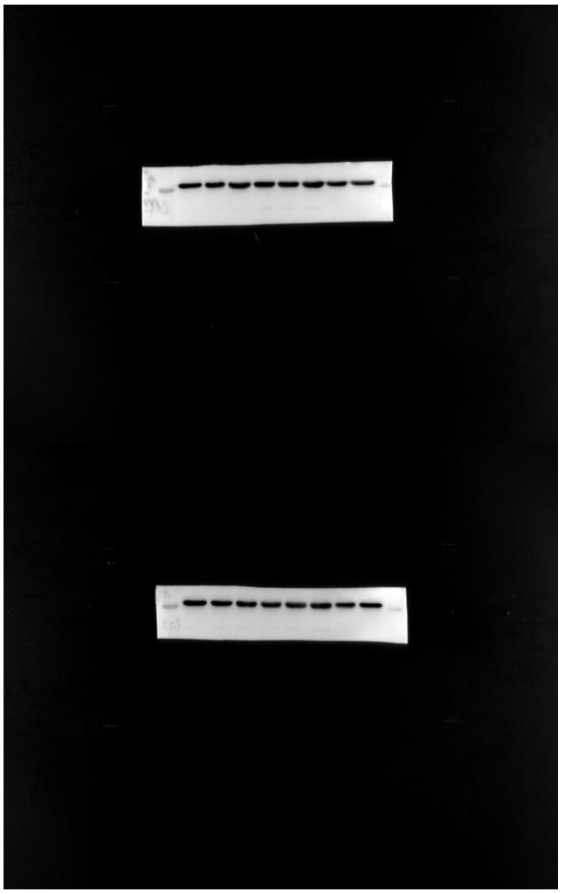

Figure 4F-FOXO1, Lamin B

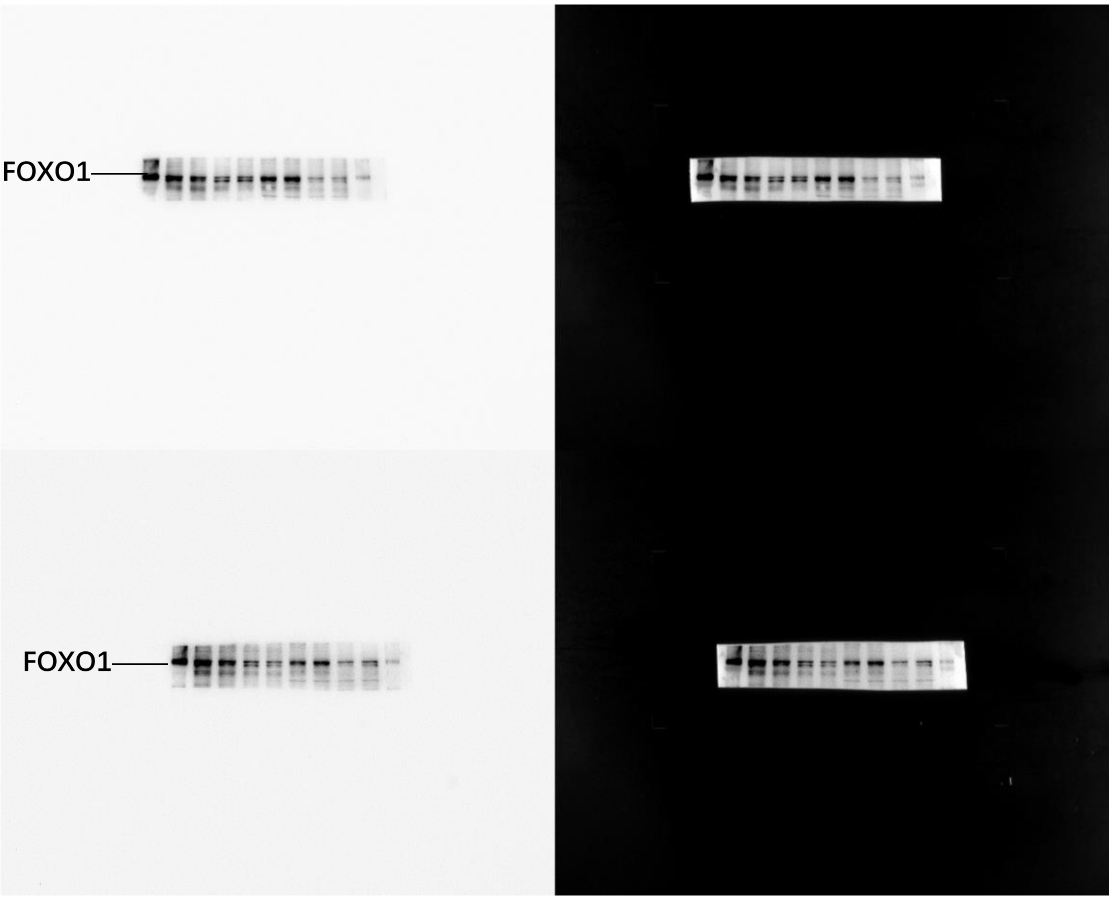

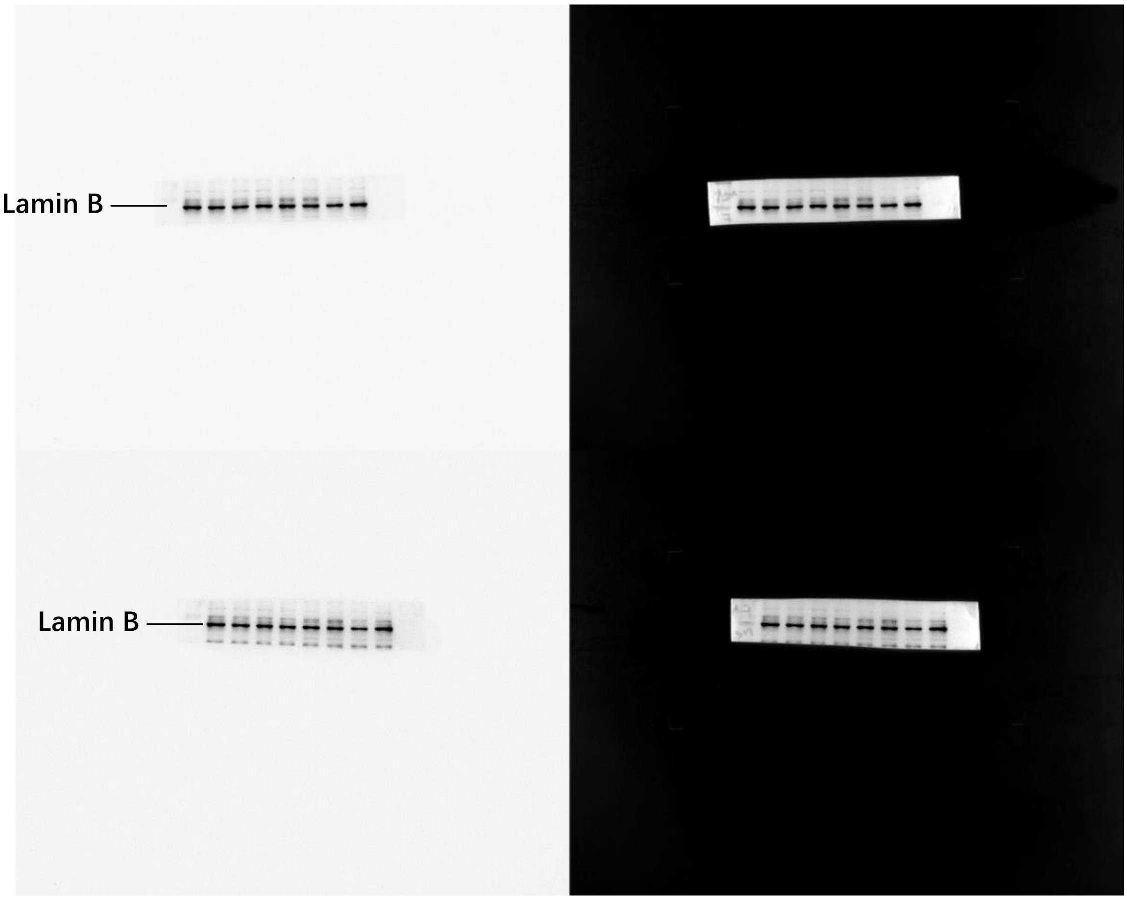

Figure 4G-claudin-5

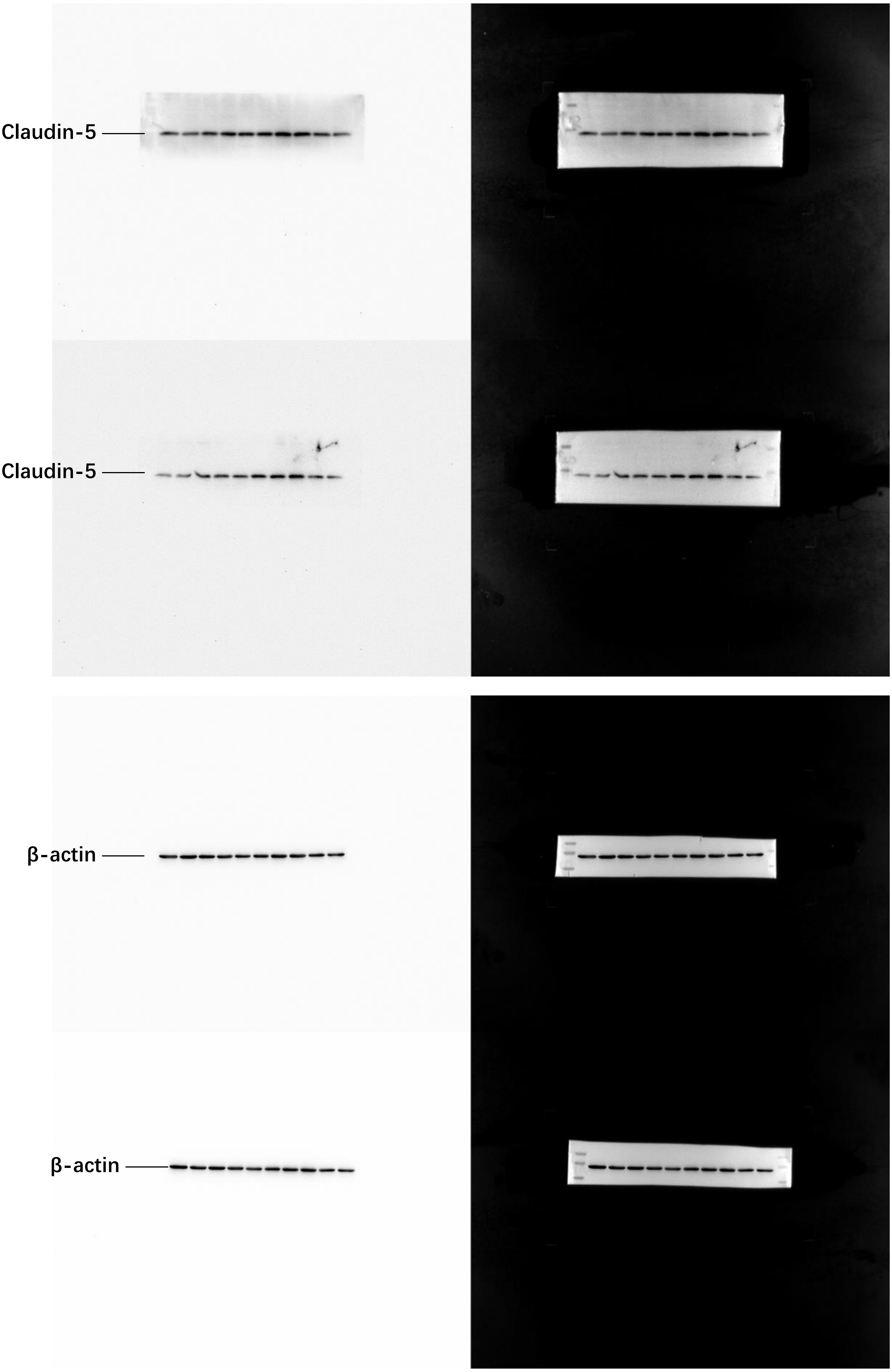

Figure 4G-FOXO1

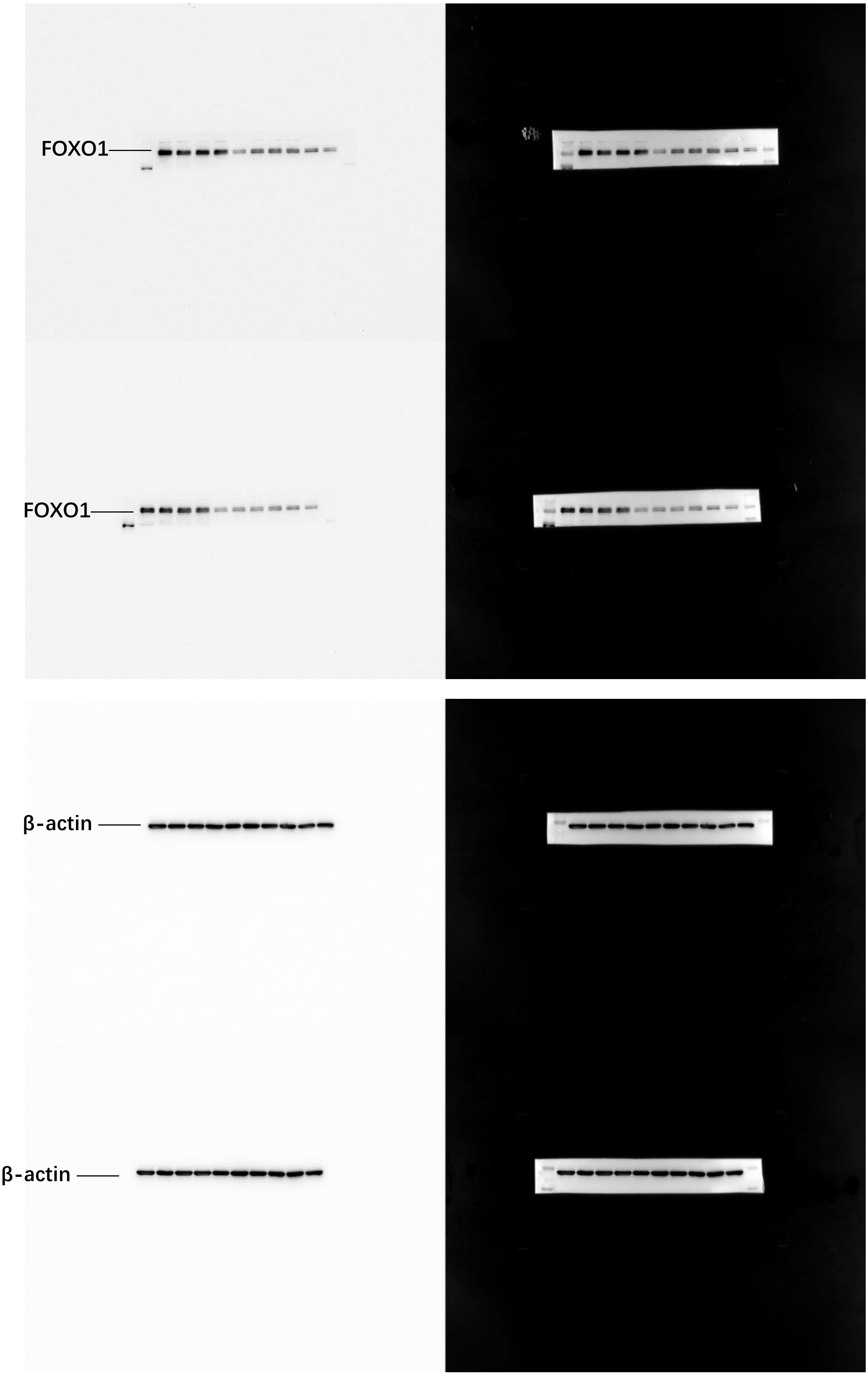

Supplement: Figure 4—source data 2. [file elife-96161-fig4-data2.zip › Figure 4-Source data2/Figure 4-Annotated western blots.pdf]
