# Supplementary material for: Neurons enhance blood–brain barrier function via upregulating claudin-5 and VE-cadherin expression due to glial cell line-derived neurotrophic factor secretion
Source: eLife. 2024 Oct 30;13:RP96161. doi: 10.7554/eLife.96161 (PMC11524583; doi:10.7554/eLife.96161)

Figure 5A-ETS1

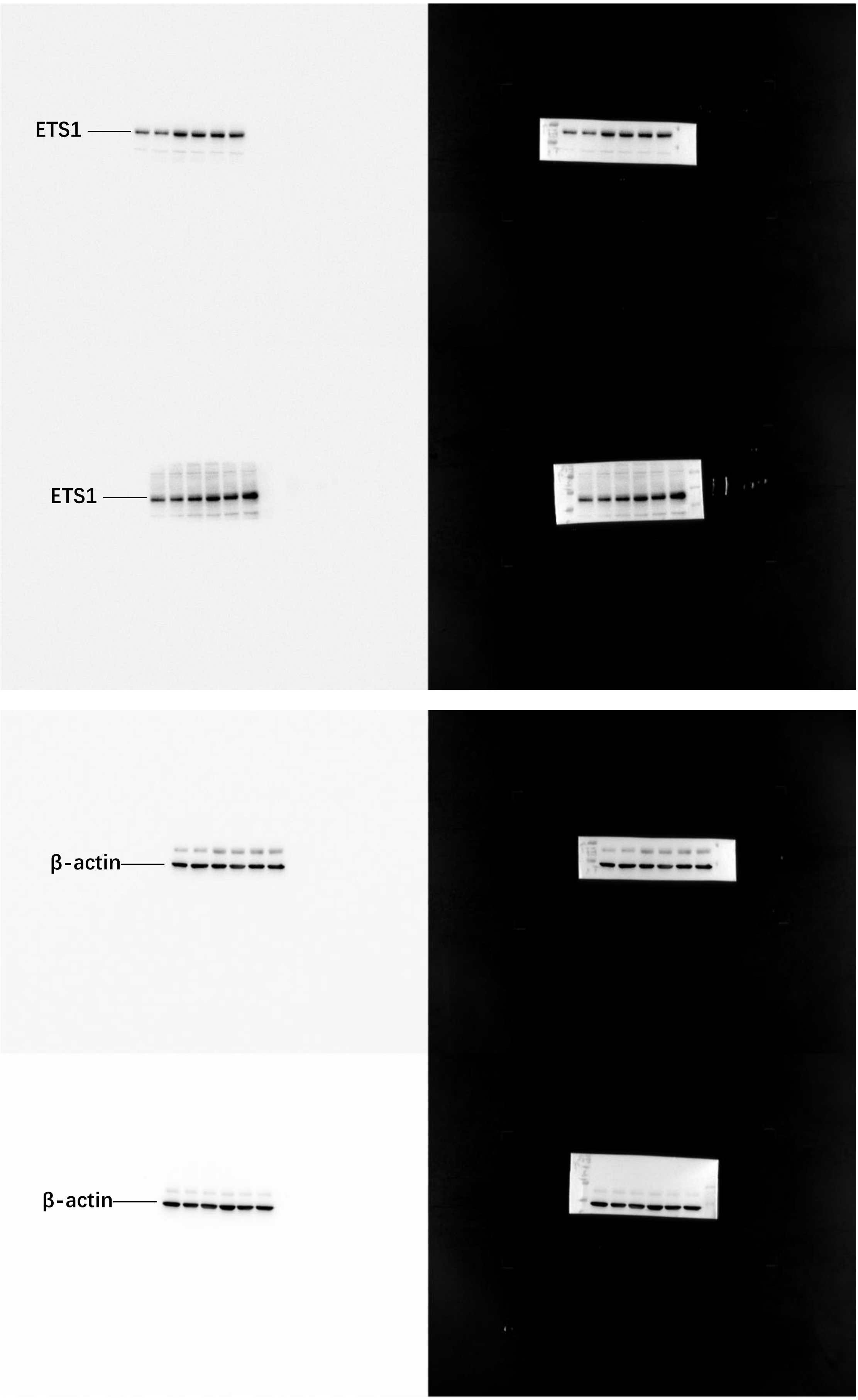

Figure 5B-ETS1

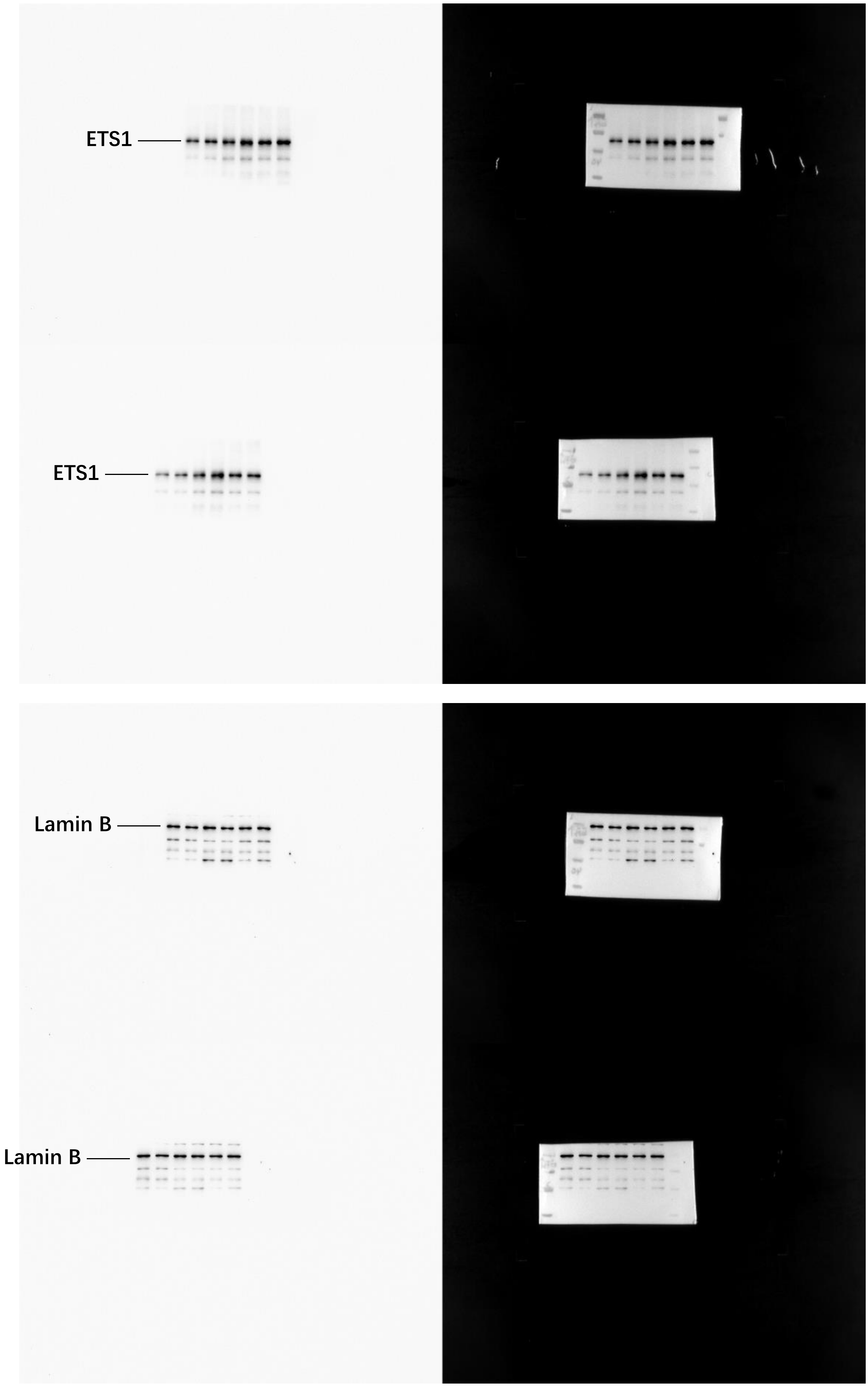

Figure 5C-ETS1

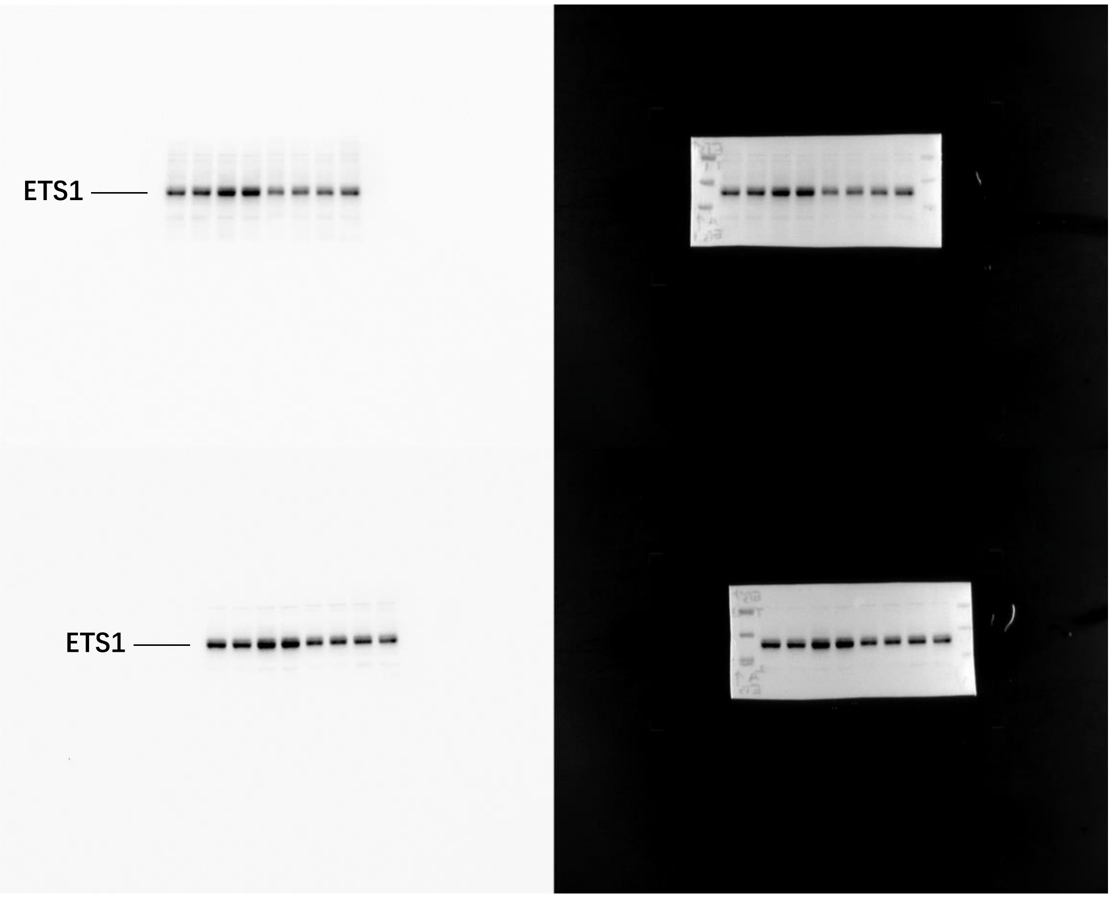

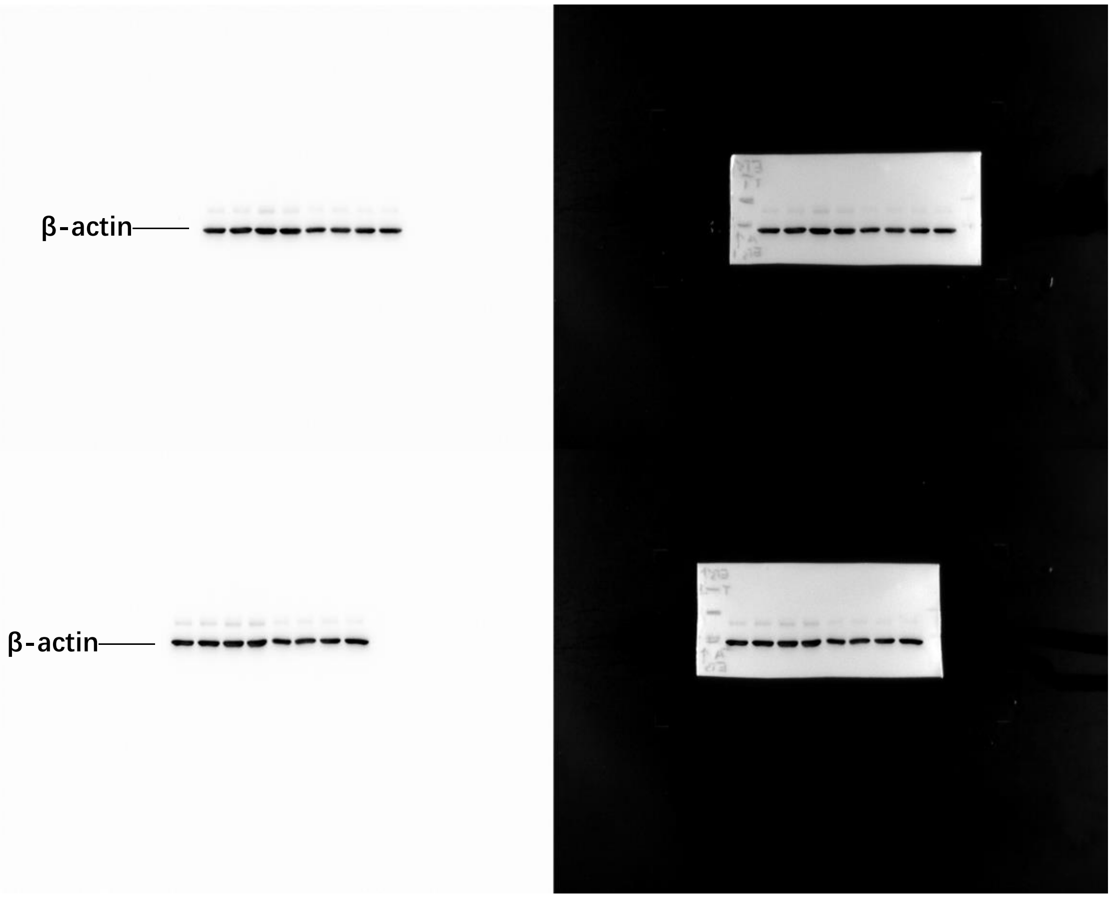

Figure 5D-ETS1

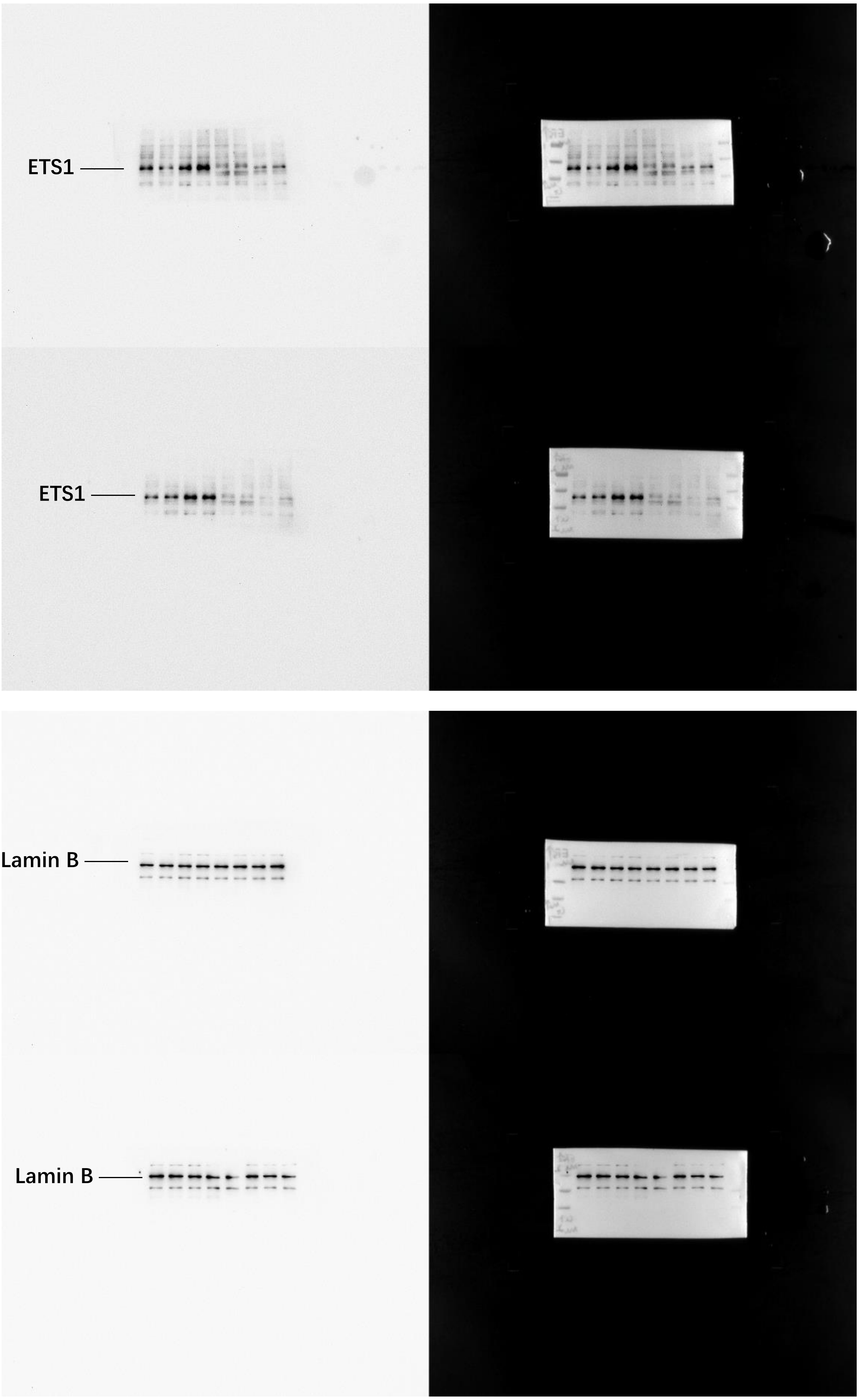

Figure 5E-ETS1

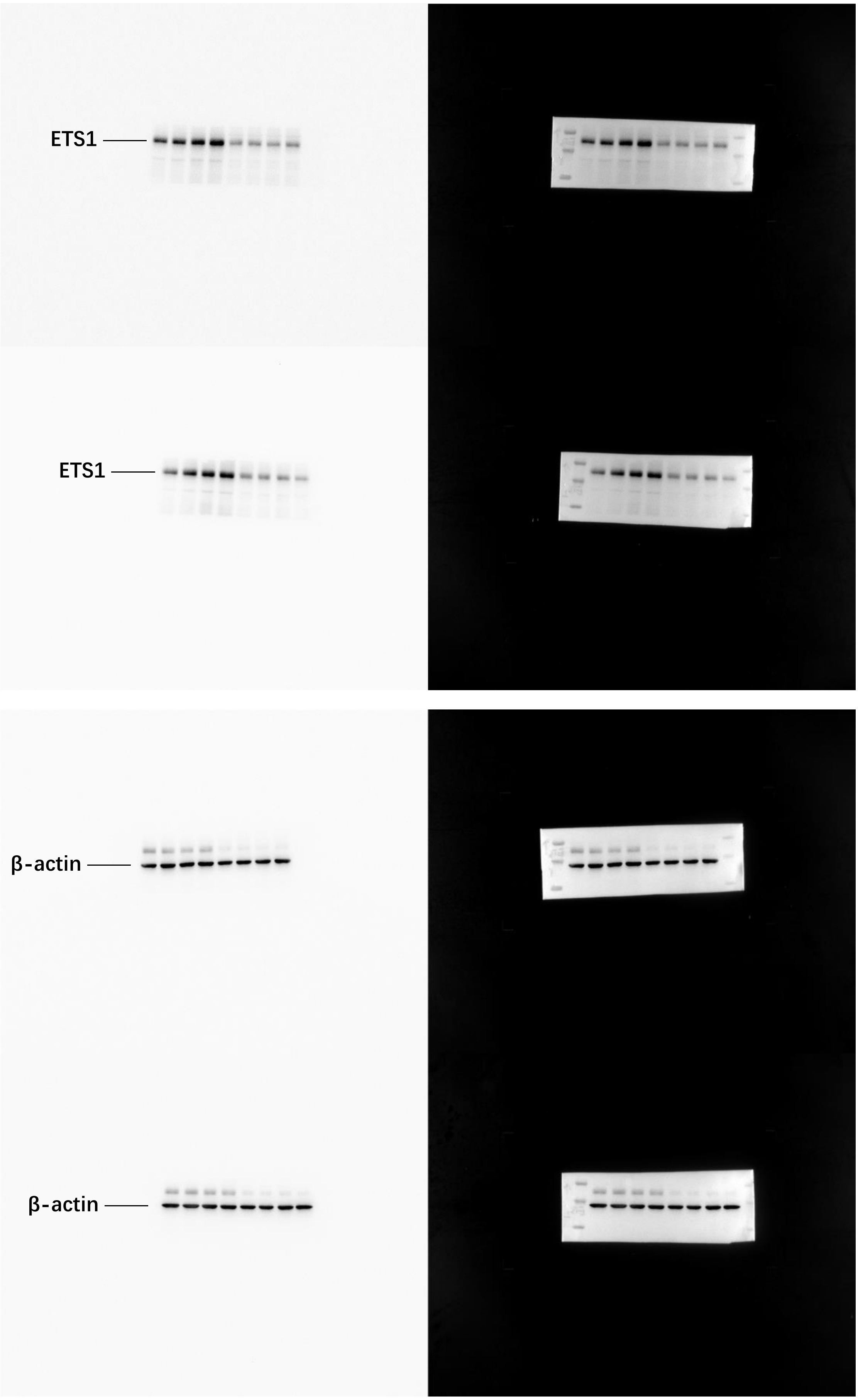

Figure 5F-ETS1

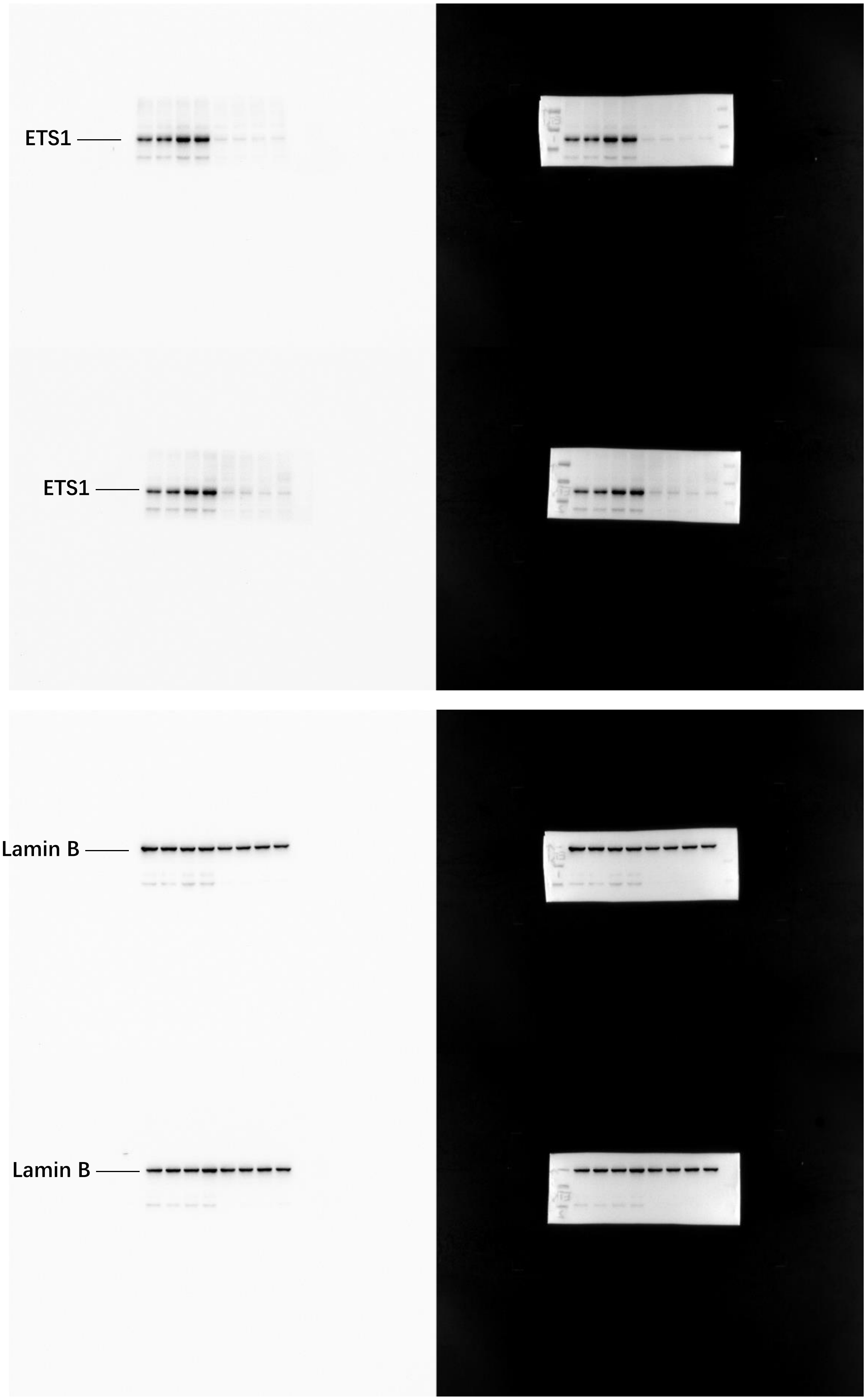

Figure 5G-VE-cadherin

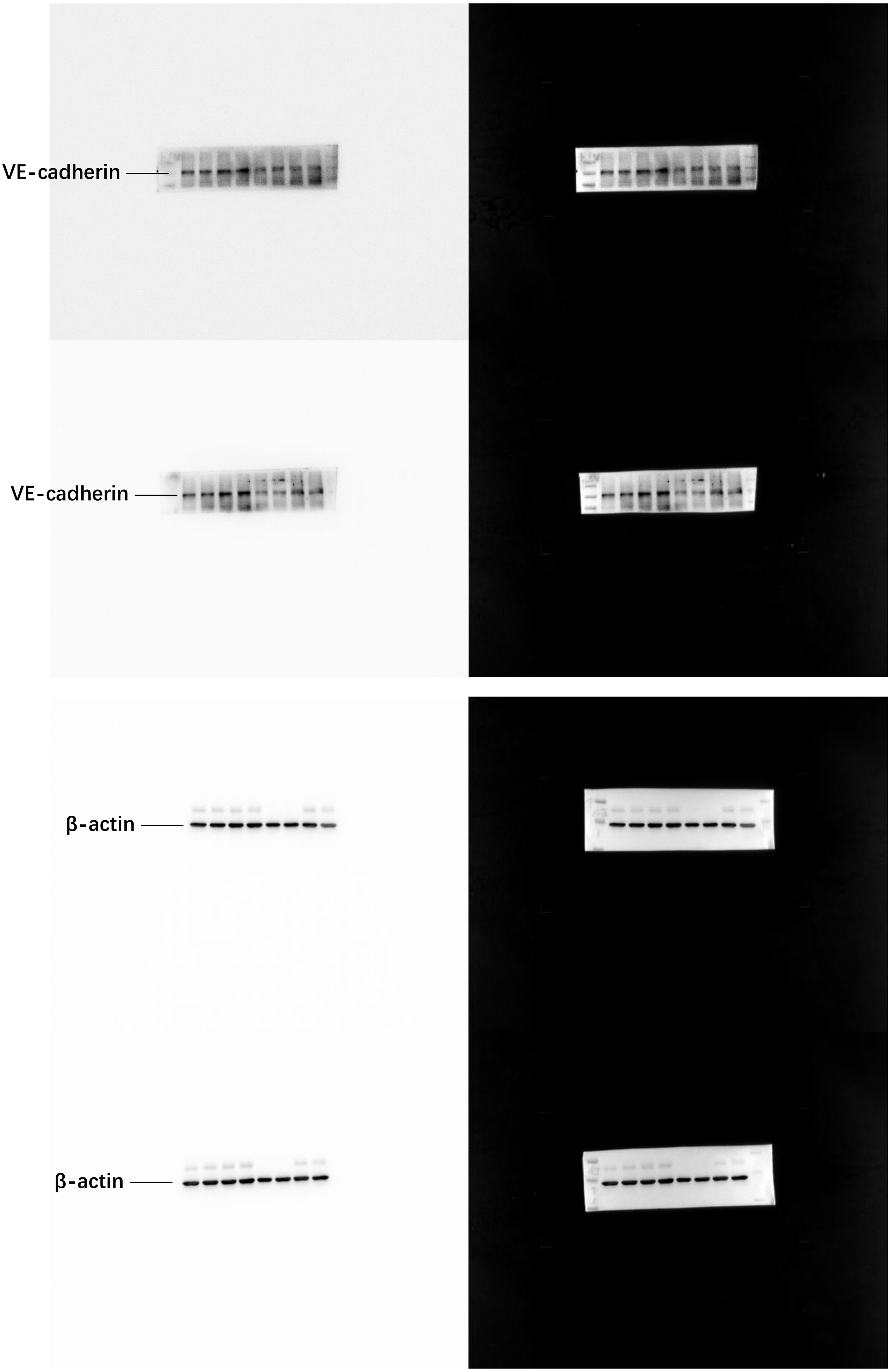

Figure 5G-claudin-5

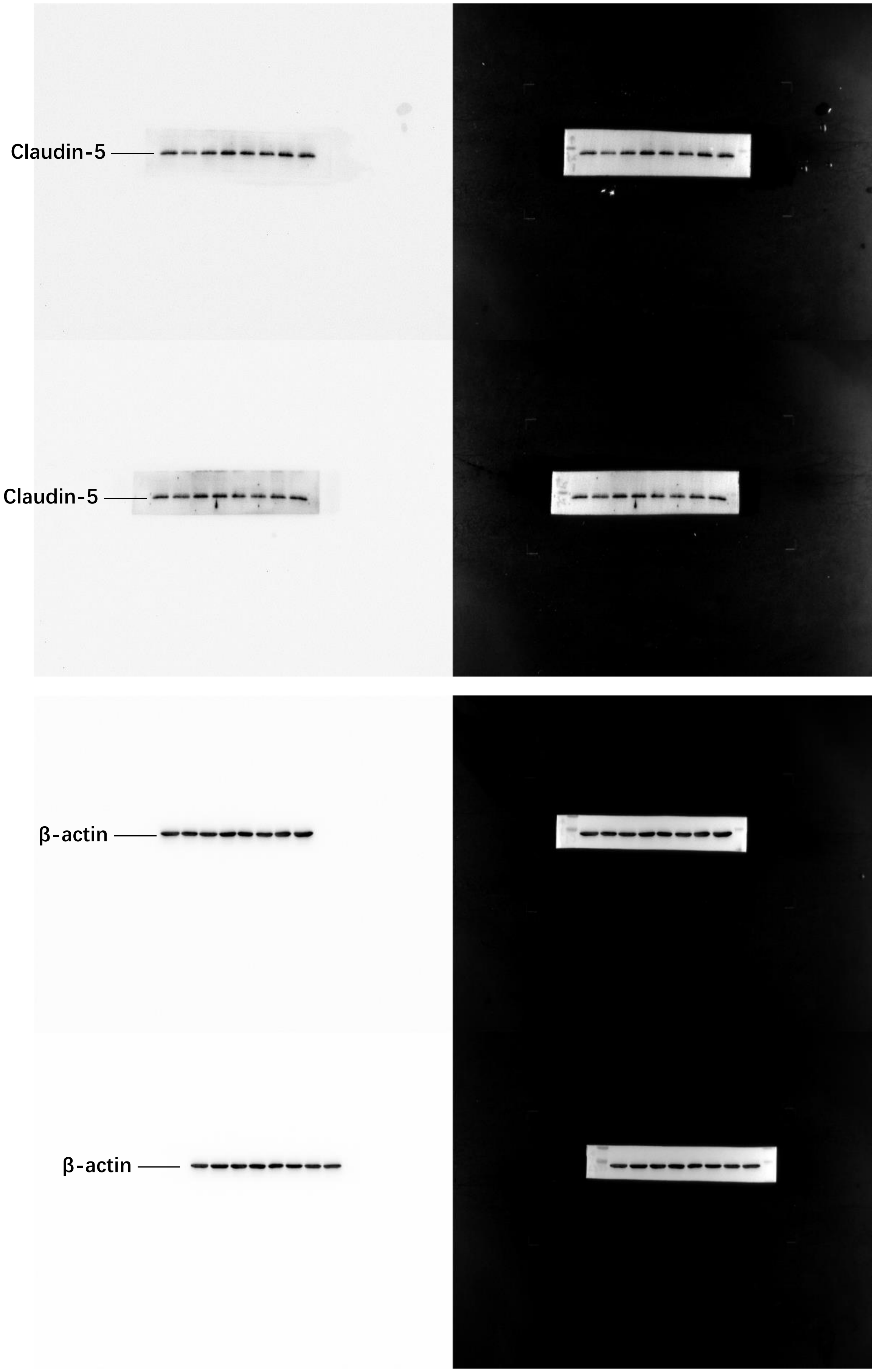

Supplement: Figure 5—source data 2. [file elife-96161-fig5-data2.zip › Figure 5-Source data2/Figure 5-Annotated western blots.pdf]
