# Supplementary material for: Neurons enhance blood–brain barrier function via upregulating claudin-5 and VE-cadherin expression due to glial cell line-derived neurotrophic factor secretion
Source: eLife. 2024 Oct 30;13:RP96161. doi: 10.7554/eLife.96161 (PMC11524583; doi:10.7554/eLife.96161)

Figure 6B-GDNF, claudin-5

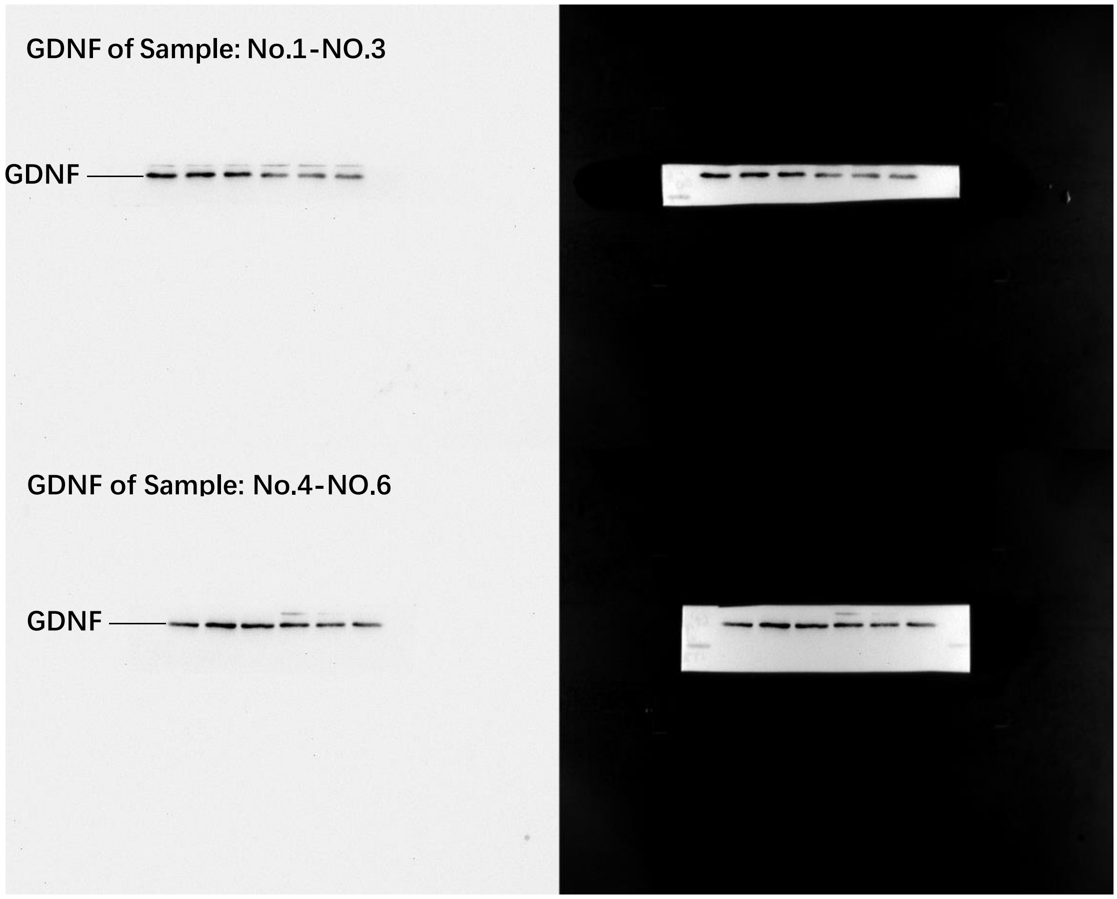

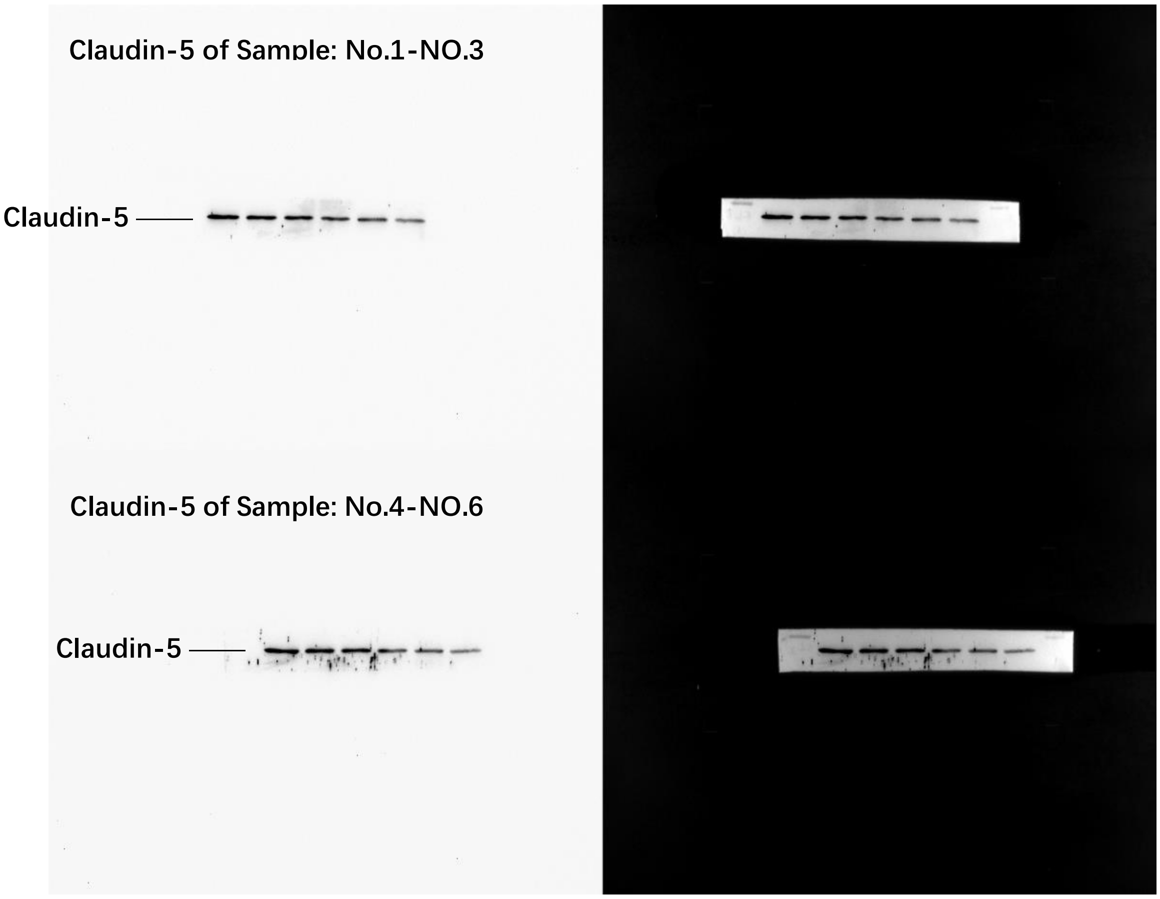

Figure 6B-β-actin

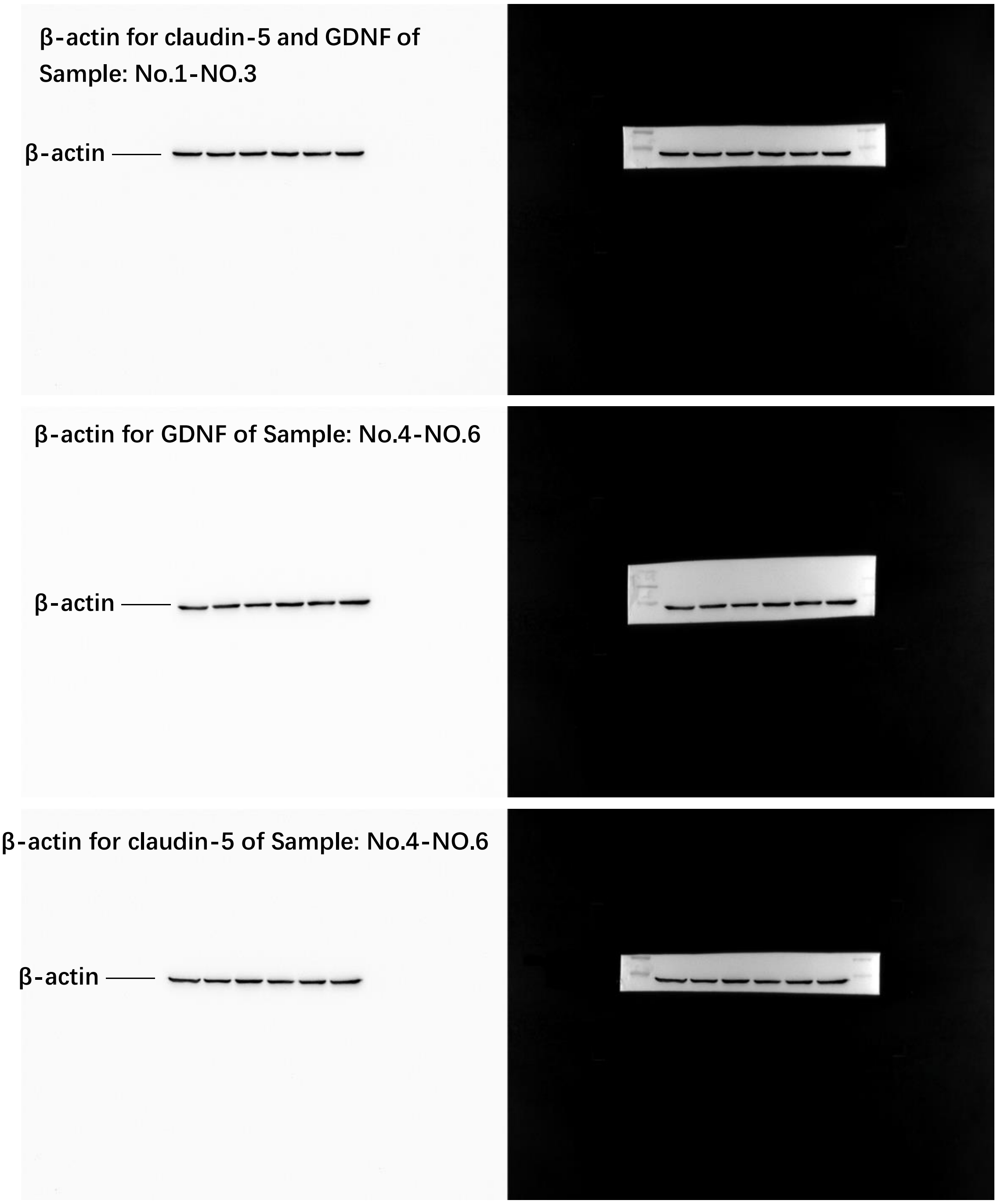

Figure 6B-VE-cadherin

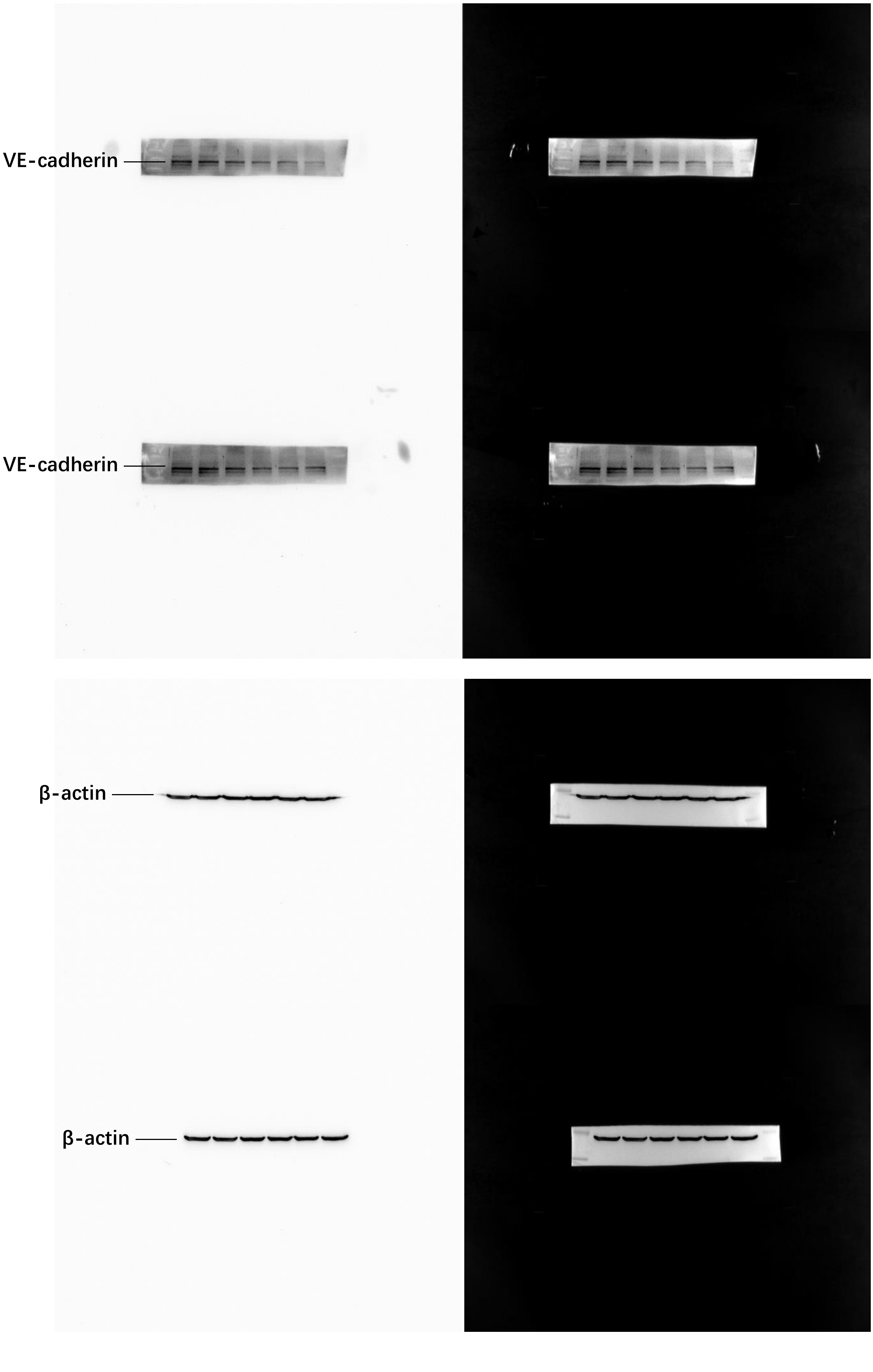

Figure 6I -pAKT/AKT

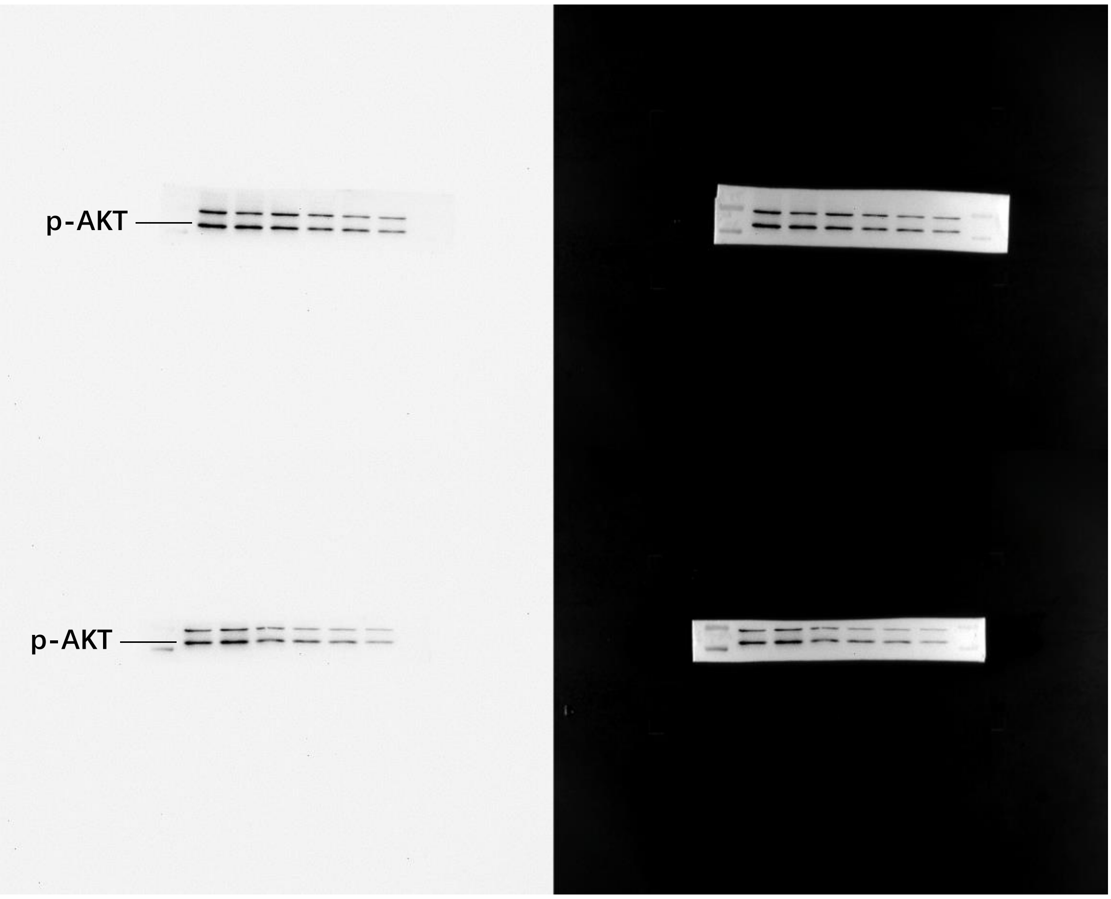

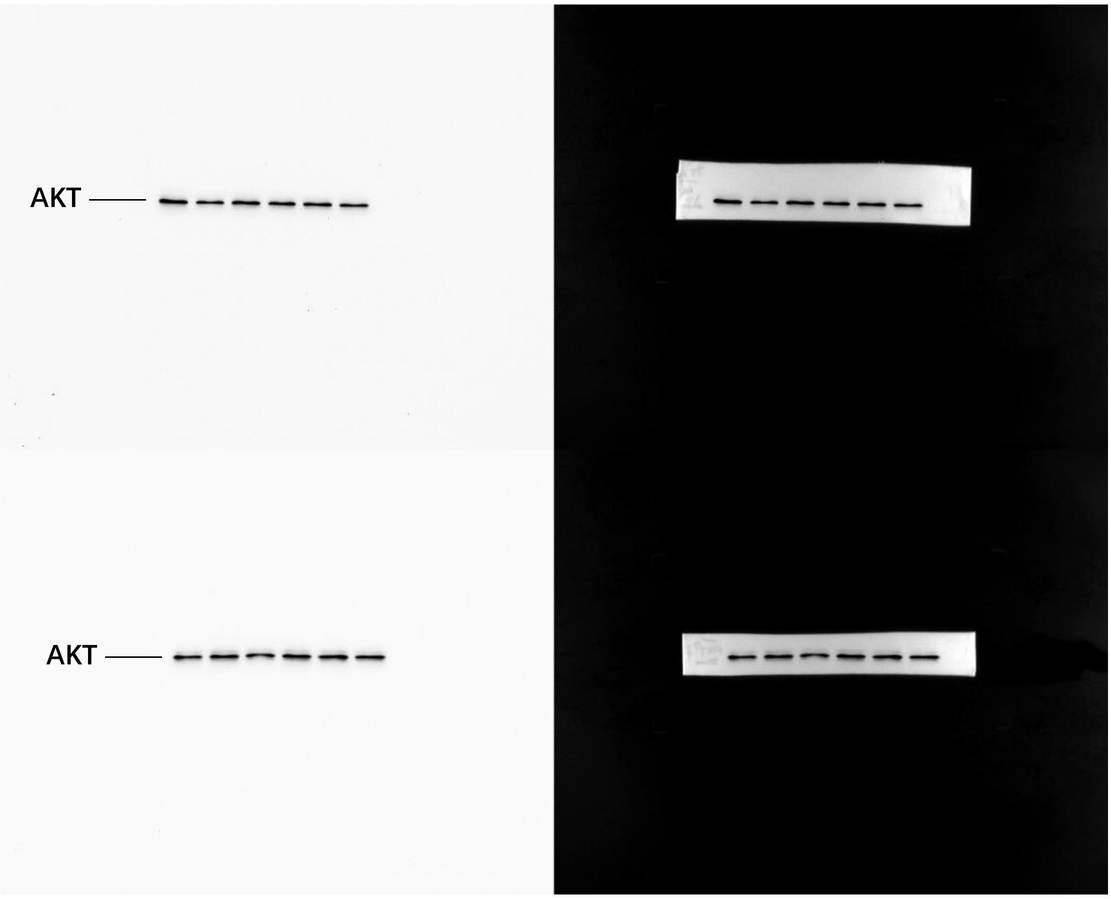

Figure 6J-p-ERK/ERK

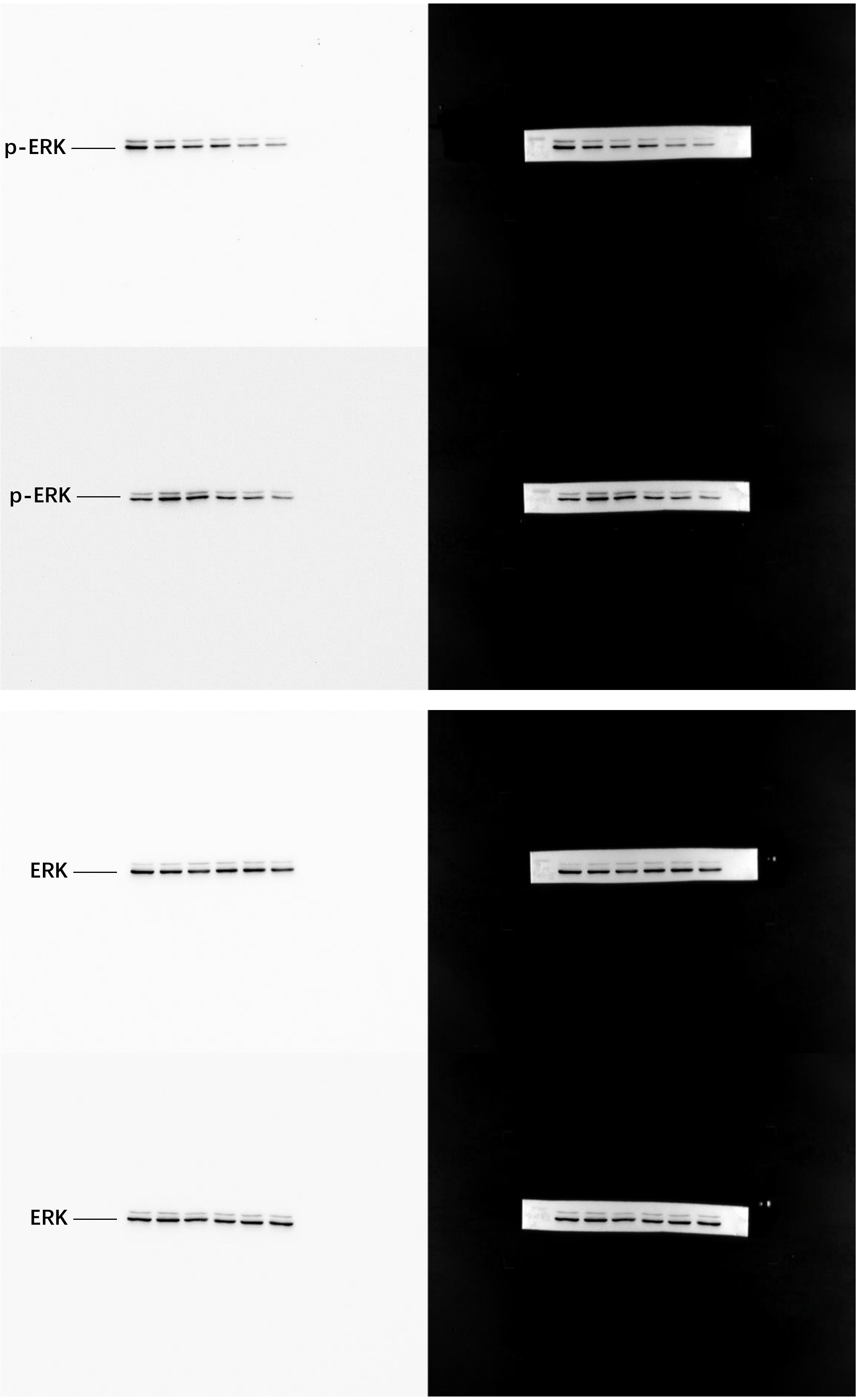

Figure 6I and 6J-β-actin

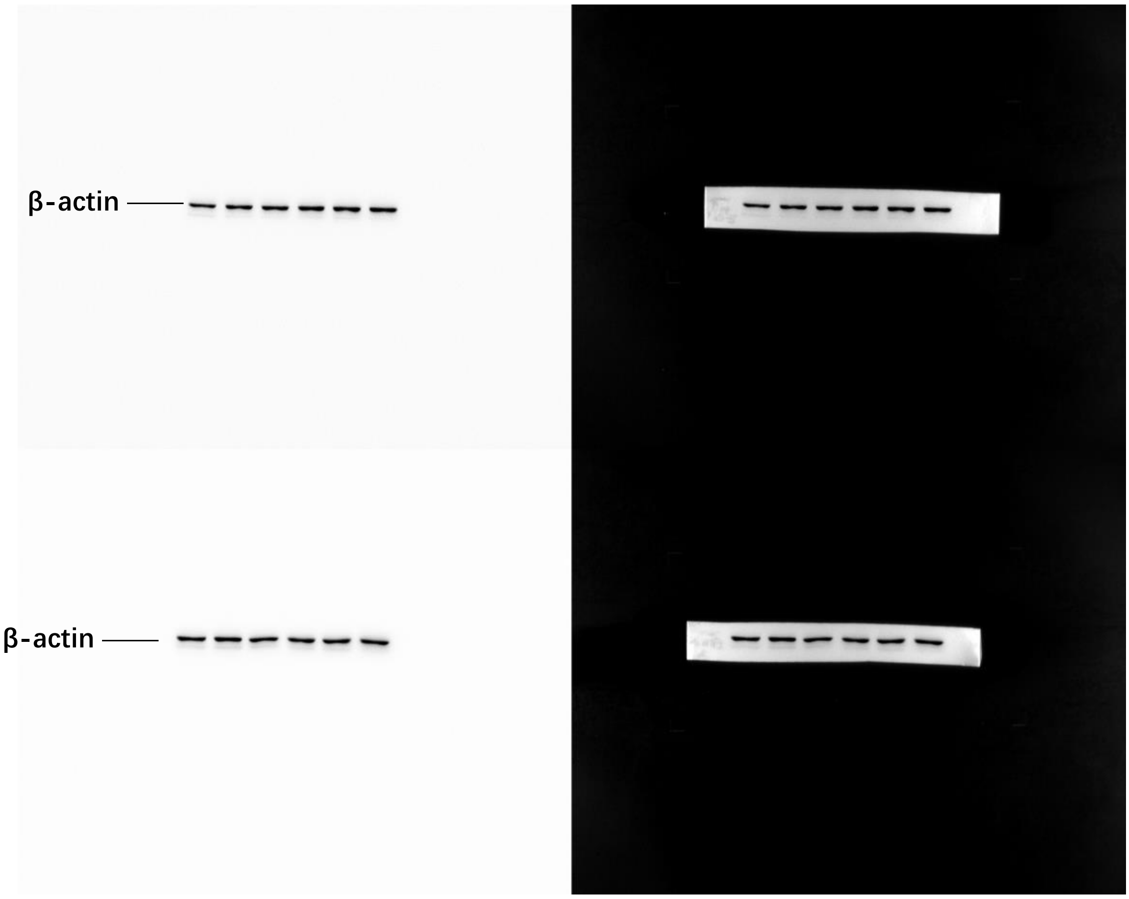

Figure 6K-p-FOXO1/FOXO1

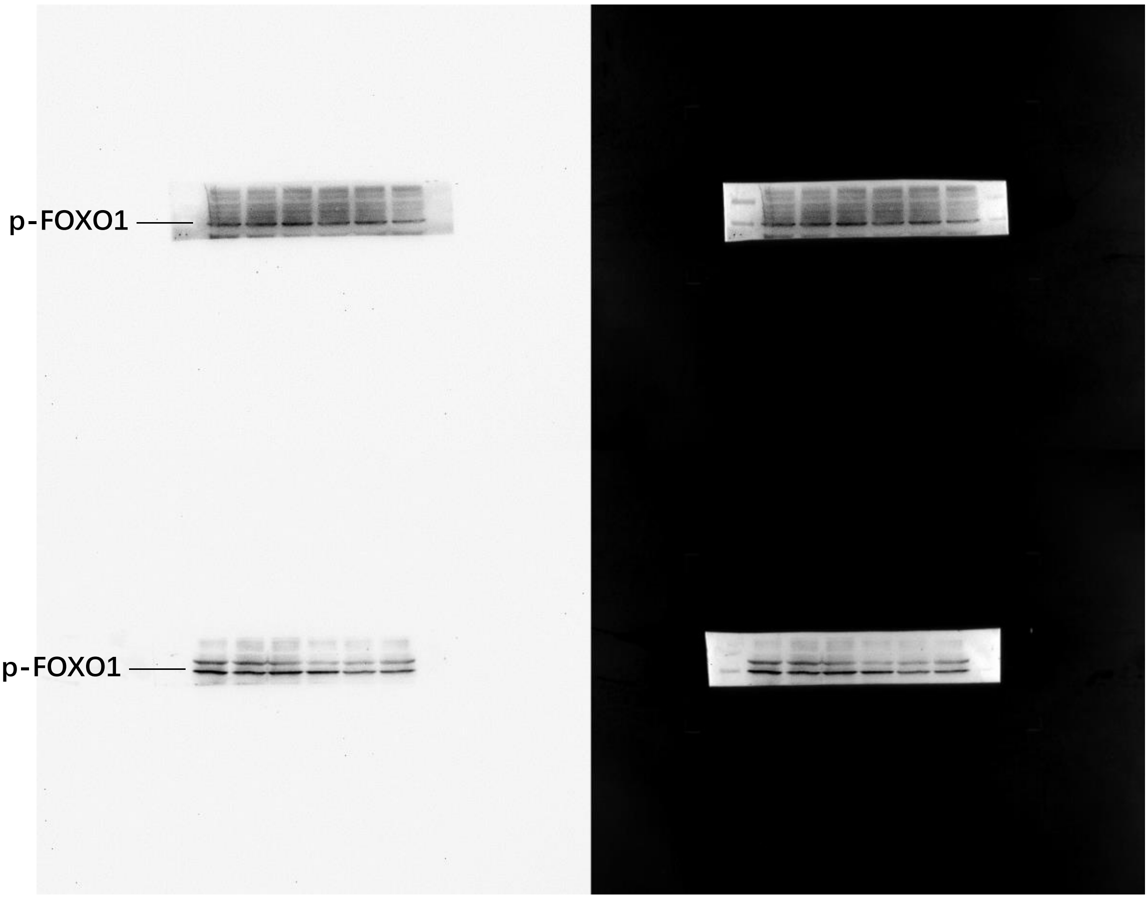

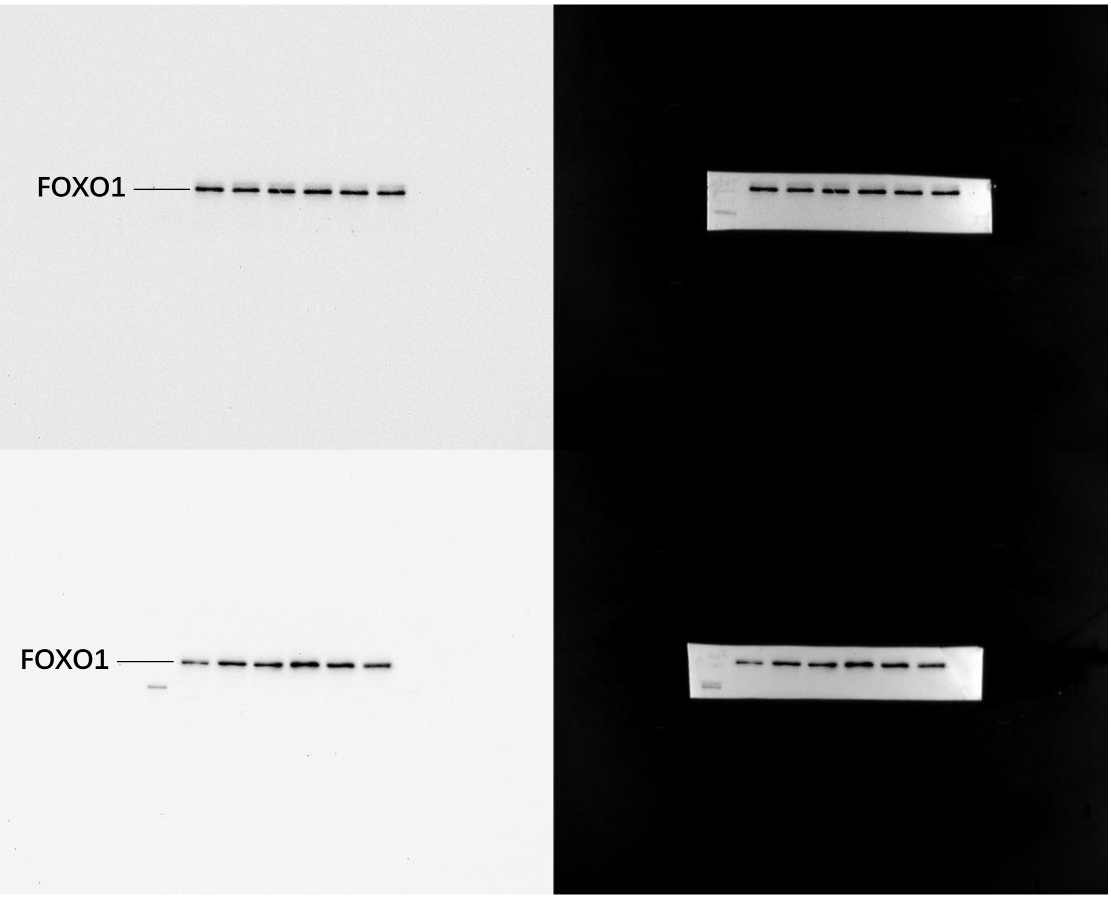

Figure 6K-β-actin

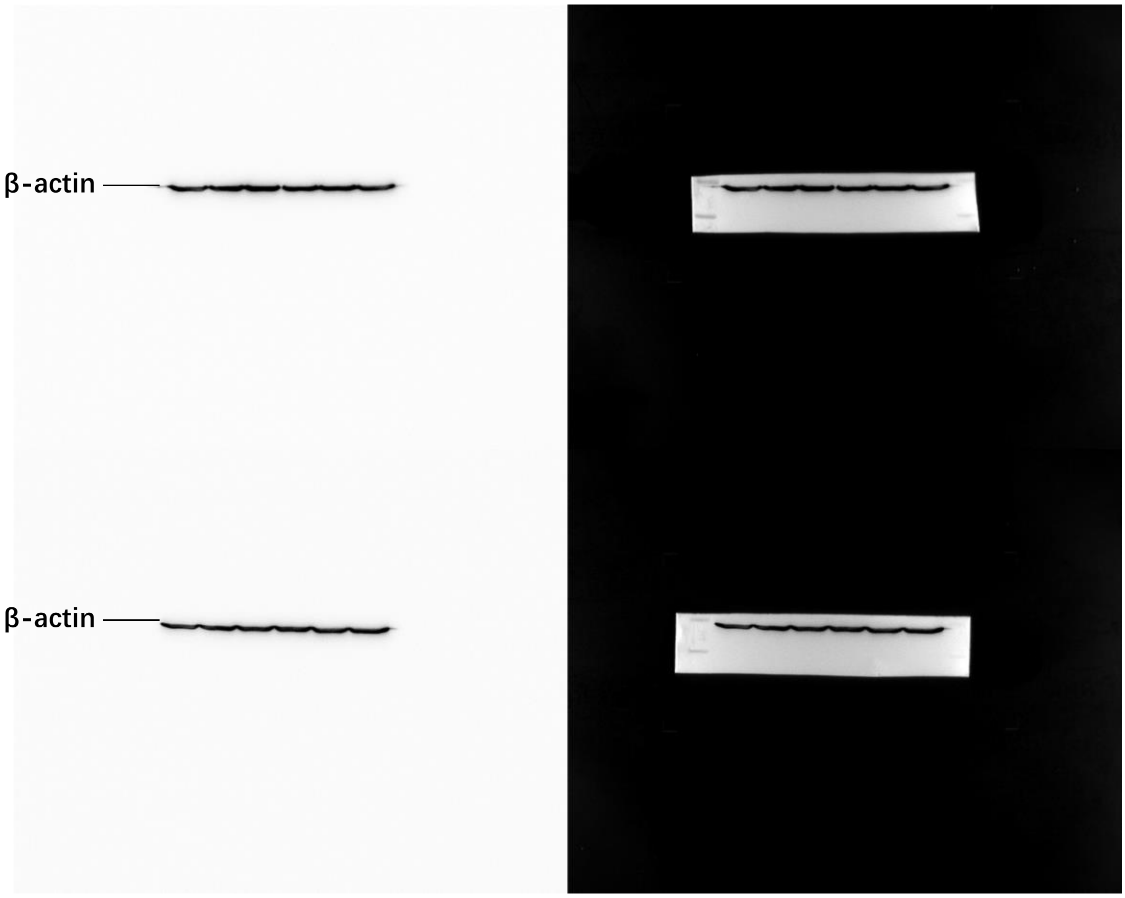

Figure 6K-ETS1

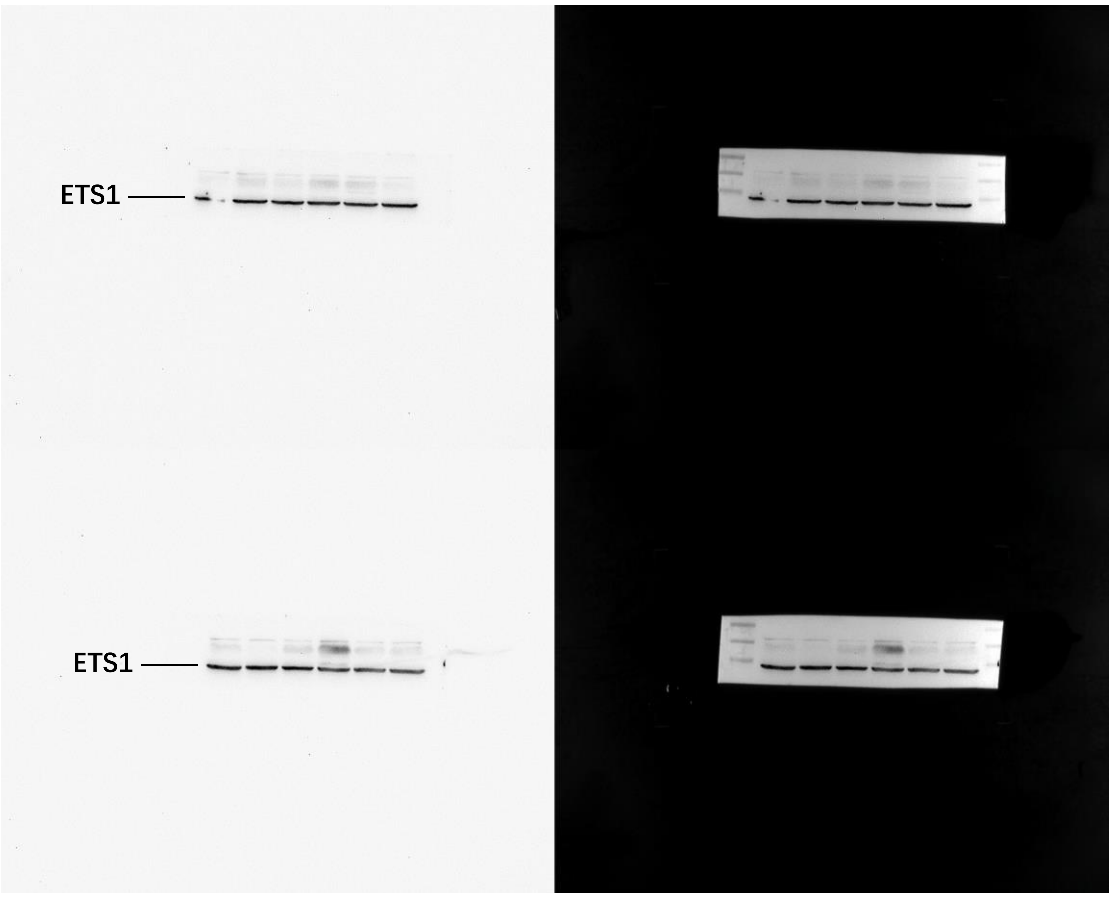

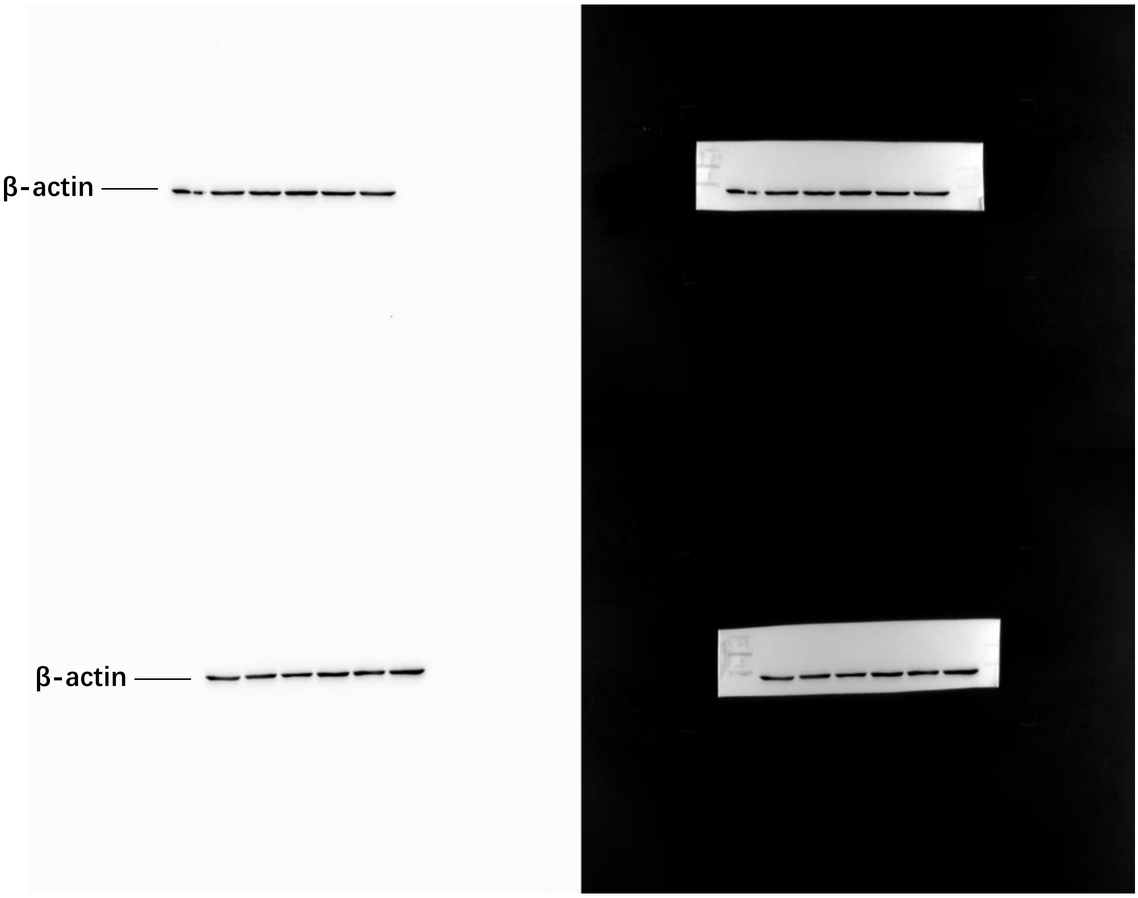

Supplement: Figure 6—source data 2. [file elife-96161-fig6-data2.zip › Figure 6-Source data2/Figure 6-Annotated western blots.pdf]
